# Annotated type catalogue of the Bulimulidae (Mollusca, Gastropoda, Orthalicoidea) in the Natural History Museum, London

**DOI:** 10.3897/zookeys.392.6328

**Published:** 2014-03-21

**Authors:** Abraham S.H. Breure, Jonathan D. Ablett

**Affiliations:** 1 Naturalis Biodiversity Center, P.O. Box 9517, Leiden, the Netherlands; 2 Natural History Museum, Division of Higher Invertebrates, London, SW7 5BD, UK

**Keywords:** Bulimulidae, types

## Abstract

The type status is described of 404 taxa classified within the family Bulimulidae (superfamily Orthalicoidea) and kept in the London museum. Lectotypes are designated for *Bulimus aurifluus* Pfeiffer, 1857; *Otostomus bartletti* H. Adams, 1867; *Helix cactorum* d’Orbigny, 1835; *Bulimus caliginosus* Reeve, 1849; *Bulimus chemnitzioides* Forbes, 1850; *Bulimus cinereus* Reeve, 1849; *Helix cora* d’Orbigny, 1835; *Bulimus fallax* Pfeiffer, 1853; *Bulimus felix* Pfeiffer, 1862; *Bulimus fontainii* d’Orbigny, 1838; *Bulimus fourmiersi* d’Orbigny, 1837; *Bulimus (Mesembrinus) gealei* H. Adams, 1867; *Bulimus gruneri* Pfeiffer, 1846; *Bulimus humboldtii* Reeve, 1849; *Helix hygrohylaea* d’Orbigny, 1835; *Bulimus jussieui* Pfeiffer, 1846; *Bulimulus (Drymaeus) binominis lascellianus* E.A. Smith, 1895; *Helix lichnorum* d’Orbigny, 1835; *Bulimulus (Drymaeus) lucidus* da Costa, 1898; *Bulimus luridus* Pfeiffer, 1863; *Bulimus meleagris* Pfeiffer, 1853; *Bulimus monachus* Pfeiffer, 1857; *Bulimus montagnei* d’Orbigny, 1837; *Helix montivaga* d’Orbigny, 1835; *Bulimus muliebris* Reeve, 1849; *Bulimus nigrofasciatus* Pfeiffer in Philippi 1846; *Bulimus nitelinus* Reeve, 1849; *Helix oreades* d’Orbigny, 1835; *Helix polymorpha* d’Orbigny, 1835; *Bulimus praetextus* Reeve, 1849; *Bulinus proteus* Broderip, 1832; *Bulimus rusticellus* Morelet, 1860; *Helix sporadica* d’Orbigny, 1835; *Bulimus sulphureus* Pfeiffer, 1857; *Helix thamnoica* var. *marmorata* d’Orbigny, 1835; *Bulinus translucens* Broderip in Broderip and Sowerby I 1832; *Helix trichoda* d’Orbigny, 1835; *Bulinus ustulatus* Sowerby I, 1833; *Bulimus voithianus* Pfeiffer, 1847; *Bulimus yungasensis* d’Orbigny, 1837.

The type status of the following taxa is changed to lectotype in accordance with Art. 74.6 ICZN: *Bulimulus (Drymaeus) caucaensis* da Costa, 1898; *Drymaeus exoticus* da Costa, 1901; *Bulimulus (Drymaeus) hidalgoi* da Costa, 1898; *Bulimulus (Drymaeus) interruptus* Preston, 1909; *Bulimulus (Drymaeus) inusitatus* Fulton, 1900; *Bulimulus latecolumellaris* Preston, 1909; *Bulimus (Otostomus) napo* Angas, 1878; *Drymaeus notabilis* da Costa, 1906; *Drymaeus notatus* da Costa, 1906; *Bulimulus (Drymaeus) nubilus* Preston, 1903; *Drymaeus obliquistriatus* da Costa, 1901; *Bulimus (Drymaeus) ochrocheilus* E.A. Smith, 1877; *Bulimus (Drymaeus) orthostoma* E.A. Smith, 1877; *Drymaeus expansus perenensis* da Costa, 1901; *Bulimulus pergracilis* Rolle, 1904; *Bulimulus (Drymaeus) plicatoliratus* da Costa, 1898; *Drymaeus prestoni* da Costa, 1906; *Drymaeus punctatus* da Costa, 1907; *Bulimus (Leptomerus) sanctaeluciae* E.A. Smith, 1889; *Bulimulus (Drymaeus) selli* Preston, 1909; *Drymaeus subventricosus* da Costa, 1901; *Bulimulus (Drymaeus) tigrinus* da Costa, 1898; *Drymaeus volsus* Fulton, 1907; *Drymaeus wintlei* Finch, 1929; *Bulimus zhorquinensis* Angas, 1879; *Bulimulus (Drymaeus) ziczac* da Costa, 1898.

The following junior subjective synonyms are established: *Bulimus antioquensis* Pfeiffer, 1855 = *Bulimus baranguillanus* Pfeiffer, 1853; *Drymaeus bellus* da Costa, 1906 = *Drymaeus blandi* Pilsbry, 1897; *Bulimus hachensis* Reeve 1850 = *Bulimus gruneri* Pfeiffer, 1846 = *Bulimus columbianus* Lea, 1838; *Bulimus (Otostomus) lamas* Higgins 1868 = *Bulimus trujillensis* Philippi, 1867; *Bulimulus (Drymaeus) binominis lascellianus* E.A. Smith, 1895 = *Bulimulus (Drymaeus) binominis* E.A. Smith, 1895; *Drymaeus multispira* da Costa, 1904 = *Helix torallyi* d’Orbigny, 1835; *Bulimulus (Drymaeus) plicatoliratus* Da Costa, 1898 = *Bulimus convexus* Pfeiffer, 1855; *Bulimus sugillatus* Pfeiffer, 1857 = *Bulimus rivasii* d’Orbigny, 1837; *Bulimus meridionalis* Reeve 1848 [June] = *Bulimus voithianus* Pfeiffer, 1847.

New combinations are: *Bostryx montagnei* (d’Orbigny, 1837); *Bostryx obliquiportus* (da Costa, 1901); *Bulimulus heloicus* (d’Orbigny, 1835); *Drymaeus (Drymaeus) lusorius* (Pfeiffer, 1855); *Drymaeus (Drymaeus) trigonostomus* (Jonas, 1844); *Drymaeus (Drymaeus) wintlei* Finch, 1929; *Drymaeus (Mesembrinus) conicus* da Costa, 1907; *Kuschelenia (Kuschelenia) culminea culminea* (d’Orbigny, 1835); *Kuschelenia (Kuschelenia) culmineus edwardsi* (Morelet, 1863); *Kuschelenia (K.) gayi* (Pfeiffer, 1857); *Kuschelenia (Kuschelenia) tupacii* (d’Orbigny, 1835); *Kuschelenia (Vermiculatus) anthisanensis* (Pfeiffer, 1853); *Kuschelenia (Vermiculatus) aquilus* (Reeve, 1848); *Kuschelenia (Vermiculatus) bicolor* (Sowerby I, 1835); *Kuschelenia (Vermiculatus) caliginosus* (Reeve, 1849); *Kuschelenia (Vermiculatus) cotopaxiensis* (Pfeiffer, 1853); *Kuschelenia (Vermiculatus) filaris* (Pfeiffer, 1853); *Kuschelenia (Vermiculatus) ochracea* (Morelet, 1863); *Kuschelenia (Vermiculatus) petiti* (Pfeiffer, 1846); *Kuschelenia (Vermiculatus) purpuratus* (Reeve, 1849); *Kuschelenia (Vermiculatus) quechuarum* (Crawford, 1939); *Naesiotus cinereus* (Reeve, 1849); *Naesiotus dentritis* (Morelet, 1863); *Naesiotus fontainii* (d’Orbigny, 1838); *Naesiotus orbignyi* (Pfeiffer, 1846); *Protoglyptus pilosus* (Guppy, 1871); *Protoglyptus sanctaeluciae* (E.A. Smith, 1889).

Type material of the following taxa is figured herein for the first time: *Bulimus cinereus* Reeve, 1849; *Bulimus coriaceus* Pfeiffer, 1857; *Bulimulus laxostylus* Rolle, 1904; *Bulimus pliculatus* Pfeiffer, 1857; *Bulimus simpliculus* Pfeiffer, 1855.

## Introduction and methods

This is the third paper on the types of Orthalicoidea in the Natural History Museum, London. Earlier papers (Breure and Ablett 2011, 2012) have presented the context of the collection, the criteria used for the selection of lectotypes, some biohistorical notes, and a list of type specimens belonging to the Amphibulimidae, Bothriembryontidae, and Odontostomidae. The aim of this paper is to supplement the earlier papers with data of the type specimens classified with the Bulimulidae (sensu Breure and Romero 2012).

References are given to the original publication, plus those of following papers where type material has been mentioned or is (re-)figured. Dates of publication are in accordance with Coan et al. (2013a, 2013b) and Duncan (1937). Abbreviations used for depositories of material are: ANSP, Academy of Natural Sciences, Philadelphia, U.S.A.; MHNG, Muséum d’Histoire Naturelle, Genève, Switserland; MNHN, Muséum d’Histoire Naturelle, Paris, France; NHMUK, Natural History Museum, London, U.K.; RBINS, Royal Belgian Institute of Natural Sciences, Brussels, Belgium; RMNH, Naturalis Biodiversity Center, Leiden, the Netherlands (formerly the collections of Rijksmuseum van Natuurlijke Historie, Leiden, and Zoölogisch Museum, Amsterdam); SMF, Senckenberg Natur-Museum, Frankfurt am Main, Germany; ZMB, Zoologisches Museum, Humboldt Universität, Berlin, Germany. Other abbreviations used are: / end of line in cited text; coll., collection; D, diameter; H, shell height; leg., *legit*, collected; W, number of whorls. See Breure and Whisson 2012: fig. 1 for the way measurements on the shell have been taken. Label styles in the Cuming collection (”M.C. label style”) are explained in Breure and Ablett (2011: 7–8). Although most figures have been composed with the shells enlarged, its sizes retained relatively, one need to check the actual shell height in the legend.

## Systematics

### Systematic list of taxa arranged in generic order

This systematic list follows Breure (1979); the family classification is amended as proposed by Breure and Romero (2012). The latter authors conserved the well-known name Bulimulidae under Art. 35.5 ICZN, although Peltellinae Gray, 1855 falls within the same group. The generic classification has been adapted from Breure (1979) and unpublished data from the senior author. It may be noted that ongoing phylogenetic research may alter the classification. Within the family, genus and species level taxa are presented in alphabetical order.

#### Family Bulimulidae Tryon, 1867

***Auris* Spix, 1827**

*Auris swainsoni* Pfeiffer, 1845.

***Bostryx* Troschel, 1847 sensu Breure 1979 (see also Breure 2012b**)

*Bostryx acalles* Pfeiffer, 1853; *Bostryx affinis* Broderip in Broderip and Sowerby I 1832; *Bostryx agueroi* Weyrauch, 1960; *Bostryx aileenae* Breure, 1978; *Bostryx albicans* Broderip in Broderip and Sowerby I 1832; *Bostryx albicolor* Morelet, 1863; *Bostryx albus* Sowerby I, 1833; *Bostryx andoicus* Morelet, 1863; *Bostryx apodemeta* d’Orbigny, 1835; *Bostryx atacamensis* Pfeiffer, 1856; *Bostryx balsanus* Morelet, 1863; *Bostryx cactorum* d’Orbigny, 1835; *Bostryx ceratacme* Pfeiffer, 1855; *Bulimus cercicola* Morelet 1863; *Bostryx compactus* Fulton, 1902; *Bostryx conspersus* Sowerby I, 1833; *Bostryx costatus* Weyrauch, 1960; *Bostryx costifer* Weyrauch, 1960; *Bostryx delumbis* Reeve, 1849; *Bostryx denickei* J.E. Gray, 1852; *Bostryx dentritis* Morelet, 1863; *Bostryx depstus* Reeve, 1849; *Bostryx derelictus* Broderip in Broderip and Sowerby I 1832; *Bostryx devians* Dohrn, 1863; *Bostryx emaciatus* Morelet, 1863; *Bostryx erosus* Broderip in Broderip and Sowerby I 1832; *Bostryx ferrugineus* Reeve, 1849; *Bostryx glomeratus* Weyrauch, 1960; *Bostryx guttatus* Broderip in Broderip and Sowerby I 1832; *Bostryx hamiltoni* Reeve, 1849; *Bostryx holostoma* Pfeiffer, 1846; *Bostryx huascensis* Reeve, 1848; *Bostryx infundibulum* Pfeiffer, 1853; *Bostryx kathiae* Breure, 1978; *Bostryx lactifluus* Pfeiffer, 1857; *Bostryx lesueureanus* Morelet, 1860; *Bostryx lichnorum* d’Orbigny, 1835; *Bostryx limensis* Reeve, 1849; *Bostryx limonoica* d’Orbigny, 1835; *Bostryx longinquus* Morelet, 1863; *Bostryx luridus* Pfeiffer, 1863; *Bostryx mejillonensis* Pfeiffer, 1857; *Bostryx metagyra* Pilsbry and Olsson 1949; *Bostryx minor* Weyrauch, 1960; *Bostryx modestus* Broderip in Broderip and Sowerby I 1832; *Bostryx moniezi* Dautzenberg, 1896; *Bostryx montagnei* d’Orbigny, 1837; *Bostryx mordani* Breure, 1978; *Bostryx multispira* da Costa, 1904; *Bostryx nanus* Reeve, 1849; *Bostryx nigrolimbatus* Pfeiffer, 1853; *Bostryx obliquistriatus* da Costa, 1901; *Bostryx orophilus* Morelet, 1860; *Bostryx papillatus* Morelet, 1860; *Bostryx paposensis* Pfeiffer, 1856; *Bostryx paucicostatus* Breure, 1978; *Bostryx philippii* Pfeiffer, 1842; *Bostryx pictus* Pfeiffer, 1855; *Bostryx pruinosus* Sowerby I, 1833; *Bostryx pupiformis* Broderip in Broderip and Sowerby I 1832; *Bostryx pustulosus* Broderip in Broderip and Sowerby I 1832; *Bostryx radiatus* Morelet, 1863; *Bostryx reconditus* Reeve, 1849; *Bostryx rehderi* Weyrauch, 1960; *Bostryx rhodolarynx* Reeve, 1849; *Bostryx rodriguezae* Weyrauch, 1967; *Bostryx rusticellus* Morelet, 1860; *Bostryx scabiosus* Sowerby I, 1833; *Bostryx scalaricosta* Morelet, 1860; *Bostryx scalariformis* Broderip in Broderip and Sowerby I 1832; *Bostryx serotinus* Morelet, 1860; *Bostryx simpliculus* Pfeiffer, 1855; *Bostryx spiculatus* Morelet, 1860; *Bostryx stenacme* Pfeiffer, 1857; *Bostryx terebralis* Pfeiffer, 1842; *Bostryx torallyi* d’Orbigny, 1835; *Bostryx tricinctus* Reeve, 1848; *Bostryx tumidulus* Pfeiffer, 1842; *Bostryx turritus* Broderip in Broderip and Sowerby I 1832; *Bostryx veruculum* Morelet, 1860; *Bostryx vilchezi* Weyrauch, 1960; *Bostryx virgultorum* Morelet, 1863; *Bostryx voithianus* Pfeiffer, 1847; *Bostryx woodwardi* Pfeiffer, 1857.

***Bulimulus* Leach, 1814**

*Bulimulus barbadensis* Pfeiffer, 1853; *Bulimulus cacticolus* Reeve, 1849; *Bulimulus dysoni* Pfeiffer, 1846; *Bulimulus effeminatus* Reeve, 1848; *Bulimulus erectus* Reeve 1849; *Bulimulus haplochrous* Pfeiffer, 1855; *Bulimulus heloica* d’Orbigny, 1835; *Bulimulus ignavus* Reeve, 1849; *Bulimulus inutilis* Reeve, 1850; *Bulimulus istapensis* Crosse & Fischer, 1873; *Bulimulus juvenilis* Pfeiffer, 1855; *Bulimulus mollicellus* Reeve, 1849; *Bulimulus monachus* Pfeiffer, 1857; *Bulimulus montevidensis* Pfeiffer, 1846; *Bulimulus nubeculatus* Pfeiffer, 1853; *Bulimulus pervius* Pfeiffer, 1853; *Bulimulus pessulatus* Reeve, 1848; *Bulimulus petenensis* Morelet, 1851; *Bulimulus pliculatus* Pfeiffer, 1857; *Bulimulus rubrifasciatus* Reeve, 1848; *Bulimulus sporadica* d’Orbigny, 1835; *Bulimulus transparens* Reeve, 1849; *Bulimulus turritella* d’Orbigny, 1835; *Bulimulus vesicalis* Pfeiffer, 1853.

***Drymaeus (Drymaeus)* Albers, 1850**

*Drymaeus (Drymaeus) abruptus* Rolle, 1904; *Drymaeus (Drymaeus) abscissus* Pfeiffer, 1855; *Drymaeus (Drymaeus) abyssorum* d’Orbigny, 1835; *Drymaeus (Drymaeus) aequatorianus* E.A. Smith, 1877; *Drymaeus (Drymaeus) acervatus* Pfeiffer, 1857; *Drymaeus (Drymaeus) acuminatus* da Costa, 1906; *Drymaeus (Drymaeus) alabastrinus* da Costa, 1906; *Drymaeus (Drymaeus) albolabiatus* E.A. Smith, 1877; *Drymaeus (Drymaeus) ambustus* Reeve, 1849; *Drymaeus (Drymaeus) angustus* da Costa, 1906; *Drymaeus (Drymaeus) antioquiensis* Pfeiffer, 1855; *Drymaeus (Drymaeus) arcuatostriatus* Pfeiffer, 1855; *Drymaeus (Drymaeus) auris* Pfeiffer, 1866; *Drymaeus (Drymaeus) baranguillanus* Pfeiffer, 1853; *Drymaeus (Drymaeus) bartletti* H. Adams, 1867; *Drymaeus (Drymaeus) bellus* da Costa, 1906; *Drymaeus (Drymaeus) bogotensis* Pfeiffer, 1855; *Drymaeus (Drymaeus) bolivarii* d’Orbigny, 1835; *Drymaeus (Drymaeus) bolivianus* Pfeiffer, 1846; *Drymaeus (Drymaeus) boucardi* da Costa, 1907; *Drymaeus (Drymaeus) bourcieri* Pfeiffer, 1853; *Drymaeus (Drymaeus) brachysoma* d’Orbigny, 1835; *Drymaeus (Drymaeus) buckleyi* Sowerby III, 1895; *Drymaeus (Drymaeus) canaliculatus* Pfeiffer, 1845; *Drymaeus (Drymaeus) castaneostrigatus* da Costa, 1906; *Drymaeus (Drymaeus) caucaensis* da Costa, 1898; *Drymaeus (Drymaeus) chamaeleon* Pfeiffer, 1855; *Drymaeus (Drymaeus) chimborasensis* Reeve, 1848; *Drymaeus (Drymaeus) chiriquensis* da Costa, 1901; *Drymaeus (Drymaeus) clathratus* Pfeiffer, 1858; *Drymaeus (Drymaeus) coarctatus* Pfeiffer, 1845; *Drymaeus (Drymaeus) confluens* Pfeiffer, 1855; *Drymaeus (Drymaeus) convexus* Pfeiffer, 1855; *Drymaeus (Drymaeus) cuticula* Pfeiffer, 1855; *Drymaeus (Drymaeus) cuzcoensis* Reeve, 1849; *Drymaeus (Drymaeus) cylindricus* da Costa, 1901; *Drymaeus (Drymaeus) dacostae* Sowerby III, 1892; *Drymaeus (Drymaeus) dombeyanus* Pfeiffer, 1846; *Drymaeus (Drymaeus) dunkeri* Pfeiffer in Philippi 1846; *Drymaeus (Drymaeus) elsteri* da Costa, 1901; *Drymaeus (Drymaeus) exoticus* da Costa, 1901; *Drymaeus (Drymaeus) expatriatus* Preston, 1909; *Drymaeus (Drymaeus) fabrefactus* Reeve, 1848; *Drymaeus (Drymaeus) fallax* Pfeiffer, 1853; *Drymaeus (Drymaeus) farrisi* Pfeiffer, 1858; *Drymaeus (Drymaeus) felix* Pfeiffer, 1862; *Drymaeus (Drymaeus) fenestratus* Pfeiffer, 1846; *Drymaeus (Drymaeus) flexilabris* Pfeiffer, 1853; *Drymaeus (Drymaeus) flexuosus* Pfeiffer, 1853; *Drymaeus (Drymaeus) fucatus* Reeve, 1849; *Drymaeus (Drymaeus) fusoides* d’Orbigny, 1835; *Drymaeus (Drymaeus) gealei* H. Adams, 1867; *Drymaeus (Drymaeus) geometricus* Pfeiffer, 1846; *Drymaeus (Drymaeus) hidalgoi* da Costa, 1898; *Drymaeus (Drymaeus) humboldtii* Reeve, 1849; *Drymaeus (Drymaeus) hygrohylea* d’Orbigny, 1835; *Drymaeus (Drymaeus) inclinatus* Pfeiffer, 1862; *Drymaeus (Drymaeus) incognitus* da Costa, 1907; *Drymaeus (Drymaeus) knorri* Pfeiffer in Philippi 1846; *Drymaeus (Drymaeus) lattrei* Pfeiffer in Philippi 1846; *Drymaeus (Drymaeus) linostoma* d’Orbigny, 1835; *Drymaeus (Drymaeus) lophoica* d’Orbigny, 1835; *Drymaeus (Drymaeus) lucidus* da Costa, 1898; *Drymaeus (Drymaeus) marmarina* d’Orbigny, 1835; *Drymaeus (Drymaeus) murrinus* Reeve, 1848; *Drymaeus (Drymaeus) musivus* Pfeiffer, 1855; *Drymaeus (Drymaeus) napo* Angas, 1878; *Drymaeus (Drymaeus) notabilis* da Costa, 1906; *Drymaeus (Drymaeus) notatus* da Costa, 1906; *Drymaeus (Drymaeus) nystianus* Pfeiffer, 1853; *Drymaeus (Drymaeus) ochrocheilus* E.A. Smith, 1877; *Drymaeus (Drymaeus) orthostoma* E.A. Smith, 1877; *Drymaeus (Drymaeus) phryne* Pfeiffer, 1863; *Drymaeus (Drymaeus) plicatoliratus* da Costa, 1898; *Drymaeus (Drymaeus) poecila* d’Orbigny, 1835; *Drymaeus (Drymaeus) praetextus* Reeve, 1849; *Drymaeus (Drymaeus) protractus* Pfeiffer, 1855; *Drymaeus (Drymaeus) pseudofusoides* da Costa, 1906; *Drymaeus (Drymaeus) pulcherrimus* H. Adams, 1867; *Drymaeus (Drymaeus) punctatus* da Costa, 1907; *Drymaeus (Drymaeus) quadrifasciatus* Angas, 1878; *Drymaeus (Drymaeus) recedens* Pfeiffer, 1864; *Drymaeus (Drymaeus) regularis* Fulton, 1905; *Drymaeus (Drymaeus) rosenbergi* da Costa, 1900; *Drymaeus (Drymaeus) saccatus* Pfeiffer, 1855; *Drymaeus (Drymaeus) scitulus* Reeve, 1849; *Drymaeus (Drymaeus) scitus* H. Adams, 1867; *Drymaeus (Drymaeus) selli* Preston, 1909; *Drymaeus (Drymaeus) serratus* Pfeiffer, 1855; *Drymaeus (Drymaeus) smithii* da Costa, 1898; *Drymaeus (Drymaeus) solidus* Preston, 1907; *Drymaeus (Drymaeus) spadiceus* da Costa, 1906; *Drymaeus (Drymaeus) spectatus* Reeve, 1849; *Drymaeus (Drymaeus) strigatus* Sowerby I, 1833; *Drymaeus (Drymaeus) subhybridus* da Costa, 1906; *Drymaeus (Drymaeus) subinterruptus* Pfeiffer, 1853; *Drymaeus (Drymaeus) subventricosus* da Costa, 1901; *Drymaeus (Drymaeus) sykesi* da Costa, 1906; *Drymaeus (Drymaeus) tigrinus* da Costa, 1898; *Drymaeus (Drymaeus) vespertinus* Pfeiffer, 1858; *Drymaeus (Drymaeus) volsus* Fulton, 1907; *Drymaeus (Drymaeus) xanthostoma* d’Orbigny, 1835; *Drymaeus (Drymaeus) yungasensis* d’Orbigny, 1837; *Drymaeus (Drymaeus) zhorquinensis* Angas, 1879; *Drymaeus (Drymaeus) ziczac* da Costa, 1898; *Drymaeus (Drymaeus) zoographica* d’Orbigny, 1835.

***Drymaeus (Mesembrinus)* Albers, 1850**

*Drymaeus (Mesembrinus) aestivus* Pfeiffer, 1857; *Drymaeus (Mesembrinus) amandus* Pfeiffer, 1855; *Drymaeus (Mesembrinus) andicola* Pfeiffer, 1847; *Drymaeus (Mesembrinus) apicepunctata* Preston, 1914; *Drymaeus (Mesembrinus) apiculata* J.E. Gray, 1834; *Drymaeus (Mesembrinus) attenuatus* Pfeiffer, 1853; *Drymaeus (Mesembrinus) aureolus* Guppy, 1866; *Drymaeus (Mesembrinus) aurifluus* Pfeiffer, 1857; *Drymaeus (Mesembrinus) broadwayi* E.A. Smith, 1896; *Drymaeus (Mesembrinus) bugabensis* Martens, 1893; *Drymaeus (Mesembrinus) californicus* Reeve, 1848; *Drymaeus (Mesembrinus) cancellata* da Costa, 1906; *Drymaeus (Mesembrinus) castus* Pfeiffer, 1847; *Drymaeus (Mesembrinus) championi* Martens, 1893; *Drymaeus (Mesembrinus) citronellus* Angas, 1879; *Drymaeus (Mesembrinus) columbiensis* Pfeiffer, 1856; *Drymaeus (Mesembrinus) conicus* da Costa, 1907; *Drymaeus (Mesembrinus) demotus* Reeve, 1850; *Drymaeus (Mesembrinus) depictus* Reeve, 1849; *Drymaeus (Mesembrinus) deshayesi* Pfeiffer, 1845; *Drymaeus (Mesembrinus) discrepans* Sowerby I, 1833; *Drymaeus (Mesembrinus) dubius* Pfeiffer, 1853; *Drymaeus (Mesembrinus) dutaillyi* Pfeiffer, 1857; *Drymaeus (Mesembrinus) electrum* Reeve, 1848; *Drymaeus (Mesembrinus) erubescens* Pfeiffer, 1847; *Drymaeus (Mesembrinus) feriatus* Reeve, 1850; *Drymaeus (Mesembrinus) fidustus* Reeve, 1849; *Drymaeus (Mesembrinus) flavidulus* E.A. Smith, 1877; *Drymaeus (Mesembrinus) floridanus* Pfeiffer, 1857; *Drymaeus (Mesembrinus) fuscobasis* E.A. Smith, 1877; *Drymaeus (Mesembrinus) gabbi* Angas, 1879; *Drymaeus (Mesembrinus) gruneri* Pfeiffer, 1846; *Drymaeus (Mesembrinus) hachensis* Reeve, 1850; *Drymaeus (Mesembrinus) hepatostomus* Pfeiffer, 1861; *Drymaeus (Mesembrinus) hoffmanni* Martens, 1893; *Drymaeus (Mesembrinus) hondurasanus* Pfeiffer, 1846; *Drymaeus (Mesembrinus) hypozonus* Martens, 1893; *Drymaeus (Mesembrinus) immaculatus* C.B. Adams in Reeve 1850; *Drymaeus (Mesembrinus) incarnatus* Pfeiffer, 1855; *Drymaeus (Mesembrinus) inglorius* Reeve, 1848; *Drymaeus (Mesembrinus) interruptus* Preston, 1909; *Drymaeus (Mesembrinus) inusitatus* Fulton, 1900; *Drymaeus (Mesembrinus) jonasi* Pferiffer in Philippi 1846; *Drymaeus (Mesembrinus) keppelli* Pfeiffer, 1853; *Drymaeus (Mesembrinus) koppeli* Sowerby III, 1892; *Drymaeus (Mesembrinus) laetus* Reeve, 1849; *Drymaeus (Mesembrinus) lascellianus* E.A. Smith, 1895; *Drymaeus (Mesembrinus) lirinus* Morelet, 1851; *Drymaeus (Mesembrinus) lividus* Reeve, 1850; *Drymaeus (Mesembrinus) loxensis* Pfeiffer, 1846; *Drymaeus (Mesembrinus) lucidus* Reeve, 1848; *Drymaeus (Mesembrinus) lusorius* Pfeiffer, 1855; *Drymaeus (Mesembrinus) manupictus* Reeve, 1848; *Drymaeus (Mesembrinus) meridanus* Pfeiffer, 1846; *Drymaeus (Mesembrinus) monilifer* Reeve, 1848; *Drymaeus (Mesembrinus) moricandi* Pfeiffer, 1847; *Drymaeus (Mesembrinus) mossi* E.A. Smith, 1896; *Drymaeus (Mesembrinus) moussoni* Pfeiffer, 1853; *Drymaeus (Mesembrinus) muliebris* Reeve, 1849; *Drymaeus (Mesembrinus) nigrofasciatus* Pfeiffer in Philippi 1846; *Drymaeus (Mesembrinus) nitelinus* Reeve, 1849; *Drymaeus (Mesembrinus) nitidus* Broderip in Broderip and Sowerby I 1832; *Drymaeus (Mesembrinus) pallens* Reeve, 1849; *Drymaeus (Mesembrinus) panamensis* Broderip in Broderip and Sowerby I 1832; *Drymaeus (Mesembrinus) pervariabilis* Pfeiffer, 1853; *Drymaeus (Mesembrinus) prestoni* da Costa, 1906; *Drymaeus (Mesembrinus) primula* Reeve, 1848; *Drymaeus (Mesembrinus) puellaris* Reeve, 1850; *Drymaeus (Mesembrinus) rawsonis* H. Adams, 1873; *Drymaeus (Mesembrinus) rectilinearis* Pfeiffer, 1855; *Drymaeus (Mesembrinus) roseatus* Reeve, 1848; *Drymaeus (Mesembrinus) signifer* Pfeiffer, 1855; *Drymaeus (Mesembrinus) sisalensis* Morelet, 1849; *Drymaeus (Mesembrinus) sowerbyi* Pfeiffer, 1847; *Drymaeus (Mesembrinus) studeri* Pfeiffer, 1847; *Drymaeus (Mesembrinus) subpellucidus* E.A. Smith, 1877; *Drymaeus (Mesembrinus) sulcosus* Pfeiffer, 1841; *Drymaeus (Mesembrinus) sulphureus* Pfeiffer, 1857; *Drymaeus (Mesembrinus) tenuilabris* Pfeiffer, 1866; *Drymaeus (Mesembrinus) translucens* Broderip in Broderip and Sowerby I 1832; *Drymaeus (Mesembrinus) trimarianus* Martens, 1893; *Drymaeus (Mesembrinus) trinitarius* E.A. Smith, 1986; *Drymaeus (Mesembrinus) tristis* Pfeiffer, 1855; *Drymaeus (Mesembrinus) tropicalis* Morelet, 1849; *Drymaeus (Mesembrinus) umbraticus* Reeve, 1850; *Drymaeus (Mesembrinus) varicosus* Pfeiffer, 1853; *Drymaeus (Mesembrinus) vincentinus* Pfeiffer, 1846; *Drymaeus (Mesembrinus) virginalis* Pfeiffer, 1856; *Drymaeus (Mesembrinus) wintlei* Finch, 1929.

***Kuschelenia (Kuschelenia)* Hylton Scott, 1951**

*Kuschelenia (Kuschelenia) confusus* Reeve, 1848; *Kuschelenia (Kuschelenia) culminea* d’Orbigny, 1835; *Kuschelenia (Kuschelenia) edwardsi* Morelt, 1863; *Kuschelenia (Kuschelenia) gayi* Pfeiffer, 1857; *Kuschelenia (Kuschelenia) jussieui* Pfeiffer, 1846; *Kuschelenia (Kuschelenia) lithoica* d’Orbigny, 1835; *Kuschelenia (Kuschelenia) thamnoica* d’Orbigny, 1835; *Kuschelenia (Kuschelenia) tupacii* d’Orbigny, 1835.

***Kuschelenia (Vermiculatus)* Breure, 1978**

*Kuschelenia (Vermiculatus) anthisanensis* Pfeiffer, 1853; *Kuschelenia (Vermiculatus) aquilus* Reeve, 1848; *Kuschelenia (Vermiculatus) badius* Sowerby I, 1835; *Kuschelenia (Vermiculatus) bicolor* Sowerby I, 1835; *Kuschelenia (Vermiculatus) caliginosus* Reeve, 1849; *Kuschelenia (Vermiculatus) coagulatus* Reeve, 1849; *Kuschelenia (Vermiculatus) cotopaxiensis* Pfeiffer, 1853; *Kuschelenia (Vermiculatus) filaris* Pfeiffer, 1853; *Kuschelenia (Vermiculatus) nucinus* Reeve, 1850; *Kuschelenia (Vermiculatus) ochraceus* Morelet, 1863; *Kuschelenia (Vermiculatus) peaki* Breure, 1978; *Kuschelenia (Vermiculatus) petiti* Pfeiffer, 1846; *Kuschelenia (Vermiculatus) polymorpha* d’Orbigny, 1835; *Kuschelenia (Vermiculatus) purpuratus* Reeve, 1849; *Kuschelenia (Vermiculatus) quechuarum* Crawford, 1939; *Kuschelenia (Vermiculatus) subfasciatus* Pfeiffer, 1853.

***Naesiotus* Albers, 1850 sensu Breure 1979**

*Naesiotus achatellinus* Forbes, 1850; *Naesiotus albemarlensis* Dall, 1917; *Naesiotus apertus* Pfeiffer, 1855; *Naesiotus catlowiae* Pfeiffer, 1853; *Naesiotus chamayensis* Weyrauch, 1967; *Naesiotus chemnitzioides* Forbes, 1850; *Naesiotus cinereus* Reeve, 1849; *Naesiotus crepundia* d’Orbigny, 1835; *Naesiotus curtus* Reibisch, 1892; *Naesiotus darwini* Pfeiffer, 1846; *Naesiotus exornatus* Reeve, 1849; *Naesiotus fernandezae* Weyrauch, 1958; *Naesiotus fontainii* d’Orbigny, 1838; *Naesiotus fourmiersi* d’Orbigny, 1837; *Naesiotus galapaganus* Pfeiffer, 1855; *Naesiotus irregularis* Pfeiffer, 1848; *Naesiotus jacobi* Sowerby I, 1833; *Naesiotus lycodus* Dall, 1917; *Naesiotus montivaga* d’Orbigny, 1835; *Naesiotus munsterii* d’Orbigny, 1837; *Naesiotus nucula* Pfeiffer, 1853; *Naesiotus nux* Broderip, 1832; *Naesiotus orbignyi* Pfeiffer, 1846; *Naesiotus pallidus* Reibisch, 1892; *Naesiotus paziana* d’Orbigny, 1835; *Naesiotus perspectivus* Pfeiffer, 1846; *Naesiotus phlegonis* Dall & Ochsner, 1928; *Naesiotus quitensis* Pfeiffer, 1848; *Naesiotus rimatus* Pfeiffer, 1847; *Naesiotus rivasii* d’Orbigny, 1837; *Naesiotus rocayana* d’Orbigny, 1835; *Naesiotus rugiferus* Sowerby I, 1833; *Naesiotus sugillatus* Pfeiffer, 1857; *Naesiotus terebra* Reibisch, 1892; *Naesiotus trichoda* d’Orbigny, 1835; *Naesiotus unifasciatus* Sowerby I, 1833; *Naesiotus ustulatus* Sowerby I, 1833; *Naesiotus ventrosus* Reibisch, 1892; *Naesiotus verrucosus* Pfeiffer, 1855; *Naesiotus wolfi* Reibisch, 1892.

***Neopetraeus* Martens, 1885**

*Neopetraeus altoperuvianus* Reeve, 1849; *Neopetraeus atahualpa* Dohrn, 1863; *Neopetraeus binneyanus* Pfeiffer, 1857; *Neopetraeus cora* d’Orbigny, 1835; *Neopetraeus decussatus* Reeve, 1849; *Neopetraeus excoriatus* Pfeiffer, 1855; *Neopetraeus lobbii* Reeve, 1849; *Neopetraeus myristicus* Reeve, 1849; *Neopetraeus patasensis* Pfeiffer, 1858; *Neopetraeus platystomus* Pfeiffer, 1858; *Neopetraeus ptychostylus* Pfeiffer, 1858.

***Newboldius* Pilsbry, 1932**

*Newboldius crichtoni* Broderip, 1836; *Newboldius illustris* Rolle, 1905.

***Protoglyptus* Pilsbry, 1897**

*Protoglyptus martinicensis* Pfeiffer, 1846; *Protoglyptus pilosus* Guppy, 1871; *Protoglyptus sanctaeluciae* E.A. Smith, 1889.

***Rabdotus* Albers, 1850**

*Rabdotus juarezi* Pfeiffer, 1866; *Rabdotus liquabilis* Reeve, 1848; *Rabdotus ragsdalei* Pilsbry, 1890; *Rabdotus schiedeanus* Pfeiffer, 1841.

***Scutalus* Albers, 1850**

*Scutalus baroni* (*Helix*) Fulton, 1896; *Scutalus baroni* (*Bulimulus*) Fulton, 1897; *Scutalus chiletensis* Weyrauch, 1967; *Scutalus cretaceus* Pfeiffer, 1855; *Scutalus grandiventris* Weyrauch, 1960; *Scutalus latecolumellaris* Preston, 1909; *Scutalus proteus* Broderip in Broderip and Sowerby I 1832; *Scutalus versicolor* Broderip in Broderip and Sowerby I 1832.

***Stenostylus* Pilsbry, 1898**

*Stenostylus meleagris* Pfeiffer, 1853; *Stenostylus nigrolimbatus* Pfeiffer, 1853.

**Nomina inquirenda**

*clarus* Pfeiffer, 1857; *dukinfieldi* Melvill, 1900; *gelidus* Reeve, 1849; *nivalis* d’Orbigny, 1835; *pallens* Reeve, 1849; *sowerbyi* Pfeiffer, 1847.

##### Alphabetic list of taxa by species name

###### Bulimulus (Drymaeus) abruptus

Rolle, 1904

http://species-id.net/wiki/Bulimulus_abruptus

Figs 26J–L , L1i

Bulimulus (Drymaeus) abruptus Rolle 1904: 35.

####### Type locality.

”Huancabamba in Peru”; see remarks.

####### Label.

”Huancabamba,/ Peru 1904”; in Rolle’s handwriting. Another label in pencil indicating that it came from the Fulton collection.

####### Dimensions.

”Alt. 44, diam. max. 24 (..) mm”; figured specimen herein H 42.5, D 21.5 W 6.3.

####### Type material.

NHMUK 1947.2.10.1, syntype (ex Rolle).

####### Remarks.

Rolle did not state on how many specimens his description was based upon. However, the shell height is close to the original measurements and together with the indication ”type” on the label in Rolle’s handwriting, there is no doubt about the status of the specimen. The locality is ambiguous, as there are several places called ”Huancabamba” in Peru. There were, however, several other species described from the same locality, amoung them *Columbinia huancabambensis*, which is regarded by Loosjes and Loosjes-van Bemmel (1984: 33), as occurring in northern Peru. Another species described in the same paper, *Systrophia moellendorffii*, was said by Haas (1955: 367) to have been rediscovered in the Chanchamayo valley [Dept. Pasco]. Given the fact that a third species described by Rolle—*Newboldius illustris*—is known to occur in the same region, makes it likely that Huancabamba in Dept. Pasco is the locality where Rolle’s taxon occurs. This species was hitherto considered as published in 1905 (Richardson 1995: 95); however, on the copy in the Smithsonian Institution Libraries the title page of the issue in which Rolle’s paper appeared is marked ”Rec’d Mar. 2/04” (see http://www.biodiversitylibrary.org/item/53294#page/211/mode/1up); p. 48 of this issue mentions ”Ausgegeben 22. Februar”.

####### Current systematic position.

Bulimulidae, *Drymaeus (Drymaeus) abruptus* (Rolle, 1904).

###### Bulimus abscissus

Pfeiffer, 1855

http://species-id.net/wiki/Bulimus_abscissus

Figs 26A–C , L1ii

Bulimus abscissus Pfeiffer 1855f: 116; Pfeiffer 1859b: 376; Breure 1979: 105 (lectotype designation).

####### Type locality.

”Province of Quito, Ecuador”.

####### Label.

”Ecuador”, taxon label in Pfeiffer’s handwriting. M.C. label style IV.

####### Dimensions.

”Long. 28, diam. 13 mill.”; lectotype H 27.8, D 14.3, W 5.5.

####### Type material.

NHMUK 1975497, lectotype; 1975498 five paralectotypes (Cuming coll.).

####### Remarks.

Pfeiffer did not state on how many specimens his description was based; no variety has been mentioned by him. Two sets of labels are with this lot, which is originating from the Cuming collection; one reading ”Ecuador” in the handwriting of one of Cuming’s secretaries. On both labels the taxon name is in Pfeiffer’s handwriting. One of the lots has been labelled ”*abscissus* var.” in a later handwriting.

####### Current systematic position.

Bulimulidae, *Drymaeus (Drymaeus) abcissus* (Pfeiffer, 1855).

###### Helix abyssorum

d’Orbigny, 1835

http://species-id.net/wiki/Helix_abyssorum

Figs 27A–C , L1iii

Helix abyssorum d’Orbigny 1835: 17.
Bulimus abyssorum ; d’Orbigny 1837 [1834–1847]: 308, pl. 39 figs 7–8 [7 Aug. 1837; text 6 May 1838]; Gray 1854: 21.
Drymaeus abyssorum ; Pilsbry 1898 [1897–1898]: 192, pl. 37 figs 3–4; Breure 1975b: 1149, pl. 7 fig. 2 (lectotype designation); Cuezzo et al. 2013: 153.
Drymaeus (Drymaeus) hygrohylaeus ; Miquel 1989b: 77, fig. 1.

####### Type locality.

”provincia Lagunacensi (republica Boliviana)”; see Breure 1973: 113.

####### Label.

”pampa ruis, P^ce^ Laguna, Bolivia”, in d’Orbigny’s handwriting.

####### Dimensions.

”Longit. 52 mil., latit. 25 millim.”; figured specimen herein H 43.7 D 23.3, W 6.2.

####### Type material.

NHMUK 1854.12.4.125, five paralectotypes (d’Orbigny coll.).

####### Remarks.

d’Orbigny did not state on how many specimens his description was based. The types series appears to have been split between the MNHN and NHMUK collections. None of the specimens in London match the original figures, while one of the specimens in Paris is close to the published dimensions. The current systematic position follows the synonymisation of this taxon with *Helix hygrohylea* d’Orbigny, 1835, by Miquel (1989b: 77); this author, however, overlooked that *Helix abyssorum* has page priority. Cuezzo et al. (2013) disageed with this synonymisation.

####### Current systematic position.

Bulimulidae, *Drymaeus (Drymaeus) abyssorum* (d’Orbigny, 1835).

###### Bulimus acalles

Pfeiffer, 1853

http://species-id.net/wiki/Bulimus_acalles

Figs 6A , L1iv

Bulimus acalles Pfeiffer 1853b [7 Dec.]: 258; Pfeiffer 1853d [31 Dec.]: 410; Pfeiffer 1853 [31 Dec.] in Küster and Pfeiffer 1840–1865: 84, pl. 30 figs 27–28.
Bulimulus (Lissoacme) acalles ; Pilsbry 1896 [1895–1896]: 160, pl. 50 figs 53–54.

####### Type locality.

”in Andibus Peruvianis”.

####### Label.

”Peru”. M.C. label style I.

####### Dimensions.

”Long. 10, diam. 6 mill.”; figured specimen herein H 13.2, D 7.7, W 5.3.

####### Type material.

NHMUK 20100651, two possible syntypes (Cuming coll.).

####### Remarks.

The specimens found are slightly larger than the original measurements; the largest has shell height 13.2 mm. The material—not accompanied by a taxon label in Pfeiffer’s handwriting—is from the Cuming collection, on which Pfeiffer based the description for this taxon; he did not state on how many specimens his description was based. The specimens are treated as possible syntypes.

####### Current systematic position.

Bulimulidae, *Bostryx acalles* (Pfeiffer, 1853).

###### Bulimus acervatus

Pfeiffer, 1857

http://species-id.net/wiki/Bulimus_acervatus

Figs 50A–C , L1v

Bulimus acervatus Pfeiffer 1857a: 157.
Drymaeus (Drymaeus) acervatus ; Breure and Eskens 1981: 4, pl. 8 fig. 6 (lectotype designation).

####### Type locality.

”Brasilia. (*Strain*)”.

####### Label.

”Brazils. M^r^ Strain”; taxon name in Pfeiffer’s handwriting. M.C. label style IV.

####### Dimensions.

”Long. 41, diam. 21 mill.”; lectotype H 41.5, D 24.7, W 6.5.

####### Type material.

NHMUK 1975461, lectotype (Cuming coll.).

####### Remarks.

The current systematic position follows Richardson (1995: 96).

####### Current systematic position.

Bulimulidae, *Drymaeus (Drymaeus) acervatus* (Pfeiffer, 1857).

###### Bulimus achatellinus

Forbes, 1850

http://species-id.net/wiki/Bulimus_achatellinus

Figs 14J , L2i

Bulimus achatellinus Forbes 1850: 56, pl. 9 figs 5a–b; Pfeiffer 1853 in Küster and Pfeiffer 1840–1865: 93, pl. 31 figs 21-23.
Bulimulus achatellinus ; Pilsbry 1897 [1897–1898]: 99, pl. 16 figs 28–29.
Naesiotus achatellinus ; Breure and Borrero 2008: 10.

####### Type locality.

[Ecuador, Galápagos, Isla San Cristóbal] ”Chatham Island, Gelepagos”.

####### Label.

”Chatham Is Galapagos”, added on a different label with E.A. Smith’s handwriting. The taxon name is on a second label ”Bulimus achatenellinus [sic] Forbes, figured specimen, Proc Zool Soc (...) fig. 5”. M.C. label style III.

####### Dimensions.

”Long. 19, diam. 10 mill.”; holotype H 19, D 9.52, W 8.7.

####### Type material.

NHMUK 1855.4.5.25, holotype (Cuming coll.).

####### Remarks.

Forbes writes ”This shell (...)”, therefore the single specimen found, together with the indication on the label that it was figured, makes it justifiable to consider it a holotype.

####### Current systematic position.

Bulimulidae, *Naesiotus achatellinus* (Forbes, 1850).

###### Drymaeus acuminatus

da Costa, 1906

http://species-id.net/wiki/Drymaeus_acuminatus

Figs 45A–C , L2ii

Drymaeus acuminatus da Costa 1906a: 8, pl. 1 fig. 4; Simone 2006: 135, fig. 440.
Drymaeus (Drymaeus) acuminatus ; Breure 1979: 106.

####### Type locality.

”Matto Grosso, Brazil”.

####### Label.

”Matto Grosso / Brazil”; in da Costa’s handwriting.

####### Dimensions.

”Long. 33, diam. 14 mm.”; holotype H 33.4, D 14.1, W 7.4.

####### Type material.

NHMUK 1907.11.21.10, holotype (da Costa coll.).

####### Remarks.

da Costa wrote ”A rather peculiar shell, unlike any known to the writer, who obtained it at the dispersal of the collection of the late Mr. Miers”; given the singular form, it may be assumed that da Costa only had one specimen at hand.

####### Current systematic position.

Bulimulidae, *Drymaeus (Drymaeus) acuminatus* da Costa, 1906.

###### Bulimus (Drymaeus) aequatorianus

E.A. Smith, 1877

http://species-id.net/wiki/Bulimus_aequatorianus

Figs 28E–G , L2iii

Bulimus (Drymaeus) aequatorianus E.A. Smith 1877: 363, pl. 30 fig. 7.
Drymaeus (Drymaeus) aequatorianus ; Breure 1979: 106 (lectotype designation); Breure and Eskens 1981: 4; Breure and Borrero 2008: 19.

####### Type locality.

”Ecuador”.

####### Label.

”Ecuador”. M.C. label style III.

####### Dimensions.

”Long. 26 1/2 mill., diam. 11”; lectotype H 26.6, D 13.4, W 6.1.

####### Type material.

NHMUK 1975137, lectotype. NHMUK 1975138, three paralectotypes (Cuming coll.).

####### Current systematic position.

Bulimulidae, *Drymaeus (Drymaeus) aequatorianus* (E.A. Smith, 1877).

###### Bulimus aequatorius

Pfeiffer, 1853

http://species-id.net/wiki/Bulimus_aequatorius

Figs 69A–B , L2v–vi

Bulimus aequatorius Pfeiffer 1853d: 420; Pfeiffer 1854 in Küster and Pfeiffer 1840–1865: 101, pl. 13 figs 1–4; Pfeiffer 1854c: 155; Breure 1979: 85 (lectotype designation).
Bulimulus aequatorius ; Pilsbry 1897 [1897–1898]: 30, pl. 7 figs 1–5.
Scutalus (Vermiculatus) aequatorius ; Breure and Borrero 2008: 18.

####### Type locality.

”in republica Aequatorius, in monte Schinchulagua Equador”.

####### Label.

”Woods on the / mountain / Schinchulaqua / Equador”, taxon label in Pfeiffer’s handwriting. M.C. label style IV.

####### Dimensions.

”Long. 34, diam. 17 mill.”. Figured specimen H 37.8, D 18.2, W 6.5.

####### Type material.

NHMUK 1975377, lectotype; 1975378, two paralectotypes (Cuming coll.).

####### Remarks.

Pfeiffer did no indicate on how many specimens his description was based. He described two varieties from ”Chimborazo (Bourcier)”. The second label in the lot indicates ”var.” and ”Chimborazo, Equador / Mons. Bourcier / Consul General”. The systematic position at the species level follows Richardson (1995: 345).

####### Current systematic position.

Bulimulidae, *Kuschelenia (Vermiculatus) aequatorius* (Pfeiffer, 1853) (**comb. n.**).

###### Bulimus aestivus

Pfeiffer, 1857

http://species-id.net/wiki/Bulimus_aestivus

Figs 19A–B , L2iv

Bulimus aestivus Pfeiffer 1857c: 331.
Drymaeus (Drymaeus) aestivus ; Breure 1979: 106.

####### Type locality.

”Meobamba, Peru (*Mr. Gueinzius*)”.

####### Label.

”Ecuador”; taxon label in Pfeiffer’s handwriting. M.C. label style I.

####### Dimensions.

”Long. 17, diam. 7 1/3 mill.”; syntype H 16.9 D 7.9, W 6.4.

####### Type material.

NHMUK 1975462, syntype (Cuming coll.).

####### Remarks.

Pfeiffer described this taxon from Cuming’s collection, but did not state on how many specimens his description was based. The specimen found corresponds to the shell height given by Pfeiffer. This is a case where the locality information provided by Cuming to the describer (viz. Pfeiffer) has been lost; the label ”Ecuador” on the frontside of the wooden tablet has been added in a later handwriting and may be erroneous.

####### Current systematic position.

Bulimulidae, *Drymaeus (Mesembrinus) aesitvus* (Pfeiffer, 1857) (**comb.n.**).

###### Bulinus affinis

Broderip in Broderip and Sowerby I 1832

http://species-id.net/wiki/Bulinus_affinis

Figs 8A , L3i–ii

Bulinus affinis Broderip in Broderip and Sowerby I 1832b: 106; Sowerby I 1833 [Sowerby I and II 1832–1841]: fig. 30.
Bulimus affinis ; Reeve 1848 [1848–1850]: pl. 23 fig. 154.

####### Type locality.

”in Peruviâ (Mexillones, desert of Atacama)”.

####### Label.

”Peru”; taxon name in Pfeiffer’s handwriting. M.C. label style I.

####### Dimensions.

”long 1, lat. 1/2 poll. [H 25.3, D 12.7 mm]”; figured specimen herein H 27.5, D 12.6, W 8.3.

####### Type material.

NHMUK 20100610, five possible syntypes (Cuming coll.).

####### Remarks.

Broderip described this taxon from the Cuming collection, but did not indicate on how many specimens his description was based. These specimens are herein regarded as possible syntypes; one is corresponding to Reeve’s figure. The presence of a label in Pfeiffer’s handwriting together with specimens apparently seen by Reeve as evidenced by the figured specimen, corroborates the assumption (Breure and Ablett 2011: 5) that—at least in some cases—material from the Cuming collection was either returned after having been sent to Pfeiffer or Pfeiffer made the identification during a visit to London.

####### Current systematic position.

Bulimulidae, *Bostryx affinis* (Broderip in Broderip and Sowerby I 1832).

###### Bostryx (Peroneus) agueroi

Weyrauch, 1960

http://species-id.net/wiki/Bostryx_agueroi

Fig. 4E

Bostryx (Peroneus) agueroi Weyrauch 1960b: 126, pl. 12 figs 39–41; Neubert and Janssen 2004: 198, pl. 9 fig. 96.
Bostryx agueroi Breure 1979: 51.

####### Type locality.

”Mittel-Peru, Fundo Yacca, auf der rechten Seite des Río Cañete, an der Autostraße von Cañete nach Yauyos, 2300 m”.

####### Label.

” WW 1462-A/ 2 Paratypes” ”C-Peru: Yacca, 2300m, Rio Canete, leg. W. Weyrauch”; printed label.

####### Dimensions.

”H. 22,8 D. 8,5”; figured specimen herein H 21.1, D 7.4, W 7.3.

####### Type material.

NHMUK 1975333, two paratypes (ex Weyrauch).

####### Current systematic position.

Bulimulidae, *Bostryx agueroi* Weyrauch, 1960.

###### Bostryx aileenae

Breure, 1978

http://species-id.net/wiki/Bostryx_aileenae

Fig. 5A

Bostryx aileenae Breure 1978: 49, pl. 1 fig. 6; Breure 1979: 51.

####### Type locality.

”Peru, Dept. Lima, Río Cañete valley, 1 km above Puente Auco, 2070 m”.

####### Label.

”Peru, Dept. Lima, Río Cañete valley, 1 km above Puente Auco, 2070 m. Sta. 79”.

####### Dimensions.

”shell height 14.9 mm, diameter 7.6 mm”; figured specimen herein H 13.7, D 7.0, W 5.9.

####### Type material.

NHMUK 1975229, three paratypes (Breure leg.).

####### Current systematic position.

Bulimulidae, *Bostryx aileenae* Breure, 1978.

###### Drymaeus alabastrinus

da Costa, 1906

http://species-id.net/wiki/Drymaeus_alabastrinus

Figs 43J–L , L3iii

Drymaeus alabastrinus da Costa 1906b: 98, pl. 11 fig. 4.
Drymaeus (Drymaeus) alabastrinus ; Breure 1979: 106.

####### Type locality.

”Honda, Colombia”.

####### Label.

”Honda, Colombia”; in da Costa’s handwriting.

####### Dimensions.

”Long. 33, diam. 15 mill.”; figured specimen herein H 33.0, D 15.3, W 7.6.

####### Type material.

NHMUK 1907.11.21.16, holotype (da Costa coll.).

####### Remarks.

da Costa did not clearly state on how many specimens his description was based. However, since he mentioned ”a shell” it may be safely assumed that he had only one specimen at hand.

####### Current systematic position.

Bulimulidae, *Drymaeus (Drymaeus) alabastrinus* da Costa, 1906.

###### Bulimulus (Naesiotus) albemarlensis

Dall, 1917

http://species-id.net/wiki/Bulimulus_albemarlensis

Figs 13F , L3iv

Bulimulus (Naesiotus) albemarlensis Dall 1917b: 377.
Naesiotus albemarlensis ; Breure 1979: 67; Breure and Borrero 2008: 10.

####### Type locality.

[Ecuador, Galápagos, Isla Isabela] ”near Villamil, at 2300 to 3300 feet elevation”.

####### Label.

”Galapagos Isl. / Albemarle Isl.”.

####### Dimensions.

”Length of shell 15; diameter 9 mm”; figured specimen herein H 14.7, D 10.0, W 5.8.

####### Type material.

NHMUK 1937.6.18.13-16, three possible paratypes, California Academy of Sciences Expedition 1905–1906 leg. (ex Schlesch).

####### Remarks.

The type status of these specimens is not fully resolved and is here viewed with much caution, as it remains unclear how Schlesch obtained specimens from the original series.

####### Current systematic position.

Bulimulidae, *Naesiotus albemarlensis* (Dall, 1917).

###### Bulinus albicans

Broderip in Broderip and Sowerby I 1832

http://species-id.net/wiki/Bulinus_albicans

Figs 7I , L3v–vi

Bulinus albicans Broderip in Broderip and Sowerby I 1832b: 105; Sowerby I 1833 [Sowerby I and II 1832–1841]: figs 22–22*.
Bulimus albicans ; Reeve 1848 [1848–1850]: pl. 22 fig. 141.

####### Type locality.

”Copiapo, Chili”.

####### Label.

”Chili”; taxon name in Pfeiffer’s handwriting. M.C. label style I.

####### Dimensions.

”long. 9/12, lat. 7/12 poll. [H 19, D 14.8 mm]”; figured specimen herein H 22.1, D 13.1, W 5.7.

####### Type material.

NHMUK 20100611, five possible syntypes (Cuming coll.).

####### Remarks.

Broderip described this taxon from the Cuming collection, but did not indicate on how many specimens his description was based. These specimens are herein regarded as possible syntypes; one is corresponding to Reeve’s figure. This is another case of material that has been studied by both Reeve and Pfeiffer. See also *affinis* Broderip, 1832.

####### Current systematic position.

Bulimulidae, *Bostryx albicans* (Broderip in Broderip and Sowerby I 1832).

###### Bulimus albicolor

Morelet, 1863

http://species-id.net/wiki/Bulimus_albicolor

Figs 4F , L3vii

Bulimus albicolor Morelet 1863: 199, pl. 11 fig. 9.
Bulimulus (Peronaeus) albicolor ; Pilsbry 1896 [1895–1896]: 148, pl. 46 figs 49–50.
Bostryx albicolor ; Breure 1979: 51.

####### Type locality.

[Peru, Dept. Ayacucho] ”Huanta et de la vallée de l’Apurimac”.

####### Label.

”Pérou / Apurimac, Huanta”; in Morelet’s handwriting.

####### Dimensions.

”Longit 28, diam. 9 mm”; figured specimen herein H 28.2, D 10.92, W 8.3.

####### Type material.

NHMUK 1893.2.4.169–170, two syntypes (Morelet coll.).

####### Remarks.

Further syntype material is present in the MHNG and RBINS collections. See also Breure (2011). The current systematic position follows Richardson (1995: 36).

####### Current systematic position.

Bulimulidae, *Bostryx orophilus* (Morelet, 1860).

###### Bulimus albolabiatus

E.A. Smith, 1877

http://species-id.net/wiki/Bulimus_albolabiatus

Figs 29G–I , L3viii

Bulimus (Drymaeus) albolabiatus E.A. Smith 1877: 363, pl. 39 fig. 4.
Drymaeus albolabiatus ; Pilsbry 1898 [1897–1898]: 201, pl. 36 fig. 37.
Drymaeus (Drymaeus) albolabiatus ; Breure 1979: 106; Breure and Borrero 2008:

####### Type locality.

”Malacatos, South Ecuador” [Prov. Loja].

####### Label.

”Malacatos, S. Ecuador”; both locality and taxon name in Smith’s handwriting.

####### Dimensions.

Not given; figured specimen herein H 34.0, D 14.2, W 6.3.

####### Type material.

NHMUK 1877.3.28.3, holotype.

####### Remarks.

According to the label, a paratype has been ”smashed [in] 1896”. The current systematic position follows Richardson (1995: 96).

####### Current systematic position.

Bulimulidae, *Drymaeus (Drymaeus) albolabiatus* (E.A. Smith, 1877).

###### Bulinus albus

Sowerby I, 1833

http://species-id.net/wiki/Bulinus_albus

Figs 7J , L3ix–x

Bulinus albus Broderip [sic]; Sowerby I 1833 [Sowerby I and II 1832–1841]: fig. 51 [19 July]; Sowerby I 1833b: 73 [20 Sept.] (see remarks).

####### Type locality.

[Chile] ”Copiapo”.

####### Label.

”Chili”. M.C. label style I.

####### Dimensions.

”long. 0.8, lat. 0.5 poll. [H 20.3, D 12.7 mm]”; figured specimen herein H 15.0, D 10.7, W 4.7.

####### Type material.

NHMUK 1842.5.10.149–151, three possible syntypes (Cuming coll.).

####### Remarks.

The name was introduced in Sowerby I 1833 [Sowerby I and II 1832–1841] as ”*B. albus* Brod.”. However, as can be seen in Sowerby I (1833 in Sowerby I and II 1832–1841: 73), the authorship should be credited to G.B. Sowerby I (”G.B.S.”). It is not known if this species was described from the Cuming collection, but this cannot be excluded a priori (see also Breure and Ablett 2011: 3). One specimen is closely resembling Sowerby’s figure. The material is herein considered as possible syntypes. The registration numbers suggest that six specimens were originally present; only three of these could be found.

As mentioned before, the publication dates of Sowerby’s publications are according to Coan et al. (2013a) and Duncan (1937). Interestingly, d’Orbigny 1838 [1834–1847]: 280 mentioned this taxon as ”*Bulimus albus* Broderip”, with reference to Sowerby I (1833 in Sowerby I and II 1832–1841), and said it had publication date 11 June 1833 [contra Duncan 1937: 78, who reports 20 September 1833]. At the same time he noticed that Duclos had published *Bulimus olorinus* on 15 June 1833 (Duclos 1833: pl. 24). The text pertaining to this plate describes this taxon from ”Chili”, based on a specimen from Duclos’ collection and collected by Gay. He signed the text with ”15 juin 1833”; however, it seems likely from the context that this was the date Duclos finished his description and not the date the text appeared in print. Without additional bibliographical research it seems premature to give priority to Duclos’ taxon. The current systematic position follows Richardson (1995: 15).

####### Current systematic position.

Bulimulidae, *Bostryx albus* (Sowerby I, 1833).

###### Bulimus altoperuvianus

Reeve, 1849

http://species-id.net/wiki/Bulimus_altoperuvianus

Figs 56A–C , L4i

Bulimus altoperuvianus Reeve 1849 [1848–1850]: pl. 72 figs 521a–b; Breure 1979: 100.
Neopetraeus altoperuvianus ; Pilsbry 1898 [1897–1898]: 173, pl. 32 figs 30–31; Breure 1978: 209 (lectotype designation).

####### Type locality.

[Peru, Dept. Amazonas] ”Chachapoyas, Alto-Peru”.

####### Label.

”Province of Caxamarca [Dept. Cajamarca], Peru”. M.C. label style IV, V.

####### Dimensions.

Not given; figured specimen herein H 46.5, D 25.4, W 7.2.

####### Type material.

NHMUK 1975437, lectotype; 1975438, one paralectotype (Cuming coll.).

####### Remarks.

The lectotype corresponds to Reeve’s fig. 521a, while fig. 521b corresponds to the paralectotype.

####### Current systematic position.

Bulimuludae, *Neopetraeus altoperuvianus* (Reeve, 1849).

###### Bulimus amandus

Pfeiffer, 1855

http://species-id.net/wiki/Bulimus_amandus

Figs 19C , L4ii

Bulimus amandus Pfeiffer 1855d: 96, pl. 31 fig. 4; Breure 1979: 117 (lectotype designation).
Drymaeus (Mesembrinus) amandus ; Breure and Eskens 1981: 50.

####### Type locality.

”Venezuela”.

####### Label.

No locality given. No taxon label in Pfeiffer’s handwriting.

####### Dimensions.

”Long. 30, diam. 11 1/2 mill.”; lectotype H 29, D 13.1, W 6.3.

####### Type material.

NHMUK 1997457, lectotype (Cuming coll.).

####### Remarks.

The lectotype corresponds to Pfeiffer’s figure, although the colours have faded. The current systematic position corresponds to Richardson (1995: 97).

####### Current systematic position.

*Drymaeus (Mesembrinus) amandus* (Pfeiffer, 1855).

###### Bulimus ambustus

Reeve, 1849

http://species-id.net/wiki/Bulimus_ambustus

Figs 40J–L , L4iii

Bulimus ambustus Reeve 1849 [1848–1850]: pl. 74 fig. 535.
Drymaeus ambustus ; Pilsbry 1898 [1897–1898]: 264, pl. 46 figs 66–67.
Drymaeus (Drymaeus) ambustus ; Breure and Eskens 1981: 5.

####### Type locality.

”—?”.

####### Label.

[Ecuador] ”Between Jacunga [Prov. Cotapaxi, Latacunga] and / [Prov. Tungurahua] Ambato”, taxon label in Pfeiffer’s handwriting. M.C. label style IV, V.

####### Dimensions.

Not given; figured specimen herein H 29.0, D 13.1, W 6.3.

####### Type material.

NHMUK 1975441/1–3, lectotype and two paralectotypes, Bourcier leg. (Cuming coll.).

####### Remarks.

Although the label given is more precise than the locality given in Reeve’s publication and the specimens bear a label in Pfeiffer’s handwriting, one of the shells does corresponds to the specimen figured by Reeve. The current systematic position follows Richardson (1995: 97).

####### Current systematic position.

*Drymaeus (Drymaeus) ambustus* (Reeve, 1849).

###### Bulimus andicola

Pfeiffer, 1847

http://species-id.net/wiki/Bulimus_andicola

Figs 23A , L4iv

Bulimus andicola Pfeiffer 1847: 115; Reeve 1848 [1848–1850]: pl. 55 fig. 364; Breure 1979: 117 (lectotype designation).
Bulimulus (Lissoacme) andicola ; Pilsbry 1896 [1895–1896]: 166, pl. 50 fig. 46.

####### Type locality.

”Columbian Andes”.

####### Label.

”Andes of Columbia”, taxon label in Pfeiffer’s handwriting. M.C. label style IV, V.

####### Dimensions.

”Long. 24, diam. 11 mill.”; figured specimen herein H 24.1, D 10.7, W 7.1.

####### Type material.

NHMUK 1975315, lectotype; NHMUK 1975316, two paralectotypes (Cuming coll.).

####### Current systematic position.

*Drymaeus (Mesembrinus) andicola* (Pfeiffer, 1847).

###### Bulimus andoicus

Morelet, 1863

http://species-id.net/wiki/Bulimus_andoicus

Figs 10G , L4v

Bulimus andoicus Morelet 1863: 198, pl. 11 fig. 13; Breure 1979: 50.
Bulimulus (Lissoacme) andoicus ; Pilsbry 1896 [1895–1896]: 147, pl. 46 figs 42–44.
Bostryx andoicus ; Breure 1978: 50 (lectotype designation).

####### Type locality.

[Peru] ”vallées (..) d’Ayacucho”.

####### Label.

”Pérou, Pomacoche”, in Morelet’s handwriting.

####### Dimensions.

”Long. 26–30; diam. 9–10 mill.”; figured specimen herein H 29.5, D 12.12, W 7+.

####### Type material.

NHMUK 1893.2.4.171, lectotype (Morelet coll.).

####### Remarks.

The type locality was broadly defined by Morelet and probably also covers adjacent parts of Dept. Apurimac. There are two places called ”Pomacocha” in the valley of Río Apurimac, one in Dept. Ayacucho and the other in Dept. Apurimac. Given the range in dimensions given by Morelet, it is clear that he had a larger type series at hand. The top of the lectotype is slightly damaged. There are additional lots in the MHNG collection, as described by Breure 1978. The current systematic position corresponds to Richardson (1995: 16).

####### Current systematic position.

Bulimulidae, *Bostryx andoicus* (Morelet, 1863).

###### Drymaeus angustus

da Costa, 1906

http://species-id.net/wiki/Drymaeus_angustus

Figs 37L–N , L4vi

Drymaeus angustus da Costa 1906a: 9, pl. 1 figs 7–8; Linares and Vera 2012: 181.
Drymaeus (Drymaeus) angusta [sic]; Breure 1979: 106.

####### Type locality.

[Colombia] Bogotá.

####### Label.

”Bogota”; in da Costa’s handwriting.

####### Dimensions.

”Long. 31.5, diam. 10”; figured specimen herein H 31.5, D 10.4, W 6.5.

####### Type material.

NHMUK 1907.11.21.14, holotype (da Costa coll.).

####### Remarks.

da Costa wrote ”this unique specimen”, which makes the specimen found the holotype. The taxon name on the label reads ”*angusta*”, but the name ”*angustus*” was published. The top of the holotype is slightly damaged.

####### Current systematic position.

Bulimulidae, *Drymaeus (Drymaeus) angustus angustus* da Costa, 1906.

###### Bulimus anthisanensis

Pfeiffer, 1853

http://species-id.net/wiki/Bulimus_anthisanensis

Figs 69E–F , 69I , L5i

Bulimus anthisanensis Pfeiffer 1853d: 406; Pfeiffer 1854 in Küster and Pfeiffer 1840–1865: 104, pl. 33 figs 20–21; Breure 1979: 85.
Bulimulus anthisanensis ; Pilsbry 1897 [1897–1898]: 32, pl. 4 figs 41–42.
Scutalus (Vermiculatus) anthisanensis ; Breure 1978: 170, pl. 8 fig. 8 (lectotype designation); Breure and Borrero 2008: 18.

####### Type locality.

[Ecuador, Prov. Napo] ”monte Anthisana (44000’ [sic]) reipublicae Aequatoris (*Bourcier*)”.

####### Label.

”the Anthisana / 14000 feet high / Mons^r^ Bourcier / Consul General”; taxon name in Pfeiffer’s handwriting. M.C. label style IV.

####### Dimensions.

”Long. 40, diam. 17 mill. (Mus. Cuming)”; figured specimen herein H 40.2, D 19.5, W 6.6.

####### Type material.

NHMUK 1975372, lectotype; 1975373, three paralectotypes (Cuming coll.).

####### Remarks.

The published altitude at which the type material was collected is clearly in error, as evidenced by the label information.

####### Current systematic position.

Bulimulidae, *Kuschelenia (Vermiculatus) anthisanensis* (Pfeiffer, 1853) (**comb. n.**).

###### Bulimus antioquensis

Pfeiffer, 1855

http://species-id.net/wiki/Bulimus_antioquensis

Figs 31D–F , L5ii

Bulimus antioquensis Pfeiffer 1855b: 291; Pfeiffer 1859b: 394; Breure 1979: 106 (lectotype designation).
Drymaeus (Drymaeus) antioquensis ; Breure and Eskens 1981: 6.
Drymaeus antioquensis ; Linares and Vera 2012: 181.

####### Type locality.

[Colombia] ”Province of Antioquia, New Granada. (*Schlim*)”.

####### Label.

”Province of Antioquia / Mons^r^ Schlim”; taxon name in Pfeiffer’s handwriting. M.C. label style I.

####### Dimensions.

”Long. 30, diam. 13 mill.”; figured specimen herein H 29.6, D 16.4, W 6.1.

####### Type material.

NHMUK 1975450, lectotype, Schlim leg. (Cuming coll.).

####### Remarks.

This taxon is now considered a junior subjective synonym of *Bulimus baranguillanus* Pfeiffer, 1853 (**syn. n.**). The current systematic position is based on a revision of Colombian *Drymaeus* species (Breure and Borrero, unpublished data).

####### Current systematic position.

Bulimulidae, *Drymaeus (Drymaeus) baranguillanus* (Pfeiffer, 1853).

###### Bulimus apertus

Pfeiffer, 1855

http://species-id.net/wiki/Bulimus_apertus

Figs 15D , L5iii

Bulimus apertus Pfeiffer in Dunker et al. 1855: 107.
Naesiotus apertus Breure 1979: 68 (lectotype designation).

####### Type locality.

”?”.

####### Label.

”Andes of Peru”; taxon name in Pfeiffer’s handwriting. M.C. label style I.

####### Dimensions.

”Long. 19, diam. 10 mill.”; figured specimen herein H 18.1 D 9.23 W 6.5.

####### Type material.

NHMUK 1975317, lectotype (Cuming coll.).

####### Remarks.

This is the first time a locality for this taxon is published; *Bulimus apertus* is now considered a junior subjective synonym of *Bulimus orbignyi* Pfeiffer, 1846 (**syn. n.**), whose type locality is ”Bolivia” (see under *Bulimus orbignyi* Pfeiffer). Any specific Peruvian locality for this taxon may likely be expected in one of the southern departments, close to the Bolivian border.

####### Current systematic position.

Bulimulidae, *Naesiotus orbignyi* (Pfeiffer, 1846).

###### Bulimulus apicepunctata

Preston, 1914

http://species-id.net/wiki/Bulimulus_apicepunctata

Figs 23J–K , L5iv

Bulimulus apicepunctata Preston 1914: 523; Breure 1979: 117.
Drymaeus (Mesembrinus) apicepunctata ; Breure and Eskens 1981: 52.

####### Type locality.

”Santa Rita, E. Peru”.

####### Label.

”Santa Rita, E. Peru”.

####### Dimensions.

”Alt. 17.5, diam. maj. 9, diam. min. 7 mm”; figured specimen herein H 17.5, D 8.3, W 6.6.

####### Type material.

NHMUK 1915.1.6.23, holotype (ex Preston).

####### Remarks.

Preston did not state on how many specimens his description was based; the reference of Breure (1979) ”HT 1915.1.6.23” has to be interpreted as lectotype designation under Art. 74.6 ICZN. This is the first time the type has been figured.

####### Current systematic position.

Bulimulidae, *Drymaeus (Mesembrinus) apicepunctata* (Preseton, 1914).

###### Bulimus apiculatus

J.E. Gray, 1834

http://species-id.net/wiki/Bulimus_apiculatus

Figs 23B , L5v

Bulimus apiculatus J.E. Gray 1834: 66; Breure 1979: 117.

####### Type locality.

Not given.

####### Label.

No locality given.

####### Dimensions.

”Axis 10, diam. 4 1/4 lin.” [H 21.1, D 9.0 mm]; figured specimen herein H 20.2, D 9.5, W 6.2.

####### Type material.

NHMUK 1982297, holotype (see remarks).

####### Remarks.

Gray (1834) refers to the type material as ”This shell...”, which may imply that he had only one specimen at hand. It is a juvenile shell, with the lip damaged. The taxon was considered to be a junior subjective synonym of *Helix elongata* Röding, 1789 by Pilsbry (1899: 25), with which we concur.

####### Current systematic position.

Bulimulidae, *Drymaeus (Mesembrinus) elongatus* (Röding, 1789).

###### Helix apodemeta

d’Orbigny, 1835

http://species-id.net/wiki/Helix_apodemeta

Figs 9A–B , L5vi

Helix apodemeta d’Orbigny 1835: 10.
Bulimus apodemetes d’Orbigny 1837 [1834–1847]: 279, pl. 30 figs 5–8 [3 April 1837; text 23 April 1838]; Gray 1854: 16.
Bulimulus (Lissoacme) apodemetus ; Pilsbry 1896 [1895–1896]: 187, pl. 51 fig. 1–3.
Bulimulus (Bulimulus) apodemetus ; Breure 1975b: 1145.

####### Type locality.

”republica Argentina; republica Boliviana”; see Breure 1973: 114.

####### Label.

”Monte grande, chiquitos (Bolivia)” [.178,. 181]; ”Pampa ruis (laguna) Bolivia” [.179]; ”Vilna (Valle grande) Bolivia” [.180]; ”S^n^ Lorenzo Parana Rep. argentina” [.182], all in in d’Orbigny’s handwriting.

####### Dimensions.

”Assez variables; les plus along´s ont, de longueur, 28 millimètres sur 18 de largeur, tandis que les plus courts offerent 23 millimètres de longueur, sur 12 de largeur”; largest specimen H 28.3, shortest H 22.7, figured specimen H 23.0, D 12.6, W 6.2 [.182].

####### Type material.

NHMUK 1854.12.4.178–182, five lots with resp. eight, seven, seven, three and three syntypes (d’Orbigny coll.).

####### Remarks.

d’Orbigny (1935) did not state on how many specimens his description was based. In d’Orbigny (1838 [1834–1847]: 280) the localities were specified as ”sur les coteaux du Parna, province d’Entre-rios, près de Feliciano (...) près de San-Lorenzo, province de Santa-Fe. (...) la république de Bolivia (...) des provinces de Valle grande et de la Laguna (...); (...) des plaines de Santa-Cruz de la Sierra, (...) la province de Chiquitos”; see also Breure 1973. The shell of the largest specimen among the lots, in. 178, is broken on the ventral side. The protoconch of the type material proves to be smooth and the taxon is now being transferred from the genus *Bulimulus* Leach, 1814 to *Bostryx* Troschel, 1847 sensu Breure 1979 (but see Breure 2012b).

####### Current systematic position.

Bulimulidae, *Bostryx apodemetus* (d’Orbigny, 1835) (**comb. n.**).

###### Bulimus aquilus

Reeve, 1848

http://species-id.net/wiki/Bulimus_aquilus

Figs 70A–B , 70K , L6i

Bulimus aquilus Reeve 1848 [1848–1850]: pl. 22 fig. 138; Breure 1979: 85.
Bulimulus aquilus ; Pilsbry 1897 [1897–1898]: 17, pl. 5 figs 72–73.
Scutalus (Vermiculatus) aquilus (Reeve); Breure 1978: 170 (lectotype designation).

####### Type locality.

”Peru, Tacna”.

####### Label.

”Peru”. M.C. label style IV, V.

####### Dimensions.

Not given; figured specimen herein H 27.1, D 14.6, W 5.8.

####### Type material.

NHMUK 1975376, lectotype (Cuming coll.).

####### Remarks.

The current systematic position at the species level follows Richardson (1995: 347).

####### Current systematic position.

Bulimulidae, *Kuschelenia (Vermiculatus) aquilus* (Reeve, 1848) (**comb. n.**).

###### Bulimus arcuatostriatus

Pfeiffer, 1855

http://species-id.net/wiki/Bulimus_arcuatostriatus

Figs 41A–C , L6iii

Bulimus arcuatostriatus Pfeiffer 1855d: 95; 1859: 394; Breure 1979: 106 (lectotype designation).
Drymaeus (Drymaeus) arcuatostriatus ; Breure and Eskens 1981: 6.

####### Type locality.

”Peru”.

####### Label.

”Peru”; taxon label in Pfeiffer’s handwriting. M.C. label style I.

####### Dimensions.

”Long. 30, lat. 13 mill.”; figured specimen herein H 27.6, D 15.6, W 6.5.

####### Type material.

NHMUK 1975455, lectotype (Cuming coll.).

####### Remarks.

The basal lip of the lectotype is damaged. The current systematic position folows Richardson (1995: 99).

####### Current systematic position.

Bulimulidae, *Drymaeus (Drymaeus) arcuatostriatus* (Pfeiffer, 1855).

###### Bulimus atacamensis

Pfeiffer, 1856

http://species-id.net/wiki/Bulimus_atacamensis

Figs 2A–B , L6ii

Bulimus atacamensis Pfeiffer 1856c: 207; Pfeiffer 1859b: 486; Breure 1979: 51.
Bostryx atacamensis ; Breure 1978: 53 (lectotype designation).

####### Type locality.

”Paposo in deserto Atacamensi reipublicae Chilensis. (*R.A. Philippi*)”.

####### Label.

”Desert of Atacama, / Chile // Philippi has sent it”, ”Bolivia” [in a later handwriting]; taxon label in Pfeiffer’s handwriting.

####### Dimensions.

”Long. 19, lat. 5 1/2 mill.”; figured specimen herein H 19.0, D 5.9, W 10.2.

####### Type material.

NHMUK 1975312, lectotype; NHMUK 1975128, two paralectotypes (Cuming coll.).

####### Remarks.

The current systematic position follows Richardson (1995: 17).

####### Current systematic position.

Bulimulidae, *Bostryx atacamensis* (Pfeiffer, 1856).

###### Bulimulus atahualpa

Dohrn, 1863

http://species-id.net/wiki/Bulimulus_atahualpa

Figs 56D–F , L6iv–v

Bulimulus atahualpa Dohrn 1863: 153.
Neopetraeus atahualpa ; Breure 1979: 100.

####### Type locality.

Not given.

####### Label.

”Huallaga”, ”Province of / Patas, Andes of Peru” [see remarks]. M.C. label style IV.

####### Dimensions.

”Long 43, lat 21 (..) mill.” (largest specimen); figured specimen herein H 38.5, D 26.4, W 6+.

####### Type material.

NHMUK 1975436, three possible syntypes (Cuming coll.).

####### Remarks.

Dohrn (1863) gives the measurements of five specimens, but it is unclear how many specimens his description was actually based upon. The specimens in NHMUK are accompanied by two sets of labels. One is in Dohrn’s handwriting (teste S.P. Dance, 1958) and has ”Huallaga” as the locality. The other label set originates from Farris and has ”Province of Patas, Andes of Peru”; the taxon name is in Pfeiffer’s handwriting, who identified it as *Bulimus tessellatus* Shuttleworth, 1852. The latter label may have been added to the material in a later stage. Due to the fact the other label has original handwriting, the specimens are considered possible syntypes. The current systematic position follows Richardson (1995: 239).

####### Current systematic position.

Bulimulidae, *Neopetraeus atahualpa* (Dohrn, 1863).

###### Bulimus attenuatus

Pfeiffer, 1853

http://species-id.net/wiki/Bulimus_attenuatus

Figs 17F–H , L6vi

Bulimus attenuatus Pfeiffer 1853b: 256; Pfeiffer 1853d: 336; Pfeiffer 1853 in Küster and Pfeiffer 1840–1865: 83, pl. 30 figs 9–10; Breure 1979: 106 (lectotype designation).
Drymaeus (Mesembrinus) attenuatus attenuatus ; Thompson 2011: 114.

####### Type locality.

[Mexico] ”Vera Cruz”.

####### Label.

”Vera Cruz”; taxon name in Pfeiffer’s handwriting. M.C. label style IV.

####### Dimensions.

”Long. 34, lat. 13 mill.”; figured specimen herein H 34.0, D 14.4, W 6.1.

####### Type material.

NHMUK 1975458, lectotype; NHMUK 1975459, two paralectotypes (Cuming coll.).

####### Remarks.

The current systematic position follows Thompson (2011).

####### Current systematic position.

Bulimulidae, *Drymaeus (Mesembrinus) attenuatus* (Pfeiffer, 1853).

###### Bulimus aureolus

Guppy, 1866

http://species-id.net/wiki/Bulimus_aureolus

Figs 21A , L7i

Bulimus aureolus Guppy 1866: 49.
Drymaeus (Mesembrinus) aureolus ; Breure 1979: 117.

####### Type locality.

[Trinidad].

####### Label.

”Trinidad”; in Guppy’s handwriting.

####### Dimensions.

”Height 0.6 inch, breadth 0.3 inch” [H 15.2, D 7.6 mm]; figured specimen H 23.2, D 10.71, W 6.2.

####### Type material.

NHMUK 1866.1.3.8, two syntypes (ex Guppy).

####### Remarks.

Guppy described this taxon from immature specimens: ”The specimens from which the species was described by me in the Annals and Magazine of Natural History (1866) were young and imperfect.” (Guppy 1871: 307). There is another shell (NHMUK 1875.2.8.1), also presented by Guppy, which could be from the series he is referring to in the second paper; it is therefore not considered a syntype since this specimen was later collected than the type series, but unfortunately all three specimens have been mixed and there is no way of separating the two lots.

####### Current systematic position.

Bulimulidae, *Drymaeus (Mesembrinus) aureolus* (Guppy, 1866).

###### Bulimus aurifluus

Pfeiffer, 1857

http://species-id.net/wiki/Bulimus_aurifluus

Figs 25A , L6vii

Bulimus aurifluus Pfeiffer 1857b: 319, pl. 35 fig. 10; Pfeiffer 1859b: 400.

####### Type locality.

[Mexico] ”Cordova”.

####### Label.

”Cordova (Mexico)”; taxon name in Pfeiffer’s handwriting. M.C. label style I.

####### Dimensions.

”Long. 22, diam. 10 mill.”; figured specimen herein H 21.8, D 11.1, W 5.1.

####### Type material.

NHMUK 1975502, lectotype; 1975502/2, one paralectotype (Cuming coll.).

####### Remarks.

One of the two specimens found corresponds to the figure of Pfeiffer and is here selected as the lectotype (**design. n.**). The current systematic position follows Thompson (2011: 112).

####### Current systematic position.

Bulimulidae, *Drymaeus (Mesembrinus) aurifluus* (Pfeiffer, 1857).

###### Bulimus auris

Pfeiffer, 1866

http://species-id.net/wiki/Bulimus_auris

Figs 37A–C , L7ii

Bulimus auris Pfeiffer 1866: 831; Pfeiffer 1868: 36; Breure 1979: 106 (lectotype designation).
Drymaeus (Drymaeus) auris ; Breure and Eskens 1981: 8.

####### Type locality.

”Venezuela”.

####### Label.

”Venezuela”; taxon name in Pfeiffer’s handwriting. M.C. label style I.

####### Dimensions.

”Long. 39, diam. 14 1/2 mill.”; figured specimen herein H 38.8, D 20.1, W 6+.

####### Type material.

NHMUK 1975499, lectotype (Cuming coll.).

####### Remarks.

Pfeiffer did not state on how many specimens his description was based. Only one specimen was found, which corresponds to Pfeiffer’s original measurements; the top is damaged.

####### Current systematic position.

Bulimulidae, *Drymaeus (Drymaeus) auris* (Pfeiffer, 1866).

###### Bulinus badius

Sowerby, 1835

http://species-id.net/wiki/Bulinus_badius

Figs 67J , L7iii

Bulinus badius Sowerby I 1835: 141.
Bulimus badius ; Reeve 1849 [1848–1850]: pl. 39 fig. 235.
Bulimulus badius ; Pilsbry 1897 [1897–1898]: 28, pl. 4 fig. 49.

####### Type locality.

”in provincia Peruviae Xagua” (see remarks).

####### Label.

”Prov. of Xagua, Peru”. M.C. label style I.

####### Dimensions.

”long. 1, lat. 0.6 poll. [H 25.3, D 15.2 mm]”; figured specimen herein H 25.8, D 15.5, W 5.9.

####### Type material.

NHMUK 20100631, three possible syntypes (Cuming coll.).

####### Remarks.

The specimen figured by Reeve was found in the collection and, together with two other specimens, are considered possible syntypes. The type locality may refer to Dept. Jauja in Central Peru (teste Crawford 1939: 330) or to Dept. Amazonas, Prov. Bagua; the latter seems slightly outside the range known for this subgenus. The current systematic position at species level follows Richardson (1995: 348).

####### Current systematic position.

Bulimulidae, *Kuschelenia (Vermiculatus) bicolor* (Sowerby I, 1835) (**comb. n.**).

###### Bulimus balsanus

Morelet, 1863

http://species-id.net/wiki/Bulimus_balsanus

Figs 5B , L7iv

Bulimus balsanus Morelet, 1863: 192, pl. 9 fig. 8; Breure 1979: 51.
Bostryx balsanus ; Breure 1978: 53 (lectotype designation).

####### Type locality.

[Peru] ”Balsa de Cocharcas”.

####### Label.

”Balsa de Cocharcas”, in Morelet’s handwriting.

####### Dimensions.

”Long. 19, diam. 8 1/2 mill.”; figured specimen herein H 17.9, D 9.0, W 6.4.

####### Type material.

NHMUK 1893.2.4.173, lectotype; 1893.2.4.174, paralectotype (Morelet coll.).

####### Remarks.

The current systematic position follows Richardson (1995: 35).

####### Current systematic position.

Bulimulidae, *Bostryx nigropileatus* (Reeve, 1849).

###### Bulimus baranguillanus

Pfeiffer, 1853

http://species-id.net/wiki/Bulimus_baranguillanus

Figs 31A–C , L7v

Bulimus baranguillanus Pfeiffer 1853d: 334; Pfeiffer 1853 in Küster and Pfeiffer 1840–1865: 246, pl. 66 figs 5–6; Pfeiffer 1854b: 136; Breure 1979: 106 (lectotype designation).
Drymaeus baranguillanus ; Pilsbry 1898 [1897–1898]: 208, pl. 35 figs 21–22; Linares and Vera 2012: 181.
Drymaeus (Drymaeus) baranguillanus ; Breure and Eskens 1981: 8.

####### Type locality.

”Baranguilla in Andibus Colombianus”.

####### Label.

”Baranguilla / Andes of Colombia”; taxon label in Pfeiffer’s handwriting. M.C. label style IV.

####### Dimensions.

”Long. 32, diam. 13 1/2 mill.”; figured specimen herein H 31.5, D 15.6, W 7.2.

####### Type material.

NHMUK 1975452, lectotype (Cuming coll., ex Bland).

####### Current systematic position.

Bulimulidae, *Drymaeus (Drymaeus) baranguillanus* (Pfeiffer, 1853).

###### Bulimus barbadensis

Pfeiffer, 1853

http://species-id.net/wiki/Bulimus_barbadensis

Figs 60A–B , L8i

Bulimus barbadensis Pfeiffer 1853d: 435; Breure 1979: 62 (lectotype designation).
Bulimulus fuscus (Guilding, 1828); Breure 1974: 38, pl. 5 figs 1–4.

####### Type locality.

[West Indies] ”Barbados. (Mus. Cuming)”.

####### Label.

No locality given. The taxon label is in Pfeiffer’s handwriting. M.C. label style I.

####### Dimensions.

”Long. 20, diam. 10 mill.”; figured specimen herein H 21.3, D 10.8 W 5.9.

####### Type material.

NHMUK 1974054, lectotype; 1974055, two paralectotypes (Cuming coll.).

####### Remarks.

The current systematic position follows the revison of Breure (1974).

####### Current systematic position.

Bulimulidae, *Bulimulus fuscus* (Guilding, 1828).

###### Helix (Xenothauma) baroni

Fulton, 1896

http://species-id.net/wiki/Helix_baroni

Figs 65A–B , L8iii

Helix (Xenothauma) baroni Fulton 1896: 101, pl. 6 figs 7–7b; Breure 1979: 83; Neubert and Janssen 2004: 201, pl. 11 fig. 135.
Bulimulus baroni ; Pilsbry 1898 [1897–1898]: 172, pl. 25 figs 64–66.
Scutalus (Scutalus) baroni ; Breure 1978: 164 (lectotype designation); Köhler 2007: 147, fig. 61.

####### Type locality.

[Peru, Dept. Cajamarca] ”Rio Yonan, Peru, 4000 feet (*C.T. Baron*)”.

####### Label.

”Rio Yonan, Peru, 4000’ / C.T. Baron”, in Fulton’s handwriting.

####### Dimensions.

”Height 12 millim., maj. diam. 30 millim.”; figured specimen herein H 12.7, D 29.3, W 4.6.

####### Type material.

NHMUK 1896.6.23.1, lectotype; 1896.6.23.2, one paralectotype, Baron leg. (ex Fulton).

####### Remarks.

Fulton did not mention on how many specimens his description was based. From Neubert and Janssen (2004: 201) it may be concluded that the type series was distributed to various museums. ”Rio Yonan” is the local name of Rio Jequetepeque at the village of Yonan, near Tembladera (V. Mogollón, pers. commun.).

####### Current systematic position.

Bulimulidae, *Scutalus baroni* (Fulton, 1896).

###### Bulimulus (Drymaeus) baroni

Fulton, 1897

http://species-id.net/wiki/Bulimulus_baroni

Figs 65E–F , L8ii

Bulimulus (Drymaeus) baroni Fulton 1897: 213, pl. 6 fig. 8–8b; Breure 1979: 83.
Scutalus (Scutalus) baroni ; Breure 1978: 164 (lectotype designation).

####### Type locality.

”Rio Yonan, Peru, 4000 feet (*C.T. Baron*)”.

####### Label.

”4000 ft / Rio Yonan / Peru”, in Fulton’s handwriting.

####### Dimensions.

”Long. 31–36 millim., maj. diam. 19–22 millim.”; figured specimen herein H 34.3, D 23.2, W 6.3.

####### Type material.

NHMUK 1897.8.3.173, lectotype; 1897.8.3.179, one paralectotype, Baron leg. (ex Fulton).

####### Remarks.

Fulton states ”Type specimens (three) in British Museum”. Only two specimens have been found. This taxon is considered a junior subjective synonym of *Scutalus cretaceus* (Pfeiffer, 1855) by Breure (1978).

####### Current systematic position.

Bulimulidae, *Scutalus cretaceus* (Pfeiffer, 1855).

###### Otostomus bartletti

H. Adams, 1867

http://species-id.net/wiki/Otostomus_bartletti

Figs 41G–I , L8iv

Otostomus bartletti H. Adams 1867a: 442, pl. 38 fig. 4; Breure 1979: 106.
Drymaeus (Drymaeus) bartletti ; Pilsbry 1898 [1897–1898]: 224, pl. 32 fig. 44.

####### Type locality.

”[Upper Amazons, and on the River Ucayali, Eastern Peru]”.

####### Label.

”E. Peru”; ”Upper Amazons” added in a later handwriting. Taxon label in H. Adam’s handwriting.

####### Dimensions.

”Long. 25 mill., diam. maj. 26 mill.”; figured specimen herein H 28.5, D 26.7, W 4+.

####### Type material.

NHMUK 1867.5.18.4, lectotype, Bartlett leg.

####### Remarks.

The type locality was not specified for each species, but is taken from the title of Adam’s paper. ”Two examples, one in good condition, of this very finely marked and interesting species were obtained by Mr. Bartlett” (H. Adams 1867a: 442). Only one specimen is present, of which the index card in the collection states ”The specimen, although not registered, appears to be 1867.5.18.4 which was purchased from Bartlett along with other types described at the same time. No specimen can be found with this number. The writings on the board are the same as those on the other specimens puchased at the same time, including the original labels. Locality exactly correct”. The specimen, of which the top is damaged, is the figured specimen by Adam and is here designated lectotype (**design. n.**).

####### Current systematic position.

Bulimulidae, *Drymaeus (Drymaeus) bartletti* (H. Adams, 1867).

###### Drymaeus bellus

da Costa, 1906

http://species-id.net/wiki/Drymaeus_bellus

Figs 28H–J , L8v

Drymaeus bellus da Costa 1906a: 8, pl. 1 fig. 5; Breure 1979: 106; Linares and Vera 2012: 181.

####### Type locality.

”Colombia, San Martin”.

####### Label.

”San Martin, Colombia”, in da Costa’s handwriting.

####### Dimensions.

”Long. 33, diam. 16 mm.”; figured specimen herein H 33.2, D 16.6, W 6.3.

####### Type material.

NHMUK 1907.11.21.8, holotype; 1907.11.21.9, paratype (da Costa coll.).

####### Remarks.

The current systematic position follows the revision of Breure and Borrero (unpublished data) and da Costa’s taxon is now placed in the synonymy of *Drymaeus blandi* Pilsbry, 1897 (**syn. n.**).

####### Current systematic position.

Bulimulidae, *Drymaeus (Drymaeus) blandi* Pilsbry, 1897.

###### Bulinus bicolor

Sowerby I, 1835

http://species-id.net/wiki/Bulinus_bicolor

Figs 66O–P , L9i

Bulinus bicolor Sowerby I 1835: 141; Reeve 1848 [1848–1850]: pl. 44 fig. 276; Breure 1979: 85.
Scutalus (Vermiculatus) bicolor ; Breure 1978: 171 (lectotype designation).

####### Type locality.

”provinciae Peruviae Xagua” (see remarks).

####### Label.

”Peru” on two labels with different handwriting, ”Zagua” written on board. M.C. label style I, V.

####### Dimensions.

”Long. 0.9, lat. 0.4 poll. [H 22.8, D 10.1 mm]”; figured specimen herein H 23.6, D 10.9, W 5.8.

####### Type material.

NHMUK 1975151 lectotype; 1975152, two paralectotypes (Cuming coll.).

####### Remarks.

Sowerby described this taxon from the Cuming collection. So far the publication date for this taxon has been cited as 1834 or 1835. According to Duncan (1937: 78) Sowerby’s paper was published on 3 April 1835. The type locality has not be found in any modern gazetteer. Weyrauch (1967: 386) simply lists this species as from Peru, but distinguished *Scutalus bicolor badius* (Sowerby I, 1835) from ”N-Peru”. According to Crawford (1939: 330) the name ”Xagua” could refer to Dept. Jauja, which is in Central Peru. The specimen figured by Reeve has been selected lectotype by Breure (1978). The current systematic position at the species level follows Richardson (1995: 348).

####### Current systematic position.

Bulimulidae, *Kuschelenia (Vermiculatus) bicolor* (Sowerby I, 1835) (**comb. n.**).

###### Bulimus binneyanus

Pfeiffer, 1857

http://species-id.net/wiki/Bulimus_binneyanus

Figs 58C–E , L9ii

Bulimus binneayanus Pfeiffer 1857e: 229; Pfeiffer 1858a: 257, pl. 42 fig. 4; Breure 1979: 100.
Neopetraeus binneyanus ; Pilsbry 1898 [1897–1898]: 164, pl. 32 fig. 43; Breure 1978: 210 (lectotype designation).

####### Type locality.

”in Andibus prov. Patas, Peru (*Dr. Farris*)”.

####### Label.

”Province of Patas / Andes of Peru”; taxon name in Pfeiffer’s handwriting. M.C. label style I.

####### Dimensions.

”Long. 26, diam. 19 mill.”; figured specimen herein H 25.2, D 20.8, W 5.2.

####### Type material.

NHMUK 1975426, lectotype; 1975427, two paralectotypes (Cuming coll.).

####### Remarks.

Pfeiffer did not state on how many specimens his description was based; from Pfeiffer 1858 it is clear this he described this taxon from the Cuming collection. The specimen originally figured by Pfeiffer (1858a: pl. 42 fig. 4) was selected lectotype by Breure (1978).

####### Current systematic position.

Bulimulidae, *Neopetraeus binneyanus* (Pfeiffer, 1857).

###### Bulimus bogotensis

Pfeiffer, 1855

http://species-id.net/wiki/Bulimus_bogotensis

Figs 42A–C , L9iii

Bulimus bogotensis Pfeiffer 1855d: 93; Pfeiffer 1859b: 390; Breure 1979: 106 (lectotype designation).
Drymaeus bogotensis ; Linares and Vera 2012: 182 [partim].
Drymaeus (Drymaeus) bogotensis ; Breure and Eskens 1981: 10.

####### Type locality.

[Colombia] ”Santa Fé de Bogota”.

####### Label.

”Santa Fe da Bogata [sic]”; taxon name in Pfeiffer’s handwriting. M.C. label style III.

####### Dimensions.

”Long. 38, diam. 14 mill.”; figured specimen herein H 37.4, D 17.5, W 6.9.

####### Type material.

NHMUK 1975191, lectotype (Cuming coll.).

####### Remarks.

Pfeiffer described this taxon from Cuming’s collection; he did not state on how many specimens his description was based. Only one specimen was found. On the original label, Pfeiffer has added ”(spectatus Reeve t. 81. / f. 601 b.)”. Reeve described *Bulimus spectatus* from specimens in ”Mus. Taylor”; his figure does not correspond to the specimen designated as lectotype and we consider Reeve’s taxon to be distinct.

####### Current systematic position.

Bulimulidae, *Drymaeus (Drymaeus) bogotensis* (Pfeiffer, 1855).

###### Helix bolivarii

d’Orbigny, 1835

http://species-id.net/wiki/Helix_bolivarii

Figs 52A–B

Helix bolivarii d’Orbigny 1835: 17.
Bulimus bolivarii d’Orbigny 1837 [1834–1847]: 309, pl. 39 figs 5–6 [7 Aug. 1837; text 6 May 1838].
Drymaeus bolivarii (d’Orbigny); Breure 1975b: 1150, pl. 4 fig. 2 (lectotype designation).

####### Type locality.

”provincia Cochabambacensi (republica Boliviana); see Breure 1973: 114.

####### Label.

Not found.

####### Dimensions.

”Longit. 54 millim., latit. 26 millim.”.

####### Type material.

Not found.

####### Remarks.

No material of this species was found. In the NHMUK-copy of the ”Voyage...” a note on p. 309 states ”No examples of this sp. could be traced in d’O. coll^n^ in Sept. 1958. S.P. Dance”. Also, in Gray (1854) there is no ”B.M.” following the name, so it may safely assumed that type material of this taxon never came to London. The only extant material is thus the material in the Paris museum. As this is an incomplete specimen, the original figures by d’Orbigny are here copied.

####### Current systematic position.

Bulimulidae, *Drymaeus (Drymaeus) bolivarii* (d’Orbigny, 1835).

###### Bulimus bolivianus

Pfeiffer, 1846

http://species-id.net/wiki/Bulimus_bolivianus

Figs 42D–F , L9iv

Bulimus bolivianus Pfeiffer 1846a: 34; Pfeiffer 1848b: 105; Reeve 1849 [1848–1850]: pl. 81 fig. 599.
Drymaeus bolivianus ; Pilsbry 1898 [1897–1898]: 244, pl. 43 fig. 66.
Drymaeus (Drymaeus) bolivianus ; Breure 1979: 107 (lectotype designation); Breure and Eskens 1981: 12.

####### Type locality.

[Venezuela] ”Merida, Andes of Bolivia”.

####### Label.

”Merida”, taxon label in Pfeiffer’s handwriting. M.C. label style IV.

####### Dimensions.

”Long. 33, diam. 13 mill.”; figured specimen herein H 33, D 13.7, W 7.2.

####### Type material.

NHMUK 1975444, lectotype; 1975445, one paralectotype (Cuming coll.); see remarks.

####### Remarks.

Pfeiffer did not state on how many specimens his description was based; the lot found contains three specimens. The smallest shell of the lot is a juvenile specimen not belonging to this species, and is here excluded from the type material. The locality as originally stated by Pfeiffer seems in error as there is no place with that name in Bolivia; the taxon is now tentatively assigned to the Venezuelan malacofauna. Reeve 1848 [1848–1850]: pl. 44 fig. 281 figured a specimen from ”Mus. Dennison”, which he mistakenly held for *Bulimus bolivianus*. Later he corrected his mistake on pl. 81, stating ”The shell here represented is the original type of the species described by Dr. Pfeiffer. To that which I have mistaken for it at Pl. XLIV Sp. 281, the name *B. annulatus* may be given”. The current systematic position follows Richardson (1995: 105).

####### Current systematic position.

Bulimulidae, *Drymaeus (Drymaeus) bolivianus* (Pfeiffer, 1846).

###### Drymaeus boucardi

da Costa, 1907

http://species-id.net/wiki/Drymaeus_boucardi

Figs 36A–C , L9v

Drymaeus boucardi da Costa 1907: 305, pl. 26 figs 5–5a; Breure 1979: 107.

####### Type locality.

[Panama] ”Chiriqui”.

####### Label.

”Chiriqui”, in da Costa’s handwriting.

####### Dimensions.

”Long. 27, diam. 11 mm.”; figured specimen herein H 26.9, D 12.4, W 5.9.

####### Type material.

NHMUK 1907.11.21.26, holotype, Boucard leg. (da Costa coll.).

####### Remarks.

In his remarks, da Costa states ”this shell’, which might imply that he had only one specimen. The material is considered herein a holotype. The basal part of the lip is damaged. This taxon has not been mentioned by Thompson (2011).

####### Current systematic position.

Bulimulidae, *Drymaeus (Drymaeus) boucardi* da Costa, 1907.

###### Bulimus bourcieri

Pfeiffer, 1853

http://species-id.net/wiki/Bulimus_bourcieri

Figs 26G–I , L9vi

Bulimus bourcieri Pfeiffer 1853d: 314; Pfeiffer 1854 in Küster and Pfeiffer 1840–1865: 98, pl. 32 figs 3–4.
Drymaeus bourcieri ; Pilsbry 1898 [1897–1898]: 241, pl. 33 fig. 45.
Drymaeus (Drymaeus) bourcieri ; Breure 1979: 107 (lectotype designation); Breure and Eskens 1981: 12; Breure and Borrero 2008: 20.

####### Type locality.

”Pichincha reipublicae Aequatoris (*Bourcier*)”.

####### Label.

”Pichincha / Ecuador / Mons^r^ Bourcier”, taxon label in Pfeiffer’s handwriting. M.C. label style IV.

####### Dimensions.

”Long. 25, diam. 11 mill.”; figured specimen herein H 23.9, D 13.0, W 5.5.

####### Type material.

NHMUK 1975446, lectotype; 1975447, two paralectotypes, Bourcier leg. (Cuming coll.).

####### Current systematic position.

Bulimulidae, *Drymaeus (Drymaeus) bourcieri* (Pfeiffer, 1853).

###### Helix brachysoma

d’Orbigny, 1835

http://species-id.net/wiki/Helix_brachysoma

Figs 52C–D

Helix brachysoma d’Orbigny 1835: 18.
Bulimus brachysoma d’Orbigny 1837 [1834–1847]: 309, pl. 39 figs 9–10 [7 Aug. 1837; text 6 May 1838].

####### Type locality.

”provincia Santa Cruz de la Sierra (republica Boliviana)”; see Breure 1973: 115.

####### Dimensions.

”Longit. 40 millim., latit. 19 millim.”.

####### Remarks.

In the NHMUK copy of the ”Voyage...”, there is a note in pencil in the margin of p. 309, which states ”No examples of this sp. could be traced in d’O. colln. in Sept. 1958. S.P. Dance”. Also, in Gray (1854) there is no ”B.M.” following the name, so it may safely assumed that type material of this taxon never came to London. As this species has not been traced in the Paris museum (Breure 1975b), the type material must be considered lost. The original figures of d’Orbigny are here reproduced.

####### Current systematic position.

Bulimulidae, *Drymaeus (Drymaeus) brachysoma* (d’Orbigny 1835).

###### Bulimulus (Drymaeus) broadwayi

E.A. Smith, 1896

http://species-id.net/wiki/Bulimulus_broadwayi

Figs 20I , L9vii

Bulimulus (Drymaeus) broadwayi E.A. Smith 1896: 243, pl. 8 fig. 9.
Drymaeus broadwayi ; Pilsbry 1899: 22, pl. 12 fig. 5.
Drymaeus (Mesembrinus) broadwayi ; Breure 1979: 117.

####### Type locality.

[Trinidad].

####### Label.

”Trinidad”.

####### Dimensions.

”Longit. 14 millim., diam. 8 1/2”; holotype H 11.9, D 7.59, W 5.4.

####### Type material.

NHMUK 1895.11.28.10, holotype, W.E. Broadway leg. (ex W. Moss).

####### Remarks.

Smith remarked ”Mr. Moss, who received from him [Broadway] a fine series (...), has liberally placed one of his three specimens of this species in the British Museum collection”. The specimen found is thus the holotype, despite the fact it is somewhat smaller than the dimensions given by Smith.

####### Current systematic position.

Bulimulidae, *Drymaeus (Mesembrinus) broadwayi* (E.A. Smith, 1896).

###### Bulimulus (Drymaeus) buckleyi

Sowerby III, 1895

http://species-id.net/wiki/Bulimulus_buckleyi

Figs 36D–F , L10i

Bulimulus (Drymaeus) buckleyi Sowerby III 1895: 214, pl. 13 figs 3–4.
Drymaeus (Drymaeus) buckleyi ; Breure 1979: 107 (lectotype designation); Breure and Eskens 1981: 12; Breure and Borrero 2008: 20.

####### Type locality.

”Ecuador”.

####### Label.

”Ecuador”. The label also mentions ”2 co-types”.

####### Dimensions.

”Long. 27, diam. 9 mm.”; figured specimen herein H 27.3, D 11.1, W 5.7.

####### Type material.

NHMUK1907.11.21.48, lectotype; 1907.11.21.49–50, two paralectotypes (da Costa coll.).

####### Remarks.

Sowerby (1895) stated: ”Types in the collection of Mr. S.I. Da Costa. The shells were collected by Mr. C. Buckley in 1872, but not mentioned in Mr. E.T. Higgins’ paper [Higgins 1872]”.

####### Current systematic position.

Bulimulidae, *Drymaeus (Drymaeus) buckleyi* (Sowerby III, 1895).

###### Otostomus bugabensis

Martens, 1893

http://species-id.net/wiki/Otostomus_bugabensis

Figs 25B , L10ii

Otostomus bugabensis Martens 1893 [1890–1901]: 218, pl. 13 figs 21–21a.
Drymaeus bugabensis ; Pilsbry 1899: 64, pl. 13 figs 34–35.
Drymaeus (Mesembrinus) bugabensis ; Breure 1979: 117 (lectotype designation); Breure and Eskens 1981: 54, pl. 6 fig. 4; Thompson 2011: 114.

####### Type locality.

”S. Panama, Bugaba, Department of Chiriqui, at an elevation of 1000 feet (*Champion*)”.

####### Label.

”Bugaba Panama” on two labels in Martens’ handwriting, one of which states ”Champion 166”.

####### Dimensions.

”Long. 27, diam. 11 millim.”; lectotype H 26.3, D 11.0, W 5.8 (see remarks).

####### Type material.

NHMUK 1901.6.22.958, lectotype; 1901.6.22.820, paralectotype, Champion leg. (Godman coll.).

####### Remarks.

The lectotype has a damaged palatal lip and could be only subadult. Both shells are discoloured by age. The current systematic position is according to Thompson (2011).

####### Current systematic position.

Bulimulidae, *Drymaeus (Mesembrinus) bugabensis* (Martens, 1893).

###### Bulimus cacticolus

Reeve, 1849

http://species-id.net/wiki/Bulimus_cacticolus

Figs 60E–F , L10iii

Bulimus cacticolus Reeve 1849 [1848–1850]: pl. 58 fig. 393.

####### Type locality.

”Curiana, Venezuela. (Mus. Dyson)”.

####### Label.

”Curiana, Venezuela”, in a later handwriting on board. M.C. label style I.

####### Dimensions.

Not given; figured specimen herein H 24.4, D 13.7, W 6.2.

####### Type material.

NHMUK 20100515, one syntype (Cuming coll.).

####### Remarks.

The specimen corresponds to Reeve’s figure, except that the spire is less pointed; therefore, considered a possible syntype. No other material is present in the collection that can be matched to Reeve’s figure or can be attributed to Dyson.

####### Current systematic position.

Bulimulidae, *Bulimulus cacticolus* (Reeve, 1849).

###### Helix cactorum

d’Orbigny, 1835

http://species-id.net/wiki/Helix_cactorum

Figs 6D , L10iv

Helix cactorum d’Orbigny 1835: 10.
Bulimus hennahi Gray; d’Orbigny 1837 [1834–1847]: 283, pl. 30 figs 3–4 [3 April 1837; text 6 May 1838].

####### Type locality.

”provincia Tacnacensi (rep. Peruviana)”.

####### Label.

”Tacna, Pérou”, in d’Orbigny’s handwriting.

####### Dimensions.

”Longit. 25 millim., latit. 15 millim.”; figured specimen herein H 24.4, D 12.8, W 7.0.

####### Type material.

NHMUK 1854.12.4.189, lectotype and six paralectotypes (d’Orbigny coll.).

####### Remarks.

Seven specimens are present in the d’Orbigny collection, of which one corresponds to d’Orbigny 1837 [1834–1847]: pl. 30 fig. 4; it is here designated lectotype (**design. n.**). This taxon has been synonymized with *Bostryx hennahi* (J.E. Gray, 1830) by Richardson (1995: 27); the latter species was described from ”Arica”, which is presumably meant to be in (nowadays) Chile, while Tacna is in southern Peru; both localities are in the coastal area.

####### Current systematic position.

Bulimulidae, *Bostryx hennahi* (J.E. Gray, 1830).

###### Bulimus californicus

Reeve, 1848

http://species-id.net/wiki/Bulimus_californicus

Figs 23C , L10v

Bulimus californicus Reeve 1848 [1848–1850]: pl. 56 fig. 378.

####### Type locality.

”California”.

####### Label.

”California”. M.C. label style III.

####### Dimensions.

Not given; figured specimen herein H 25.9, D 11.7, W. 6.3.

####### Type material.

NHMUK 1975136, three syntypes (Cuming coll.).

####### Remarks.

This taxon is considered as a junior subjective synonym of *Drymaeus (Mesembrinus) ziegleri* (Pfeiffer, 1847) by Richardson (1995: 198).

####### Current systematic position.

Bulimulidae, *Drymaeus (Mesembrinus) ziegleri* (Pfeiffer, 1847).

###### Bulimus caliginosus

Reeve, 1849

http://species-id.net/wiki/Bulimus_caliginosus

Figs 69C–D , 69K , L10vi

Bulimus caliginosus Reeve 1849 [1848–1850]: pl. 82 fig. 609.

####### Type locality.

”—?”.

####### Label.

”?”. M.C. label style I.

####### Dimensions.

Not given; figured specimen herein H 35.4, D 17.9, W 6.1.

####### Type material.

NHMUK 20100518.1–3, lectotype and two paralectotypes (Cuming coll.).

####### Remarks.

This taxon was classified as a *Naesiotus* species by Breure (1979). The sculpture of the protoconch is, however, not straightly axially ribbed but with a waving sculpture that is typical for species classified with *Vermiculatus* Breure, 1978. The specimen figured by Reeve is here designated lectotype (**design. n.**) to define the taxon.

####### Current systematic position.

Bulimulidae, *Kuschelenia (Vermiculatus) caliginosus* (Reeve, 1849) (**comb. n.**).

###### Bulimus canaliculatus

Pfeiffer, 1845

http://species-id.net/wiki/Bulimus_canaliculatus

Figs 38G–I , L11i

Bulimus canaliculatus Pfeiffer 1845a: 68; Reeve 1848 [1848–1850]: pl. 41 fig. 256.
Drymaeus (Drymaeus) canaliculatus ; Breure 1979: 107 (lectotype designation); Breure and Eskens 1981: 12, pl. 5 fig. 6.

####### Type locality.

”Bolivia”.

####### Label.

”Bolivia”; taxon label in Pfeiffer’s handwriting. M.C. label style I.

####### Dimensions.

”Long. 37, diam. 14 mill.”; figured specimen herein H 36.0, D 16.2, W 6.6.

####### Type material.

NHMUK 1975514, lectotype; 1975515, one paralectotype (Cuming coll.).

####### Remarks.

The lectotype has also been figured by Reeve (1848 [1848–1850]). The current systematic position follows Richardson (1995: 107).

####### Current systematic position.

Bulimulidae, *Drymaeus (Drymaeus) canaliculatus* (Pfeiffer, 1845).

###### Drymaeus prestoni cancellata

da Costa, 1906

http://species-id.net/wiki/Drymaeus_prestoni_cancellata

Figs 24K , L11ii

Drymaeus prestoni cancellata da Costa 1906a: 9, pl. 1 fig. 10.
Drymaeus (Mesembrinus) prestoni cancellata ; Breure 1979: 117.
Drymaeus (Mesembrinus) tripictus hoffmanni (Martens, 1893); Thompson 2011: 117.

####### Type locality.

”Chiriqui, Panama”.

####### Label.

”Chiriqui”, in da Costa’s handwriting.

####### Dimensions.

Not given; figured specimen herein H 18.1, D 9.8, W 5.3.

####### Type material.

NHMUK 1907.11.21.13, holotype (da Costa coll.).

####### Remarks.

As da Costa speaks about ”this pretty shell”, it is implied that he had only one specimen at hand; thus the specimen found should be regarded holotype. The current systematic position follows the synonymisation by Thompson (2011).

####### Current systematic position.

Bulimulidae, *Drymaeus (Mesembrinus) tripictus hoffmanni* (Martens, 1893).

###### Drymaeus castaneostrigatus

da Costa, 1906

http://species-id.net/wiki/Drymaeus_castaneostrigatus

Figs 36G–I , L11iii

Drymaeus castaneostrigatus da Costa 1906b: 98, pl. 11 fig. 5; Breure 1979: 107.

####### Type locality.

”Pozuzo, Eastern Peru”.

####### Label.

”Pozuzo. E. Peru”, in da Costa’s handwriting.

####### Dimensions.

”Long. 19, diam. 8.5 mm”; figured specimen herein H 20.4, D 8.6, W 6.3.

####### Type material.

NHMUK 1907.11.21.19; holotype (da Costa coll.).

####### Remarks.

da Costa referred to ”This shell (..)”, which might imply that he had only seen one specimen. The NHMUK material is herein regarded the holotype.

####### Current systematic position.

Bulimulidae, *Drymaeus (Drymaeus) castaneostrigatus* da Costa, 1906.

###### Bulimus castus

Pfeiffer, 1847

http://species-id.net/wiki/Bulimus_castus

Figs 19D , L11iv

Bulimus castus Pfeiffer 1847: 112; Reeve 1848 [1848–1850]: pl. 40 figs 1–2.
Drymaeus (Mesembrinus) castus ; Breure 1979: 117 (lectotype designation).

####### Type locality.

”Central America ?”.

####### Label.

”Central America?”. M.C. label style III.

####### Dimensions.

”Long. 19, diam. 9 mill.”; figured specimen herein H 19.0, D 9.4, W 5.1.

####### Type material.

NHMUK 1975197, lectotype; 1975198, two paralectotypes (Cuming coll.).

####### Remarks.

The specimen figured by Reeve (1848 [1848–1850]) was designated lectotype by Breure (1979). The current systematic position follows Thompson (2011: 109).

####### Current systematic position.

Bulimulidae, *Drymaeus (Mesembrinus) castus castus* (Pfeiffer, 1847).

###### Bulimus catlowiae

Pfeiffer, 1853

http://species-id.net/wiki/Bulimus_catlowiae

Figs 15C , L11v

Bulimus catlowiae Pfeiffer 1853d: 427; Breure and Coppois 1978: 182 (lectotype designation); Breure 1979: 68.

####### Type locality.

[Ecuador] ”prope Quito (*Bourcier*)”.

####### Label.

”near Quito / Mons^r^ Bourcier / Consul General”; taxon label in Pfeiffer’s handwriting. M.C. label style IV.

####### Dimensions.

”Long. 25, diam. 11 mill.”; figured specimen herein H 25.1, D 12.8, W 6.7.

####### Type material.

NHMUK 1975414, lectotype; 1975415, two paralectotypes (Cuming coll.).

####### Remarks.

This taxon is considered a junior subjective synonym of *Naesiotus quitensis* (Pfeiffer, 1848) (Richardson 1995: 229).

####### Current systematic position.

Bulimulidae, *Naesiotus quitensis* (Pfeiffer, 1848).

###### Bulimulus (Drymaeus) caucaensis

da Costa, 1898

http://species-id.net/wiki/Bulimulus_caucaensis

Figs 31G–I , L11vi

Bulimulus (Drymaeus) caucaensis da Costa 1898: 81, pl. 6 fig. 3.
Drymaeus (Drymaeus) caucaensis ; Pilsbry 1898 [1897–1898]: 247, pl. 50 fig. 83; Linares and Vera 2012: 183.
Drymaeus (Drymaeus) caucaensis ; Breure 1979: 107.

####### Type locality.

”Valley of the R. Cauca, Colombia”.

####### Label.

”Cauca, Colombia”, in da Costa’s handwriting.

####### Dimensions.

”Long. 35, diam. 16 mm.”; figured specimen herein H 34.8, D 16.6, W 6.3.

####### Type material.

NHMUK 1907.11.21.43, lectotype (da Costa coll.).

####### Remarks.

The specimen is labelled ”type”; however, since da Costa did not state on how many specimens his description was based upon, the reference of Breure (1979) to ”HT 1907.11.21.43” has to be interpreted as lectotype designation according to Art. 74.6 ICZN.

####### Current systematic position.

Bulimulidae, *Drymaeus (Drymaeus) caucaensis* (da Costa, 1898).

###### Bulimus ceratacme

Pfeiffer, 1855

http://species-id.net/wiki/Bulimus_ceratacme

Figs 3F–G , L11vii

Bulimus ceratacme Pfeiffer 1855c: 8; Pfeiffer 1859b: 424.
Bostryx ceratacme ; Breure 1978: 57 (lectotype designation); Breure 1979: 52.

####### Type locality.

”Peru?”.

####### Label.

”Peru”; taxon label in Pfeiffer’s handwriting. M.C. label style IV.

####### Dimensions.

”Long. 18 1/2, diam. 6 mill.”; figured specimen herein H 18.0, D 6.11, W 7.5.

####### Type material.

NHMUK 1975374, lectotype (Cuming coll.).

####### Remarks.

The current systematic position follows Richardson (1995: 19).

####### Current systematic position.

Bulimulidae, *Bostryx ceratacme* (Pfeiffer, 1855).

###### Bulimus cercicola

Morelet, 1863

http://species-id.net/wiki/Bulimus_cercicola

Figs 4G , L12i

Bulimus cercicola Morelet 1863: 192, pl. 9 fig. 7.
Bulimulus (Lissoacme) cercicola ; Pilsbry 1896 [1895–1896]: 184, pl. 46 fig. 63.
Bostryx cereicola [sic]; Breure 1978: 56; Breure 1979: 52.

####### Type locality.

[Peru] ”vallées chaudes d’Abancay et d’Acostambo, situées à l’ouest du Cuzco”.

####### Label.

”Abancay, Pérou”, in Morelet’s handwriting.

####### Dimensions.

”Long. 20; diam. 9 mill.”; figured specimen herein H 18.7, D 8.75, W 5+.

####### Type material.

NHMUK 1893.2.4.175–176, two syntypes (Morelet coll.).

####### Remarks.

One of the syntypes is severely damaged; the other has the top damaged. The current systematic position as given by Richardson (1995: 36).

####### Current systematic position.

Bulimulidae, *Bostryx orophilus* (Morelet, 1860).

###### Bulimus chamaeleon

Pfeiffer, 1855

http://species-id.net/wiki/Bulimus_chamaeleon

Figs 40A–C , L12ii

Bulimus chamaeleon Pfeiffer 1855f: 116; Breure 1979: 107.

####### Type locality.

[Ecuador] ”Quito (*Mr. Bourcier*)”.

####### Label.

”near Quito / Ecuador / Monsr Bourcier / Consul General”; taxon label in Pfeiffer’s handwriting. M.C. label style IV.

####### Dimensions.

”Long 27, diam. 11 1/2 mill.”; figured specimen herein H 27.6, D 12.2, W 6.2.

####### Type material.

NHMUK 1975448, three syntypes (Cuming coll.).

####### Remarks.

Richardson (1995: 98) listed this taxon as a nomen nudum in Pfeiffer 1853d: 422, and Pfeiffer 1854 in Küster and Pfeiffer 1840–1865: 105, pl. 33 figs 17–18. However, the name *Bulimus chamaeleon* seems to have been published for the first time in Pfeiffer 1855f: 116. The current systematic position follows Richardson (1995).

####### Current systematic position.

Bulimulidae, *Drymaeus (Drymaeus) ambustus* (Reeve, 1849).

###### Naesiotus (Naesiotus) subcostatus chamayensis

Weyrauch, 1967

http://species-id.net/wiki/Naesiotus_subcostatus_chamayensis

Fig. 15E

Naesiotus (Naesiotus) subcostatus chamayensis Weyrauch 1967a: 409, figs 97–99; Breure 1979: 68; Neubert and Janssen 2004: 203, pl. 10 fig. 121; Breure 2012a: 6.

####### Type locality.

”Norte interandino de Perú, cerca de pueblo Chamaya, sobre la margen izquierda del río Chamaya, afluente occidental del río Maranon, 550–660 m”.

####### Label.

”Above Chamaya, Río Chamaya, western affluent of Río Marañon, 600 m”; printed label.

####### Dimensions.

”A. 18.2, D. 7.3 [mm]”; figured specimen H 17.5 D 7.7 W 7.5.

####### Type material.

NHMUK 1975423, three paratypes (ex Weyrauch).

####### Current systematic position.

Bulimulidae, *Naesiotus subcostatus chamayensis* Weyrauch, 1967.

###### Otostomus championi

Martens, 1893

http://species-id.net/wiki/Otostomus_championi

Figs 19E–F , L12iii

Otostomus championi Martens 1893 [1890–1901]: 222, pl. 14 fig. 5.
Drymaeus (Mesembrinus) championi (von Martens); Breure 1979: 107 (lectotype designation); Breure and Eskens 1981: 14; Thompson 2011: 117.

####### Type locality.

”W. Guatemala, Hacienda de Los Nubes, Cerro Zunil, Pacific slope, in the vicinity of the coffee-plantations, at an elevation of about 5000 feet (*Champion*)”.

####### Label.

”Cerro Zunil / Champion”.

####### Dimensions.

”Long. 27, diam. 14 1/2 mill.”; figured specimen herein H 27.0, D 13.8, W 6.2.

####### Type material.

NHMUK 1901.6.22.781, lectotype (Godman coll.).

####### Remarks.

The specimen is not accompanied by a taxon label in von Marten’s handwriting, but bears the name of the collector and corresponds to the original figure. The current systematic position follows Thompson (2011).

####### Current systematic position.

Bulimulidae, *Drymaeus (Mesembrinus) championi* (Martens, 1893).

###### Bulimus chemnitzioides

Forbes, 1850

http://species-id.net/wiki/Bulimus_chemnitzioides

Figs 12I , L12iv

Bulimus chemnitzioides Forbes 1850: 55, pl. 9 figs 6a–b; Pfeiffer 1854 in Küster and Pfeiffer 1840–1865: 94, pl. 31 figs 21–23.
Bulimulus chemnitzioides ; Pilsbry 1897 [1897–1898]: 124, pl. 24 figs 45–46.

####### Type locality.

[Ecuador] ”Chatham Island, Gelepagos [sic, Galápagos]”.

####### Label.

”Chatham Island / Galapagos / Proc. Zool. Soc. / fig. 6”.

####### Dimensions.

”Long. 19, diam. 4 mil..”; figured specimen herein H 18.5, D 3.64, W 16.0.

####### Type material.

NHMUK 1855.4.5.23, lectotype; 1850.4.23.14–15, two paralectotypes (ex Kellett and Wood).

####### Remarks.

The specimen originally figured is here designated lectotype (**design. n.**). The two other specimens also originate from the voyage of Kellett and Wood (Forbes 1850, Seemann 1853), and are considered to be part of the original series.

####### Current systematic position.

Bulimulidae, *Naesiotus chemnitzioides* (Forbes, 1850).

###### Scutalus (Scutalus) chiletensis

Weyrauch, 1967

http://species-id.net/wiki/Scutalus_chiletensis

Fig. 65G

Scutalus (Scutalus) chiletensis Weyrauch 1967a: 373, figs 24–30; Breure 1979: 83; Neubert and Janssen 2004: 204, pl. 12 fig. 138; Breure 2012a: 6, pl. 5 figs 46–47.

####### Type locality.

”Norte de Péru, vertiente occidental de la Cordillera Occidental, cerro encima del pueblo Chilete, sobre la margen izquierda del río Jequetepeque, 850–950 m”.

####### Label.

”N-Peru, mountain above Chilete, Rio Jequetepeque, 850-950m”; printed label.

####### Dimensions.

”A. 33.1, D. 21 [mm]”; figured specimen herein H 34.2 D 23.4 W 6.0.

####### Type material.

NHMUK 1975384, five paratypes (ex Weyrauch).

####### Current systematic position.

Bulimulidae, *Scutalus chiletensis* Weyrauch, 1967.

###### Bulimus chimborasensis

Reeve, 1848

http://species-id.net/wiki/Bulimus_chimborasensis

Figs 38A–C , L12v

Bulimus chimborasensis Reeve 1848 [1848–1850]: pl. 44 fig. 275.
Drymaeus chimborasensis ; Pilsbry 1898 [1897–1898]: 261, pl. 40 fig. 1; Linares and Vera 2012: 183.
Drymaeus (Drymaeus) chimborasensis ; Breure 1979: 107; Breure and Borrero 2008: 20.

####### Type locality.

”Chimborazo, Columbia, New Granada”.

####### Label.

”Chimborazo”. M.C. label style IV, V.

####### Dimensions.

Not given; figured specimen herein H 39.7, D 15.0, W 7.0.

####### Type material.

NHMUK 1975460, three syntypes (Cuming coll.).

####### Remarks.

Reeve described this species from ”Mus. Dennison”, and said it was identical to *Bulimus decoratus* Lea, 1838 (not Férussac, 1821); he referred to Lea’s figure (pl. 23 fig. 8). The specimens found correspond to Reeve’s description, but none can be matched to the shell figured on his plate 44.

####### Current systematic position.

Bulimulidae, *Drymaeus (Drymaeus) chimborasensis* (Reeve, 1848).

###### Drymaeus chiriquensis

da Costa, 1901

http://species-id.net/wiki/Drymaeus_chiriquensis

Figs 42J–L , L12vi

Drymaeus chiriquiensis da Costa 1901: 238, pl. 24 fig. 1; Pilsbry 1901 [1901–1902]: 162, pl. 48 fig. 47.
Drymaeus (Drymaeus) chiriquiensis ; Breure 1979: 107.
Drymaeus (Mesembrinus) chiriquiensis Thompson 2011: 114.

####### Type locality.

”Boqueti, Chiriquí, Panama”.

####### Label.

”Boqueti / Chiriqui / Panama”, in da Costa’s handwriting.

####### Dimensions.

”Long. 29, diam. 14 mm.”; figured specimen herein H 29.1, D 13.6, W 5.5.

####### Type material.

NHMUK 1907.11.21.119, holotype (da Costa coll.).

####### Remarks.

As da Costa (1901) writes ”A single specimen has been received of this pretty shell”, it is clear that the specimen has to be regarded as the holotype. The current systematic position follows Thompson (2011).

####### Current systematic position.

Bulimulidae, *Drymaeus (Mesembrinus) chiriquensis* da Costa, 1901.

###### Bulimus cinereus

Reeve, 1849

http://species-id.net/wiki/Bulimus_cinereus

Figs 15F–G , L12vii

Bulimus cinereus Reeve 1849 [1848–1850]: pl. 56 fig. 372.

####### Type locality.

”Bolivia”.

####### Label.

”Bolivia”. M.C. label style I, V.

####### Dimensions.

Not given; figured specimen herein H 15.5, D 6.8, W 7.5.

####### Type material.

NHMUK 20100519, lectotype (Cuming coll.).

####### Remarks.

The specimen figured by Reeve 1849 [1848–1850] was found and is now designated lectotype (**design. n.**). This hitherto unfigured taxon was regarded a nomen inquirendum by Breure (1979: 136) and Richardson (1995: 387). The protoconch is sculptured with axial riblets, typical of *Naesiotus* sensu Breure 1979.

####### Current systematic position.

Bulimulidae, *Naesiotus cinereus* (Reeve, 1849) (**comb. n.**).

###### Bulimus citronellus

Angas, 1879

http://species-id.net/wiki/Bulimus_citronellus

Figs 20E , L13i

Bulimus citronellus Angas 1879: 479, pl. 40 fig. 5; Thompson 2011: 119.
Drymaeus (Mesembrinus) citronellus ; Breure 1979: 117 (lectotype designation).

####### Type locality.

”Uren to Lipurio, Costa Rica (*Gabb*)”.

####### Label.

”Uren to Lipurio / low hills / Gabb / Costa rica”, in Angas’s handwriting.

####### Dimensions.

”Diam. 6, alt. 12 lin. [H 25.0, D 12.5 mm]”; figured specimen herein H 25.0, D 11.9, W 6.9.

Type material. NHMUK 1879.7.22.19–22, lectotype and three paralectotypes (ex Angas).

####### Remarks.

Angas (1879) writes that he has seen ”only two specimens”. The lot is marked ”type” and has been registered as having four specimens (1879.7.22.19-22). The current systematic position follows Richardson (1995: 183).

####### Current systematic position.

Bulimulidae, *Drymaeus (Mesembrinus) sulphureus* (Pfeiffer, 1857).

###### Bulimus clarus

Pfeiffer, 1857

http://species-id.net/wiki/Bulimus_clarus

Figs 73I–J , L13ii

Bulimus clarus Pfeiffer 1857c: 330.

####### Type locality.

”Meobamba, Peru (*Mr. Gueinzius*)”.

####### Label.

”Ecuador” [see remarks]; taxon label in Pfeiffer’s handwriting. M.C. label style I.

####### Dimensions.

”Long. 16, diam. 7 2/3 mill.”. See remarks.

####### Type material.

NHMUK 1975487, two possible syntypes [see remarks] (Cuming coll.).

####### Remarks.

The type material consists of a mixed lot, one specimen belonging to *Naesiotus* sensu Breure 1979 (H 15.9, D 8.2, W 7.0), the other to *Drymaeus* (H 15.8, D 7.6, W 5.4). The latter corresponds more closely to the original measurements, but is subadult. However, as the locality differs from the type locality, and the collector is not mentioned on the label, there remains doubt whether this is the original lot. The taxon is now considered a nomen inquirendum.

####### Current systematic position.

Bulimulidae.

###### Bulimus clathratus

Pfeiffer, 1858

http://species-id.net/wiki/Bulimus_clathratus

Figs 43D–F , L13iii

Bulimus clathratus Pfeiffer 1858a: 258; Pfeiffer 1859a: 47.
Drymaeus (Drymaeus) clathratus ; Breure 1979: 107 (lectotype designation); Breure and Eskens 1981: 14.

####### Type locality.

”Province of Patas, Andes of Peru (*Dr. Farris*)”.

####### Label.

”Province of Patas, / Andes of Peru / D^r^ Farris”; taxon label in Pfeiffer’s handwriting.

####### Dimensions.

”Long. 30, diam. 11 mill.”; figured specimen herein H 30, D 12.2, W 7.8.

####### Type material.

NHMUK 1975449, lectotype (Cuming coll.).

####### Remarks.

This hitherto unfigured taxon is known from a single specimen. The current systematic position follows Richardson (1995: 110).

####### Current systematic position.

Bulimulidae, *Drymaeus (Drymaeus) clathratus* (Pfeiffer, 1858).

###### Bulimus coagulatus

Reeve, 1849

http://species-id.net/wiki/Bulimus_coagulatus

Figs 66E , L13iv

Bulimus coagulatus Reeve 1849 [1848–1850]: pl. 77 fig. 558.
Scutalus (Vermiculatus) coagulatus (Reeve); Breure 1978: 173 (lectotype designation); Breure 1979: 85.

####### Type locality.

”Peru”.

####### Label.

”Peru”. M.C. label style IV, V.

####### Dimensions.

Not given; figured specimen herein H 15.6, D 9.3, W 5.1.

####### Type material.

NHMUK 1975351, lectotype; 1975352 two paralectotypes (Cuming coll.).

####### Remarks.

This is a mixted lot of four specimens. The lectotype is an inmature specimen and is considered by Ramírez and Thomé (2005) to be synonymous with *Scutalus versicolor* (Broderip in Broderip and Sowerby 1832). They identified one of the other specimens as *Bostryx conspersus* (NHMUK 1975352); this specimen is no longer considered as type material.

####### Current systematic position.

Bulimulidae, *Scutalus versicolor* (Broderip in Broderip and Sowerby 1832).

###### Bulimus coarctatus

Pfeiffer, 1845

http://species-id.net/wiki/Bulimus_coarctatus

Figs 37F–H , L13v

Bulimus coarctatus Pfeiffer 1845b: 73; Pfeiffer 1848b: 90; Pfeiffer 1856 [1854–1860]: 80, pl. 22 figs 22–23; Breure 1979: 108 (lectotype designation).
Drymaeus coarctatus ; Pilsbry 1898 [1897–1898]: 195, pl. 28 figs 17–18; Simone 2006: 136, fig. 445.
Drymaeus (Drymaeus) coarctatus ; Breure and Eskens 1981: 16, pl. 5 fig. 5.

####### Type locality.

”Locality unknown”.

####### Label.

”Brazil” (added in later handwriting); taxon label in Pfeiffer’s handwriting. M.C. label style I.

####### Dimensions.

”Long. 34, diam. 17 mill.”; figured specimen herein 34.6, D 20.2, W 6.9.

####### Type material.

NHMUK 1975560, lectotype; 1975561, one paralectotype (Cuming coll.).

####### Remarks.

The specimen designated lectotype has the best match with the original measurements. The other specimen has been figured in Pfeiffer 1856 [1854–1860]: pl. 22 figs 22–23.

####### Current systematic position.

Bulimulidae, *Drymaeus (Drymaeus) coarctatus* (Pfeiffer, 1845).

###### Bulimus columbiensis

Pfeiffer, 1855

http://species-id.net/wiki/Bulimus_columbiensis

Figs 19I–J , L25v

Bulimus columbiensis Pfeiffer 1855g: 124; Breure 1979: 118 (lectotype designation).
Bulimus gratus Pfeiffer 1856a: 159. New name for *Bulimus columbiensis* Pfeiffer non Lea.
Drymaeus (Mesembrinus) gratus ; Breure and Eskens 1981: 71, pl. 7 fig. 9.
Drymaeus columbiensis ; Linares and Vera 2012: 193.

####### Type locality.

”Columbia”.

####### Label.

”Columbia”; ”Venezuela” in a later handwriting. Labels for both taxon names in Pfeiffer’s handwriting. M.C. label style I.

####### Dimensions.

”Long. 28, diam. 11 1/2 mill.”; figured specimen herein H 28.0, D 11.9, W 7.2.

####### Type material.

NHMUK 1975521, lectotype (Cuming coll.).

####### Remarks.

The original taxon label has been corrected to *Bulimus gratus*, in a different ink. However, also a label with this name in Pfeiffer’s handwriting has been glued to the board. It may be noted that Lea’s name (*Bulimus columbianus*) is sufficiently different to retain Pfeiffer’s original taxon name. On the front side, a later hand has written ”Venezuela”, which may be seen as a further precision to reflect the later political-administrative situation. Breure and Borrero (unpublished data) could not recognise this species in the Colombian material they studied.

####### Current systematic position.

Bulimulidae, *Drymaeus (Mesembrinus) columbiensis* (Pfeiffer, 1855).

###### Bulimulus compactus

Fulton, 1902

http://species-id.net/wiki/Bulimulus_compactus

Figs 7H , L13vi

Bulimulus compactus Fulton 1902: 69.
Bostryx compactus ; Breure 1979: 52.

####### Type locality.

”Chicani, Bolivia”.

####### Label.

”Chicani Bolivia”, in Fulton’s handwriting.

####### Dimensions.

”Alt. 16, maj. diam. 9 millim.”; figured specimen herein H 15.8, D 7.62, W 6.0.

####### Type material.

NHMUK 1902.5.28.1, syntype (ex Fulton).

####### Remarks.

Fulton did not state on how many specimens his description was based; the specimen is therefore regarded as a syntype. The current systematic position follows Richardson (1995: 20).

####### Current systematic position.

Bulimulidae, *Bostryx compactus* (Fulton, 1902).

###### Bulimus confluens

Pfeiffer, 1855

http://species-id.net/wiki/Bulimus_confluens

Figs 31J–L , L14i

Bulimus confluens Pfeiffer 1855f: 115; Pfeiffer 1859b: 443; Breure 1979: 108 (lectotype designation).
Drymaeus confluens ; Linares and Vera 2012: 183.
Drymaeus (Drymaeus) confluens ; Breure and Eskens 1981: 16, pl. 8 fig. 1.

####### Type locality.

[Colombia] ”Marmato, New Granada”.

####### Label.

”Venezuela” [see remarks]; taxon label in Pfeiffer’s handwriting. M.C. label style IV.

####### Dimensions.

”Long. 40, diam. 17 mill.”; figured specimen herein H 39.5, D 20.8, W 6.3.

####### Type material.

NHMUK 1975196, lectotype and one paralectotype (Cuming coll.).

####### Remarks.

According to Pfeiffer (1859: 443) the type is in ”Mus. Cuming”. The original specimen appears to be lost. The specimens are labelled ”Bulimus con-/fluens Pfr. var. / Venezuela”. There is no variety mentioned by Pfeiffer, nor in his original paper nor in subsequent publications, which is interpreted as him considering this a variable taxon; the specimens are thus regarded to be from the type series.

####### Current systematic position.

Bulimulidae, *Drymaeus (Drymaeus) confluens* (Pfeiffer, 1855).

###### Bulimus confusus

Reeve, 1848

http://species-id.net/wiki/Bulimus_confusus

Figs 66I–K , L14ii

Bulimus confusus Reeve 1848 [1848–1850]: pl. 48 fig. 316; Pfeiffer 1853d: 426; Breure 1979: 85.
Bulimulus confusus ; Pilsbry 1898 [1897–1898]: 282, pl. 45 fig. 32.
Scutalus (Vermiculatus) confusus ; Breure 1978: 173 (lectotype designation).

####### Type locality.

Not given.

####### Label.

”Peru”, corrected to ”?Peru” on a label in E.A. Smith’s handwriting. M.C. label style III, V.

####### Dimensions.

Not given; figured specimen herein H 29.0, D 13.1, W 6.0.

####### Type material.

NHMUK 1975194, lectotype; 1975195, two paralectotypes (Cuming coll.).

####### Remarks.

The protoconch sculpture is typically for *Kuschelenia* Hylton Scott, 1952. The placement of this taxon within *Drymaeus* by Richardson (1995) is thus erroneous. Breure (1978) mentioned ”New Grenada” as locality on the label; however, no mentioning of Peru which may be the original locality, if Crawford’s interpretation (Crawford 1939: 328) was correct to synonymize this taxon with *Kuschelenia culminea* (d’Orbigny, 1835).

####### Current systematic position.

Bulimulidae, *Kuschelenia (Kuschelenia) culminea* (d’Orbigny, 1835) (**comb. n.**).

###### Drymaeus conicus

da Costa, 1907

http://species-id.net/wiki/Drymaeus_conicus

Figs 25E , L14iii

Drymaeus conicus da Costa 1907: 305, pl. 26 figs 7–7a; Breure 1979: 108 (lectotype designation).

####### Type locality.

”Oaxarca [sic], Mexico”.

####### Label.

”Oaxarca, Mexico”, in da Costa’s handwriting.

####### Dimensions.

”Long. 17.5, diam. 7 mm.”; lectotype H 17.5, D 7.5, W 6.9.

####### Type material.

NHMUK 1907.11.21.32, lectotype and one paralectotype (da Costa coll.).

####### Remarks.

da Costa did not state on how many specimens his description was based. A lectotype was designated by Breure (1979); the paralectotype is slightly damaged at the palatal lip. The current systematic position follows Richardson (1995: 111), but the taxon is now placed in the subgenus *Mesembrinus* Albers, 1850 (**comb. n.**).

####### Current systematic position.

Bulimulidae, *Drymaeus (Mesembrinus) conicus* da Costa, 1907.

###### Bulinus conspersus

Sowerby I, 1833

http://species-id.net/wiki/Bulinus_conspersus

Figs 6C , L15i

Bulinus conspersus Sowerby I 1833b: 73.
Bulimus conspersus ; Reeve 1848 [1848–1850]: pl. 22 fig. 137.

####### Type locality.

[Peru] ”collinis prope Lima” (see remarks).

####### Label.

”Peru”. M.C. label style I, V.

####### Dimensions.

”Long. 0.65, lat. 0.4 poll. [H 16.5, D 10.1 mm]”; figured specimen herein H 19.8, D 12.1, W 5.5.

####### Type material.

NHMUK 20100619, five probable syntypes (Cuming coll.).

####### Remarks.

The original material of Sowerby has not been traced; however, one of the specimens from this lot corresponds to Reeve (1848 [1848-1850]) and the specimens are regarded probable syntypes. Ramírez (2004) has restricted the type locality to Lomas de Atocongo, near Lima.

####### Current systematic position.

Bulimulidae, *Bostryx conspersus* (Sowerby I, 1833).

###### Bulimus convexus

Pfeiffer, 1855

http://species-id.net/wiki/Bulimus_convexus

Figs 39A–C , L14iv

Bulimus convexus Pfeiffer 1855f: 116; Pfeiffer 1859b: 444; Breure 1979: 108 (lectotype designation).
Drymaeus (Drymaeus) convexus ; Breure and Eskens 1981: 16. pl. 8 fig. 7.

####### Type locality.

”New Granada”.

####### Label.

”Ecuador”, added in a later handwriting; taxon label in Pfeiffer’s handwriting. M.C. label style I.

####### Dimensions.

”Long. 38, diam. 14 mill.”; figured specimen herein H 38.5, D 16.2, W 7.2.

####### Type material.

NHMUK 1975192, lectotype; 1975193, two paralectotypes (Cuming coll.).

####### Current systematic position.

Bulimulidae, *Drymaeus (Drymaeus) convexus* (Pfeiffer, 1855).

###### Helix cora

d’Orbigny, 1835

http://species-id.net/wiki/Helix_cora

Figs 58A–B , L14v

Helix cora d’Orbigny 1835: 15.
Bulimus cora d’Orbigny 1837 [1834–1847]: 307, pl. 34 figs 14–15 [3 April 1837; text 6 May 1838].

####### Type locality.

[Peru] ”republica Peruviana”.

####### Label.

”Perou”, in d’Orbigny’s handwriting.

####### Dimensions.

”Latit. 44 millim.; longit. 29 millim.”; figured specimen herein H 43.9, D 28.0, W 6.7.

####### Type material.

NHMUK 1854.12.4.124, lectotype and two paralectotypes (d’Orbigny coll.).

####### Remarks.

d’Orbigny 1838 [1834–1847] specifies the type locality as ”sur les montagnes des environs de Trujillo”, which is in Dept. La Libertad. The lot contains two juvenile, and one adult specimen; the latter corresponds to d’Orbigny’s pl. 34 fig. 15 and is here designated lectotype (**design. n.**). The arrangement by Richardson (1995: 356), referring d’Orbigny 1837–1838 to *Bulinus proteus* Broderip in Broderip and Sowerby I 1832, may be in error.

####### Current systematic position.

Bulimulidae, *Neopetraeus cora* (d’Orbigny, 1835).

###### Bulimus coriaceus

Pfeiffer, 1857

http://species-id.net/wiki/Bulimus_coriaceus

Fig. 60K–L , L67ii

Bulimus coriaceus Pfeiffer 1857b: 318.

####### Type locality.

[Mexico] ”Cordova”.

####### Label.

”Mexico”, taxon label in Pfeiffer’s handwriting. M.C. label style I.

####### Dimensions.

”Long. 18, diam. 9 mill.”. Figured specimen H 17.1, D 9.5, W 5.8.

####### Type material.

NHMUK 1975409, three possible syntypes (Cuming coll.).

####### Remarks.

The type material was based on material collected by Sallé; however, the material is accompanied by a taxon label in Pfeiffer’s handwriting and is herein considered as possible syntypes. Pfeiffer did not state on how many specimens his description was based. The type material was up till now unfigured. The current systematic position follows Thompson (2011: 106).

####### Current systematic position.

Bulimulidae, *Bulimulus coriaceus* (Pfeiffer, 1857).

###### Bostryx (Bostryx) pygmaeus costatus

Weyrauch, 1960

http://species-id.net/wiki/Bostryx_pygmaeus_costatus

Fig. 1I

Bostryx (Bostryx) pygmaeus costatus Weyrauch 1960b: 122, pl. 11 figs 12–16; Breure 1979: 53; Neubert and Janssen 2004: 206, pl. 4 fig. 47; Breure 2012a: 7.

####### Type locality.

[Peru] ”Bei Alis, auf der rechten Seite des Río Alis, Zufluss des Río Cañete, 3300 m”.

####### Label.

”C-Peru, Alis, Río Alis, affluent of Río Cañete, 3300 m”; printed label.

####### Dimensions.

”H. 8.7 D. 5.1”; figured specimen herein H 8.45, D 4.9, W 5.2.

####### Type material.

NHMUK 1975357, one paratype (ex Weyrauch, WW 318).

####### Current systematic position.

Bulimulidae, *Bostryx pygmaeus* Weyrauch, 1960.

###### Bostryx (Elatibostryx) imeldae costifer

Weyrauch, 1960

http://species-id.net/wiki/Bostryx_imeldae_costifer

Fig. 1J

Bostryx (Elatibostryx) imeldae costifer Weyrauch 1960b: 129, pl. 12 figs 35–38; Breure 1979: 53; Neubert and Janssen 2004: 206, pl. 7 fig. 77; Breure 2012a: 7.

####### Type locality.

[Peru, Dept. Lima] ”Mittel-Peru, Quichao, 5 km von Laraos, am Fusspad nach Yauyos, auf der linken Seite des Río Mayo im Tal des Río Cañete, 3500 m”.

####### Label.

”C-Peru, Quichao, 5 kms from Laraos, valley of Río Cañete, 3500 m”; printed label.

####### Dimensions.

”H. 11.0 D. 5.0 [mm]”; figured specimen herein H 9.6, D 4.2, W 6.2.

####### Type material.

NHMUK 1975354, one paratype, ex Weyrauch (WW 3319).

####### Current systematic position.

Bulimulidae, *Bostryx imeldae* Weyrauch, 1958.

###### Bulimus cotopaxiensis

Pfeiffer, 1853

http://species-id.net/wiki/Bulimus_cotopaxiensis

Figs 69G–H , 69J , L15ii

Bulimus cotopaxiensis Pfeiffer 1853d: 419; Pfeiffer 1854c: 155; Pfeiffer 1854 in Küster and Pfeiffer 1840–1865: 103, pl. 33 figs 9–10; Breure 1979: 86; Breure and Borrero 2008: 18.
Bulimulus cotopaxiensis ; Pilsbry 1897 [1897–1898]: 31, pl. 4 figs 50–51.
Scutalus (Vermiculatus) cotopaxiensis ; Breure 1978: 175, pl. 9 fig. 9 (lectotype designation).

####### Type locality.

[Ecuador] ”reipublicae Aequatoris, montem Cotopaxi”.

####### Label.

”Cotopaxi, Equador / Monsr Bourcier / Consul General”, taxon label in Pfeiffer’s handwriting. M.C. label style IV.

####### Dimensions.

”Long. 34, diam. 16 mill.”; figured specimen herein H 33.9, D 17.1, W 6.2.

####### Type material.

NHMUK 1975370, lectotype; 1975371, two paralectotypes (Cuming coll.).

####### Remarks.

The original paper only quotes material collected by Bourcier as a variety from Cayembe. However, if the type was in the Pfeiffer collection it must now considered to be lost (Dance 1966). Both the locality, and measurements agree with Pfeiffer’s paper; the taxon label is from his hand, so there is no doubt that these specimens can be considered as type material. The shell height of the lectotype as quoted by Breure (1978) is in error. This species was listed as synonym under *Scutalus anthisanensis* (Pfeiffer, 1853) by Richardson (1995: 346); however, the shell shape of the two taxa is different, and provisionally the two taxa are kept separate awaiting further studies.

####### Current systematic position.

Bulimulidae, *Kuschelenia (Vermiculatus) cotopaxiensis* (Pfeiffer, 1853) (**comb. n.**).

###### Helix crepundia

d’Orbigny, 1835

http://species-id.net/wiki/Helix_crepundia

Figs 15H–I , L14vi

Helix crepundia d’Orbigny 1835: 14.
Bulimus crepundia ; d’Orbigny 1837 [1834–1847]: 275, pl. 33 figs 18–19 [19 June / 7 Aug 1837; text 23 April 1838].
Bulimulus crepundia ; Pilsbry 1897 [1897–1898]: 90, pl. 11 figs 33–34.
Naesiotus crepundia ; Breure 1975b: 1146, pl. 8 fig. 5.

####### Type locality.

”provincia Chiquitensi, republica Boliviana”; see Breure 1973: 116.

####### Label.

Only the taxon name is given in d’Orbigny’s handwriting.

####### Dimensions.

”Latit. 15 millim., long. 10 millim.”, corrected to ”Longueur totale, 25 millimètres” in d’Orbigny 1838 [1834–1847]; figured specimen herein H 26.6, D 11.1, W 7.9.

####### Type material.

NHMUK 1854.12.4.173, six paralectotypes; 1854.12.4.174, one paralectotype (d’Orbigny coll.).

####### Remarks.

One specimen was marked with ‘x’ on the original board, which suggests a special selection; however, it is smaller than the type (H 23.7) and has the lip damaged. Another specimen, which shows the full lip as in d’Orbigny’s figure, is here figured. The lectotype is in MNHN (Breure 1975b). In the collation of Coan et al. (2013a) the plates as issued in ‘Livraison 25’ and ‘Livraison 26’ appear to be incorrect; plate 38 is mentioned twice, while plate 33 is missing. Since no taxonomic implications seem involved, these dates have both been cited herein as it is not clear which is the correct date for each plate.

####### Current systematic position.

Bulimulidae, *Naesiotus crepundia* (d’Orbigny, 1835).

###### Bulimus cretaceus

Pfeiffer, 1855

http://species-id.net/wiki/Bulimus_cretaceus

Figs 65C–F , L15iii

Bulimus cretaceus Pfeiffer 1855g: 123; Breure 1979: 83.
Scutalus (Scutalus) cretaceus (Pfeiffer); Breure 1978: 166, figs 277–278, pl. 8 fig. 7 (lectotype designation).

####### Type locality.

”Eastern Islands [sic, see remarks] (*Captain Keppell*)”.

####### Label.

”No locality but I / had the from Cap. Keppell / who got them in the / Eastern Islands but don’t / recollect”, taxon label in Pfeiffer’s handwriting. M.C. label style IV.

####### Dimensions.

”Long. 36, diam. 18 mill.”; figured specimen herein H 35.2, D 23.2, W 6.2.

####### Type material.

NHMUK 1975388, lectotype; 1975389, one paralectotype, ex Keppell (Cuming coll.).

####### Remarks.

Pilsbry (1902 [1901–1902]: 141) was the first to recognize that this species was from Peru. The type locality is here restricted to Dept. Cajamarca, Río Jequetepeque, near Tembladera (**restr. n.**).

####### Current systematic position.

Bulimulidae, *Scutalus cretaceus* (Pfeiffer, 1855).

###### Bulinus crichtoni

Broderip, 1836

http://species-id.net/wiki/Bulinus_crichtoni

Figs 59A–C , L15iv

Bulinus crichtoni Broderip 1836: 44.
Newboldius crichtoni ; Breure 1979: 99.

####### Type locality.

[Peru] ”ad Ambo juxta Huanuco Peruviae”.

####### Label.

”Peru, Ambo, Sir A Crichton”, added in pencil. Taxon label probably in Broderip’s handwriting.

####### Dimensions.

”Long. 3 (circiter), latit. 1 1/8 poll. [H 76.0, D 28.5 mm]”; holotype H 71.3, D 33.5, W 4+.

####### Type material.

NHMUK 1958.9.3.4, holotype (Broderip coll.).

####### Remarks.

The top whorl of the specimen is missing. Broderip refers to ”the apex of the shell under description, the only specimen I ever saw, is broken, and its actual length is 2 inches and 7/8 [72.8 mm]”; we consider this specimen the holotype.

####### Current systematic position.

Bulimulidae, *Newboldius crichtoni* (Broderip, 1836).

###### Helix culminea

d’Orbigny, 1835

http://species-id.net/wiki/Helix_culminea

Figs 66F–H , L15v

Helix culminea d’Orbigny 1835: 13; Breure 1979: 88.
Bulimus culmineus ; d’Orbigny 1837 [1834–1847]: 288, pl. 33 figs 8–9 [19 June / 7 Aug 1837; text 6 May 1838].
Bulimulus culmineus ; Pilsbry 1897 [1897–1898]: 25, pl. 5 figs 74–75.
Scutalus culmineus culmineus ; Breure 1975b: 1143, pl. 1 fig. 3.

####### Type locality.

”culminibus Andesensibus, republica Boliviana” (see remarks).

####### Label.

”Titicaca, Bolivia”, in d’Orbigny’s handwriting.

####### Dimensions.

”Latit. 17 millim., longit. 13 millim.”; figured specimen herein H 31.2, D 14.1, W 6.3.

####### Type material.

NHMUK 1854.12.4.198, six paralectotypes (d’Orbigny coll.).

####### Remarks.

d’Orbigny (1838 [1834–1847]: 289) specified the type locality as ”les montagnes de la province de Carangas, à l’ouest d’Oruro, principalement sur celle du ‘Pucara’, à cinq lieues du bourg de Totora”. Breure (1973: 130, fig. 3) located ‘Pucara’ at approximately 17°58’S, 068°21’W. This place could not be found using modern gazetteers, but Totora is located at 17°47’S, 068°08’W. The old French distance ‘lieu’ equals 4.44 kms, thus the Pucara mountain is about 22.2 km from Totora; however, d’Orbigny did not state in which direction he travelled. Breure (1975) restricted the type locality to this area, contrary to Weyrauch (1967a: 393) who selected the peninsula Capachica, in the Peruvian part of Lake Titicaca, as type locality; he referred to Crawford (1939) who had studied the types of d’Orbigny in NHMUK. However, Weyrauch may have overlooked that d’Orbigny (1838 [1834–1847]: 289) specified a second locality ”sur toutes les îles et sur toutes les montagnes du lac de Titicaca”. The specimens from the latter locality are those in the NHMUK collection, while those from ”Carangas” are in the MNHN collection (Breure 1975b); the lectotype has been selected from the latter collection, and the type locality is thus in Bolivia.

####### Current systematic position.

Bulimulidae, *Kuschelenia (Kuschelenia) culminea* (d’Orbigny, 1835).

###### Bulimulus (Naesiotus) curtus

Reibisch, 1892

http://species-id.net/wiki/Bulimulus_curtus

Figs 13G–H , L16i

Bulimulus (Naesiotus) curtus Reibisch 1892: 21, pl. 1 fig. 14.
Naesiotus curtus ; Breure 1979: 68.

####### Type locality.

[Ecuador, Galápagos, Isla San Cristóbal] ”Chatam-Island [sic] (Wolf)”.

####### Label.

”Chatham Is. Galapagos”.

####### Dimensions.

”Long. 10,25, diam. maj. 5 mm”; figured specimen herein H 9.6, D 4.62, W 6.9.

####### Type material.

NHMUK 1894.6.8.8–9, two syntypes, T. Wolf leg.

####### Remarks.

Reibisch did not state on how many specimens his description was based. The current systematic position follows Richardson (1995: 217).

####### Current systematic position.

Bulimulidae, *Naesiotus curtus* (Reibisch, 1892).

###### Bulimus cuticula

Pfeiffer, 1855

http://species-id.net/wiki/Bulimus_cuticula

Figs 42G–I , L16ii

Bulimus cuticula Pfeiffer 1855d: 95; Breure 1979: 108 (lectotype designation).
Drymaeus (Drymaeus) cuticulus ; Breure and Eskens 1981: 20; Simone 2006: 136, fig. 446.

####### Type locality.

”Rio Janeiro”.

####### Label.

”Rio”, taxon label in Pfeiffer’s handwriting. M.C. label style I.

####### Dimensions.

”Long. 28, diam. 13 mill.”; figured specimen herein H 27.5, D 13.75, W 4.7.

####### Type material.

NHMUK 1975451, lectotype (Cuming coll.).

####### Remarks.

There is only one specimen, of which the lip has been broken, and the last whorl is badly damaged. The specimen is probably subadult.

####### Current systematic position.

Bulimulidae, *Drymaeus (Drymaeus) cuticula* (Pfeiffer, 1855).

###### Bulimus cuzcoensis

Reeve, 1849

http://species-id.net/wiki/Bulimus_cuzcoensis

Figs 43A–C , L16iii

Bulimus cuzcoensis Reeve 1849 [1848–1850]: pl. 71 fig. 514; Reeve 1850: 98; Pfeiffer 1853d: 344; Breure 1979: 108 (lectotype designation).

####### Type locality.

[Peru] ”Cuzco, Bolivia [sic]”.

####### Label.

”Peru”, added in a later handwriting. M.C. label style IV, V.

####### Dimensions.

Not given; figured specimen herein H 36.6, D 15.7, W 7.2.

####### Type material.

NHMUK 1975453, lectotype; 1975454, two paralectotypes (Cuming coll.).

####### Remarks.

The current systematic position follows Richardson (1995: 112).

####### Current systematic position.

Bulimulidae, *Drymaeus (Drymaeus) cuzcoensis* (Reeve, 1849).

###### Bulimulus dacostae

Sowerby III, 1892

http://species-id.net/wiki/Bulimulus_dacostae

Figs 33M–O , L16iv

Bulimulus dacostae Sowerby III 1892: 297, pl. 23 figs 15–16.
Drymaeus dacostae ; Pilsbry 1898 [1897–1898]: 214, pl. 43 figs 77–78; Linares and Vera 2012: 184.
Drymaeus (Drymaeus) dacostae ; Breure 1979: 108.

####### Type locality.

[Colombia] ”Bogota (*Mus. Da Costa*)”.

####### Label.

”Bogota”, in da Costa’s handwriting.

####### Dimensions.

”Long. 26, diam. maj. 11 millim.”; figured specimen herein H 26.3, D 11.0, W 6.3.

####### Type material.

NHMUK 1907.11.21.51, holotype (da Costa coll.).

####### Remarks.

Sowerby writes ”I have seen as yet only one specimen of this species”. Therefore the specimen is considered as holotype.

####### Current systematic position.

Bulimulidae, *Drymaeus (Drymaeus) dacostae* (Sowerby III, 1892).

###### Bulimus darwini

Pfeiffer, 1846

http://species-id.net/wiki/Bulimus_darwini

Figs 13J , L66vii

Bulimus darwini Pfeiffer 1846a: 29; Pfeiffer 1848b: 199; Reeve 1848 [1848–1850]: pl. 21 fig. 136.

####### Type locality.

[Ecuador] ”Gallapagos [sic] Islands”.

####### Label.

”Galapagos Is.”, in E.A. Smith’s handwriting. M.C. label style III, V.

####### Dimensions.

”Long. 17, diam. 19 mill.”; figured specimen herein H 8.64, D 6.87 W 4.9.

####### Type material.

NHMUK 20100523, two possible syntypes, C. Darwin leg. (Cuming coll.).

####### Remarks.

The two juvenile specimens were labelled ”ustulatus Sow.” by E.A. Smith as coming from the Cuming museum, and were probably collected by Darwin during his visit to the archipelago with the ‘Beagle’. They are considered as possible syntypes. The current systematic position follows Richardson (1995: 217).

####### Current systematic position.

Bulimulidae, *Naesiotus darwini* (Pfeiffer, 1846).

###### Bulimus decussatus

Reeve, 1849

http://species-id.net/wiki/Bulimus_decussatus

Figs 56G–I , L16vi

Bulimus decussatus Reeve 1849 [1848–1850]: pl. 72 fig. 519; Reeve 1850: 99; Pfeiffer 1853d: 431; Breure 1979: 100.
Neopetraeus decussatus ; Breure 1978: 215 (lectotype designation).

####### Type locality.

”Andes of Caxamarca [sic, Cajamarca], Peru”.

####### Label.

”Andes of Caxamarca, Peru”; in unknown handwriting, obviously added in a later stage (see remarks).

####### Dimensions.

Not given; figured specimen herein H 37.7, D 16.5, W 7.7.

####### Type material.

NHMUK 1975180, lectotype (Cuming coll.).

####### Remarks.

The printed label, that is typical of specimens figured by Reeve, has been lost. The label, probably in the handwriting of a NHMUK employee during the latter half of the 19th century, refers to Reeve’s species number (519), stating that it is ”the type”. In the lower left corner, ”one on right” has been added (probably by E.A. Smith), which seems to indicate that the specimens originally were glued on board. There is a second note ”now marked x”, which apparently refers to the type specimen. See also *Bulimus myristicus* Reeve, 1849, which is a junior synonym (Breure 1978).

####### Current systematic position.

Bulimulidae, *Neopetraeus decussatus* (Reeve, 1849).

###### Bulimus delumbis

Reeve, 1849

http://species-id.net/wiki/Bulimus_delumbis

Figs 9C , L17i

Bulimus delumbis Reeve 1849 [1848–1850]: pl. 76 fig. 555; Pfeiffer 1853d: 418; Breure 1979: 53.
Bulimulus delumbis ; Pilsbry 1901 [1901–1902]: 138, pl. 25 fig. 15.
Bostryx delumbis ; Breure 1978: 71 (lectotype designation).

####### Type locality.

”—?”.

####### Label.

”Peru + Bolivia”, added in a later handwriting. M.C. label style I, V.

####### Dimensions.

Not given; figured specimen herein H 21.2, D 14.0, W 6.0.

####### Type material.

NHMUK 1975124, lectotype; 1975558, one paralectotype (Cuming coll.).

####### Remarks.

The current systematic position follows Richardson (1995: 21).

####### Current systematic position.

Bulimulidae, *Bostryx delumbis* (Reeve, 1849).

###### Bulimus demotus

Reeve, 1850

http://species-id.net/wiki/Bulimus_demotus

Figs 25F , L17iii

Bulimus feriatus Reeve 1848 [1848–1850]: pl. 54 fig. 354; Breure 1979: 118 (lectotype designation).
Bulimus demotus Reeve 1850 [1848–1850]: ii. New name for *Bulimus feriatus* Reeve, 1848 (Dec.), not Reeve, 1848 (Nov.); Pfeiffer 1853d: 340.
Drymaeus demotus ; Pilsbry 1898 [1897–1898]: 306, pl. 43 fig. 81.

####### Type locality.

”Venezuela”.

####### Label.

”Venezuela”. M.C. label style IV, V.

####### Dimensions.

Not given; figured specimen herein H 31.8, D 15.3, W 6.2.

####### Type material.

NHMUK 1975504, lectotype; 1975505, two paralectotypes (Cuming coll.).

####### Remarks.

The lectotype corresponds to Reeve’s figure. The current systematic position follows Richardson (1995: 131).

####### Current systematic position.

Bulimulidae, *Drymaeus (Mesembrinus) granadensis* (Pfeiffer, 1848).

###### Bulimus denickei

J.E. Gray, 1852

http://species-id.net/wiki/Bulimus_denickei

Figs 7G , L17iv

Bulimus denickei J.E. Gray 1852: 92.
Bostryx denickei ; Breure 1979: 53.

####### Type locality.

[Peru, Dept. Arequipa] ”Chala, near Callao”.

####### Label.

”Chala, near Callao”. M.C. label style IV.

####### Dimensions.

Not given; figured specimen herein H 26.4, D 17.5, W 5.9.

####### Type material.

NHMUK 1851.5.15.1–3, three syntypes, E. Denicke leg. (Cuming coll.).

####### Remarks.

The current systematic position follows Richardson (1995: 40).

####### Current systematic position.

Bulimulidae, *Bostryx reentsi* (Philippi, 1851).

###### Bulimus dentritis

Morelet, 1863

http://species-id.net/wiki/Bulimus_dentritis

Figs 16G , L17ii

Bulimus dentritis Morelet, 1863: 206, pl. 9 fig. 5; Breure 1979: 62.
Bulimulus dentritis ; Breure 1978: 72 (lectotype designation).

####### Type locality.

[Peru, Dept. Cuzco] ”près de Huiro, vallée de Santa-Anna”.

####### Label.

”Miota, Vallée de S^ta^ Anna”, in Morelet’s handwriting.

####### Dimensions.

”Longit. 20, diam. 8 mill.”; figured specimen herein H 19.9, D 7.42, W.

####### Type material.

NHMUK 1893.2.4.237, paralectotype (Morelet coll.).

####### Remarks.

The species is hitherto classified as *Bulimulus*. However, the protoconch sculpture consists of axial riblets; it is here re-classified under *Naesiotus* sensu Breure 1979. The NHMUK specimen fits the measurements given by Morelet, but is not from the type locality. Other type material is present in MHNG from various other localities, of which one specimen from the type locality was designated lectotype (Breure 1978).

####### Current systematic position.

Bulimulidae, *Naesiotus dentritis* (Morelet, 1863) (**comb.n.**).

###### Bulimus depictus

Reeve, 1849

http://species-id.net/wiki/Bulimus_depictus

Figs 22C , L17v

Bulimus depictus Reeve 1849 [1848–1850]: pl. 74 fig. 529; Pfeiffer 1853d: 337; Breure 1979: 118 (lectotype designation).
Drymaeus depictus ; Pilsbry 1898 [1897–1898]: 290, pl. 45 fig. 17.
Drymaeus (Mesembrinus) depictus ; Breure and Eskens 1981: 55, pl. 6 fig. 5.

####### Type locality.

”New Granada”.

####### Label.

”Venezuela”, added in a later handwriting. M.C. label style IV, V.

####### Dimensions.

Not given; figured specimen herein H 27.9, D 12.1, W 5+.

####### Type material.

NHMUK 1975529, lectotype; 1975530, two paralectotypes (Cuming coll.).

####### Remarks.

The top of the lectotype is damaged. The difference between the published type locality and the locality on the label is explained by political changes around 1850.

####### Current systematic position.

Bulimulidae, *Drymaeus (Mesembrinus) depictus* (Reeve, 1849).

###### Bulimus depstus

Reeve, 1849

http://species-id.net/wiki/Bulimus_depstus

Figs 6B , L17vi

Bulimus depstus Reeve 1849 [1848–1850]: pl. 73 fig. 525 [text species 524]; Reeve 1850: 97; Pfeiffer 1853d: 428; Breure 1979: 53.
Bostryx depstus ; Breure 1978: 73 (lectotype designation).

####### Type locality.

”Chachapoyas, Alto-Peru”.

####### Label.

No label found (see remarks).

####### Dimensions.

Not given; figured specimen herein H 18.5, D 9.71, W 7.1.

####### Type material.

NHMUK 1975318, lectotype; 1975319, two paralectotypes (Cuming coll.).

####### Remarks.

Breure (1978) stated he found a label with the text ”Peru”; however, this label may have been lost.

####### Current systematic position.

Bulimulidae, *Bostryx depstus* (Reeve, 1849).

###### Bulinus derelictus

Broderip, 1832

http://species-id.net/wiki/Bulinus_derelictus

Figs 10H , L17vii

Bulinus derelictus Broderip in Broderip and Sowerby I 1832b: 107; Reeve 1848 [1848–1850]: pl. 23 fig. 151; Neubert and Janssen 2004: 208, pl. 6 fig. 65.

####### Type locality.

”Cobijam Bolivia [now Chile] (Puerto del Mar)”.

####### Label.

”Bolivia S. America”, with a taxon label in Pfeiffer’s handwriting. M.C. label style I, V.

####### Dimensions.

”Long. 6/12, lat. 5/8 poll. [H 12.7, D 15.8 mm, see remarks]”; figured specimen herein H 30.3, D 18.8, W 6.6.

####### Type material.

NHMUK 20100609, four probable syntypes (Cuming coll.).

####### Remarks.

Broderip described this taxon from the Cuming collection, but did not state on how many specimens his description was based. The original measurments seem to be erroneous, as these indicate a height/diameter ratio uncommon for this genus. Neubert and Janssen (2004) reported three specimens, with a maximum height of 23.1 and diameter of 15.3 mm, labelled ”Peru” (ex Cuming), which they consider syntypes. The specimen corresponding to Reeve’s figure has been found in the NHMUK collection, together with three others; they are considered probable syntypes.

####### Current systematic position.

Bulimulidae, *Bostryx derelictus* (Broderip in Broderip and Sowerby I 1832).

###### Bulimus deshayesi

Pfeiffer, 1845

http://species-id.net/wiki/Bulimus_deshayesi

Figs 24D , L18i

Bulimus deshayesi Pfeiffer 1845c: 73; Pfeiffer 1848b: 200; Reeve 1848 [1848–1850]: pl. 40 fig. 250; Breure 1979: 118 (lectotype designation).
Drymaeus deshayesi ; Pilsbry 1898 [1897–1898]: 303, pl. 45 fig. 27; Linares and Vera 2012: 194.
Drymaeus (Mesembrinus) deshayesi ; Breure and Eskens 1981: 55, pl 7 fig. 1.

####### Type locality.

”Locality unknown”.

####### Label.

”New Granada”, taxon label in Pfeiffer’s handwriting. M.C. label style IV, V.

####### Dimensions.

”Long. 45, diam. 15 mill.”; figured specimen herein H 44.5, D 16.8, W 8.1.

####### Type material.

NHMUK 1975526, lectotype; 1975527, one paralectotype (Cuming coll.).

####### Remarks.

The current systematic position follows the revision of Breure and Borrero (unpublished data).

####### Current systematic position.

Bulimulidae, *Drymaeus (Mesembrinus) deshayesi* (Pfeiffer, 1845).

###### Bulimus devians

Dohrn, 1863

http://species-id.net/wiki/Bulimus_devians

Figs 4C , L18ii

Bulimus devians Dohrn 1863: 155.

####### Type locality.

No type locality given [Peru].

####### Label.

”[Peru]”, added in a later handwriting. M.C. label style III.

####### Dimensions.

”Long. 15, lat. 6 mill.”; figured specimen herein H 14.9, D 6.78, W 5.1.

####### Type material.

NHMUK 1975339, one possible syntype (Cuming coll.).

####### Remarks.

Dohrn’s collection is known to be completely destroyed in the 1939–1945 war (Dance 1966, Köhler 2007). The specimen fits the original measurement, although it is unknown on how many specimens Dohrn based his description. Moreover, the lack of original data on the label prevents to ascertain the status of this specimen, and the specimen is only treated as possible syntype.

####### Current systematic position.

Bulimulidae, *Bostryx devians* (Dohrn, 1863).

###### Bulinus discrepans

Sowerby I, 1833

http://species-id.net/wiki/Bulinus_discrepans

Figs 23D , L18iii

Bulinus discrepans Sowerby I 1833b: 72; Reeve 1848 [1848–1850]: pl. 23 fig. 145.

####### Type locality.

”Americâ Centrali” (see remarks).

####### Label.

 ”Conchagua, C. America”. M.C. label style IV, V.

####### Dimensions.

”Long. 0.7, lat. 0.33 poll. [H 17.7, D 8.4 mm]”; figured specimen herein H 20.7, D 9.3, W 6.5.

####### Type material.

NHMUK 1975181, four syntypes (Cuming coll.).

####### Remarks.

Sowerby added is his remarks ”This was found at Conchagua” [El Salvador, dept. La Union], which is thus the type locality. Of the four specimens in the lot, two are juveniles and one is subadult; the largest specimen is marked ”59”. The current systematic position follows Thompson (2011: 119).

####### Current systematic position.

Bulimulidae, *Drymaeus (Mesembrinus) discrepans* (Sowerby I, 1833).

###### Bulimus dombeyanus

‘Férussac’ Pfeiffer, 1846

http://species-id.net/wiki/Bulimus_dombeyanus

Figs 37I–K , L18v

Helix dombeyana ‘F[érussac]’ Pfeiffer 1842: 76 (= *Bulimus*) [nomen nudum].
Bulimus dombeyanus ‘Fér.’ Pfeiffer 1842: 114 [nomen nudum]; ‘Fér.’ Pfeiffer 1846c: 83.

####### Type locality.

”Peru (Mus. Britt.)”.

####### Label.

”Peru”. M.C. label style IV.

####### Dimensions.

”Long. 56, diam. 26 mill.”; figured specimen herein H 56.8, D 31.7, W 7.1.

####### Type material.

NHMUK 1844.10.2.32, syntype (ex Férussac, ”purch^d^ of Turner”).

####### Remarks.

This taxon is generally regarded as a Mexican species (Pilsbry 1899, Solem 1957, Thompson 2011); the locality thus could be a case of mislabeling.

####### Current systematic position.

Bulimulidae, *Drymaeus (Drymaeus) dombeyanus* (Pfeiffer, 1846).

###### Bulimus dubius

Pfeiffer, 1853

http://species-id.net/wiki/Bulimus_dubius

Figs 25C , L18iv

Bulimus dubius Pfeiffer 1853b: 257; Breure 1979: 118 (lectotype designation).
Drymaeus (Mesembrinus) dubius ; Breure and Eskens 1981: 57, pl. 7 fig. 10.
Drymaeus dubius ; Linares and Vera 2012: 194.

####### Type locality.

”in Andibus Novae Granadae”.

####### Label.

”Andes, N. Granada”, taxon label in Pfeiffer’s handwriting. M.C. label style IV.

####### Dimensions.

”Long. 28, diam. 10 mill.”; figured specimen herein H 27.1, D 11.0, W 6.2+.

####### Type material.

NHMUK 1975519, lectotype; 1975520, two paralectotypes (Cuming coll.).

####### Remarks.

Of the lectotype the lip and the apex are damaged. Of the paralectotypes, one shell is broken.

####### Current systematic position.

Bulimulidae, *Drymaeus (Mesembrinus) dubius* (Pfeiffer, 1853).

###### Bulimulus (Drymaeus) dukinfieldi

Melvill, 1900

http://species-id.net/wiki/Bulimulus_dukinfieldi

Figs 60C–D , L19i

Bulimulus (Drymaeus) dukinfieldi Melvill 1900: 116, fig.
Bulimulus dukinfieldi ? ; Breure 1979: 62.
Bulimulus dukenfieldi ; Simone 2006: 118, fig. 359.
Thaumastus dukinfieldi ; Simone 2006: 152, fig. 518.

####### Type locality.

”Salto Grande do Rio dos Patos (upper waters of Rio Ivahy), Paraná, Brazil”.

####### Label.

 ”Salto Grande do Rio / dos Patos. (Upper waters of River Ivahy). / Paraná. Brazil.”.

####### Dimensions.

”Long. 28, lat. 13 mm”; figured specimen herein H 28.1, D 14.8, W 5.9.

####### Type material.

NHMUK 1900.9.27.2, lectotype (ex Melvill).

####### Remarks.

Melvill wrote ”Two specimens, precisely similar, were obligingly handed me by Mr. R. Dukinfield Darbishire”; ”the type” was placed in the NHMUK collection, the whereabouts of the second specimen are unknown. The reference to ”HT 1900.9.27.2” in Breure 1979 has to be interpreted as lectotype designation under Art. 74.6 ICZN. This shell seems to have been damaged several times during life-time, which may have influenced the shape of the shell and aperture. This is probably the reason why this taxon has been referred to different genera (Simone 2006: 118, 152); the classification of Breure (1979) is here tentatively retained until further studies have clarified the systematic position of this nomen inquirendum.

####### Current systematic position.

*Bulimulus* (?) *dukinfieldi* Melvill, 1900. Nomen inquirendum.

###### Bulimus dunkeri

Pfeiffer, 1846

http://species-id.net/wiki/Bulimus_dunkeri

Figs 50D–F , L19ii

Bulimus dunkeri Pfeiffer in Philippi 1846 [1845–1847]: 112, pl. 4 fig. 10; Breure 1979: 108 (lectotype designation).
Drymaeus (Drymaeus) dunkeri ; Breure and Eskens 1981: 20; Thompson 2011: 110.

####### Type locality.

”respublica mexicana, prope Michoacan”.

####### Label.

”Michoacan”, taxon label in Pfeiffer’s handwriting. M.C. label style IV.

####### Dimensions.

 ”Long. 18, diam. 8’’’ [H 39.2, D 17.4 mm]”; figured specimen herein H 34.8, D 18.5, W 6.0.

####### Type material.

NHMUK 1975512, lectotype; 1975513, two paralectotypes (Cuming coll.).

####### Remarks.

The original measurements are interpreted as German lines (2.18 mm), and do not correspond to those of the specimen in the NHMUK. However, Pfeiffer’s collection is known to have been destroyed (Dance 1966), and type material of this taxon is neither in the SMF (Neubert and Janssen 2004) nor ZMB (Köhler 2007) collections. The current systematic position follows Thompson (2011).

####### Current systematic position.

Bulimulidae, *Drymaeus (Drymaeus) dunkeri dunkeri* (Pfeiffer, 1846).

###### Bulimulus (Peronaeus) durangoanus

Martens, 1893

http://species-id.net/wiki/Bulimulus_durangoanus

Figs 73F–H , L19iii

Bulimulus (Peronaeus) durangoanus Martens 1893 [1890–1901]: 246, pl. 15 figs 11–11a.
Bulimulus durangoanus ; Pilsbry 1897 [1897–1898]: 127, pl. 18 figs 32–33.

####### Type locality.

”N. Mexico, Villa Lerdo, State of Durango”.

####### Label.

”Mexico”, taxon label in Martens’ handwriting.

####### Dimensions.

”Long. 15, diam. 6 (...) millim.”. Figured specimen H 15.0, D 6.4, W 6.3.

####### Type material.

NHMUK 1901.6.22.871, holotype (ex Godman).

####### Remarks.

Martens wrote ”I have seen only one specimen of this species”; the specimen found is thus the holotype. The current systematic position follows Thompson (2011: 124).

####### Current systematic position.

Bulimulidae, *Naesiotus durangoanus* (Martens, 1893).

###### Bulimus dutaillyi

Pfeiffer, 1857

http://species-id.net/wiki/Bulimus_dutaillyi

Figs 22G , L19iv

Bulimus dutaillyi Pfeiffer 1857d: 390; Breure 1979: 118 (lectotype designation).
Drymaeus (Mesembrinus) dutaillyi ; Breure and Eskens 1981: 57, pl. 7 fig. 2; Simone 2006: 145, fig. 485.

####### Type locality.

”Brazils”.

####### Label.

 ”Brazils Mon. Dutailly”, taxon label in Pfeiffer’s handwriting. M.C. label style I.

####### Dimensions.

”Long. 31, diam. 12 mill.”; figured specimen herein H 30.8, D 12.5, W 7.1.

####### Type material.

NHMUK 1975516, lectotype, Dutailly leg. (Cuming coll.).

####### Remarks.

Simone (2006) erroneously had the shell height as 33.0 mm.

####### Current systematic position.

Bulimulidae, *Drymaeus (Mesembrinus) dutaillyi* (Pfeiffer, 1857).

###### Bulimus dysoni

Pfeiffer, 1846

http://species-id.net/wiki/Bulimus_dysoni

Figs 62K–L , L19v

Bulimus dysoni Pfeiffer 1846b: 39; Pfeiffer 1848b: 183; Reeve 1849 [1848–1850]: pl. 62 fig. 425; Breure 1979: 62.
Bulimulus dysoni ; Pilsbry 1897 [1897–1898]: 56, pl. 10 fig. 83.
Bulimulus (Bulimulus) dysoni ; Breure 1974: 47, pl. 4 figs 4–5.

####### Type locality.

”Honduras”.

####### Label.

”Honduras”, taxon label in Pfeiffer’s handwriting. M.C. label style IV, V.

####### Dimensions.

”Long. 20, diam. 9 1/2 mill.”; figured specimen herein H 19.2, D 9.5, W 7.4.

####### Type material.

NHMUK 1975453, three syntypes (Cuming coll.).

####### Remarks.

The specimen figured by Reeve is damaged at the penultimate whorl.

####### Current systematic position.

Bulimulidae, *Bulimulus dysoni* (Pfeiffer, 1846).

###### Bulimus edwardsi

Morelet, 1863

http://species-id.net/wiki/Bulimus_edwardsi

Figs 67C–D , 67L , L19vi

Bulimus edwardsi Morelet 1863: 182, pl. 9 fig. 1; Breure 1979: 88.
Bulimulus edwardsi ; Pilsbry 1897 [1897–1898]: 27, pl. 7 figs 11–13.

####### Type locality.

[Peru, Dept. Huancavelica] ”le type (...) Huancabelica; la variété, (...) vallée de Huanta” [Dept. Ayacucho].

####### Label.

”Pérou Huanta”, taxon label (”var.”) in Morelet’s handwriting.

####### Dimensions.

”Longit. 29; diam. 12 mill.”; figured specimen herein H 25.4, D 13.3, W 5.7.

####### Type material.

NHMUK 1893.2.4.177–178, two syntypes (Morelet coll.).

####### Remarks.

Morelet considered this to be a variable taxon and distinguished explicitly a smaller variety ”Testa minor, (...)”. Crawford (1939: 329) compared the two specimens in NHMUK to *Scutalus bicolor* (Sowerby, 1835), and expressed doubts whether the variety is identical to typical *edwardsi*; the typical form is considered by him as a synonym of *Scutalus culmineus* (d’Orbigny, 1835). With the current material at hand we prefer to treat Morelet’s material as a geographical form. Weyrauch (1967a: 386) considers Morelet’s taxon as a subspecies of *Scutalus culmineus*. This species is now placed in the genus *Kuschelenia* Hylton Scott, 1951.

####### Current systematic position.

Bulimulidae, *Kuschelenia (Kuschelenia) culmineus edwardsi* (Morelet, 1863) (**comb. n.**).

###### Bulimus effeminatus

Reeve, 1848

http://species-id.net/wiki/Bulimus_effeminatus

Figs 62A–B , L20i

Bulimus effeminatus Reeve 1848 [1848–1850]: pl. 51 fig. 338; Breure 1979: 62.
Bulimulus effeminatus ; Breure 1978: 143, pl. 9 figs 10–11 (lectotype designation).

####### Type locality.

”—?”.

####### Label.

”Valley of the / Madeleine [= Río Magdalena, Colombia] / N. Grenada”. M.C. label style IV, V.

####### Dimensions.

Not given; figured specimen herein H 31.1, D 14.2, W 7.9.

####### Type material.

NHMUK 1975508, lectotype; 1975509, two paralectotypes (Cuming coll.).

####### Remarks.

This taxon was erroneously placed under *Drymaeus* by Richardson (1995: 119).

####### Current systematic position.

Bulimulidae, *Bulimulus effeminatus* (Reeve, 1848).

###### Bulimus electrum

Reeve, 1848

http://species-id.net/wiki/Bulimus_electrum

Figs 22E , L20ii

Bulimus electrum Reeve 1848 [1848–1850]: pl. 56 fig. 373; Breure 1979: 118 (lectotype designation).
Drymaeus electrum ; Pilsbry 1898 [1897–1898]: 310, pl. 41 fig. 36.
Drymaeus (Mesembrinus) electrum ; Breure and Eskens 1981: 57, pl. 7 fig. 3.

####### Type locality.

”Venezuela”.

####### Label.

”Venezuela”. M.C. label style IV, V.

####### Dimensions.

Not given; figured specimen herein H 29.6, D 14.1, W 5.8.

####### Type material.

NHMUK 1975510, lectotype; 1975511, two paralectotypes (Cuming coll.).

####### Remarks.

The lectotype corresponds to Reeve’s original figure. The current systematic position is in accordance with Richardson (1995).

####### Current systematic position.

Bulimulidae, *Drymaeus (Mesembrinus) electrum* (Reeve, 1848).

###### Drymaeus elsteri

da Costa, 1901

http://species-id.net/wiki/Drymaeus_elsteri

Figs 33G–I , L20iii

Drymaeus elsteri da Costa 1901: 238, pl. 24 fig. 6; Pilsbry 1901 [1901–1902]: 156, pl. 48 fig. 53; Breure 1979: 108 (lectotype designation).

####### Type locality.

”Chachapoyas, prov. Amazonas, Peru”.

####### Label.

”Chacapoyas [sic]”, in da Costa’s handwriting.

####### Dimensions.

”Long. 34, diam. 15 mm.”; figured specimen herein H 33.9, D 14.9, W 6.0.

####### Type material.

NHMUK 1907.11.21.34, lectotype; 1907.11.21.35–36, two paralectotypes (da Costa coll.).

####### Remarks.

da Costa indicated that he had several specimens at hand when describing this taxon. The lectotype chosen is closely matching the original measurements.

####### Current systematic position.

Bulimulidae, *Drymaeus (Drymaeus) elsteri* da Costa, 1901.

###### Bulimus emaciatus

Morelet, 1863

http://species-id.net/wiki/Bulimus_emaciatus

Figs 3E , L20iv

Bulimus emaciatus Morelet 1863: 201, pl. 11 fig. 10; Breure 1979: 53.
Bulimulus (Peronaeus) emaciatus ; Pilsbry 1896 [1895–1896]: 143, pl. 45 figs 27–28.
Bostryx emaciatus ; Breure 1978: 74, fig. 101 (lectotype designation).

####### Type locality.

[Peru] ”les vallées et sur les plateaux de l’intérieur de la Sierra, depuis Ayacucho jusqu’au Cuzco” (see remarks).

####### Label.

”Pérou”, taxon label in Morelet’s handwriting.

####### Dimensions.

 ”Longit. 22, diam. 5 1/2 mill.”; figured specimen herein H 21.7, D 5.43, W 10.6.

####### Type material.

NHMUK 1893.2.4.248–250, three paralectotypes (Morelet coll.).

####### Remarks.

Breure (1978) upon selecting a lectotype from the MHNG collection, also fixated the type locality to Acobamba according to the label information of the specimen. Although there are several localities with that name in southern Peru, it is now assumed that it should be in Dept. Huancavelica [S -12.866667, W -74.566667], given the context of the other localities mentioned in Breure (1978). The current systematic position follows Richardson (1995: 23).

####### Current systematic position.

Bulimulidae, *Bostryx emaciatus* (Morelet, 1863).

###### Bulimus erectus

Reeve 1849

http://species-id.net/wiki/Bulimus_erectus

Figs 60G–H , L20v

Bulimus erectus Reeve 1849 [1848–1850]: pl. 58 fig. 392; Pfeiffer 1853d: 439.
Bulimulus erectus ; Pilsbry 1897 [1897–1898]: 60, pl. 10 fig. 99.

####### Type locality.

”Curiana, Venezuela”.

####### Label.

”Curiana, Venezuela”. M.C. label style I, V.

####### Dimensions.

Not given; figured specimen herein H 22.0 D 12.24 W 6.8.

####### Type material.

NHMUK 20100516, three syntypes, Dyson leg. (Cuming coll.).

####### Remarks.

Although none of the specimens exactly fits Reeve’s figure, they are regarded as possibly syntypes as they are originating from Dyson. See also *Bulimus cacticolus* Reeve, 1849.

####### Current systematic position.

Bulimulidae, *Bulimulus erectus* (Reeve, 1849).

###### Bulimus erosus

Broderip in Broderip and Sowerby I 1832

http://species-id.net/wiki/Bulimus_erosus

Figs 7A , L20vi

Bulimus erosus Broderip in Broderip and Sowerby I 1832b: 106; Reeve 1848 [1848–1850]: pl. 22 fig. 140.
Bulimulus (Lissoacme) erosus ; Pilsbry 1896 [1895–1896]: 160, pl. 49 fig. 34.

####### Type locality.

”Peruviâ, Huantajaya near Iquiqui”.

####### Label.

”Peru”, taxon label in Pfeiffer’s handwriting. M.C. label style I, V.

####### Dimensions.

”long. 11/12, lat. 6/12 poll. [H 23.2, D 12.66 mm]”; figured specimen herein H 22.9, D 13.32, W 6.3.

####### Type material.

NHMUK 20100612, five syntypes (Cuming coll.).

####### Remarks.

Breure (1979: 136), not having seen any type material, considered this taxon doubtfully a *Bostryx* and listed it as nomen inquirendum. The specimen now found in the NHMUK collection confirms this classification and agrees with Richardson (1995: 24).

####### Current systematic position.

Bulimulidae, *Bostryx erosus* (Broderip in Broderip and Sowerby I 1832).

###### Bulimus erubescens

Pfeiffer, 1847

http://species-id.net/wiki/Bulimus_erubescens

Figs 25D , L21i

Bulimus erubescens Pfeiffer 1847: 112; Reeve 1848 [1848–1850]: pl. 57 fig. 381; Breure 1979: 118 (lectotype designation).
Drymaeus erubescens ; Pilsbry 1899: 9, pl. 13 fig. 89.

####### Type locality.

”Locality unknown”.

####### Label.

”Jamaica”, added in a later handwriting on the front of the cardboard. M.C. label style I, V.

####### Dimensions.

”Long. 24, diam. 10 mill.”; figured specimen herein H 24.2, D 9.74 W 6.3.

####### Type material.

NHMUK 1975562, lectotype; 1975563, paralectotype (Cuming coll.).

####### Remarks.

The lectotype corresponds to Pfeiffer’s original measurements and is figured in Reeve (1848 [1848–1850]). The locality is probably erroneous; the species is not listed in Rosenberg and Muratov (2005). This taxon is considered by Richardson (1995: 172) to be a synonym of *Drymaeus (Mesembrinus) rufescens* (J.E. Gray, 1825), described from Jamaica but with very poor diagnostic features. The locality of the latter taxon may also be in error; the type material of *Bulimus rufescens* Gray, 1825 has not been located and may be lost.

####### Current systematic position.

Bulimulidae, *Drymaeus (Mesembrinus)* (?) *rufescens* (J.E. Gray, 1825).

###### Bulinus eschariferus

Sowerby I, 1838

http://species-id.net/wiki/Bulinus_eschariferus

Figs 74A , L21ii

Bulinus eschariferus Sowerby I 1838 [Sowerby I and II 1832–1841]: fig. 85.
Bulimus eschariferus ; Reeve 1848 [1848–1850]: pl. 20 fig. 121.
Bulimulus eschariferus ; Pilsbry 1897 [1897–1898]: 108, pl. 22 fig. 9.

####### Type locality.

”Galapagos”.

####### Label.

”Chatham Is. / Galapagos”, in E.A. Smith’s handwriting. M.C. label style III.

####### Dimensions.

Not given. Figured specimen H 18.4, D 7.1, W 7.5.

####### Type material.

NHMUK 1975153, one possible syntype (Cuming coll.).

####### Remarks.

Sowerby did not state on how many specimens he based himself. The specimen found was figured by Reeve and the label is evidently based on the information given by him. The material is considered as possible syntype; see Breure and Ablett 2011: 2–12 for historical details on the Cuming collection and the relationship with Sowerby’s and Reeve’s taxa.

####### Current systematic position.

Bulimulidae, *Naesiotus eschariferus* (Sowerby I, 1833).

###### Bulimus excoriatus

Pfeiffer, 1855

http://species-id.net/wiki/Bulimus_excoriatus

Figs 58F–G , L21iii

Bulimus excoriatus Pfeiffer 1855g: 123; Breure 1979: 100.
Neopetraeus excoriatus ; Breure 1978: 215, pl. 3 fig. 2 (lectotype designation).

####### Type locality.

”Andes of Peru (*Captain Keppell*)”.

####### Label.

”Andes of Peru”, taxon label in Pfeiffer’s handwriting. M.C. label style IV.

####### Dimensions.

”Long. 39, diam. 19 mill.”; figured specimen herein H 38.4, D 25.2, W 6.2.

####### Type material.

NHMUK 1975500, lectotype, Keppell leg. (Cuming coll.).

####### Remarks.

The specimen is subadult and aged; it is possibly a synonym of *Neopetraeus catamarcanus* (Pfeiffer, 1858), in which case *Neopetraeus excoriatus* would have priority.

####### Current systematic position.

Bulimulidae, *Neopetraeus excoriatus* (Pfeiffer, 1855).

###### Bulimus exornatus

Reeve, 1849

http://species-id.net/wiki/Bulimus_exornatus

Fig. L21iv

Bulimus exornatus Reeve 1849 [1848–1850]: pl. 77 fig. 560; Breure 1979: 69 (lectotype designation).
Bulimulus (Lissoacme) exornatus ; Pilsbry 1896 [1895–1896]: 171, pl. 50 fig. 55.

####### Type locality.

”Chilon, Bolivia” [Dept. Santa Cruz].

####### Label.

”Bolivia”. M.C. label style I, V.

####### Dimensions.

Not given; lectotype H 13.1, D —, W 5.5.

####### Type material.

NHMUK 1975331, lectotype (Cuming coll.).

####### Remarks.

The lectotype was damaged when it was returned from a loan in 1988; the remaining shell is too fragile to be figured herein. The lot consisted of a second specimen (1975332), which appears to be a juvenile *Drymaeus* species; this specimen is now excluded as type material.

####### Current systematic position.

Bulimulidae, *Naesiotus exornatus* (Reeve, 1849).

###### Drymaeus exoticus

da Costa, 1901

http://species-id.net/wiki/Drymaeus_exoticus

Figs 33D–F , L21v

Drymaeus exoticus da Costa 1901: 239, pl. 24 fig. 10; Pilsbry 1901 [1901–1902]: 156, pl. 48 fig. 52; Breure 1979: 109; Linares and Vera 2012: 185.

####### Type locality.

”The hot country, Upper Magdalena River, Colombia”.

####### Label.

”hot country / Columbia”, taxon label in da Costa’s handwriting.

####### Dimensions.

”Long. 23.5, diam. 11 mm.”; figured specimen herein H 24.8, D 12.2, W 6.1.

####### Type material.

NHMUK 1907.11.21.38, lectotype (da Costa coll.).

####### Remarks.

This material was considered as holotype by Breure (1979). However, da Costa did not state on how many specimens his description was based. Breure (unpublished data) found another specimen in the Dautzenberg collection with a label in da Costa’s handwriting, but without enough evidence to consider it a type specimen. However, it cannot be excluded that da Costa had more than one specimen at hand; according to Art. 74.6 ICZN, the NHMUK specimen should be considered as a lectotype.

####### Current systematic position.

Bulimulidae, *Drymaeus (Drymaeus) exoticus* (da Costa, 1901).

###### Bulimulus (Drymaeus) expatriatus

Preston, 1909

http://species-id.net/wiki/Bulimulus_expatriatus

Figs 51A–C , L21vi

Bulimulus (Drymaeus) expatriatus Preston 1909: 510, pl. 10 fig. 4; Breure 1979: 109 (lectotype designation).
Drymaeus (Drymaeus) expatriatus ; Breure and Eskens 1981: 21.

####### Type locality.

”E. Bolivia”.

####### Label.

 ”E. Bolivia”, taxon label in Preston’s handwriting.

####### Dimensions.

”Alt. 28, diam. maj. 11.5 mm”; figured specimen herein H 28.1, D 11.5, W 7.0.

####### Type material.

NHMUK 1975201, lectotype; 1915.1.6.43, one paralectotype (ex Preston).

####### Remarks.

The paralectotype is a juvenile shell.

####### Current systematic position.

Bulimulidae, *Drymaeus (Drymaeus) expatriatus* (Preston, 1909).

###### Bulimus fabrefactus

Reeve, 1848

http://species-id.net/wiki/Bulimus_fabrefactus

Figs 38D–F , L22i

Bulimus fabrefactus Reeve 1848 [1848–1850]: pl. 49 fig. 319; Breure 1979: 109 (lectotype designation).
Drymaeus fabrefactus ; Pilsbry 1898 [1897–1898]: 260, pl. 40 fig. 5; Linares and Vera 2012: 185.
Drymaeus (Drymaeus) fabrefactus ; Breure and Eskens 1981: 22, pl. 5 fig. 4.

####### Type locality.

[Venezuela] ”Province of Merida, New Granada”.

####### Label.

”New Granada”. M.C. label style I, V.

####### Dimensions.

Not given; figured specimen herein H 38.5, D 16.5, W 7.2.

####### Type material.

NHMUK 1975531, lectotype (Cuming coll.).

####### Remarks.

Linares and Vera (2012) have a number of specific localities for this taxon within Colombia.

####### Current systematic position.

Bulimulidae, *Drymaeus (Drymaeus) fabrefactus* (Reeve, 1848).

###### Bulimus fallax

Pfeiffer, 1853

http://species-id.net/wiki/Bulimus_fallax

Figs 26D–F , L21vii

Bulimus fallax Pfeiffer 1853d: 375; Pfeiffer 1854 in Küster and Pfeiffer 1840–1865: 98, pl. 32 figs 5–6.
Drymaeus fallax ; Pilsbry 1898 [1897–1898]: 239, pl. 33 figs 43–44.

####### Type locality.

[Ecuador] ”Tunguragua reipublicae Aequatoris (*Bourcier*)”.

####### Label.

”Tunguragua / Equador / Mons^r^ Boissier [sic] / Consul General”, taxon label in Pfeiffer’s handwriting. M.C. label style IV.

####### Dimensions.

”Long. 24, diam. 12 mill.”; figured specimen herein H 22.4, D 13.8, W 5.7.

####### Type material.

NHMUK 1969142, lectotype and one paralectotype, Bourcier leg. (Cuming coll.).

####### Remarks.

Given the label, there is no doubt that this material was part of the type series. One specimen is now designated lectotype (**design. n.**) to define this poorly understood taxon.

####### Current systematic position.

Bulimulidae, *Drymaeus (Drymaeus) fallax* (Pfeiffer, 1853).

###### Bulimus farrisi

Pfeiffer, 1858

http://species-id.net/wiki/Bulimus_farrisi

Figs 45J–L , L22ii

Bulimus farrisi Pfeiffer 1858a: 258, pl. 42 fig. 8; Breure 1979: 109 (lectotype designation).
Drymaeus farrisi ; Pilsbry 1898 [1897–1898]: 268, pl. 47 fig. 6.
Drymaeus (Drymaeus) farrisi ; Breure and Eskens 1981: 22.

####### Type locality.

”Province of Patas, Andes of Peru (*Dr. Farris*)”.

####### Label.

”Province of Patas, / Andes of Peru / D^r^. Farris”, taxon label in Pfeiffer’s handwriting. M.C. label style IV.

####### Dimensions.

”Long. 47, diam. 16 mill.”; figured specimen herein H 46.8, D 18.4, W 7.2.

####### Type material.

NHMUK 1975506, lectotype; 1975507, three paralectotypes, Farris leg. (Cuming coll.).

####### Remarks.

Breure and Eskens (1981) stated they had seen four paralectotypes. Lot NHMUK 1975509 seems to be missing.

####### Current systematic position.

Bulimulidae, *Drymaeus (Drymaeus) farrisi* (Pfeiffer, 1858).

###### Bulimus felix

Pfeiffer, 1862

http://species-id.net/wiki/Bulimus_felix

Figs 45D–F , L22iii

Bulimus felix Pfeiffer 1862: 387, pl. 37 fig. 2.
Drymaeus felix ; Pilsbry 1898 [1897–1898]: 211, pl. 35 fig. 20; Linares and Vera 2012: 185 [partim].

####### Type locality.

”New Granada”.

####### Label.

”New Granada”, taxon label in Pfeiffer’s handwriting. M.C. label style III.

####### Dimensions.

”Long. 33, diam. 13 mill.”; lectotype H 34.0, D 14.8, W 6.2.

####### Type material.

NHMUK 1975206, lectotype and three paralectotypes (Cuming coll.).

####### Remarks.

 Pfeiffer did not state on how many specimens his description was based. A lot with four specimens was found, which is considered as type material. The specimen marked with ‘x’ has been selected as lectotype (**design. n.**) to define this often confused taxon.

####### Current systematic position.

Bulimulidae, *Drymaeus (Drymaeus) felix* (Pfeiffer, 1862).

###### Bulimus fenestratus

Pfeiffer, 1846

http://species-id.net/wiki/Bulimus_fenestratus

Figs 51J–L , L22iv

Bulimus fenestratus Pfeiffer 1846a: 29; Pfeiffer 1848b: 101; Reeve 1848 [1848–1850]: pl. 36 fig. 214; Breure 1979: 109 (lectotype designation).
Drymaeus (Drymaeus) fenestratus ; Pilsbry 1899: 34, pl. 7 fig. 11.
Drymaeus (Drymaeus) fenestratus ; Breure and Eskens 1981: 22, pl. 5 fig. 2; Thompson 2011: 110.

####### Type locality.

”Mexico”.

####### Label.

”Mexico”, taxon label in Pfeiffer’s handwriting. M.C. label style IV, V.

####### Dimensions.

”Long. 45, diam. 18 mill.”; figured specimen herein H 44.0, D 20.3, W 6.6.

####### Type material.

NHMUK 1975525, lectotype (Cuming coll.).

####### Remarks.

The current systematic position follows Thompson (2011).

####### Current systematic position.

Bulimulidae, *Drymaeus (Drymaeus) fenestratus* (Pfeiffer, 1846).

###### Bulimus feriatus

Reeve, 1848

http://species-id.net/wiki/Bulimus_feriatus

Figs 17I–K , L22vi

Bulimus feriatus Reeve 1848 [1848–1850]: pl. 54 fig. 354; Pfeiffer 1853d: 323; Breure 1979: 118 (lectotype designation).
Drymaeus feriatus ; Pilsbry 1898 [1897–1898]: 203, pl. 34 fig. 10.
Drymaeus (Mesembrinus) feriatus ; Breure and Eskens 1981: 26.

####### Type locality.

”Venezuela”.

####### Label.

No locality label. M.C. label style III, V.

####### Dimensions.

Not given; figured specimen herein H 29.4, D 12.1, W 6.0.

####### Type material.

NHMUK 1975204, lectotype; 1975205, two paralectotypes (Cuming coll.).

####### Remarks.

The current systematic position follows Richardson (1995: 126).

####### Current systematic position.

Bulimulidae, *Drymaeus (Mesembrinus) feriatus* (Reeve, 1848).

###### Naesiotus (Maranhoniellus) fernandezae

Weyrauch, 1958

http://species-id.net/wiki/Naesiotus_fernandezae

Fig. 12H

Naesiotus (Maranhoniellus) fernandezae Weyrauch 1958: 122, pl. 9 figs 45–46; Breure 1979: 69; Neubert and Janssen 2004: 209, pl. 10 fig. 111; Breure 2012a: 8.

####### Type locality.

[Peru, Dept. Amazonas] ”Interandines Nord-Peru, auf rechter Seite des Río Marañon, bei Balsas, 850 m”.

####### Label.

”N-Peru: Balsas, Rio Marañon, 850 m, leg. B. Fernandez”, printed label.

####### Dimensions.

”H. 16.0 D. 3.4”; figured specimen herein H 15.1, D 3.3, W 8.1.

####### Type material.

NHMUK 19831, one paratype (ex Weyrauch).

####### Current systematic position.

Bulimulidae, *Naesiotus fernandezae* Weyrauch, 1958.

###### Bulimus ferrugineus

Reeve, 1849

http://species-id.net/wiki/Bulimus_ferrugineus

Figs 9D , L22v

Bulimus ferrugineus Reeve 1849 [1848–1850]: pl. 62 fig. 424; Pfeiffer 1853d: 416; Breure 1979: 53.
Bulimulus ferrugineus ; Pilsbry 1897 [1897–1898]: 29, pl. 9 fig. 37.
Bostryx ferrugineus ; Breure 1978: 77 (lectotype designation).

####### Type locality.

”Peru”.

####### Label.

”Peru”. M.C. label style V.

####### Dimensions.

Not given; figured specimen herein H 19.0, D 10.7, W 5.4.

####### Type material.

NHMUK 1975380, lectotype; 1975381, two paralectotypes (Cuming coll.).

####### Remarks.

All type material is subadult; the two paralectypes are relatively bigger, with the largest having a shell height of 26.4 mm. The current systematic position follows Richardson (1995: 25).

####### Current systematic position.

Bulimulidae, *Bostryx ferrugineus* (Reeve, 1849).

###### Bulimus fidustus

Reeve, 1849

http://species-id.net/wiki/Bulimus_fidustus

Figs 23G–H , L23i

Bulimus fidustus Reeve 1849 [1848–1850]: pl. 76 fig. 557; Breure 1979: 119 (lectotype designation).
Drymaeus fidustus ; Pilsbry 1898 [1897–1898]: 308, pl. 50 fig. 95; Linares and Vera 2012: 194.
Drymaeus (Mesembrinus) fidustus ; Breure and Eskens 1981: 69.

####### Type locality.

[Colombia, Dept. Putumayo / Ecuador, Prov. Napo] ”Sebundoi, New Granada”.

####### Label.

”Sebundoi / New Granada”. M.C. label style I, V.

####### Dimensions.

Not given; figured specimen herein H 22.5, D 11.4, W 6.8.

####### Type material.

NHMUK 1975517, lectotype; 1975518, one paralectotype (Cuming coll.).

####### Remarks.

The lip of the lectotype is slightly damaged.

####### Current systematic position.

Bulimulidae, *Drymaeus (Mesembrinus) fidustus* (Reeve, 1849).

###### Bulimus filaris

Pfeiffer, 1853

http://species-id.net/wiki/Bulimus_filaris

Figs 70C–D , 70L , L23ii

Bulimus filaris Pfeiffer 1853d: 653; Pfeiffer 1854e: 50; Breure 1979: 86.

####### Type locality.

Not given.

####### Label.

No locality given, taxon label in Pfeiffer’s handwriting. M.C. label style I.

####### Dimensions.

”Long 26, diam. 12 mill.”; figured specimen herein H 25.1, D 12.3, W 5.6.

####### Type material.

NHMUK 1975569, two syntypes (Cuming coll.).

####### Remarks.

Pfeiffer did not state on how many specimens his description was based. On the label a note is added by a later hand ”same as one of / Morelet”; it is unclear to us to what taxon this refers. The current systematic position at the species level follows Richardson (1995).

####### Current systematic position.

Bulimulidae, *Kuschelenia (Vermiculatus) filaris* (Pfeiffer, 1853) (**comb. n.**).

###### Bulimus (Liostracus) flavidulus

E.A. Smith, 1877

http://species-id.net/wiki/Bulimus_flavidulus

Figs 24A , L23iii

Bulimus (Liostracus) flavidulus E.A. Smith 1877: 364, pl. 39 fig. 3; Breure 1979: 119 (lectotype designation).
Drymaeus (Mesembrinus) flavidulus ; Breure and Eskens 1981: 70; Breure and Borrero 2008: 24.

####### Type locality.

[Ecuador, Prov. El Oro] ”Zaruma, South Ecuador”.

####### Label.

”Zaruma, S. Ecuador”, taxon label in Smith’s handwriting.

####### Dimensions.

”Long. 21 mill, diam. 9”; figured specimen herein H 21.4, D 9.4, W 7.1.

####### Type material.

NHMUK 1975134, lectotype; 1877.3.28.5, two paralectotypes.

####### Remarks.

The lip of the lectotype is slightly damaged.

####### Current systematic position.

Bulimulidae, *Drymaeus (Mesembrinus) flavidulus* (E.A. Smith, 1877).

###### Bulimus flexilabris

Pfeiffer, 1853

http://species-id.net/wiki/Bulimus_flexilabris

Figs 49J–L , L23iv

Bulimus flexilabris Pfeiffer 1853d: 652; Pfeiffer 1854e: 50; Breure 1979: 109 (lectotype designation).
Drymaeus flexilabris ; Simone 2006: 137, fig. 451.
Drymaeus (Drymaeus) bivittatus (Sowerby I, 1833); Breure and Eskens 1981: 10.

####### Type locality.

”in Brasilia”.

####### Label.

”Brazils”, taxon label in Pfeiffer’s handwriting. M.C. label style I.

####### Dimensions.

”Long. 28, diam. 12 1/2 mill.”; figured specimen herein H 27.1, D 13.8, W 7.1.

####### Type material.

NHMUK 1975559, lectotype (Cuming coll.).

####### Remarks.

This taxon is a junior subjective synonym of *Drymaeus bivittatus* (Sowerby I, 1833), of which the type material has not been traced in the NHMUK collection.

####### Current systematic position.

Bulimulidae, *Drymaeus (Drymaeus) bivittatus* (Sowerby I, 1833).

###### Bulimus flexuosus

Pfeiffer, 1853

http://species-id.net/wiki/Bulimus_flexuosus

Figs 34A–C , L23v

Bulimus flexuosus Pfeiffer 1853d: 329; Pfeiffer 1854b: 136; Pfeiffer 1854 in Küster and Pfeiffer 1840–1865: 244, pl. 65 figs 6–7; Breure 1979: 109 (lectotype designation).
Drymaeus flexuosus ; Linares and Vera 2012: 186 [partim].
Drymaeus (Drymaeus) flexuosus ; Breure and Eskens 1981: 26, pl. 5 fig. 3.

####### Type locality.

[Colombia, Dept. Caldas] ”Marinato [sic, Marmato] Novae Granadae”.

####### Label.

”Marinata / New Grenada”, taxon label in Pfeiffer’s handwriting. M.C. label style III.

####### Dimensions.

”Long. 40, diam. 14 mill.”; figured specimen herein H 43.0, D 20.0, W 7.2.

####### Type material.

NHMUK 1975202, lectotype; 1975203, two paralectotypes (Cuming coll.).

####### Remarks.

This taxon is restricted to the Central Cordillera in Colombia and has partly been confused with other *Drymaeus* species (Breure and Borrero, unpublished data).

####### Current systematic position.

Bulimulidae, *Drymaeus (Drymaeus) flexuosus* (Pfeiffer, 1853).

###### Bulimus floridanus

Pfeiffer, 1857

http://species-id.net/wiki/Bulimus_floridanus

Figs 19H , L24i

Bulimus floridanus Pfeiffer 1857c: 330; Breure 1979: 119 (lectotype designation).
Drymaeus (Mesembrinus) dominicus (Reeve)[sic]; Breure and Eskens 1981: 55.

####### Type locality.

[U.S.A.] ”Florida”.

####### Label.

”Florida”, taxon label in Pfeiffer’s handwriting. M.C. label style III.

####### Dimensions.

”Long. 15 2/3–17, diam. 7 1/2 mill.”; figured specimen herein H 16.7, D 8.0, W 6.2.

####### Type material.

NHMUK 1975199, lectotype; 1975200, one paralectotype (Cuming coll.).

####### Remarks.

This taxon was considered by Richardson (1995: 190) as a junior subjective synonym of *Drymaeus umbraticus* (Reeve, 1850), of which the type material also is present in the NHMUK collection (see page 201); this classification is here followed.

####### Current systematic position.

Bulimulidae, *Drymaeus (Mesembrinus) umbraticus* (Reeve, 1850).

###### Bulimus fontainii

d’Orbigny, 1838

http://species-id.net/wiki/Bulimus_fontainii

Figs 16H , L24ii

Bulimus fontainii d’Orbigny 1838 [1834–1847]: 273.
Bulimulus fontainii ; Breure and Borrero 2008: 10.

####### Type locality.

[Ecuador, Prov. Guayas] ”environs de Guayaquil”.

####### Label.

”Guayaquil”, label in d’Orbigny’s handwriting.

####### Dimensions.

”Longueur totale, 13 millimètres; largeur 6 millimètres”; figured specimen herein H 13.2, D 6.4, W 8.5.

####### Type material.

NHMUK 1854.12.4.165, lectotype (d’Orbigny coll.).

####### Remarks.

d’Orbigny did not state on how many specimens his description was based. The single specimen found in his collection matches the dimensions given, and is here designated lectotype (**design. n.**). The weak axial sculpture of the protoconch makes us considering this taxon to belong to *Naesiotus* sensu Breure 1979, awaiting further studies of this group.

####### Current systematic position.

Bulimulidae, *Naesiotus fontainii* (d’Orbigny, 1838) (**comb. n.**).

###### Bulimus fourmiersi

d’Orbigny, 1837

http://species-id.net/wiki/Bulimus_fourmiersi

Figs 16I , L24iii

Bulimus fourmiersi d’Orbigny 1837 [1834–1847]: 273, pl. 30 figs 12–14 [3 April 1837; text 23 April 1838].

####### Type locality.

[Argentina] ”la province de Corrientes, non loin du Rio de Santa-Lucia, au lieu nommé *Pasto reito*”; see Breure 1973: 133.

####### Label.

”corrientes Rep. argentina”, in d’Orbigny’s handwriting.

####### Dimensions.

”Long. 11 millim., lat. 7 millim.”; see remarks.

####### Type material.

NHMUK 1854.12.4.210, lectotype (d’Orbigny coll.).

####### Remarks.

The single specimen is severely damaged, and only the upper whorls are retained. The original figures of d’Orbigny, which are heirein designated as lectotype (**design. n.**), are figured instead of the type specimen. The protoconch scultpture suggests this taxon to belong to *Naesiotus*; more detailed studies are needed to corraborate this classification. According to the collation data the figures were published in 1837, the text in 1838 (Coan et al. 2013a). Miquel (1989a: 2) considered it as ”sp. inquirenda”; Cuezzo et al. (2013: 151) classified it with *Bulimulus* Leach, 1814.

####### Current systematic position.

Bulimulidae, *Naesiotus fourmiersi* (d’Orbigny, 1837).

###### Bulimus fucatus

Reeve, 1849

http://species-id.net/wiki/Bulimus_fucatus

Figs 45G–I , L24v

Bulimus fucatus Reeve 1849 [1848–1850]: pl. 83 fig. 615; Breure 1979: 109 (lectotype designation).
Drymaeus fucatus ; Pilsbry 1898 [1897–1898]: 234, pl. 42 fig. 63; Breure and Borrero 2008: 21; Linares and Vera 2012: 187.

####### Type locality.

[Colombia] ”Sebundoi, New Granada”.

####### Label.

”New Granada”.

####### Dimensions.

Not given; figured specimen herein H 23.5, D 11.1, W 6.2.

####### Type material.

NHMUK 1874.12.11.224, lectotype, ex Mrs. T. Lombe-Taylor.

####### Remarks.

Reeve did not state on how many specimens his description was based. The distribution of this species in Colombia needs further evidence (Breure and Borrero, unpublished data).

####### Current systematic position.

Bulimulidae, *Drymaeus (Drymaeus) fucatus* (Reeve, 1849).

###### Bulimus (Liostracus) fuscobasis

E.A. Smith, 1877

http://species-id.net/wiki/Bulimus_fuscobasis

Figs 24B , L24vi

Bulimus (Liostracus) fuscobasis E.A. Smith 1877: 365, pl. 39 fig. 6; Breure 1979: 119 (lectotype designation).
Drymaeus (Mesembrinus) fuscobasis (E.A. Smith); Breure and Eskens 1981: 70.

####### Type locality.

”Tarapoto, Andes of Peru, Mr. Spruce (Mus. Cuming)”.

####### Label.

”Tarapoto, Andes of Peru / Mr. Spruce”; taxon label in Smith’s handwriting. M.C. label style III.

####### Dimensions.

”Longit. 29 mill., diam. 12”; figured specimen herein H 28.7, D 12.3, W 8.2.

####### Type material.

NHMUK 1975139, lectotype; 1975140, one paralectotype, Spruce leg. (Cuming coll.).

####### Remarks.

The specimen selected lectotype by Breure (1979) is marked with a red dot. The specimens were accompanied by a label ”holotype”; however, the original label clearly states ”types” and it is not clear whom has attached the (subsequent?) label ”holotype”. Smith described the species from ”two specimens”.

####### Current systematic position.

Bulimulidae, *Drymaeus (Mesembrinus) fuscobasis* (E.A. Smith, 1877).

###### Helix fusoides

d’Orbigny, 1835

http://species-id.net/wiki/Helix_fusoides

Figs 29D–F , L24iv

Helix fusoides d’Orbigny 1835: 19.
Bulimus fusoides d’Orbigny 1837 [1834–1847]: 315, pl. 40 figs 12–13 [18 Sept. 1837; text 6 May 1838].
Drymaeus fusoides ; Pilsbry 1898 [1897–1898]: 201, pl. 38 figs 17–18.

####### Type locality.

”provincia Yungacensi (republica Boliviana)”

####### Label.

”Yunga..... olivia”; in d’Orbigny’s handwriting.

####### Dimensions.

”Longit. 4 centim., latit. 1 centim. 4 millim.” (corrected to ”Long. 40 millim., lat. 15 millim.” in d’Orbigny 1837); figured specimen herein H 24.6, D —, W 5.4.

####### Type material.

NHMUK 1854.12.4.133, one paralectotype.

####### Remarks.

d’Orbigny did not state on how many specimens his description was based. The type locality was specified in d’Orbigny 1838 [1834-1847]: 316 as ”près du lieu dit Yunga de la Palma” (see Breure 1973: 117, 132). Breure (1975: 1151) has selected a lectotype from the material found in MNHN. The specimen in London has lost the lower part of the last whorl and moreover does not correspond to d’Orbigny 1837 [1834-1847]: pl. 40 figs 12-13. The current systematic position follows Richardson (1995: 128).

####### Current systematic position.

Bulimulidae, *Drymaeus (Drymaeus) fusoides* (d’Orbigny, 1835).

###### Bulimus gabbi

Angas, 1879

http://species-id.net/wiki/Bulimus_gabbi

Figs 24L , L24vii

Bulimus gabbi Angas 1879: 477, pl. 40 fig. 3; Breure 1979: 119 (lectotype designation).
Drymaeus gabbi ; Pilsbry 1899: 70, pl. 6 figs 7–9, 11.
Drymaeus (Mesembrinus) gabbi ; Thompson 2011: 117.

####### Type locality.

[Costa Rica, Prov. Limón, Cerro Kámuk] ”flanks of Pico Blanco, at an altitude from 3000 to 6000 feet [914–1828 m]”.

####### Label.

”flanks / of Pico Blanco / 3–6000 ft / Costa Rica”.

####### Dimensions.

”Diam. 7, alt. 10 1/2 lin. [H 22.2, D 14.8 mm]”; figured specimen herein H 20.6, D 12.06, W 4.7.

####### Type material.

NHMUK 1879.7.22.23, lectotype; 1879.7.22.24–26, three paralectotypes, Gabb leg. (ex Angas).

####### Remarks.

Angas did not state on how many specimens his description was based; he distinguished three varieties, which differ in their colour pattern. The lectotype corresponds to Angas’ right-hand sided figure. The current systematic position follows Thompson (2011).

####### Current systematic position.

Bulimulidae, *Drymaeus (Mesembrinus) gabbi* (Angas, 1879).

###### Bulimus galapaganus

Pfeiffer, 1855

http://species-id.net/wiki/Bulimus_galapaganus

Figs 14H , L24viii

Bulimus galapaganus Pfeiffer 1855a: 58; Pfeiffer 1859b: 503; Breure 1979: 69 (lectotype designation).

####### Type locality.

[Ecuador] ”Galapagos Islands”.

####### Label.

”Galapagos Islds”, taxon label in Pfeiffer’s handwriting. M.C. label style III.

####### Dimensions.

”Long. 15 1/2, diam. 6 mill.”; figured specimen herein H 15.1, D 6.28, W 7.6.

####### Type material.

NHMUK 1975146, lectotype; 1975147, one paralectotype (Cuming coll.).

####### Remarks.

The current systematic position follows Richardson (1995: 220).

####### Current systematic position.

Bulimulidae, *Naesiotus galapaganus* (Pfeiffer, 1855).

###### Bulimus gayi

Pfeiffer, 1857

http://species-id.net/wiki/Bulimus_gayi

Figs 67E–F , 67M , L25i

Bulimus gayi Pfeiffer 1857d: 389; Breure 1979: 88.
Scutalus (Kuschelenia) gayi ; Breure 1978: 177 (lectotype designation).

####### Type locality.

”Bolivia”.

####### Label.

”Bolivia”, taxon label in Pfeiffer’s handwriting. M.C. label style IV.

####### Dimensions.

”Long. 27, diam. 16 mill.”; figured specimen herein H 27.7, D 16.2, W 5.8.

####### Type material.

NHMUK 1975382, lectotype; 1975383, one paralectotype (Cuming coll.).

####### Remarks.

This taxon is now transferred to the genus *Kuschelenia* Hylton Scott, 1952.

####### Current systematic position.

Bulimulidae, *Kuschelenia (Kuschelenia) gayi* (Pfeiffer, 1857) (**comb. n.**).

###### Bulimus (Mesembrinus) gealei

H. Adams, 1867

http://species-id.net/wiki/Bulimus_gealei

Figs 51G–I , L25iii

Bulimus (Mesembrinus) gealei H. Adams 1867b: 309, pl. 19 fig. 21.

####### Type locality.

”Mexico. Collected by Mr. Boucard”.

####### Label.

”Mexico”.

####### Dimensions.

”Long. 29, diam. 14 mill.”; figured specimen herein H 26.4, D 13.4, W 6+.

####### Type material.

NHMUK 1867.1.9.20, lectotype and two paralectotypes; 1867.1.9.21, three paralectotypes, ex Geale.

####### Remarks.

One of the specimens corresponds to Adams 1867b: pl. 19 fig. 21 and is here selected lectotype (**design. n.**) to fixate this taxon; it has the top whorls slightly damaged. According to the register the specimens were purchased from Mr R. Geale; the label does not mention the name Boucard. Köhler (2007: 145, fig. 90) selected a lectotype (ZMB 4457a) for the senior subjective synonym *Bulimulus (Scutalus) fenestrellus* Martens, 1864.

####### Current systematic position.

Bulimulidae, *Drymaeus (Drymaeus) fenestrellus* (Martens, 1864).

###### Bulimus gelidus

Reeve, 1849

http://species-id.net/wiki/Bulimus_gelidus

Figs 62C–D , L25ii

Bulimus gelidus Reeve 1849 [1848–1850]: pl. 76 fig. 555; Breure 1978: 142; Breure 1979: 62 (lectotype designation); Miquel 1991: 98, fig. 25.
Bulimulus gelidus ; Breure 2008: 245, figs 6–7 [not 8]; see remarks.

####### Type locality.

”Central America”.

####### Label.

”C. America”. M.C. label style I, V.

####### Dimensions.

Not given; figured specimen herein H 32.5, D 15.0, W 7.7.

####### Type material.

NHMUK 1975402, lectotype (Cuming coll.).

####### Remarks.

On the back of the cardboard a handwriting in pencil has noted ”seems to be the same as monte-vidensis Pfr.”. Miquel (1991) placed this taxon is the synonymy of *Syphalomphix bonariensis* Rafinesque, 1833, a species from Argentina. However, it must be noted that most *Bulimulus* species are very similar and often hard to tell apart. Reconsidering the identification made in Breure (2008), we now disagree with him, as the Ecuadorian shells have different dimensions, and the last whorl is less globose than in the lectotype of *Bulimus gelidus*. The locality on the label may be erroneous, and Reeve’s taxon is herein considered as nomen inquirendum.

####### Current systematic position.

Bulimulidae, *Bulimulus*. Nomen inquirendum.

###### Bulimus geometricus

Pfeiffer, 1846

http://species-id.net/wiki/Bulimus_geometricus

Figs 46J–L , L25iv

Bulimus geometricus Pfeiffer 1846c: 84; Pfeiffer 1848b: 59; Reeve 1848 [1848–1850]: pl. 44 fig. 278; Breure 1979: 109 (lectotype designation).
Drymaeus geometricus ; Linares and Vera 2012: 187.
Drymaeus (Drymaeus) geometricus ; Breure and Eskens 1981: 27, pl. 8 fig. 2.

####### Type locality.

[Colombia] ”Vallis Magdalenae Novae Granadae”.

####### Label.

”Valley of the Madelein / New Granada”, taxon label in Pfeiffer’s handwriting. M.C. label style IV, V.

####### Dimensions.

”Long. 35, diam. 13 mill.”; figured specimen herein H 34.6, D 18.0, W 6.9.

####### Type material.

NHMUK 1975564, lectotype; 1975565, two paralectotypes (Cuming coll.).

####### Current systematic position.

Bulimulidae, *Drymaeus (Drymaeus) geometricus* (Pfeiffer, 1846).

###### Bostryx (Bostryx) zilchi glomeratus

Weyrauch, 1960

http://species-id.net/wiki/Bostryx_zilchi_glomeratus

Fig. 3I

Bostryx (Bostryx) zilchi glomeratus Weyrauch 1960b: 124, pl. 12 figs 29–34; Neubert and Janssen 2004: 210, pl. 3 fig. 35; Breure 2012a: 8.

####### Type locality.

”Mittel-Peru: Quichao, 5 km von Laraos, am Fusspfad nach Yauyos, im Tale des Río Cañete und auf der linken Seite des Río Mayo, 3500 m”.

####### Label.

”C-Peru, Quichao, 5 kms. from Laraos, valley of Río Cañete, 3500 m”; printed label.

####### Dimensions.

”H. 19.5, D. 6.5”; figured specimen herein H 18.6, D 6.1, W 7.7.

####### Type material.

NHMUK 1975154, five paratypes, ex Weyrauch (WW 3320).

####### Current systematic position.

Bulimulidae, *Bostryx zilchi* Weyrauch, 1958.

###### Scutalus grandiventris

Weyrauch, 1960

http://species-id.net/wiki/Scutalus_grandiventris

Fig. 65H

Scutalus grandiventris Weyrauch, 1960a: 42, pl. 5 figs 27–33; Neubert and Janssen 2004: 211, pl. 12 fig. 140; Breure 2012a: 8.

####### Type locality.

”N-Peru, am Westhang der westlichen Anden, oberhalb Cascas ± 100 km nö. Trujillo und Cajamarca (1400 m)”.

####### Label.

”N-Peru: Cascas, 1400 m”; printed label.

####### Dimensions.

”H. 53.0, D. 29.7”; figured specimen herein H 54.2 D 29.1 W 7.0.

####### Type material.

NHMUK 1975386, two paratypes, ex Weyrauch (WW 1364).

####### Current systematic position.

Bulimulidae, *Scutalus grandiventris* Weyrauch, 1960.

###### Bulimus gruneri

Pfeiffer, 1846

http://species-id.net/wiki/Bulimus_gruneri

Figs 24E , L25vii

Bulimus gruneri Pfeiffer 1846a: 30; Pfeiffer 1848b: 213; Reeve 1849 [1848–1850]: pl. 51 fig. 332.

####### Type locality.

”Mexico”.

####### Label.

”Mexico”, added in a later handwriting. M.C. label style I, V.

####### Dimensions.

”Long. 28, diam. 10 mill.”; figured specimen herein H 27.8, D 10.5, W 7.3.

####### Type material.

NHMUK 20100563/1, lectotype; NHMUK 20100563/2–3, two paralectotypes; NHMUK 20100562, three paralectotypes (Cuming coll.).

####### Remarks.

The quotation by Richardson (1995: 196) of 1842 as the year of publication of *Bulimus gruneri* appears erroneous, as Pfeiffer does not mention this taxon until his 1846 paper. Two lots have been traced, one of which contains a specimen closest matching the original dimensions; this specimen is here designated lectotype (**design. n.**) to define the taxon. The other lot contains one specimen resembling Reeve 1848 [1848–1850]: pl. 51 fig. 332. The locality ”Mexico” seems in error, as this taxon has been recognised as a Colombian species (Breure and Borrero, unpublished data) and is now considered as a junior subjective synonym of *Bulimus columbianus* Lea, 1838 (**syn. n.**).

####### Current systematic position.

Bulimulidae, *Drymaeus (Mesembrinus) columbianus* (Lea, 1838).

###### Bulimus gueinzii

Pfeiffer, 1857

http://species-id.net/wiki/Bulimus_gueinzii

Figs 30M–O , L25vi

Bulimus gueinzii Pfeiffer 1857c: 330; Breure 1979: 109 (lectotype designation).
Drymaeus (Drymaeus) gueinzii ; Breure and Eskens 1981: 27, pl. 6 fig. 8.

####### Type locality.

”Meobamba, Peru (*Mr Gueinzius*)”.

####### Label.

”Meobamba”, taxon label in Pfeiffer’s handwriting. M.C. label style I.

####### Dimensions.

”Long. 23, diam. 10 mill.”; figured specimen herein H 23.2, D 10.9, W 6.0.

####### Type material.

NHMUK 1975539, lectotype, Gueinzius leg. (Cuming coll.).

####### Remarks.

The current systematic position follows Richardson (1995: 134).

####### Current systematic position.

Bulimulidae, *Drymaeus (Drymaeus) gueinzii* (Pfeiffer, 1857).

###### Bulinus guttatus

Broderip, 1832

http://species-id.net/wiki/Bulinus_guttatus

Figs 7E , L26i

Bulinus guttatus Broderip in Broderip and Sowerby I 1832a: 31.
Bulimus guttatus ; Reeve 1848 [1848–1850]: pl. 22 fig. 144.

####### Type locality.

[Chile] ”Peruviâ. (Cobija or Puerto De la Mar)”.

####### Label.

”Bolivia, S. America”, added in a later handwriting. M.C. label style III, V.

####### Dimensions.

”long. 7/8, lat. 3/8 poll. [H 22.1, D 9.5 mm]”; figured specimen herein H 22.0, D —, W 6.2.

####### Type material.

NHMUK 20110175, five possible syntypes (Cuming coll.).

####### Remarks.

The type locality is now part of Chile, but during the 19^th^ century has also been part of Bolivia and Peru. This taxon has been treated as nomen inquirendum by Breure (1979). Of the five specimens, two are subadult or juvenile. One specimen corresponds to Reeve 1848 [1848–1850]: pl. 22 fig. 144, and is damaged both at the lip and on the dorsal side of the last whorl.

####### Current systematic position.

Bulimulidae, *Bostryx guttatus* (Broderip in Broderip and Sowerby I 1832).

###### Bulimus hachensis

Reeve, 1850

http://species-id.net/wiki/Bulimus_hachensis

Figs 19K–L , L26ii

Bulimus hachensis Reeve 1850 [1848–1850]: pl. 85 fig. 627; Pfeiffer 1853d: 421; Breure 1979: 119 (lectotype designation).
Drymaeus hachensis ; Pilsbry 1899: 90, pl. 12 fig. 20; Linares and Vera 2012: 195.
Drymaeus (Mesembrinus) hachensis ; Breure and Eskens 1981: 71.

####### Type locality.

”Guatemala, banks of the Rio Hacha”.

####### Label.

”Rio Hacha, Guatemala”. M.C. label style I, V.

####### Dimensions.

Not given; figured specimen herein H 26.4, D 11.3, W 7.7.

####### Type material.

NHMUK 1975392, lectotype (Cuming coll.).

####### Remarks.

Reeve did not state on how many specimens his description was based. We consider this taxon to be a junior subjective synonym of *Bulimus columbianus* Lea, 1838 (**syn. n.**), which is partly based on unpublished data of Breure and Borrero.

####### Current systematic position.

Bulimulidae, *Drymaeus (Mesembrinus) columbianus* (Lea, 1838).

###### Bulimus hamiltoni

Reeve, 1849

http://species-id.net/wiki/Bulimus_hamiltoni

Figs 1F , L26iii

Bulimus hamiltoni Reeve 1849 [1848–1850]: pl. 83 fig. 610; Pfeiffer 1853d: 429.
Bulimulus (Peronaeus) hamiltoni ; Pilsbry 1896 [1895–1896]: 149, pl. 46 fig. 51.
Bostryx hamiltoni ; Breure 1978: 80 (lectotype designation).

####### Type locality.

”Near the Lake of Titicaca, Bolivia”.

####### Label.

”Bolivia”. A second label indicates ”appartient [?] à Mr Pentlan[d]” [belongs to Mr. Pentland] and refers to the figure published in Reeve 1848–1850.

####### Dimensions.

Not given; figured specimen herein H 17.0, D 6.55, W 8.0.

####### Type material.

NHMUK 1849.5.14.53, lectotype; 1849.5.14.54–57, four paralectotypes.

####### Remarks.

According to Reeve the material was collected by Mr. Pentland and belonged to ”Mus. Hamilton”; the lectotype corresponds to Reeve 1849 [1848–1850]: pl. 83 fig. 610. Breure (1978) erroneously mentioned the lectotype as 1975329 and the paralectotypes as 1975330. The current systematic position follows Richardson (1995: 26).

####### Current systematic position.

Bulimulidae, *Bostryx hamiltoni* (Reeve, 1849).

###### Bulimus haplochrous

Pfeiffer, 1855

http://species-id.net/wiki/Bulimus_haplochrous

Figs 61G–H , L26iv

Bulimus haplochrous Pfeiffer 1855g: 125; Pfeiffer 1859b: 502; Breure 1979: 63.
Bulimulus haplochrous ; Breure 1978: 144 (lectotype designation).

####### Type locality.

”—?”.

####### Label.

”New Granada”, taxon label in Pfeiffer’s handwriting. M.C. label style IV.

####### Dimensions.

”Long. 30, diam. 12 mill.”; figured specimen herein H 28.6, D 13.3, W 5.3.

####### Type material.

NHMUK 1975405, lectotype; 1975406, two paralectotypes (Cuming coll.).

####### Remarks.

Pfeiffer did not state on how many specimens his description was based. The current systematic position follows Richardson (1995: 76).

####### Current systematic position.

Bulimulidae, *Bulimulus haplochrous* (Pfeiffer, 1855).

###### Helix heloica

d’Orbigny, 1835

http://species-id.net/wiki/Helix_heloica

Figs 62E–F , L26v

Helix heloica d’Orbigny 1835: 11.
Bulimus heloicus ; d’Orbigny 1837 [1834–1847]: 272, pl. 30 figs 9–11 [3 April 1837; text 23 April 1838].
Bulimulus (Lissoacme) heloicus ; Pilsbry 1896 [1895–1896]: 193, pl. 51 figs 12–13.

####### Type locality.

[Bolivia] ”provincia Chiquitensi, republica Boliviana”.

####### Label.

”Sn Xavier de Chiquitos Bolivia”, label in d’Orbigny’s handwriting.

####### Dimensions.

”Longit. 28 millim., diam. 6 millim.”; figured specimen herein H 25.6, D 12.0, W 6.8.

####### Type material.

NHMUK 1854.12.4.164, five syntypes (d’Orbigny coll.).

####### Remarks.

d’Orbigny (1835) did not state on how many specimens his description was based; the type locality was specified in d’Orbigny 1838 [1834–1847]: 273 as ”près de la Mission de Bibosi, province de Santa-Cruz de la Sierra, et dans la partie orientaler de l’immense fôret (Monte grande) qui sépare Santa-Cruz de la province de Chiquitos, non loin du lieu nommé Potrero de la Cruz”. The locality on the label probably refers to San Javier, Dept. Santa Cruz in Bolivia; see Breure 1973: 119, 127. The protoconch sculpture suggests that this taxon belongs to *Bulimulus* Leach, 1814.

####### Current systematic position.

Bulimulidae, *Bulimulus heloicus* (d’Orbigny, 1835) (**comb. n.**).

###### Bulimus hepatostomus

Pfeiffer, 1861

http://species-id.net/wiki/Bulimus_hepatostomus

Figs 17D–E , L26vii

Bulimus hepatostomus Pfeiffer 1861: 23, pl. 3 fig. 4; Breure 1979: 110 (lectotype designation).
Drymaeus (Drymaeus) hepatostomus ; Breure and Eskens 1981: 28, pl. 8 fig. 10.
Drymaeus (Mesembrinus) hepatostomus ; Thompson 2011: 115.

####### Type locality.

”Mexico (*Mr Boucard*)”.

####### Label.

”Tepenistlahuaca / Mexico. M^r^ Boucard”, taxon label in Pfeiffer’s handwriting. M.C. label style IV.

####### Dimensions.

”Long. 32, diam. 13 mill.”; figured specimen herein H 30.1, D 15.0, W 6.1.

####### Type material.

NHMUK 1975571, lectotype; 1975221, one paralectotype, Boucard leg. (Cuming coll.).

####### Remarks.

The current systematic position is according to Thompson (2011).

####### Current systematic position.

Bulimulidae, *Drymaeus (Mesembrinus) hepatostomus* (Pfeiffer, 1861).

###### Bulimulus (Drymaeus) hidalgoi

da Costa, 1898

http://species-id.net/wiki/Bulimulus_hidalgoi

Figs 32A–C , L26vi

Bulimulus (Drymaeus) hidalgoi da Costa, 1898: 80, pl. 6 fig. 2; Breure 1979: 110.
Drymaeus (Drymaeus) hidalgoi ; Breure and Borrero 2008: 21.

####### Type locality.

”Ecuador”.

####### Label.

”Equador”, label in da Costa’s handwriting.

####### Dimensions.

”Long. 39, diam. 13 mm.”; figured specimen herein H 39.0, D 17.0, W 6.6.

####### Type material.

NHMUK 1907.11.21.28, lectotype; 1907.11.21.29–30, two paralectotypes, Buckley leg. (da Costa coll.).

####### Remarks.

da Costa did not state on how many specimens his description was based, only that he had ”examples collected by the late Mr. Buckley”. The reference of Breure (1979) to ”HT 1907.11.21.28” has to be interpreted as a lectotype designation under Art. 74.6 ICZN.

####### Current systematic position.

Bulimulidae, *Drymaeus (Drymaeus) hidalgoi* (da Costa, 1898).

###### Bulimus holostoma

Pfeiffer, 1846

http://species-id.net/wiki/Bulimus_holostoma

Figs 2O–P , L26viii

Bulimus holostoma Pfeiffer 1846a: 28; Pfeiffer 1848b: 161; Reeve 1848 [1848–1850]: pl. 69 fig. 490; Breure 1979: 54 (lectotype designation).

####### Type locality.

[Chile] ”Cobija, Bolivia”.

####### Label.

”Cobija Bolivia Hills under Bushes”, taxon label in Pfeiffer’s handwriting. M.C. label style IV, V.

####### Dimensions.

”Long. 9, diam. 2 2/3 mill.”; figured specimen herein H 7.84, D 2.43, W 5.9.

####### Type material.

NHMUK 1975345, lectotype; 1975346, one paralectotype (Cuming coll.).

####### Remarks.

Pfeiffer described this species from the Cuming collection, but did not state on how many specimens his description was based. The current systematic position follows Richardson (1995: 27).

####### Current systematic position.

Bulimulidae, *Bostryx holostoma* (Pfeiffer, 1846).

###### Bulimus hondurasanus

Pfeiffer, 1846

http://species-id.net/wiki/Bulimus_hondurasanus

Figs 24I , L27i

Bulimus hondurasanus Pfeiffer 1846a: 29; Reeve 1849 [1848–1850]: pl. 59 fig. 400; Breure 1979: 119 (lectotype designation).
Drymaeus hondarusanus ; Rehder 1966: 286, figs 5–6.
Drymaeus (Mesembrinus) hondurasanus ; Thompson (2011: 121).

####### Type locality.

”Honduras”.

####### Label.

”Honduras on leaves / of Bushes Dyson”, taxon label in Pfeiffer’s handwriting. M.C. label style IV, V.

####### Dimensions.

”Long. 18 1/2, diam. 10 mill.”; figured specimen herein H 28.6, D 13.7, W 6.6.

####### Type material.

NHMUK 1975265, lectotype; 1975117, one paralectotype, Dyson leg. (Cuming coll.).

####### Remarks.

Pfeiffer (1846a: 30) mentioned that his material originated from Dyson, which is confirmed by the information on the label. Therefore there is no doubt about the type status of the specimens. However, Pfeiffer’s dimensions (H/D ration 1.85) do not fit the specimens found in the NHMUK collection (H/D ratio 2.08). The figure as presented by Reeve 1849 [1848–1850] confirms the latter ratio, which makes it likely that Pfeiffer’s measurements are either erroneous or that he had more material in his own collection. As the Pfeiffer collection must considered to be lost (Dance 1966), the London specimens are the sole reference. We consider it probable that the published dimensions of Pfeiffer are in error.

####### Current systematic position.

Bulimulidae, *Drymaeus (Mesembrinus) hondurasanus* (Pfeiffer, 1846).

###### Bulimus huascensis

Reeve, 1848

http://species-id.net/wiki/Bulimus_huascensis

Figs 8B , L27ii

Bulimus huascensis Reeve 1848 [1848–1850]: pl. 23 fig. 147; Breure 1979: 54.
Bostryx huascensis ; Breure 1978: 90 (lectotype designation).

####### Type locality.

[Chile] ”Huasco, Chili”.

####### Label.

”Chili”. M.C. label style III, IV, V.

####### Dimensions.

Not given; figured specimen herein H 19.1, D 10.9, W 6.0.

####### Type material.

NHMUK 1975159, lectotype; 1975160, four paralectotypes (Cuming coll.).

####### Remarks.

The current systematic position follows Richardson (1995: 28).

####### Current systematic position.

Bulimulidae, *Bostryx huascensis* (Reeve, 1848).

###### Bulimus humboldtii

Reeve, 1849

http://species-id.net/wiki/Bulimus_humboldtii

Figs 37D–E , L27iii

Bulimus humboldtii Reeve 1849 [1848–1850]: pl. 58 fig. 391; Breure 1979: 110.

####### Type locality.

”Mexico”.

####### Label.

”Mexico”. M.C. label style IV, V.

####### Dimensions.

Not given; figured specimen herein H 27.6, D 14.5, W 6.6.

####### Type material.

NHMUK 1975528, lectotype and one paralectotype (Cuming coll.).

####### Remarks.

The specimen corresponding to Reeve’s figure is selected lectotype (**design. n.**) to define this taxon, for which Reeve did not state on how many specimens his description was based. This taxon is considered a junior subjective synonym of *Bulimus mexicanus* Lamarck, 1822 by Richardson (1995: 150), which is a Peruvian species.

####### Current systematic position.

Bulimulidae, *Drymaeus (Drymaeus) mexicanus* (Lamarck, 1822).

###### Helix hygrohylaea

d’Orbigny, 1835

http://species-id.net/wiki/Helix_hygrohylaea

Figs 27D–F , L27iv

Helix hygrohylaea d’Orbigny 1835: 18.
Bulimus hygrohylaeus d’Orbigny 1837 [1834–1847]: 311, pl. 40 figs 3–5 [18 Sept. 1837; text 6 May 1838]; Gray 1854: 21.
Drymaeus hygrohylaeus ; Pilsbry 1898 [1897–1898]: 194, pl. 37 figs 9–10; Breure 1975b: 1151; Cuezzo et al. 2013: 154.
Drymaeus (Drymaeus) hygrohylaeus ; Miquel 1989b: 77, figs 3–4.

####### Type locality.

”provincia Chiquitensi (republica Boliviana)”.

####### Label.

Three labels, ”S^ta^ Cruz de la sierra, Bolivia” [.126], ”guarayos, Bolivia” [.127], ”chiquitos, Bolivia” [.128]; all in d’Orbigny’s handwriting.

####### Dimensions.

”Longit. 41 millim., latit. 19 millim.”; figured specimen herein H 40.4, D 19, W 6.7.

####### Type material.

NHMUK 1854.12.4.127, lectotype and five paralectotypes; 1854.12.4.126, four paralectotypes; 1854.12.4.128, two paralectotypes.

####### Remarks.

The specimen that corresponds to the original figure of d’Orbigny 1837 [1834–1847], is now chosen as lectotype (**design. n.**) to fixate this taxon. The locality ”guarayos” probably refers to Guarayos Province, in the northwestern part of Santa Cruz Department; see also Breure 1973: 117. It is here restricted as type locality. There are two additional paralectotypes in Paris (Breure 1975b: 1151). The current systematic position follows the synonymisation of this taxon with *Helix abyssorum* d’Orbigny, 1835, by Miquel (1989b: 77); this author, however, overlooked that the latter name has page priority. Cuezzo et al. (2013), however, disagreed with Miquel’s classification; this is here tentatively retained awaiting further studies on the position of both taxa.

####### Current systematic position.

Bulimulidae, *Drymaeus (Drymaeus) abyssorum* (d’Orbigny, 1835).

###### Otostomus emeus hypozonus

Martens, 1893

http://species-id.net/wiki/Otostomus_emeus_hypozonus

Figs 23E , L27v

Bulimulus palpaloensis Strebel in Strebel and Pfeffer 1882: 85, pl. 5 figs 12d, 16.
Otostomus emeus var. *hypozonus*Martens 1893 [1890–1901]: 223.
Drymaeus (Mesembrinus) hypozonus ; Köhler 2007: 150, fig. 116 (lectotype designation).

####### Type locality.

”East Mexico”.

####### Label.

”E. Mexico”, in Martens handwriting.

####### Dimensions.

Not given; figured specimen herein H 19.3, D 11.3, W 5.7.

####### Type material.

NHMUK 1901.6.22.794–795, two paralectotypes, H. Strebel leg. (Godman coll.).

####### Remarks.

Strebel gave the measurements of nearly 30 specimens in his description of *Bulimulus palpaloensis*, but did not mention the material as presented by Martens—who remarked on the label ”*Otostomus palpaloensis* Streb /.../ collected by Strebel”. From their remarks (Strebel and Pfeffer 1882: 86) it is likely that Strebel collected the material himself (see also Martens 1893 [1890–1901]: 223). The specimens found are evidently material studied by Martens and described as his variety *hypozonus*; this variety is based on Strebel and Pfeffer 1882: pl. 5 figs 12d, 16. No further evidence is given in this paper on which specimens these figures are based. The suggestion in Köhler (2007) that Martens based this taxon on ZMB material is questionable, as Martens 1890–1901 was entirely based on the Godman collection which is now in NHMUK. The current systematic position is according to Thompson (2011).

####### Current systematic position.

Bulimulidae, *Drymaeus (Mesembrinus) emeus* (Say, 1830).

###### Bulimus ignavus

Reeve, 1849

http://species-id.net/wiki/Bulimus_ignavus

Figs 62I–J , L27vi

Bulimus ignavus Reeve 1849 [1848–1850]: pl. 77 fig. 562; Breure 1979: 63.
Bulimulus unicolor unicolor (Sowerby I, 1833); Breure 1978: 147 (lectotype designation).

####### Type locality.

”Central America”.

####### Label.

”Central America”. M.C. label style I, V.

####### Dimensions.

Not given; figured specimen herein H 17.1 (see remarks).

####### Type material.

NHMUK 1975411, lectotype (Cuming coll.).

####### Remarks.

The last whorl of the specimen is very damaged. The current systematic position follows Thompson (2011).

####### Current systematic position.

Bulimulidae, *Bulimulus unicolor* (Sowerby I, 1833).

###### Bulimus (?) illustris

Rolle, 1904

Figs 59A–C , L28i

Bulimus (?) *illustris*Rolle 1904: 36.

####### Type locality.

”Peru, Huancabamba”.

####### Label.

”Huancabamba Peru”.

####### Dimensions.

”Alt. 63, diam. max. 26,5 (...) mm”; figured specimen herein H 61.2, D 24.9, W 5+.

####### Type material.

NHMUK 1922.2.24.40, holotype (ex Rolle).

####### Remarks.

The top of the holotype is damaged. The current systematic position is after Richardson (1995). See also remarks on locality and publication date under *Bulimulus (Drymaeus) abruptus* Rolle, 1904.

####### Current systematic position.

Bulimulidae, *Newboldius crichtoni* (Broderip, 1836).

###### Bulimus immaculatus

C.B. Adams in Reeve 1850

http://species-id.net/wiki/Bulimus_immaculatus

Figs 17L–M , L28iii

Bulimus immaculatus C.B. Adams in Reeve 1850 [1848–1850]: pl. 85 fig. 631; Breure 1979: 120 (lectotype designation).
Drymaeus (Mesembrinus) immaculatus ; Breure and Eskens 1981: 73, pl. 7 fig. 4.

####### Type locality.

”Jamaica”.

####### Label.

”Jamaica”. M.C. label style IV, V.

####### Dimensions.

Not given; figured specimen herein H 30.5, D 13.17, W 6.8.

####### Type material.

NHMUK 1975540, lectotype; 1975541, two paralectotypes (Cuming coll.).

####### Remarks.

The specimen was described and figured on the basis of specimens received from Adams with a manuscript name. Breure (1979) selected as lectotype the specimen that best fitted the figure of Reeve. The current systematic position is according Rosenberg and Muratov (2005).

####### Current systematic position.

Bulimulidae, *Drymaeus (Mesembrinus) immaculatus* (C.B. Adams in Reeve 1850).

###### Bulimus incarnatus

Pfeiffer, 1855

http://species-id.net/wiki/Bulimus_incarnatus

Figs 22A , L28ii

Bulimus incarnatus Pfeiffer 1855d: 95; Breure 1979: 120 (lectotype designation).
Drymaeus (Mesembrinus) incarnatus ; Breure and Eskens 1981: 73, pl. 7 fig. 5.

####### Type locality.

”Venezuela”.

####### Label.

”Venezuela”. M.C. label style I.

####### Dimensions.

”Long. 31, diam. 12 mill.”; figured specimen herein H 30.7, D 13.1, W 7.1.

####### Type material.

NHMUK 1975566, lectotype; 1975567, one paralectotype (Cuming coll.).

####### Remarks.

The paralectotype is a juvenile shell. The current systematic position follows Richardson (1995).

####### Current systematic position.

Bulimulidae, *Drymaeus (Mesembrinus) granadensis* (Pfeiffer, 1848).

###### Bulimus inclinatus

Pfeiffer, 1862

http://species-id.net/wiki/Bulimus_inclinatus

Figs 47A–C , L28iv

Bulimus inclinatus Pfeiffer, 1862: 387, pl. 37 fig. 3; Breure 1979: 110 (lectotype designation).
Drymaeus inclinatus ; Linares and Vera 2012: 187.
Drymaeus (Drymaeus) inclinatus ; Breure and Eskens 1981: 28, pl. 8 fig. 9.

####### Type locality.

”New Grenada”.

####### Label.

”New Grenada”. M.C. label style IV.

####### Dimensions.

”Long. 33, diam. 12 mill.”; figured specimen herein H 33.1, D 17.2, W 5.7.

####### Type material.

NHMUK 1975532, lectotype; 1975533, two paralectotypes (Cuming coll.).

####### Remarks.

The diameter mentioned by Pfeiffer does not fit with his figure and is likely in error.

####### Current systematic position.

Bulimulidae, *Drymaeus (Drymaeus) inclinatus* (Pfeiffer, 1862).

###### Drymaeus incognita

da Costa, 1907

http://species-id.net/wiki/Drymaeus_incognita

Figs 33A–C , L28v

Drymaeus incognita da Costa 1907: 304, pl. 26 figs 4–4a; Breure 1979: 110; Linares and Vera 2012: 188.

####### Type locality.

[Colombia] ”Bogota”.

####### Label.

”Bogota”, in da Costa’s handwriting.

####### Dimensions.

”Long. 29.5, diam. 12 mm”; figured specimen herein H 29.8, D 13.7, W 6.3.

####### Type material.

NHMUK 1907.11.21.24, holotype; 1907.11.21.25, one paratype (da Costa coll.).

####### Remarks.

This taxon was described by da Costa on the basis of ”two specimens”.

####### Current systematic position.

Bulimulidae, *Drymaeus (Drymaeus) incognitus* da Costa, 1907.

###### Bulimus incrassatus

Pfeiffer, 1853

http://species-id.net/wiki/Bulimus_incrassatus

Figs 14B–C , L29i

Bulimus incrassatus Pfeiffer 1853d: 475; Pfeiffer 1853 in Küster and Pfeiffer 1840–1865: 79, pl. 30 figs 13–14; Pfeiffer 1854d: 157; Breure 1979: 69 (lectotype designation).
Bulimulus nux var. *incrassatus*; Pilsbry 1897 [1897–1898]: 102, pl. 16 figs 42–43.

####### Type locality.

[Ecuador] ”insulis Gallapagos”.

####### Label.

”Brazils”, ”Galapagos”, taxon label in Pfeiffer’s handwriting. M.C. label style III.

####### Dimensions.

”Long. 17 1/2, diam. 8 1/2 mill.”; figured specimen herein H 17.3, D 9.0, W 7.0.

####### Type material.

NHMUK 1975157, lectotype; 1975158, three paralectotypes (Cuming coll.).

####### Remarks.

The original label had the locality as ”Brazils”, but in a later hand (probably E.A. Smith’s) this has been corrected to Galapagos. The current systematic position follows Richardson (1995: 225).

####### Current systematic position.

Bulimulidae, *Naesiotus nux* (Broderip, 1832).

###### Bulimus infundibulum

Pfeiffer, 1853

http://species-id.net/wiki/Bulimus_infundibulum

Figs 1C–D , L30iii

Bulimus infundibulum Pfeiffer, 1853b: 255; Pfeiffer 1853 in Küster and Pfeiffer 1840–1865: 85, pl. 30 figs 19–20; Pfeiffer 1853d: 375; Breure 1979: 55.
Bulimulus (Ataxus) infundibulum ; Pilsbry 1896 [1895–1896]: 131, pl. 44 figs 91–92.
Bostryx infundibulum ; Breure 1978: 92 (lectotype designation).

####### Type locality.

”Andibus Peruvianis”.

####### Label.

”Peru + Bolivia”. M.C. label style I, IV.

####### Dimensions.

”Long. 18, diam. 7 mill.”; figured specimen herein H 18.3, D 6.82, W 9.0.

####### Type material.

NHMUK 1975163, lectotype; 1975164, two paralectotypes (Cuming coll.).

####### Remarks.

The label is apparently partly in error, as this species is only known from southern Peru. The current systematic position is after Breure (1979).

####### Current systematic position.

Bulimulidae, *Bostryx infundibulum* (Pfeiffer, 1853).

###### Bulimus inglorius

Reeve, 1848

http://species-id.net/wiki/Bulimus_inglorius

Figs 24J , L30i

Bulimus inglorius Reeve 1848 [1848–1850]: pl. 55 fig. 368; Breure 1979: 120 (lectotype designation).
Drymaeus inglorius ; Pilsbry 1899: 67, pl. 3 fig. 46.
Drymaeus (Mesembrinus) inglorius ; Breure and Eskens 1981: 73; Thompson 2011: 115.

####### Type locality.

”—?”.

####### Label.

”—?”. M.C. label style IV, V.

####### Dimensions.

Not given; figured specimen herein H 25.6, D 13.2, W 5.7.

####### Type material.

NHMUK 1975536, lectotype; 1975537, two paralectotypes (Cuming coll.).

####### Remarks.

The current systematic position is according to Thompson (2011).

####### Current systematic position.

Bulimulidae, *Drymaeus (Mesembrinus) inglorius* (Reeve, 1848).

###### Bulimulus (Drymaus) interruptus

Preston, 1909

http://species-id.net/wiki/Bulimulus_interruptus

Figs 18A–B , L29iii

Bulimulus (Drymaeus) interruptus Preston 1909: 511, fig. 1; Breure 1979: 120.
Drymaeus (Mesembrinus) interruptus ; Köhler 2007: 151, fig. 119.

####### Type locality.

”Merida, Venezuela”.

####### Label.

”Merida, Venezuela”.

####### Dimensions.

”Alt. 25.5, diam. maj. 10.5 mm”; figured specimen herein H 24.5, D 11.3, W 6.3.

####### Type material.

NHMUK 1914.4.3.38, lectotype and five paralectotypes (ex Preston).

####### Remarks.

Preston described for this taxon also two forms (*interruptus* form α and β), and three varieties (var. *pallidus*, var. *pallidus* form γ, and var. *pallidu* s form δ); as he gave them explicitly infrasubspecific rank these names are not available (Art. 45.6 ICZN). The shells, from the same locality, clearly show the intraspecific variation that may occur in this taxon. Köhler (2007) has interpreted the reference to ”HT BMNH 1914.4.3.38” in Breure (1979: 120) as an unjustified designation since Preston evidently did base his description on more than one specimen. However, under Art. 74.6 ICZN the reference of Breure (1979) may be considered as a lectotype designation; specimen ZMB 59597 is therefore a paralectotype.

####### Current systematic position.

Bulimulidae, *Drymaeus (Mesembrinus) interruptus* (Preston, 1909).

###### Bulimulus (Drymaeus) inusitatus

Fulton, 1900

http://species-id.net/wiki/Bulimulus_inusitatus

Figs 20F–G , L30ii

Bulimulus (Drymaeus) inusitatus Fulton 1900: 87; Breure 1979: 120.
Drymaeus (Mesembrinus) inusitatus ; Kohler 2007: 151, fig. 121; Thompson 2011: 120.

####### Type locality.

”Costa Rica”.

####### Label.

”Costa Rica”, in Fulton’s handwriting.

####### Dimensions.

”Alt. 29 1/5, diam. maj. 13 (...) mill.”; figured specimen herein H 28.3, D 13.1, W 7.8.

####### Type material.

NHMUK 1901.4.25.28, lectotype (ex Fulton).

####### Remarks.

Fulton did not mention on how many specimens his description was based. Breure (1979: 120) referred to the specimen in NHMUK as ”HT BMNH 1901.4.25.28”. Köhler (2007) found an additional specimen in ZMB which he regarded as probable type material and considered all specimens as syntypes. However, under Art. 74.6 ICZN Breure’s designation may be considered as a lectotype designation; specimen ZMB 52678 is therefore a probable paralectotype.

####### Current systematic position.

Bulimulidae, *Drymaeus (Mesembrinus) inusitatus* (Fulton, 1900).

###### Bulimus inutilis

Reeve, 1850

http://species-id.net/wiki/Bulimus_inutilis

Figs 62G–H , L30iv

Bulimus inutilis Reeve 1850 [1848–1850]: pl. 86 fig. 639; Breure 1979: 63.
Bulimulus inutilis ; Pilsbry 1897 [1897–1898]: 73, pl. 11 fig. 37; Breure 1978: 144 (lectotype designation); Thompson 2011: 107.

####### Type locality.

”—?”.

####### Label.

”?Centr. America”. M.C. label style III, V.

####### Dimensions.

Not given; figured specimen herein H 16.5, D 8.9, W 6.0.

####### Type material.

NHMUK 1975162, lectotype (Cuming coll.).

####### Remarks.

The current systematic position follows Thompson (2011).

####### Current systematic position.

Bulimulidae, *Bulimulus inutilis* (Reeve, 1850).

###### Bulimus iodostylus

Pfeiffer, 1861

http://species-id.net/wiki/Bulimus_iodostylus

Figs 54C–D , L30v

Bulimus iodostylus Pfeiffer 1861: 23; Pfeiffer 1868: 47; Breure 1979: 110.
Drymaeus (Mesembrinus) ghiesbreghti iodostylus ; Thompson 2011: 113.

####### Type locality.

”Mexico”.

####### Label.

”Mexico”, taxon label in Pfeiffer’s handwriting. M.C. label style IV.

####### Dimensions.

”Long. 30, diam. 12 mill.”; figured specimen herein H 32.2, D 15.3, W 6.2.

####### Type material.

NHMUK 1975568, three syntypes (Cuming coll.).

####### Remarks.

The largest specimen in the syntype lot was measured and is here figured. The current systematic position follows Thompson (2011), who regarded Oaxaca as the type locality.

####### Current systematic position.

Bulimulidae, *Drymaeus (Mesembrinus) ghiesbreghti iodostylus* (Pfeiffer, 1861).

###### Bulimus irregularis

Pfeiffer, 1848

http://species-id.net/wiki/Bulimus_irregularis

Figs 15B , L30vi

Bulimus irregularis Pfeiffer 1848a: 231; Pfeiffer 1848b: 183; Reeve 1849 [1848–1850]: pl. 65 fig. 454.

####### Type locality.

”Quito, Ecuador”.

####### Label.

”Ecuador”. M.C. label style I, V.

####### Dimensions.

”Long. 19, diam. 9 mill.”; figured specimen herein H 18.8, D 9.28, W 6.2.

####### Type material.

NHMUK 20100564, one possible syntype (Cuming coll.).

####### Remarks.

A lot was found under this name, containing two specimens. One specimen corresponds to the measurements given by Pfeiffer and to Reeve’s figure; it is, however, not accompanied by a taxon label in Pfeiffer’s handwriting, and therefore considered a possible syntype. The other specimen belongs to a different species, viz. *Kuschelenia (Vermiculatus) anthisanensis* (Pfeiffer, 1853), and is here excluded from the type series. The current systematic position follows Richardson (1995).

####### Current systematic position.

Bulimulidae, *Naesiotus quitensis* (Pfeiffer, 1848).

###### Bulimulus istapensis

Crosse and Fischer, 1873

http://species-id.net/wiki/Bulimulus_istapensis

Figs 63O–P , L30vii

Bulimulus istapensis Crosse and Fischer 1873: 286; Fischer and Crosse 1875: 549, pl. 20 fig. 18.

####### Type locality.

”Istapa, Guatemalae, in silvis (A. Morelet)”.

####### Label.

”Forests d’Istapa, Guatemala”, in Morelet’s hamdwriting.

####### Dimensions.

”Long. 14 1/2, diam. maj. vix 7 mill.”; figured specimen herein H 11.2, D 6.33, W 5.9.

####### Type material.

NHMUK 1893.2.4.1185–1187, three possible syntypes (Morelet coll.).

####### Remarks.

The original description does not mention on how many specimens it was based, but does refer to a specimen in the Morelet collection. The specimens, of which one is broken, are considered possible syntypes. The current systematic position follows Thompson (2011).

####### Current systematic position.

Bulimulidae, *Bulimulus unicolor* (Sowerby I, 1833).

###### Bulinus jacobi

(Sowerby I, 1833)

http://species-id.net/wiki/Bulinus_jacobi

Figs 13B–C , L31i

Bulinus jacobi Sowerby I 1833: 74; Sowerby I 1833 [Sowerby I and II 1832–1841]; fig. 45.
Bulimus jacobi ; Reeve 1848 [1848–1850]: pl. 21 fig. 135.

####### Type locality.

”ad Insulam Jacobi, inter Gallapagos”.

####### Label.

”Galapagos Is.” [1842.5.10.250–252], ”James I. Galapagos” [20100648], both in E.A. Smith’ handwriting. M.C. label style III, V.

####### Dimensions.

”long. 0.55, lat. 0.3 poll. [H 13.9 D 7.6 mm]”; figured specimen herein H 11.9, D 6.8, W 5.7.

####### Type material.

NHMUK 1842.5.10.250–252, three possible syntypes; 20100648, four possible syntypes (Cuming coll.).

####### Remarks.

The two lots originating from the Cuming collection are considered possible syntypes. One specimen from lot 20100648 corresponds to Reeve’s figure. There is one additional specimen (NHMUK 1837.4.8.33.576), which according to the registration book, was ”purchased from Mr. Eling, Deptford, from Capt Fitzroy’s vessel”; this is not considered as type material but may have been collected by Darwin on his voyage with the ‘Beagle’.

####### Current systematic position.

Bulimulidae, *Naesiotus jacobi* (Sowerby I, 1833).

###### Otostomus lilacinus jansoni

Martens, 1893

http://species-id.net/wiki/Otostomus_lilacinus_jansoni

Figs 53D–F , L30viii

Otostomus lilacinus jansoni Martens 1893 [1890–1901]: 201, pl. 12 figs 3, 3a–b; Breure 1979: 110 (lectotype designation).
Drymaeus lilacinus var. *jansoni*; Pilsbry 1899: 37, pl. 7 figs 7–9.
Drymaeus (Drymaeus) lilacinus ; Breure and Eskens 1981: 28.
Drymaeus (Drymaeus) lilacinus jansoni ; Köhler 2007: 146, fig. 98; Thompson 2011: 111.

####### Type locality.

”Nicaragua”.

####### Label.

”Nicaragua”, taxon label in Martens’ handwriting.

####### Dimensions.

”Long. 46, diam. 22 millim.”; figured specimen herein H 45.7, D 22.3, W 6.8.

####### Type material.

NHMUK 1901.6.22.951, lectotype, Janson leg. (Godman coll.).

####### Remarks.

The current systematic position follows Thompson (2011).

####### Current systematic position.

Bulimulidae, *Drymaeus (Drymaeus) lilacinus jansoni* (Martens, 1893).

###### Bulimus jonasi

Pfeiffer, 1846

http://species-id.net/wiki/Bulimuss_jonasi

Figs 18F–G , L31ii

Bulimus jonasi Pfeiffer in Philippi 1846 [1845–1847]: 125, pl. 5 fig. 4; Pfeiffer 1848b: 107; Reeve 1848 [1848–1850]: pl. 55 fig. 363; Breure 1979: 120.
Drymaeus jonasi ; Pilsbry 1899: 54, pl. 10 fig. 71.
Drymaeus (Mesembrinus) jonasi ; Thompson 2011: 125.

####### Type locality.

”Vera Cruz Americae centralis (Lattre in coll. Cuming)”.

####### Label.

”Vera Cruz, C. America”, taxon label in Pfeiffer’s handwriting. M.C. label style I, IV, V.

####### Dimensions.

”Long 13´´´, Diam. 5´´´ [H 28.3, D 10.9 mm]”; figured specimen herein herein H 26.6, D 11.6, W 6.0.

####### Type material.

NHMUK 1975557, six syntypes (Cuming coll.).

####### Remarks.

The measurements as given by Pfeiffer are interpreted as German lines, i.e. 2.18 mm. The current systematic position is following Thompson (2011).

####### Current systematic position.

Bulimulidae, *Drymaeus (Mesembrinus) jonasi* (Pfeiffer in Philippi 1846).

###### Bulimus josephus

Angas, 1878

http://species-id.net/wiki/Bulimus_josephus

Fig. L31iii

Bulimus josephus Angas 1878a: 72, pl. 5 figs 13–14; Breure 1979: 110.
Drymaeus (Drymaeus) josephus ; Thompson 2011: 108.

####### Type locality.

”San José, Costa Rica”.

####### Label.

”in the low hills / Talamanca / Costa Rica”.

####### Dimensions.

”Diam. 7 lines, alt. 16 [H 33.8 D 14.8 mm]”; figured specimen herein H 31.0, D 15.6, W 6.8.

####### Type material.

NHMUK 1879.7.22.16–18, three possible syntypes (ex Angas).

####### Remarks.

Angas did not mention on how many specimens his description was based, but mentioned ”Mus. Boucard”. The lot found consists of smaller specimens and are considered as possible syntypes. The current systematic position follows Thompson (2011).

####### Current systematic position.

Bulimulidae, *Drymaeus (Drymaeus) josephus* (Angas, 1878).

###### Bulimus juarezi

Pfeiffer, 1866

http://species-id.net/wiki/Bulimus_juarezi

Figs 72G–I , L31iv

Bulimus juarezi Pfeiffer 1866: 832; Pfeiffer 1866 [1860–1866]: 230, pl. 69 figs 1–2; Breure 1979: 76.
Rabdotus (Rabdotus) sufflatus (Gould, 1859); Thompson 2011: 128.

####### Type locality.

[Mexico] ”in provinciae pacifica reipublicae mexicanae”.

####### Label.

”West Mexico”. M.C. label style IV.

####### Dimensions.

”Long. 31, diam. 18 mill.”; figured specimen herein H 31.5, D 19.3, W 5.4.

####### Type material.

NHMUK 1975421, three syntypes (Cuming coll.).

####### Remarks.

The current systematic position follows Thompson (2011).

####### Current systematic position.

Bulimulidae, *Rabdotus (Rabdotus) sufflatus* (Gould, 1859).

###### Bulimus jussieui

Pfeiffer, 1846

http://species-id.net/wiki/Bulimus_jussieui

Figs 66L–N , L31v

Bulimus jussieui Pfeiffer 1846a: 33; Pfeiffer 1846c: 55; Reeve 1848 [1848–1850]: pl. 39 fig. 242; Breure 1979: 88.
Bulimulus (Scutalus) culmineus (d’Orbigny, 1835); Crawford 1939: 328.
Scutalus (Kuschelenia) culmineus culmineus ; Breure 1978: 177.

####### Type locality.

[Peru] ”Cusoo” (sic, Cusco).

####### Label.

”Cusco / Peru / Val.”, taxon label in Pfeiffer’s handwriting. M.C. label style III, IV.

####### Dimensions.

”Long. 32, diam. 15 mill.”; figured specimen herein H 31.6, D 16.3, W 5.9.

####### Type material.

NHMUK 1975170, lectotype and two paralectotypes (Cuming coll.).

####### Remarks.

Pfeiffer (1846a) indicated that he described shells from ”Val. Mur.” (sic, Val. Mus. = Valenciennes collection). According to Crawford (1939) he found two specimens with reference to ”Val.” on the label; however, we found the same lot containing three specimens. Crawford noted on a separate label ”The left hand spec. seems from its label + the dimensions to be the spec. described as B. jussieui Pfr. 1846 + fig. Reeve C.I (V) 242”; this specimen is here designated lectotype (**design. n.**) to define the taxon. There is no label from Reeve accompanying the lot. The current systematic position follows Richardson (1995: 350).

####### Current systematic position.

Bulimulidae, *Kuschelenia (Kuschelenia) culminea culminea* (d’Orbigny, 1835) (**comb. n.**).

###### Bulimus juvenilis

Pfeiffer, 1855

http://species-id.net/wiki/Bulimus_juvenilis

Figs 61A–B , L31vi

Bulimus juvenilis Pfeiffer 1855d: 97; Pfeiffer 1859b: 503; Breure 1979: 63.
Bulimulus juvenilis ; Breure 1978: 145 (lectotype designation); Linares and Vera 2012: 163.

####### Type locality.

[Colombia] ”Santa Fé de Bogota”.

####### Label.

”Santa Fé de Bogota”, taxon label in Pfeiffer’s handwriting. M.C. label style III.

####### Dimensions.

”Long 20, diam. 8 1/2 mill.”; figured specimen herein H 19.5, D 8.7, W 6.5.

####### Type material.

NHMUK 1975161, lectotype (Cuming coll.).

####### Remarks.

The current systematic position follows Richardson (1995: 77).

####### Current systematic position.

Bulimulidae, *Bulimulus juvenilis* (Pfeiffer, 1855).

###### Bostryx kathiae

Breure, 1978

http://species-id.net/wiki/Bostryx_kathiae

Fig. 8D

Bostryx kathiae Breure 1978: 92, figs 138–141.

####### Type locality.

”Peru, Dept. Lima, Río Cañete valley, 1 km above Puente Auco, 2070 m”.

####### Label.

”Peru, Dept. Lima, Río Cañete valley, 1 km above Puente Auco, 2070 m”.

####### Dimensions.

”H 18.5 D 9.4”; figured specimen herein H 17.5, D 8.8, W 6.4.

####### Type material.

NHMUK 1975228, one paratype, A.S.H. Breure leg.

####### Current systematic position.

Bulimulidae, *Bostryx kathiae* Breure, 1978.

###### Bulimus keppelli

Pfeiffer, 1853

http://species-id.net/wiki/Bulimus_keppelli

Figs 25G , L32i

Bulimus keppelli Pfeiffer 1853d: 653; Pfeiffer 1854e: 50.
Drymaeus (Mesembrinus) keppelli ; Breure and Eskens 1981: 76, pl. 7 fig. 11 (lectotype designation).

####### Type locality.

”in Andibus Peruanis (Keppell)”.

####### Label.

”Andes of Peru / Hon. Cap. Keppell”, taxon label in Pfeiffer’s handwriting. M.C. label style IV.

####### Dimensions.

”Long. 34 1/2, diam. 14 mill.”; figured specimen herein H 33.2, D 14.2, W 6+.

####### Type material.

NHMUK 1975538, lectotype (Cuming coll.).

####### Remarks.

The top of the specimen is damaged. The current systematic position is following Richardson (1995).

####### Current systematic position.

Bulimulidae, *Drymaeus (Drymaeus) vexillum* (Wood, 1828).

###### Bulimus knorri

Pfeiffer in Philippi 1846

http://species-id.net/wiki/Bulimus_knorri

Figs 51D–F , L32iii

Bulimus knorri Pfeiffer in Philippi 1846 [1845–1847]: 115, pl. 4 fig. 3; Pfeiffer 1848b: 95; Reeve 1848 [1848–1850]: pl. 43 fig. 270.

####### Type locality.

”La Guayra”.

####### Label.

”La Guayra”, taxon label in Pfeiffer’s handwriting. M.C. label style I, V.

####### Dimensions.

”Long. 18, diam 6´´´ [H 39.2, D 13.1 mm]”; figured specimen herein H 34.1, D 17.1, W 6.5.

####### Type material.

NHMUK 20100654, three possible syntypes (Cuming coll.).

####### Remarks.

Pfeiffer did not describe on how many specimens his description was based. The lot found has the taxon label in Pfeiffer’s handwriting and the locality corresponds to the original one; it is not know whether this locality lies in the Colombian or Venezuelan part of La Guayra. The largest specimen is smaller than indicated in the original description; the specimens are considered as possible syntypes. Given the shell shape, and the expanded lip this taxon may be better placed in *Drymaeus* *s. str.* At species level the current systematic position is according to Richardson (1995: 187).

####### Current systematic position.

Bulimulidae, *Drymaeus (Drymaeus) trigonostomus* (Jonas, 1844) (**comb.n.**).

###### Bulimus koppeli

Sowerby III, 1892

http://species-id.net/wiki/Bulimus_koppeli

Figs 24H , L32iv

Bulimus koppeli Sowerby III 1892: 297, pl. 23 figs 9–12; Breure 1979: 120 (lectotype designation).
Drymaeus koppeli ; Pilsbry 1899: pl. 6 figs 1–4; Linares and Vera 2012: 195.

####### Type locality.

[Colombia] ”Bogota”.

####### Label.

”Bogota”, in da Costa’s handwriting.

####### Dimensions.

”Long. 25, diam. maj. 15 millim.”; figured specimen herein H 25.5, D 15.0, W 5.6.

####### Type material.

NHMUK 1907.11.21.133, lectotype, 1907.11.21.134, one paralectotype (da Costa coll.).

####### Remarks.

Sowerby III (1892) described the species on the basis of ”two specimens lent [to] me for description by Mr. Da Costa”. The current systematic position follows Richardson (1995).

####### Current systematic position.

Bulimulidae, *Drymaeus (Mesembrinus) koppeli* (Sowerby III, 1892).

###### Bulimus lactifluus

Pfeiffer, 1857

http://species-id.net/wiki/Bulimus_lactifluus

Figs 2C–D , L32ii

Bulimus lactifluus Pfeiffer 1857c: 330; Pfeiffer 1859b: 407; Pfeiffer 1868 [1866–1869]: 425, pl. 96 figs 13–14.
Bulimulus (Peronaeus) lactifluus ; Pilsbry 1896 [1895–1896]: 140, pl. 10 figs 15–16.

####### Type locality.

”Chili”.

####### Label.

”Chile”. M.C. label style I.

####### Dimensions.

”Long. 16 1/2–17, diam. 4 2/3 mill.”; figured specimen herein H 16.2, D 4.7, W 10.6.

####### Type material.

NHMUK 20100642, four possible syntypes (Cuming coll.).

####### Remarks.

From the measurements given by Pfeiffer it is clear that he had multiple specimens for his description. As he described this species from the Cuming collection, but no taxon label in Pfeiffer’s handwriting was found, this lot is considered as possible syntypes. The current systematic position is according to Richardson (1995: 30).

####### Current systematic position.

Bulimulidae, *Bostryx lactifluus* (Pfeiffer, 1857).

###### Bulimus laetus

Reeve, 1849

http://species-id.net/wiki/Bulimus_laetus

Figs 18E , L32v

Bulimus laetus Reeve 1849 [1848–1850]: pl. 83 fig. 616; Breure 1979 (lectotype designation).
Drymaeus laetus ; Pilsbry 1898 [1897–1898]: 245, pl. 43 fig. 72; Linares and Vera 2012: 195 [partim].

####### Type locality.

[Colombia] ”Sebundoi, New Granada”.

####### Label.

”in Abejonal [?, labels partly illegible]”. M.C. label style IV, V.

####### Dimensions.

Not given; figured specimen herein H 26.6, D 13.15, W 6.0.

####### Type material.

NHMUK 1975534, lectotype, 1975535, two paralectotypes (Cuming coll.).

####### Remarks.

Reeve described the taxon from ”Mus. Cuming” and noted ”Mr Taylor has a specimen collected independently of this, of exactly similar pattern and colour”. The lot found is accompanied by a label that indicates that it was figured by Reeve (Breure and Ablett 2011: 8, fig. 4C–F). The current systematic position follows Richardson (1995).

####### Current systematic position.

Bulimulidae, *Drymaeus (Mesembrinus) laetus* (Reeve, 1849).

###### Bulimus (Otostomus) lamas

Higgins, 1868

http://species-id.net/wiki/Bulimus_lamas

Figs 54E , L33i

Bulimus (Otostomus) lamas Higgins 1868: 179, pl. 14 figs 3–3a; Breure 1979: 110.
Drymaeus lamas ; Pilsbry 1898 [1897–1898]: 272, pl. 48 figs 26–27.

####### Type locality.

”Jouctabamba, Peru”.

####### Label.

”Peru”.

####### Dimensions.

”Long. 33, diam. 10 mill.”; figured specimen herein H 29.2, D 9.6, W 7.9.

####### Type material.

NHMUK 1868.4.3.3, three possible syntypes (ex R. Geale).

####### Remarks.

Higgins (1868) did not mention on how many specimens his description was based. The taxon label bears a handwriting that may be Higgins’, but this cannot be ascertained. The largest shell in the lot found is smaller than the original measurements given; the specimens are considered as possible syntypes. The taxon is now considered a junior subjective synonym of *Bulimus trujillensis* Philippi, 1867 (**syn. n.**).

####### Current systematic position.

Bulimulidae, *Drymaeus (Drymaeus) trujillensis* (Philippi, 1867).

###### Bulimulus (Drymaeus) binominis lascellianus

E.A. Smith, 1895

http://species-id.net/wiki/Bulimulus_binominis_lascellianus

Figs 23I , L33ii

Bulimulus (Drymaeus) binominis lascellianus E.A. Smith 1895: 316, pl. 21 fig. 14.

####### Type locality.

[West Indies, Grenada] ”Annandale estate” (see remarks).

####### Label.

”Grenada”.

####### Dimensions.

Not given; figured specimen herein H 22.1, D 11.22, W 5.9.

####### Type material.

NHMUK 1895.9.10.1, lectotype; 1895.9.10.2, one paralectotype.

####### Remarks.

Smith (1895) wrote ”Mr. J.H. Ponsonby possesses a new and interesting colour-variety (var. *Lascellianus*) of this species. (...) only found on the Annandale estate, and only on one small part of that—a strip of land facing west on a rocky mountain side, at an elevation of 1,000 to 1,200 feet.” Smith did not mention on how many specimens his description was based, but one of the two specimens found corresponds to his figure and is here selected lectotype (**design. n.**). The taxon is now regarded a junior synonym of *Bulimulus (Drymaeus) binominis* E.A. Smith, 1895 (**syn. n.**).

####### Current systematic position.

Bulimulidae, *Drymaeus (Mesembrinus) binominis* (E.A. Smith, 1895).

###### Bulimulus latecolumellaris

Preston, 1909

http://species-id.net/wiki/Bulimulus_latecolumellaris

Figs 65I–J , L33iii

Bulimulus latecolumellaris Preston 1909: 510, fig. 11; Breure 1979: 83 (lectotype designation).

####### Type locality.

”Peru”.

####### Label.

”Peru”, label in Preston’s handwriting.

####### Dimensions.

”Alt. 54, diam. maj. 24 mm”; figured specimen herein H 54, D 23.9, W 7+.

####### Type material.

NHMUK 1922.2.24.39, lectotype (ex Preston).

####### Remarks.

The top of the specimen is damaged. Preston (1909) did not mention on how many specimens his description was based; Breure’s record of the specimen as holotype has therefore to be interpreted as lectotype designation according to Art. 74.6 ICZN. The current systematic position follows Richardson (1995: 352).

####### Current systematic position.

Bulimulidae, *Scutalus latecolumellaris* Preston, 1909.

###### Bulimus lattrei

Pfeiffer in Philippi 1846

http://species-id.net/wiki/Bulimus_lattrei

Figs 50G–I , L33iv

Bulimus lattrei Pfeiffer in Philippi 1846 [1845–1847]: 112, pl. 4 fig. 11; Breure 1979: 110 (lectotype designation).
Bulimus focillatus Reeve 1848 [1848–1850]: pl. 36 fig. 211.
Drymaeus lattrei ; Pilsbry 1899: 41, pl. 8 fig. 24.
Drymaeus (Drymaeus) lattrei ; Breure and Eskens 1981: 28; Thompson 2011: 110.

####### Type locality.

”America centralis; prope Veracruz legit Lattre” (see remarks).

####### Label.

”Mon^s^ Lattre / Mountains of Vera / Cruz, Central America”. M.C. label style IV.

####### Dimensions.

”Long. 21, diam. 10´´´ [H 45.8, D 21.8 mm]”; figured specimen herein H 42.6, D 22.2, W 6.4.

####### Type material.

NHMUK 1975555, lectotype; 1975556, eight paralectotypes (Cuming coll.) (see remarks).

####### Remarks.

The type locality was corrected to Guatemala, Vera Paz [Dept. Altaverapaz] by Pilsbry (1899: 42). Breure and Eskens (1981) mentioned two paralectotypes; a modern label (after 1975) states ”Also Syntypes of Bulimus focillatus”, which makes it likely that six specimens have been added. There is no conclusive evidence that these were Reeve’s material; however, the lot cannot be untangled. The current systematic position is following Thompson (2011).

####### Current systematic position.

Bulimulidae, *Drymaeus (Drymaeus) lattrei lattrei* (Pfeiffer in Philippi 1846).

###### Bulimulus laxostylus

Rolle, 1904

http://species-id.net/wiki/Bulimulus_laxostylus

Figs 74E–G , L33v

Bulimulus laxostylus Rolle 1904: 37; Breure 1979: 110.

####### Type locality.

”Huancabamba in Peru”.

####### Label.

”Huancabamba / Peru / Andes 6–10.000 ft.”, taxon label in Rolle’s handwriting.

####### Dimensions.

”Alt. 40, diam. 16,5 mm”; figured specimen herein H 39.9, D 15.4, W 7.1.

####### Type material.

NHMUK 1922.2.24.32, lectotype (ex Rolle).

####### Remarks.

Rolle did not mention on how many specimens his description was based; the label reads ”largest is type”, suggesting that more than one specimen might have been seen. The record of the holotype in Breure (1979: 110) has therefore to be interpreted as lectotype designation. The taxon is here figured for the first time. See also remarks on locality and publication date under *Bulimulus (Drymaeus) abruptus* Rolle, 1904.

####### Current systematic position.

Bulimulidae, *Drymaeus (Drymaeus) laxostylus* (Rolle, 1904).

###### Bulimus lesueureanus

Morelet, 1860

http://species-id.net/wiki/Bulimus_lesueureanus

Figs 1G–H , L33vi

Bulimus lesueureanus Morelet 1860: 374; Morelet 1863: 200, pl. 9 fig. 4; Breure 1979: 55.
Bulimulus (Peronaeus) lusueureanus ; Pilsbry 1896 [1895–1896]: 149, pl. 46 fig. 45.
Bostryx lesueureanus ; Breure 1978: 97.

####### Type locality.

”[in intimâ Peruvii regione]” (see remarks).

####### Label.

”Pomacocha, Pérou”, taxon label in Morelet’s handwriting.

####### Dimensions.

”Longit. 21; diam. 7 mill.”; figured specimen herein H 22.5, D 7.7, W 7.7.

####### Type material.

NHMUK 1893.2.4.1182–1183, two syntypes (Morelet coll.).

####### Remarks.

The type locality was specified in Morelet (1863: 201) ”Il provient de Pomacocha et Cocharcas”. The current systematic position is according to Richardson (1995: 37).

####### Current systematic position.

Bulimulidae, *Bostryx orophilus* (Morelet, 1860).

###### Helix lichnorum

d’Orbigny, 1835

http://species-id.net/wiki/Helix_lichnorum

Figs 2E–F , L34i

Helix lichnorum d’Orbigny 1835: 20.
Bulimus lichenorum ; d’Orbigny 1837 [1834–1847]: 264, pl. 41 figs 9–10 (emendation) [6 Nov. 1837; text 23 April 1838]; Gray 1854: 13.
Bulimulus (Peronaeus) lichenorum ; Pilsbry 1896 [1895–1896]: 149, pl. 46 figs 34-35.

####### Type locality.

[Chile] ”Cobija (republica Boliviana)”.

####### Label.

”cobija. Bolivia”, taxon label in d’Orbigny’s handwriting.

####### Dimensions.

”Longit. 17 millim., latit. 5 millim.”; figured specimen herein H 15.8, D 5.14, W 7.1.

####### Type material.

NHMUK 1854.12.4.96, lectotype and four paralectotypes (d’Orbigny coll.).

####### Remarks.

The type locality now belongs to the political administration of Chile. The lot found consists of five specimens, of which one corresponds to the figure of d’Orbigny and is here designated lectotype (**design. n.**) to define the taxon.

####### Current systematic position.

Bulimulidae, *Bostryx lichnorum* (d’Orbigny, 1835).

###### Bulimus lilacinus

Reeve, 1849

http://species-id.net/wiki/Bulimus_lilacinus

Figs 53A–C , L34iii

Bulimus lilacinus Reeve 1849 [1848–1850]: pl. 74 fig. 532.

####### Type locality.

”—?”.

####### Label.

”New Granada”, in a later handwriting (see remarks). M.C. label style I, V.

####### Dimensions.

Not given; figured specimen herein H 45.5, D 20.3, W 6+.

####### Type material.

NHMUK 20100520, four probable syntypes (Cuming coll.).

####### Remarks.

One lot was found, of which one shell is very similar to Reeve’s figure. The shells are considered probable syntypes. Pfeiffer (1853: 326) considered these shells juveniles and conspecific with *Bulimus patricius* Reeve, 1849, which was published later in the same year. Fischer and Crosse (1875: 478, pl. 20 figs 1–2) were the first to recognize that this a Guatemalan species.

####### Current systematic position.

Bulimulidae, *Drymaeus (Drymaeus) lilacinus* (Reeve, 1849).

###### Bulimus limensis

Reeve, 1849

http://species-id.net/wiki/Bulimus_limensis

Figs 6E , L34v

Bulimus limensis Reeve 1849 [1848–1850]: pl. 77 fig. 563; Breure 1979: 55; Ramírez 2004: 41, fig. 21H.
Bostryx limensis ; Breure 1978: 97, pl. 9 fig. 1 (lectotype designation).

####### Type locality.

”Lima and Quito, South America”.

####### Label.

”Peru”. M.C. label style IV, V.

####### Dimensions.

Not given; figured specimen herein H 19.6, D 10.46, W 7.0.

####### Type material.

NHMUK 1975326, lectotype; 1975327, three paralectotypes (Cuming coll.).

####### Remarks.

Ramírez (2004: 41, 47) placed this taxon in the synonymy of *Bulimus modestus* (Broderip in Broderip and Sowerby 1832); her—formally unpublished treatment—of this taxon is followed in the current systematic position.

####### Current systematic position.

Bulimulidae, *Bostryx modestus* (Broderip in Broderip and Sowerby 1832).

###### Helix limonoica

d’Orbigny, 1835

http://species-id.net/wiki/Helix_limonoica

Figs 11B , L34ii

Helix limonoica d’Orbigny 1835: 13.
Bulimus limonoicus d’Orbigny 1837 [1834–1847]: 284, pl. 33 figs 15–17 [19 June / 7 Aug 1837; text 6 May 1838].
Peronaeus (Lissoacme) limonoicus ; Breure 1975b: 1141, fig. 1 (lectotype designation).
Bostryx limonoicus ; Breure 1979: 55.

####### Type locality.

”provincia Chiquitensi (republica Boliviana)”; see Breure 1973: 118.

####### Label.

”S^n^ Juan. P.Chiquitos Bolivia”, in d’Orbigny’s handwriting.

####### Dimensions.

”Latit. 19 millim., longit. 8 millim.”; figured specimen herein H 15.8, D 5.14, W 7.1.

####### Type material.

NHMUK 1854.12.4.190, two paralectotype (d’Orbigny coll.).

####### Remarks.

d’Orbigny (1838 [1834–1847]: 284) emended the type location ”près des ruines de l’ancienne Mission de San-Juan”. One of the paralectotypes correspondonds to the figures given by d’Orbigny. The lectotype in MNHN is a subadult specimen. The current systematic position follows Richardson (1995).

####### Current systematic position.

Bulimulidae, *Bostryx limonoicus* (d’Orbigny, 1835).

###### Helix linostoma

d’Orbigny, 1835

http://species-id.net/wiki/Helix_linostoma

Figs 28A–C , L34iv

Helix linostoma d’Orbigny 1835: 19.
Bulimus linostoma ; d’Orbigny 1837 [1834–1847]: 314, pl. 40 figs 9–11 [18 Sept. 1837; text 6 May 1838]; Gray 1854: 17.
Drymaeus linostoma ; Pilsbry 1898 [1897–1898]: 218, pl. 36 figs 41–42; Breure 1975b: 1151.

####### Type locality.

”provincia Chiquitensi (republica Boliviana)”; see Breure 1973: 118.

####### Label.

”guarayos, Bolivia”; in d’Orbigny’s handwriting.

####### Dimensions.

”Longit. a 26 ad 31 millim., latit. 2 ad 14 millim.”; in d’Orbigny 1837 [1834–1847]: ”Long. 29 millim., lat. 14 millim.”; figured specimen herein H 29.7, D 14.9, W 6.2.

####### Type material.

NHMUK 1854.12.4.132, four paralectotypes (d’Orbigny coll.).

####### Remarks.

None of the specimens correspond to the original figured by d’Orbigny. A lectotype—closest to the measurements given by d’Orbigny—has been chosen by Breure from among the material in the Paris museum (Breure 1975b: 1151). The current systematic position follows Richardson (1995: 145).

####### Current systematic position.

Bulimulidae, *Drymaeus (Drymaeus) linostoma* (d’Orbigny 1835).

###### Bulimus liquabilis

Reeve, 1848

http://species-id.net/wiki/Bulimus_liquabilis

Figs 73D–E , L34vi

Bulimus liquabilis Reeve 1848 [1848–1850]: pl. 57 fig. 387; Breure 1979: 76 (lectotype designation).
Rabdotus dealbatus dealbatus (Say, 1821); Thompson 2011: 127.

####### Type locality.

”Texas”.

####### Label.

”Texas”. M.C. label style IV, V.

####### Dimensions.

Not given; figured specimen herein H 21.3, D 12.3, W 6.7.

####### Type material.

NHMUK 1975422, lectotype (Cuming coll.).

####### Remarks.

The current systematic position is according to Thompson (2011).

####### Current systematic position.

Bulimulidae, *Rabdotus dealbatus dealbatus* (Say, 1821).

###### Bulimus lirinus

Morelet, 1851

http://species-id.net/wiki/Bulimus_lirinus

Figs 20A , L34viii

Bulimus lirinus Morelet 1851: 11; Breure 1979: 120.
Drymaeus (Mesembrinus) lirinus ; Thompson 2011: 113.

####### Type locality.

[Guatemala] ”Petenensium San-Luis”.

####### Label.

”Peten, Forets de S. Luis”, in Morelet’s handwriting.

####### Dimensions.

”Longit. 30.—Diam. 11 [mm]”; figured specimen herein H 30.2, D 13.15, W 6.1.

####### Type material.

NHMUK 1893.2.4.1954, holotype (ex Morelet).

####### Remarks.

Morelet indicated that he had only one specimen at hand, thus this is the holotype. The current systematic position is according to Thompson (2011).

####### Current systematic position.

Bulimulidae, *Drymaeus (Mesembrinus) lirinus* (Morelet, 1851).

###### Helix lithoica

d’Orbigny, 1835

http://species-id.net/wiki/Helix_lithoica

Figs 67A–B , 67K , L34vii

Helix lithoica d’Orbigny 1835: 13; Breure 1979: 88.
Bulimus lithoicus d’Orbigny 1837 [1834–1847]: 288, pl. 33 figs 10–11 [19 June / 7 Aug 1837; text 6 May 1838]; Gray 1854: 18.
Bulimulus (Scuatlus) lithoicus ; Crawford 1939: 328.
Scutalus culmineus lithoicus ; Breure 1975b: 1143, pl. 3 fig. 1 (lectotype designation).

####### Type locality.

”provincia Pazensi (republica Boliviana)”.

####### Label.

”La Paz. Bolivia”, in d’Orbigny’s handwriting.

####### Dimensions.

”Longit 15 millim.; latit. 35 millim. [sic, H 35, D 15 mm]”; figured specimen herein H 34.3, D 17.1, W 6.4.

####### Type material.

NHMUK 1854.12.4.197, six paralectotypes (d’Orbigny coll.).

####### Remarks.

d’Orbigny (1838 [1834–1847]: 288) specified the type locality as ”descendant de la ville de la Paz vers le petit bourg de los Obrages”; see Breure 1973: 129. The current systematic position at species level follows Richardson (1995).

####### Current systematic position.

Bulimulidae, *Kuschelenia (Kuschelenia) culminea culminea* (d’Orbigny, 1835) (**comb. n.**).

###### Bulimus lividus

Reeve, 1850

http://species-id.net/wiki/Bulimus_lividus

Figs 18C–D , L34ix

Bulimus lividus Reeve 1850 [1848–1850]: pl. 85 fig. 626; Breure 1979: 121 (lectotype designation).

####### Type locality.

”Venezuela”.

####### Label.

”Venezuela”. M.C. label style III, V.

####### Dimensions.

Not given; figured specimen herein H 24.5, D 11.22, W 6.7+.

####### Type material.

NHMUK 1975208, lectotype; 1975209, two paralectotypes (Cuming coll.).

####### Remarks.

The aperture of the lectotype has been damaged and the thin lip has been broken off; also the top is slightly damaged. We tentively follow Richardson (1995) in grouping this taxon with *Bulimus granadensis* Pfeiffer, 1848, but have not seen any type material of the latter for comparison.

####### Current systematic position.

Bulimulidae, *Drymaeus (Mesembrinus) granadensis* (Pfeiffer, 1848).

###### Bulimus lobbii

Reeve, 1849

http://species-id.net/wiki/Bulimus_lobbii

Figs 57A–C , L35i

Bulimus lobbii Reeve 1849 [1848–1850]: pl. 72 fig. 516; Reeve 1850: 98; Breure 1979: 101.
Neopetraeus lobbii ; Breure 1978: 215, fig. 365 (lectotype designation).

####### Type locality.

”Banks of the Maranon near Balsas, Peru”.

####### Label.

See remarks.

####### Dimensions.

Not given; figured specimen herein H 44.3, D 19.4, W 7.6.

####### Type material.

NHMUK 1975431, lectotype; 1975432, two paralectypes (Cuming coll.).

####### Remarks.

The locality label is no longer legible. The lectotype corresponds to Reeve 1849 [1848–1850]: pl. 72 fig. 516a.

####### Current systematic position.

Bulimulidae, *Neopetraeus lobbii* (Reeve, 1849).

###### Bulimus longinquus

Morelet, 1863

http://species-id.net/wiki/Bulimus_longinquus

Figs 11A , L35iii

Bulimus longinquus Morelet 1863: 195, pl. 11 fig. 2; Breure 1979: 55.
Bostryx longinquus ; Breure 1978: 98 (lectotype designation).

####### Type locality.

[Peru, Dept. Cuzco] ”Limatambo, Ollantaïtambo, Yucay et Piré”.

####### Label.

”Ollantaitambo Pérou”, in Morelet’s handwriting.

####### Dimensions.

”Longit. 31, diam. 12 mill.”; figured specimen herein H 30.9, D 13.8, W 7.8.

####### Type material.

NHMUK 1893.2.4.185–187, three paralectotypes (ex Morelet).

####### Remarks.

This taxon was described from several localities and with two unnamed varieties; the lectotype is in the MHNG collection. The current systematic position follows Richardson (1995).

####### Current systematic position.

Bulimulidae, *Bostryx longinquus* (Morelet, 1863).

###### Helix lophoica

d’Orbigny, 1835

http://species-id.net/wiki/Helix_lophoica

Figs 44M–O , L35ii

Helix lophoica d’Orbigny 1835: 19.
Bulimus lophoicus d’Orbigny 1837 [1834–1847]: 316, pl. 40 figs 14–15 [18 Sept. 1837; text 6 May 1838].
Drymaeus lophoicus ; Breure 1975b: 1152, pl. 1 fig. 4 (lectotype designation).

####### Type locality.

”provincia Yungacensi (republica Boliviana)”; see Breure 1973: 119.

####### Label.

”Chulumani de Yungas”; in d’Orbigny’s handwriting.

####### Dimensions.

”Longit. 33 millim., latit. 12 millim.”; figured specimen herein H 32.7, D 12.9, W 7.3.

####### Type material.

NHMUK 1854.12.4.135, three paralectotypes (d’Orbigny coll.).

####### Remarks.

The lectotype is in the MNHN collection (Breure 1975b). The current systematic position follows Richardson (1995).

####### Current systematic position.

Bulimulidae, *Drymaeus (Drymaeus) lophoicus* (d’Orbigny, 1835).

###### Otostomus loxanus

Higgins, 1872

http://species-id.net/wiki/Otostomus_loxanus

Fig. L35iv

Otostomus loxanus Higgins 1872: 685, pl. 56 figs 2–2a; Breure 1979: 121 (lectotype designation).
Drymaeus (Mesembrinus) loxanus ; Linares and Vera 2012: 196.

####### Type locality.

[Ecuador] ”Loxa” [Loja].

####### Label.

”Ecuador”. M.C. label style I.

####### Dimensions.

”Long. 29, diam. 11 mill.”; specimen found H 26.5, D 12.2, W 6.7.

####### Material.

 NHMUK 1975552, one specimen (Cuming coll.).

####### Remarks.

Higgins did not state on how many specimens his description was based; however, his material was said to be collected by Buckley. This shell from the Cuming collection cannot be traced to that collector, but corresponds to the original figure of Higgins albeit the colour has faded away; the shell height is slightly less than given by Higgins. This specimen is no longer considered to be type material and loses its status as lectotype.

###### Bulimus loxensis

Pfeiffer, 1846

http://species-id.net/wiki/Bulimus_loxensis

Figs 22I , L35v

Bulimus loxensis Pfeiffer 1846c: 85; Pfeiffer 1848b: 203; Reeve 1848 [1848–1850]: pl. 40 fig. 251; Breure 1979: 121 (lectotype designation).
Drymaeus (Mesembrinus) loxensis ; Breure and Eskens 1981: 77, pl. 7 fig. 6.

####### Type locality.

[Ecuador] ”El Catamaija prope Loxa reipublicae Aequatoris (Hartweg in coll. Cuming)”.

####### Label.

”from El Catamaija / near Loxa. Republic of / the Equator. 4 South Lat / on Bushes. Mr Hartweg”, taxon label in Pfeiffer’s handwriting. M.C. label style I, V.

####### Dimensions.

”Long. 35, diam. 14 mill.”; figured specimen herein H 34.7, D 14.2, W 7.3.

####### Type material.

NHMUK 1975553, lectotype; 1975554, two paralectotypes, Hartweg leg. (Cuming coll.).

####### Remarks.

The current systematic position follows Richardson (1995).

####### Current systematic position.

Bulimulidae, *Drymaeus (Mesembrinus) loxensis* (Pfeiffer, 1846).

###### Bulimulus (Drymaeus) lucidus

da Costa, 1898

http://species-id.net/wiki/Bulimulus_lucidus

Figs 35G–I , L35vi

Bulimulus (Drymaeus) lucidus da Costa 1898: 82, pl. 6 fig. 4; Breure 1979: 111.

####### Type locality.

”Ecuador”.

####### Label.

”Ecuador”, taxon label in da Costa’s handwriting.

####### Dimensions.

”Long. 19, diam. 11 mm.”; figured specimen herein H 18.6, D 10.7, W 4.9.

####### Type material.

NHMUK 1907.11.21.44, lectotype; 1907.11.21.45, one paralectotype, Buckley leg. (da Costa coll.).

####### Remarks.

da Costa described this taxon on the basis of material collected by Buckley, but did not state on how many specimens his description was based. The specimen figured by da Costa is selected as lectotype (**design. n.**) to define this taxon.

####### Current systematic position.

Bulimulidae, *Drymaeus (Drymaeus) lucidus* (da Costa, 1898).

###### Bulimus lucidus

Reeve, 1848

http://species-id.net/wiki/Bulimus_lucidus

Figs 20B , L35viii

Bulimus lucidus Reeve 1848 [1848–1850]: pl. 40 fig. 245; Breure 1979: 121.

####### Type locality.

[West Indies] ”St. Vincents”.

####### Label.

”St. Vincents”. M.C. label style IV, V.

####### Dimensions.

Not given; figured specimen herein H 28.9, D 14.2, W 6.3.

####### Type material.

NHMUK 1975524, three syntypes (Cuming coll.).

####### Remarks.

One of the specimens corresponds to Reeve’s figure. The current systematic position is according to Richardson (1995).

####### Current systematic position.

Bulimulidae, *Drymaeus (Mesembrinus) stramineus* (Guilding, 1824).

###### Bulimus luridus

Pfeiffer, 1863

http://species-id.net/wiki/Bulimus_luridus

Figs 9E , L35vii

Bulimus luridus Pfeiffer 1863: 274.

####### Type locality.

”New Caledonia” (see remarks).

####### Label.

”New Caledonia”, taxon label in Pfeiffer’s handwriting. M.C. label style III.

####### Dimensions.

”Long. 22, diam. 11 mill.”; figured specimen herein H 21.7, D 12.55, W 6.2.

####### Type material.

NHMUK 19991535, lectotype and one paralectotype (Cuming coll.).

####### Remarks.

This taxon, hitherto unfigured, has not been collected on New Caledonia (see also Crosse 1871: 183). Pilsbry (1896 [1895–1896]: 194) suggested that it might belong to his South American group of ‘*Bulimulus–Bostryx–Lissoacme*’ species. The protoconch is smooth and fits within *Bostryx* sensu Breure 1979 (see also Breure 2012b). The type locality is clearly in error and the species might possibly be found in northern Peru or Ecuador. The specimen marked with ‘x’ near the basal margin of the aperture is now selected lectotype (**design. n.**) to fixate this taxon.

####### Current systematic position.

Bulimulidae, *Bostryx luridus* (Pfeiffer, 1863).

###### Bulimus lusorius

Pfeiffer, 1855

http://species-id.net/wiki/Bulimus_lusorius

Figs 17A–C , L36i

Bulimus lusorius Pfeiffer, 1855c: 291; Breure 1979: 121 (lectotype designation).
Drymaeus (Mesembrinus) lusorius ; Breure and Eskens 1981: 77, pl. 7 fig. 12.
Mesembrinus lusorius ; Simone 2006: 145, fig. 487.

####### Type locality.

”Banks of Amazon, Brazils”.

####### Label.

”Brazil Banks of Ama[zon]”, taxon label in Pfeiffer’s handwriting. M.C. label style I.

####### Dimensions.

”Long. 25, diam. 10 mill.”; figured specimen herein H 24.4, D 10.84, W 5.9.

####### Type material.

NHMUK 1975543, lectotype (Cuming coll.).

####### Remarks.

The shape of the shell, aperture and the colour pattern suggest in combination that this taxon belongs to *Drymaeus* *s. str.*

####### Current systematic position.

Bulimulidae, *Drymaeus (Drymaeus) lusorius* (Pfeiffer, 1855) (**comb. n.**).

###### Bulimulus (Naesiotus) lycodus

Dall, 1917

http://species-id.net/wiki/Bulimulus_lycodus

Figs 55F , L36ii

Bulimulus (Naesiotus) lycodus Dall 1917b: 379; Breure 1979: 70.
Naesiotus lycodus ; Breure and Borrero 2008: 13.

####### Type locality.

”Galapagos, Indefatigable Island, 450–550 ft.”.

####### Label.

”Galapagos, Indefatigable Island, 450–550 ft.”.

####### Dimensions.

”Length of shell 11; diameter 8 mm”; figured specimen herein H 10.0, D 7.3, W 6.2.

####### Type material.

NHMUK 1937.6.18.11–12, two possible paratypes, CAS expedition leg. 1905–1906 (ex Schlesch).

####### Remarks.

These specimens are here considered only as possible paratypes, despite the evidence on the label. The reason is that material of *Naesiotus snodgrassi* (Dall, 1900) was found (NHMUK 1937.6.18.6–7), originating from Schlesch and designated as ‘paratypes’, which proved to be collected by the expedition of the California Academy of Sciences in 1905–1906 according to the label; for that reason it is impossible that they belonged to the type series. All type material from this source is thus viewed with much suspicion.

####### Current systematic position.

Bulimulidae, *Naesiotus lycodus* (Dall, 1917).

###### Bulimulus (Drymaeus) malleatus

da Costa, 1898

http://species-id.net/wiki/Bulimulus_malleatus

Figs 53G–I , L36iii

Bulimulus (Drymaeus) malleatus da Costa 1898: 82, pl. 6 fig. 7; Breure 1979: 11.
Drymaeus (Drymaeus) malleatus ; Breure 1978: 29.

####### Type locality.

”La Paz, Bolivia, 3,600 metres”.

####### Label.

”La Paz, 3600 Mts / Bolivia”, in da Costa’s handwriting.

####### Dimensions.

”Long. 34, diam. 15 mm.”; figured specimen herein H 34.5, D 15.5, W 6.1.

####### Type material.

NHMUK 1907.11.21.130, holotype (da Costa coll.).

####### Remarks.

This taxon was explicitly described ”from a single specimen”.

####### Current systematic position.

Bulimulidae, *Drymaeus (Drymaeus) malleatus* (da Costa, 1898).

###### Bulimus manupictus

Reeve, 1848

http://species-id.net/wiki/Bulimus_manupictus

Figs 21E , L36iv

Bulimus manupictus Reeve 1848 [1848–1850]: pl. 55 fig. 369; Breure 1979: 111 (lectotype designation).
Drymaeus (Mesembrinus) manupictus ; Breure and Eskens 1981: 77, pl. 7 fig. 7.

####### Type locality.

[Venezuela, Edo. Merida] ”Andes of Columbia” (see remarks).

####### Label.

”Merida, Columb.”. M.C. label style I, V.

####### Dimensions.

Not given; figured specimen herein H 33.3, D 14.24, W 6.1.

####### Type material.

NHMUK 1975522, lectotype (Cuming coll.).

####### Remarks.

The label reveals that this is a species from the Venezuelan Andes region of Merida, for which in the beginning of the 19^th^ century politically the name ‘Colombia’ has been applied. Contrary to a remark on a modern label from 1980s, this specimen fits the figure by Reeve. The top of the specimen is slightly damaged.

####### Current systematic position.

Bulimulidae, *Drymaeus (Mesembrinus) manupictus* (Reeve, 1848).

###### Bulimus marcidus

Pfeiffer, 1853

http://species-id.net/wiki/Bulimus_marcidus

Figs 63I–J , L36v

Bulimus marcidus Pfeiffer 1853d: 435; Pfeiffer 1854 in Küster and Pfeiffer 1840–1865: 188, pl. 49 figs 11–12; Pfeiffer 1854a: 67.

####### Type locality.

”Brasilia”.

####### Label.

”Brazil”. M.C. label style I.

####### Dimensions.

”Long. 20, diam. 8 mill.”; figured specimen herein H 18.0, D 8.4, W 5.9.

####### Type material.

NHMUK 20100649, three possible syntypes (Cuming coll.).

####### Remarks.

Pfeiffer based himself on material from Cuming’s collection, although his description was not published until 1854 (Pfeiffer 1854a). The label bears no taxon name in Pfeiffer’s handwriting, but the specimens are treated herein as possible syntypes.

####### Current systematic position.

Bulimulidae, *Bulimulus marcidus* (Pfeiffer, 1853).

###### Helix marmarina

d’Orbigny, 1835

http://species-id.net/wiki/Helix_marmarina

Figs 27G–I , L36vi

Helix marmarina d’Orbigny 1835: 18.
Bulimus marmarinus d’Orbigny 1837 [1834–1847]: 310, pl. 39 figs 11–12 [7 Aug. 1837; text 6 May 1838].

####### Type locality.

”provincia Yungacensi (republica Boliviana)”; see Breure 1973: 119.

####### Label.

”Yungas, Bolivia”; in d’Orbigny’s handwriting.

####### Dimensions.

”Longit. 42 millim., latit. 18 millim.”; lectotype H 37.9, D 18.8, W 6.7.

####### Type material.

NHMUK 1854.12.4.129, lectotype and five paralectotypes.

####### Remarks.

One specimen, corresponding to d’Orbigny 1837 [1834–1847]: pl. 39 figs 11–12, is selected lectotype; its lip is slightly damaged at the columellar-basal part. The largest specimen in the lot has a shell height of 40.7 mm. The current systematic position follows Richardson (1995: 148).

####### Current systematic position.

Bulimulidae, *Drymaeus (Drymaeus) marmarinus* (d’Orbigny, 1835).

###### Bulimus martinicensis

Pfeiffer, 1846

http://species-id.net/wiki/Bulimus_martinicensis

Figs 53B–C , L37i

Bulimus martinicensis Pfeiffer 1846b: 40; Reeve 1849 [1848–1850]: pl. 63 fig. 434.
Naesiotus martinicensis ; Breure 1975a: 85, pl. 8 figs 5–8, 10; Breure 1979: 70.

####### Type locality.

”island of Martinique”.

####### Label.

”Martinique”, taxon label in Pfeiffer’s handwriting. M.C. label style IV, V.

####### Dimensions.

”Long. 20, diam. 8 mill.”; figured specimen herein H 19.5, D 8.14, W 7.1.

####### Type material.

NHMUK 197451, lectotype; 197452, two paralectotypes (ex Petit, Cuming coll.).

####### Remarks.

Pfeiffer described this species on the basis of material from Petit. One of the labels is nearly illegible, but corresponds to the handwriting of Petit (cf. Breure 2013: figs 1B, 11ii, 12i). This taxon is transferred to the genus *Protoglyptus* Pilsbry, 1897 based on data presented by Breure and Romero (2012).

####### Current systematic position.

Bulimulidae, *Protoglyptus martinicensis* (Pfeiffer, 1846) (**comb. n.**).

###### Bulimus mejillonensis

Pfeiffer, 1857

http://species-id.net/wiki/Bulimus_mejillonensis

Figs 8C , L37iii

Bulimus mejillonensis Pfeiffer in Pfeiffer and Dunker 1857: 230; Breure 1979: 56.
Bostryx mejillonensis ; Breure 1978: 102 (lectotype designation).

####### Type locality.

[Chile] ”Mejillones in deserto Atacamensi”.

####### Label.

”Desert of Atacama”, taxon label in Pfeiffer’s handwriting. M.C. label style III.

####### Dimensions.

”Long. 25, diam. 12 mill.”; figured specimen herein H 24.1, D 12.8, W 6.2.

####### Type material.

NHMUK 1975322, lectotype; 1975323, two paralectotypes (Cuming coll.).

####### Remarks.

Pfeiffer did not state on how many specimens his description was based. The current systematic position follows Richardson (1995).

####### Current systematic position.

Bulimulidae, *Bostryx mejillonensis* (Pfeiffer, 1857).

###### Bulimus meleagris

Pfeiffer, 1853

http://species-id.net/wiki/Bulimus_meleagris

Figs 75D–F , L37ii

Bulimus meleagris Pfeiffer 1853b: 257; 1853d: 382; Pfeiffer 1854 in Küster and Pfeiffer 1840–1865: 81, pl. 21 figs 24–25.

####### Type locality.

”Andibus Novae Granadae”.

####### Label.

”Andes N. Granada”, taxon label in Pfeiffer’s handwriting. M.C. label style I.

####### Dimensions.

”Long. 31, diam. 14 mill.”; figured specimen herein H 31.3, D 16.0, W 5.0.

####### Type material.

NHMUK 20100583, lectotype and one paralectotype (Cuming coll.).

####### Remarks.

One of the specimens corresponds to the original measurements and Pfeiffer’s figure, and is here designated lectotype (**design. n.**). The paralectotype is subadult. Upon re-examining these specimens, it is clear that the specimens referred to by Breure (1978: 219, pl. 7 fig. 18) are not this species. If the type locality is correct, this species might be expected in southern Colombia or the adjacent region in Ecuador.

####### Current systematic position.

Bulimulidae, *Stenostylus meleagris* (Pfeiffer, 1853).

###### Bulimus meridanus

Pfeiffer, 1846

http://species-id.net/wiki/Bulimus_meridanus

Figs 22J , L37iv

Bulimus meridanus Pfeiffer 1846a: 33; Reeve 1848 [1848–1850]: pl. 57 fig. 386; Breure 1979: 121.

####### Type locality.

[Venezuela] ”Merida, Andes of Bolivia [sic]”.

####### Label.

”New Granada”. taxon label in Pfeiffer’s handwriting. M.C. label style III, V.

####### Dimensions.

”Long. 29, diam. 11 mill.”; figured specimen herein H 35.9, D 14.0, W 7.3.

####### Type material.

NHMUK 1975523, two syntypes (Cuming coll.).

####### Remarks.

Pfeiffer did not state on how many specimens his description was based. The material has a taxon label in Pfeiffer’s handwriting and is considered as syntypes. One of the specimens corresponds to Reeve’s figure.

####### Current systematic position.

Bulimulidae, *Drymaeus (Mesembrinus) meridanus* (Pfeiffer, 1846).

###### Bostryx metagyra

Pilsbry & Olsson, 1949

http://species-id.net/wiki/Bostryx_metagyra

Figs 1K , L37v

Bostryx metagyra Pilsbry and Olsson 1949: 9, fig. 12; Baker 1963: 229; Neubert and Janssen 2004: 217, pl. 7 fig. 83; Breure 2011: 35.

####### Type locality.

”Peru”.

####### Label.

”without locality, from the original series”.

####### Dimensions.

”Height 8.2 mm., diameter 8.6 mm.”; figured specimen herein H 7.2, D 7.6, W 4.9.

####### Type material.

NHMUK 20100630, four probable paratypes (ex Weyrauch).

####### Remarks.

Pilsbry and Olsson (1949: 10) remarked ”paratypes 184900 ANSP and in Museo Historia Natural, Lima, where many specimens were preserved without locality”. As it is known that Weyrauch worked in the Lima museum during many years in the 1940s–1950s (Barbosa et al. 2008), and Weyrauch exchanged many shells, these specimens are considered probable paratypes. Of the supposedly five specimens originally sent by Weyrauch, one is missing and two are broken.

####### Current systematic position.

Bulimulidae, *Bostryx metagyra* Pilsbry & Olsson, 1949.

###### Bostryx (Bostryx) haasi minor

Weyrauch, 1960

http://species-id.net/wiki/Bostryx_haasi_minor

Fig. 3H

Bostryx (Bostryx) haasi minor Weyrauch, 1960a: 35, pl. 5 fig. 34; Breure 1979: 56; Neubert and Janssen 2004: 217, pl. 9 fig. 102.

####### Type locality.

[Peru] ”Mittel-Peru, am Westhang der westlichen Anden: auf der rechten Seite des Río Rimac, gegenüber der Selterswasserquelle San Mateo (3300 m)”.

####### Label.

”C-Peru, on the right side of Rio Rimac, above San Mateo, 3300 m”; printed label.

####### Dimensions.

”H 19–23.5, D 10.5”. Figured specimen herein H 14.0, D 5.2, W 7.9.

####### Type material.

NHMUK 1975353, ten paratypes (ex Weyrauch).

####### Current systematic position.

Bulimulidae, *Bostryx haasi* Weyrauch, 1960.

###### Bulinus modestus

Broderip in Broderip and Sowerby I 1832

http://species-id.net/wiki/Bulinus_modestus

Fig. 6G

Bulinus modestus Broderip in Broderip and Sowerby I 1832b: 106; Sowerby I 1833 [Sowerby I and II 1832–1841]: fig. 19.

####### Type locality.

”Peruviae montibus, Huacho”.

####### Label.

Lost, see remarks.

####### Dimensions.

”Long. 1 2/8, lat. 4/8 poll. [H 31.6, D 12.66 mm]”; figured specimen herein H 31.6, D 13.8, W 8.6.

####### Type material.

NHMUK 20120232, four possible syntypes (Cuming coll.).

####### Remarks.

Broderip described this taxon from the Cuming collection, but did not state on how many specimens his description was based. The label is lost, but considering this material is from the Cuming collection and the specimen figured herein corresponds with the original measurements, the material is considered as possible syntypes.

####### Current systematic position.

Bulimulidae, *Bostryx modestus* (Broderip in Broderip and Sowerby I 1832).

###### Bulimus mollicellus

Reeve, 1849

http://species-id.net/wiki/Bulimus_mollicellus

Figs 60I–J , L37vi

Bulimus mollicellus Reeve 1849 [1848–1850]: pl. 77 fig. 565; Breure 1979: 63.
Bulimulus mollicellus ; Breure 1978: 146 (lectotype designation).

####### Type locality.

”—?”.

####### Label.

See remarks. M.C. label style III, V.

####### Dimensions.

Not given; figured specimen herein H 17.2, D 7.88, W 5.8.

####### Type material.

NHMUK 1975185, lectotype; 1975186, one paralectotype (Cuming coll.).

####### Remarks.

Reeve did not state on how many specimens his description was based; of the two specimens the smaller corresponds to Reeve’s figure. Lubomirski (1880: 725) identified a shell collected by Jelski in Peru, Dept. Junín, Tarma as this species (”*Bulimus (Leptomerus) molecillus* [sic]”, an error in the name which has been copied by Pilsbry 1897 [1897–1898]: 63). The voucher material is known to exist in the Warsaw museum (D. Mierzwa, pers. commun.); a photograph suggests a conspecific identification for this specimen, which would thereby disclose the region of occurrence for this taxon.

####### Current systematic position.

Bulimulidae, *Bulimulus mollicellus* (Reeve, 1849).

###### Bulimus monachus

Pfeiffer, 1857

http://species-id.net/wiki/Bulimus_monachus

Figs 63A–B , L37vii

Bulimus monachus Pfeiffer 1857c: 333.

####### Type locality.

”Meobamba, Peru”.

####### Label.

”Meobamba M. Gueinzius”, taxon label in Pfeiffer’s handwriting. M.C. label style I.

####### Dimensions.

”Long. 31, diam. 11 1/2 mill.”; figured specimen herein H 31.2, D 12.6, W 8.3.

####### Type material.

NHMUK 20100584, lectotype and two paralectotypes, Gueinzius leg. (Cuming coll.).

####### Remarks.

The specimen which is nearest to the original shell height is herein selected lectotype (**design. n.**) to define this taxon.

####### Current systematic position.

Bulimulidae, *Bulimulus monachus* (Pfeiffer, 1857).

###### Bulimulus (Bostryx) moniezi

Dautzenberg, 1896

http://species-id.net/wiki/Bulimulus_moniezi

Figs 1B , L37viii

Bulimulus (Bostryx) moniezi Dautzenberg 1896: 224, pl. 7 fig. 3; Breure 1979: 56; Breure 2011: 35, figs 11G, 11vii.

####### Type locality.

”le Haut-Pérou”.

####### Label.

”Andes of Bolivia” (handwriting of E.A. Smith?).

####### Dimensions.

”Longit. 14 millim., latit. 6 1/2 millim.”; figured specimen herein H 12.5, D 5.9, W 7.9.

####### Type material.

NHMUK 1908.6.13.22, one possible syntype (ex Preston, ex Dautzenberg?).

####### Remarks.

The specimen is labelled ”co-type” but is not accompanied by a Dautzenberg label; however, its type status is not questioned here as Dautzenberg is known to have been in regular contact with Preston and the London museum (Duchamps 1999, Breure unpublished data). The locality on the label is clearly in error (cf. Breure 2011).

####### Current systematic position.

Bulimulidae, *Bostryx moniezi* (Dautzenberg, 1896).

###### Bulimus monilifer

Reeve, 1848

http://species-id.net/wiki/Bulimus_monilifer

Figs 23F , L38i

Bulimus monilifer Reeve 1848 [1848–1850]: pl. 48 fig. 318.
Bulimus indistinctus Pfeiffer 1852: 63. New name for *Bulimus monilifer* Reeve, 1848 not *Bulimus moniliferus* Gould, 1846.

####### Type locality.

”—?”.

####### Label.

”—?”. M.C. label style IV.

####### Dimensions.

Not given; figured specimen herein H 26.3, D 13.22, W 5.7.

####### Type material.

NHMUK 1975403, lectotype; 1975404, one paralectotypes (Cuming coll.).

####### Remarks.

Pfeiffer (1852) introduced a new name for Reeve’s taxon, but since the name of Gould (1846: 99) is sufficiently distinct (*Bulimus moniliferus*, now in *Amphidromus*), this was an unneccesary nomenclatural act. All later authors (see Richardson 1995: 138) have followed Pfeiffer in this error, which is here corrected.

####### Current systematic position.

Bulimulidae, *Drymaeus (Mesembrinus) monilifer* (Reeve, 1848).

###### Bulimus montagnei

d’Orbigny, 1837

http://species-id.net/wiki/Bulimus_montagnei

Figs 12D , L37ix

Bulimus montagnei d’Orbigny 1837 [1834–1847]: 286, pl. 32 figs 5–7 [3 April 1837; text 6 May 1838]; Gray 1854: 17.

####### Type locality.

[Bolivia] ”principalement à la côte de Petaca [Dept. Santa Cruz, Cuevas Petacas; see Breure 1973]”.

####### Label.

”côte de Petaca Valle grande (Bolivia)”, in d’Orbigny’s handwriting.

####### Dimensions.

”Long. 21 millim.; lat. 9 mill.”; figured specimen herein H 23.3, D 9.4, W 8.0.

####### Type material.

NHMUK 1854.12.4.194, lectotype and seven paralectotype (d’Orbigny coll.).

####### Remarks.

d’Orbigny did not state on how many specimens his description was based. One of the specimens corresponds to d’Orbigny 1837 [1834–1847]: pl. 32 figs 5–6, and is here selected lectotype (**design. n.**). The protoconch is smooth and relatively small; this taxon—hitherto classified with *Drymaeus*—is now transferred to the genus *Bostryx* Troschel, 1847 (**comb. n.**).

####### Current systematic position.

Bulimulidae, *Bostryx montagnei* (d’Orbigny, 1837).

###### Bulimus montevidensis

Pfeiffer, 1846

http://species-id.net/wiki/Bulimus_montevidensis

Figs 64G–H , L38ii

Bulimus montevidensis Pfeiffer 1846a: 33; Pfeiffer 1848b: 202; Reeve 1848 [1848–1850]: pl. 19 fig. 114; Breure 1979: 63.
Bulimulus bonariensis (Rafinesque, 1833); Miquel 1991: 98, figs 23–24.

####### Type locality.

”Montevideo, Buenos Ayres”.

####### Label.

”Montevideo & Buenos Ayres”, taxon label in Pfeiffer’s handwriting. M.C. label style IV.

####### Dimensions.

”Long. 28, diam. 12 mill.”; figured specimen herein H 34.2, D 17.0, W 7.7.

####### Type material.

NHMUK 1975401, four syntypes (Cuming coll.).

####### Remarks.

The current systematic position is according to Miquel (1991).

####### Current systematic position.

Bulimulidae, *Bulimulus bonariensis bonariensis* (Rafinesque, 1833).

###### Helix montivaga

d’Orbigny, 1835

http://species-id.net/wiki/Helix_montivaga

Figs 15J , L38iv

Helix montivaga d’Orbigny 1835: 14.
Bulimus montivagus d’Orbigny 1837 [1834–1847]: 275, pl. 34 figs 1–3 [3 April 1837; text 23 April 1838]; Gray 1854: 15.
Naesiotus montivagus ; Breure 1975b: 1146.

####### Type locality.

[Bolivia] ”provincia Lagunensis (republica Boliviana)”; see remarks.

####### Label.

”pampa ruis; Laguna. Bolivia” [1854.12.4.167], ”pampa grande Bolivia” [1854.12.4.168], ”[..] Grande S^ta^ Cruz Bolivia” [1854.12.4.169], ”[illegible]” [1854.12.4.170], all labels in d’Orbigny’s handwriting.

####### Dimensions.

”Longit. 16 millim.; latit. 7 millim.”; figured specimen herein H 20.5, D 7.45, W 8.3.

####### Type material.

NHMUK 1854.12.4.167, eight paralectotypes; 1854.12.4.168, seven juvenile paralectotypes; 1854.12.4.169, five paralectotypes; 1854.12.4.170, lectotype and five paralectotypes; 1854.12.4.172, five paralectotypes (d’Orbigny coll.).

####### Remarks.

d’Orbigny (1835) originally also mentioned ”et provincia Entre-Rios (republica Argentina)” as type locality. The type locality was restricted to ”Bolivia, Dept. Santa Cruz” by Breure (1975b: 1146); see also Breure 1973. Miquel (1989a: 62) concluded that this species does not occur in Argentina. One of the syntypes from lot 1854.12.4.170, corresponding to d’Orbigny 1837 [1834–1847]: pl. 34 fig. 2, is here designated as lectotype (**design. n.**). One specimen each is missing from lots 1854.12.4.167 resp. 1854.12.4.169.

####### Current systematic position.

Bulimulidae, *Naesiotus montivagus* (d’Orbigny, 1835).

###### Bostryx mordani

Breure, 1978

http://species-id.net/wiki/Bostryx_mordani

Fig. 4D

Bostryx mordani Breure 1978: 103, pl. 3 fig. 3.

####### Type locality.

”Peru, Dept. Lima, Río Santa Eulalia valley, 3 km above Autisha, 2500 m, sta. 67”.

####### Label.

”67. Peru, Dept. Lima, Río Santa Eulalia valley, 3 km above Autisha, 2500 m”.

####### Dimensions.

”shell height 23.5, diameter 9.1 mm”; figured specimen herein H 22.5, D 8.9, W 7.7.

####### Type material.

NHMUK 1975266, paratype, A.S.H. Breure leg., 1.ii.1975.

####### Current systematic position.

Bulimulidae, *Bostryx mordani* Breure, 1978

###### Bulimus moricandi

Pfeiffer, 1847

http://species-id.net/wiki/Bulimus_moricandi

Figs 22H , L38v

Bulimus moricandi Pfeiffer 1847: 113; Reeve 1848 [1848–1850]: pl. 40 fig. 283; Breure 1979: 121 (lectotype designation).
Drymaeus (Mesembrinus) moricandi ; Breure and Eskens 1981: 78, figs 247–248; Thompson 2011: 118.

####### Type locality.

[Guatemala] ”Mount Coban, Central America (*Lattre*)”.

####### Label.

”M. Lattre / Coban C Am[erica]”. M.C. label style III, V.

####### Dimensions.

”Lat. 24, diam. 12 mill.”; figured specimen herein H 23.6, D 12.7, W 5.8.

####### Type material.

NHMUK 1975212, lectotype (Cuming coll.).

####### Remarks.

Pfeiffer did not state on how many specimens his description was based. The lectotype, however, matches the original measurements and also corresponds to Reeve’s figure. The current systematic position is after Thompson (2011).

####### Current systematic position.

Bulimulidae, *Drymaeus (Mesembrinus) moricandi moricandi* (Pfeiffer, 1847).

###### Otostomus moritinctus

Martens, 1893

http://species-id.net/wiki/Otostomus_moritinctus

Figs 54I , L39i

Otostomus moritinctus Martens 1893 [1890–1901]: 228, pl. 14 figs 9–10; Breure 1979: 121 (lectotype designation); Neubert and Janssen 2004: 218, pl. 16 fig. 191.
Drymaeus (Mesembrinus) moritinctus ; Breure and Eskens 1981: 78; Köhler 2007: 152, fig. 128; Thompson 2011: 120.

####### Type locality.

”W. Mexico: Chilpancingo, in the State of Guerrero, at an elevation of 4600 feet above the sea (*H.H. Smith*)”.

####### Label.

”Chilpancingo”.

####### Dimensions.

”Long. 26–29, diam. 13 millim.”; figured specimen herein H 25.2, D 12.8, W 5.6.

####### Type material.

NHMUK 1901.6.22.841, lectotype; 1901.6.22.842–847, 851–852, eight paralectotypes, H.H. Smith leg. (Godman coll.).

####### Remarks.

The current systematic position follows Thompson (2011).

####### Current systematic position.

Bulimulidae, *Drymaeus (Mesembrinus) moritinctus* (Martens, 1893).

###### Bulimulus (Drymaeus) mossi

E.A. Smith, 1896

http://species-id.net/wiki/Bulimulus_mossi

Figs 21B , L39iv

Bulimulus (Drymaeus) mossi E.A. Smith 1896: 243, pl. 8 fig. 8.

####### Type locality.

[Trinidad].

####### Label.

”Trinidad”.

####### Dimensions.

”Longit. 21 1/2 millim., diam. 9 1/2”; figured specimen herein H 21.8, D 9.7, W 5.5.

####### Type material.

NHMUK 1912.5.11.1, holotype, W. Moss leg.

####### Remarks.

Smith mentioned that he examined a single specimen.

####### Current systematic position.

Bulimulidae, *Drymaeus (Mesembrinus) mossi* (E.A. Smith, 1896).

###### Bulimus moussoni

Pfeiffer, 1853

http://species-id.net/wiki/Bulimus_moussoni

Figs 23L , L39ii

Bulimus moussoni Pfeiffer 1853a: 147; Breure 1979: 121 (lectotype designation).
Drymaeus (Mesembrinus) moussoni ; Breure and Eskens 1981: 78.

####### Type locality.

”St. Domingo (*Sallé*)”.

####### Label.

”Haiti”, taxon label in Pfeiffer’s handwriting. M.C. label style III.

####### Dimensions.

”Long. 26, diam. 12 mill.”; figured specimen herein H 26.0, D 12.9, W 6.2.

####### Type material.

NHMUK 1975210, lectotype; 1975211, two paralectotypes, Sallé leg. (Cuming coll.).

####### Remarks.

Pfeiffer did not state on how many specimens his description was based. The published type locality could be interpreted as located in the Dominican Republic, while the label indicates Haiti; he did, however, mention Sallé as collector.

####### Current systematic position.

Bulimulidae, *Drymaeus (Mesembrinus) moussoni* (Pfeiffer, 1853).

###### Bulimus muliebris

Reeve, 1849

http://species-id.net/wiki/Bulimus_muliebris

Figs 18K–L , L39iii

Bulimus muliebris Reeve 1849 [1848–1850]: pl. 81 fig. 598.
Drymaeus muliebris ; Linares and Vera 2012: 197.

####### Type locality.

”New Granada”.

####### Label.

”New Granada”.

####### Dimensions.

Not given; figured specimen herein H 29.9, D 13.06, W 6.6.

####### Type material.

NHMUK 1879.2.26.251, lectotype (ex Taylor).

####### Remarks.

Reeve did not mention on how many specimens his description was based. The sole specimen corresponds to Reeve’s figure and is here designated lectotype (**design. n.**). The current systematic position as a sepatate taxon follows Richardson (1995). This specimen has been recognized in material from Colombia, Dept. Santander, Los Santos (Breure and Borrero, unpublished data), as first precise locality for this taxon.

####### Current systematic position.

Bulimulidae, *Drymaeus (Mesembrinus) muliebris* (Reeve, 1849).

###### Drymaeus multispira

da Costa, 1904

http://species-id.net/wiki/Drymaeus_multispira

Figs 11C , L39vi

Drymaeus multispira da Costa 1904: 5, pl. 1 fig. 4; Breure 1979: 122 (lectotype designation).

####### Type locality.

”Chuco Chaca, Bolivia, 4,000 feet”.

####### Label.

”Chuco Chaca / Bolivia 4000 ft”, in da Costa’s handwriting.

####### Dimensions.

”Long. 20, diam. 11 mm.”; figured specimen herein H 19.9, D 11.11, W 6.5.

####### Type material.

NHMUK 1907.11.21.31, lectotype (da Costa coll.).

####### Remarks.

da Costa wrote ”the shells of this species”, implying that he had seen more than one specimen. Breure (1979) referred to this (subadult) specimen as ”HT BMNH 1907.21.31”; under Art. 74.6 ICZN Breure’s designation may be considered as a lectotype designation. The protoconch is sculptured with very indistinct spiral lines and some granules; the taxon is here transferred to *Bostryx* sensu Breure 1979 (**comb. n.**); see also Breure 2012b. It is now considered as a subjective junior synonym of *Helix torallyi* d’Orbigny, 1835 (**syn. n.**).

####### Current systematic position.

Bulimulidae, *Bostryx torallyi* (d’Orbigny, 1835).

###### Bulimus munsterii

d’Orbigny, 1837

http://species-id.net/wiki/Bulimus_munsterii

Figs 74D , L39v

Bulimus munsterii d’Orbigny 1837 [1834–1847]: 278, pl. 34 figs 4–7 [3 April 1837; text 23 April 1838]; Gray 1854: 16.
Naesiotus munsterii ; Breure, 1975b: 1147 (lectotype designation); Miquel 1989a: 67, figs 7–8.

####### Type locality.

”la côte de Petaca (...) dans les plaines de Santa-Cruz de la Sierra, en Bolivia”; see Breure 1973.

####### Label.

”cote de Petaca S^ta^ Cruz”, in d’Orbigny’s handwriting.

####### Dimensions.

”Longueur, 23 millimètres; largeur, 10 millimètres”; figured specimen herein H 21.7, D 10.85, W 8.2.

####### Type material.

NHMUK 1854.12.11.177, six paralectotypes (d’Orbigny coll.).

####### Remarks.

This taxon was published with a wrong name (*Helix camba*) in the legend of the plate (as noticed and corrected by d’Orbigny in his text and on the label). According to Coan et al. (2013a: 33) d’Orbigny’s plate 34 was published on 3 April 1837 and his text on 23 April 1838. The correct date of publication of the taxon is therefore 1837. The figured specimen herein corresponds to d’Orbigny 1837 [1834–1847]: pl. 34 figs 5–6. The current systematic position is according to Miquel 1989a.

####### Current systematic position.

Bulimulidae, *Naesiotus munsterii* (d’Orbigny, 1837).

###### Bulimus murrinus

Reeve, 1848

http://species-id.net/wiki/Bulimus_murrinus

Figs 38J–L , L40i

Bulimus murrinus Reeve 1848 [1848–1850]: pl. 43 fig. 273b; Breure 1979: 111 (lectotype designation).
Drymaeus murrinus ; Linares and Vera 2012: 188 [partim].

####### Type locality.

”Santa Fé de Bogota”.

####### Label.

”Marinata / New Granada”, taxon label also in Pfeiffer’s handwriting. M.C. label style I.

####### Dimensions.

Not given; figured specimen herein H 37.1, D 16.2, W 6.8.

####### Type material.

NHMUK 1975213, lectotype (Cuming coll.).

####### Remarks.

The specimen designated lectotype by Breure (1979) corresponds to Reeve 1848 [1848–1850]: pl. 43 fig. 273b only. The shell corresponding to Reeve’s fig. 273a has not been found, but appears to be a distinct taxon. As a consequence *Bulimus murrinus* has been misinterpreted by most authors, and all references in literature have to be viewed with this in mind. This interpretation of *Bulimus murrinus* is based on data not yet published by Breure and Borrero.

####### Current systematic position.

Bulimulidae, *Drymaeus (Drymaeus) murrinus* (Reeve, 1848).

###### Bulimus musivus

Pfeiffer, 1855

http://species-id.net/wiki/Bulimus_musivus

Figs 29J–L , L39vi

Bulimus musivus Pfeiffer 1855d: 95, pl. 31 fig. 3; Breure 1979: 111 (lectotype designation).
Drymaeus (Drymaeus) strigatus (Sowerby I, 1833); Breure and Eskens 1981: 41.

####### Type locality.

”Meobamba, Eastern Peru (*Mr. Yates*)”.

####### Label.

”Meobamba Eastern / Peru M Yates”, taxon label in Pfeiffer’s handwriting. M.C. label style I.

####### Dimensions.

”Long. 22, diam. 9 1/2 mill.”; figured specimen herein H 21.5, D 11.9, W 6.3.

####### Type material.

NHMUK 1975292, lectotype; 1975293, two paralectotypes, Yates leg. (Cuming coll.).

####### Remarks.

The current systematic position follows Richardson (1995).

####### Current systematic position.

Bulimulidae, *Drymaeus (Drymaeus) strigatus* (Sowerby I, 1833).

###### Bulimus myristicus

Reeve, 1849

http://species-id.net/wiki/Bulimus_myristicus

Figs 57G–I , L40ii

Bulimus myristicus Reeve 1849 [1848–1850]: pl. 72 fig. 520; Breure 1979: 101 (lectotype designation).

####### Type locality.

”Andes of Caxamarca, Peru”.

####### Label.

”Andes of Caxamarca, Peru”. M.C. label style IV.

####### Dimensions.

Not given; figured specimen herein H 40.3, D 17.6, W 8.1.

####### Type material.

NHMUK 1975433, lectotype, W. Lobb leg. (Cuming coll.).

####### Remarks.

The current systematic position is following Richardson (1995), *Bulimus decussatus* Reeve, 1849 having page priority; in Richardson’s index this taxon is erroneously grouped with *Neopetraeus cora* (d’Orbigny, 1835).

####### Current systematic position.

Bulimulidae, *Neopetraeus decussatus* (Reeve, 1849).

###### Bulimus nanus

Reeve, 1849

http://species-id.net/wiki/Bulimus_nanus

Figs 2G–H , L40iv

Bulimus nanus Reeve 1849 [1848–1850]: pl. 70 fig. 585; Breure 1979: 56.

####### Type locality.

”Chili”.

####### Label.

”Chili”. M.C. label style I.

####### Dimensions.

Not given; figured specimen herein H 15.4, D 4.75, W 7.8.

####### Type material.

NHMUK 1975463, six syntypes (Cuming coll.).

####### Remarks.

The material was found with the specimens still glued on cardboard; one specimen was removed and is figured herein. Rehder (1945: 106) provided a new name for *Bulimus nanus* Reeve, 1849 non Lamarck, 1804. The current systematic position is according to Stuardo and Vega (1985).

####### Current systematic position.

Bulimulidae, *Bostryx pumilio* Rehder, 1945.

###### Bulimus (Otostomus) napo

Angas, 1878

http://species-id.net/wiki/Bulimus_napo

Figs 47G–I , L40iii

Bulimus (Otostomus) napo Angas 1878b: 312, pl. 18 figs 4–5; Breure 1979: 111.
Drymaeus (Drymaeus) quadrifasciatus (Angas, 1878); Breure and Borrero 2008: 22.

####### Type locality.

”Ecuador”.

####### Label.

”Ecuador”.

####### Dimensions.

”Alt. 1 inch 3 lines, diam. 5 1/2 lines [H 31.7, D 11.6 mm]”; figured specimen herein H 31.3, D 15.0, W 6.4.

####### Type material.

NHMUK 1879.1.21.4, lectotype (ex Angas).

####### Remarks.

As Angas did not state on how many specimens his description was based, the reference of Breure (1979) to ”HT 1879.1.21.4” has to be interpreted as a lectotype designation under Art. 74.6 ICZN. The current systematic position follows Breure and Borrero (2008).

####### Current systematic position.

Bulimulidae, *Drymaeus (Drymaeus) quadrifasciatus* (Angas, 1878).

###### Bulimus nigrofasciatus

Pfeiffer in Philippi 1846

http://species-id.net/wiki/Bulimus_nigrofasciatus

Figs 21J , L40v–vi

Bulimus nigrofasciatus Pfeiffer in Philippi 1846 [1845–1847]: 125, pl. 5 fig. 7; Reeve 1848 [1848–1850]: pl. 55 fig. 379.
Mesembrinus nigrofasciatus ; Simone 2006: 146, fig. 488.
Drymaeus nigrofasciatus ; Linares and Vera 2012: 198 [partim].

####### Type locality.

”Novae Granadae, vallis Magdalenae”.

####### Label.

”Brazil” and ”Venezuela” (see remarks), taxon label in Pfeiffer’s handwriting. M.C. label style IV / I.

####### Dimensions.

”Long. 12´´´. Diam. 6´´´ [H 26.1, D 13.0 mm]”; figured specimen herein H 23.5, D 12.95, W 6.2.

####### Type material.

NHMUK 1975542, lectotype and two paralectotypes; 20120333, one probable syntype (Cuming coll.).

####### Remarks.

According to the labels of lot NHMUK 1975542, the specimens would have originated from Brazil. This is probably an erroneous Cumingian label, as the species has only been found in Colombia (Breure and Borrero, unpublished data). A label stating ”*Bul. helena* Quoy” and a second, illegable, label have supposedly been added afterwards. Another label makes clear that one of the specimens was figured by Reeve (1848). As the taxon label is written by Pfeiffer, there is little doubt about the type status of the specimens in this lot. The shell figured by Simone (2006) is a juvenile syntype. Another lot, NHMUK 20120333, was found with locality ”Venezuela” (striked-through in pencil), of which the largest specimen is said to originate from the Cuming collection. As this lot also has a taxon label in Pfeiffer’s handwriting, the specimen is considered another probable syntype. It is assumed that the original measurements were given in German lines. The largest specimen in lot NHMUK 1975542 is here designated lectotype (**design. n.**) to fixate this taxon, which seems to have been misinterpreted in most cases (Breure and Borrero, unpublished data).

####### Current systematic position.

Bulimulidae, *Drymaeus (Mesembrinus) nigrofasciatus* (Pfeiffer in Philippi 1846).

###### Bulimus nigrolimbatus

Pfeiffer, 1853

http://species-id.net/wiki/Bulimus_nigrolimbatus

Figs 75A–C , L40vii

Bulimus nigrolimbatus Pfeiffer 1853b: 257; Pfeiffer 1853 in Küster and Pfeiffer 1840–1865: 81, pl. 21 figs 26–30.
Stenostylus nigrolimbatus ; Breure 1978: 219 (lectotype designation); Breure 2008: 247, figs 11–13.

####### Type locality.

”Andes of New Granada”.

####### Label.

”Andes, N. Granada”, taxon label in Pfeiffer’s handwriting. M.C. label style IV.

####### Dimensions.

”Long. 28, diam. 10 mill.”; figured specimen herein H 28.0, D 16.4, W 4.8.

####### Type material.

NHMUK 1975549, lectotype; 197550, one paralectotype (Cuming coll.).

####### Remarks.

Breure (2008) has shown that the type specimens are subadults. The species has been found in Colombia, Dept. Santander, Páramaro de Almorzadero, to which the type locality may be restricted (**restr. n.**).

####### Current systematic position.

Bulimulidae, *Stenostylus nigrolimbatus* (Pfeiffer, 1853).

###### Bulimus nigropileatus

Reeve, 1849

http://species-id.net/wiki/Bulimus_nigropileatus

Figs 5C , L41i

Bulimus nigropileatus Reeve 1849 [1848–1850]: pl. 73 fig. 724 (text no. 725); Breure 1979: 56.
Bostryx nigropileatus ; Breure 1978: 104 (lectotype designation).

####### Type locality.

”Chachapoyas, Alto-Peru”.

####### Label.

No locality given. M.C. label style I.

####### Dimensions.

Not given; figured specimen herein H 20.5, D 10.25, W 7.0.

####### Type material.

NHMUK 1975335, lectotype; 1975336, two paralectotypes (Cuming coll.).

####### Remarks.

The specimens were said by Reeve to have been collected by W. Lobb and were described from the Cuming collection; the type status is herein not disputed.

####### Current systematic position.

Bulimulidae, *Bostryx nigropileatus* (Reeve, 1849).

###### Bulimus nitelinus

Reeve, 1849

http://species-id.net/wiki/Bulimus_nitelinus

Figs 22F , L41ii

Bulimus nitelinus Reeve 1849 [1848–1850]: pl. 59 fig. 398.

####### Type locality.

”Mexico”.

####### Label.

”Mexico”. M.C. label style I.

####### Dimensions.

Not given; figured specimen herein H 29.4, D 14.0, W 6.3.

####### Type material.

NHMUK 20100570, lectotype (Cuming coll.).

####### Remarks.

Reeve did not state on how many specimens his description was based. The single specimen, with the colours faded on the ventral side, corresponds to his figure and is here selected lectotype (**design.n.**). The current systematic position is following Richardson (1995: 177).

####### Current systematic position.

Bulimulidae, *Drymaeus (Mesembrinus) serperastrus* (Say, 1830).

###### Bulinus nitidus

Broderip in Broderip and Sowerby I 1832

http://species-id.net/wiki/Bulinus_nitidus

Figs 23M , L41iii

Bulinus nitidus Broderip in Broderip and Sowerby I 1832a: 31; Sowerby I 1833 [Sowerby I and II 1832–1841]: fig. 2; Breure 1979: 122.
Bulimus nitidus ; Reeve 1848 [1848–1850]: pl. 18 figs 103a–b.

####### Type locality.

”in Peruviâ”.

####### Label.

”Peru”. M.C. label style IV.

####### Dimensions.

”long. 1 1/5, lat. 4/5 poll.” [H 30.4, D 20.2 mm]; figured specimen herein H 28.8, D 13.0, W 6.8.

####### Type material.

NHMUK 1975551, five possible syntypes; 1842.5.10.135–138, four + two possible syntypes (see remarks) (Cuming coll.).

####### Remarks.

Broderip described his taxon from the Cuming collection, but did not specify on how many specimens his description was based. Two lots are considered possible syntypes. Lot 1842.5.10.135–138 contains four specimens, with a note by FGN[aggs] that labels had been stuffed inside; also two specimens, which were apparently glued upon the same cardboard, have been added to this lot. Lot 1975551 has been used for the figure in Reeve 1848 [1848–1850]: pl. 18 figs 103a–b, and has also a taxon label in Pfeiffer’s handwriting. Both lots are labelled ”Peru”. It is unclear upon which shells the record of ”Columbia” in Reeve (1848 [1848–1850]) is based. The current systematic position follows Richardson (1995).

####### Current systematic position.

Bulimulidae, *Drymaeus (Mesembrinus) cactivorus* Broderip in Broderip and Sowerby I 1832).

###### Helix nivalis

d’Orbigny, 1835

http://species-id.net/wiki/Helix_nivalis

Fig. L41iv

Helix nivalis d’Orbigny 1835: 12.
Bulimus nivalis ; d’Orbigny 1837 [1834–1847]: 287, pl. 32 figs 8–9 [3 April 1837; text 6 May 1838]; Gray 1854: 18.
Scutalus nivalis ; Breure 1975b: 1143 (lectotype designation).

####### Type locality.

Not given; ”Potosi (Bolivia)”, in d’Orbigny 1838 [1834–1847]: 288.

####### Label.

”Potosi, Bolivia”, in d’Orbigny’s handwriting.

####### Dimensions.

Not given; see remarks.

####### Type material.

NHMUK 1854.12.4.144, one paralectotype (d’Orbigny coll.).

####### Remarks.

The type locality was mentioned for the first time by d’Orbigny 1838 [1834–1847]; he also specified the measurements: ”Long. 14 millim.; lat. 9 millim.”. The single specimen is badly damaged and therefore is not figured here. This taxon is probably a juvenile *Kuschelenia* species.

####### Current systematic position.

Bulimulidae, *Kuschelenia*. Nomen inquirendum.

###### Drymaeus notabilis

da Costa, 1906

http://species-id.net/wiki/Drymaeus_notabilis

Figs 32J–L , L41v

Drymaeus notabilis da Costa 1906a: 7, pl. 1 fig. 2; Breure 1979: 112; Linares and Vera 2012: 188.

####### Type locality.

”Antioquia, Colombia”.

####### Label.

”Antioquia / Colombia”, in da Costa’s handwriting.

####### Dimensions.

”Long. 33, diam. 17 mm.”; figured specimen herein H 32.8, D 16.8, W 6.0.

####### Type material.

NHMUK 1907.11.21.5, lectotype (da Costa coll.).

####### Remarks.

Da Costa did not mention on how many specimens his description was based; therefore, the reference of Breure (1979) to ”HT 1907.11.21.5” has to be interpreted as a lectotype designation under Art. 74.6 ICZN.

####### Current systematic position.

Bulimulidae, *Drymaeus (Drymaeus) notabilis* (da Costa, 1906).

###### Drymaeus notatus

da Costa, 1906

http://species-id.net/wiki/Drymaeus_notatus

Figs 32G–I , L41vi

Drymaeus notatus da Costa 1906a: 7, pl. 1 fig. 3; Breure 1979: 112; Linares and Vera 2012: 189.

####### Type locality.

”Antioquia, Colombia”.

####### Label.

”Antioquia”, in da Costa’s handwriting.

####### Dimensions.

”Long. 34.5, diam. 16 mm.”; figured specimen herein H 34.5, D 15.4, W 6.5.

####### Type material.

NHMUK 1907.11.21.6, lectotype; 1907.11.21.7, one paralectotype (da Costa coll.).

####### Remarks.

da Costa did not mention on how many specimens his description was based; in his remarks he wrote ”this shell”, but as there were two specimens in the type lot, this cannot be interpreted as reference to a single shell. The reference of Breure (1979) to ”HT 1907.11.21.6” has to be interpreted as a lectotype designation under Art. 74.6 ICZN.

####### Current systematic position.

Bulimulidae, *Drymaeus (Drymaeus) notatus* (da Costa, 1906).

###### Bulimus nubeculatus

Pfeiffer, 1853

http://species-id.net/wiki/Bulimus_nubeculatus

Figs 63E–F , L41vii

Bulimus nubeculatus Pfeiffer 1853b: 257; Breure 1979: 63 (lectotype designation).
Bulimulus corneus nubeculatus ; Thompson 2011: 106.

####### Type locality.

”America centrali (*Morelet*)”.

####### Label.

”Central Americ[a]”, taxon label in Pfeiffer’s handwriting. M.C. label style I.

####### Dimensions.

”Long. 16, diam. 8 1/2 mill.”; figured specimen herein H 16.0, D 9.12, W 5.8.

####### Type material.

NHMUK 1975407, lectotype; 1975408, two paralectotypes (Cuming coll.).

####### Remarks.

This taxon is regarded as a questionable variety of *Bulimulus corneous* (Sowerby I, 1833) by Martens (1893 [1890–1901]: 247) and Pilsbry (1897 [1897–1898]: 56. The exact type locality remains unknown. For the current systematic position we prefer, therefore, to follow Richardson (1995: 68) who treated Pfeiffer’s taxon as a subjective junior synonym of Sowerby’s species.

####### Current systematic position.

Bulimulidae, *Bulimulus corneous* (Sowerby I, 1833).

###### Bulimulus (Drymaeus) nubilus

Preston, 1903

http://species-id.net/wiki/Bulimulus_nubilus

Figs 54J–K , L42i

Bulimulus (Drymaeus) nubilus Preston 1903: 4, text-fig.; Breure 1979: 112.

####### Type locality.

”Azarhar de Cartago, Costa Rica”.

####### Label.

”Azarhar de Cartago, / Costa Rica”.

####### Dimensions.

”Diam. maj. 10.5, alt. 25 millim.”; figured specimen herein H 22.3, D 10.45, W 6.2.

####### Type material.

NHMUK 1903.5.4.1, lectotype (ex Preston).

####### Remarks.

Preston did not mention on how many specimens his description was based. The single specimen in the NHMUK is not accompanied by a label in Preston’s handwriting, but its type status is here not disputed as the specimen originates from Preston. The record of Breure (1979) of ”HT 1903.5.4.1” has to be interpreted as a lectotype designation under Art. 74.6 ICZN. The current systematic position follows (Richardson 1995: 170).

####### Current systematic position.

Bulimulidae, *Drymaeus (Mesembrinus) recluzianus* (Pfeiffer, 1847).

###### Bulimus nucinus

Reeve, 1850

http://species-id.net/wiki/Bulimus_nucinus

Figs 70G–H , L42ii

Bulimus nucinus Reeve 1850 [1848–1850]: pl. 85 fig. 625; Breure 1979: 86.
Scutalus (Vermiculatus) nucinus ; Breure 1978: 180 (lectotype designation).

####### Type locality.

”—?”.

####### Label.

”—?”. M.C. label style IV.

####### Dimensions.

Not given; figured specimen herein H 36.8, D 18.1, W 6.2.

####### Type material.

NHMUK 1975379, lectotype (Cuming coll.).

####### Remarks.

Reeve did not state on how many specimens his description was based, but mentioned the specimens were from Cuming’s collection. The current systematic position is according to Richardson (1995).

####### Current systematic position.

Bulimulidae, *Kuschelenia (Vermiculatus) nucinus* (Reeve, 1850).

###### Bulimus nucula

Pfeiffer, 1853

http://species-id.net/wiki/Bulimus_nucula

Figs 14G , L42iii

Bulimus nucula Pfeiffer 1853d: 415; Pfeiffer 1854a: 60; Breure 1979: 70.
Naesiotus nuculus ; Breure and Coppois 1978: 178 (lectotype designation).

####### Type locality.

[Ecuador] ”insulis Gallapagos”.

####### Label.

”Galapagos”. M.C. label style III.

####### Dimensions.

”Long. 11 1/2, diam. 6 mill.”; figured specimen herein H 11.25, D 5.62, W 6+.

####### Type material.

NHMUK 1975155, lectotype; 1975156, three paralectotypes (Cuming coll.).

####### Remarks.

The top of the lectotype is slightly damaged.

####### Current systematic position.

Bulimulidae, *Naesiotus nucula* (Pfeiffer, 1853).

###### Bulinus nux

Broderip, 1832

http://species-id.net/wiki/Bulinus_nux

Figs 14A , L42iv

Bulinus nux Broderip 1832: 125; Sowerby I 1833 [Sowerby I and II 1832–1841]: figs 37–37*; Reeve 1848 [1848–1850]: pl. 23 fig. 150; Breure 1979: 70.
Naesiotus nux ; Breure and Coppois 1978: 178 (lectotype designation); Breure and Borrero 2008: 14.

####### Type locality.

”Insula Gallapagos. (Charles’s Island)”.

####### Label.

”Charles I. Galapagos”. M.C. label style III.

Dimensions. ”long. 10/12, lat. 9/12 poll.” [H 21.1, D 18.9]; figured specimen herein H 21.5, D 12.06, W 7.2.

####### Type material.

NHMUK 1975172, lectotype: 1975173, three paralectotypes (Cuming coll.).

####### Remarks.

The material is accompanied by labels denoting its figuration in Reeve (1848–1850) and taxon labels in Pfeiffer’s handwriting, one ”*Bul. nux*” and one ”*Bul. unifasciatus*”. All labels seem to have been glued together during E.A. Smith’s period, who has marked on the board both the locality and the Cumingian origin. The H/D ratio as given by Broderip is probably erroneous.

####### Current systematic position.

Bulimulidae, *Naesiotus nux* (Broderip, 1832).

###### Bulimus nystianus

Pfeiffer, 1853

http://species-id.net/wiki/Bulimus_nystianus

Figs 39J–L , L42v

Bulimus nystianus Pfeiffer 1853d: 374; Pfeiffer 1854 in Küster and Pfeiffer 1840–1865: 99, pl. 32 figs 15–16; Pfeiffer 1854c: 154; Breure 1979: 112 (lectotype designation).
Drymaeus (Drymaeus) nystianus ; Breure and Borrero 2008: 21.

####### Type locality.

”in valle Pomasqui reipublicae Aequatoris (*Bourcier*)”.

####### Label.

”Valley of / Pomasqui, Equador / Mons^r^ Bourcier / Consul General”. M.C. label style IV.

####### Dimensions.

”Long. 32, diam. 15 mill.”; figured specimen herein H 31.9, D 16.5, W 5.8.

####### Type material.

NHMUK 1975573, lectotype; 1975574, two paralectotypes, Bourcier leg. (Cuming coll.).

####### Current systematic position.

Bulimulidae, *Drymaeus (Drymaeus) nystianus* (Pfeiffer, 1853).

###### Drymaeus obliquistriatus

da Costa, 1901

http://species-id.net/wiki/Drymaeus_obliquistriatus

Figs 8E , L42vi

Drymaeus obliquistriatus da Costa 1901: 238, pl. 24 fig. 2; Breure 1979: 136.

####### Type locality.

”San Pablo, Peru”.

####### Label.

”San Pablo, Peru”, in da Costa’s handwriting.

####### Dimensions.

”Long. 30, diam. 12 mm.”; figured specimen herein H 28.9, D 12.7, W 7.8.

####### Type material.

NHMUK 1907.11.21.41, lectotype (da Costa coll.).

####### Remarks.

The type locality remains unspecified, as there are several localities named ”San Pablo” throughout Peru. Da Costa did not mention on how many specimens his description was based. The reference of Breure (1979) to ”HT 1907.11.21.41” has to be interpreted as a lectotype designation under Art. 74.6 ICZN. The protoconch of the specimen is smooth, and the shape is like those found in *Bostryx* sensu Breure 1979; thefore, the taxon is now transferred to this genus (**comb. n.**). There is a mismatch in the taxon name between the label and the published name.

####### Current systematic position.

Bulimulidae, *Bostryx obliquistriatus* (da Costa, 1901) (**comb. n.**).

###### Bulimus ochraceus

Morelet, 1863

http://species-id.net/wiki/Bulimus_ochraceus

Figs 71E–F , 71J , L42viii

Bulimus ochraceus Morelet 1863: 176, pl. 7 fig. 6; Breure 1979: 86.
Scutalus (Vermiculatus) ochraceus ; Breure 1978: 180 (lectotype designation).

####### Type locality.

[Peru, Dept. Cuzco] ”à Soraï et à Salcantaï”.

####### Label.

”Pérou. Soraï”, in Morelet’s handwriting.

####### Dimensions.

”Long. 37–40; diam. 17–18 mill.”; figured specimen herein H 36.0, D 18.5, W 5.3.

####### Type material.

NHMUK 1893.2.4.164, lectotype; 1893.2.4.165–166, two paralectotypes (ex Morelet).

####### Remarks.

Morelet remarked that this taxon occurs in the lower part of ‘puna brava’, i.e. at high elevations. The type locality is thus at the slopes of Nevado Salcantay and near Soray, which is at ca. 4100 m elevation in Dept. Cuzco, to which the type locality is now restricted.

####### Current systematic position.

Bulimulidae, *Kuschelenia (Vermiculatus) ochracea* (Morelet, 1863) (**comb. n.**).

###### Bulimus (Drymaeus) ochrocheilus

E.A. Smith, 1877

http://species-id.net/wiki/Bulimus_ochrocheilus

Figs 48A–C , L42vii

Bulimus (Drymaeus) ochrocheilus E.A. Smith 1877: 364, pl. 39 fig. 5; Breure 1979: 112.
Drymaeus (Drymaeus) ochrocheilus ; Breure and Borrero 2008: 22.

####### Type locality.

”Malacatos, South Ecuador”.

####### Label.

”Malacatos, S. Ecuador”, in E.A. Smith’s handwriting.

####### Dimensions.

”Long. 37 mill., diam. 13”; figured specimen herein H 37.1, D 14.2, W 6.8.

####### Type material.

NHMUK 1877.3.28.4, lectotype.

####### Remarks.

Smith did not mention on how many specimens his description was based. The reference of Breure (1979) to ”HT 1877.3.28.4” has to be interpreted as a lectotype designation under Art. 74.6 ICZN.

####### Current systematic position.

Bulimulidae, *Drymaeus (Drymaeus) ochrocheilus* (E.A. Smith, 1877).

###### Bulimus orbignyi

Pfeiffer, 1846

http://species-id.net/wiki/Bulimus_orbignyi

Figs 15D , L43i

Bulimus orbignyi Pfeiffer 1846a: 31; Breure 1979: 64 (lectotype designation).

####### Type locality.

”Locality unknown”.

####### Label.

”Bolivia”, taxon label in Pfeiffer’s handwriting. M.C. label style IV.

####### Dimensions.

”Long. 19, diam. 8 mill.”; figured specimen herein H 18.5, D 9.1, W 6.6.

####### Type material.

NHMUK 1975349, lectotype; 1975350, two paralectotypes (Cuming coll.).

####### Remarks.

In his original publication Pfeiffer stated the type locality as unknown. In Pfeiffer (1848b: 208) he quoted ”prope Lima (De Lattre)”. This may have led Pilsbry (1896 [1895–1896]: 162) to put this taxon into the synonymy of *Bulinus modestus* Broderip, 1832. However, as the material has the taxon label in Pfeiffer’s handwriting, and the measurements closely agree with those originally given, the type status is here not disputed. It is assumed therefore that this is a Bolivian taxon. Breure (1979) classified this taxon with *Bulimulus* Leach, 1814. The protoconch sculpture consists of rather straight axial riblets, which is atypical for this genus. Pfeiffer’s taxon is now transferred to *Naesiotus* sensu Breure 1979 (**comb. n.**).

####### Current systematic position.

Bulimulidae, *Naesiotus orbignyi* (Pfeiffer, 1846).

###### Helix oreades

d’Orbigny, 1835

http://species-id.net/wiki/Helix_oreades

Figs 54L–M , L43ii

Helix oreades d’Orbigny 1835: 11.
Bulimus oreades ; d’Orbigny 1837 [1834–1847]: 270, pl. 31 figs 11–12 [? before 27 Feb. 1837; text 23 April 1838]; Gray 1854: 15.
Mesembrinus oreades ; Simone 2006: 146, fig. 489.
Drymaeus oreades ; Cuezzo et al. 2013: 155.

####### Type locality.

”provinvia Corrientes (republica Argentina)”.

####### Label.

”corrientes Rep. argentina”, in d’Orbigny’s handwriting.

####### Dimensions.

”Longit. 32 millim.; latit. 7 millim.”; figured specimen herein H 31.6, D 14.1, W 6.4.

####### Type material.

NHMUK 1854.12.4.161, lectotype and five paralectotypes (d’Orbigny coll.).

####### Remarks.

The type locality has been specified in d’Orbigny 1838 [1834–1847]: 270 as ”la rive sud du Rio de Santa-Lucia, dans les environs de San-Roque”; see Breure 1973: 120, fig. 8. According to Miquel (1989b: 76) this taxon does not occur in Argentina, but in Brasil. The diameter as given by d’Orbigny (1835) seems to be erroneous, also seen the H/D ration in his figure (d’Orbigny 1837 [1834–1847]: pl. 31 figs 11–12); the publication date of this plate is not given in the collation of Coan et al. (2013a), but is herein assumed to be 1837. The specimen corresponding to this figure is selected lectotype (**design.n.**). The shell height given by Simone (2006) is erroneous.

####### Current systematic position.

Bulimulidae, *Drymaeus (Mesembrinus) oreades* (d’Orbigny, 1835).

###### Bulimus orophilus

Morelet, 1860

http://species-id.net/wiki/Bulimus_orophilus

Figs 4H , L43iii

Bulimus orophilus Morelet 1860: 374; Morelet 1863: 189, pl. 9 fig. 6; Breure 1979: 56.
Bostryx orophilus ; Breure 1978: 107 (lectotype designation).

####### Type locality.

”les vallées tempérées des plateaux de Cuzco; (..) notablement à Talavera, Silque, Incahuasi et Mollepata” (restricted to Peru, Dept. Cuzco, Prov. Anta, Distr. Limatambo, Mollepata; Breure 1978).

####### Label.

”Pérou, Mollepata” [.188–190], ”Pérou, Ollantaytambo” [.182–184], ”Pérou, Talavera” [.191], all in Morelet’s handwriting.

####### Dimensions.

”Long. 22; diam. 9 mill.”; figured specimen herein H 20.8, D 10.5, W 6+.

Type material. NHMUK 1893.2.4.188, lectotype; 1893.2.4.189–190, two paralectotypes; 1893.2.4.182–184, three paralectotypes; 1893.2.4.191, one paralectotypes (ex Morelet).

####### Remarks.

Morelet described this taxon based on various specimens and described also two colour forms. The current systematic position follows Richardson (1995).

####### Current systematic position.

Bulimulidae, *Bostryx orophilus* (Morelet, 1860).

###### Bulimus (Drymaeus) orthostoma

E.A. Smith, 1877

http://species-id.net/wiki/Bulimus_orthostoma

Figs 29A–C , L43iv

Bulimus (Drymaeus) orthostoma E.A. Smith 1877: 364, pl. 39 fig. 5; Breure 1979: 112.
Drymaeus (Drymaeus) orthostomus [sic]; Breure and Borrero 2008: 22.

####### Type locality.

”Ecuador?”.

####### Label.

”Ecuador”. M.C. label style III.

####### Dimensions.

”Long. 37 mill., diam. 11 1/2”; figured specimen herein H 36.5, D 13.05, W 6.5.

####### Type material.

NHMUK 1975132, lectotype; 1975133, two paralectotypes, Buckley leg. (Cuming coll.).

####### Remarks.

Smith did not mention that he based himself on a shell from the Cuming collection; the question mark in his published type locality may be associated with this source (see Breure and Ablett 2011: 5). Also the number of specimens on which the description was based was not mentioned by Smith; therefore, the reference of Breure (1979) to ”HT 1975132” has to be interpreted as a lectotype designation under Art. 74.6 ICZN. The current systematic position follows Richardson (1995).

####### Current systematic position.

Bulimulidae, *Drymaeus (Drymaeus) orthostoma* (E.A. Smith, 1877).

###### Bulimus pallens

Reeve, 1849

http://species-id.net/wiki/Bulimus_pallens

Figs 20J , L43v

Bulimus pallens Reeve 1849 [1848–1850]: pl. 62 fig. 423.

####### Type locality.

”—?”.

####### Label.

No locality given. M.C. label style II.

####### Dimensions.

Not given; figured specimen herein H 17.9, D 11.25, W 4.9.

####### Type material.

NHMUK 20120056, one syntype (Cuming coll.).

####### Remarks.

The shell corresponds with Reeve’s figure; its protoconch sculpture equals those found in *Drymaeus* species. The shell is a juvenile, but at present its systematic specific position cannot be ascertained. This taxon has not been mentioned in monographs (e.g., Pilsbry 1902) or catalogues (e.g., Richardson 1995).

####### Current systematic position.

Bulimulidae, *Drymaeus*. Nomen inquirendum.

###### Bulimulus (Naesiotus) pallidus

Reibisch, 1892

http://species-id.net/wiki/Bulimulus_pallidus

Figs 13D , L43vi

Bulimulus (Naesiotus) pallidus Reibisch 1892: 18, pl. 1 fig. 9.

####### Type locality.

”Albemarle-Island (Wolf)”.

####### Label.

”Albemarle Is Galapagos”.

####### Dimensions.

”Long. 12.7, diam maj. 6.6 mm”; figured specimen herein H 11.37, D 5.59, W 6.6.

####### Type material.

NHMUK 1894.5.8.3, syntype, Theodor Wolf leg.

####### Remarks.

Reibisch based his description on four shells, of which only one was fully adult. The current systematic position follows Richardson (1995: 222).

####### Current systematic position.

Bulimulidae, *Naesiotus jacobi* (Sowerby I, 1833).

###### Bulinus panamensis

Broderip, 1832

http://species-id.net/wiki/Bulinus_panamensis

Figs 18Q , L44i

Bulinus panamensis Broderip in Broderip and Sowerby I 1832b: 105; Sowerby I 1833 [Sowerby I and II 1832–1841]: fig. 25.

####### Type locality.

”Sinu Panamae. (King’s and Saboga Islands.)”.

####### Label.

”Panama”, taxon label in Broderip’s handwriting (?). M.C. label style I.

####### Dimensions.

”long. 1, lat. 1/2 poll.” [H 25.3, D 12.6 mm]; figured specimen herein H 18.2, D 11.0, W 6.2.

####### Type material.

NHMUK 20100614, six possible syntypes (Cuming coll.).

####### Remarks.

The lot is accompanied by a label—possibly added in the 1980s—stating ”The writing on the old label [Broderip’s handwriting?] on back of board corresponds with that on labels on material purchased from H. Cuming in 1842. Possible type”. The current systematic position is according to Thompson (2011).

####### Current systematic position.

Bulimulidae, *Drymaeus (Mesembrinus) translucens panamensis* (Broderip in Broderip and Sowerby I 1832).

###### Bulimus papillatus

Morelet, 1860

http://species-id.net/wiki/Bulimus_papillatus

Figs 8E , L44ii

Bulimus papillatus Morelet 1860: 372; Morelet 1863: 186, pl. 8 fig. 2; Breure 1979: 56.
Bostryx rhodolarynx papillatus ; Breure 1978: 116.

####### Type locality.

”[intimâ Peruvii regionae]” (see remarks).

####### Label.

”Pérou, Pucra”, in Morelet’s handwriting.

####### Dimensions.

”Longit 25; diam. 1 1/4 [sic, 14] mill.”; figured specimen herein H 22.7, D 18.0, W 6.2.

####### Type material.

NHMUK 1893.2.4.192–194, three syntypes (ex Morelet).

####### Remarks.

Morelet (1863) emended the brief diagnoses published in 1860, and specified the type locality as ”notamment à Pucra”; this locality could not be found in gazetteers. The current systematic position follows Breure (1978); the classification by Richardson (1995: 241) with *Neopetraeus* seems to be erroneous.

####### Current systematic position.

Bulimulidae, *Bostryx rhodolarynx papillatus* (Morelet, 1860).

###### Bulimus paposensis

Pfeiffer, 1856

http://species-id.net/wiki/Bulimus_paposensis

Figs 9F–H , L44iii

Bulimus paposensis Pfeiffer 1856c: 207; Breure 1979: 56.
Bostryx paposensis ; Köhler 2007: 133, fig. 29 (lectotype designation).

####### Type locality.

[Chili] ”Paposo in deserto Atacamensi reipublicae Chilensis”.

####### Label.

”Atacama”, taxon label in Pfeiffer’s handwriting. M.C. label style IV.

####### Dimensions.

”Long. 18, diam. 9 1/2 mill.”; figured specimen herein H 21.2, D 10.3, W 6.4.

####### Type material.

NHMUK 1975311, four paralectotypes (Cuming coll.).

####### Remarks.

Pfeiffer described his taxon on the basis of specimens collected by R.A. Philippi. Part of this material reached the ZMB via Albers (Köhler 2007), but apparently part of the material ended up in the Cuming collection. The current systematic position follows Köhler (2007).

####### Current systematic position.

Bulimulidae, *Bostryx paposensis* (Pfeiffer, 1856).

###### Bulimus patasensis

Pfeiffer, 1858

http://species-id.net/wiki/Bulimus_patasensis

Figs 56J–L , L44iv

Bulimus patasensis Pfeiffer 1858a: 257, pl. 42 fig. 6; Breure 1979: 101.
Neopetraeus patasensis ; Breure 1978: 216 (lectotype designation).

####### Type locality.

”Province of Patas, Andes of Peru (*Dr. Farris*)”.

####### Label.

”Province of Patas, / Andes of Peru / D^r^. Farris”, taxon label in Pfeiffer’s handwriting. M.C. label style IV.

####### Dimensions.

”Long. 47, diam. 24 mill.”; figured specimen herein H 46.8, D 27.7, W 7.1.

####### Type material.

NHMUK 1975439, lectotype; 1975440, two paralectotypes (Cuming coll.).

####### Remarks.

The current systematic position follows Richardson (1995).

####### Current systematic position.

Bulimulidae, *Neopetraeus patasensis* (Pfeiffer, 1858).

###### Bulimus patricius

Reeve, 1849

http://species-id.net/wiki/Bulimus_patricius

Figs 75H–I , L44v

Bulimus patricius Reeve 1849 [1848–1850]: pl. 81 fig. 600.
Drymaeus (Drymaeus) lilacinus (Reeve, 1849); Breure and Eskens 1981: 29 (lectotype designation); Thompson 2011: 111.

####### Type locality.

”—?”.

####### Label.

”New Granada ?” (see remarks).

####### Dimensions.

Not given; figured specimen herein H 52.1, D 25.0, W 8.1.

####### Type material.

NHMUK 1874.12.11.220, lectotype (T. Lombe Taylor coll.).

####### Remarks.

In his remarks in the text to his plate, Reeve added ”probably from New Granada, but I have no authority for stating it to be the locality”; the taxon was described from ”Mus. Taylor”. The current systematic position follows Richardson (1995: 144) and Thompson (2011), who recognises this as a Guatemalan species.

####### Current systematic position.

Bulimulidae, *Drymaeus (Drymaeus) lilacinus* (Reeve, 1849).

###### Bostryx spiculatus paucicostatus

Breure, 1978

http://species-id.net/wiki/Bostryx_spiculatus_paucicostatus

Fig. 3D

Bostryx spiculatus paucicostatus Breure 1978: 125, figs 183–186; Breure 1979: 56.

####### Type locality.

”Peru, Dept. Cuzco, 2.8 km SE Pisac, 2980 m”.

####### Label.

”102: Peru, Dept. Cuzco, 2.8 km SE Pisac, 2980 m”.

####### Dimensions.

”H 16.52 D 4.81”; figured specimen herein H 16.5, D 5.0, W 8.0.

####### Type material.

NHMUK 1975267, ten paratypes, A.S.H. Breure leg., 15.ii.1975.

####### Current systematic position.

Bulimulidae, *Bostryx spiculatus paucicostatus* Breure, 1978.

###### Helix paziana

d’Orbigny, 1835

http://species-id.net/wiki/Helix_paziana

Figs 73I–J , L45i

Helix paziana d’Orbigny 1835: 12.
Bulimus paziana d’Orbigny 1837 [1834–1847]: 286, pl. 32 figs 10–11 [3 April 1837; text 6 May 1838].
Naesiotus pazianus ; Breure 1975b: 1147, pl. 8 fig. 1.

####### Type locality.

[Bolivia] ”provincia Sicasica (republica Boliviana)”; see remarks.

####### Label.

Two labels, ”Cavari Sicasica (Bolivia)” and ”rio Miguilla Yungas Bolivia”, in in d’Orbigny’s handwriting.

####### Dimensions.

”Longit. 27 millim.; long. 11 millim.”, corrected to ”Longit. 25 millim.; lat. 11 millim.” in d’Orbigny 1838 [1834–1847]: 286; figured specimen herein H 24.3, D 12.8, W 6.8.

####### Type material.

NHMUK 1854.12.4.196, eight paralectotypes (d’Orbigny coll.).

####### Remarks.

The type locality refers to the environs of Sicasica, prov. Aroma, Dept. La Paz. In d’Orbigny 1838 [1834-1847] two varieties were mentioned: var. A. ”dans la province de Sicasica, près du bourg de Cavari” (Breure 1973: 129, fig. 7), and var. B. ”sur les coteaux du Rio de Meguilla, entre la ville de Lauza [sic, Laura] et Carcuato [sic, Circuata], province de Yungas” (Breure 1973: 129, fig. 7). Breure (1975b) found in the MNHN collection only material from Cavari, thus var. A; the shell of this variety appeared to belong to *Naesiotus*. In the NHMUK collection, the specimens from the same locality (1854.12.4.195) prove to be a *Bulimulus* species; this lot is now excluded from the type series. The protoconch of the specimens in the second lot (1854.12.4.196) show that these can be unambiguously classified in *Naesiotus*.

####### Current systematic position.

*Naesiotus pazianus* (d’Orbigny, 1835).

###### Scutalus (Vermiculatus) peaki

Breure, 1978

http://species-id.net/wiki/Scutalus_peaki

Figs 71A–B , 71I

Scutalus (Vermiculatus) peaki Breure 1978: 180, figs 308–313, pl. 10 fig. 20; Breure 1979: 86.

####### Type locality.

”Peru, Dept. Ancash, 20 km [W] Huaráz, 3750 m”.

####### Label.

”Peru, Dept. Ancash, 20 km W Huaráz, 3750 m”.

####### Dimensions.

”H 25.6 D 15.27”; figured specimen herein H 23.5, D 15.7, W 5.8.

####### Type material.

NHMUK 1975579, three paratypes, F.G. Thompson leg., 31.iii.1971.

####### Current systematic position.

Bulimulidae, *Kuschelenia (Vermiculatus) peaki* (Breure, 1978) (**comb. n.**).

###### Drymaeus expansus perenensis

da Costa, 1901

http://species-id.net/wiki/Drymaeus_expansus_perenensis

Figs 54A–B , L45ii

Drymaeus expansus perenensis da Costa 1901: 239, pl. 24 fig. 5; Breure 1979: 112.

####### Type locality.

”Peréné, Peru” [Dept. Junín, Perené].

####### Label.

”Peréné, Peru”, in da Costa’s handwriting.

####### Dimensions.

”Long. 46, lat. 23 mm.”; figured specimen herein H 45.8, D 23.4, W 6.7.

####### Type material.

NHMUK 1907.11.21.39, lectotype, C. Codd leg. (da Costa coll.).

####### Remarks.

da Costa did not mention on how many specimens his description was based. The reference of Breure (1979) to ”HT 1907.11.21.39” has to be interpreted as a lectotype designation under Art. 74.6 ICZN.

####### Current systematic position.

Bulimulidae, *Drymaeus (Drymaeus) expansus perenensis* da Costa, 1901.

###### Bulimulus pergracilis

Rolle, 1904

http://species-id.net/wiki/Bulimulus_pergracilis

Figs 54G , L45iii

Bulimulus pergracilis Rolle 1904: 37.
Drymaeus (Drymaeus) pergracilis ; Breure 1979: 112.

####### Type locality.

”Huancabamba in Peru”.

####### Label.

”Huancabamba / Peru”, in Rolle’s handwriting.

####### Dimensions.

”Alt. 35, diam. max. 12 mm.”; figured specimen herein H 33.2, D 11.0, W 7.8.

####### Type material.

NHMUK 1922.2.4.33, lectotype (ex Rolle).

####### Remarks.

Rolle did not mention on how many specimens his description was based. The reference of Breure (1979) to ”HT 1922.2.4.33” has to be interpreted as a lectotype designation under Art. 74.6 ICZN. See also remarks on locality and publication date under *Bulimulus (Drymaeus) abruptus* Rolle, 1904.

####### Current systematic position.

Bulimulidae, *Drymaeus (Drymaeus) pergracilis* (Rolle, 1904).

###### Bulimus perspectivus

Pfeiffer, 1846

http://species-id.net/wiki/Bulimus_perspectivus

Figs 13I , L45iv

Bulimus perspectivus Pfeiffer 1846a: 33; Pfeiffer 1848b: 97; Reeve 1849 [1848–1850]: pl. 63 fig. 435; Breure 1979: 71.
Naesiotus perspectivus ; Breure and Coppois 1978: 181 (lectotype designation); Breure and Borrero 2008: 14.

####### Type locality.

”Locality unknown”.

####### Label.

”Galapagos Is.” (in E.A. Smith’s handwriting), taxon label in Pfeiffer’s handwriting. M.C. label style III, V.

####### Dimensions.

”Long. 16, diam. 6 1/2 mill.”; figured specimen herein H 15.7, D 6.34, W 6.8.

####### Type material.

NHMUK 1975166, lectotype; 1975176, two paralectotypes (Cuming coll.).

####### Remarks.

Pfeiffer described this taxon from Cuming’s collection, but did not state on how many specimens his description was based. Reibisch (1892: 19) reported this taxon for the first time from a precise locality, ”Chatham Island, Galapagos”, on account of specimens collected by Th. Wolf.

####### Current systematic position.

Bulimulidae, *Naesiotus perspectivus* (Pfeiffer, 1846).

###### Bulimus pervariabilis

Pfeiffer, 1853

http://species-id.net/wiki/Bulimus_pervariabilis

Figs 21I , L45iv

Bulimus pervariabilis Pfeiffer 1853d: 337; Pfeiffer 1854a: 59; Breure 1979: 122 (lectotype designation).
Drymaeus (Mesembrinus) perviabilis ; Breure and Eskens 1981: 81, pl. 6 fig. 3.
Drymaeus pervariabilis ; Linares and Vera 2012: 199 [partim].

####### Type locality.

”Columbia”.

####### Label.

”New Granada”, taxon label in Pfeiffer’s handwriting. M.C. label style IV.

####### Dimensions.

”Long. 33, diam. 14 1/2 mill.”; figured specimen herein H 33.3, D 17.1, W 6.7.

####### Type material.

NHMUK 1975547, lectotype; 1975548, two paralectotypes (Cuming coll.).

####### Remarks.

Pfeiffer gave ”Columbia” [= Colombia] as type locality; the Cumingian material is, however, labelled as ”New Granada”, which may refer to a broader area than Colombia. During a revision of Colombian *Drymaeus* species, Breure and Borrero were unable to recognize this taxon in the material studied (unpublished data); the data in Linares and Vera (2012) have to viewed with suspicion.

####### Current systematic position.

Bulimulidae, *Drymaeus (Mesembrinus) perviabilis* (Pfeiffer, 1853).

###### Bulimus pervius

Pfeiffer, 1853

http://species-id.net/wiki/Bulimus_pervius

Figs 64C–D , L45vi

Bulimus pervius Pfeiffer 1853d: 651; 1854b: 50; Breure 1979: 64.
Bulimulus pervius ; Breure 1978: 146 (lectotype designation).

####### Type locality.

”—?”.

####### Label.

No locality given. Taxon label in Pfeiffer’s handwriting. M.C. label style IV.

####### Dimensions.

”Long. 24, diam. 12 mill.”; figured specimen herein H 24.3, D 14.0, W 6.4.

####### Type material.

NHMUK 1975165, lectotype (Cuming coll.).

####### Remarks.

No locality was given in both papers of Pfeiffer. According to Duncan (1937) the paper of Pfeiffer (1853–1854) appeared in two parts, p. 48 on December 13, 1853 and p. 49–53 on March 22, 1854. Breure (1978) suggested that this taxon may prove to be related ot identical to *Bulimulus riisei* (Pfeiffer, 1855); in the latter case *Bulimulus pervius* will have priority.

####### Current systematic position.

Bulimulidae, *Bulimulus pervius* (Pfeiffer, 1853).

###### Bulimus pessulatus

Reeve, 1848

http://species-id.net/wiki/Bulimus_pessulatus

Figs 64A–B , L46i

Bulimus pessulatus Reeve 1848 [1848–1850]: pl. 23 fig. 153; Pilsbry 1896 [1895–1896]: 188, pl. 51 fig. 4; Breure 1979: 64.
Bulimulus apodemetes (d’Orbigny, 1835); Breure 1978: 141 (lectotype designation); Cuezzo et al. 2013: 149.

####### Type locality.

”Santa Cruz de la Sierra, Bolivia”.

####### Label.

”Bolivia”. M.C. label style IV.

####### Dimensions.

Not given; figured specimen herein H 21.6, D 13.45, W 5.5.

####### Type material.

NHMUK 1975313, lectotype; 1975314, two paralectotypes (Cuming coll.).

####### Remarks.

This taxon was described by Reeve on the basis of material collected by Bridges in the Cuming collection. The collector is not mentioned on the label, but the specimen confirms to Reeve’s figure and its type status is not disputed here. The current systematic position is according to Pilsbry (1896 [1895–1896]) and Miquel (1991), but takes into account the new combination made for this taxon.

####### Current systematic position.

Bulimulidae, *Bostryx apodemetes* (d’Orbigny, 1835).

###### Bulimus petenensis

Morelet, 1851

http://species-id.net/wiki/Bulimus_petenensis

Figs 63M–N , L45vii

Bulimus petenensis Morelet 1851: 10; Breure 1979: 64 (lectotype designation); Neubert and Janssen 2004: 222, pl. 10 fig. 108.
Bulimulus unicolor petenensis ; Breure 1978: 149.
Bulimulus unicolor (Sowerby I, 1833); Thompson 2011: 107.

####### Type locality.

[Guatemala] ”campos Petenensis”.

####### Label.

”Savanes du Peten”, in Morelet’s handwriting.

####### Dimensions.

”Longit. 19.—Diam. 8”; figured specimen herein H 18.7, D 9.23, W 5.5.

####### Type material.

NHMUK 1893.2.4.1176, lectotype; 1893.2.4.1177–1178, paralectotypes (ex Morelet).

####### Remarks.

Morelet did not state on how many specimens his description was based. The current systematic position follows Thompson (2011).

####### Current systematic position.

Bulimulidae, *Bulimulus unicolor* (Sowerby I, 1833).

###### Bulimus petiti

Pfeiffer, 1846

http://species-id.net/wiki/Bulimus_petiti

Figs 70I–J , L46ii

Bulimus petiti Pfeiffer 1846a: 31; Reeve 1848 [1848–1850]: pl. 37 fig. 222; Breure 1979: 86.
Scutalus (Vermiculatus) petiti ; Breure 1978: 181 (lectotype designation).

####### Type locality.

”Peru”.

####### Label.

”Bolivia”, ”Chachopo”, taxon label in Pfeiffer’s handwriting. M.C. label style IV.

####### Dimensions.

”Long. 26, diam. 16 mill.”; figured specimen herein H 36.0, D 18.5, W 5.8.

####### Type material.

NHMUK 1975374, lectotype; 1975375, one paralectotype (Cuming coll.).

####### Remarks.

Pfeiffer described the taxon from ”Peru” on account of specimens from the Cuming collection. Reeve based himself on the same collection and gave ”Chacopo [sic], Bolivia”; one of labels mentions ”Chachopo”, which is in Venezuela where this genus does not occur. The measurements given by Pfeiffer appear to have been erroneous, as already mentioned by Pilsbry (1897 [1897–1898]: 21) and Breure (1978). With the taxon label in Pfeiffer’s handwriting, the type status of this material is not disputed. Weyrauch (1967a: 387) considered this taxon as a distinct species from Peru. However, the region where it may be found has still to be confirmed.

####### Current systematic position.

Bulimulidae, *Kuschelenia (Vermiculatus) petiti* (Pfeiffer, 1846) (**comb. n.**).

###### Bulimus philippii

Pfeiffer, 1842

http://species-id.net/wiki/Bulimus_philippii

Figs 6F , L46iii

Bulimus philippii Pfeiffer 1842: 120; 1848b: 208.

####### Type locality.

”Peru; prope Lima”.

####### Label.

”Peru”, taxon label in Pfeiffer’s handwriting. M.C. label style IV.

####### Dimensions.

”Long. 20, diam. 10 mill.”; figured specimen herein H 24.3, D 14.1, W 6.8.

####### Type material.

NHMUK 1975348, four syntypes (Cuming coll.).

####### Remarks.

Pfeiffer first mentioned his taxon as a synonym of ”[*Bulimus*] *striatulus* Sow.”, and stated in Pfeiffer (1848b) it was meant as a new name for that taxon, which was preoccupied by *Bulimus striatulum* Lamarck, 1822. The taxon is available under Art. 11.6.1 ICZN. The locality and dimensions quoted above are derived from Pfeiffer (1848b). The largest shell in the lot is figured herein. The current systematic position follows Richardson (1995).

####### Current systematic position.

Bulimulidae, *Bostryx modestus* (Broderip in Broderip and Sowerby I 1832).

###### Bulimulus (Naesiotus) phlegonis

Dall & Ochsner, 1928

http://species-id.net/wiki/Bulimulus_phlegonis

Figs 14F , L46iv

Bulimulus (Naesiotus) ustulatus var. *phlegonis*Dall and Ochsner 1928: 160, pl. 9 figs 11–12, 15–17.

####### Type locality.

[Ecuador, Galápagos, Isla San Cristóbal] ”lemon grove, on bushes at 1650 ft.”.

####### Label.

”Galapagos Isl. / Charles Isl”.

####### Dimensions.

Not given; figured specimen herein H 12.5, D 7.90, W 6.0.

####### Type material.

NHMUK 1937.6.1.8.2–5, four possible syntypes, California Academy of Sciences Expedition 1905–1906 leg. (ex Schlesch).

####### Remarks.

These specimens have been purchased from Schlesch as syntypes. However, their status has to be treated with much caution (see *lycodus*, p. 116). The current systematic position is according to Richardson (1995).

####### Current systematic position.

Bulimulidae, *Naesiotus ustulatus* (Sowerby I, 1833).

###### Bulimus phryne

Pfeiffer, 1863

http://species-id.net/wiki/Bulimus_phryne

Figs 39G–I , L46v

Bulimus phryne Pfeiffer 1863: 274; Pfeiffer 1859b: 444; Breure 1979: 112 (lectotype designation).

####### Type locality.

”Andes of Peru”.

####### Label.

”Andes of Peru”, taxon label in Pfeiffer’s handwriting. M.C. label style III.

####### Dimensions.

”Long. 31, diam. 12 mill.”; figured specimen herein H 30.8, D 13.05, W 6.7.

####### Type material.

NHMUK 1975214, lectotype; 1975215, two paralectotypes (Cuming coll.).

####### Remarks.

The current systematic position is according to Richardson (1995).

####### Current systematic position.

Bulimulidae, *Drymaeus (Drymaeus) murrinus* (Reeve, 1848).

###### Bulimus pictus

Pfeiffer, 1855

http://species-id.net/wiki/Bulimus_pictus

Figs 11H , L46vi

Bulimus pictus Pfeiffer 1855a: 58; Pfeiffer 1859b: 483; Breure 1979: 57.
Bostryx pictus ; Breure 1978: 109, pl. 10 fig. 15 (lectotype designation).

####### Type locality.

”Peru”.

####### Label.

”Peru”, taxon label in Pfeiffer’s handwriting. M.C. label style IV.

####### Dimensions.

”Long. 23, diam. 11 mill.”; figured specimen herein H 22.5, D 12.1, W 7.2.

####### Type material.

NHMUK 1975545, lectotype; 1975546, two paralectotypes (Cuming coll.).

####### Remarks.

The current systematic position follows Richardson (1995).

####### Current systematic position.

Bulimulidae, *Bostryx pictus* (Pfeiffer, 1855).

###### Buliminus pilosus

Guppy, 1871

http://species-id.net/wiki/Buliminus_pilosus

Figs 55A , L46vii

Buliminus pilosus Guppy 1871: 310, pl. 17 fig. 12; Breure 1979: 71.
Naesiotus pilosus ; Breure 1975a: 80, pl. 8 figs 1–3.

####### Type locality.

”[Trinidad]”.

####### Label.

”Trinidad”.

####### Dimensions.

”Length 14 mill., breath 7 mill.”; figured specimen herein H 14.95, D 8.05, W 6.5.

####### Type material.

NHMUK 1875.2.8.3, four syntypes (ex R. Lechmere Guppy).

####### Remarks.

Breure and Romero (2012) have recently shown that the West Indian members of *Naesiotus* fall in a distinct clade. Therefore, this species is now transferred to the genus *Protoglyptus* Pilsbry, 1897.

####### Current systematic position.

Bulimulidae, *Protoglyptus pilosus* (Guppy, 1871) (**comb.n.**).

###### Bulimus platystomus

Pfeiffer, 1858

http://species-id.net/wiki/Bulimus_platystomus

Figs 57J–L , L46viii

Bulimus platystomus Pfeiffer 1858a: 256, pl. 42 fig. 2; Breure 1979: 101.
Neopetraeus platystomus ; Breure 1978: 217, pl. 8 figs 5–6 (lectotype designation).

####### Type locality.

”Province of Patas, Andes of Peru (*Dr. Farris*)”.

####### Label.

”Province of Patas, / Andes of Peru / D^r^. Farris”, taxon label in Pfeiffer’s handwriting. M.C. label style IV.

####### Dimensions.

”Long. 40, diam. 18 mill.”; figured specimen herein H 34.4, D 22.0, W 5+.

####### Type material.

NHMUK 1975428, lectotype; 1975429, two paralectotypes (Cuming coll.).

####### Remarks.

The measurements given originally by Pfeiffer seem to be erroneous. The type status of this lot is not disputed herein, and the specimen selected as lectotype corresponds to Pfeiffer’s figure. The top of the lectotype is damaged.

####### Current systematic position.

Bulimulidae, *Neopetraeus platystomus* (Pfeiffer, 1858).

###### Bulimulus (Drymaeus) plicatoliratus

da Costa, 1898

http://species-id.net/wiki/Bulimulus_plicatoliratus

Figs 39D–F , L47i

Bulimulus (Drymaeus) plicatoliratus da Costa 1898: 80, pl. 6 fig. 1; Breure 1979: 113.

####### Type locality.

[Colombia] ”Bogotá”.

####### Label.

”Bogota”.

####### Dimensions.

”Long. 37, diam. 15.5 mm.”; figured specimen herein H 36.6, D 16.8, W 6.6.

####### Type material.

NHMUK 1907.11.21.120, lectotype; 1907.11.21.121, one paralectotype (da Costa coll.).

####### Remarks.

da Costa did not mention on how many specimens his description was based. The reference of Breure (1979) to ”HT 1907.11.21.120” has to be interpreted as a lectotype designation under Art. 74.6 ICZN. This taxon is now considered to be a junior subjective synonym of *Bulimus convexus* Pfeiffer, 1855 (**syn. n.**).

####### Current systematic position.

Bulimulidae, *Drymaeus (Drymaeus) convexus* (Pfeiffer, 1855).

###### Bulimus pliculatus

Pfeiffer, 1857

http://species-id.net/wiki/Bulimus_pliculatus

Figs 61E–F , L47ii

Bulimus pliculatus Pfeiffer 1857d: 390; Pfeiffer 1859b: 488.
Bulimus plicatulus Pfeiffer and Clessin 1881: 244 (emendation); Breure 1979: 64.
Bulimulus plicatulus ; Breure 1978: 147 (lectotype designation).

####### Type locality.

”Bolivia”.

####### Label.

”Bolivia”, taxon label in Pfeiffer’s handwriting. M.C. label style IV.

####### Dimensions.

”Long. 23, diam. 11 1/3 mill.”; figured specimen herein H 23.0, D 12.02, W 7.0.

####### Type material.

NHMUK 1975390, lectotype; 1975391, two paralectotypes (Cuming coll.).

####### Remarks.

This taxon, which has mostly been cited under the unjustified emendated name, was hitherto unfigured.

####### Current systematic position.

Bulimulidae, *Bulimulus pliculatus* (Pfeiffer, 1857).

###### Helix poecila

d’Orbigny, 1835

http://species-id.net/wiki/Helix_poecila

Figs 45M–N , L47iii

Helix poecila d’Orbigny, 1835: 11.
Bulimus poecilus ; d’Orbigny 1837 [1834–1847]: 268, pl. 31 figs 1–10 [?before 27 Feb. 1837; text 23 April 1838]; Gray 1854: 15.
Drymaeus cf. *draparnaudi* (Pfeiffer, 1847); Breure 1975b: 1150, pl. 8 fig. 2 [partim].
Drymaeus poecilus ; Breure 1975b: 1152 [partim]; Cuezzo et al. 2013: 156.

####### Type locality.

”provincia Chiquitensi (republica Boliviana)”; see remarks.

####### Label.

”valle grande, Bolivia” [1854.12.4.152], ”Monte grande. S^ta^ Cruz (Bolivia)” [.153], ”chiquitos, Bolivia” [.154], ”Chiquitos, Bolivia” [.155], ”Chiquitos, Bolivia” [.156], [No locality given,. 157], all in d’Orbigny’s handwriting.

####### Dimensions.

”Longit 22 1/2 mil.; latit. a 15 ad 16 millim.”; figured specimen herein H 34.8, D 17.9, W 5+.

####### Type material.

NHMUK 1854.12.4.152–157, 53 paralectotypes (d’Orbigny coll.).

####### Remarks.

In d’Orbigny (1838 [1834–1847]) the type locality is specified for two varieties, var. *major* and var. *minor*. Var. *major* was found especially at ”la porte de Tasajos et du bourg de Pampa grande”. Var. *minor* occurs in the forests bordering the ”Rio grande”, the forests bordering ”Rio de Tacabaca, entre San-Juan et Santo-Corazon de Chiquitos et aux environs de cette première Mission [San Juan]”. See also Breure (1973). The six lots in NHMUK represent two different varieties recognized by d’Orbigny, which are as such not mentioned in his papers, labelled as ”B” and ”C”. Lot 1854.12.4.152 consists of five specimens (”var. B”), the largest with H = 33.7. Lot. 153 has seven specimens of the same variety, the largest with H = 33.9. Lot. 154 consists of eight juveniles (”var. C”). Lot. 155 consisted originally of eight specimens, one is now missing; one corresponds to d’Orbigny 1837 [1834–1847]: pl. 31 figs 7–8 and is re-figured herein. Lot. 156 originally counted 16 specimens (one now missing), the largest measures H = 31.5. Finally, lot. 157 has 11 specimens of which the largest has H = 30.9. All material belongs to the same species, contrasting the findings of Breure (1975b) for the material in MNHN.

####### Current systematic position.

Bulimulidae, *Drymaeus (Drymaeus) poecilus* (d’Orbigny, 1835).

###### Helix polymorpha

d’Orbigny, 1835

http://species-id.net/wiki/Helix_polymorpha

Figs 67H–I , L48i

Helix polymorpha d’Orbigny 1835: 20.
Bulimus polymorphus ; d’Orbigny 1837 [1834–1847]: 289, pl. 41 figs 1–5 [19 June 1837; text 6 May 1838]; Gray 1854: 18.

####### Type locality.

”republica Peruviana”.

####### Label.

”le Pérou”, in d’Orbigny’s handwriting.

####### Dimensions.

”Longit. 33 millim., latit. a 11 ad 12 millim.” (see remarks); figured specimen herein H 22.8, D 11.6, W 5.6.

####### Type material.

NHMUK 1854.12.4.199, lectotype and one paralectotype, Fontaine leg. (d’Orbigny coll.).

####### Remarks.

d’Orbigny (1835) recognized two varieties, ”var. A. *brevis*” and ”var. B. *elongata*”, without further description. The publication date of his paper is not known and therefore has to be dated on 31 December 1835 (Art. 21.3.2 ICZN). In d’Orbigny (1838 [1834–1847]) these varieties are mentioned simply as ”A” and ”B”; he corrected the measurements to ”Long. 23 millim.; lat. 10 ad 13 millim.”. The specimen corresponding to d’Orbigny 1837 [1834–1847]: pl. 41 figs 3–4 (= var. B) is here selected as lectotype (**design. n.**). The other specimen (= var. A) corresponds to his pl. 41 fig. 1. Both specimens are conspecific. d’Orbigny’s taxon was considered a junior subjective synonym of *Bulinus bicolor* Sowerby I by previous authors (Weyrauch 1967a: 385, Richardson 1995: 348); Weyrauch considered Sowerby’s taxon to be published in 1834, Richardson gave 1835. This taxon was published in Proceedings of the Zoological Society of London 4 (1834), p. 141. According to Duncan (1937:78) the publication date is 3 April 1835 and d’Orbigny’s name is thus the junior synonym of *Bulinus bicolor* Sowerby I, 1835.

####### Current systematic position.

Bulimulidae, *Kuschelenia (Kuschelenia) bicolor* (Sowerby I, 1835).

###### Drymaeus ponsonbyi

da Costa, 1907

http://species-id.net/wiki/Drymaeus_ponsonbyi

Figs 54H , L48ii

Drymaeus ponsonbyi da Costa 1907: 305, pl. 26 figs 6–6a; Breure 1979: 113.

####### Type locality.

”Surco, Peru, at an elevation of 2,050 metres”.

####### Label.

”Surco 2050 M. / Peru”, in da Costa’s handwriting.

####### Dimensions.

”Long. 33, diam. 12 mm.”; figured specimen herein H 33.5, D 12.82, W 8.0.

####### Type material.

NHMUK 1907.11.21.27, holotype (ex da Costa).

####### Remarks.

da Costa writes ”a shell”, implying that he had only one specimen; thus the specimen is the holotype. Searching for ”Surco” in modern gazetteers for Peru does not give any locality at the elevation indicated; the type locality thus remains to be ascertained.

####### Current systematic position.

Bulimulidae, *Drymaeus ponsonbyi* da Costa, 1907.

###### Bulimus praetextus

Reeve, 1849

http://species-id.net/wiki/Bulimus_praetextus

Figs 43G–I , L48iii

Bulimus praetextus Reeve 1849 [1848–1850]: pl. 71 fig. 515; Reeve 1850: 98; Pilsbry 1898 [1897–1898]: 238, pl. 44 fig. 94.

####### Type locality.

”Andes of Caxamarca, Peru”.

####### Label.

”Peru”. M.C. label style I.

####### Dimensions.

Not given; figured specimen herein H 39.0, D 15.9, W 8.2.

####### Type material.

NHMUK 198340, lectotype (Cuming coll.).

####### Remarks.

Reeve did not mention on how many specimens his description was based. The specimen matches his figure and is here selected as lectotype (**design. n.**). The current systematic position follows Richardson (1995).

####### Current systematic position.

Bulimulidae, *Drymaeus (Drymaeus) praetextus* (Reeve, 1849).

###### Drymaeus prestoni

da Costa, 1906

http://species-id.net/wiki/Drymaeus_prestoni

Figs 18J , L48iv

Drymaeus prestoni da Costa 1906a: 9, pl. 1 fig. 9; Breure 1979: 123.

####### Type locality.

”Chiriqui, Panama”.

####### Label.

”Chiriqui. / Panama”, in da Costa’s handwriting.

####### Dimensions.

”Long. 21, diam. 10 mm.”; figured specimen herein H 21.3, D 10.58, W 5.4.

####### Type material.

NHMUK 1907.11.21.12, lectotype (da Costa coll.).

####### Remarks.

da Costa did not state on how many specimens his description was based, but mentioned ”represented by several examples hitherto unnamed in the British Museum”; in the same paper he did also describe a ”var. *cancellata*” (see p. 38). On the original label ”Type” is striked through and, in a different handwriting, added ”Holotype”. This cannot, however, be regarded as holotype designation by da Costa, so the reference of Breure (1979) to ”HT 1907.11.21.12” has to be interpreted as a lectotype designation under Art. 74.6 ICZN.

####### Current systematic position.

Bulimulidae, *Drymaeus (Mesembinus) prestoni* da Costa, 1906.

###### Bulimus primula

Reeve, 1848

http://species-id.net/wiki/Bulimus_primula

Figs 18H–I , L48v

Bulimus primula Reeve 1848 [1848–1850]: pl. 57 fig. 385; Pilsbry 1898 [1897–1898]: 247, pl. 43 fig. 70; Breure 1979: 123.
Drymaeus (Mesembrinus) primulus ; Breure and Eskens 1981: 81 (lectotype designation).

####### Type locality.

”Venezuela”.

####### Label.

”Venezuela”. M.C. label style I.

####### Dimensions.

Not given; figured specimen herein H 25.0, D 11.61, W 5.9.

####### Type material.

NHMUK 1975478, lectotype; 1975479, one paralectotype (Cuming coll.).

####### Remarks.

Reeve did not state on how many specimens his description was based. Richardson (1995: 207) treated this taxon as a junior subjective synonym of *Bulimus studeri* Pfeiffer, 1847; we concur with this opinion, but contrary to this author consider this species to belong to *Drymaeus (Mesembrinus)* Albers, 1850.

####### Current systematic position.

Bulimulidae, *Drymaeus (Mesembrinus) studeri* (Pfeiffer, 1847).

###### Bulinus proteus

Broderip, 1832

http://species-id.net/wiki/Bulinus_proteus

Figs 66A–B , L49i

Bulinus proteus Broderip in Broderip and Sowerby I 1832: 107; Sowerby I 1833 [Sowerby I and II 1832–1841]: figs 14–14c;
Bulimus sordidus Lesson; Reeve 1848 [1848–1850]: pl. 17 fig. 100c; Pilsbry 1897 [1897–1898]: 13, pl. 1 fig. 2.

####### Type locality.

”in Peruviae montibus. (St. Jacinta, near Samanca)”.

####### Label.

”Peru”. M.C. label style I.

####### Dimensions.

”long. 1 8/9, lat. 1 2/10 poll. [H 47.8, D 30.4 mm]”; figured specimen herein H 44.1, D 24.1, W 6.8.

####### Type material.

NHMUK 20100638, lectotype and three paralectotypes (Cuming coll.).

####### Remarks.

Broderip described this taxon and three varieties from the Cuming collection; they were figured by Sowerby (Sowerby I and II 1832–1841). The two lots found represent *Bulinus proteus* and one variety. Reeve 1848 [1848–1850]: pl. 17 fig. 100 named this taxon as *Bulimus sordidus* (Lesson, 1826), but stated ”The name given to this species by Mr. Broderip is a far more appropriate one (...) it is to Mr. Cuming that we are mainly indebted for the beautiful varieties obtained in different parts of Peru”. He also corrected this in the text related to Reeve 1849 [1848–1850]: pl. 59 fig. 401. A specimen corresponding to the dimensions given by Broderip could not be located in the material. One of the specimens, however, corresponds to Sowerby’s figure 14c and is here designated lectotype (**design.n.**) to fixate the nominal taxon.

####### Current systematic position.

Bulimulidae, *Scutalus proteus* (Broderip in Broderip and Sowerby I 1832).

###### Bulimus protractus

Pfeiffer, 1855

http://species-id.net/wiki/Bulimus_protractus

Figs 49G–I , L48vii

Bulimus protractus Pfeiffer 1855d: 94, pl. 31 fig. 1; Pfeiffer 1856 [1854–1860]: 66, pl. 18 figs 13–14; Breure 1979: 113 (lectotype designation).
Drymaeus protractus ; Pilsbry 1898 [1897–1898]: 224, pl. 42 fig. 62; Breure and Eskens 1981: 34.

####### Type locality.

”Meobamba, Eastern Peru (*Mr. Yates*)”.

####### Label.

”Meobamba Eastern / Peru M. Yates”, taxon label in Pfeiffer’s handwriting. M.C. label style IV.

####### Dimensions.

”Long. 30, diam. 11 1/1 mill.”; figured specimen herein H 29.3, D 13.06, W 7.3.

####### Type material.

NHMUK 1975494, lectotype; 1975495, two paralectotypes (Cuming coll.).

####### Remarks.

Given our current understanding, we disagree with Richardson (1995: 123), who considered this taxon to be a junior subjective synonym of *Bulimus expansus* Pfeiffer, 1848. However, the species complex of *Drymaeus (Drymaeus) expansus* certainly needs further research to establish the relationships. The current systematic position follows Weyrauch 1967b: 485.

####### Current systematic position.

Bulimulidae, *Drymaeus (Drymaeus) protractus* (Pfeiffer, 1855).

###### Bulinus pruinosus

Sowerby I, 1833

http://species-id.net/wiki/Bulinus_pruinosus

Figs 7F , L48vi

Bulinus pruinosus Sowerby I 1833a: 36.
Bulimus pruinosus ; Reeve 1848 [1848–1850]: pl. 20 fig. 120.

####### Type locality.

”in Peruviâ” (see remarks).

####### Label.

”Peru”. M.C. label style I.

####### Dimensions.

”long. 0.55, lat. 0.3 poll. [H 13.9, D 7.6 mm]”; figured specimen herein H 13.48, D 7.32, W 5.8.

####### Type material.

NHMUK 1975544, four syntypes (Cuming coll.).

####### Remarks.

Sowerby described this species from the Cuming collection, but did not state on how many specimens his description was based. In the remarks he stated ”found on dead leaves in the clefts of rocks in the mountains of Cobija”. This area now belongs to Chile.

####### Current systematic position.

Bulimulidae, *Bostryx pruinosus* (Sowerby I, 1833).

###### Drymaeus pseudofusoides

da Costa, 1906

http://species-id.net/wiki/Drymaeus_pseudofusoides

Figs 44J–L , L49ii

Drymaeus pseudofusoides da Costa 1906a: 8, pl. 1 fig. 6; Breure 1979: 113; Linares and Vera 2012: 189.

####### Type locality.

”Bogota, Colombia”.

####### Label.

”Bogota”, in da Costa’s handwriting.

####### Dimensions.

”Long. 33.5, diam. 12 mm.”; figured specimen herein H 33.6, D 13.35, W 6.2.

####### Type material.

NHMUK 1907.11.21.11, holotype (da Costa coll.).

####### Remarks.

da Costa wrote ”only one example of this form (...) was obtained”. The shell in the NHMUK is thus the holotype.

####### Current systematic position.

Bulimulidae, *Drymaeus (Drymaeus) pseudofusoides* da Costa, 1906.

###### Bulimus ptychostylus

Pfeiffer, 1858

http://species-id.net/wiki/Bulimus_ptychostylus

Figs 57D–F , L49iv

Bulimus ptychostylus Pfeiffer 1858a: 256, pl. 42 fig. 7; Pilsbry 1898 [1897–1898]: 178, pl. 29 fig. 27; Breure 1979: 101.
Neopetraeus ptychostylus ; Breure 1978: 217, pl. 9 fig. 16.

####### Type locality.

”Province of Patas, Andes of Peru (*Dr. Farris*)”.

####### Label.

”Province of Patas, / Andes of Peru /D^r^. Farris”, taxon label in Pfeiffer’s handwriting. M.C. label style IV.

####### Dimensions.

”Long. 47, diam. 15 mill.”; figured specimen herein H 46.7, D 19.7, W 5+.

####### Type material.

NHMUK 1975430, lectotype (Cuming coll.).

####### Remarks.

The top of the lectotype is damaged. The current systematic position is in accordance with Richardson (1995).

####### Current systematic position.

Bulimulidae, *Neopetraeus lobbii* (Reeve, 1849).

###### Bulimus puellaris

Reeve, 1850

http://species-id.net/wiki/Bulimus_puellaris

Figs 20H , L49iii

Bulimus puellaris Reeve 1850 [1848–1850]: pl. 86 fig. 637; Breure 1979: 123 (lectotype designation).
Bulimulus puellaris ; Pilsbry 1897 [1897–1898]: 66, pl. 11 fig. 8.
Bulimulus tenuissimus (d’Orbigny, 1835); Simone 2006: 120, fig. 370B.

####### Type locality.

”Brazil”.

####### Label.

”Brazil”. M.C. label style I.

####### Dimensions.

Not given; figured specimen herein H 22.4, D 10.68, W 6.2.

####### Type material.

NHMUK 1975400, lectotype (Cuming coll.).

####### Remarks.

Richardson (1995: 85) and Simone (2006) mistakenly considered this taxon as synonym of *Helix tenuissima* Férussac, 1832; the name should not be attributed to d’Orbigny, 1835, as the figure of Férussac (in Férussac and Deshayes 1819–1850: pl. 142B fig. 8) may be considered as indication and is thus available. The protoconch sculpture of *Bulimus puellaris* Reeve, 1850 shows this taxon to belong in the genus *Drymaeus* Albers, 1850. This taxon is tentatively kept as a separate species until further research has established its position.

####### Current systematic position.

Bulimulidae, *Drymaeus (Mesembrinus) puellaris* (Reeve, 1850).

###### Otostomus pulcherrimus

H. Adams, 1867

http://species-id.net/wiki/Otostomus_pulcherrimus

Figs 46D–F , L49v

Otostomus pulcherrimus H. Adams 1867a: 442, pl. 38 fig. 3; Breure 1979: 113.
Drymaeus pulcherrimus ; Pilsbry 1898 [1897–1898]: 266, pl. 34 fig. 9.

####### Type locality.

”[Eastern Peru]”.

####### Label.

”E. Peru”, taxon label in H. Adam’s handwriting.

####### Dimensions.

”Long. circa 45, diam. 17 1/2 mill.”; figured specimen herein H 39.1, D 18.2, W [1.3].

####### Type material.

NHMUK 1867.5.18.3, holotype, Bartlett leg. (ex H. Adams).

####### Remarks.

As is shown in Adam’s figure, his shell (”one example of this beautiful species”) was badly broken, with only the lower part of the shell remaining. The shell height he gave was clearly a guess, as the actual shell is smaller. The lot is accompanied by a photo ”of [a] perfect specimen from H.B. Preston”; assuming that this photograph is natural size, the dimensions are H 58.1, D 21.0. The current systematic position follows Richardson (1995: 165).

####### Current systematic position.

Bulimulidae, *Drymaeus (Drymaeus) pulcherrimus* (H. Adams, 1867).

###### Drymaeus punctatus

da Costa, 1907

http://species-id.net/wiki/Drymaeus_punctatus

Figs 46A–C , L50i

Drymaeus punctatus da Costa 1907: 304, pl. 26 figs 1–1a; Breure 1979: 113; Köhler 2007: 148, fig. 106.
Drymaeus (Drymaeus) punctatus ; Breure and Eskens 1981: 34, pl. 8 fig. 4.

####### Type locality.

”Chanchamayo, Peru”.

####### Label.

”Chanchamayo. / Peru”, in da Costa’s handwriting.

####### Dimensions.

”Long. 34, diam. 11 mm.”; figured specimen herein H 36.6, D 12.7, W 6.7.

####### Type material.

NHMUK 1907.11.21.20, lectotype; 1907.11.21.21, one paralectotype (da Costa coll.).

####### Remarks.

**Figure 1.**
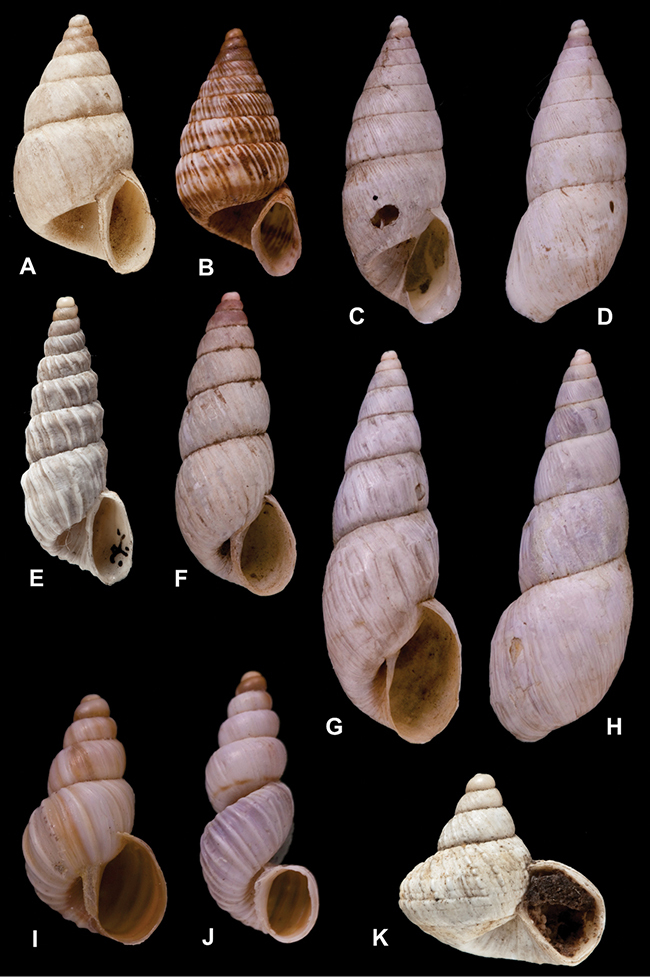
*Bostryx* species. **A** *Bostryx umbilicaris* (Souleyet, 1842), paralectotype NHMUK 1854.7.24.359 (H = 13.3) **B** *Bostryx moniezi* (Dautzenberg, 1896), syntype NHMUK 1908.6.13.22 (H = 12.46) **C–D** *Bostryx infundibulum* (Pfeiffer, 1853), lectotype NHMUK 1975163 (H = 18.25) **E** *Bostryx scalaricosta* (Morelet, 1860), lectotype NHMUK 1893.2.4.1170 (H = 15.5) **F** *Bostryx hamiltoni* (Reeve, 1849), lectotype NHMUK 1849.5.14.53 (H = 17.0) **G–H** *Bostryx lesueurianus* (Morelet, 1860), syntype NHMUK 1893.2.4.1182 (H = 22.5) **I** *Bostryx pygmaeus costatus* Weyrauch, 1960, paratype NHMUK 1975357 (H = 8.45) **J** *Bostryx imeldae costifer* Weyrauch, 1960, paratype NHMUK 1975354 (H = 9.6) **K** *Bostryx metagyra* Pilsbry & Olsson, 1949, paratype NHMUK 20100630 (H = 7.1). All enlarged.

**Figure 2.**
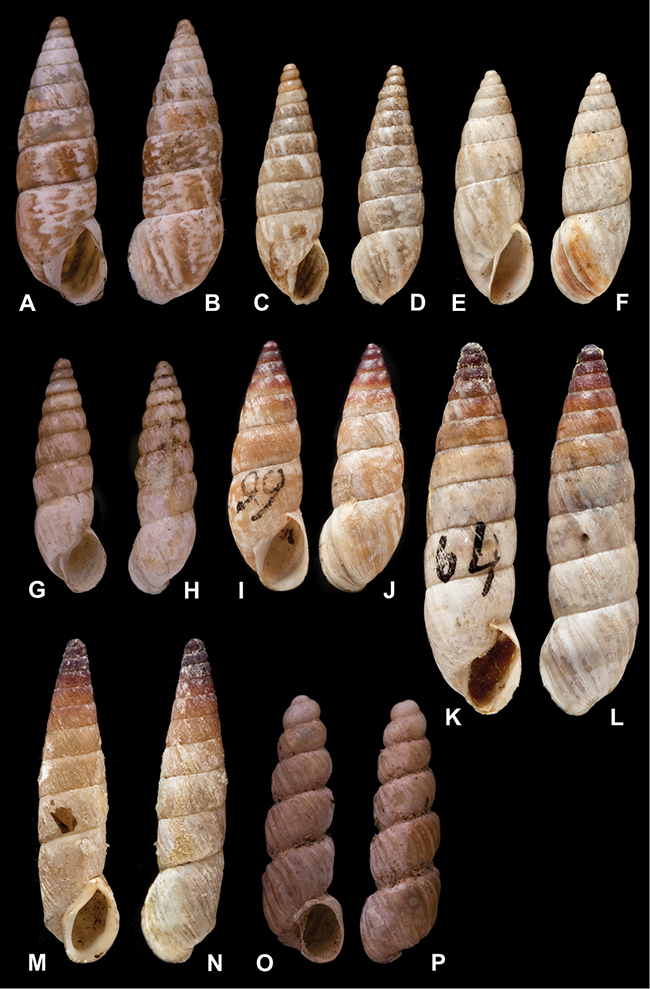
*Bostryx* species. **A–B** *Bostryx atacamensis* (Pfeiffer, 1856), lectotype NHMUK 1975312 (H = 19.0) **C–D** *Bostryx lactifluus* (Pfeiffer, 1857), syntype NHMUK 20100642 (H = 16.2) **E–F** *Bostryx lichnorum* (d’Orbigny, 1835), syntype NHMUK 1854.12.4.96 (H = 15.8) **G–H** *Bostryx nanus* (Reeve, 1849), syntype NHMUK 1975463 (H = 15.4) **I–J** *Bostryx scabiosus* (Sowerby I, 1833), syntype NHMUK 20110176 (H = 15.9) **K–L** *Bostryx pupiformis* (Broderip, 1832), syntype NHMUK 20100613 (H = 23.5) **M–N** *Bostryx terebralis* (Pfeiffer, 1842), syntype NHMUK 20110082 (H = 19.8) **O–P** *Bostryx holostoma* (Pfeiffer, 1846), lectotype NHMUK 1975345 (H = 7.84). All enlarged.

**Figure 3.**
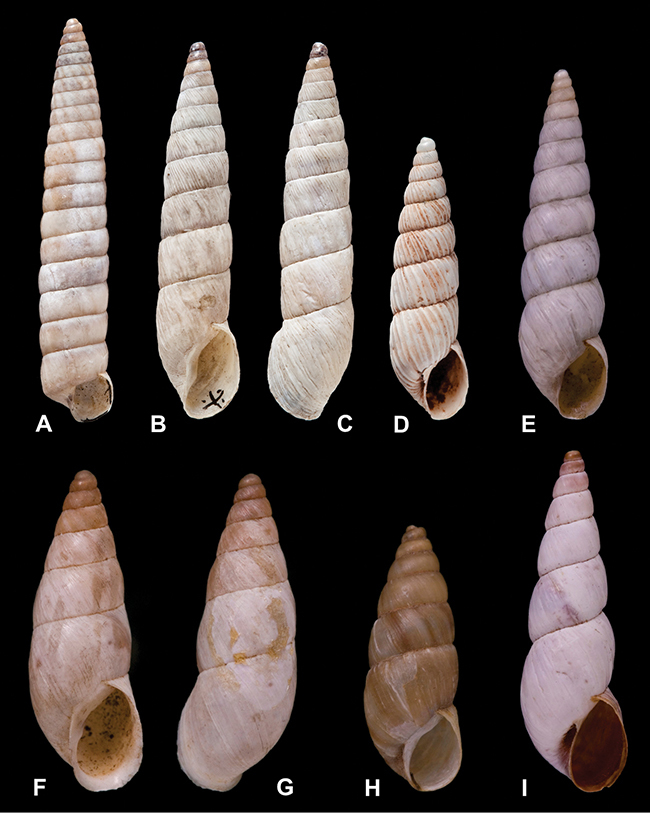
*Bostryx* species. **A** *Bostryx veruculum* (Morelet, 1860), syntype NHMUK 1893.2.4.1141 (H = 24.5) **B–C** *Bostryx spiculatus spiculatus* (Morelet, 1860), lectotype NHMUK 1893.2.4.1156 (H = 23.3) **D** *Bostryx spiculatus paucicostatus* Breure, 1978, paratype NHMUK 1975276 (H = 16.5) **E** *Bostryx emaciatus* (Morelet, 1863), syntype NHMUK 1893.2.4.248 (H = 21.7) **F–G** *Bostryx ceratacme* (Pfeiffer, 1855), lectotype NHMUK 1975347 (H = 18.0) **H** *Bostryx haasi minor* Weyrauch, 1960, paratype NHMUK 1975353 (H = 14.0) **I** *Bostryx zilchi glomeratus* Weyrauch, 1960, paratype NHMUK 1975154 (H = 18.7). All enlarged.

da Costa did not state on how many specimens his description was based, but mentioned ”there appears to be much variation both in form and colour among the specimens collected of this species”; on the same page he did also describe a ”var. *albida*” (his figs 2–2a) and a ”var. *ventricosa*” (his figs 3–3a). The reference of Breure (1979) to ”HT 1907.11.21.20” has to be interpreted as a lectotype designation under Art. 74.6 ICZN. This specimen corresponds to da Costa’s fig. 1a; the paralectotype matches closely the measurements given by da Costa (H 34.3, D 14.18, W 7.0). Köhler (2007) reported a specimen originating from Preston in the ZMB, which he considered as syntype.

####### Current systematic position.

Bulimulidae, *Drymaeus (Drymaeus) punctatus* da Costa, 1907.

###### Bulinus pupiformis

Broderip, 1832

http://species-id.net/wiki/Bulinus_pupiformis

Figs 2K–L , L50ii

Bulinus pupiformis Broderip in Broderip and Sowerby I 1832b: 105; Sowerby I 1833 [Sowerby I and II 1832–1841]: fig. 27.
Bulimus pupiformis ; Reeve 1848 [1848–1850]: pl. 14 fig. 85.

####### Type locality.

”Chili (Huasco)”.

####### Label.

”Chili”. M.C. label style I.

####### Dimensions.

”long. 7/8, lat. 2/8 poll. [H 22.2, D 6.33 mm]”; figured specimen herein H 23.5, D 6.29, W 10+.

####### Type material.

NHMUK 20100613, four probable syntypes (Cuming coll.).

####### Remarks.

One of the specimens, which are considered probable syntypes, corresponds to Reeve’s figure. The current systematic position follows Richardson (1995).

####### Current systematic position.

Bulimulidae, *Bostryx pupiformis* (Broderip, 1832).

###### Bulimus purpuratus

Reeve, 1849

http://species-id.net/wiki/Bulimus_purpuratus

Figs 71G–H , L50iii

Bulimus purpuratus Reeve 1849 [1848–1850]: pl. 71 fig. 517; Reeve 1850: 98; Breure 1979: 86.
Bulimulus purpuratus ; Pilsbry 1897 [1897–1898]: 21, pl. 4 fig. 57.
Scutalus (Vermiculatus) purpuratus ; Breure 1978: 184 (lectotype designation).

####### Type locality.

”Andes of Caxamarca, Peru; Mr. W. Lobb”.

####### Label.

No locality (see remarks). M.C. label style IV, V.

####### Dimensions.

Not given; figured specimen herein H 37.3, D 18.3, W 6.3.

####### Type material.

NHMUK 1975364, lectotype; 1975365, two paralectotypes (Cuming coll.).

####### Remarks.

The locality on the registration card in the collection is ”Andes of Catamarca”; the original label seems to be lost. The current systematic position follows Weyrauch (1967a) and Richardson (1995).

####### Current systematic position.

Bulimulidae, *Kuschelenia (Vermiculatus) purpuratus* (Reeve, 1849) (**comb. n.**).

###### Bulinus pustulosus

Broderip, 1832

http://species-id.net/wiki/Bulinus_pustulosus

Figs 9I–K , L50iv

Bulinus pustulosus Broderip in Broderip and Sowerby I 1832b: 105; Sowerby I 1833 [Sowerby I and II 1832–1841]: fig. 23.
Bulimus pustulosus ; Reeve 1848 [1848–1850]: pl. 20 fig. 127.

####### Type locality.

”Chili (Huasco)”.

####### Label.

”Chili”. M.C. label style IV.

####### Dimensions.

”long. 7/12, lat. 3/12 (circa) poll. [H 14.8, D 6.33 mm]”; figured specimen herein H 16.4, D 8.1, W 5.5.

####### Type material.

NHMUK 1975589, five probable syntypes (Cuming coll.).

####### Remarks.

Broderip did not state on how many specimens his description was based. One of the specimens corresponds to Reeve’s figure. The current systematic position follows Richardson (1995).

####### Current systematic position.

Bulimulidae, *Bostryx pustulosus* (Broderip, 1832).

###### Bulimus (Otostomus) quadrifasciatus

Angas, 1878

http://species-id.net/wiki/Bulimus_quadrifasciatus

Figs 47D–F , L50v

Bulimus (Otostomus) quadrifasciatus Angas 1878b: 312, pl. 18 figs 2–3; Breure 1979: 113 (lectotype designation).
Drymaeus quadrifasciatus ; Pilsbry 1898 [1897–1898]: 243, pl. 41 figs 22–23.
Drymaeus (Drymaeus) quadrifasciatus ; Breure and Eskens 1981: 34; Breure and Borrero 2008: 22.

####### Type locality.

”Ecuador”.

####### Label.

”Ecuador”.

Dimensions. ”Alt. 1 inch 3 lines, diam. 5 1/2 lines [H 31.6, D 11.1 mm]”; figured specimen herein H 28.9, D 15.1, W 6.0.

####### Type material.

NHMUK 1879.1.21.3, lectotype (ex Angas).

####### Remarks.

Angas did not state on how many specimens his description was based. The label states ”P.Z.S., the type”, but this is not in Angas’ handwriting.

####### Current systematic position.

Bulimulidae, *Drymaeus (Drymaeus) quadrifasciatus* (Angas, 1878).

###### Bulimulus (Scutalus) quechuarum

Crawford, 1939

http://species-id.net/wiki/Bulimulus_quechuarum

Figs 71C–D , 71K , L51i

Bulimulus (Scutalus) quechuarum Crawford 1939: 330, pl. 19 figs 11–12; Breure 1979: 86.

####### Type locality.

[Peru, Dept. Puno] ”Capachica”.

####### Label.

”Capachica, near Puno, / Peru / 12,600 ft.” [1939.4.17.226–227], ”Saracocha 13,600 ft.” [.230–231], ”Lake Junin, alt. 13000 ft Peru” [1925.12.16.20].

####### Dimensions.

”length 21.5 mm., breath 9.0 mm.”; figured specimen herein H 21.5, D 9.0, W 5.2.

####### Type material.

NHMUK 1939.4.17.226, holotype (soft parts. 229); 1939.4.17.227–228,. 230–231, four paratypes, Percy Sladen Expedition leg., 16.iv.1937; 1925.12.16.20, five paratypes, Godman Thomas Expedition leg.

####### Remarks.

The holotype is indicated on the label, on which is remarked ”smallest spec. quite likely / a different sp.”; in fact, this specimen is a juvenile *Bostryx* species and may have been mixed up with this lot. Crawford mentioned two specimens from the type locality and two from Saracocha, which is in the same region; the fifth, juvenile specimen was not mentioned by him and is here excluded from the type material. Another lot is from the Lake Junín area; we concur with the doubt expressed by Crawford himself about this being conspecific (”Junin lies …about 500 miles NW of the other two localities”), but refrain from making definitive conclusions as this is beyond the scope of this paper.

####### Current systematic position.

Bulimulidae, *Kuschelenia (Vermiculatus) quechuarum* (Crawford, 1939) (**comb. n.**).

###### Bulimus quitensis

Pfeiffer, 1848

http://species-id.net/wiki/Bulimus_quitensis

Figs 15A , L50vi

Bulimus quitensis Pfeiffer 1848a: 230; Pfeiffer 1848b: 182; Breure 1979: 71.
Naesiotus quitensis ; Breure and Coppois 1978: 181 (lectotype designation); Breure and Borrero 2008: 15.

####### Type locality.

”Quito (De Lattre)”.

####### Label.

”Quito / Mon^sr^ Delattre”, taxon label in Pfeiffer’s handwriting. M.C. label style I/IV.

####### Dimensions.

”Long. 26, diam. 12 mill.”; figured specimen herein H 26.5, D 13.25, W 6.0.

####### Type material.

NHMUK 1975320, lectotype; 1975321, two paralectotypes; 19991539, three paralectotypes, Delattre leg. (Cuming coll.).

####### Remarks.

Pfeiffer did not state on how many specimens his description was based when he described this taxon from Cuming’s collection.

####### Current systematic position.

Bulimulidae, *Naesiotus quitensis* (Pfeiffer, 1848).

###### Bulimus radiatus

Morelet, 1863

http://species-id.net/wiki/Bulimus_radiatus

Figs 12A–C , L51ii

Bulimus radiatus Morelet 1863: 188, pl. 9 fig. 2; Breure 1979: 57 (lectotype designation).
Bulimulus angrandianus Pilsbry 1897 [1897–1898]: 19 (new name for *Bulimus radiatus* Morelet not Bruguière, 1789).

####### Type locality.

[Peru, Dept. Junín/Cuzco] ”la vallée de Jauja et des pentes du Cuzco”.

####### Label.

”Chaullas, Pérou”, in Morelet’s handwriting.

####### Dimensions.

”Longit. 24–29; diam. 10–10 1/2 mill.”; figured specimen herein H 23.7, D 11.58, W 6.9.

####### Type material.

NHMUK 1893.2.4.198, lectotype; 1893.2.4.199–200, two paralectotypes (Morelet coll.).

####### Remarks.

According to Pilsbry 1897 [1897–1898]: 19 this taxon is a junior homonym of *Bulimus radiatus* Bruguière, 1789. Richardson (1995: 35, 167) placed this taxon in the synonymy of *Bulimus nigropileatus* Reeve, 1849, which we do not endorse. It is clear from the dimensions quoted by Morelet, that he had ample material at hand. The locality on the label could not be found in modern gazetteers. The mixture of material from two widely separated regions in Peru, and the lack of a precise location for the type material, calls for additional research to establish the position of this taxon.

####### Current systematic position.

Bulimulidae, *Bostryx angrandianus* (Pilsbry 1897). Nomen inquirendum.

###### Bulimulus ragsdalei

Pilsbry, 1890

http://species-id.net/wiki/Bulimulus_ragsdalei

Figs 73A–C , L51iii

Bulimulus ragsdalei Pilsbry 1890a: 122; Pilsbry 1890b: 63; Pilsbry 1890c: 296, pl. 5 fig. 3; Breure 1979: 77.

####### Type locality.

[U.S.A.] ”Cook and Montagu Counties, Texas”.

####### Label.

”St. Joe, Montagu Co. / Texas”.

####### Dimensions.

”Alt. 22, diam. 10 mill.”; figured specimen herein H 18.3, D 10.34, W 6.1.

####### Type material.

NHMUK 1898.2.1.9–10, two paratypes.

####### Remarks.

The material is labelled as ”co-types”. The current systematic position follows Richardson (1995).

####### Current systematic position.

Bulimulidae, *Rabdotus dealbatus* (Say, 1821).

###### Bulimulus (Drymaeus) rawsonis

H. Adams, 1873

http://species-id.net/wiki/Bulimulus_rawsonis

Figs 20C , L51iv

Bulimulus (Drymaeus) rawsonis H. Adams, 1873: 208, pl. 13 fig. 12.

####### Type locality.

[West Indies] ”Tobago (*Mr. Rawson*)”.

####### Label.

”Tobago”, in H. Adam’s handwriting.

####### Dimensions.

”Long. 24, diam. 10 mill.”; figured specimen herein H 23.7, D 10.06, W 6.3.

####### Type material.

NHMUK 1878.1.28.209, lectotype (H. Adams coll.).

####### Remarks.

Adams did not state on how many specimens his description was based. The specimen was designated as lectotype by Breure (1979: 123), but erroneously mentioned under *Bulimulus aureolus rawsoni* Guppy, 1871. Guppy’s shell was from the same source and his taxon was considered the senior subjective synonym of Adam’s taxon by Pilsbry (1899: 20).

####### Current systematic position.

Bulimulidae, *Drymaeus (Mesembrinus) rawsoni* (Guppy, 1871).

###### Bulimus recedens

Pfeiffer, 1864

http://species-id.net/wiki/Bulimus_recedens

Figs 48D–F , L51v

Bulimus recedens Pfeiffer 1864: 525; Breure 1979: 113 (lectotype designation).
Drymaeus (Drymaeus) recedens ; Breure and Eskens 1981: 36, pl. 6 fig. 1.

####### Type locality.

[Peru] ”Meobamba”.

####### Label.

”Meobamba”, taxon label in Pfeiffer’s handwriting. M.C. label style IV.

####### Dimensions.

”Long. 27, diam. 12 mill.”; figured specimen herein H 27.3, D 13.3, W 5.7.

####### Type material.

NHMUK 1975477, lectotype (Cuming coll.).

####### Remarks.

The current systematic position agrees with Richardson (1995).

####### Current systematic position.

Bulimulidae, *Drymaeus (Drymaeus) recedens* (Pfeiffer, 1864).

###### Bulimus reconditus

Reeve, 1849

http://species-id.net/wiki/Bulimus_reconditus

Figs 5D–F , L51vi

Bulimus reconditus Reeve 1849 [1848–1850]: pl. 76 fig. 549; Breure 1979: 57 (lectotype designation).

####### Type locality.

”—?”.

####### Label.

”Peru”. M.C. label style IV.

####### Dimensions.

Not given; figured specimen herein H 19.8, D 9.7, W 7.3.

####### Type material.

NHMUK 1975189, lectotype; 1975190, one paralectotype (Cuming coll.).

####### Remarks.

Reeve did not state a locality; his description was based on the Cuming collection. Pfeiffer (1853d: 422), referring to the same collection, mentioned ”Long. 20, diam. 9 1/2 mill.”. Pfeiffer (1868: 129) gave the locality ”Peru”; it is not known, however, if he based himself on the same Cumingian material, as there is no taxon label in Pfeiffer’s handwriting. The current systematic position follows Richardson (1995).

####### Current systematic position.

Bulimulidae, *Bostryx nigropileatus* (Reeve, 1849).

###### Bulimus rectilinearis

Pfeiffer, 1855

http://species-id.net/wiki/Bulimus_rectilinearis

Figs 24F , L51vii

Bulimus rectilinearis Pfeiffer 1855d: 96, pl. 31 fig. 7; Pfeiffer 1859b: 405; Pfeiffer 1868 [1866–1869]: 414, pl. 94 figs 19–20; Breure 1979: 123.

####### Type locality.

”Meobamba, Eastern Peru (*Mr. Yates*)”.

####### Label.

”Tarapoto Andes of P[eru] / Mr Spence”, taxon label in Pfeiffer’s handwriting. M.C. label style I.

####### Dimensions.

”Long. 24, diam. 10 1/2 mill.”; figured specimen herein H 23.9, D 11.2, W 7.7.

####### Material.

 NHMUK 1975337, three specimens (Cuming coll.).

####### Remarks.

Pfeiffer (1855g) described this species from an imperfect shell (cf. Pfeiffer 1859b: 405 and Pfeiffer 1868 [1866–1869]: 414). Pfeiffer (1859b) also mentioned ”Habitat Moyobamba Peruviae (Yates), Tarapoto (Spence)”, indicating that he had seen additional material in the Cuming collection. The material found is from the second source and one specimen corresponds to Pfeiffer 1868 [1866–1869]: pl. 94 figs 19–20; the material of Yates could not be traced in the collection. The indication as ”ST” by Breure (1979) was evidently based on the wrong series, although the label authorizes the material as seen by Pfeiffer. The current systematic position follows Richardson (1995).

####### Current systematic position.

Bulimulidae, *Drymaeus (Mesembrinus) rectilinearis* (Pfeiffer, 1855).

###### Drymaeus regularis

Fulton, 1905

http://species-id.net/wiki/Drymaeus_regularis

Figs 48J–L , L51viii

Drymaeus regularis Fulton 1905b: 25, pl. 6 fig. 6; Breure 1979: 113 (lectotype designation).

####### Type locality.

”Chanchamayo, Peru”.

####### Label.

”Chanchamayo / Peru”, in Fulton’s handwriting.

####### Dimensions.

”Maj. diam. 16, alt. 31 [mm]”; figured specimen herein H 29.6, D 16.3, W 6.1.

####### Type material.

NHMUK 1905.11.17.2, lectotype (ex Fulton).

####### Remarks.

Fulton did not state on how many specimens his description was based. The specimen found corresponds to Fulton’s figure. Richardson (1995: 170) placed this taxon wrongly in the synonymy of *Bulimus regularis* Pfeiffer, 1852, which is a Brazilian taxon based on material collected by Macgillivray. Pfeiffer’s figure (Pfeiffer 1854 in Küster and Pfeiffer 1840–1865: pl. 39 fig. 22), copied by Simone (2006: fig. 366) and listed as *Bulimulus regularis*, is clearly different from Fulton’s taxon and may prove to be a non-orthalicoid.

####### Current systematic position.

Bulimulidae, *Drymaeus regularis* Fulton, 1905.

###### Bostryx (Elatibostryx) rehderi

Weyrauch, 1960

http://species-id.net/wiki/Bostryx_rehderi

Fig. 4B

Bostryx (Elatibostryx) rehderi Weyrauch 1960a: 35, pl. 3 figs 4–5; Breure 1979: 57; Neubert and Janssen 2004: 227, pl. 7 fig. 78; Breure 2012a: 12.

####### Type locality.

”Mittel-Peru am Westhang der westlichen Anden: 2–3 km weit von Churin, an der Autostraβe nach Oyón, Río Huaura (2400–2450 m), zwischen Huacho und Cerro de Pasco”.

####### Label.

”C-Peru, 2–3 km from Churin on the highway to Oyón, 2400 m”; printed label.

####### Dimensions.

”H. 12.6, D. 3.3”; figured specimen herein H 10.45, D 3.0, W 7.9.

####### Type material.

NHMUK 1975356, five paratypes (ex Weyrauch).

####### Current systematic position.

Bulimulidae, *Bostryx rehderi* Weyrauch, 1960.

###### Bulimus rhodolarynx

Reeve, 1849

http://species-id.net/wiki/Bulimus_rhodolarynx

Figs 8G , L52i

Bulimus rhodolarynx Reeve 1849 [1848–1850]: pl. 72 fig. 518; Reeve 1850: 98; Breure 1979: 58.
Bostryx rhodolarynx rhodolarynx ; Breure 1978: 116 (lectotype designation).

####### Type locality.

[Peru] ”Banks of the Aparimao [sic, Apurimac], Alto-Peru, W. Lobb”.

####### Label.

”Andes of Peru”. M.C. label style IV.

####### Dimensions.

Not given; figured specimen herein H 34.5, D 19.6, W 7.7.

####### Type material.

NHMUK 1975434, lectotype; 1975435, two paralectotypes (Cuming coll.).

####### Current systematic position.

Bulimulidae, *Bostryx rhodolarynx* (Reeve, 1849).

###### Bulimus rimatus

Pfeiffer, 1847

http://species-id.net/wiki/Bulimus_rimatus

Figs 72A–C , L52ii

Bulimus rimatus Pfeiffer 1847: 112; Pfeiffer 1848b: 104; Reeve 1848 [1848–1850]: pl. 54 fig. 359; Breure 1979: 77 (lectotype designation).
Bulimulus rimatus ; Pilsbry 1898 [1897–1898]: 157, pl. 21 fig. 4.

####### Type locality.

”Locality unknown”.

####### Label.

No locality given. Taxon label in Pfeiffer’s handwriting. M.C. label style IV.

####### Dimensions.

”Long. 33, diam. 11 mill.”; figured specimen herein H 33.1, D 12.7, W 6.8.

####### Type material.

NHMUK 1975418, lectotype; 1975419, two paralectotypes (Cuming coll.).

####### Remarks.

Pfeiffer did not state on how many specimens his description was based. The taxon label in Pfeiffer’s hand is only partially conserved. Later authors have recognized this taxon as a Mexican species (see e.g., Martens 1893 [1890–1901]: 252); Pilsbry (1897 [1897–1898]) included this taxon under *Bulimulus* section *Sonorina*. The current systematic position follows the generic re-arragement of A.G. Smith et al. (1990: 118), who list a number of localities in Mexico, Baja California.

####### Current systematic position.

Bulimulidae, *Naesiotus rimatus* (Pfeiffer, 1847).

###### Bulimus rivasii

d’Orbigny, 1837

http://species-id.net/wiki/Bulimus_rivasii

Figs 16A–C , L52iii

Bulimus rivasii d’Orbigny 1837 [1834–1847]: 276, pl. 34 figs 8–10 [3 April 1837; text 23 April 1838]; Gray 1854: 16.
Naesiotus rivasii ; Breure 1975b: 1147; Miquel 1989a: 70, fig. 12 (lectotype designation).

####### Type locality.

[Bolivia] ”les plaines de Santa-Cruz de la Sierra, principalement à la Cuesta de Petaca, et dans tous les ravins qui bordent le Rio grande”.

####### Label.

”Monte grande Chiquitos (Bolivia)”, in d’Orbigny’s handwriting.

####### Dimensions.

”Longueur totale, 19 millimètres; largeur, 8 millimètres”; figured specimen herein H 17.7, D 8.67, W 8.2.

####### Type material.

NHMUK 1854.12.4.171, seven paralectotypes (d’Orbigny coll.).

####### Remarks.

The specimen originally figured in d’Orbigny 1837 [1834–1847]: pl. 34 fig. 9 has the top broken. According to the collation of Coan et al. (2013a) d’Orbigny’s plate was published on 3 April 1837, the text on 23 April 1838. Miquel (1989a) identified two specimens in the MNHN as syntypes of this taxon and designated one as lectotype; according to him these specimens were considered until then as part of the syntypes of *Helix crepundia* d’Orbigny, 1835. Breure (1975b), however, listed two syntypes of *Bulimus rivasii* in the MNHN collection, with locality ”Cuesta de Petaca”; see also Breure 1973: 127, fig. 5.

####### Current systematic position.

Bulimulidae, *Naesiotus rivasii* (d’Orbigny, 1837).

###### Helix rocayana

d’Orbigny, 1835

http://species-id.net/wiki/Helix_rocayana

Figs 15K–L , L52iv

Helix rocayana d’Orbigny 1835: 13.
Bulimus rocayanus ; d’Orbigny 1837 [1834–1847]: 277, pl. 33 figs 6–7 [19 June / 7 Aug 1837; text 23 April 1838]; Gray 1854: 16.
Naesiotus rocayanus ; Breure 1975b: 1147 (lectotype designation).

####### Type locality.

”provincia Santa Cruz de la Sierra (republica Boliviana)”.

####### Label.

”rio Grande. Sta Cruz (Bolivia)”, in d’Orbigny’s handwriting.

####### Dimensions.

”Latit. 14 millim. [see remarks]; longit. 8 millim.”; figured specimen herein H 22.9, D 9.07, W 8.5.

####### Type material.

NHMUK 1854.12.4.176, six paralectotypes (d’Orbigny coll.).

####### Remarks.

d’Orbigny (1838 [1834–1847]) corrected the measurements to ”Longueur totale, 24 millimètres”, and specified the locality as ”les bois épais qui bordent le Rio grande (...) surtout prés du hameau de Pacu” (see Breure 1973: 128, fig. 5).

####### Current systematic position.

Bulimulidae, *Naesiotus rocayanus* (d’Orbigny, 1835).

###### Bostryx (Bostryx) rodriguezae

Weyrauch, 1967

http://species-id.net/wiki/Bostryx_rodriguezae

Figs 4A , L52v

Bostryx (Bostryx) rodriguezae Weyrauch 1967b: 475, figs 33–48; Breure 1979: 57; Neubert and Janssen 2004: 227, pl. 3 fig. 32; Breure 2012a: 12, pl. 1 fig. 13.

####### Type locality.

”Peru, [Dept. Lima] left margin of Mayo Creek [Río Laraos], tributary to Río Cañete, 4 km from Laraos on the trail to [Cerro] Quichao, 3500 m”.

####### Label.

”C-Peru, between Laraos and Quichao, valley of Rio Cañete, 3500 m”; printed label.

####### Dimensions.

”H. 27.5, D. 10.4”; figured specimen herein H 25.5, D 8.8, W 8.3.

####### Type material.

NHMUK 1975355, five paratypes (ex Weyrauch WW3331).

####### Current systematic position.

Bulimulidae, *Bostryx rodriguezae* Weyrauch, 1967.

###### Bulimus roseatus

Reeve, 1848

http://species-id.net/wiki/Bulimus_roseatus

Figs 18M–N , L52vi

Bulimus roseatus Reeve 1848 [1848–1850]: pl. 54 fig. 353a–b; Breure 1979: 123 (lectotype designation).
Drymaeus (Mesembrinus) roseatus ; Breure and Eskens 1981: 83.

####### Type locality.

”Venezuela”.

####### Label.

”Venezuela”. M.C. label style IV.

####### Dimensions.

Not given; figured specimen herein H 35.6, D 14.45, W 7.0.

####### Type material.

NHMUK 1975309, lectotype; 1975310, two paralectotypes (Cuming coll.).

####### Remarks.

The lectotype corresponds to Reeve 1848 [1848–1850]: pl. 54 fig. 353b. Richardson (1995: 133) considered this taxon as synonym of *Bulimus granadensis* Pfeiffer, 1848; we tentatively retain it as a separate taxon.

####### Current systematic position.

Bulimulidae, *Drymaeus (Mesembrinus) roseatus* (Reeve, 1848).

###### Drymaeus rosenbergi

da Costa, 1906

http://species-id.net/wiki/Drymaeus_rosenbergi

Figs 40D–F , L52vii

Drymaeus rosenbergi da Costa 1906b: 98, pl. 11 fig. 6; Breure 1979: 113 (lectotype designation).

####### Type locality.

”Pozuzo, Eastern Peru”.

####### Label.

”Pozuzo / E Peru”, in da Costa’s handwriting.

####### Dimensions.

”Long. 20.5, diam. 9 mm.”; figured specimen herein H 19.0, D 8.66, W 5.9.

####### Type material.

NHMUK 1907.11.21.17, lectotype; 1907.11.21.18, one paralectotype (da Costa coll.).

####### Remarks.

da Costa did not state on how many specimens his description was based.

####### Current systematic position.

Bulimulidae, *Drymaeus (Drymaeus) rosenbergi* da Costa, 1906.

###### Bulimus rubrifasciatus

Reeve, 1848

http://species-id.net/wiki/Bulimus_rubrifasciatus

Figs 63C–D , L52x

Bulimus rubrifasciatus Reeve 1848 [1848–1850]: pl. 44 fig. 277; Breure 1979: 64 (lectotype designation).

####### Type locality.

”—?”.

####### Label.

No locality label.

####### Dimensions.

Not given; figured specimen herein H 22.9, D 9.55, W 6.0.

####### Type material.

NHMUK 1975393, lectotype; 1975394, two paralectotypes (Cuming coll.).

####### Remarks.

Reeve did not state on how many specimens his description was based; the shell matches his figure. The label may have either been lost or never have been present. The current systematic position follows Richardson (1995).

####### Current systematic position.

Bulimulidae, *Bulimulus gadalupensis* (Bruguière, 1789).

###### Bulimus (Otostomus) rubrovariegatus

Higgins, 1868

http://species-id.net/wiki/Bulimus_rubrovariegatus

Figs 54F , L67i

Bulimus (Otostomus) rubrovariegatus Higgins 1868: 178, pl. 12 figs 2–2a.

####### Type locality.

”Huamachuco, Peru”.

####### Label.

”Peru”.

####### Dimensions.

”Long. 37, diam. 13 mill.”. Figured specimen H 32.8, D 12.1, W 7.5.

####### Type material.

NHMUK 1868.4.3.1, three possible syntypes, ex R.F. Geale.

####### Remarks.

Higgins described this taxon from ”several specimens” which were found (in part?) by Mr. Farris. The lot found was received from the shell dealer R.F. Geale; further data are missing. As the shells were registered at the same time of Higgins’ publication, it is possible that they were part of the original series; they are treated herein as possible syntypes. The current systematic position is according to Richardson (1995).

####### Current systematic position.

Bulimulidae, *Drymaeus (Drymaeus) rubrovariegatus* Higgins, 1868.

###### Bulinus rugiferus

Sowerby I, 1833

http://species-id.net/wiki/Bulinus_rugiferus

Figs 12J , L52viii

Bulinus rugiferus Sowerby I 1833a: 36; Sowerby I 1833 [Sowerby I and II 1832–1841]: fig. 40; Reeve 1848 [1848–1850]: pl. 20 fig. 118; Breure 1979: 71.
Naesiotus rugiferus ; Breure and Coppois 1978: 185 (lectotype designation).

####### Type locality.

”ad Insulam Jacobi, inter Gallapagos Insulis”.

####### Label.

”James Is.”, in E.A. Smith’s handwriting. M.C. label style III.

Dimensions. ”long. 0.5, lat. 0.2 poll. [H 12.6, D 5.06 mm]”; figured specimen herein H 12.52, D 4.28, W 8.4.

####### Type material.

NHMUK 1975178, lectotype; 1975179, four paralectotypes (Cuming coll.).

####### Remarks.

Sowerby I did not state on how many specimens from the Cuming collection his description was based. One of the specimens found has also been figured by Reeve, and was selected as lectotype by Breure (1979).

####### Current systematic position.

Bulimulidae, *Naesiotus rugiferus* (Sowerby I, 1833).

###### Bulinus rugulosus

Sowerby I, 1833

http://species-id.net/wiki/Bulinus_rugulosus

Fig. 74B , L67iv

Bulinus rugulosus Sowerby I 1833 [Sowerby I and II 1832–1841]: fig. 37; Breure 1979: 71.
Bulimus rugulosus ; Reeve 1848 [1848–1850]: pl. 20 fig. 123.
Naesiotus rugulosus ; Breure and Coppois 1978: 186 (lectotype designation).

####### Type locality.

”Galapagos”.

####### Label.

”Chatham Island / Galapagos”. M.C. label style III.

####### Dimensions.

Not given. Figured specimen H 20.5, D 8.25, W 7.6.

####### Type material.

NHMUK 1975176, lectotype; 1975177, two paralectotypes (Cuming coll.).

####### Remarks.

Sowerby did not state on how many specimens his description was based. The current systematic position follows Richardson (1995).

####### Current systematic position.

Bulimulidae, *Naesiotus rugulosus* (Sowerby I, 1833).

###### Bulimus rusticellus

Morelet, 1860

http://species-id.net/wiki/Bulimus_rusticellus

Figs 7D , L52ix

Bulimus rusticellus Morelet 1860: 373; Morelet 1863: 185, pl. 8 fig. 5.
Bulimulus (Lissoacme) rusticellus ; Pilsbry 1896 [1895–1896]: 170, pl. 49 figs 23–24.

####### Type locality.

[Peru] ”[intimâ Peruviii regionae]”; see remarks.

####### Label.

”Pérou, Jauja”, in Morelet’s handwriting.

####### Dimensions.

”Longit. 20; diam 10 1/1 mill.”; figured specimen herein H 19.9, D 13.83, W 6.3.

####### Type material.

NHMUK 1893.2.4.201, lectotype; 1893.2.4.202–203, two paralectotypes (Morelet coll.).

####### Remarks.

Morelet (1860) did not state on how many specimens his description was based. In Morelet (1863) the locality is specified as ”la vallée de Jauja”. The specimen corresponding to his figure is now selected lectotype (**design. n.**) to fixate this taxon. The current systematic position is according to Richardson (1995).

####### Current systematic position.

Bulimulidae, *Bostryx rusticellus* (Morelet, 1860).

###### Bulimus saccatus

Pfeiffer, 1855

http://species-id.net/wiki/Bulimus_saccatus

Figs 30D–F , L53i

Bulimus saccatus Pfeiffer 1855d: 94, pl. 31 fig. 2; Breure 1979: 114 (lectotype designation).

####### Type locality.

”Meobamba, Eastern Peru (*Mr. Yates*)”.

####### Label.

”Meobamba Eastern / Peru M Yates”, taxon label in Pfeiffer’s handwriting. M.C. label style I.

####### Dimensions.

”Long. 22, diam. 10 mill.”; figured specimen herein H 21.5, D 11.5, W 6.4.

####### Type material.

NHMUK 1975207, lectotype; 1975218, two paralectotypes (Cuming coll.).

####### Remarks.

This taxon appears to be a colour form of *Bulinus strigatus* Sowerby I, 1833; the current systematic position follows Richardson (1995).

####### Current systematic position.

Bulimulidae, *Drymaeus (Drymaeus) strigatus* (Sowerby I, 1833).

###### Bulimus (Leptomerus) sanctaeluciae

E.A. Smith, 1889

http://species-id.net/wiki/Bulimus_sanctaeluciae

Figs 53D–E , L53ii

Bulimus (Leptomerus) sanctaeluciae E.A. Smith 1889: 403; Breure 1979: 71.
Naesiotus sanctaeluciae ; Breure 1975a: 82, pl. 7 fig. 7 (lectotype designation).

####### Type locality.

[West Indies] ”[St. Lucia]” (see remarks).

####### Label.

”St. Lucia / Fonds S^t^ Jacques”.

####### Dimensions.

”Longit. 21 millim., lat. 9”; figured specimen herein H 20.7, D 9.5, W 7.3.

####### Type material.

NHMUK 1889.3.23.12, lectotype, G.A. Ramage leg.

####### Remarks.

Smith (1889) reported on shells from Dominica and St. Lucia; those from the latter island were marked with an asterisk. He did not mention details on the locality for this taxon, nor on the number of specimens examined. Therefore, the reference of Breure (1975) to ”holotype: BMNH” has to be interpreted as a lectotype designation under Art. 74.6 ICZN. The periostracum behind the lip of the type specimen has begun to peel off. The current systematic position takes into account the results of Breure and Romero (2012); this taxon is now placed in the genus *Protoglyptus* Pilsbry, 1897.

####### Current systematic position.

Bulimulidae, *Protoglyptus sanctaeluciae* (E.A. Smith, 1889) (**comb. n.**).

###### Bulinus scabiosus

Sowerby I, 1833

http://species-id.net/wiki/Bulinus_scabiosus

Figs 2I–J , L53iii

Bulinus scabiosus Sowerby I 1833b: 74; Sowerby I 1833 [Sowerby I and II 1832–1841]: fig. 24; Reeve 1848 [1848–1850]: pl. 14 fig. 84.

####### Type locality.

[Chile] ”ad Cobijam”.

####### Label.

”Peru” (see remarks). M.C. label style I.

####### Dimensions.

Not given; figured specimen herein H 15.9, D 4.9, W 6.8.

####### Type material.

NHMUK 2011076, three probable syntypes (Cuming coll.).

####### Remarks.

Sowerby described his species from the Cuming museum; see Petit (2009) and Breure and Ablett (2011: 3, 10, 12) for the connection between the Sowerby’s and Cuming. The specimens found—and herein considered as probable syntypes—have also been figured by Reeve, who cited as locality ”Cobijam, Peru”; this was correct at the time of collecting, as the area of Cobija only later became part of Chile. The current systematic position follows Richardson (1995).

####### Current systematic position.

Bulimulidae, *Bostryx scabiosus* (Sowerby I, 1833).

###### Bulimus scalaricosta

Morelet, 1860

http://species-id.net/wiki/Bulimus_scalaricosta

Figs 1E , L53iv

Bulimus scalaricosta Morelet 1860: 375; Morelet 1863: 205, pl. 11 fig. 8; Breure 1979: 58.
Bostryx tubulatus scalaricostus ; Breure 1978: 132 (lectotype designation).

####### Type locality.

[Peru] ”[intimâ Peruviii regionae]”; see remarks.

####### Label.

”Huerta de Yucay”, in Morelet’s handwriting.

####### Dimensions.

”Longit 15; diam. 5 mill.”; figured specimen herein H 15.5, D 5.7, W 7.5.

####### Type material.

NHMUK 1893.2.4.1170, lectotype; 1893.2.4.1171, one paralectotype (Morelet coll.).

####### Remarks.

Morelet (1860) did not state on how many specimens his description was based. In Morelet (1863) the locality is specified as ”sur le plateau d’Andamarca”; there are different localities with that name in southern Peru. The locality mentioned on the label is near Urubamba, from where the species has been reported by Breure (1978).

####### Current systematic position.

Bulimulidae, *Bostryx tubulatus scalaricostus* (Morelet, 1860).

###### Bulinus scalariformis

Broderip, 1832

http://species-id.net/wiki/Bulinus_scalariformis

Figs 7B , L53v

Bulinus scalariformis Broderip in Broderip and Sowerby I 1832a: 31; Sowerby I 1833 [Sowerby I and II 1832–1841]: fig. 13; Reeve 1848 [1848–1850]: pl. 21 figs 129a–b.
Bulimulus (Lissoacme) scalariformis ; Pilsbry 1896 [1895–1896]: 169, pl. 47 figs 79–80.

####### Type locality.

”in Peruviâ. (Ancon)”.

####### Label.

”Peru”. M.C. label style I.

####### Dimensions.

”long. 5/10 lat. 3/10 poll. [H 12.6, D 7.6 mm]”; figured specimen herein H 11.6, D 7.7, W 5.5.

####### Type material.

NHMUK 20100635, five probable syntypes; 20100636, five probable syntypes (Cuming coll.).

####### Remarks.

There are two lots with the same same label. As Broderip described this taxon from Cuming’s collection, we consider them as probable syntypes; see also the remarks under *Bulinus scabiosus*. One of the specimens of lot 20100635 corresponds to Reeve 1848 [1848–1850]: pl. 21 fig. 129a; the other figure (129b) corresponds to one of the specimens from the second lot (20100636). The current systematic position follows Richardson (1995).

####### Current systematic position.

Bulimulidae, *Bostryx scalariformis* (Broderip in Broderip and Sowerby I 1832).

###### Bulimus schiedeanus

Pfeiffer, 1841

http://species-id.net/wiki/Bulimus_schiedeanus

Figs 72D–F , L53vi

Bulimus schiedeanus Pfeiffer 1841: 43; Reeve 1848 [1848–1850]: pl. 54 fig. 361; Breure 1979: 77.

####### Type locality.

”Mejico (Dr. Schiede)”.

####### Label.

”Mexico”, taxon label in Pfeiffer’s handwriting. M.C. label style IV.

####### Dimensions.

”Long. 16, diam. 9 lin. [H 34.8, D 19.6 mm]”; figured specimen herein H 28.4, D 16.0, W 6.2.

####### Type material.

NHMUK 1975240, three probable syntypes (Cuming coll.).

####### Remarks.

Pfeiffer probably described this taxon from a specimen in his own collection, which is likely to be lost. The measurements given by Pfeiffer are thus interpreted as German lines. The specimens from Cuming’s collection also bear Pfeiffer’s handwriting and are probably the only extant ones that confirms his identification; they are considered as probable syntypes. The current systematic position follows Thompson (2011: 127).

####### Current systematic position.

Bulimulidae, *Rabdotus (Rabdotus) schiedeanus schiedeanus* (Pfeiffer, 1841).

###### Bulimus schmidti

Pfeiffer, 1854

http://species-id.net/wiki/Bulimus_schmidti

Fig. L54i

Bulimus coarctatus Reeve 1848 [1848–1850]: pl. 41 fig. 260; not *Bulimus coarctatus* Pfeiffer, 1845.
Bulimus schmidti Pfeiffer 1854a: 65.

####### Type locality.

”...?”.

####### Label.

”—?”.

####### Dimensions.

”Long. 34, diam 17 mill.”. Figured specimen H 34.0, D 18.9, W 6.6.

####### Type material.

NHMUK 1975270, two specimens (Cuming coll.).

####### Remarks.

Pfeiffer described his taxon from ”einer alten Sammlung” from an unknown locality. His first reference of Reeve’s figure being conspecific is given in Pfeiffer 1858b: 166 (Richardson’s reference (1995: 174) to Proceedings of the Zoological Society of London 1852 [1854]: 65 is a lapsus). The specimens in this lot corresponds to Pfeiffer’s description and one of the shells fits his dimensions and also Reeve’s figure. However, since no taxon label in Pfeiffer’s handwriting nor a label from Reeve’s Conchologica Iconica is present, the specimens are not considered type specimens.

####### Current systematic position.

Bulimulidae, *Drymaeus (Drymaeus) schmidti* (Pfeiffer, 1854).

###### Bulimus scitulus

Reeve, 1849

http://species-id.net/wiki/Bulimus_scitulus

Figs 44D–F , L54ii

Bulimus scitulus Reeve 1849 [1848–1850]: pl. 71 fig. 513; Reeve 1850: 97.
Drymaeus scitulus ; Pilsbry 1898 [1897–1898]: 271, pl. 47 fig. 16.
Drymaeus (Drymaeus) scitulus ; Breure and Eskens 1981: 39 (lectotype designation).

####### Type locality.

”Chachapoyas, Alto-Peru”.

####### Label.

”Chachapoyas, Peru”. M.C. label style III.

####### Dimensions.

Not given; figured specimen herein H 29.5, D 11.7, W 7.5.

####### Type material.

NHMUK 1975217, lectotype and two paralectotypes (Cuming coll.).

####### Remarks.

The label is in E.A. Smith’s handwriting; the type has been marked with a ‘x’. The current systematic position follows Richardson (1995: 174).

####### Current systematic position.

Bulimulidae, *Drymaeus (Drymaeus) scitulus* (Reeve, 1849).

###### Otostomus scitus

H. Adams, 1867

http://species-id.net/wiki/Otostomus_scitus

Figs 41D–F , L54iii

Otostomus scitus H. Adams 1867a: 442, pl. 38 fig. 5.

####### Type locality.

[Peru] ”[Upper Amazons, and on the River Ucayali, Eastern Peru]”.

####### Label.

”Upper Amazons”.

####### Dimensions.

”Long. 28, diam. maj. 17 mill.”; figured specimen herein H 27.1, D 16.8, W 6.1.

####### Type material.

NHMUK 1867.5.18.5, holotype (ex H. Adams).

####### Remarks.

Adams stated ”one example only”, thus there is no doubt this is the holotype. The current systematic position is according to Richardson (1995: 123).

####### Current systematic position.

Bulimulidae, *Drymaeus (Drymaeus) expansus* (Pfeiffer, 1848).

###### Bulimus sculpturatus

Pfeiffer, 1846

http://species-id.net/wiki/Bulimus_sculpturatus

Figs 74C , L67iii

Bulimus sculpturatus Pfeiffer 1846a: 29; Pfeiffer 1848b: 183; Reeve 1848 [1848–1850]: pl. 20 fig. 125; Breure 1979: 71.
Bulimulus sculpturatus ; Pilsbry 1897 [1897–1898]: 120, pl. 24 fig. 41.
Naesiotus sculpturatus ; Breure and Coppois 1978: 187 (lectotype designation).

####### Type locality.

”Gallapagos Islands”.

####### Label.

”Galapagos Is.”. M.C. label style III, V.

####### Dimensions.

”Long. 14, diam. 6 1/2 mill.”. Figured specimen H 14.4, D 7.7, W 6.8.

####### Type material.

NHMUK 1975174, lectotype; 1975175, three paralectotypes (Cuming coll.).

####### Remarks.

Pfeiffer described his material from the Cuming collection, based on material ”found on bushes by Darwin”. Reeve based himself on the same material. Although the specimens are not accompanied by a taxon label in Pfeiffer’s handwriting, their type status is not disputed as the shell height closely agrees with Pfeiffer’s data. The current systematic position follows Richardson (1995).

####### Current systematic position.

Bulimulidae, *Naesiotus sculpturatus* (Pfeiffer, 1846).

###### Bulimulus (Drymaeus) selli

Preston, 1909

http://species-id.net/wiki/Bulimulus_selli

Figs 49A–C , L54iv

Bulimulus (Drymaeus) selli Preston 1909: 511, pl. 10 fig. 3; Breure 1979: 114 (lectotype designation).

####### Type locality.

”British Guiana”.

####### Label.

”British Guiana”.

####### Dimensions.

”Alt. 24, diam. maj. 13 mm.”; figured specimen herein H 24.0, D 12.8, W 6.2.

####### Type material.

NHMUK 1915.1.6.36, lectotype (ex Preston).

####### Remarks.

Preston did not state on how many specimens his description was based; the reference of Breure (1979) to ”HT BMNH 1915.1.6.36” has to be interpreted as a lectotype designation under Art. 74.6 ICZN. The columellar margin of the specimen is damaged. The current systematic position agrees with Richardson (1995: 174).

####### Current systematic position.

Bulimulidae, *Drymaeus (Drymaeus) selli* (Preston, 1909).

###### Bulimus serotinus

Morelet, 1860

http://species-id.net/wiki/Bulimus_serotinus

Figs 12E , L54v

Bulimus serotinus Morelet 1860: 374; Morelet 1863: 207, pl. 11 fig. 5; Breure 1979: 58 (lectotype designation).

####### Type locality.

[Peru] ”[intimâ Peruviii regionae]”; see remarks.

####### Label.

”Pérou. Andahuaylas”, in Morelet’s handwriting.

####### Dimensions.

”Longit. 26; diam. 10 1/2 mil.”; figured specimen herein H 25.5, D 11.75, W 7.5.

####### Type material.

NHMUK 1893.2.4.204, lectotype; 1893.2.4.205–206, two paralectotypes (Morelet coll.).

####### Remarks.

Morelet (1860) did not state on how many specimens his description was based. In Morelet (1863) the locality is specified as ”notammant à Andahuyalas, Abancay et Chupan”. The current systematic position follows Richardson (1995: 44).

####### Current systematic position.

Bulimulidae, *Bostryx serotinus* (Morelet, 1860).

###### Bulimus serratus

Pfeiffer, 1855

http://species-id.net/wiki/Bulimus_serratus

Figs 49D–F , L54vi

Bulimus serratus Pfeiffer 1855d: 94, pl. 31 fig. 6; Pfeiffer 1856 [1854–1860]: 66, pl. 18 figs 15–16; Breure 1979: 114 (lectotype designation).
Drymaeus serratus ; Pilsbry 1898 [1897–1898]: 218, pl. 36 figs 46–47.
Drymaeus (Drymaeus) serratus ; Breure and Eskens 1981: 39.

####### Type locality.

”Meobamba, Eastern Peru (*Mr. Yates*)”.

####### Label.

”Meobamba Eastern / Peru M Yates”, taxon label in Pfeiffer’s handwriting. M.C. label style IV.

####### Dimensions.

”Long. 27, diam. 11 mill.”; figured specimen herein H 26.3, D 12.6, W 5.7.

####### Type material.

NHMUK 1975475, lectotype; 1975476, two paralectotypes (Cuming coll.).

####### Remarks.

The lectotype is damaged on the penultimate whorl. The current systematic position follows Richardson (1995: 177).

####### Current systematic position.

Bulimulidae, *Drymaeus (Drymaeus) serratus* (Pfeiffer, 1855).

###### Bulimus signifer

Pfeiffer, 1855

http://species-id.net/wiki/Bulimus_signifer

Figs 25I–K , L55i

Bulimus signifer Pfeiffer 1855c: 8; Breure 1979: 123 (lectotype designation).
Drymaeus (Mesembrinus) signifer ; Breure and Eskens 1981: 88.

####### Type locality.

”Venezuela?”.

####### Label.

”Venezuela?”, taxon label in Pfeiffer’s handwriting. M.C. label style III.

####### Dimensions.

”Long. 33, diam. 13 1/1 mill.”; figured specimen herein H 32.9, D 15.1, W 6.3.

####### Type material.

NHMUK 1975216, lectotype (Cuming coll.).

####### Remarks.

The current systematic position is in agreement with Richardson (1995: 178).

####### Current systematic position.

Bulimulidae, *Drymaeus (Mesembrinus) signifer* (Pfeiffer, 1855).

###### Bulimus simpliculus

Pfeiffer, 1855

http://species-id.net/wiki/Bulimus_simpliculus

Figs 10C , L55ii

Bulimus simpliculus Pfeiffer 1855g: 124; Breure 1979: 58.
Bostryx simpliculus ; Breure 1978: 120 (lectotype designation).

####### Type locality.

”—?”.

####### Label.

”Peru + Bolivia”, taxon label in Pfeiffer’s handwriting. M.C. label style IV.

####### Dimensions.

”Long. 19 1/2, diam. 9 1/2 mill.”; figured specimen herein H 18.9, D 9.3, W 6.0.

####### Type material.

NHMUK 1975340, lectotype; 1975341, two paralectotype (Cuming coll.).

####### Remarks.

This taxon, for which no precise localities are known, is here figured for the first time. The current systematic position follows Richardson (1995: 44).

####### Current systematic position.

Bulimulidae, *Bostryx simpliculus* (Pfeiffer, 1855).

###### Bulimus sisalensis

Morelet, 1849

http://species-id.net/wiki/Bulimus_sisalensis

Figs 24G , L55iii

Bulimus sisalensis Morelet 1849: 9; Breure 1975b: 1152 (lectotype designation); Breure 1979: 123; Neubert and Janssen 2004: 230, pl. 16 fig. 193.

####### Type locality.

”cum precedente [circa Sisalensem pagum Yucatanorum]”.

####### Label.

”Sisal”, in Morelet’s handwriting.

####### Dimensions.

”Longit. 24.—Diam. 9 1/2”; figured specimen herein H 24.1, D 10.25, W 7.2.

####### Type material.

NHMUK 1893.2.4.1655–1657, three paralectotypes (Morelet coll.).

####### Remarks.

The current systematic position follows Thompson (2011: 120).

####### Current systematic position.

Bulimulidae, *Drymaeus (Mesembrinus) multilineatus* (Say, 1825).

###### Bulimulus (Drymaeus) smithii

da Costa, 1898

http://species-id.net/wiki/Bulimulus_smithii

Figs 30G–I , L55iv

Bulimulus (Drymaeus) smithii da Costa 1898: 81, pl. 6 fig. 8; Breure 1979: 114 (lectotype designation).
Drymaeus smithii ; Pilsbry 1898 [1897–1898]: 247, pl. 50 fig. 1; Linares and Vera 2012: 190.

####### Type locality.

[Colombia] ”Bogotá”.

####### Label.

”Bogota”, in da Costa’s handwriting.

####### Dimensions.

”Long. 29.5, diam. 15 mm.”; figured specimen herein H 29.5, D 15.4, W 5.8.

####### Type material.

NHMUK 1907.11.21.52, lectotype; 1907.11.21.53–54, two paralectotypes (da Costa coll.).

####### Remarks.

da Costa wrote ”this shell”, although three specimens are present in the original lot. Only if we assume that ”this shell” is implying that da Costa had only one specimen at hand, the lectotype designation by Breure (1979) would lose its status; however, the presence of multiple specimens in the original lot argues against this assumption.

####### Current systematic position.

Bulimulidae, *Drymaeus (Drymaeus) smithii* (da Costa, 1898).

###### Bulimulus (Drymaeus) solidus

Preston, 1907

http://species-id.net/wiki/Bulimulus_solidus

Figs 32D–F , L55v

Bulimulus (Drymaeus) solidus Preston 1907: 494, fig. 9; Breure 1979: 114; Köhler 2007: 148, fig. 109; Breure 2013: 40, figs 13C, 13iii; Linares and Vera 2012: 190.

####### Type locality.

”Bogota, United States of Colombia”.

####### Label.

”Bogota, U.S. Colombia”.

####### Dimensions.

”Alt. 32.5, diam. maj. 15 mm.”; figured specimen herein H 32.8, D 15.0, W 6.1.

####### Type material.

NHMUK 1908.7.2.72–73, two syntypes (ex Preston).

####### Remarks.

Preston did not state on how many specimens his description was based. Breure and Borrero (unpublished data) have found no material with a precise locality for this species. The locality quoted by Linares and Vera (2012: ”Sintipó, Cundinamarca”) is clearly in error.

####### Current systematic position.

Bulimulidae, *Drymaeus (Drymaeus) solidus* (Preston, 1907).

###### Bulimus sowerbyi

Pfeiffer, 1847

http://species-id.net/wiki/Bulimus_sowerbyi

Figs 23N , L55vi

Bulimus sowerbyi Pfeiffer 1847: 114; Pfeiffer 1848b: 195; Reeve 1848 [1848–1850]: pl. 57 fig. 383.
Neopetraeus sowerbyi ; Pilsbry 1898 [1897–1898]: 174, pl. 29 fig. 31.

####### Type locality.

”Columbian Andes (Linden)”.

####### Label.

”Andes of Colombia”. M.C. label style I.

####### Dimensions.

”Long. 22, diam. 10 mill.”; figured specimen herein H 21.7, D 11.15, W 6.2.

####### Type material.

NHMUK 20100566, one probable syntype (Cuming coll.).

####### Remarks.

Pfeiffer, who described this taxon from Cuming’s collection, did not state on how many specimens his description was based. The specimen found corresponds to Reeve’s figure, which was copied by Pilsbry (1898 [1897–1898]), who attributed this taxon to Reeve. Pilsbry classified the taxon with *Neopetraeus* Martens, 1885, but this is likely to be unjustified; Breure (1979: 136) treated it as a nomen inquirendum. Although the material is not accompanied by a taxon label in Pfeiffer’s handwriting, its type status is not disputed as the shell height agrees with Pfeiffer’s measurements. On the taxon label, the locality is given ”Andes of Colombia”; on the front side of the card board is written ”Venezuela”. The latter locality has clearly been added during later years, and may reflect the change in political-administrative borders at that time. The protoconch sculpture is worn, making a definite conclusion difficult; judged from the colour pattern in this shell, this taxon may prove to belong to *Drymaeus (Mesembrinus)*.

####### Current systematic position.

Bulimulidae, *Drymaeus (Mesembrinus)*. Nomen inquirendum.

###### Drymaeus spadiceus

da Costa, 1906

http://species-id.net/wiki/Drymaeus_spadiceus

Figs 34D–F , L56i

Drymaeus spadiceus da Costa 1906b: 97, pl. 11 figs 2–3; Breure 1979: 114; Linares and Vera 2012: 190.

####### Type locality.

”Bogota”.

####### Label.

”Bogota”, in da Costa’s handwriting.

####### Dimensions.

”Long. 39, diam. 19 mm.”; figured specimen herein H 37.3, D 19.8, W 6.3.

####### Type material.

NHMUK 1907.11.21.15, holotype (da Costa coll.).

####### Remarks.

da Costa described this species ”from an unique specimen”; the specimen found is thus the holotype. The top of the specimen is slightly damaged and appears to be subadult.

####### Current systematic position.

Bulimulidae, *Drymaeus (Drymaeus) spadiceus* da Costa, 1906.

###### Bulimus spectatus

Reeve, 1849

http://species-id.net/wiki/Bulimus_spectatus

Figs 48G–I , L56ii

Bulimus spectatus Reeve 1849 [1848–1850]: pl. 81 fig. 601a; Breure 1979: 114 (lectotype designation).
Drymaeus spectatus ; Linares and Vera 2012: 191.

####### Type locality.

”New Granada”.

####### Label.

”New Granada”.

####### Dimensions.

Not given; figured specimen herein H 39.2, D 17.0, W 6.3.

####### Type material.

NHMUK 1874.12.11.226, lectotype (ex Mrs. T. Lombe Taylor).

####### Remarks.

Reeve described this taxon from ”Mus. Taylor” and figured three different shells. The specimen selected lectotype by Breure (1979) corresponds to fig. 601a; the other two figures do not belong to this species. The distribution of this—easily misinterpreted—taxon as given by Linares and Vera (2012), entirely based on records from literature, need to be viewed with suspicion.

####### Current systematic position.

Bulimulidae, *Drymaeus (Drymaeus) spectatus* (Reeve, 1849).

###### Bulimus spiculatus

Morelet, 1860

http://species-id.net/wiki/Bulimus_spiculatus

Figs 3B–C , L56iii

Bulimus spiculatus Morelet 1860: 375; Morelet 1863: 203, pl. 11 fig. 3; Breure 1979: 58.
Bulimulus (Peronaeus) spiculatus ; Pilsbry 1896 [1895–1896]: 144, pl. 45 fig. 29.
Bostryx spiculatus spiculatus ; Breure 1978: 122 (lectotype designation).

####### Type locality.

[Peru] ”[intimâ Peruviii regionae]”; see remarks.

####### Label.

”Pérou Ollantaïtambo”, in Morelet’s handwriting.

####### Dimensions.

”Longit. 20; diam. 5 mill.”; figured specimen herein H 23.3, D 5.07, W 11+.

####### Type material.

NHMUK 1893.2.4.1156, lectotype; 1893.2.4.1157–1160, four paralectotypes (Morelet coll.).

####### Remarks.

Morelet (1860) did not state on how many specimens his description was based. In Morelet (1863) the locality is specified as ”la vallée d’Ollantaïtambo”. The top of the lectotype is damaged.

####### Current systematic position.

Bulimulidae, *Bostryx spiculatus spiculatus* (Morelet, 1860).

###### Helix sporadica

d’Orbigny, 1835

http://species-id.net/wiki/Helix_sporadica

Figs 64E–F , L56iv

Helix sporadica d’Orbigny 1835: 12 [partim].
Bulimus sporadicus d’Orbigny 1837 [1834–1847]: 271, pl. 32 figs 12–15 [3 April 1837; text 23 April 1838] [partim]; Gray 1854: 15.
Bulimulus (Bulimulus) bonariensis sporadicus ; Breure 1975b: 1145 [partim]; Miquel 1991: 101, figs 39–42.

####### Type locality.

”provincia Corrientes (republica Argentina); provincia Chiquitensi (republica Boliviana)”; see remarks.

####### Label.

”Corrientes. rep. Argentina [.158]”, ”Chiquitos Bolivia [.159–160]”, all in d’Orbigny’s handwriting.

####### Dimensions.

”Longit. 33 millim.; latit. 14 ad 17 mill.”; figured specimen herein H 27.8, D 12.8, W 7.6.

####### Type material.

NHMUK 1854.12.4.160, lectotype and six paralectotypes; 1854.12.4.158, seven specimens (see remarks); 1854.12.4.159, six paralectotypes (d’Orbigny coll.); see remarks.

####### Remarks.

d’Orbigny (1838 [1834–1847]: 271–272) mentioned variation observed in specimens from different localities, without assigning specific varietal names or signs. Three lots are present in the NHMUK collection, resp. seven (1854.12.4.158), six (.159) and seven specimens (.160). The largest specimen (in lot. 158) measures H 39.8, D 15.8, W 8.6, and corresponds to the ”variété la plus allongée”; this variety from northern Argentina is considered by Miquel (1991: 95, 98) as *Bulimulus bonariensis bonariensis* (Rafinesque, 1833). Of the two other lots—from central Bolivia—one specimen, corresponding to d’Orbigny 1837 [1834–1847]: pl. 32 figs 12–13, is here selected lectotype (**design. n.**) to define the taxon. Breure (1975b) listed in total 15 syntypes in the MNHN collection; Miquel (1991) only considered those of ”var. B” to belong to this taxon.

####### Current systematic position.

Bulimulidae, *Bulimulus bonariensis sporadicus* (d’Orbigny, 1835).

###### Bulimus stenacme

Pfeiffer, 1857

http://species-id.net/wiki/Bulimus_stenacme

Figs 12F , L56v

Bulimus stenacme Pfeiffer 1857c: 333; Pfeiffer 1859b: 492; Pfeiffer 1869 [1866–1869]: 464, pl. 101 figs 12–13; Breure 1979: 58.
Bulimulus (Lissoacme) stenacme ; Pilsbry 1896 [1895–1896]: 182, pl. 49 figs 32–33.
Bostryx stenacme ; Breure 1978: 130, pl. 9 fig. 5 (lectotype designation).

####### Type locality.

”Bolivia”.

####### Label.

”Province of Patas, / Andes of Peru / D^r^ Farris [1975342–3]”, ”Peru”, in a later handwriting; in both cases taxon label in Pfeiffer’s handwriting. M.C. label style I.

####### Dimensions.

”Long. 20 1/2, diam. 9 mil..”; figured specimen herein H 28.0, D 12.6, W 6.5.

####### Type material.

NHMUK 1975342, lectotype; 1975343, two paralectotypes, Farris leg.; 1975344, three paralectotypes (Cuming coll.).

####### Remarks.

Pfeiffer (1857) described this taxon from Cuming’s collection, but did not state on how many specimens his description was based. In Pfeiffer (1859) he emendated his measurements and locality to ”Long. 20 1/2–28, diam. 9–11 mill.” resp. ”Bolivia; in prov. Patas, Peru (Farris)”. Subsequently, in Pfeiffer (1869 [1866–1869]) he only gave ”Patas Perurvae [sic] (Farris)” as locality; the figured specimens by him belong to lot 1975344. His definition of this taxon might have been shifted over these years. The lot from Bolivia could not be traced in the NHMUK collection. The lectotype chosen by Breure (1978) was collected by Farris in Peru. Richardson (1995: 35) considered this taxon as junior subjective synonym of *Bulimus nigropileatus* Reeve, 1849. We tentatively regard this as a different—but evidently closely allied—species.

####### Current systematic position.

Bulimulidae, *Bostryx stenacme* (Pfeiffer, 1857).

###### Bulinus strigatus

Sowerby I, 1833

http://species-id.net/wiki/Bulinus_strigatus

Figs 29J–L , L57i

Bulinus strigatus Sowerby I 1833 [Sowerby I and II 1832–1841]: figs 95–96.
Drymaeus strigatus ; Pilsbry 1898 [1897–1898]: 228, pl. 42 figs 39–40.

####### Type locality.

[Peru] ”Huallaga”.

####### Label.

”Huallaga, Peru”, added in a later hand; taxon label in Pfeiffer’s handwriting. M.C. label style I.

####### Dimensions.

Not given; figured specimen herein H 24.9, D 13.7, W 6.5.

####### Type material.

NHMUK 20090168, three possible syntypes (Cuming coll.).

####### Remarks.

Sowerby likely based his material on the Cuming collection; however, none of his material has been traced with certainty. The specimens found, and labelled by Pfeiffer, are therefore treated as possible syntypes; they are partly damaged.

####### Current systematic position.

Bulimulidae, *Drymaeus (Drymaeus) strigatus* (Sowerby I, 1833).

###### Bulimus studeri

Pfeiffer, 1847

http://species-id.net/wiki/Bulimus_studeri

Figs 18O–P , L57ii

Bulimus studeri Pfeiffer 1847: 112; Pfeiffer 1848b: 107; Reeve 1848 [1848–1850]: pl. 57 fig. 384; Pfeiffer 1853d: 347; Breure 1979: 124 (lectotype designation).
Drymaeus studeri ; Pilsbry 1898 [1897–1898]: 246, pl. 43 fig. 69.
Drymaeus (Mesembrinus) studeri ; Breure and Eskens 1981: 88.

####### Type locality.

”Central America? (H. Cuming)”.

####### Label.

”Merida / New Granada”, taxon label in Pfeiffer’s handwriting. M.C. label style IV.

####### Dimensions.

”Long. 25, diam. 10 mill.”; figured specimen herein H 25.2, D 11.24, W 6.6.

####### Type material.

NHMUK 1975480, lectotype; 1975481, two paralectotypes (Cuming coll.).

####### Remarks.

Pfeiffer based himself on material from Cuming’s collection, but did not state on how many specimens his description was based. The type locality was corrected to ”Merida / Novae Granadae” in Pfeiffer 1853d: 347. The lectotype chosen by Breure (1979) corresponds to Reeve’s figure. This taxon has been misclassified as *Leiostracus* by Richardson (1995: 207). The material mentioned as *Drymaeus studeri* from Colombia by Linares and Vera (2012: 200), may only partially belong to that species. Unpublished data from Breure and Borrero strongly suggest that further morphological and molecular studies are needed to ascertain the status of this taxon and some taxa that have been associated with it in literature.

####### Current systematic position.

Bulimulidae, *Drymaeus (Mesembrinus) studeri* (Pfeiffer, 1847).

###### Bulimus subfasciatus

Pfeiffer, 1853

http://species-id.net/wiki/Bulimus_subfasciatus

Figs 70E–F , 70M , L57iii

Bulimus subfasciatus Pfeiffer 1853d: 408; Pfeiffer 1854 in Küster and Pfeiffer 1840–1865: 105, pl. 33 fig. 19; Pfeiffer 1854a: 60; Breure 1979: 88.
Bulimulus subfasciatus ; Pilsbry 1897 [1897–1898]: 33, pl. 8 fig. 33.
Scutalus (?*Kuschelenia*) *subfasciatus*; Breure 1978: 185, pl. 9 fig. 8.
Scutalus (Vermiculatus) anthisanensis ; Breure and Borrero 2008: 18.

####### Type locality.

[Ecuador] ”repulicae Aequatoris, monte Anthisana”.

####### Label.

”Anthisana Equador / Monsr Bourcier”, taxon label in Pfeiffer’s handwriting. M.C. label style IV.

####### Dimensions.

”Long. 32, diam. 14 mill.”; figured specimen herein H 31.0, D 16.0, W 6.0.

####### Type material.

NHMUK 1975368, lectotype; 1975369, two paralectotypes (Cuming coll.).

####### Remarks.

This taxon is now placed in the genus *Kuschelenia*; the synonymy follows Weyrauch (1967a: 385) and Breure (1978: 185).

####### Current systematic position.

Bulimulidae, *Kuschelenia (Vermiculatus) anthisanensis* (Pfeiffer, 1853) (**comb. n.**).

###### Gonyostomus subhybridus

da Costa, 1906

http://species-id.net/wiki/Gonyostomus_subhybridus

Figs 46G–I , L57iv

Gonyostomus subhybridus da Costa 1906b: 97, pl. 9 fig. 1; Breure 1979: 114.

####### Type locality.

”Pozuzo, Eastern Peru, 800 metres”.

####### Label.

”Pozuzo. Eastern Peru. 800 M^s^”, in da Costa’s handwriting.

####### Dimensions.

”Long. 50, diam. 19 mm.”; figured specimen herein H 49.2, D 19.5, W 6+.

####### Type material.

NHMUK 1907.11.21.127, holotype (da Costa coll.).

####### Remarks.

”Of this fine shell only one specimen has been received” (da Costa 1906b: 97); the specimen is thus the holotype. The top whorl is missing. This taxon may prove to be identical to *Otostomus pulcherrimus* H. Adams, 1867.

####### Current systematic position.

Bulimulidae, *Drymaeus (Drymaeus) subhybridus* (da Costa, 1906).

###### Bulimus subinterruptus

Pfeiffer, 1853

http://species-id.net/wiki/Bulimus_subinterruptus

Figs 49O , L57v

Bulimus subinterruptus Pfeiffer 1853b: 256; Pfeiffer 1853 in Küster and Pfeiffer 1840–1865: 80, pl. 21 fig. 20–21; Pfeiffer 1853d: 333; Breure 1979: 115 (lectotype designation).

####### Type locality.

”in Andibus Boliviae”.

####### Label.

”Andes, N. Granada”, taxon label in Pfeiffer’s handwriting. M.C. label style IV.

####### Dimensions.

”Long. 37, diam. 13 1/1 mill.”; figured specimen herein H 36.7, D 15.7, W 6.6.

####### Type material.

NHMUK 1975470, lectotype (Cuming coll.).

####### Remarks.

Pfeiffer described this species from Cuming’s collection; he did not state on how many specimens his description was based. The type locality may have been in error, as Pfeiffer (1853 [1840–1865]: 80) corrected it to ”Anden von Neu-Granada”. In the same work Pfeiffer recognized a smaller variety (figured on pl. 21 figs 22–23; these figures were copied by Pilsbry 1898 [1897–1898]: pl. 41 figs 34–35, who did not illustrate the typical form). Breure and Borrero (unpublished data) were unable, however, to recognize this taxon in the material they revised from Colombia.

####### Current systematic position.

Bulimulidae, *Drymaeus (Drymaeus) subinterruptus* (Pfeiffer, 1853).

###### Bulimulus (Liostracus) subpellucidus

E.A. Smith, 1877

http://species-id.net/wiki/Bulimulus_subpellucidus

Figs 21G , L57vi

Bulimulus (Liostracus) subpellucidus E.A. Smith 1877: 364, pl. 39 fig. 2.
Drymaeus subpellucidus ; Pilsbry 1898 [1897–1898]: 288; Pilsbry 1899: pl. 5 fig. 1.

####### Type locality.

”Ecuador”.

####### Label.

”Ecuador”.

####### Dimensions.

”Long. 23 mill. diam. 9”; figured specimen herein H 20.0, D 10.0, W 4+.

####### Type material.

NHMUK 1872.5.22.19, one syntype.

####### Remarks.

Smith did not state on how many specimens his description was based. The label states ”type”, but this cannot be interpreted as holotype designation; there is also remarked ”Purch[ased] of Cutter”, and ”1/6” in the lower right corner of the label. The top of the specimen is broken, hence the lower shell height than the measurement given by Smith.

####### Current systematic position.

Bulimulidae, *Drymaeus (Mesembrinus) subpellucidus* (E.A. Smith, 1877).

###### Drymaeus subventricosus

da Costa, 1901

http://species-id.net/wiki/Drymaeus_subventricosus

Figs 33J–L , L57vii

Drymaeus subventricosus da Costa 1901: 239, pl. 24 fig. 4; Pilsbry 1901 [1901–1902]: 156, pl. 48 fig. 48; Breure 1979: 115; Linares and Vera 2012: 191.

####### Type locality.

”Bogota, Colombia”.

####### Label.

”Bogotá”, in da Costa’s handwriting.

####### Dimensions.

”Long. 30, diam. 14 mm.”; figured specimen herein H 30.1, D 14.0, W 6.6.

####### Type material.

NHMUK 1907.11.21.37, lectotype (da Costa coll.).

####### Remarks.

da Costa did not state on how many specimens his description was based; the reference of Breure (1979) to ”HT BMNH 1907.11.21.37” has to be interpreted as a lectotype designation under Art. 74.6 ICZN.

####### Current systematic position.

Bulimulidae, *Drymaeus (Drymaeus) subventricosus* da Costa, 1901.

###### Bulimus sugillatus

Pfeiffer, 1857

http://species-id.net/wiki/Bulimus_sugillatus

Figs 16C , L58ii

Bulimus sugillatus Pfeiffer 1857d: 389; Pfeiffer 1859b: 490.
Anctus sugillatus ; Breure and Schouten 1985: 6, pl. 1 figs 5–7 (lectotype designation).

####### Type locality.

”Bolivia”.

####### Label.

”Bolivia”, taxon label in Pfeiffer’s handwriting. M.C. label style III.

####### Dimensions.

”Long. 24, diam. 9 1/2 mill.”; figured specimen herein H 23.7, D—, W 8.3.

####### Type material.

NHMUK 1975259, lectotype and two paralectotypes (Cuming coll.).

####### Remarks.

Restudy of the type lot reveals that the protoconch sculpture consists of axial riblets with fine, spiral lines in between. This characteristic makes us now to classify this taxon with *Naesiotus* sensu Breure 1979 (**comb. n.**). We consider Pfeiffer’s taxon to be a junior subjective synonym of *Bulimus rivasii* d’Orbigny, 1837 (**syn. n.**).

####### Current systematic position.

Bulimulidae, *Naesiotus rivasii* (d’Orbigny, 1837).

###### Bulimus sulcosus

Pfeiffer, 1841

http://species-id.net/wiki/Bulimus_sulcosus

Figs 23O , L58i

Bulimus sulcosus Pfeiffer 1841: 43; Pfeiffer 1843 in Philippi 1842–1845: 56, pl. 1 fig. 9; Pfeiffer 1848b: 196.

####### Type locality.

[Mexico] ”prope Tacubaya Mexicanorum”.

####### Label.

”Mexico”, taxon label in Pfeiffer’s handwriting. M.C. label style I.

####### Dimensions.

”Long. 15 1/2, diam. 7 lin. [H 31, D 13.5 mm]”; figured specimen herein H 37.0, D 16.5, W 6.5.

####### Type material.

NHMUK 20110084, two possible syntypes (Cuming coll.).

####### Remarks.

Pfeiffer probably described this taxon from a specimen in his own collection, which is likely to be lost; Pfeiffer (1843 in Philippi 1842–1845: 56) stated the specimen(s) to have been collected by Hegewisch. The specimens from Cuming’s collection also bear Pfeiffer’s handwriting and are probably the only extant ones that confirms his identification; they are considered as probable syntypes. The current systematic position follows Thompson (2011: 112).

####### Current systematic position.

Bulimulidae, *Drymaeus (Mesembrinus) sulcosus* (Pfeiffer, 1841).

###### Bulimus sulphureus

Pfeiffer, 1857

http://species-id.net/wiki/Bulimus_sulphureus

Figs 20D , L58iv

Bulimus sulphureus Pfeiffer 1857b: 318, pl. 35 fig. 11.

####### Type locality.

[Mexico, Edo. Veracruz] ”Cordova”.

####### Label.

”Cordova (Mexico)”, taxon label in Pfeiffer’s handwriting. M.C. label style I.

####### Dimensions.

”Long. 29, diam. 12 mill.”; figured specimen herein H 28.7, D 13.6, W 6.5.

####### Type material.

NHMUK 20100585, lectotype and two paralectotypes (Cuming coll.).

####### Remarks.

Pfeiffer described this taxon on the basis of specimens collected by Sallé and kept in the Cuming collection; Pfeiffer did not state on how many specimens his description was based. One of the specimens found corresponds to the figure and original shell height mentioned by Pfeiffer, and is here designated lectotype to define the taxon (**design. n.**). The current systematic position follows Thompson (2011: 119).

####### Current systematic position.

Bulimulidae, *Drymaeus (Mesembrinus) sulphureus* (Pfeiffer, 1857).

###### Bulimus swainsoni

Pfeiffer, 1845

http://species-id.net/wiki/Bulimus_swainsoni

Figs 75G , L59i

Bulinus melanostoma [Swainson, 1820] var. Sowerby I 1838 [Sowerby I and II 1832–1841]: fig. 88.
Bulimus swainsoni Pfeiffer 1845d: 156
Bulimus melanostoma ; Reeve 1848 [1848–1850]: pl. 33 fig. 203b.

####### Type locality.

”Brasilia”.

####### Label.

”Brazil”. M.C. label style I, V.

####### Dimensions.

”Long. 61. diam. 23 mill.”; figured specimen herein H 60.0, D 37.2, W 5+.

####### Type material.

NHMUK 20100586, three probable syntypes (Cuming coll.).

####### Remarks.

Pfeiffer referred in his original description to ”*Bul. melanost. var. Conch. Ill. f. 88*”; this figure was likely published in 1838 (Coan et al. 2013a) and was probably based on material present in the collection of H. Cuming. The material found was purchased by Cuming, and one of the specimens fits the shell height mentioned by Pfeiffer. The material has also been figured by Reeve and has a taxon label in Pfeiffer’s handwriting. The specimens are considered as probable syntypes. The current systematic position follows Simone (2006: 131); however, the classification of *Auris* with the Bulimulidae needs further confirmation from molecular studies.

####### Current systematic position.

Bulimulidae, *Auris chrysostoma* (Moricand, 1836).

###### Drymaeus sykesi

da Costa, 1906

http://species-id.net/wiki/Drymaeus_sykesi

Figs 35J–L , L58iii

Drymaeus sykesi da Costa 1906a: 7, pl. 1 fig. 1; Breure 1979: 124; Linares and Vera 2012: 200.

####### Type locality.

[Colombia] ”Bogotá”.

####### Label.

”Bogota”, in da Costa’s handwriting.

####### Dimensions.

”Long. 52, diam. 19 mm.”; figured specimen herein H 51.7, D 19.8, W 6+.

####### Type material.

NHMUK 1907.11.21.4, holotype (da Costa coll.).

####### Remarks.

da Costa described this species ”from a single specimen”; the specimen found is thus the holotype. The top whorls are missing.

####### Current systematic position.

Bulimulidae, *Drymaeus (Drymaeus) sykesi* da Costa, 1906.

###### Bulimus tenuilabris

Pfeiffer, 1866

http://species-id.net/wiki/Bulimus_tenuilabris

Figs 21F , L59ii

Bulimus tenuilabris Pfeiffer 1866: 831; Breure 1979: 124 (lectotype designation).
Drymaeus (Mesembrinus) tenuilabris ; Breure and Eskens 1981: 88, pl. 7 fig. 8.

####### Type locality.

”Venezuela”.

####### Label.

”Venezuela”, taxon label in Pfeiffer’s handwriting. M.C. label style I.

####### Dimensions.

”Long. 30, diam. 12 mill.”; figured specimen herein H 30.2, D 15.1, W 7.2.

####### Type material.

NHMUK 1975338, lectotype (Cuming coll.).

####### Remarks.

The current systematic position agrees with Richardson (1995: 183).

####### Current systematic position.

Bulimulidae, *Drymaeus (Mesembrinus) tenuilabris* (Pfeiffer, 1866).

###### Bulimulus (Pleuropyrgus) terebra

Reibisch, 1892

http://species-id.net/wiki/Bulimulus_terebra

Figs 55G , L59iii

Bulimulus (Pleuropyrgus) terebra Reibisch 1892: 24, pl. 2 fig. 3.

####### Type locality.

”Chatam-Island [sic] (Wolf)”.

####### Label.

”Chatham I^s^ / Galapagos”.

####### Dimensions.

”Long. 18–19, diam. maj. 4.67 mm”; figured specimen herein H 19.1, D 4.5, W 15.2.

####### Type material.

NHMUK 1894.6.8.2, one possible syntype, T. Wolf leg. (ex Reibisch).

####### Remarks.

Reibisch described this taxon from four specimens. The current systematic position accounts the fact that Dall published his *Bulimulus (Pleuropyrgus) habeli* ‘Stearns ms.’ in January 1892, while Reibisch’ taxon has not been associated with a specific date and hence has to be dated as 31 December 1892.

####### Current systematic position.

Bulimulidae, *Naesiotus habeli* (Dall, 1892).

###### Bulimus terebralis

Pfeiffer, 1842

http://species-id.net/wiki/Bulimus_terebralis

Figs 2M–N , L60i

Bulimus terebralis Pfeiffer 1842: 51; Pfeiffer 1843a: 187; Reeve 1848 [1848–1850]: pl. 14 fig. 79.
Bulimulus (Peronaeus) terebralis ; Pilsbry 1896 [1895–1896]: 142, pl. 45 fig. 30.
Bulimulus ischnus Pilsbry 1902: lxxi. New name for *Bulimus terebralis* Pfeiffer, 1842 not Bruguière, 1789.

####### Type locality.

”Coquimbo, Chile”.

####### Label.

”Chili”. M.C. label style I.

####### Dimensions.

”Long. 21, diam. 4 1/2 mill.”; figured specimen herein H 19.8, D 5.0, W 10.0.

####### Type material.

NHMUK 20110082, five probable syntypes (Cuming coll.).

####### Remarks.

Pfeiffer described this species from specimens collected by Mr. Bridges [a son-in-law of H. Cuming]; he did not state on how many specimens his description was based. The material was likely deposited in the Cuming collection and one specimen corresponds to Reeve’s figure. The material is herein considered as probable syntypes. The current systematic position follows Richardson (1995: 29).

####### Current systematic position.

Bulimulidae, *Bostryx ischnus* (Pilsbry, 1902).

###### Helix thamnoica

d’Orbigny, 1835

http://species-id.net/wiki/Helix_thamnoica

Figs 68A , L60ii

Helix thamnoica d’Orbigny 1835: 16.
Bulimus thamnoicus d’Orbigny 1837 [1834–1847]: 290, pl. 37 figs 4–9 [5 June 1837; text 6 May 1838].
Scutalus thamnoicus ; Breure 1975b: 1144, pl. 4 fig. 1.

####### Type locality.

”Var. A. [Not given]. Var. B. [Not given]. Var. C. Cavari (republica Boliviana). Var. D. provincia Chuquisacensi (republica Boliviana)”; see remarks and Breure 1973: 123.

####### Label.

”Var. A. Palca d’ayupaya Bolivia”, ”Var. B. Cochabamba, Bolivia”, ”Var. C. Cavari, Bolivia”, ”Var. D chuquisaca Bolivia”, all in d’Orbigny’s handwriting.

####### Dimensions.

”Longit. a 45 ad 80 millim.; latit. a 22 ad 39 millim.”, corrected in d’Orbigny 1837 [1834–1847] to: ”Long. ex 25 ad 65 millim.; lat. ex. 15 ad 35 millim.”; figured specimen herein H 44.0, D 25.3, W 6.8.

####### Type material.

NHMUK 1854.12.4.111, five paralectotypes ”var. A.”; 1854.12.4.109, six paralectotypes ”var. B.”; 1854.12.4.107, four paralectotypes [var. B]; 1854.12.4.110, nine syntypes ”var. C.”; 1854.12.4.108, lectotype (**design. n.**) and four paralectotypes ”var. D.” (all material d’Orbigny coll.).

####### Remarks.

d’Orbigny’s varieties have proven to belong to different species, already noticed by Pilsbry (1897 [1897–1898]) and Richardson (1995), although they disagreed on the specific assignments of the four varieties. Breure (1975b) mentioned only material pertaining to the varieties A and B in the Paris museum; however, also material corresponding to variety D is available in this collection (Breure, unpublished data). The data presented in d’Orbigny 1837–1838 [1834–1847], together with the material of both the NHMUK and MNHN collections, are used here to resolve this complicated issue.

Var. A. ”*Magna*, *reticulata*” (pl. 37 figs 4–5) is recorded from ”près de Palca, capitale de la province d’Ayupaya” [Dept. Cochabamba, Indepencia; see Breure 1973: 133, fig. 6] and ”Longueur, 45 millimètres”. The lectotype (MNHN 23258) has a shell height of 47.7 mm; the largest specimen in NHMUK labelled ”var. A” has shell height 44 mm. The name *thamnoica* d’Orbigny is restricted to this variety.

Var. B. ”*Magna*, *crassa*, *striata*” (pl. 37 fig. 7) is listed from ”environs de Capiñata [Dept. La Paz]” and from ”vallée de Cochabamba (...) principalement à Viloma. Longueur, 65 millimètres”; see Breure 1973: 133, figs 6–7. The largest specimen in lot NHMUK 1854.12.4.109 measures 52.7 mm in height (Fig. 68B); note that there is also material without variety designation (1854.12.4.107), of which the largest specimen has a shell height of 61.5 mm. This variety is considered to be identical with *Bulimus alauda* Hupé, 1857.

Var. C. ”*Minor*, *fasciata*” (pl. 37 fig. 8) has been found at ”les coteaux du bourg de Cavari”, near the top of the mountains; see Breure 1973: 129, fig. 7. ”Longueur, 25 millimètres”. The largest specimen in NHMUK has dimensions H 32.2, D 19.0, W 6.3. This variety was considered a synonym of *Bulimus revinctus* Hupé, 1857 by both Pilsbry (1897 [1897–1898]) and Richardson (1995). We consider this variety as *Bulimus gayi* Pfeiffer, 1857.

Var. D. ”*Marmorata*” (pl. 37 fig. 9) was collected ”aux environs de la ville de Chuquisaca”; see Breure 1973: 130, fig. 4. No shell height was given by d’Orbigny. The largest NHMUK specimen, corresponding to d’Orbigny’s figure, has dimensions H 40.8, D 23.1, W 6.2 (Fig. 68C). Three specimens are present in Paris (MNHN 23261), of which the largest one with dimensions H 45.3, D 23.9, W 6.7. Pilsbry (1897 [1897–1898]) regarded var. D as synonymous with *Bulimus alauda* Hupé, 1857; Richardson (1995) synonymyzed it with *Bulimus revinctus* Hupé, 1857. The types of both species have not been located, leaving only the possibility to check against Hupe’s figures (1857: pl. 7 fig. 3 resp. pl. 7 fig. 2), in which case we concur with Pilsbry’s view.

####### Current systematic position.

Bulimulidae, *Kuschelenia (Kuschelenia)* species. See under remarks.

###### Bulimulus (Drymaeus) tigrinus

da Costa, 1898

http://species-id.net/wiki/Bulimulus_tigrinus

Figs 36J–L , L60iv

Bulimulus (Drymaeus) tigrinus da Costa 1898: 82, pl. 6 fig. 6; Breure 1979: 115.
Drymaeus tigrinus ; Pilsbry 1898 [1897–1898]: 231, pl. 50 fig. 88;
Drymaeus (Drymaeus) tigrinus ; Breure and Borrero 2008: 23.

####### Type locality.

”Ecuador”.

####### Label.

”Equador”, in da Costa’s handwriting.

####### Dimensions.

”Long. 21, diam. 10 mm.”; figured specimen herein H 20.8, D 9.96, W 6.3.

####### Type material.

NHMUK 1907.11.21.55, lectotype; 1907.11.21.56, one paralectotype (da Costa coll.).

####### Remarks.

da Costa did not state on how many specimens his description was based; the material was said to be from ”Buckley’s Coll.”. The reference of Breure (1979) to ”HT BMNH 1907.11.21.55” has to be interpreted as a lectotype designation under Art. 74.6 ICZN.

####### Current systematic position.

Bulimulidae, *Drymaeus (Drymaeus) tigrinus* (da Costa, 1898).

###### Helix torallyi

d’Orbigny, 1835

http://species-id.net/wiki/Helix_torallyi

Figs 11D , L60iii

Helix torallyi d’Orbigny 1835: 11.
Bulimus torallyi d’Orbigny 1837: 285, pl. 32 figs 1–4 [5 June 1837; text 6 May 1838].
Bulimulus (?) *torallyi* (d’Orbigny); Breure 1975b: 1146, pl. 8 fig. 4 [corresponding to d’Orbigny 1837: pl. 32 figs 1–2].
Drymaeus cf. *draparnaudi* (Pfeiffer); Breure 1975b: 1150, pl. 8 fig. 2 [corresponding to d’Orbigny 1837: pl. 32 figs 3–4].
Bostryx torallyi (d’Orbigny); Breure 1979: 59; Cuezzo et al. 2013: 146.

####### Type locality.

”provincia Valle-Grande, republica Boliviana” [Bolivia, Dept. Santa Cruz, Prov. Valle Grande].

####### Label.

”pampa ruis (Laguna) Bolivia”; in d’Orbigny’s handwriting.

####### Dimensions.

”Long. 31 millim.; lat. 11 millim.”; figured specimen H 28.6, D 11.3, W 8.5 [.191].

####### Type material.

”var. A.” NHMUK 1854.12.4.191, seven paralectotypes; ”var. B.” NHMUK 1854.12.4.192, nine paralectotypes.

####### Remarks.

**Figure 4.**
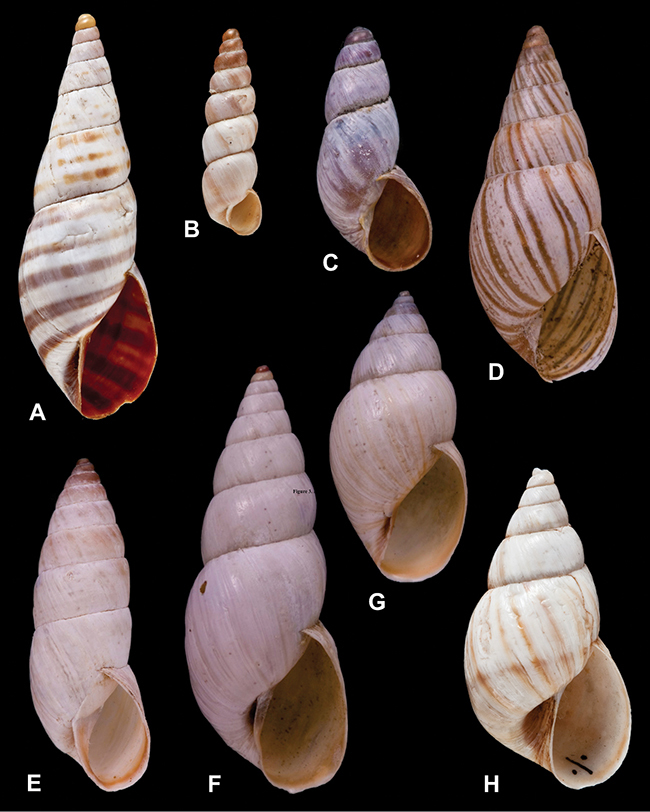
*Bostryx* species. **A** *Bostryx rodriguezae* Weyrauch, 1967, paratype NHMUK 1975355 (H = 25.5) **B** *Bostryx rehderi* Weyrauch, 1960, paratype NHMUK 1975356 (H = 10.45) **C** *Bostryx devians* (Dohrn, 1863), syntype NHMUK 1975339 (H = 14.9) **D** *Bostryx mordani* Breure, 1978, paratype NHMUK 1975266 (H = 22.5) **E** *Bostryx agueroi* Weyrauch, 1960, paratype NHMUK 1975333 (H = 21.1) **F–H** *Bostryx orophilus* (Morelet, 1860) **F** *Bulimus albicolor* Morelet, 1863, syntype NHMUK 1893.2.4.169 (H = 28.2) **G** *Bulimus cercicola* Morelet, 1863, syntype NHMUK 1893.2.4.175 (H = 18.7) **H** *Bulimus orophilus* Morelet, 1860, lectotype NHMUK 1893.2.4.188 (H = 20.8). All enlarged.

Breure (1975b) found type material in the Paris museum and concluded that it belonged to two different species, one lot corresponding to pl. 32 figs 1–2 (this is d’Orbigny’s var. A) and one lot to figs 3–4 (var. B). In the London museum all specimens proved to belong to the same taxon, despite their being labelled with different variety letters; however, they represent different colour forms.

####### Current systematic position.

Bulimulidae, *Bostryx torallyi* (d’Orbigny, 1835).

###### Bulinus translucens

Broderip in Broderip and Sowerby I 1832

http://species-id.net/wiki/Bulinus_translucens

Figs 18R , L61i

Bulinus translucens Broderip in Broderip and Sowerby I 1832a: 31; Sowerby I 1833 [Sowerby I and II 1832–1841]: fig. 11; Reeve 1848 [1848–1850]: pl. 13 fig. 71.

####### Type locality.

”in America Meridionali (King’s and Saboga Islands, Bay of Panama)”.

####### Label.

”Panama” (see remarks). M.C. label style I.

####### Dimensions.

”long. 7/8, lat. 4/8 poll. [H 22.2, D 12.66 mm]”; figured specimen herein H 21.2, D 11.7, W 5.3.

####### Type material.

NHMUK 1842.5.10.139, eight paralectoypes; 20100634, lectotype and three paralectotypes (Cuming coll.).

####### Remarks.

Two lots were found, of which one has a taxon label ”*B. translucidus*” possibly written in Broderip’s hand; note the different spelling compared to the published taxon name. The other lot has been used by Reeve for his fig. 71; this lot has a taxon label in Pfeiffer’s handwriting. All material consists mostly of subadults and juveniles, but one of adult shells agrees with Broderip’s shell height and is here designated lectotype (**design.n.**) to define the taxon.

####### Current systematic position.

Bulimulidae, *Drymaeus (Mesembrinus) translucens* (Broderip in Broderip and Sowerby I 1832).

###### Bulimus transparens

Reeve, 1849

http://species-id.net/wiki/Bulimus_transparens

Figs 63G–H , L60v

Bulimus transparens Reeve 1849 [1848–1850]: pl. 77 fig. 566; Breure 1979: 64.
Bulimulus transparens ; Pilsbry 1897 [1897–1898]: 73, pl. 11 fig. 11; Breure 1978: 147 (lectotype designation).

####### Type locality.

”—?”.

####### Label.

”Venezuela”, added in a later hand. M.C. label style I.

####### Dimensions.

Not given; figured specimen herein H 18.4, D 8.38, W 6.3.

####### Type material.

NHMUK 1975397, lectotype (Cuming coll.).

####### Remarks.

Reeve described this taxon from ”Mus. Cuming”, but did not give a locality. It is not clear if the locality added during later years may be trusted. This is a subadult specimen and the taxon may prove to be synonymous with one of the many similar-looking other *Bulimulus* taxa occurring in this region.

####### Current systematic position.

Bulimulidae, *Bulimulus transparens* (Reeve, 1849).

###### Helix trichoda

d’Orbigny, 1835

http://species-id.net/wiki/Helix_trichoda

Figs 16D–F , L60vi

Helix trichoda d’Orbigny 1835: 12.
Bulimus trichodes ; d’Orbigny 1837 [1834–1847]: 277, pl. 33 figs 1–5 [19 June / 7 Aug 1837; text 23 April 1838]; Gray 1854: 16.
Naesiotus trichodes ; Breure 1975b: 1147.

####### Type locality.

”provincia Santa Cruz de la Sierra (republica Boliviana)”.

####### Label.

”S^ta^ Cruz (Bolivia)”. in d’Orbigny’s handwriting.

####### Dimensions.

”Longit. 20 millim.; latit. 10 millim.”; figured specimen herein H 20.8, D 9.06, W 7.7.

####### Type material.

NHMUK 1854.12.4.175, lectotype and four paralectotypes (d’Orbigny coll.).

####### Remarks.

d’Orbigny (1837 [1834–1847]: 277) specified this species to be found in gardens of Santa Cruz de la Sierra city. Breure (1975b) mentioned four syntypes in the MNHN collection. One of the specimens in NHMUK corresponds to d’Orbigny 1837 [1834–1847]: pl. 33 fig. 3 and has been selected as lectotype (**design. n.**) to define the taxon.

####### Current systematic position.

Bulimulidae, *Naesiotus trichodes* (d’Orbigny, 1835).

###### Bulimus tricinctus

Reeve, 1848

http://species-id.net/wiki/Bulimus_tricinctus

Figs 11F , L61ii

Bulimus tricinctus Reeve 1848 [1848–1850]: pl. 57 fig. 380; Breure 1979: 59.
Bostryx tricinctus ; Breure 1978: 132 (lectotype designation).

####### Type locality.

”.—?”.

####### Label.

No locality. M.C. label style III.

####### Dimensions.

Not given; figured specimen herein H 19.2, D 8.94, W 7.0.

####### Type material.

NHMUK 1975182, lectotype (Cuming coll.).

####### Remarks.

Reeve described this taxon from the Cuming collection, without giving a locality. The figured specimen was selected lectotype by Breure (1978) and placed in *Bostryx* *s. l.*.

####### Current systematic position.

Bulimulidae, *Bostryx tricinctus* (Reeve, 1848).

###### Otostomus trimarianus

Martens, 1893

http://species-id.net/wiki/Otostomus_trimarianus

Figs 22D , L61iii

Otostomus trimarianus Martens 1893 [1890–1901]: 216, pl. 13 fig. 17; Breure 1979: 124 (lectotype designation).
Drymaeus trimarianus ; Pilsbry 1899: 62, pl. 2 figs 17–18.
Drymaeus (Mesembrinus) trimarianus ; Thompson 2011: 116.

####### Type locality.

”N.W. Mexico: Tres Marias Islands”.

####### Label.

”Tres Marias / Richardson”.

####### Dimensions.

”Long. 32, diam. 14 (...) millim.”; figured specimen herein H 31.5, D 13.5, W 6.7.

####### Type material.

NHMUK 1901.6.22.950, lectotype (Godman coll.).

####### Remarks.

The current systematic position is according to Thompson (2011).

####### Current systematic position.

Bulimulidae, *Drymaeus (Mesembrinus) trimarianus* (Martens, 1893).

###### Bulimulus (Drymaeus) trinitarius

E.A. Smith, 1896

http://species-id.net/wiki/Bulimulus_trinitarius

Figs 21C , L61iv

Bulimulus (Drymaeus) trinitarius E.A. Smith 1896: 242, pl. 8 figs 7–7a.

####### Type locality.

”[Trinidad]”.

####### Label.

”Trinidad”.

####### Dimensions.

”Longit. 19 millim., diam. 9”; figured specimen herein H 20.1, D 9.84, W 6.1.

####### Type material.

NHMUK 1875.2.8.2, lectotype and one paralectotype; 1866.1.3.13 (part), ten paralectotypes, both R.J.L. Guppy leg.

####### Remarks.

Smith remarked ”the only two adult specimens I have seen”. Pilsbry (1899: 19) already recognized that this taxon was a junior synonym of *Bulimulus multifasciatus imperfectus* Guppy, 1866. Type material of the latter taxon—though expected in the NHMUK—was not found. One specimen of lot 1875.2.8.2 corresponds to Smith’s fig. 7a and is here designated lectotype to define the taxon. The specimens of the other lot are all juveniles.

####### Current systematic position.

Bulimulidae, *Drymaeus (Mesembrinus) imperfectus* (Guppy, 1866).

###### Bulimus tristis

Pfeiffer, 1855

http://species-id.net/wiki/Bulimus_tristis

Figs 22B , L62i

Bulimus tristis Pfeiffer 1855g: 124; Breure 1979: 124.
Drymaeus pertristis Pilsbry 1898 [1897–1898]: 301. New name for *Bulimus tristis* Pfeiffer, 1855 not Jay, 1839.
Drymaeus tristis ; Linares and Vera 2012: 201 [partim].
Drymaeus (Mesembrinus) pertristis ; Breure and Eskens 1981: 81.

####### Type locality.

”New Granada”.

####### Label.

”New Granada”, taxon label in Pfeiffer’s handwriting. M.C. label style IV.

####### Dimensions.

”Long. 28, diam. 11 mill.”; figured specimen herein H 29.3, D 13.3, W 6.6.

####### Type material.

NHMUK 1975299, three syntypes (Cuming coll.).

####### Remarks.

Pfeiffer did not state on how many specimens his description was based. Breure and Borrero were not able to recognize this species in the material they revised from Colombia (unpublished data). The locality data in Linares and Vera (2012) need further evidence.

####### Current systematic position.

Bulimulidae, *Drymaeus (Mesembrinus) pertristis* (Pfeiffer, 1855).

###### Bulimus tropicalis

Morelet, 1849

http://species-id.net/wiki/Bulimus_tropicalis

Figs 21K , L62ii

Bulimus tropicalis Morelet 1849: 9; Breure 1979: 124.
Drymaeus (Mesembrinus) tropicalis ; Breure and Eskens 1981: 89; Thompson 2011: 121.

####### Type locality.

”ad plagam civitas Campeche”.

####### Label.

”Campeche”, in Morelet’s handwriting.

####### Dimensions.

”Long. 28—Diam. 11 [mm]”; figured specimen herein H 27.5, D 12.9, W 6.7.

####### Type material.

NHMUK 1893.2.4.210, lectotype; 1893.2.4.211–212, two paralectotypes (Morelet coll.).

####### Remarks.

Morelet did not state on how many specimens his description was based. This sinistral species may prove to be identical with one of the other, dextral, *Drymaeus* species occurring in its distribution range as enantiomorphy within this group may be more commenly found than currently thought (Breure, unpublished data).

####### Current systematic position.

Bulimulidae, *Drymaeus (Mesembrinus) tropicalis* (Morelet, 1849).

###### Bulimus tumidulus

Pfeiffer, 1842

http://species-id.net/wiki/Bulimus_tumidulus

Figs 11G , L62iii

Bulinus inflatus Broderip 1838 in Sowerby I and II 1832–1841: fig. 61.
Bulimus tumidulus Pfeiffer 1842: 117, 123; Pfeiffer 1848b: 193; Reeve 1848 [1848–1850]: pl. 19 fig. 111.
Bostryx tumidulus ; Breure 1978: 134 (lectotype designation).

####### Type locality.

Not given, see remarks.

####### Label.

”Peru”, taxon label in Pfeiffer’s handwriting. M.C. label style IV, V.

####### Dimensions.

Not given; figured specimen herein H 22.7, D 12.85, W 7.0.

####### Type material.

NHMUK 1975324, lectotype; 1975325, two paralectotypes (Cuming coll.).

####### Remarks.

Pfeiffer (1842 [1841–1846]) introduced this name as replacement for *Bulinus inflatus* Broderip, 1838 not *Bulimus inflatus* Lamarck, 1822 [which is a homonym of *Bulimus inflatus* Olivier, 1801; see Kendrick and Wilson 1975: 307]. Pfeiffer (1848) gave as locality ”juxta Ambo Peruviae” and gave as measurements ”Long. 23, diam. 12 mill.”.

####### Current systematic position.

Bulimulidae, *Bostryx tumidulus* (Pfeiffer, 1842).

###### Helix tupacii

d’Orbigny, 1835

http://species-id.net/wiki/Helix_tupacii

Figs 68D–E , L62iv

Helix tupacii d’Orbigny 1835: 16.
Bulimus tupacii ; d’Orbigny 1837 [1834–1847]: 292, pl. 38 figs 1–5 [19 June / 7 Aug 1837; text 6 May 1838]; Gray 1854: 18.
Scutalus tupacii ; Breure 1975b: 1144, pl. 2 fig. 3 (lectotype designation); Cuezzo et al. 2013: 161.

####### Type locality.

”provincia Yungasensi (republica Boliviana)”; restricted to Dept. La Paz, Yanacachi (Breure 1975b).

####### Label.

”cavari. Sicasica. Bolivia ” [.113], ”Palca d’ayapaya, Bolivia” [.114], ”yungas Bolivia ” [.115], all in d’Orbigny’s handwriting.

####### Dimensions.

”Longit. 4 centim.; latit. 2 centim.”; figured specimen herein H 76.0, D 35.3, W 6+.

####### Type material.

NHMUK 1854.12.4.113, four paralectotypes; 1854.12.4.114, five paralectotypes; 1854.12.4.115, seven paralectotypes (all d’Orbigny coll.).

####### Remarks.

d’Orbigny (1835) did not state on how many specimens his description was based. In d’Orbigny 1838 [1834–1847]: 292 he corrected the measurements as ”Long. ex 40 ad 75 millim.; lat. ex 12 ad 35 millim.”. In this paper he also specified the localities as ”principalement à Yanacache et à Chupé, dans la province de Yungas, et dans celles de Sicasica et d’Ayapaya”; see also Breure (1973: 133, fig. 7). Breure (1975b) selected a lectotype from among the syntypes, present in the MNHN collection without locality data. In the three lots at the NHMUK one specimen from lot 1854.12.4.113 can be matched to d’Orbigny’s pl. 38 fig. 5 (H 76.0, D 35.3, W 6+; top damaged), another from the same lot corresponds to pl. 38 fig. 4 (H 69.8, D 35.8, W 7.0).

####### Current systematic position.

Bulimulidae, *Kuschelenia (Kuschelenia) tupacii* (d’Orbigny, 1835) (**comb. n.**).

###### Helix turritella

d’Orbigny, 1835

http://species-id.net/wiki/Helix_turritella

Figs 63K–L , L62v

Helix turritella d’Orbigny 1835: 13. Not *Helix turritella* Férussac, 1821.
Bulimus turritella ; d’Orbigny 1837 [1834–1847]: 274, pl. 33 figs 12–14 [19 June / 7 Aug 1837; text 23 April 1838]; Gray 1854: 15.
Bulimulus turritellatus Beck 1838: 67.
Bulimulus (Lissoacme) turritellatus ; Pilsbry 1896 [1895–1896]: 193, pl. 50 figs 65–66.
Bulimulus (Bulimulus) turritellatus ; Breure 1975b: 1146, pl. 8 fig. 6 (lectotype designation).

####### Type locality.

”provincia Chiquitensi (republica Boliviana)”.

####### Label.

”S^n^ Juan Chiquitos Bolivia”, in d’Orbigny’s handwriting.

####### Dimensions.

”Latit. 19 millim.; longit. 9 millim.”; figured specimen herein H 19.3, D 9.57, W 6.3.

####### Type material.

NHMUK 1854.12.4.166, seven paralectotypes (d’Orbigny coll.).

####### Remarks.

d’Orbigny (1835) did not state on how many specimens his description was based. In d’Orbigny 1838 [1834–1847]: 274 he specified the locality as ”non loin des ruines de l’ancienne Mission de San-Juan”; see Breure 1973: 127, fig. 5. Breure (1975b) found three specimens in the MNHN collection, labelled ”Chiquitos”, of which he selected one as lectotype; this specimen is larger than d’Orbigny’s measurements. One of the specimens in lot 1854.12.4.166 corresponds to d’Orbigny 1838 [1834–1847]: pl. 33 figs 12–13 and is here re-figured. The name *Bulimus turritellatus* Beck, 1838 was an emendation of d’Orbigny’s name.

####### Current systematic position.

Bulimulidae, *Bulimulus turritellatus* (Beck, 1838).

###### Bulinus turritus

Broderip in Broderip and Sowerby I 1832

http://species-id.net/wiki/Bulinus_turritus

Figs 10D , L62vi

Bulinus turritus Broderip in Broderip and Sowerby I 1832b: 106; Sowerby I 1833 [Sowerby I and II 1832–1841]: fig. 31.
Bulimus turritus ; Reeve 1848 [1848–1850]: pl. 20 fig. 124.
Bulimulus (Geopyrgus) turritus ; Pilsbry 1896 [1895–1896]: 135, pl. 45 fig. 5.

####### Type locality.

”in Peruviae montibus (Truxillo)”.

####### Label.

”Peru”. M.C. label style I.

####### Dimensions.

”long. 11/12, lat. 4/12 poll. [H 23.2, D 8.4 mm]”; figured specimen herein H 25.3, D 8.59, W 10.5.

####### Type material.

NHMUK 20100616, five possible syntypes (Cuming coll.).

####### Remarks.

Broderip described this taxon from Cuming’s collection; it is likely that Reeve also used the same material in his work. One of the specimens corresponds to Reeve’s figure and the material is herein considered as possible syntypes.

####### Current systematic position.

Bulimulidae, *Bostryx turritus* (Broderip in Broderip and Sowerby I 1832).

###### Bulimus umbilicaris

Souleyet, 1842

http://species-id.net/wiki/Bulimus_umbilicaris

Figs 1A , L63i

Bulimus umbilicaris Souleyet 1842: 102; Souleyet 1852: 513, pl. 29 figs 12–15.
Bostryx (Ataxus) umbilicaris ; Breure 1975b: 1140, pl. 10 fig. 5 (lectotype designation).

####### Type locality.

”Les environs de Cobija, en Bolivie”.

####### Label.

”de Cobija au Bolivia”, taxon label in Souleyet’s handwriting.

####### Dimensions.

”Long. 13, diam. 7 mill.”; figured specimen herein H 12.8, D 7.2, W 6.8.

####### Type material.

NHMUK 1854.7.24.359, five paralectotypes (‘Voyage of the Bonité’ coll.).

####### Remarks.

Souleyet (1842) did not state on how many specimens his description was based. He wrote ”Ces espèces seront décrites avec plus de détails et avec figures dans la Zoologie du voyage de la *Bonite*” (i.e. Souleyet 1852). The collection of this trip is partly present in the NHMUK (ex Parzudaki ex Eydoux, Souleyet and Gaudichaud), partly in the MNHN. Breure (1975b) designated one of the specimens in the latter collection as lectotype, despite the fact that the locality and measurements did not exactly match the original data, contrary to the specimen figured herein.

####### Current systematic position.

Bulimulidae, *Bostryx umbilicaris* (Souleyet, 1842).

###### Bulimus umbricatus

Reeve, 1849

http://species-id.net/wiki/Bulimus_umbricatus

Figs 19G , L63ii

Bulimus umbricatus Reeve 1849 [1848–1850]: pl. 77 fig. 559; Breure 1979: 124 (lectotype designation).
Bulimulus umbraticus ; Pilsbry 1897 [1897–1898]: 52, pl. 10 fig. 88.
Drymaeus (Mesembrinus) umbraticus ; Breure and Eskens 1981: 89.

####### Type locality.

”Central America”.

####### Label.

”Central America”. M.C. label style IV.

####### Dimensions.

Not given; figured specimen herein H 15.3, D 8.0, W 6.0.

####### Type material.

NHMUK 1975184, lectotype (Cuming coll.).

####### Remarks.

The classification of this taxon by Thompson (2011: 107) with *Bulinus unicolor* Sowerby I, 1833 was incorrect, as shown by the typical *Drymaeus* protoconch sculpture of the lectotype. The specimen is subadult and the locality is very inprecise, making it difficult to match the material with any known taxon. The current systematic position tentively follows Breure and Eskens (1981).

####### Current systematic position.

Bulimulidae, *Drymaeus (Mesembrinus) umbraticus* (Reeve, 1849).

###### Bulinus unifasciatus

Sowerby I, 1833

http://species-id.net/wiki/Bulinus_unifasciatus

Figs 13A , L63iii

Bulinus unifasciatus Sowerby I 1833a: 37; Sowerby I 1833 [Sowerby I and II 1832–1841]: fig. 55; Breure 1979: 72.
Bulimulus unifasciatus ; Pilsbry 1897 [1897–1898]: 117, pl. 23 fig. 27.
Naesiotus unifasciatus ; Breure and Coppois 1978: 188 (lectotype designation); Breure and Borrero 2008: 17.

####### Type locality.

[Ecuador] ”ad Insulas Gallapagos”.

####### Label.

”Charles I^d^ / Galapagos”, in E.A. Smith’s handwriting.

####### Dimensions.

”long. 0.8, lat. 0.45 poll. [H 20.3, D 11.4 mm]”; figured specimen herein H 20.4, D 11.1, W 6.3.

####### Type material.

NHMUK 1975187, lectotype; 1975188, one paralectotype (Cuming coll.).

####### Remarks.

Sowerby described this taxon from the collection of H. Cuming; he specified ”Charles’s Island”, currently called Isla San Cristóbal. E.A. Smith, as curator of the collection, considered this probable type material; its status is here not disputed. The paralectotype (H 22.0, D 11.06, W 6.7) is better preserved and shows the characteristic pale band along the periphery of the last whorl.

####### Current systematic position.

Bulimulidae, *Naesiotus unifasciatus* (Sowerby I, 1833).

###### Bulinus ustulatus

Sowerby I, 1833

http://species-id.net/wiki/Bulinus_ustulatus

Figs 14E , L63iv

Bulinus ustulatus Sowerby I 1833b: 72; Sowerby I 1833 [Sowerby I and II 1832–1841]: fig. 42; Reeve 1848 [1848–1850]: pl. 21 fig. 130; Neubert and Janssen 2004: 233, pl. 11 fig. 134.
Naesiotus ustulatus ; Köhler 2007: 136, fig. 56; Breure and Borrero 2008: 17.

####### Type locality.

[Ecuador] ”ad Insulas Gallapagos”.

####### Label.

”Charles I^d^ / Galapagos”, in E.A. Smith’s handwriting. M.C. label style III, V.

####### Dimensions.

”long. 0.6, lat. 0.3 poll. [H 15.2, D 7.6 mm]”; figured specimen herein H 13.95, D 7.24, W 7.0.

####### Type material.

NHMUK 20100633, lectotype and one paralectotype (Cuming coll.).

####### Remarks.

Sowerby described this taxon from the collection of H. Cuming; in his remarks he specified ”Charles’s”, nowadays called Isla San Cristóbal. The specimen illustrated by Sowerby 1833 [Sowerby I and II 1832–1841] is now designated lectotype (**design.n.**) to define the taxon. The paralectotype was figured by Reeve.

####### Current systematic position.

Bulimulidae, *Naesiotus ustulatus* (Sowerby I, 1833).

###### Bulimus varicosus

Pfeiffer, 1853

http://species-id.net/wiki/Bulimus_varicosus

Figs 25H , L63v

Bulimus varicosus Pfeiffer 1853b: 256; Pfeiffer 1854 in Küster and Pfeiffer 1840–1865: 83, pl. 30 figs 7–8; Breure 1979: 115 (lectotype designation).

####### Type locality.

”in republicâ Mexicanâ”.

####### Label.

”Mexico”, taxon label in Pfeiffer’s handwriting. M.C. label style IV.

####### Dimensions.

”Long. 35, diam. 14 mill.”; figured specimen herein H 35.5, D 16.7, W 6.4.

####### Type material.

NHMUK 1975466, lectotype; 1975467, one paralectotype (Cuming coll.).

####### Remarks.

The current systematic position follows Thompson (2011: 114).

####### Current systematic position.

Bulimulidae, *Drymaeus (Mesembrinus) attenuatus varicosus* (Pfeiffer, 1853).

###### Bulimulus (Naesiotus) ventrosus

Reibisch, 1892

http://species-id.net/wiki/Bulimulus_ventrosus

Figs 14I , L63vi

Bulimulus (Naesiotus) ventrosus Reibisch 1892: 19, pl. 1 figs 12a–b.
Bulimulus ventrosus ; Pilsbry 1897 [1897–1898]: 109, pl. 22 figs 14–15.
Naesiotus ventrosus ; Köhler 2007: 138, fig. 57 (lectotype designation).

####### Type locality.

”Burrington-Island (Wolf)”.

####### Label.

”Barrington Is / Galapagos”.

####### Dimensions.

”Long. 17,5, diam. maj. 8.3 mm”; figured specimen herein H 18.9, D 7.93, W 7.2.

####### Type material.

NHMUK 1894.6.8.1, one paralectotype, T. Wolf leg. (ex Reibisch).

####### Remarks.

Reibisch reported three adult and one subadult shell to have seen for his description. The current systematic position is according to Richardson (1995).

####### Current systematic position.

Bulimulidae, *Naesiotus ventrosus* (Reibisch, 1892).

###### Bulimus verrucosus

Pfeiffer, 1855

http://species-id.net/wiki/Bulimus_verrucosus

Figs 14D , L64i

Bulimus verrucosus Pfeiffer 1855f: 116; Pfeiffer 1859b: 475; Breure 1979: 74.
Naesiotus nux ; Breure and Coppois 1978: 178 (lectotype designation); Breure and Borrero 2008: 14.

####### Type locality.

[Ecuador] ”Galapagos Islands”.

####### Label.

”Gallapagos Islds”, taxon label in Pfeiffer’s handwriting. M.C. label style III.

####### Dimensions.

”Long. 23, diam. 11 1/2 mill.”; figured specimen herein H 22.5, D 12.4, W 7.6.

####### Type material.

NHMUK 1975168, lectotype; 1975169, two paralectotypes (Cuming coll.).

####### Remarks.

Pfeiffer did not state on how many specimens his description was based.

####### Current systematic position.

Bulimulidae, *Naesiotus nux* (Broderip, 1832).

###### Bulinus versicolor

Broderip in Broderip and Sowerby I 1832

http://species-id.net/wiki/Bulinus_versicolor

Figs 66C–D , L64iii

Bulinus versicolor Broderip in Broderip and Sowerby I 1832b: 108; Sowerby I 1833 [Sowerby I and II 1832–1841]: fig. 16–16*.
Bulimus versicolor ; Reeve 1848 [1848–1850]: pl. 19 fig. 113.

####### Type locality.

”in montibus Peruviae (Mongon, near Casma)”.

####### Label.

”Peru”. M.C. label style I.

####### Dimensions.

”long. 1 1/8, lat. 5/8 poll. [H 28.5, D 15.8 mm]”; figured specimen herein H 29.6, D 17.7, W 6.2.

####### Type material.

NHMUK 1842.5.10.180–182, four possible syntypes; 20100637, four possible syntypes (Cuming coll.).

####### Remarks.

Broderip described this taxon from Cuming’s collection; he did not state on how many specimens his description was based. Reeve’s figure was also based on material from the same collection. One of the specimens in lot 20100637 corresponds to Reeve’s figure.

####### Current systematic position.

Bulimulidae, *Scutalus versicolor versicolor* (Broderip in Broderip and Sowerby I 1832).

###### Bulimus veruculum

Morelet, 1860

http://species-id.net/wiki/Bulimus_veruculum

Figs 3A , L64ii

Bulimus veruculum Morelet 1860: 376; Morelet 1863: 211, pl. 11 fig. 11.
Bulimulus (Geoceras) veruculum ; Pilsbry 1896 [1895–1896]: 137, pl. 45 fig. 8.

####### Type locality.

[Peru] ”[intimâ Peruviii regionae]”; see remarks.

####### Label.

”Pérou, Ayacucho”, in Morelet’s handwriting.

####### Dimensions.

”Long. 24; diam. 4 1/2 millim.”; figured specimen herein H 24.5, D 4.2, W 19.1.

####### Type material.

NHMUK 1893.2.4.1141–1145, five syntypes (Morelet coll.).

####### Remarks.

Morelet (1860) did not state on how many specimens his description was based. In Morelet (1863) the locality is specified as ”Balsa de Cocharcas”, which might be Dept. Ayacucho, Cocharcas. The current systematic position follows Richardson (1995: 50).

####### Current systematic position.

Bulimulidae, *Bostryx veruculum* (Morelet, 1860).

###### Bulimus vesicalis

Pfeiffer, 1853

http://species-id.net/wiki/Bulimus_vesicalis

Figs 64I–J , L64iv

Bulimus vesicalis Pfeiffer 1853c: 58; Pfeiffer 1853d: 654; Pfeiffer 1855 in Küster and Pfeiffer 1840–1865: pl. 70 figs 23–24; Breure 1979: 64.
Bulimulus vesicalis ; Pilsbry 1897 [1897–1898]: 69, pl. 12 figs 41–42; Breure 1978: 149 (lectotype designation); Simone 2006: 121, fig. 375; Cuezzo et al. 2013: 153.

####### Type locality.

”in Brasilia (Cuming)”.

####### Label.

”Brazils”, taxon label in Pfeiffer’s handwriting. M.C. label style IV.

####### Dimensions.

”Long. 25, diam. 13 mill.”; figured specimen herein H 24.8, D 12.7, W 6.2.

####### Type material.

NHMUK 1975395, lectotype; 1975396, two paralectotypes (Cuming coll.).

####### Remarks.

The current systematic position follows Simone (2006).

####### Current systematic position.

Bulimulidae, *Bulimulus vesicalis* (Pfeiffer, 1853)

###### Bulimus vespertinus

Pfeiffer, 1858

http://species-id.net/wiki/Bulimus_vespertinus

Figs 44G–I , L64v

Bulimus vespertinus Pfeiffer 1858a: 257, pl. 42 fig. 3; Pfeiffer 1868: 109; Pfeiffer 1869 [1866–1869]: 465, pl. 101 figs 16–19; Breure 1979: 115 (lectotype designation).
Drymaeus vespertinus ; Pilsbry 1898 [1897–1898]: 269, pl. 47 figs 8–10.
Drymaeus (Drymaeus) vespertinus ; Breure and Eskens 1981: 4, pl. 6 fig. 6.

####### Type locality.

”Province of Patas, Andes of Peru (Dr. Farris)”.

####### Label.

”Province of Patas, / Andes of Peru / D^r^. Farris”, taxon label in Pfeiffer’s handwriting. M.C. label style IV.

####### Dimensions.

”Long. 36, diam. 14 mill.”; figured specimen herein H 34.6, D 14.8, W 6.3.

####### Type material.

NHMUK 1975471, lectotype; 1975472, two paralectotypes (Cuming coll.).

####### Remarks.

The current systematic position follows Richardson (1995: 193).

####### Current systematic position.

Bulimulidae, *Drymaeus (Drymaeus) vespertinus* (Pfeiffer, 1858).

###### Bostryx (Bostryx) vilchezi

Weyrauch, 1960

http://species-id.net/wiki/Bostryx_vilchezi

Fig. 7C

Bostryx (Bostryx) vilchezi Weyrauch 1960a: 32, pl. 3 figs 8–10; Breure 1979: 59; Neubert and Janssen 2004: 234, pl. 4 fig. 41.

####### Type locality.

”Interandines N-Peru: Sócota, 20 km nö. Cutervo (1950 m), im Tale des Río Guineamaya, im Becken des Marañon”.

####### Label.

”N-Peru, Socota, 20 kms northeast of Cutervo, 1950m”; printed label.

####### Dimensions.

”H 8.4 D 4.5”; figured specimen herein H 8.2, D 4.7, W 6.5.

####### Type material.

NHMUK 1975468, five paratypes (ex Weyrauch).

####### Current systematic position.

Bulimulidae, *Bostryx vilchezi* Weyrauch, 1960.

###### Bulimus vincentinus

Pfeiffer, 1846

http://species-id.net/wiki/Bulimus_vincentinus

Figs 21D , L64vi

Bulimus vincentinus Pfeiffer 1846a: 30; Pfeiffer 1848b: 103; Reeve 1848 [1848–1850]: pl. 55 fig. 366b; Breure 1979: 125 (lectotype designation).
Drymaeus (Mesembrinus) vincentinus ; Köhler 2007: 154, fig. 138.

####### Type locality.

”Island of St. Vincent (Rev. L. Guilding)”.

####### Label.

”St. Vincent W.I.”. M.C. label style III.

####### Dimensions.

”Long. 30, diam. 11 1/2 mill.”; figured specimen herein H 29.9, D 12.4, W 6.5.

####### Type material.

NHMUK 1975219, lectotype; 1975220, two paralectotypes (Cuming coll.).

####### Remarks.

Pfeiffer described this species from H. Cuming’s collection, but did not state on how many specimens his description was based. The variety he described from Venezuela is not considered to be this species.

####### Current systematic position.

Bulimulidae, *Drymaeus (Mesembrinus) vincentinus* (Pfeiffer, 1846).

###### Bulimus virginalis

Pfeiffer, 1856

http://species-id.net/wiki/Bulimus_virginalis

Figs 24C , L65i

Bulimus virginalis Pfeiffer 1856b: 46; Pfeiffer 1859b: 405.

####### Type locality.

[Venezuela] ”prope Caracas”.

####### Label.

”Venezuela”, taxon label in Pfeiffer’s handwriting. M.C. label style IV.

####### Dimensions.

”Long. 26, diam. 91/2 mill.”; figured specimen herein H 27.9, D 11.5, W 7.2.

####### Type material.

NHMUK 1975503, five probable syntypes (Cuming coll.).

####### Remarks.

Pfeiffer described this taxon based on material ”teste E. Klocke”, and did not state on how many specimens his description was based. The material in the Cuming collection has a taxon label in Pfeiffer’s handwriting, and is considered as probable syntypes.

####### Current systematic position.

Bulimulidae, *Drymaeus (Mesembrinus) virginalis* (Pfeiffer, 1856).

###### Bulimus virgultorum

Morelet, 1863

http://species-id.net/wiki/Bulimus_virgultorum

Figs 10E , L65ii

Bulimus virgultorum Morelet 1863: 194, pl. 10 fig. 1; Breure 1979: 59.
Bulimulus (Lissoacme) virgultorum ; Pilsbry 1896 [1895–1896]: 168, pl. 10 fig. 1.
Bostryx virgultorum ; Breure 1978: 139 (lectotype designation).

####### Type locality.

”les vallées chaudes du versant oriental de la Cordillère, notammant celle de Santa-Anna”.

####### Label.

”Pérou”, in Morelet’s handwriting.

####### Dimensions.

”Long. 31; diam. 14 mill.”; figured specimen herein H 27.8, D 16.0, W 6+.

####### Type material.

NHMUK 1893.2.4.179–181, three paralectotypes; 1893.2.4.213–218, six paralectotypes (Morelet coll.).

####### Remarks.

The classification of Richardson (1995: 237) as *Naesiotus virgulatorus* [sic] is erroneous; this taxon belongs to *Bostryx* *s. l.*.; see also Breure 2012b.

####### Current systematic position.

Bulimulidae, *Bostryx virgultorum* (Morelet, 1863).

###### Bulimus voithianus

Pfeiffer, 1847

http://species-id.net/wiki/Bulimus_voithianus

Figs 10A–B , L65v

Bulimus voithianus Pfeiffer 1847: 114; Pfeiffer 1848b: 210;
Bulimus meridionalis Reeve 1848 [1848–1850]: pl. 21 fig. 131.

####### Type locality.

”Chili (T. Bridges)”.

####### Label.

”Chili”. M.C. label style I.

####### Dimensions.

”Long. 19, diam. 7 1/2 mill.”; figured specimen herein H 19.1, D 8.1, W 6.2.

####### Type material.

NHMUK 20100565.1–4, lectotype and three paralectotypes (Cuming coll.).

####### Remarks.

Pfeiffer described this species from H. Cuming’s collection, but did not state on how many specimens his description was based. Reeve described the same material (”Chili; Bridges”) as *Bulimus meridionalis* [June 1848, not *Bulimus meridionalis* Reeve Dec. 1848 (pl. 56 fig. 370)]. In the index (Reeve 1850 [1848–1850]: ix) he corrected this to ”Voithianus, *Pfr*.”, apparently having realized that Pfeiffer’s taxon was the same; this has escaped the notice of later authors. Thus *Bulimus meridionalis* Reeve, 1848 [June] is a junior synonym of *Bulimus voithianus* Pfeiffer, 1847 (**syn. n.**). One of the specimens found corresponds with Pfeiffer’s shell height and is here selected lectotype (**design. n.**) to define the taxon. The current systematic position follows Richardson (1995: 50).

####### Current systematic position.

Bulimulidae, *Bostryx voithianus* (Pfeiffer, 1847).

###### Drymaeus volsus

Fulton, 1907

http://species-id.net/wiki/Drymaeus_volsus

Figs 35A–C , L64vii

Drymaeus volsus Fulton 1907: 153, pl. 10 fig. 2; Breure 1979: 115.
Drymaeus (Drymaeus) volsus ; Breure and Borrero 2008: 23.

####### Type locality.

”Ecuador”.

####### Label.

”Ecuador”, in Fulton’s handwriting.

####### Dimensions.

”Maj. diam. 12 1/2, alt. 30 1/2 mm.”; figured specimen herein H 30.3, D 12.7, W 6.3.

####### Type material.

NHMUK 1907.5.3.162, lectotype (ex Fulton).

####### Remarks.

Fulton did not state on how many specimens his description was based. The reference of Breure (1979) to ”HT BMNH 1907.5.3.162” has to be interpreted as a lectotype designation under Art. 74.6 ICZN.

####### Current systematic position.

Bulimulidae, *Drymaeus (Drymaeus) volsus* Fulton, 1907.

###### Drymaeus wintlei

Finch, 1929

http://species-id.net/wiki/Drymaeus_wintlei

Figs 21H , L65iii

Drymaeus wintlei Finch 1929: 275, figs; Breure 1979: 125.
Drymaeus (Mesembrinus) wintlei ; Breure and Borrero 2008: 24.

####### Type locality.

”Ecuador”.

####### Label.

”Ecuador”.

####### Dimensions.

”alt. 21.5, diam. 9 mm.”; figured specimen herein H 21.7, D 10.1, W 5.8.

####### Type material.

NHMUK 1929.6.11.1, lectotype (ex Finch).

####### Remarks.

Finch remarked ”a large series (...) was collected by the late Mr. Clarence Buckley”. Therefore, the reference of Breure (1979) to ”HT BMNH 1929.6.11.1” has to be interpreted as a lectotype designation under Art. 74.6 ICZN.

####### Current systematic position.

Bulimulidae, *Drymaeus (Drymaeus) wintlei* Finch, 1929 (**comb. n.**).

###### Bulimulus (Naesiotus) wolfi

Reibisch, 1892

http://species-id.net/wiki/Bulimulus_wolfi

Figs 12G , L65iv

Bulimulus (Naesiotus) wolfi Reibisch 1892: 22, pl. 2 figs 1a–b; Breure 1979: 72.
Bulimulus wolfi ; Pilsbry 1897 [1897–1898]: 115, pl. 23 figs 22–23.
Naesiotus wolfi ; Köhler 2007: 140, fig. 59 (lectotype designation); Breure and Borrero 2008: 18.

####### Type locality.

[Ecuador, Galápagos] ”Indefatigale-Island (Wolf)”.

####### Label.

”Indefatigale I^s^ / Galapagos”.

####### Dimensions.

”Long. 13.25–14, diam. maj. abunde 8”; figured specimen herein H 12.19, D 7.97, W 6.7.

####### Type material.

NHMUK 1894.6.8.7, paralectotype, T. Wolf leg. (ex Reibisch).

####### Remarks.

Reibisch described this species on the basis of two adult and one subadult shell. Richardson (1995: 230) suppressed Reibisch’s name on account of the supposed existence of *Bulimulus wolfi* Miller, 1878. In Miller (1878) no such name exists, and therefore Reibisch’s name is valid.

####### Current systematic position.

Bulimulidae, *Naesiotus wolfi* (Reibisch, 1892).

###### Bulimus woodwardi

Pfeiffer, 1857

http://species-id.net/wiki/Bulimus_woodwardi

Figs 10F , L66i

Bulimus woodwardi Pfeiffer 1857c: 332; Pfeiffer 1859b: 488; Breure 1979: 136 (lectotype designation).

####### Type locality.

”Andes of Peru”.

####### Label.

”Andes of Peru”, taxon label in Pfeiffer’s handwriting. M.C. label style IV.

####### Dimensions.

”Long. 31, diam. 13 1/2 mill.”; figured specimen herein H 31.0, D 13.7, W 8.1.

####### Type material.

NHMUK 1975334, lectotype (Cuming coll.).

####### Remarks.

Pfeiffer described this taxon from the Cuming collection, but did not state on how many specimens his description was based. The protoconch is sculptured with indistinct granules, the shell shape is like those found in several taxa of *Bostryx* sensu Breure 1979, who treated this taxon as a nomen inquirendum. The current systematic position follows Richardson (1995: 51).

####### Current systematic position.

Bulimulidae, *Bostryx woodwardi* (Pfeiffer, 1857).

###### Helix xanthostoma

d’Orbigny, 1835

http://species-id.net/wiki/Helix_xanthostoma

Figs 27J–L , L65iii

Helix xanthostoma d’Orbigny 1835: 18.
Bulimus xanthostomus d’Orbigny 1837 [1834–1847]: 312, pl. 40 figs 1–2 [18 Sept. 1837; text 6 May 1838]; Gray 1854: 21.
Drymaeus xanthostomus ; Pilsbry 1898 [1897–1898]: 196, pl. 36 figs 52–53; Breure 1975b: 1153, pl. 2 fig. 6 (lectotype designation).

####### Type locality.

”provincia Yungacensi (republica Boliviana)”.

####### Label.

”Carcuata, Yungas, Bolivia”, in d’Orbigny’s handwriting.

####### Dimensions.

”Longit. 46 millim., latit. 17 millim.”; figured specimen herein H 43.8, D 19.0, W 6.8.

####### Type material.

NHMUK 1854.12.4.221, seven paralectotypes (d’Orbigny coll.).

####### Remarks.

d’Orbigny (1835) did not state on how many specimens his description was based. In d’Orbigny 1838 [1834–1847]: 312 he specified the locality as ”seulement sur le coteau qui sépare les villages de Circuata et de Carcuata”; see Breure 1973: 133, fig. 7. Breure (1975b) found five specimens in the MNHN collection, labelled ”Carcuata Yas”, of which he selected one as lectotype. One of the specimens in lot 1854.12.4.221 corresponds to d’Orbigny 1837 [1834–1847]: pl. 40 figs 1–2; one of the other specimens is a fragment only.

####### Current systematic position.

Bulimulidae, *Drymaeus (Drymaeus) xanthostomus* (d’Orbigny, 1835).

###### Bulimus yungasensis

d’Orbigny, 1837

http://species-id.net/wiki/Bulimus_yungasensis

Figs 35D–F , L65iv

Bulimus yungasensis d’Orbigny 1837 [1834–1847]: 316, pl. 40 fig. 8 [18 Sept. 1837; text 6 May 1838]; Gray 1854: 21.
Drymaeus yungasensis ; Pilsbry 1898 [1897–1898]: 196, pl. 36 figs 52–53; Breure 1975b: 1153, pl. 2 fig. 6 (lectotype designation).

####### Type locality.

[Bolovia] ”seulement dans les bois qui bordent le Rio de Meguilla, près de son confluent avec le Rio de la Paz”.

####### Label.

”megu[illa], Yungas Bolivia”, in d’Orbigny’s handwriting.

####### Dimensions.

”Long. 35 millim.; lat. 13 millim.”; figured specimen herein H 32.6, D 13.54, W 6.4.

####### Type material.

NHMUK 1854.12.4.134, lectotype and four paralectotypes (d’Orbigny coll.).

####### Remarks.

d’Orbigny did not state on how many specimens his description was based. One of the specimens corresponds to his figure and is here designated lectotype (**design.n.**) to define the taxon.

####### Current systematic position.

Bulimulidae, *Drymaeus (Drymaeus) yungasensis* (d’Orbigny, 1837).

###### Bulimus zhorquinensis

Angas, 1879

http://species-id.net/wiki/Bulimus_zhorquinensis

Figs 40G–I , L66ii

Bulimus zhorquinensis Angas 1879: 478, pl. 40 fig. 4; Breure 1979: 115.
Drymaeus zhorquinensis ; Pilsbry 1899: 31, pl. 26 figs 14–15.

####### Type locality.

[Costa Rica] ”Middle Zhorquia to Cuabre”.

####### Label.

”Middle Zhorquia to / Cuabre (low hills + / flat ground) / Costa Rica”.

####### Dimensions.

”Diam. 12, alt. 23 lin. [H 48.5, D 25.3 mm]”; figured specimen herein H 46.1, D 24.4, W 7.2.

####### Type material.

NHMUK 1879.7.22.15, lectotype, W.M. Gabb leg. (ex Angas).

####### Remarks.

Angas wrote ”only three specimens were obtained”; thus the reference of Breure (1979) to ”HT BMNH 1879.7.22.15” has to be interpreted as a lectotype designation under Art. 74.6 ICZN. The whereabouts of the other two specimens mentioned by Angas are unknown. The current systematic position follows Thompson (2011: 109).

####### Current systematic position.

Bulimulidae, *Drymaeus (Drymaeus) zhorquinensis* (Angas, 1879).

###### Bulimulus (Drymaeus) ziczac

da Costa, 1898

http://species-id.net/wiki/Bulimulus_ziczac

Figs 30J–L , L66v

Bulimulus (Drymaeus) ziczac da Costa 1898: 81, pl. 6 fig. 5; Breure 1979: 115.
Drymaeus ziczac ; Pilsbry 1898 [1897–1898]: 212, pl. 50 fig. 92; Linares and Vera 2012: 192.

####### Type locality.

”Valley of the R. Cauca, Colombia”.

####### Label.

”Llanos de Cavariare / Colombia”. in da Costa’s handwriting.

####### Dimensions.

”Long. 28, diam. 15 mm.”; figured specimen herein H 26.5, D 15.0, W 4+.

####### Type material.

NHMUK 1907.11.21.46, lectotype;. 47, one paralectotype (da Costa coll.).

####### Remarks.

da Costa did not state on how many specimens his description was based; the reference of Breure (1979) to ”HT BMNH 1907.11.21.46” has to be interpreted as a lectotype designation under Art. 74.6 ICZN. The top of the lectotype is damaged, hence the shell height appears smaller than mentioned by da Costa. The name ”Cavariare” could not be found using modern gazetteers.

####### Current systematic position.

Bulimulidae, *Drymaeus (Drymaeus) ziczac* (da Costa, 1898).

###### Bulimus ziegleri

Pfeiffer, 1847

http://species-id.net/wiki/Bulimus_ziegleri

Bulimus ziegleri Pfeiffer 1847: 113; Reeve 1849 [1848–1850]: pl. 58 fig. 389.

####### Type locality.

”Locality unknown”.

####### Label.

”Brazils” [1975570], ”[C]hiapes [Chiapas] Mexico” [20120338].

####### Dimensions.

”Long. 21, diam. 10 mill.”.

####### Material.

 NHMUK 1975570, two specimens; 20120338, four specimens (Cuming coll.).

####### Remarks.

Pfeiffer described this taxon from the collection of H. Cuming, but did not state on how many specimens his description was based; he also did describe a variety and stated that the locality was unknown. In Pfeiffer (1848b: 175) he stated ”Habitat in America centrali (Largilliert) et in republica mexicana? (Liebmann.)”; the depository of these specimens was not given. Two lots have been found which have taxon labels in Pfeiffer’s handwriting indicating ”*ziegleri* Pfr.”. Lot 1975570 has a label mentioning ”Brazils”, on which is added in pencil ”var β? Pfr. Mon Hel. II / p. 175”. Lot 20120338 has two taxon labels in Pfeiffer’s hand and one of them has ”[C]hiapes [Chiapas] Mexico / Mon Geisbright”; this is the lot that has been used by Reeve and was referred by him to ”Central America”. Pfeiffer (1853d: 413) refers to this figure as being [*Bulimus*] *ziegleri*, without giving further data. Hupé (1857: 51) associated this species with Peruvian material without further data or illustration. Tryon (1867: 168, pl. 13 fig. 6) mentioned this species from ”Cinalos, North-western Mexico”. Binney and Bland (1869: 193, fig. 336) gave as locality ”Mazatlan and Central America”, and their figure was ”drawn from a specimen received from Dr. Pfeiffer”. Pfeiffer (1872: 76), reporting on shells collected in Suriname by Kühn, mentioned ”aus der Umgegend von Paramaribo: (...) *Bulimus Ziegleri Pfr.*”; this was likely a misidentification, but may have led to the confusion mentioned below. Stearns (1894: 167) reported three living specimens of this species intercepted at San Francisco, and originating in Altata [Mexico, Edo. Sinaloa]. Pilsbry (1899: 40)—who seemed to be convinced of the northwestern Mexican origin of this taxon—suggested that Pfeiffer’s original description was based on immature specimens. He further remarked ”Probably it [Pfeiffer’s variety] has nothing to do with the typical *ziegleri*.”; he considered *Bulimus californicus* Reeve 1848 ([1848–1850]: pl. 56 fig. 378) as a junior subjective synonym of Pfeiffer’s taxon. Vernhout (1914: 14–15) refers to Pfeiffer (1872) and said ”as Pfeiffer himself records this species from Paramaribo, I feel not justified to doubt of its occurrence in Surinam.” Simone (2006: 146, fig. 490) figured specimens from lot 1975570 as ”possible syntypes” from Brazil, as *Mesembrinus ziegleri*, without expressing doubts about its occurrence. Finally, Thompson (2011: 111) records this species from Mexico, Edo. Sinaloa.

From the data given above, we may safely conclude that Pfeiffer’s taxon—though described from an unknown locality—occurs in northwestern Mexico. The original material may have eighter been lost, or the label ”Brazils” was mistrusted by Pfeiffer as he may have seen material from other sources (see Pfeiffer 1848b: 175); it may be noted that one of the specimens from lot 1975570 corresponds to the shell height given by him. The lot from Chiapas [20120338] is not conspecific with Pfeiffer’s taxon. Both lots are not considered herein as type material.

###### Helix zoographica

d’Orbigny, 1835

http://species-id.net/wiki/Helix_zoographica

Figs 28D , L66vi

Helix zo[o]graphica d’Orbigny 1835: 19.
Bulimus zoographicus ; d’Orbigny 1837 [1834–1847]: 313, pl. 40 figs 6–7 [not 6–8] [18 Sept. 1837; text 6 May 1838]; Gray 1854: 22.
Drymaeus zoographicus ; Pilsbry 1898 [1897–1898]: 197, pl. 38 figs 6–7; Breure 1975b: 1153, pl. 1 fig. 6 (lectotype designation).

####### Type locality.

”provincia Yungacensi (republica Boliviana)”.

####### Label.

”Tutulima Bolivia”, in d’Orbigny’s handwriting.

####### Dimensions.

”Longit. 35 millim., latit. 13 millim.”; figured specimen herein H 31.7, D 13.72, W 6.1.

####### Type material.

NHMUK 1854.12.4.131, one paralectotype (d’Orbigny coll.).

####### Remarks.

d’Orbigny (1835) did not state on how many specimens his description was based. In d’Orbigny 1838 [1834–1847]: 314 he specified the locality as ”entre les provinces de Cochabamba et de Moxos, au fond d’un ravin dans lequel coule le Rio Altamachi, le premier affluent oriental du Rio Beni, un peu en avant de Tutulima”; see Breure 1973: 130. Breure (1975b) found five specimens in the MNHN collection, labelled ”Tutulima” and ”... Yungas”, of which he selected one as lectotype. The specimen in lot 1854.12.4.131 corresponds to d’Orbigny 1837 [1834–1847]: pl. 40 figs 6–7.

####### Current systematic position.

Bulimulidae, *Drymaeus (Drymaeus) zoographicus* (d’Orbigny, 1835).

#### Non-Bulimulidae

**Remarks.** The following taxon, hitherto classified with the Bulimulidae, proved not to belong this group.

*Bulimus inermis* Morelet, 1851 (: 10). Type locality. [Mexico, Edo. Campeche] ”circa vicum Palizada provinciae Yucatanensis”. Type material. NHMUK 1893.2.4.1134–1135, two syntypes, ex Morelet.

Remarks. The specimens are both glued on paper. The protoconch is smooth, the teleoconch is sculptured with axial riblets, the spaces 2–3 times the width of the riblets. This is likely a *Leptinaria* species (Subulinidae).

## Plates

**Figure 5.**
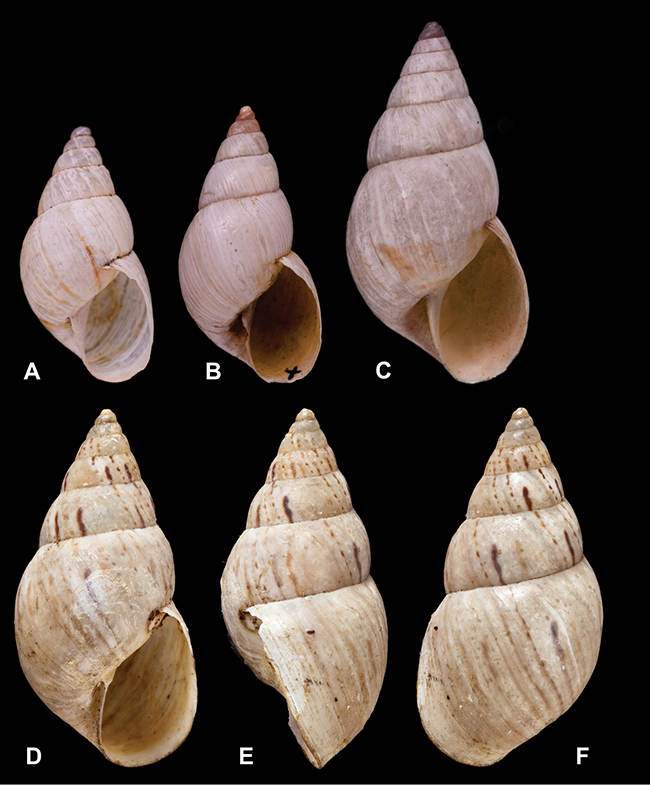
*Bostryx* species. **A** *Bostryx aileenae* Breure, 1978, paratype NHMUK 1975229 (H = 13.7) **B–C** *Bostryx nigropileatus* (Reeve, 1849) **B** lectotype of *Bulimus balsanus* Morelet, 1863, NHMUK 1893.2.4.173 (H = 17.9) **C** lectotype of *Bulimus nigropileatus* Reeve, 1849, NHMUK 1975335 (H = 20.5) **D–F** *Bostryx reconditus* (Reeve, 1849), lectotype NHMUK 1975189 (H = 19.8). All enlarged.

**Figure 6.**
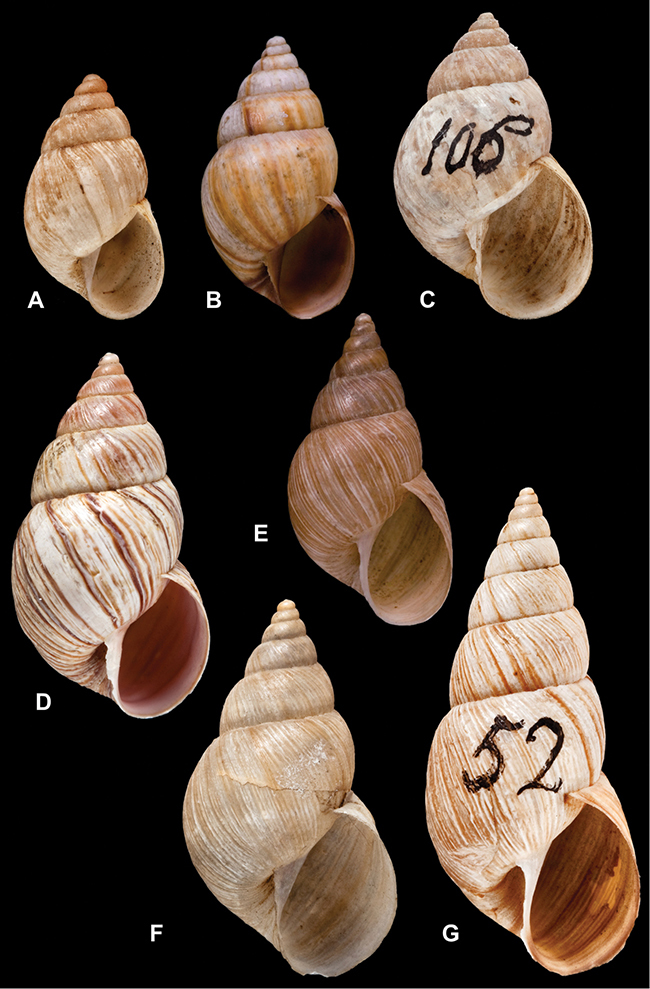
*Bostryx* species. **A** *Bostryx acalles* (Pfeiffer, 1853), possible syntype NHMUK 20100651 (H = 13.2) **B** *Bostryx depstus* (Reeve, 1849), lectotype NHMUK 1975318 (H = 18.5) **C** *Bostryx conspersus* (Sowerby I, 1833), probable syntype NHMUK 20100619 (H = 19.8) **D** *Bostryx ?hennahi* (J.E. Gray, 1830), lectotype of *Helix cactorum* d’Orbigny, 1835, NHMUK 1854.12.4.189 (H = 24.4) **E–G** *Bostryx modestus* (Broderip in Broderip and Sowerby I 1832) **E** Lectotype of *Bulimus limensis* Reeve, 1849, NHMUK 1975326 (H = 19.6) **F** syntype of *Bulimus philippii* Pfeiffer, 1842, NHMUK 1975348 (H = 24.3) **G** *Bulinus modestus* Broderip in Broderip and Sowerby I 1832, possible syntype NHMUK 20120232 (H = 31.6). All enlarged.

**Figure 7.**
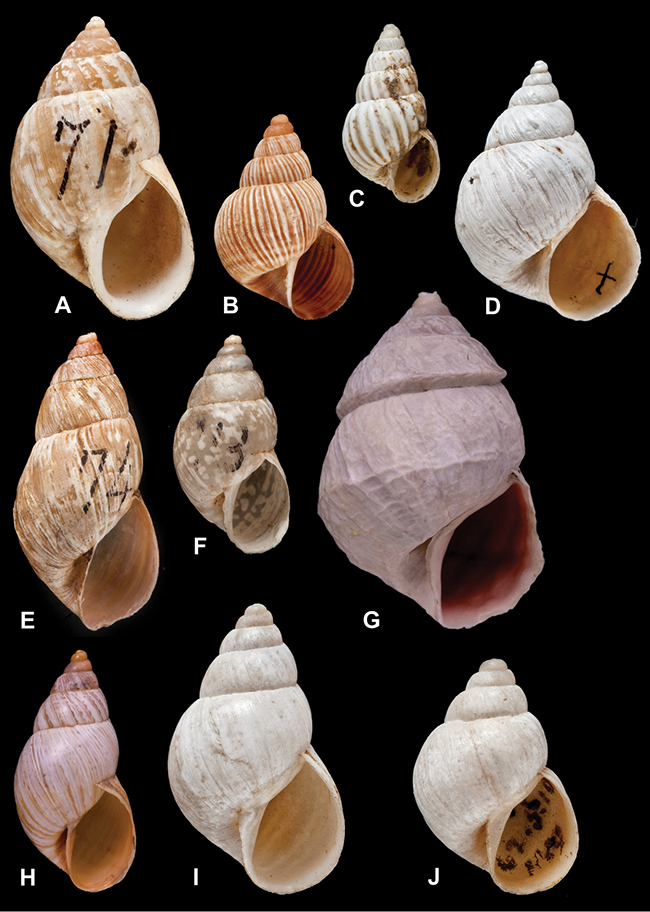
*Bostryx* species. **A** *Bostryx erosus* (Broderip in Broderip and Sowerby I 1832), syntype NHMUK 20100612 (H = 22.9) **B** *Bostryx scalariformis* (Broderip in Broderip and Sowerby I 1832), probable syntype NHMUK 20100635 (H = 11.6) **C** *Bostryx vilchezi* Weyrauch, 1960, paratype NHMUK 1975468 (H = 8.2) **D** *Bostryx rusticellus* (Morelet, 1860), lectotype NHMUK 1893.2.4.201 (H = 19.9) **E** *Bostryx guttatus* (Broderip in Broderip and Sowerby I 1832), possible syntype NHMUK 20110175 (H = 22.0) **F** *Bostryx pruinosus* (Sowerby I, 1833), syntype NHMUK 1975544 (H = 13.48) **G** *Bostryx reentsi* (Philippi, 1851), syntype of *Bulimus denickei* J.E. Gray, 1852, NHMUK 1851.5.15.1 (H = 26.4) **H** *Bostryx compactus* (Fulton, 1902), syntype NHMUK 1902.5.28.1 (H = 15.8) **I** *Bostryx albicans* (Broderip in Broderip and Sowerby I 1832), possible syntype NHMUK 20100611 (H = 22.1) **J** *Bostryx albus* (Sowerby I, 1833), possible syntype NHMUK 1842.5.10.149 (H = 15.0). All enlarged.

**Figure 8.**
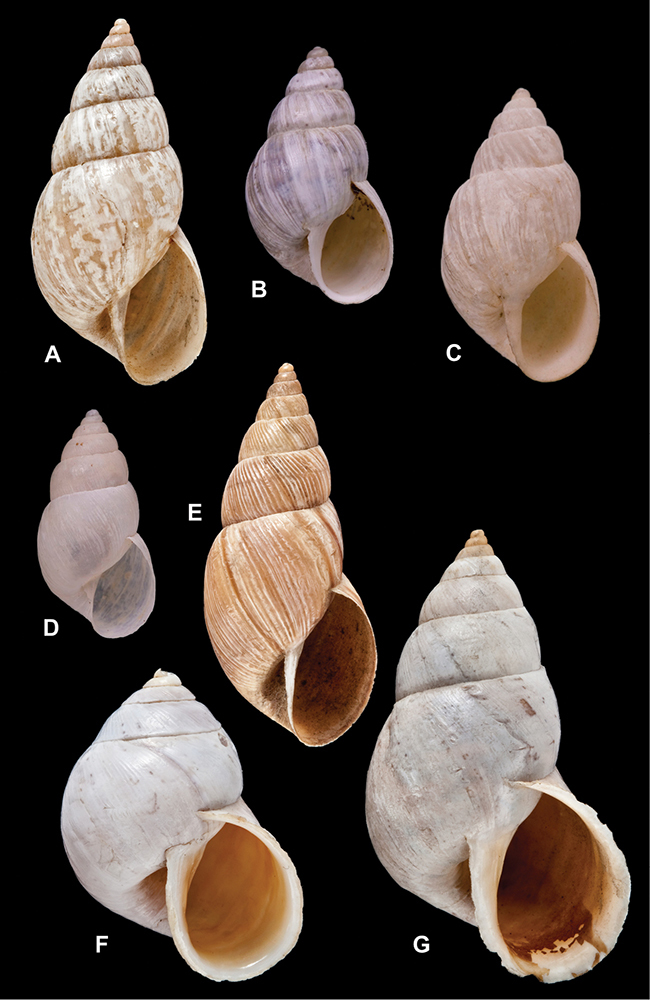
*Bostryx* species. **A** *Bostryx affinis* (Broderip in Broderip and Sowerby I 1832), possible syntype NHMUK 20100610 (H = 27.5) **B** *Bostryx huascensis* (Reeve, 1848), lectotype NHMUK 1975159 (H = 19.1) **C** *Bostryx mejillonensis* (Pfeiffer, 1857), lectotype NHMUK 1975322 (H = 24.1) **D** *Bostryx kathiae* Breure, 1978, paratype NHMUK 1975228 (H = 17.5) **E** *Bostryx obliquistriatus* (da Costa, 1901), lectotype NHMUK 1907.11.21.41 (H = 28.9) **E** *Bostryx rhololarynx papillatus* (Morelet, 1860), syntype NHMUK 1893.2.4.192 (H = 22.7) **G** *Bostryx rhololarynx rhodolarynx* (Reeve, 1849), lectotype NHMUK 1975434 (H = 34.5). All enlarged.

**Figure 9.**
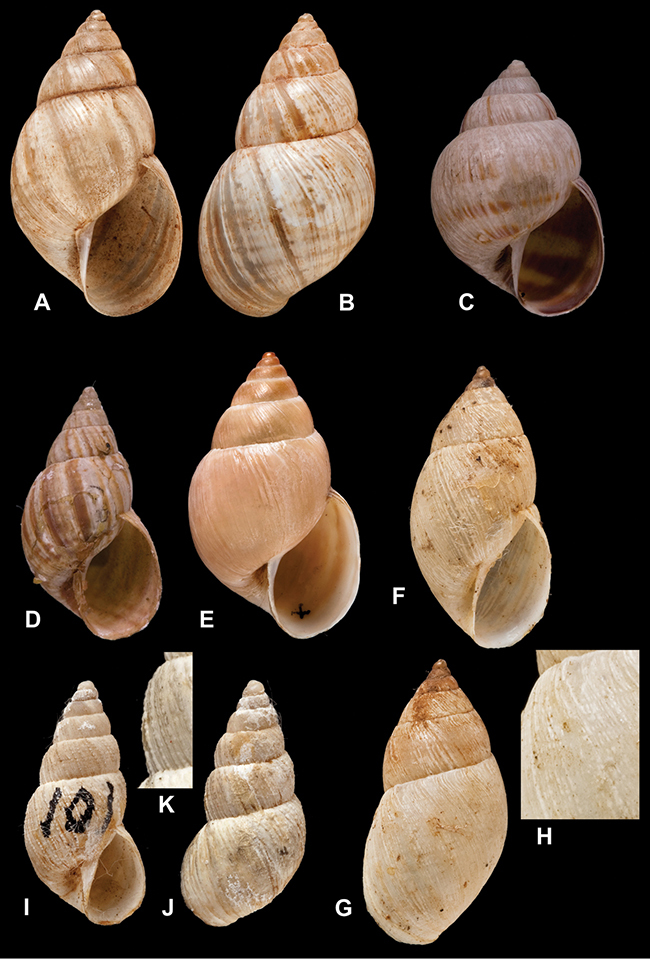
*Bostryx* species. **A–B** *Bostryx apodemetus* (d’Orbigny, 1835), syntype NHMUK 1854.12.4.182 (H = 23.0) **C** *Bostryx delumbis* (Reeve, 1849), lectotype NHMUK 1975124 (H = 21.2) **D** *Bostryx ferrugineus* (Reeve, 1849), lectotype NHMUK 1975380 (H = 19.0) **E** *Bostryx luridus* (Pfeiffer, 1863), lectotype NHMUK 19991535 (H = 21.7) **F–H** *Bostryx paposensis* (Pfeiffer, 1856), paralectotype NHMUK 1975311 (H = 21.2) **H** sculpture on dorsal side of last whorl **I–K** *Bostryx pustulosus* (Broderip in Broderip and Sowerby I 1832), probable syntype NHMUK 1975589 (H = 16.4) **K** sculpture on dorsal side of penultimate whorl. All enlarged.

**Figure 10.**
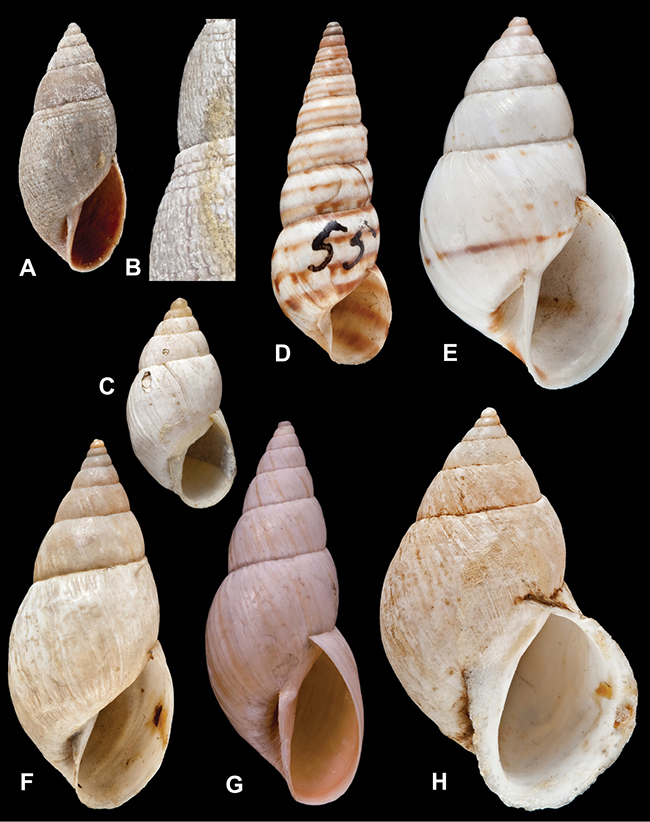
*Bostryx* species. **A–B** *Bostryx voithianus* (Pfeiffer, 1847), lectotype NHMUK 20100565.1 (H = 19.1) **B** sculpture on dorsal side of (pen)ultimate whorl **C** *Bostryx simpliculus* (Pfeiffer, 1855), lectotype NHMUK 1975340 (H = 18.9) **D** *Bostryx turritus* (Broderip in Broderip and Sowerby I 1832), possible syntype NHMUK 20100616 (H = 25.3) **E** *Bostryx virgultorum* (Morelet, 1863), paralectotype NHMUK 1893.2.4.179 (H = 27.8) **F** *Bostryx woodwardi* (Pfeiffer, 1857), lectotype NHMUK 1975334 (H = 31.0) **G** *Bostryx andoicus* (Morelet, 1863), lectotype NHMUK 1893.2.4.171 (H = 29.5) **H** *Bostryx derelictus* (Broderip in Broderip and Sowerby I 1832), probable syntype NHMUK 20100609 (H = 30.3). All enlarged.

**Figure 11.**
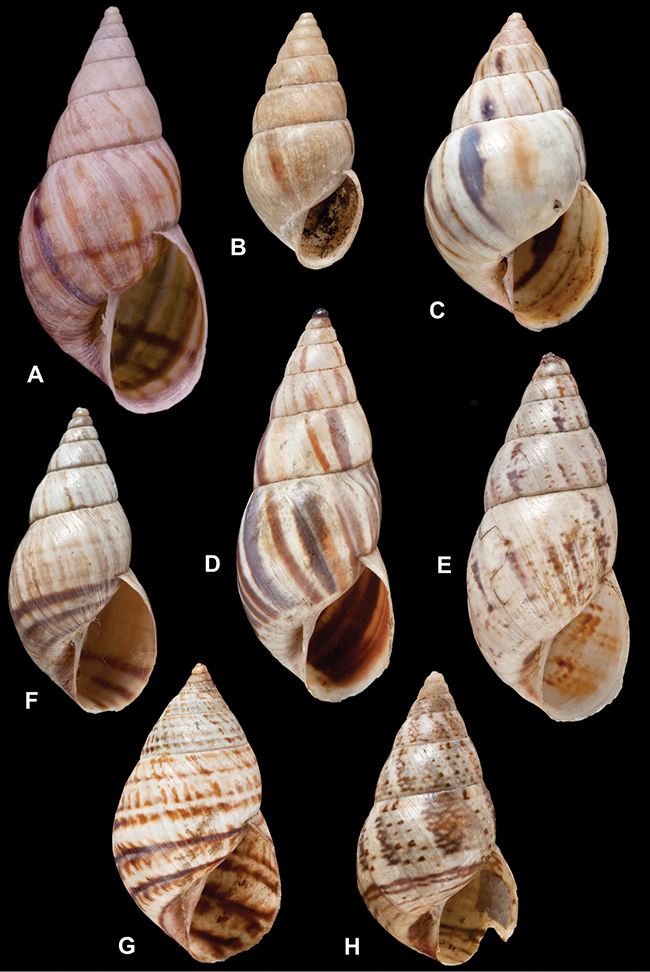
*Bostryx* species. **A** *Bostryx longinquus* (Morelet, 1863), paralectotype NHMUK 1893.2.4.185 (H = 30.9) **B** *Bostryx limonoicus* (d’Orbigny, 1835), paralectotype NHMUK 1854.12.4.190 (H = 15.8) **C–E** *Bostryx torallyi* (d’Orbigny, 1835) **C** lectotype of *Drymaeus multispira* da Costa, 1904 NHMUK 1907.11.21.31 (H = 19.9) **D** paralectotype of *Helix torallyi* d’Orbigny, 1835 NHMUK 1854.12.4.191 (H = 28.6) **E** ibidem NHMUK 1854.12.4.192 (H = 27.3) **F** *Bostryx tricinctus* (Reeve, 1848), lectotype NHMUK 1975182 (H = 19.2) **G** *Bostryx tumidulus* (Pfeiffer, 1842), lectotype NHMUK 1975324 (H = 22.7) **H** *Bostryx pictus* (Pfeiffer, 1855), lectotype NHMUK 1975545 (H = 22.5). All enlarged.

**Figure 12.**
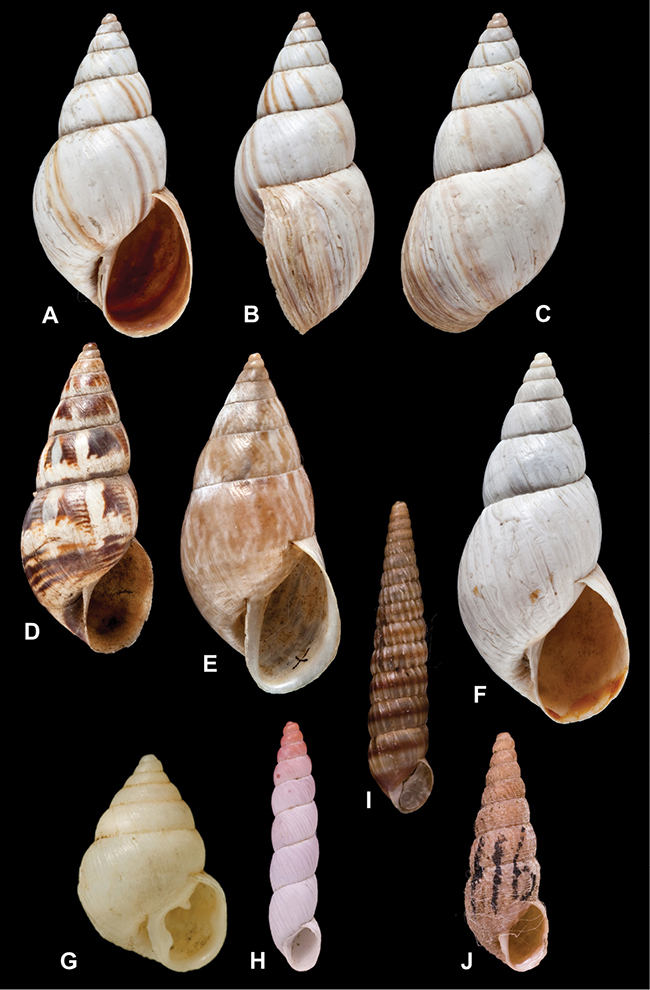
**A–F** *Bostryx* species. **A–C** *Bostryx angrandianus* (Pilsbry, 1897), lectotype of *Bulimus radiatus* Morelet, 1863 NHMUK 1893.2.4.198 (H = 23.7) **D** *Bostryx montagnei* (d’Orbigny, 1837), lectotype NHMUK 1854.12.4.194 (H = 23.3) **E** *Bostryx serotinus* (Morelet, 1860), lectotype NHMUK 1893.2.4.204 (H = 25.5) **F** *Bostryx nigropileatus* (Reeve, 1849), lectotype of *Bulimus stenacme* Pfeiffer, 1857 (H = 28.0). **G–J** *Naesiotus* species. **G** *Naesiotus wolfi* (Reibisch, 1892), paralectotype NHMUK 1894.6.8.7 (H = 12.19) **H** *Naesiotus fernandezae* Weyrauch, 1958, paratype NHMUK 19831 (H = 15.1) **I** *Naesiotus chemnitzioides* (Forbes, 1850), lectotype NHMUK 1855.4.5.23 (H = 18.5) **J** *Naesiotus rugiferus* (Sowerby I, 1833), lectotype NHMUK 1975178 (H = 12.5). All enlarged.

**Figure 13.**
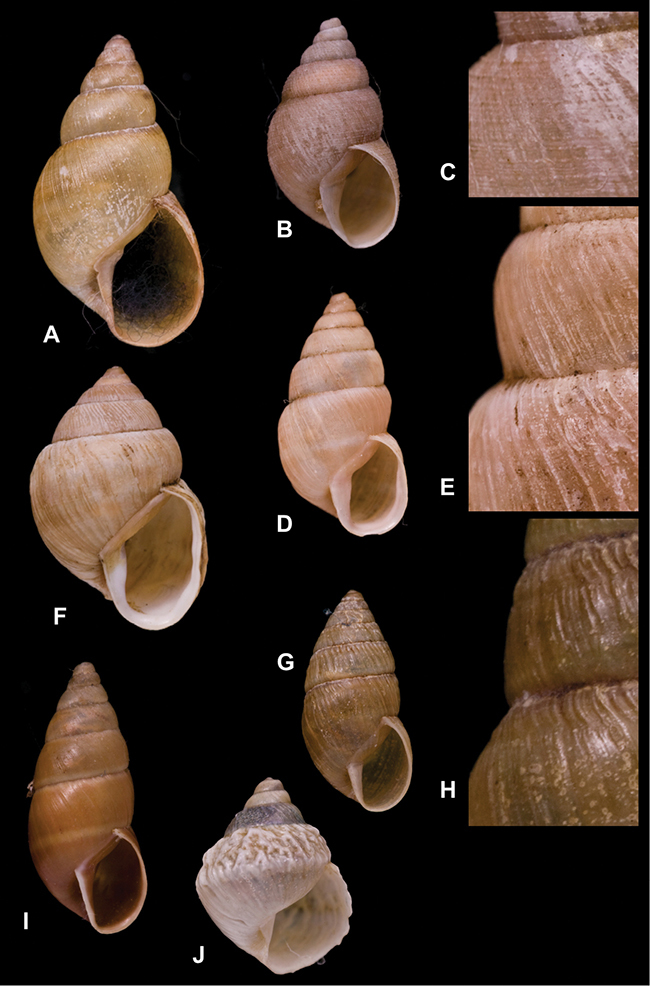
*Naesiotus* species. **A** *Naesiotus unifasciatus* (Sowerby I, 1833), lectotype NHMUK 1975187 (H = 20.4) **B–E** *Naesiotus jacobi* (Sowerby I, 1833) **B** possible syntype NHMUK 1842.5.10.250 (H = 11.9) **C** sculpture on dorsal side of ultimate whorl **D** syntype of *Bulimulus (Naesiotus) pallidus* Reibisch, 1892 NHMUK 1894.5.8.3 (H = 11.37) **E** sculpture on dorsal side of (pen)ultimate whorl **F** *Naesiotus albemarlensis* Dall, 1917, possible paratype NHMUK 1937.6.18.13 (H = 14.5) **G–H** *Naesiotus curtus* (Reibisch, 1892), syntype NHMUK 1894.6.8.8 (H = 9.6) **H** sculpture on dorsal side of (pen)ultimate whorl **I** *Naesiotus perspectivus* (Pfeiffer, 1846), lectotype NHMUK 1975166 (H = 15.7) **J** *Naesiotus darwini* (Pfeiffer, 1846), possible syntype NHMUK 20100523 (H = 8.64). All enlarged.

**Figure 14.**
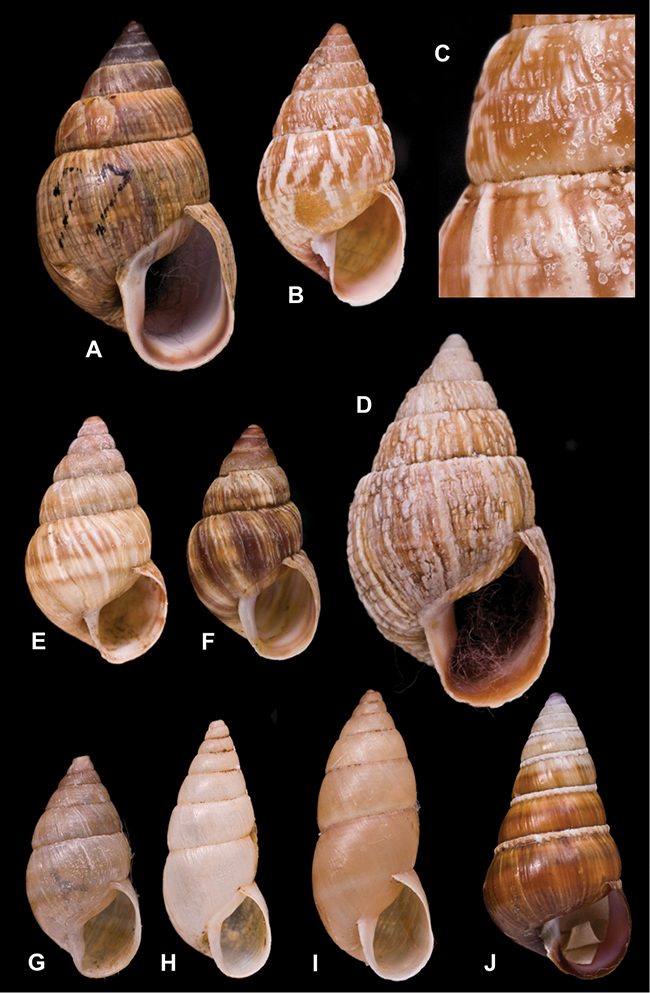
*Naesiotus* species. **A–D** *Naesiotus nux* (Broderip, 1832) **A** lectotype NHMUK 1975172 (H = 21.5) **B–C** lectotype of *Bulimus incrassatus* Pfeiffer, 1853 NHMUK 1975157 (H = 17.3) **C** sculpture on dorsal side of (pen)ultimate whorl **D** lectotype of *Bulimus verrucosus* (Pfeiffer, 1855) NHMUK 1975168 (H = 22.5) **E–F** *Naesiotus ustulatus* (Sowerby I, 1833) **E** lectotype NHMUK 20100633 (H = 13.95) **F** possible syntype of *Bulimulus (Naesiotus) ustulatus phlegonis* Dall & Ochsner, 1928 NHMUK 1937.6.18.2 (H = 12.5) **G** *Naesiotus nucula* (Pfeiffer, 1853), lectotype NHMUK 1975155 (H = 11.2) **H** *Naesiotus galapaganus* (Pfeiffer, 1855), lectotype NHMUK 1975146 (H = 15.1) **I** *Naesiotus ventrosus* (Reibisch, 1892), paralectotype NHMUK 1894.6.8.1 (H = 18.9) **J** *Naesiotus achatellinus* (Forbes, 1850), holotype NHMUK 1855.4.5.25 (H = 19.0). All enlarged.

**Figure 15.**
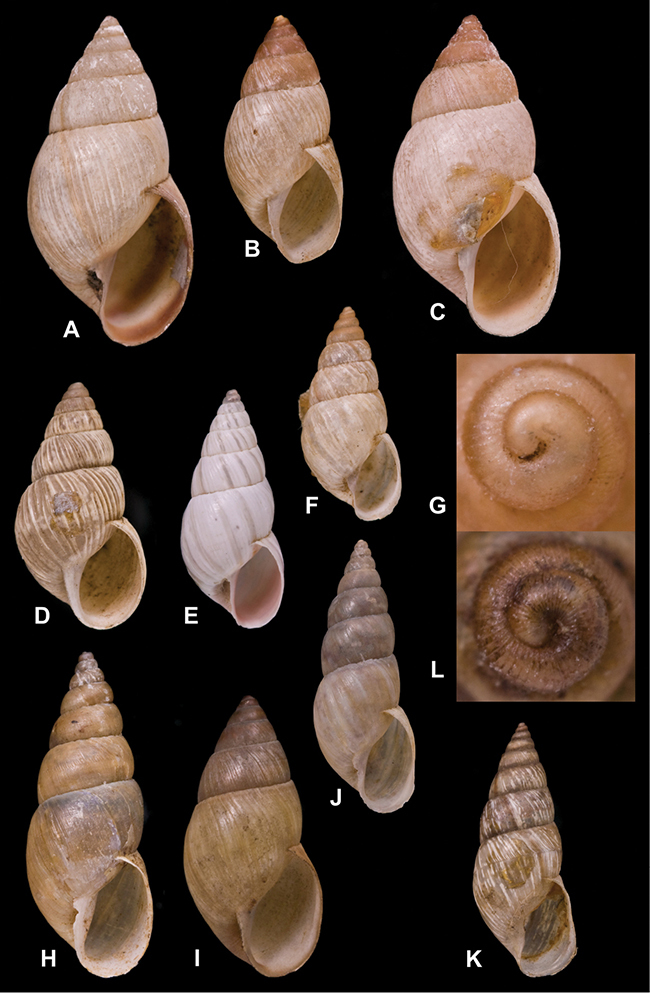
*Naesiotus* species. **A–C** *Naesiotus quitensis* (Pfeiffer, 1848) **A** lectotype NHMUK 1975320 (H = 26.5) **B** possible syntype of *Bulimus irregularis* Pfeiffer, 1848 NHMUK 20100564 (H = 18.8) **C** lectotype of *Bulimus catlowiae* Pfeiffer, 1853 NHMUK 1975414 (H = 25.1) **D** *Naesiotus orbignyi* (Pfeiffer, 1846), lectotype of *Bulimus apertus* Pfeiffer, 1855 NHMUK 1975317 (H = 18.1) **E** *Naesiotus subcostatus chamayensis* Weyrauch, 1967, paratype NHMUK 1975423 (H = 17.5) **F–G** *Naesiotus cinereus* (Reeve, 1849), lectotype NHMUK 20100519 (H = 15.5) **G** apical view of protoconch sculpture **H–I** *Naesiotus crepundia* (d’Orbigny, 1835), paralectotypes **H** NHMUK 1854.12.4.173 (H = 26.6) **I** NHMUK 1854.12.4.174 (H = 23.2) **J** *Naesiotus montivagus* (d’Orbigny, 1835), lectotype NHMUK 1854.12.4.170 (H = 20.5) **K–L** *Naesiotus rocayanus* (d’Orbigny, 1835), paralectotype NHMUK 1854.12.4.176 (H = 22.9) **L** apical view of protoconch sculpture. All enlarged.

**Figure 16.**
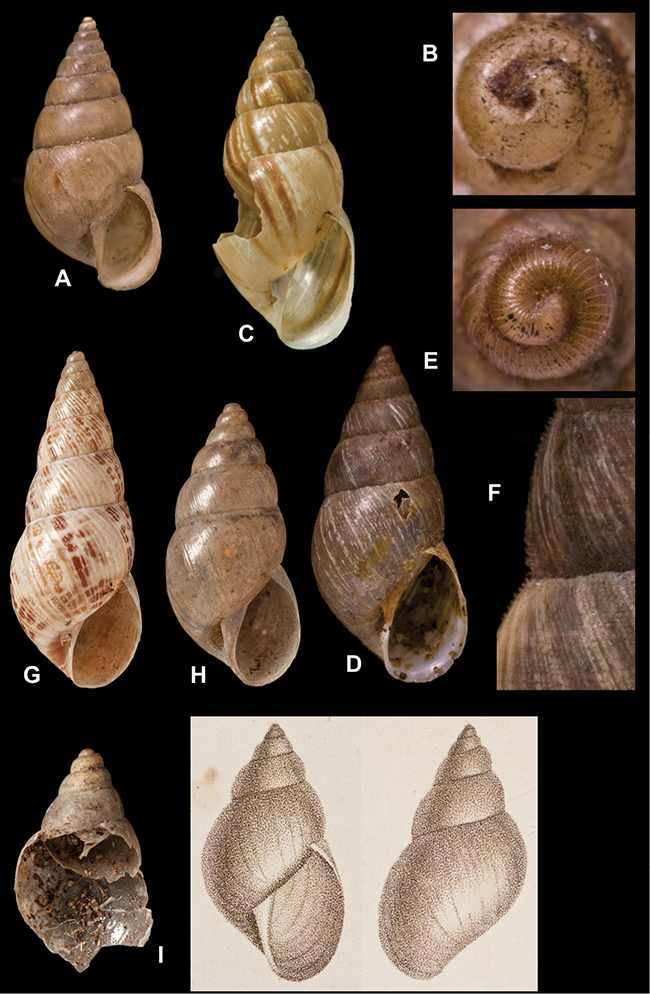
**A–F** *Naesiotus* species. **A–C** *Naesiotus rivasii* (d’Orbigny, 1837) **A–B** paralectotype NHMUK 1854.12.4.171 (H = 17.7) **B** apical view of protoconch sculpture **C** lectotype of *Bulimus sugillatus* Pfeiffer, 1857 NHMUK 1975259 (H = 23.7) **D–F** *Naesiotus trichodes* (d’Orbigny, 1835), lectotype NHMUK 1854.12.4.175 (H = 20.8) **E** apical view of protoconch sculpture **F** sculpture on dorsal side of (pen)ultimate whorl. **G** *Naesiotus dentritis* (Morelet, 1863), paralectotype NHMUK 1893.2.4.237 (H = 19.8) **H** *Naesiotus fontainii* (d’Orbigny, 1838), lectotype NHMUK 1854.12.4.165 (H = 13.2) **I** *Naesiotus fourmiersi* (d’Orbigny, 1837) NHMUK 1854.12.4.210, lectotype specimen and d’Orbigny 1837 [1834–1847]: pl. 30 figs 12–13. All enlarged.

**Figure 17.**
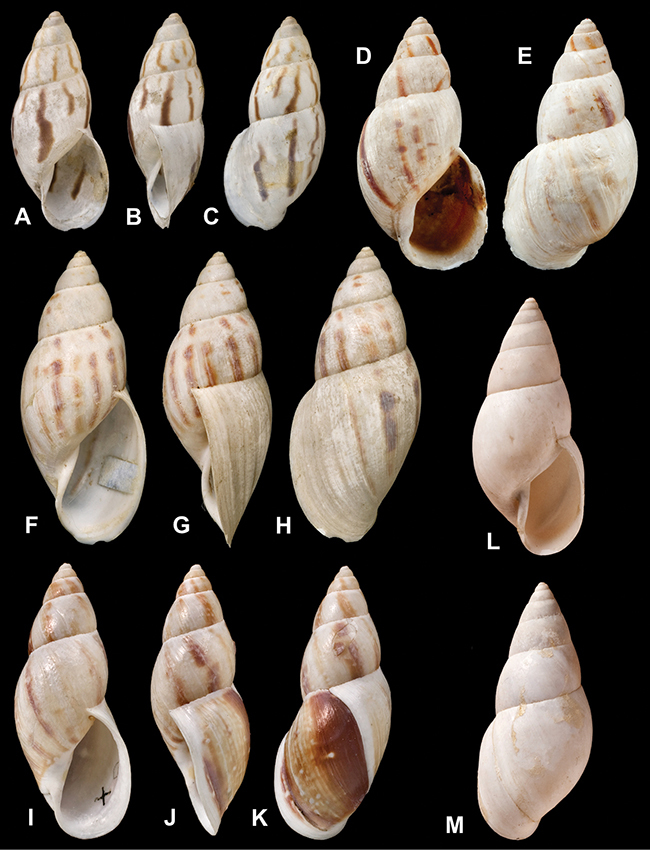
*Drymaeus* species. **A–C** *Drymaeus (Mesembrinus) lusorius* (Pfeiffer, 1855), lectotype NHMUK 1975543 (H = 24.4) **D–E** *Drymaeus (Mesembrinus) hepatostomus* (Pfeiffer, 1861), lectotype NHMUK 1975571 (H = 30.1) **F–H** *Drymaeus (Mesembrinus) attenuatus* (Pfeiffer, 1853), lectotype NHMUK 1975458 (H = 34.0) **I–K** *Drymaeus (Mesembrinus) granadensis* (Pfeiffer, 1848), lectotype of *Bulimus feriatus* Reeve, 1850, NHMUK 1975504 (H = 31.8) **L–M** *Drymaeus (Mesembrinus) immaculatus* (C.B. Adams in Reeve 1850), lectotype NHMUK 1975540 (H = 30.5). All enlarged.

**Figure 18.**
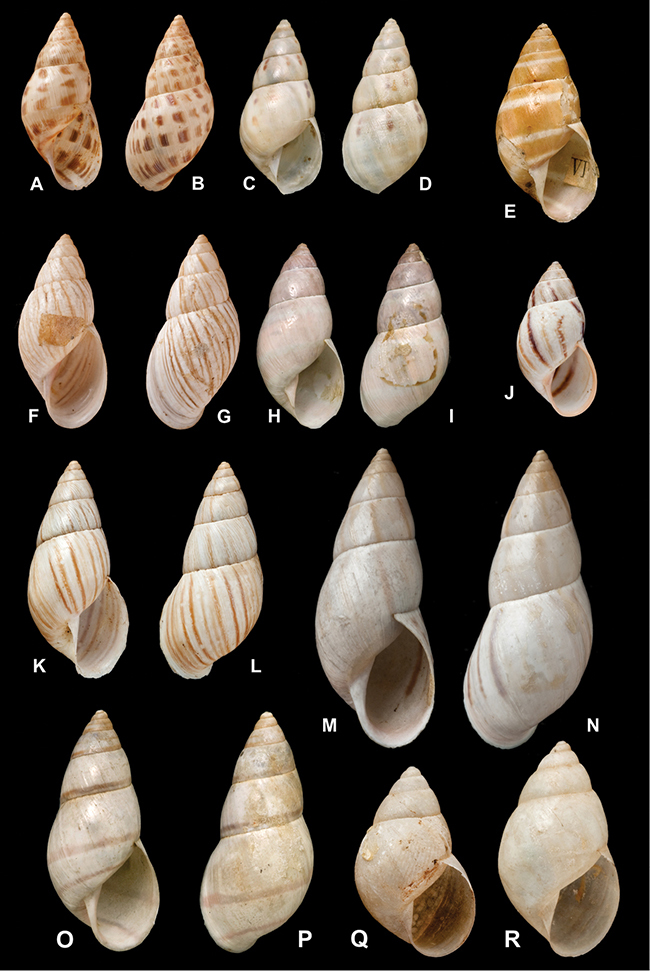
*Drymaeus* species. **A–B** *Drymaeus (Mesembrinus) interruptus* (Preston, 1909), lectotype NHMUK 1914.4.3.38 (H = 24.5) **C–D** *Drymaeus (Mesembrinus) granadensis* (Pfeiffer, 1848), lectotype of *Bulimus lividus* (Reeve, 1850), lectotype NHMUK 1975208 (H = 24.5) **E** *Drymaeus (Mesembrinus) laetus* (Reeve, 1849), lectotype NHMUK 1975534 (H = 26.6) **F–G** *Drymaeus (Mesembrinus) jonasi* (Pfeiffer in Philippi 1846), syntype NHMUK 1975557 (H = 26.6) **H–I** *Drymaeus (Mesembrinus) studeri* (Pfeiffer, 1847), lectotype of *Bulimus primula* (Reeve, 1848) NHMUK 1975478 (H = 25.0) **J** *Drymaeus (Mesembrinus) prestoni* da Costa, 1906, lectotype NHMUK 1907.11.21.12 (H = 21.3) **K–L** *Drymaeus (Mesembrinus) muliebris* (Reeve, 1849), lectotype NHMUK 1879.2.26.251 (H = 29.9) **M–N** *Drymaeus (Mesembrinus) roseatus* (Reeve, 1848), lectotype NHMUK 1975309 (H = 35.6) **O–P** *Drymaeus (Mesembrinus) studeri* (Pfeiffer, 1847), lectotype NHMUK 1975480 (H = 25.6) **Q** *Drymaeus (Mesembrinus) translucens panamensis* (Broderip in Broderip and Sowerby I 1832), possible syntype NHMUK 20100614 (H = 18.2) **R** *Drymaeus (Mesembrinus) translucens translucens* (Broderip in Broderip and Sowerby I 1832), lectotype NHMUK 20100634 (H = 21.2). All enlarged.

**Figure 19.**
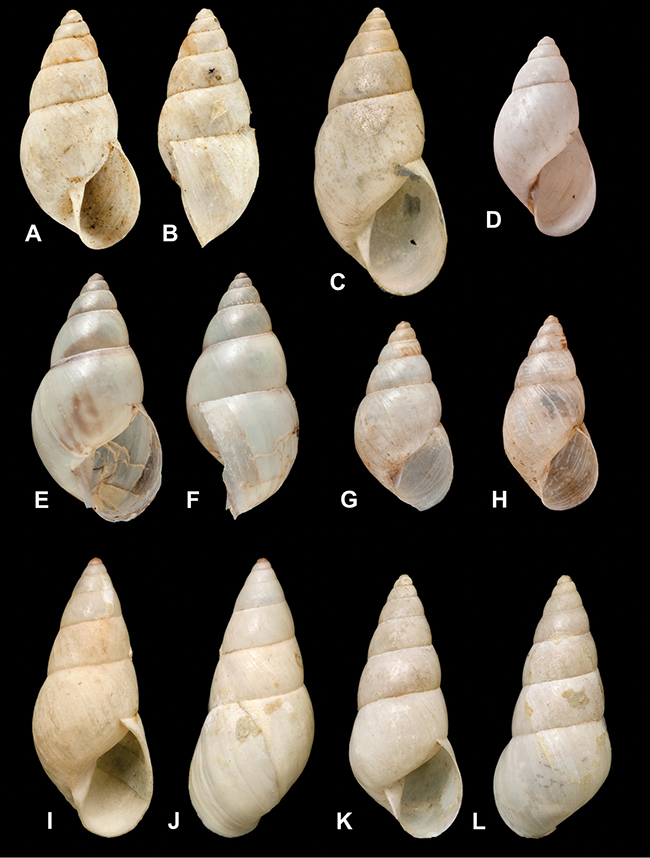
*Drymaeus* species. **A–B** *Drymaeus (Mesembrinus) aestivus* (Pfeiffer, 1857), syntype NHMUK 1975462 (H = 16.9) **C** *Drymaeus (Mesembrinus) amandus* (Pfeiffer, 1855), lectotype NHMUK 1975457 (H = 29.0) **D** *Drymaeus (Mesembrinus) castus* (Pfeiffer, 1847), lectotype NHMUK 1975197 (H = 19.0) **E–F** *Drymaeus (Mesembrinus) championi* (Martens, 1893), lectotype NHMUK 1901.6.22.451 (H = 27.0) **G–H** *Drymaeus (Mesembrinus) umbraticus* (Reeve, 1850) **G** lectotype NHMUK 1975184 (H = 15.3) **H** lectotype of *Bulimus floridanus* Pfeiffer, 1857 NHMUK 1975199 (H = 16.7) **I–J** *Drymaeus (Mesembrinus) columbiensis* (Pfeiffer, 1856), lectotype NHMUK 1975521 (H = 28.0) **K–L** *Drymaeus (Mesembrinus) columbianus* (Lea, 1838), lectotype of *Bulimus hachensis* Reeve, 1850 NHMUK 1975392 (H = 26.4). All enlarged.

**Figure 20.**
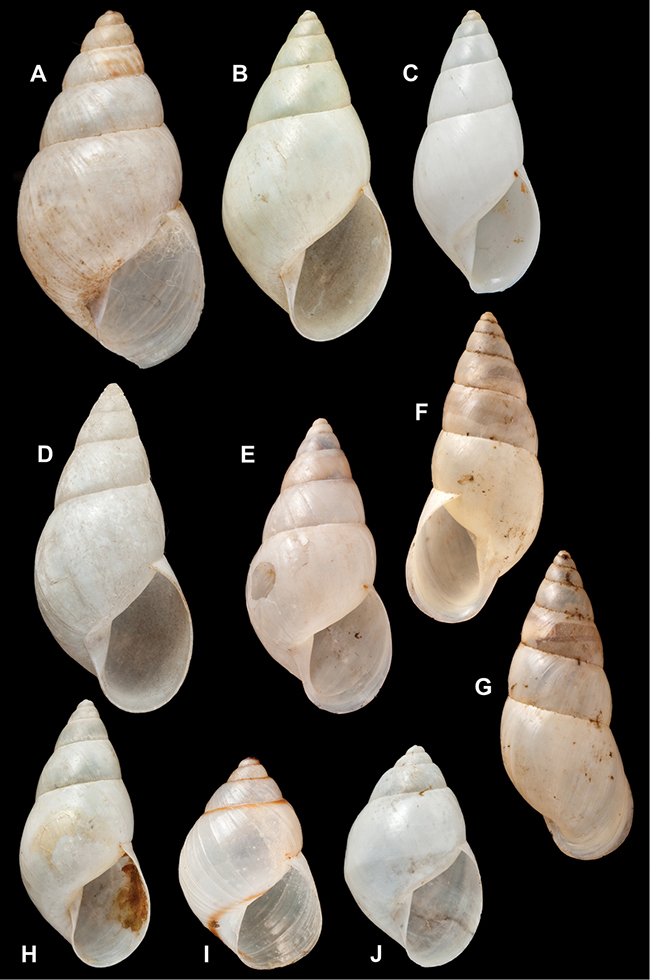
*Drymaeus* species. **A** *Drymaeus (Mesembrinus) lirinus* (Morelet, 1851), holotype NHMUK 1893.2.4.1954 (H = 30.2) **B** *Drymaeus (Mesembrinus) stramineus* (Guilding, 1828), syntype of *Bulimus lucidus* Reeve, 1848 NHMUK 1975524 (H = 28.9) **C** *Drymaeus (Mesembrinus) rawsoni* (Guppy, 1871), lectotype of *Bulimulus (Drymaeus) rawsonis* H. Adams, 1873 NHMUK 1878.1.28.209 (H = 23.7) **D–E** *Drymaeus (Mesembrinus) sulphureus* (Pfeiffer, 1857) **D** lectotype NHMUK 20100585 (H = 28.7) **E** lectotype of *Bulimus citronellus* Angas, 1879 NHMUK 1879.7.22.19 (H = 25.0) **F–G** *Drymaeus (Mesembrinus) inusitatus* (Fulton, 1900), lectotype NHMUK 1901.4.25.28 (H = 28.3) **H** *Drymaeus (Mesembrinus) puellaris* (Reeve, 1850), lectotype NHMUK 1975400 (H = 22.4) **I** *Drymaeus (Mesembrinus) broadwayi* (E.A. Smith, 1896), holotype NHMUK 1895.11.28.10 (H = 11.9) **J** *Drymaeus* sp., syntype of *Bulimus pallens* Reeve, 1849 [nomen inquirendum] NHMUK 20120056 (H = 17.9). All enlarged.

**Figure 21.**
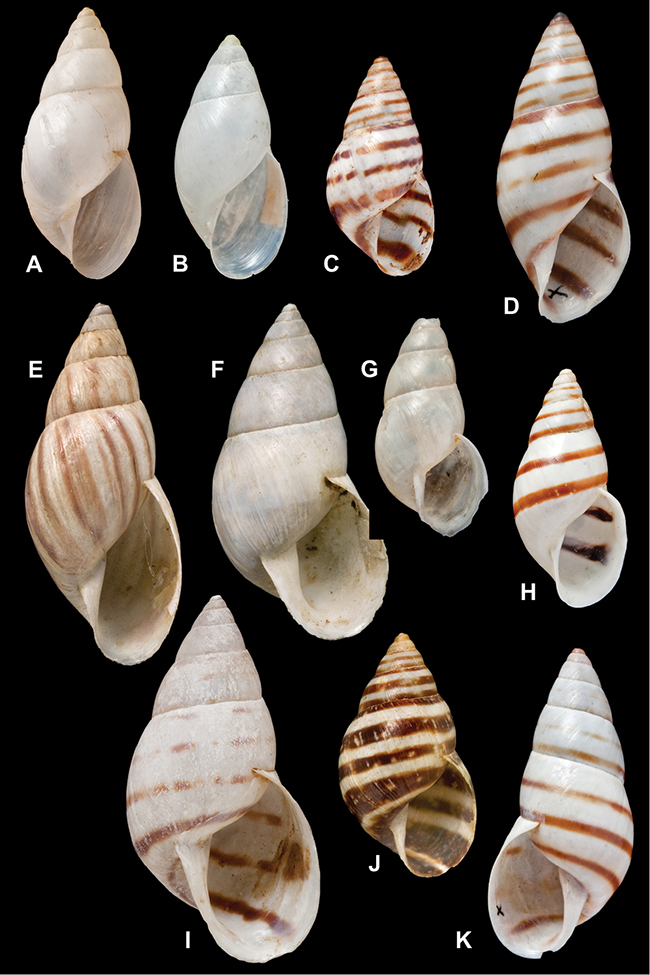
*Drymaeus* species. **A** *Drymaeus (Mesembrinus) aureolus* (Guppy, 1866), syntype NHMUK 1866.1.3.8 (H = 23.2) **B** *Drymaeus (Mesembrinus) mossi* (E.A. Smith, 1896), holotype NHMUK 1912.5.11.1 (H = 21.8) **C** *Drymaeus (Mesembrinus) imperfectus* (Guppy, 1866), lectotype of *Bulimulus (Drymaeus) trinitarius* (E.A. Smith, 1896) NHMUK 1875.2.8.2 (H = 20.1) **D** *Drymaeus (Mesembrinus) vincentinus* (Pfeiffer, 1846), lectotype NHMUK 1975219 (H = 27.9) **E** *Drymaeus (Mesembrinus) manupictus* (Reeve, 1848), lectotype NHMUK 1975522 (H = 33.3) **F** *Drymaeus (Mesembrinus) tenuilabris* (Pfeiffer, 1866), lectotype NHMUK 1975338 (H = 30.2) **G** *Drymaeus (Mesembrinus) subpellucidus* (E.A. Smith, 1877), syntype NHMUK 1872.5.22.19 (H = 20.0) **H** *Drymaeus (Mesembrinus) wintlei* Finch, 1929, lectotype NHMUK 1929.6.11.1 (H = 21.7) **I** *Drymaeus (Mesembrinus) pervariabilis* (Pfeiffer, 1853), lectotype NHMUK 1975547 (H = 33.3) **J** *Drymaeus (Mesembrinus) nigrofasciatus* (Pfeiffer in Philippi 1846), lectotype NHMUK 1975542 (H = 23.5) **K** *Drymaeus (Mesembrinus) tropicalis* (Morelet, 1849), lectotype NHMUK 1893.2.4.210 (H = 27.5). All enlarged.

**Figure 22.**
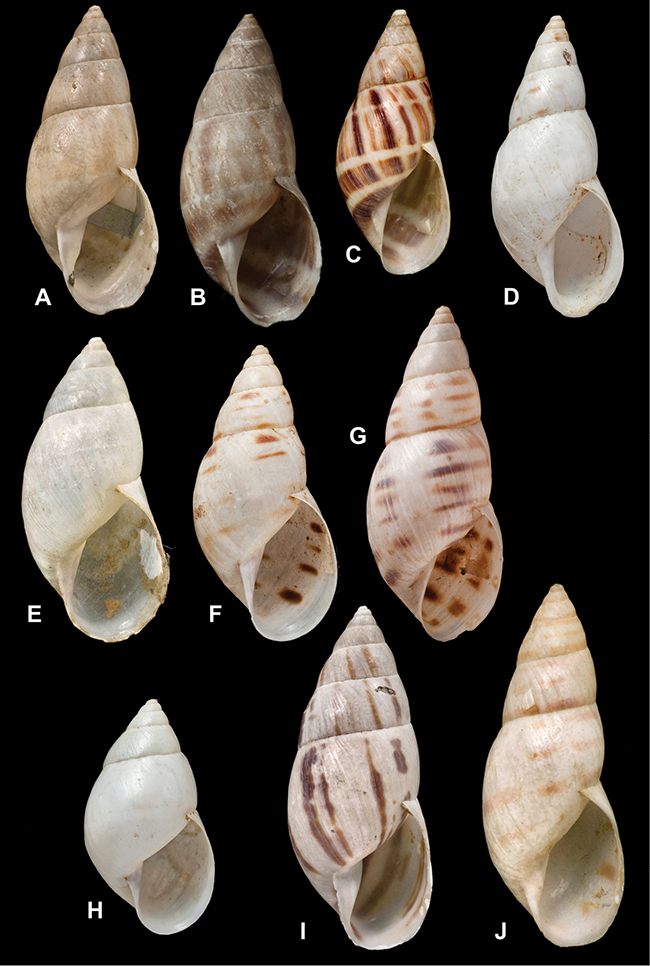
*Drymaeus* species. **A** *Drymaeus (Mesembrinus) granadensis* (Pfeiffer, 1848), lectotype of *Bulimus incarnatus* Pfeiffer, 1855 NHMUK 1975566 (H = 30.7) **B** *Drymaeus (Mesembrinus) pertristis* Pilsbry, 1898, syntype of *Bulimus tristis* Pfeiffer, 1855 NHMUK 1975299 (H = 29.3) **C** *Drymaeus (Mesembrinus) depictus* (Reeve, 1849), lectotype NHMUK 1975529 (H = 27.9) **D** *Drymaeus (Mesembrinus) trimarianus* (Martens, 1893), lectotype NHMUK 1901.6.22.950 (H = 31.5) **E** *Drymaeus (Mesembrinus) electrum* (Reeve, 1848), lectotype NHMUK 1975510 (H = 29.6) **F** *Drymaeus (Mesembrinus) serperastrus* (Say, 1830), lectotype of *Bulimus nitelinus* Reeve, 1849 NHMUK 20100570 (H = 29.4) **G** *Drymaeus (Mesembrinus) dutaillyi* (Pfeiffer, 1857), lectotype NHMUK 1975516 (H = 30.8) **H** *Drymaeus (Mesembrinus) moricandi* (Pfeiffer, 1847), lectotype NHMUK 1975212 (H = 23.6) **I** *Drymaeus (Mesembrinus) loxensis* (Pfeiffer, 1846), lectotype NHMUK 1975553 (H = 34.7) **J** *Drymaeus (Mesembrinus) meridanus* (Pfeiffer, 1846), syntype NHMUK 1975523 (H = 35.9). All enlarged.

**Figure 23.**
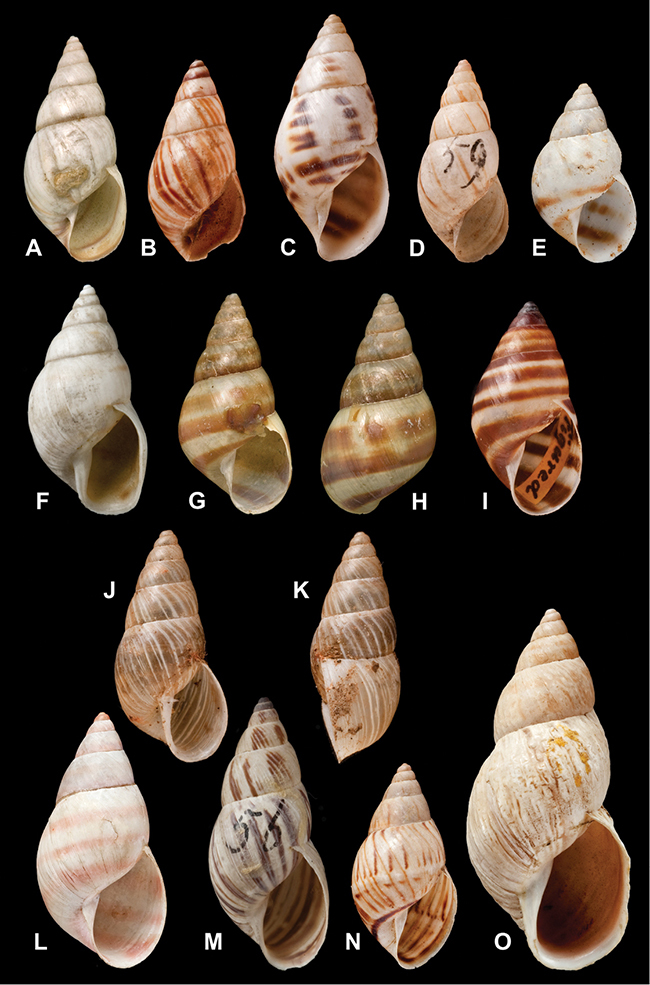
*Drymaeus* species. **A** *Drymaeus (Mesembrinus) andicola* (Pfeiffer, 1847), lectotype NHMUK 1975315 (H = 24.1) **B** *Drymaeus (Mesembrinus) elongatus* (Röding, 1789), holotype of *Bulimus apiculata* J.E. Gray, 1834 NHMUK 1982297 (H = 20.2) **C** *Drymaeus (Mesembrinus) ziegleri* (Pfeiffer, 1847), syntype of *Bulimus californicus* Reeve, 1848 NHMUK 1975136 (H = 25.9) **D** *Drymaeus (Mesembrinus) discrepans* (Sowerby I, 1833), syntype NHMUK 1975181 (H = 20.7) **E** *Drymaeus (Mesembrinus) emeus* (Say, 1830), paralectotype of *Otostomus emeus hypozonus* Martens, 1893 NHMUK 1901.6.22.794 (H = 19.3) **F** *Drymaeus (Mesembrinus) monilifer* (Reeve, 1848), lectotype NHMUK 1975403 (H = 26.3) **G–H** *Drymaeus (Mesembrinus) fidustus* (Reeve, 1849), lectotype NHMUK 1975517 (H = 22.5) **I** *Drymaeus (Mesembrinus) binominis* (E.A. Smith, 1895), lectotype of *Bulimulus (Drymaeus) binominis lascellianus* E.A. Smith, 1895 NHMUK 1895.9.10.1 (H = 22.1) **J–K** *Drymaeus (Mesembrinus) apicepunctata* (Preston, 1914), holotype NHMUK 1915.1.6.23 (H = 17.5) **L** *Drymaeus (Mesembrinus) moussoni* (Pfeiffer, 1853), lectotype NHMUK 1975211 (H = 26.0) **M** *Drymaeus (Mesembrinus) cactivorus* (Broderip in Broderip and Sowerby I 1832), possible syntype of *Bulinus nitidus* Broderip in Broderip and Sowerby I 1832 NHMUK 1975551 (H = 28.8) **N** *Drymaeus (Mesembrinus)* sp., possible syntype of *Bulimus sowerbyi* Pfeiffer, 1847 [nomen inquirendum] NHMUK 20100566 (H = 21.7) **O** *Drymaeus (Mesembrinus) sulcosus* (Pfeiffer, 1841), possible syntype NHMUK 20110084 (H = 37.0). All enlarged.

**Figure 24.**
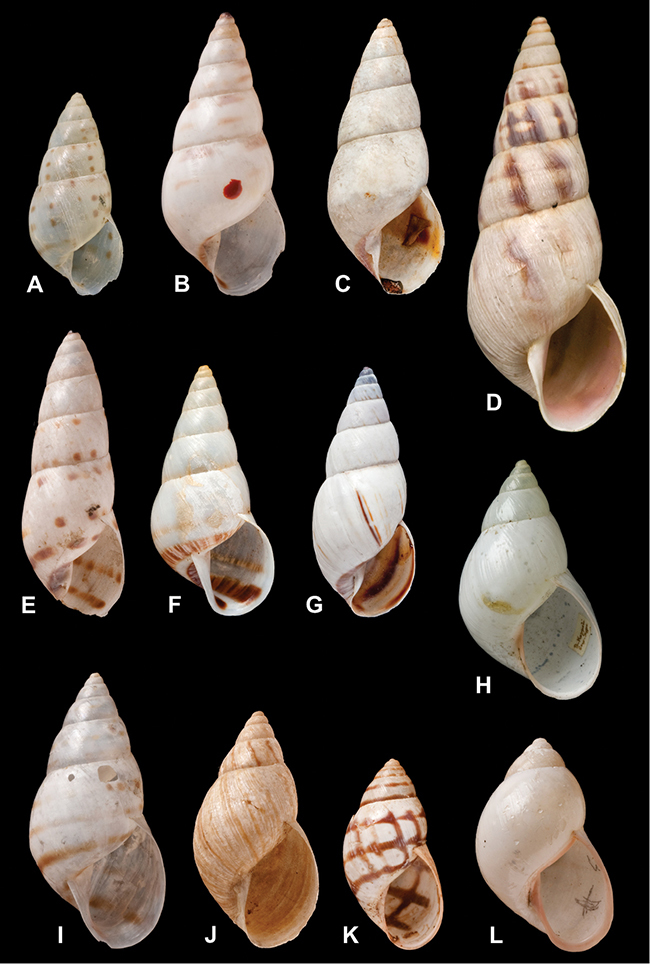
*Drymaeus* species. **A** *Drymaeus (Mesembrinus) flavidulus* (E.A. Smith, 1877), lectotype NHMUK 1975134 (H = 21.4) **B** *Drymaeus (Mesembrinus) fuscobasis* (E.A. Smith, 1877), lectotype NHMUK 1975139 (H = 28.7) **C** *Drymaeus (Mesembrinus) virginalis* (Pfeiffer, 1856), probable syntype NHMUK 1975503 (H = 27.9) **D** *Drymaeus (Mesembrinus) deshayesi* (Pfeiffer, 1845), lectotype NHMUK 1975526 (H = 44.5) **E** *Drymaeus (Mesembrinus) columbianus* (Lea, 1838), lectotype of *Bulimus gruneri* Pfeiffer, 1846 NHMUK 20100563/1 (H = 27.8) **F** *Drymaeus (Mesembrinus) rectilinearis* (Pfeiffer, 1855), NHMUK 1975337 (H = 23.9) **G** *Drymaeus (Mesembrinus) multilineatus* (Say, 1825), paralectotype of *Bulimus sisalensis* Morelet, 1849 NHMUK 1893.2.4.1655 (H = 24.1) **H** *Drymaeus (Mesembrinus) koppeli* (Sowerby III, 1892), lectotype NHMUK 1907.11.21.133 (H = 25.5) **I** *Drymaeus (Mesembrinus) hondurasanus* (Pfeiffer, 1846), lectotype NHMUK 1975265 (H = 28.6) **J** *Drymaeus (Mesembrinus) inglorius* (Reeve, 1848), lectotype NHMUK 1975536 (H = 25.6) **K** *Drymaeus (Mesembrinus) tripictus hoffmanni* (Martens, 1893), holotype of *Drymaeus prestoni cancellata* da Costa, 1906 NHMUK 1907.11.21.13 (H = 18.1) **L** *Drymaeus (Mesembrinus) gabbi* (Angas, 1879), lectotype NHMUK 1879.7.22.23 (H = 20.6). All enlarged.

**Figure 25.**
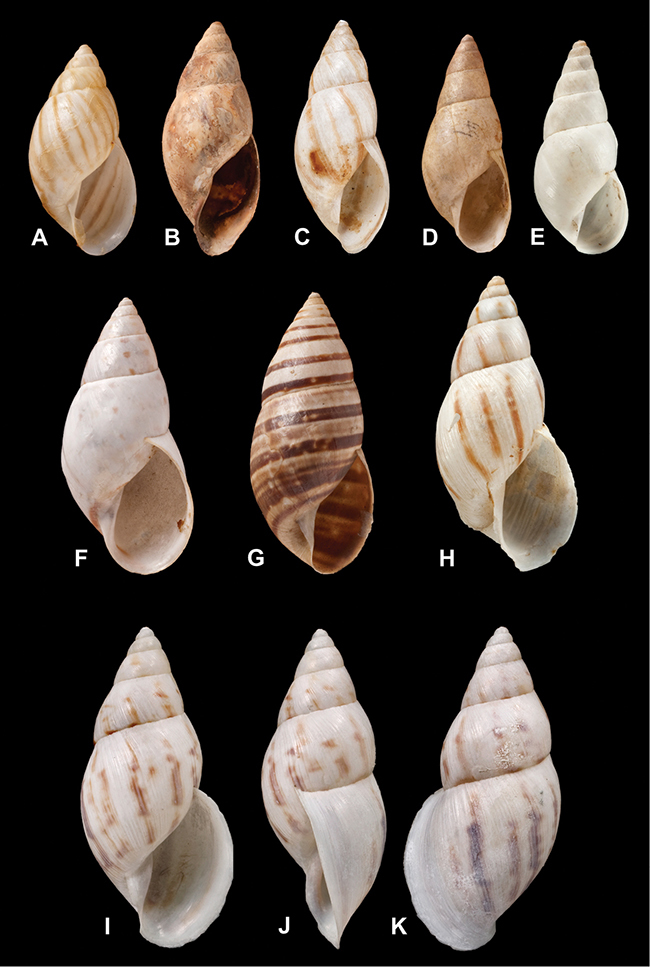
*Drymaeus* species. **A** *Drymaeus (Mesembrinus) aurifluus* (Pfeiffer, 1857), lectotype NHMUK 1975502 (H = 21.8) **B** *Drymaeus (Mesembrinus) bugabensis* (Martens, 1893), lectotype NHMUK 1901.6.22.958 (H = 26.3) **C** *Drymaeus (Mesembrinus) dubius* (Pfeiffer, 1853), lectotype NHMUK 1975519 (H = 27.1) **D** *Drymaeus (Mesembrinus) (?) rufescens* (J.E. Gray, 1825), lectotype of *Bulimus erusbescens* Pfeiffer, 1847 NHMUK 1975562 (H = 24.2) **E** *Drymaeus (Mesembrinus) conicus* da Costa, 1907, lectotype NHMUK 1907.11.21.32 (H = 17.5) **F** *Drymaeus (Mesembrinus) granadensis* (Pfeiffer, 1848), lectotype of *Bulimus demotus* Reeve, 1850 NHMUK 1975504 (H = 31.8) **G** *Drymaeus (Mesembrinus) vexillum* (Wood, 1828), lectotype of *Bulimus keppelli* Pfeiffer, 1853 NHMUK 1975538 (H = 33.2) **H** *Drymaeus (Mesembrinus) attenuatus varicosus* (Pfeiffer, 1853), lectotype NHMUK 1975466 (H = 35.5) **I–K** *Drymaeus (Drymaeus) signifer* (Pfeiffer, 1855), lectotype NHMUK 1975216 (H = 32.9). All enlarged.

**Figure 26.**
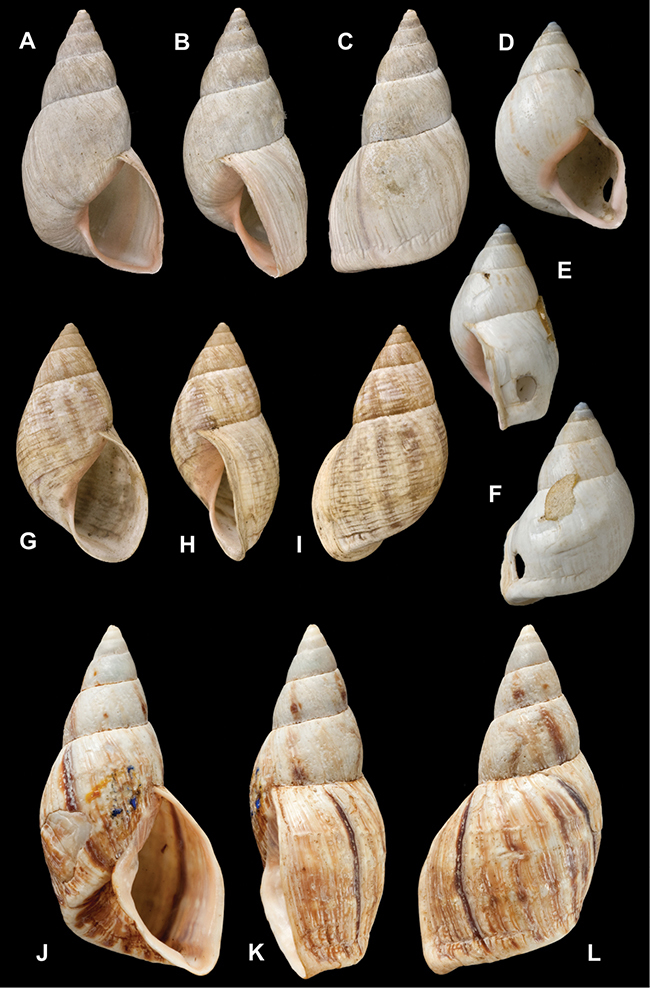
*Drymaeus* species. **A–C** *Drymaeus (Drymaeus) abscissus* (Pfeiffer, 1855), lectotype NHMUK 1975497 (H = 27.8) **D–F** *Drymaeus (Drymaeus) fallax* (Pfeiffer, 1853), lectotype NHMUK 1969142 (H = 22.4) **G–I** *Drymaeus (Drymaeus) bourcieri* (Pfeiffer, 1853), lectotype NHMUK 1975446 (H = 23.9) **J–L** *Drymaeus (Drymaeus) abruptus* (Rolle, 1904), syntype NHMUK 1947.2.10.1 (H = 42.5). All enlarged.

**Figure 27.**
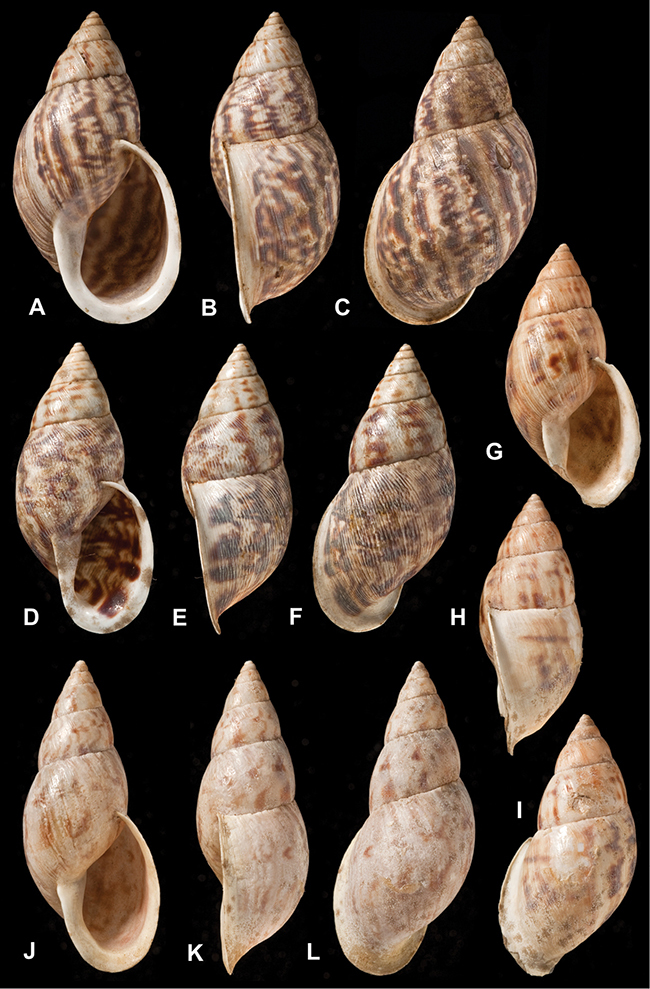
*Drymaeus* species. **A–F** *Drymaeus (Drymaeus) abyssorum* (d’Orbigny, 1835) **A–C** Paralectotype of *Helix abyssorum* d’Orbigny, 1835, NHMUK 1854.12.4.125 (H = 43.7) **D–F** lectotype of *Helix hygrohylaea* d’Orbigny, 1835, NHMUK 1854.12.4.127 (H = 40.4) **G–I** *Drymaeus (Drymaeus) marmarinus* (d’Orbigny, 1835), lectotype NHMUK 1854.12.4.129 (H = 37.9) **J–L** *Drymaeus (Drymaeus) xanthostomus* (d’Orbigny, 1835), paralectotype NHMUK 1854.12.4.221 (H = 43.8). All enlarged.

**Figure 28.**
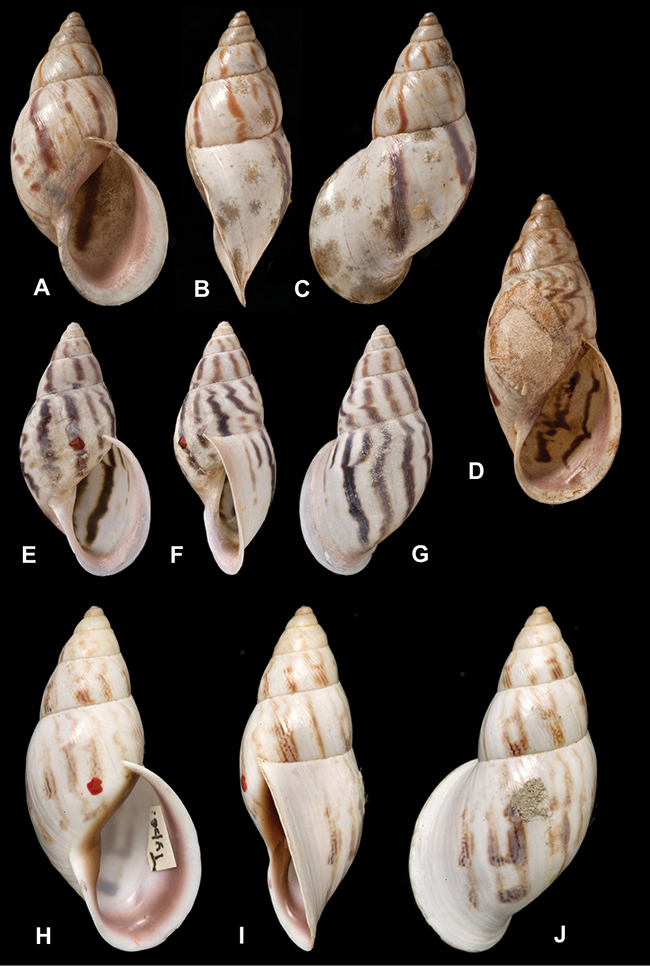
*Drymaeus* species. **A–C** *Drymaeus (Drymaeus) linostoma* (d’Orbigny, 1835), paralectotype NHMUK 1854.12.4.132 (H = 29.7) **D** *Drymaeus (Drymaeus) zoographicus* (d’Orbigny, 1835), paralectotype NHMUK 1854.12.4.131 (H = 31.7) **E–G** *Drymaeus (Drymaeus) aequatorianus* (E.A. Smith, 1877), lectotype NHMUK 1975137 (H = 26.6) **H–J** *Drymaeus (Drymaeus) bellus* da Costa, 1906, holotype NHMUK 1907.11.21.8 (H = 33.2). All enlarged.

**Figure 29.**
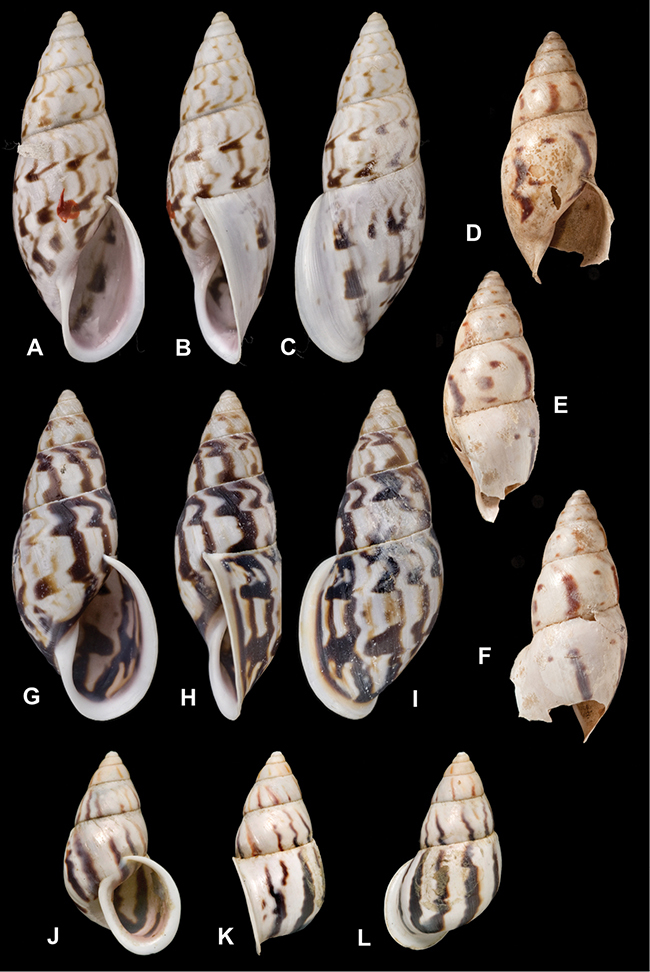
*Drymaeus* species. **A–C** *Drymaeus (Drymaeus) orthostoma* (E.A. Smith, 1877), lectotype NHMUK 1975132 (H = 36.5) **D–F** *Drymaeus (Drymaeus) fusoides* (d’Orbigny, 1835), paralectotype NHMUK 1854.12.4.133 (H = 24.6) **G–I** *Drymaeus (Drymaeus) albolabiatus* (E.A. Smith, 1877), holotype NHMUK 1877.3.28.3 (H = 34.0) **J–L** *Drymaeus (Drymaeus) strigatus* (Sowerby I, 1833), lectotype of *Bulimus musivus* Pfeiffer, 1855, NHMUK 1975292 (H = 21.5). All enlarged.

**Figure 30.**
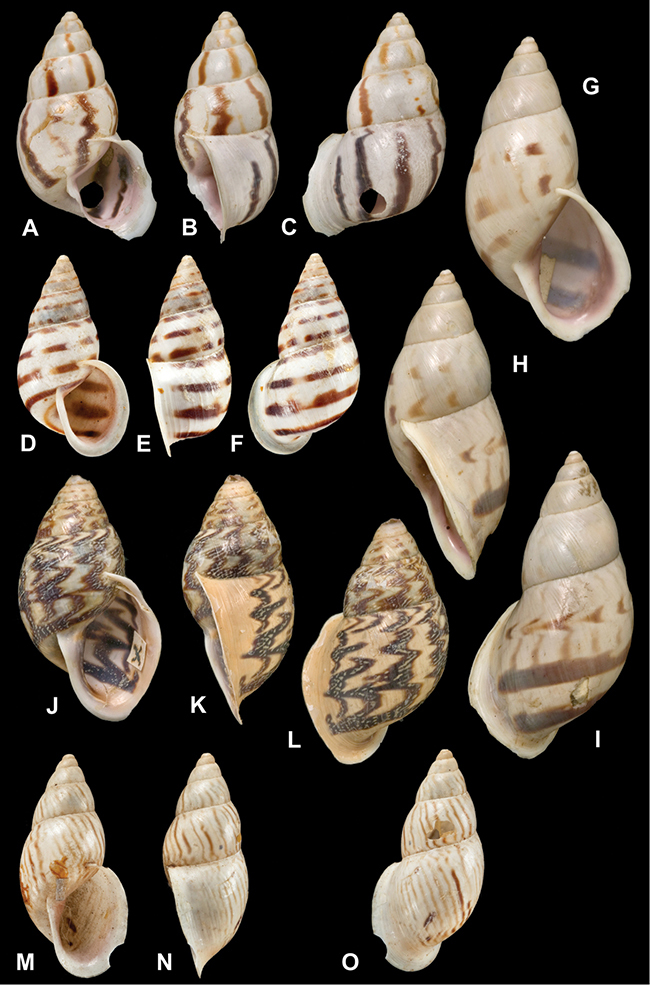
*Drymaeus* species. **A–F** *Drymaeus (Drymaeus) strigatus* (Sowerby I, 1833) **A–C** possible syntype NHMUK 20090168 (H = 24.9) **D–F** lectotype of *Bulimus saccatus* Pfeiffer, 1855 NHMUK 1975207 (H = 21.5) **G–I** *Drymaeus (Drymaeus) smithii* (da Costa, 1898), holotype NHMUK 1907.11.21.52 (H = 29.5) **J–L** *Drymaeus (Drymaeus) ziczac* (da Costa, 1898), lectotype NHMUK 1907.11.21.46 (H = 26.5) **M–O** *Drymaeus (Drymaeus) gueinzii* (Pfeiffer, 1857), lectotype NHMUK 1975539 (H = 23.2). All enlarged.

**Figure 31.**
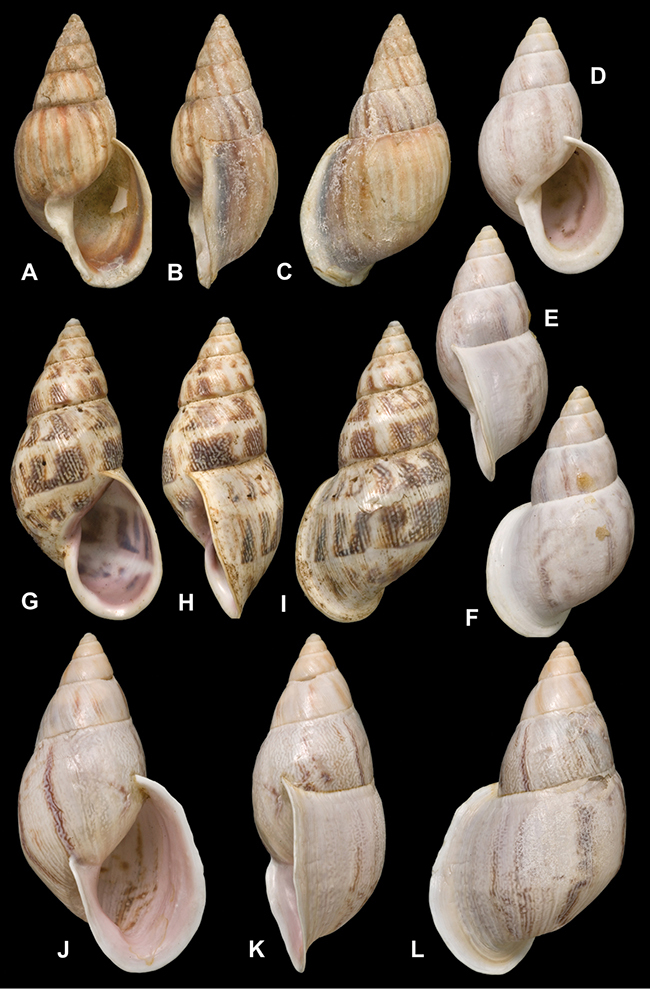
*Drymaeus* species. **A–F** *Drymaeus (Drymaeus) baranguillanus* (Pfeiffer, 1853) **A–C** lectotype NHMUK 1975452 (H = 31.5) **D–F** lectotype of *Bulimus antioquensis* Pfeiffer, 1855 NHMUK 1975450 (H = 29.6) **G–I** *Drymaeus (Drymaeus) caucaensis* (da Costa, 1898), lectotype NHMUK 1907.11.21.43 (H = 34.8) **J–L** *Drymaeus (Drymaeus) confluens* (Pfeiffer, 1855), lectotype NHMUK 1975196 (H = 39.5). All enlarged.

**Figure 32.**
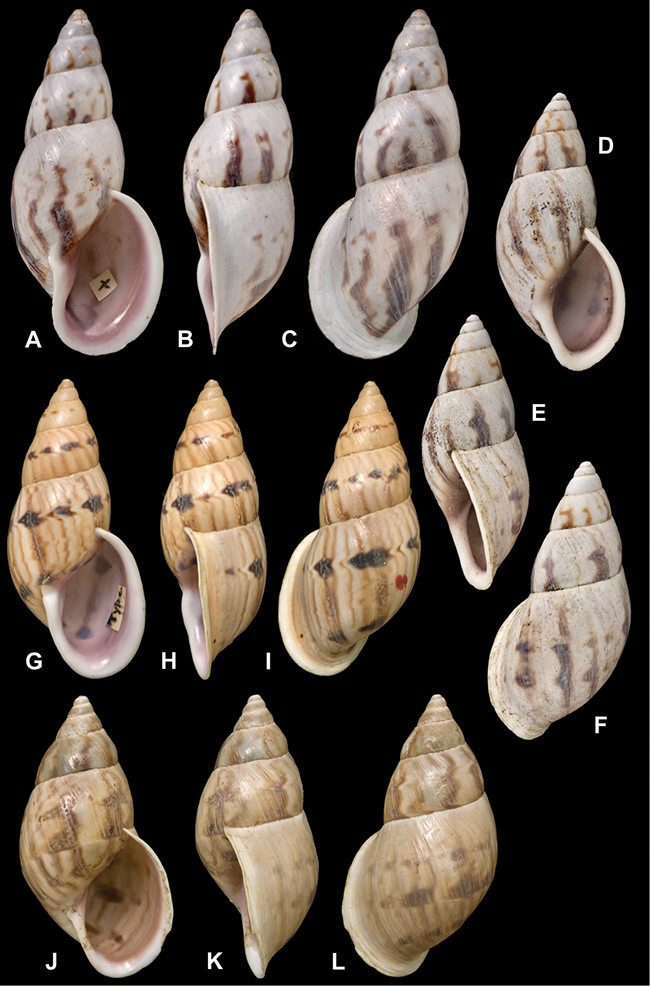
*Drymaeus* species. **A–C** *Drymaeus (Drymaeus) hidalgoi* (da Costa, 1898), lectotype NHMUK 1907.11.21.28 (H = 39.0) **D–F** *Drymaeus (Drymaeus) solidus* (Preston, 1907), syntype NHMUK 1908.7.2.72 (H = 32.8) **G–I** *Drymaeus (Drymaeus) notatus* (da Costa, 1906), lectotype NHMUK 1907.11.21.6 (H = 34.5) **J–L** *Drymaeus (Drymaeus) notabilis* da Costa, 1906, lectotype NHMUK 1907.11.21.5 (H = 32.8). All enlarged.

**Figure 33.**
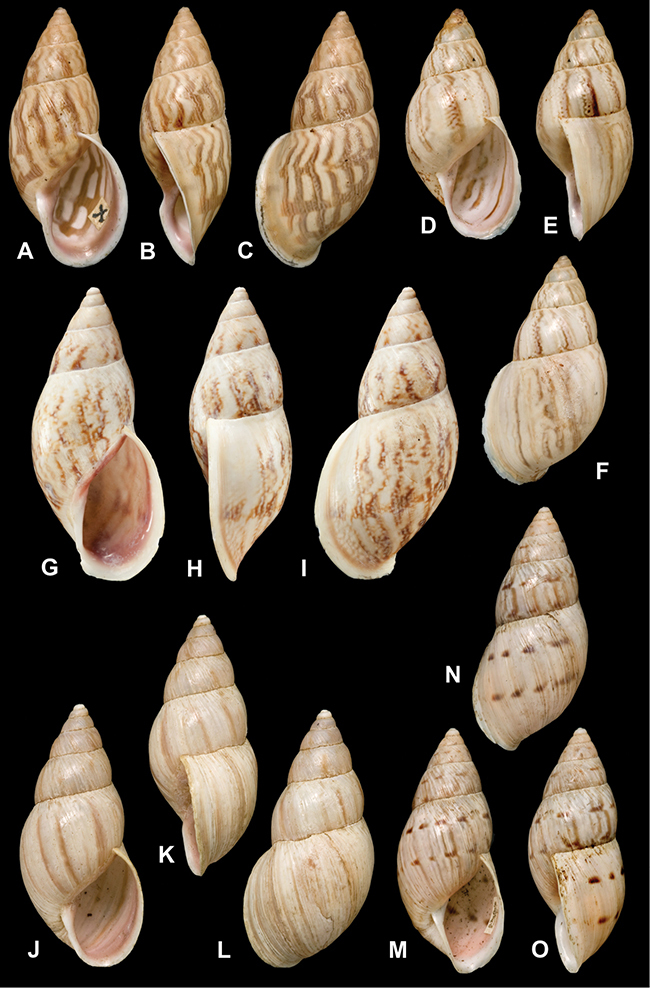
*Drymaeus* species. **A–C** *Drymaeus (Drymaeus) incognitus* da Costa, 1907, holotype NHMUK 1907.11.21.24 (H = 29.8) **D–F** *Drymaeus (Drymaeus) exoticus* (da Costa, 1901), lectotype NHMUK 1907.11.21.38 (H = 24.8) **G–I** *Drymaeus (Drymaeus) elsteri* da Costa, 1901, lectotype NHMUK 1907.11.21.34 (H = 33.9) **J–L** *Drymaeus (Drymaeus) subventricosus* da Costa, 1901, lectotype NHMUK 1907.11.21.37 (H = 30.1) **M–O** *Drymaeus (Drymaeus) dacostae* (Sowerby III, 1892), holotype NHMUK 1907.11.21.51 (H = 26.3). All enlarged.

**Figure 34.**
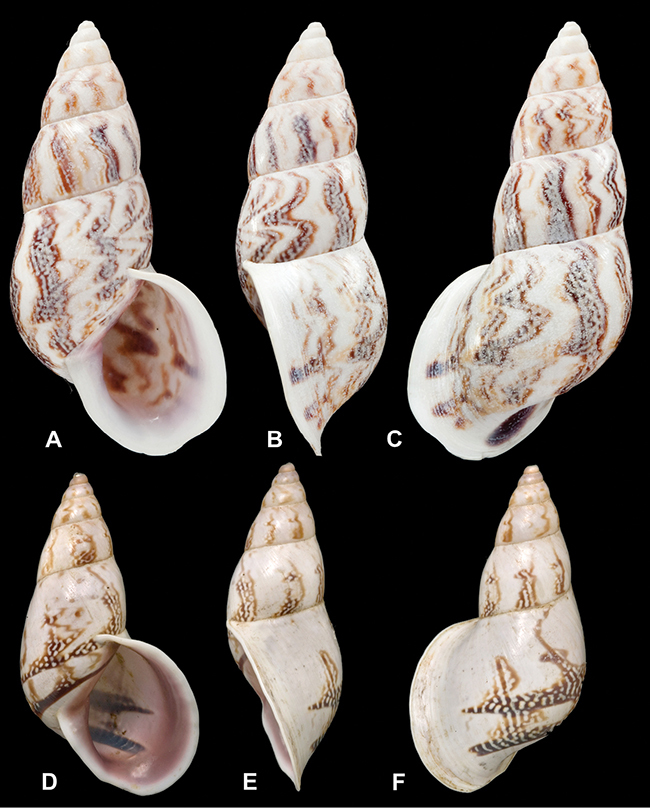
*Drymaeus* species. **A–C** *Drymaeus (Drymaeus) flexuosus* (Pfeiffer, 1853), lectotype NHMUK 1975202 (H = 43.0) **D–F** *Drymaeus (Drymaeus) spadiceus* da Costa, 1906, holotype NHMUK 1907.11.21.15 (H = 37.3). All enlarged.

**Figure 35.**
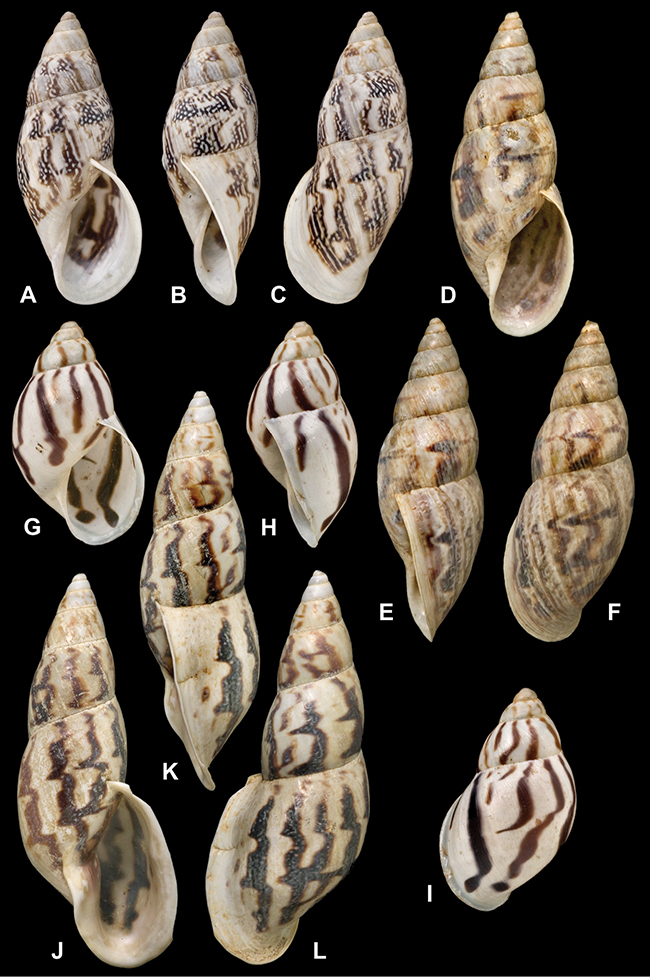
*Drymaeus* species. **A–C** *Drymaeus (Drymaeus) volsus* Fulton, 1907, lectotype NHMUK 1907.5.3.162 (H = 30.3) **D–F** *Drymaeus (Drymaeus) yungasensis* (d’Orbigny, 1837), lectotype NHMUK 1854.12.4.134 (H = 32.6) **G–I** *Drymaeus (Drymaeus) lucidus* (da Costa, 1898), lectotype NHMUK 1907.11.21.44 (H = 18.6) **J–L** *Drymaeus (Drymaeus) sykesi* da Costa, 1906, holotype NHMUK 1907.11.21.4 (H = 51.7). All enlarged.

**Figure 36.**
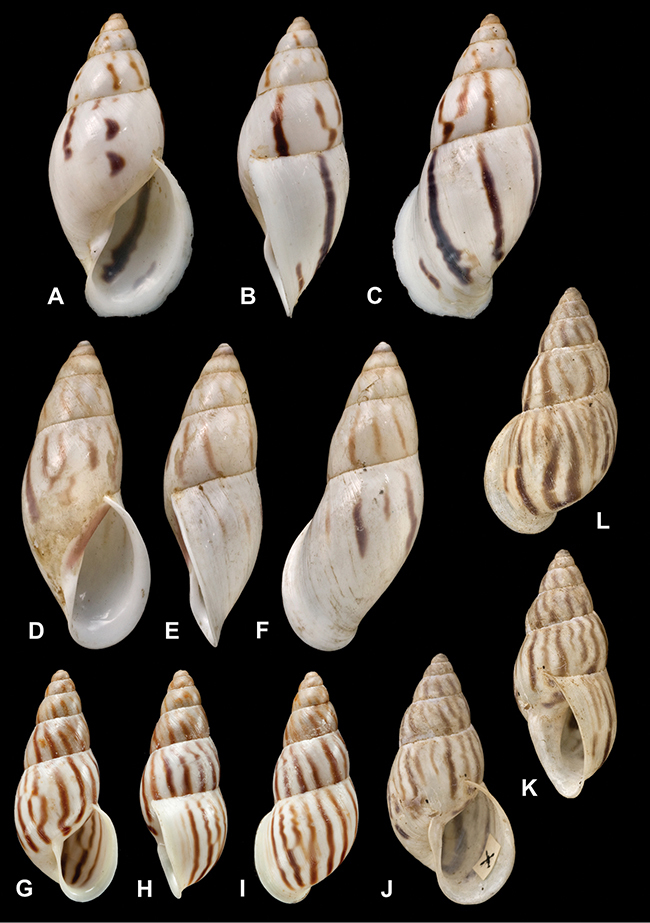
*Drymaeus* species. **A–C** *Drymaeus (Drymaeus) boucardi* da Costa, 1907, holotype NHMUK 1907.11.21.26 (H = 26.9) **D–F** *Drymaeus (Drymaeus) buckleyi* (Sowerby III, 1895), lectotype NHMUK 1907.11.21.48 (H = 27.3) **G–I** *Drymaeus (Drymaeus) castaneostrigatus* da Costa, 1906, holotype NHMUK 1907.11.21.13 (H = 20.4) **J–L** *Drymaeus (Drymaeus) tigrinus* (da Costa, 1898), lectotype NHMUK 1907.11.21.55 (H = 20.8). All enlarged.

**Figure 37.**
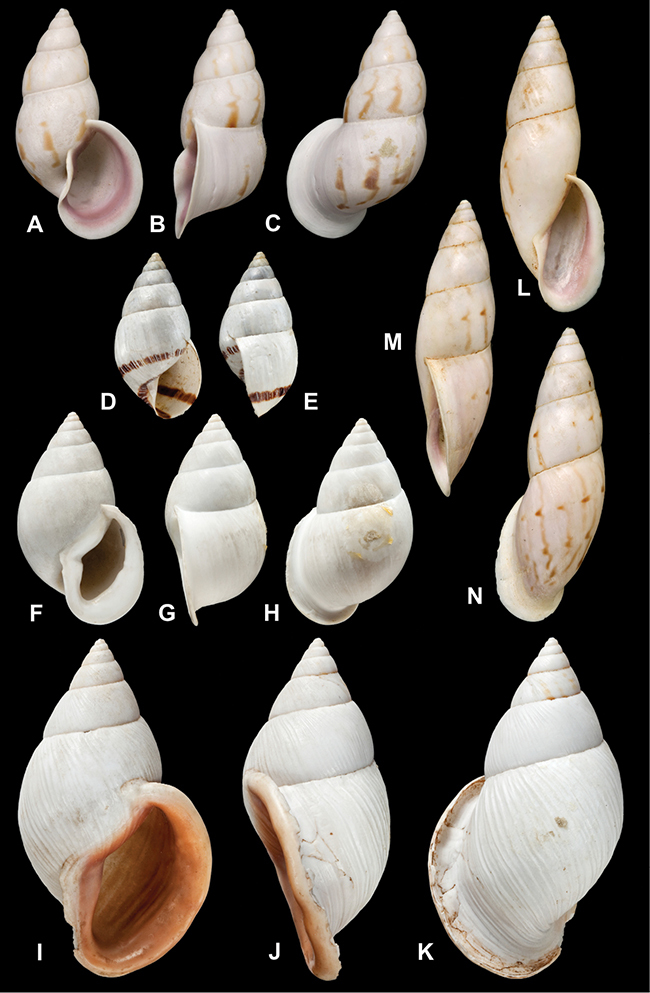
*Drymaeus* species. **A–C** *Drymaeus (Drymaeus) auris* (Pfeiffer, 1866), lectotype NHMUK 1975499 (H = 38.8) **D–E** *Drymaeus (Drymaeus) mexicanus* (Lamarck, 1822), lectotype of *Bulimus humboldtii* Reeve, 1849 NHMUK 1975528 (H = 27.6) **F–H** *Drymaeus (Drymaeus) coarctatus* (Pfeiffer, 1845), lectotype 1975560 (H = 34.6) **I–K** *Drymaeus (Drymaeus) dombeyanus* (Pfeiffer, 1846), syntype NHMUK 1844.10.2.32 (H = 56.8) **L–N** *rymaeus (Drymaeus)* angustus da Costa, 1906, holotype NHMUK 1907.11.21.14 (H = 31.5). All enlarged.

**Figure 38.**
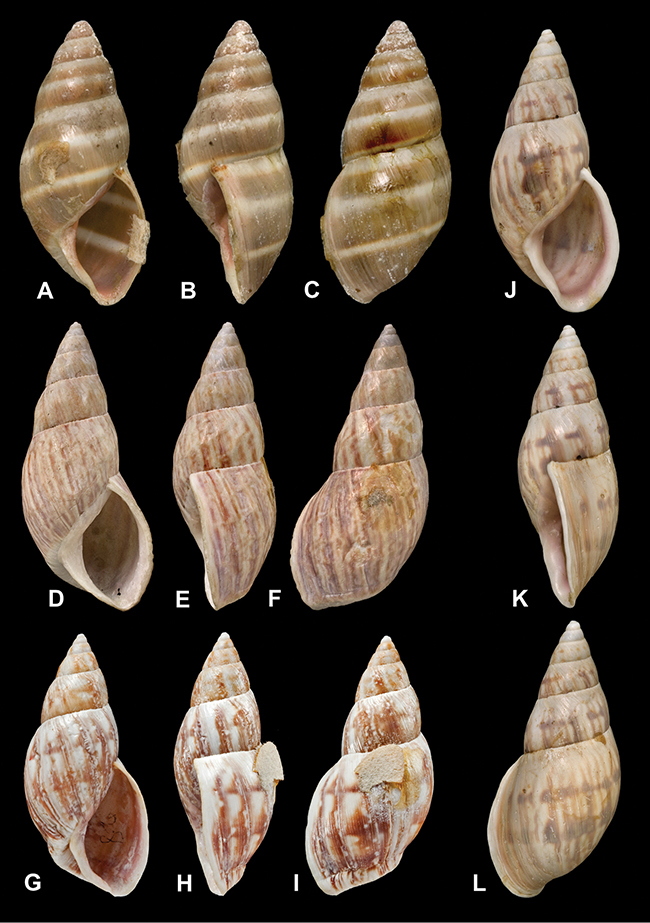
*Drymaeus* species. **A–C** *Drymaeus (Drymaeus) chimborasensis* (Reeve, 1848), syntype NHMUK 1975460 (H = 39.7) **D–F** *Drymaeus (Drymaeus) fabrefactus* (Reeve, 1848), lectotype NHMUK 1975531 (H = 38.5) **G–I** *Drymaeus (Drymaeus) canaliculatus* (Pfeiffer, 1845), lectotype NHMUK 1975514 (H = 36.0) **J–L** *Drymaeus (Drymaeus) murrinus* (Reeve, 1848), lectotype NHMUK 1975213 (H = 37.1). All enlarged.

**Figure 39.**
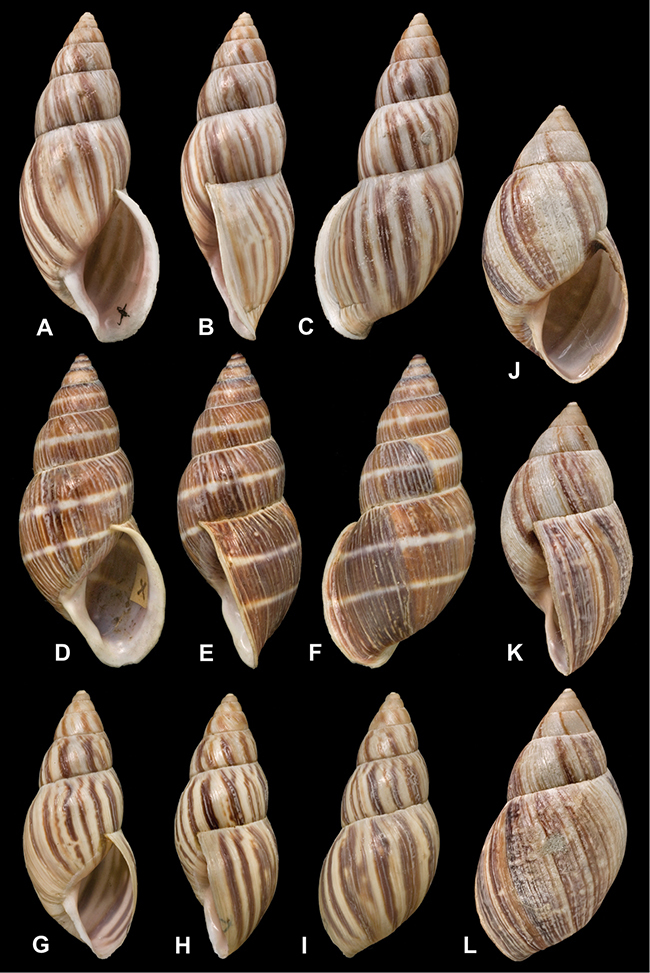
*Drymaeus* species. **A–F** *Drymaeus (Drymaeus) convexus* (Pfeiffer, 1855) **A–C** lectotype NHMUK 1975192 (H = 38.5) **D–F** lectotype of *Bulimulus (Drymaeus) plicatoliratus* da Costa, 1898, NHMUK 1907.11.21.120 (H = 36.6) **G–I** *Drymaeus (Drymaeus) phryne* (Pfeiffer, 1863), lectotype NHMUK 1975214 (H = 30.8) **J–L** *Drymaeus (Drymaeus) nystianus* (Pfeiffer, 1853), lectotype NHMUK 1975573 (H = 31.9). All enlarged.

**Figure 40.**
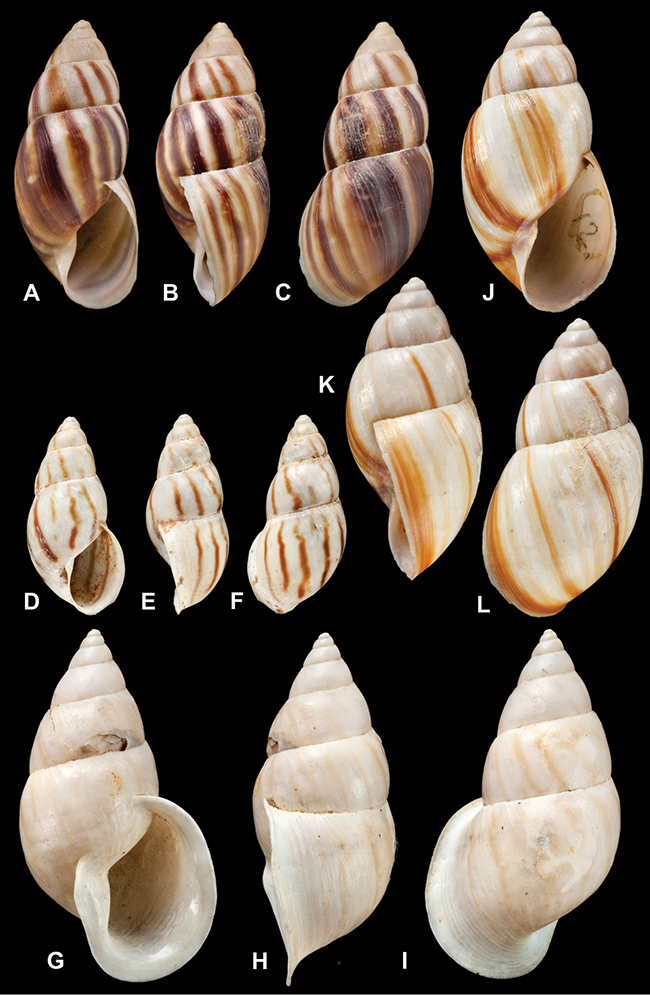
*Drymaeus* species. **A–C** *Drymaeus (Drymaeus) chamaeleon* (Pfeiffer, 1855), syntype NHMUK 1975448 (H = 27.6) **D–F** *Drymaeus (Drymaeus) rosenbergi* da Costa, 1900, lectotype NHMUK 1907.11.21.17 (H = 19.0) **G–I** *Drymaeus (Drymaeus) zhorquinensis* (Angas, 1879), lectotype NHMUK 1879.7.22.15 (H = 46.1) **J–L** *Drymaeus (Drymaeus) ambustus* (Reeve, 1849), lectotype NHMUK 1975441/1 (H = 29.0). All enlarged.

**Figure 41.**
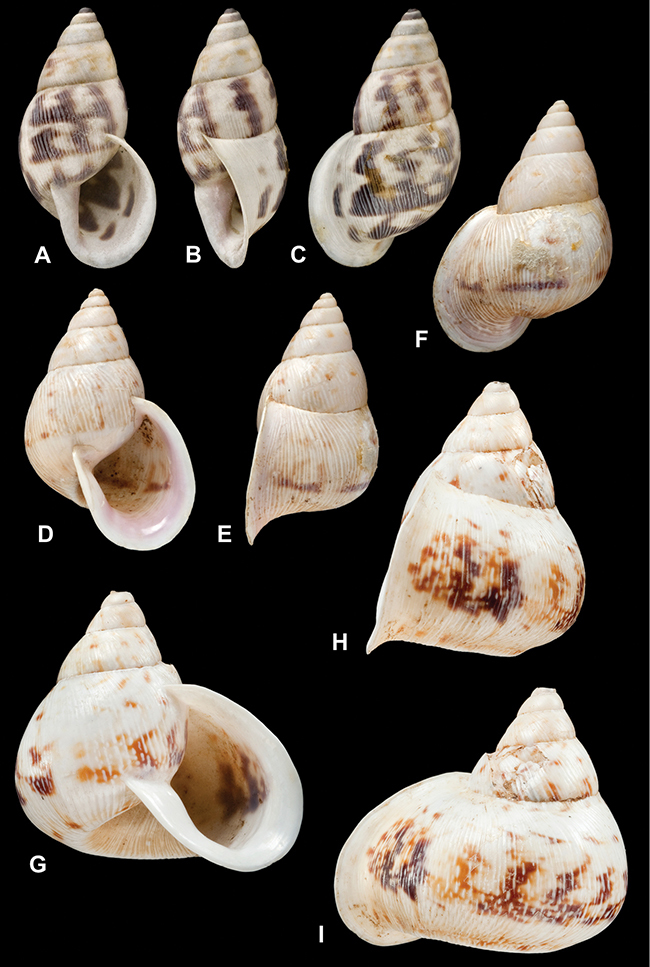
*Drymaeus* species. **A–C** *Drymaeus (Drymaeus) arcuatostriatus* (Pfeiffer, 1855), lectotype NHMUK 1975455 (H = 27.6) **D–F** *Drymaeus (Drymaeus) expansus* (Pfeiffer, 1848), holotype of *Otostomus scitus* H. Adams, 1867 NHMUK 1867.5.18.5 (H = 27.1) **G–I** *Drymaeus (Drymaeus) bartletti* (H. Adams, 1867), lectotype NHMUK 1867.5.18.4 (H = 28.5). All enlarged.

**Figure 42.**
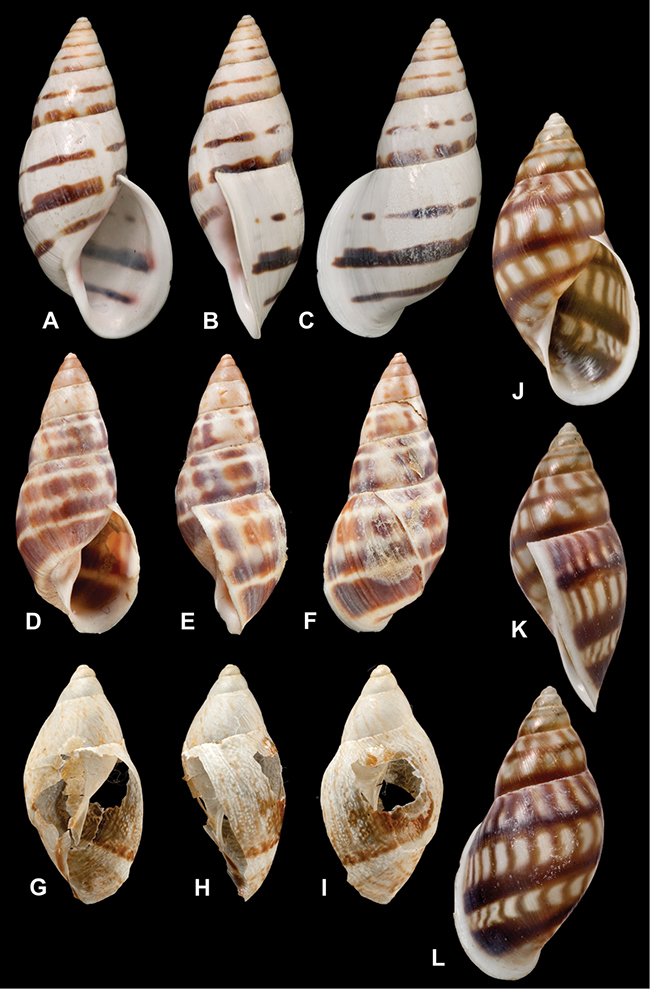
*Drymaeus* species. **A–C** *Drymaeus (Drymaeus) bogotensis* (Pfeiffer, 1855), lectotype NHMUK 1975191 (H = 37.4) **D–F** *Drymaeus (Drymaeus) bolivianus* (Pfeiffer, 1846), lectotype NHMUK 1975444 (H = 33.0) **G–I** *Drymaeus (Drymaeus) cuticula* (Pfeiffer, 1855), lectotype NHMUK 1975451 (H = 27.5) **J–L** *Drymaeus (Drymaeus) chiriquensis* da Costa, 1901 (H = 29.1). All enlarged.

**Figure 43.**
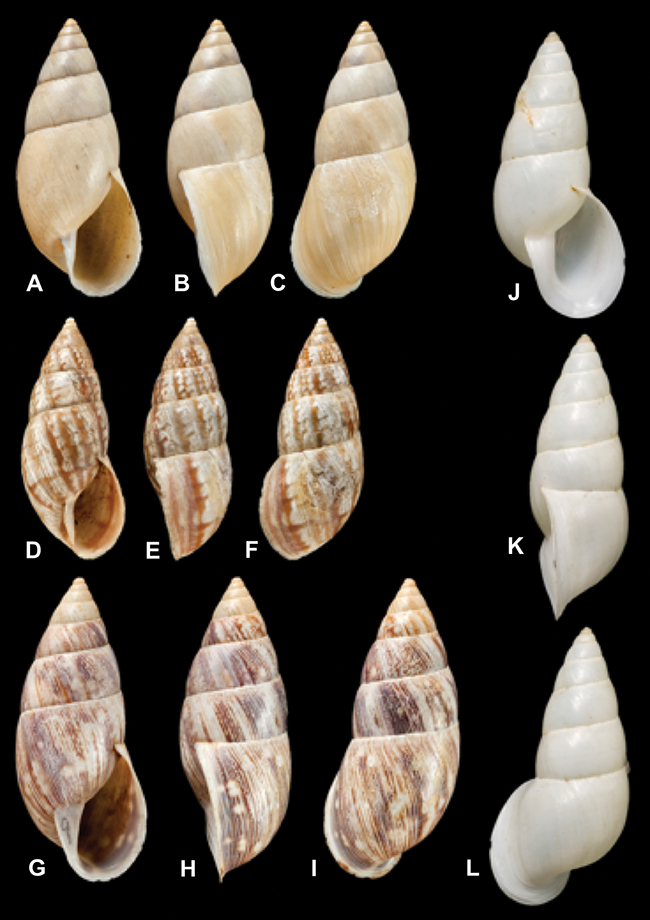
*Drymaeus* species. **A–C** *Drymaeus (Drymaeus) cuzcoensis* (Reeve, 1849), lectotype NHMUK 1975453 (H = 36.6) **D–F** *Drymaeus (Drymaeus) clathratus* (Pfeiffer, 1858), lectotype NHMUK 1975449 (H = 30.0) **G–I** *Drymaeus (Drymaeus) praetextus* (Reeve, 1849), lectotype NHMUK 198340 (H = 39.0) **J–L** *Drymaeus (Drymaeus) alabastrinus* da Costa, 1906, holotype NHMUK 1907.11.21.16 (H = 33.0). All enlarged.

**Figure 44.**
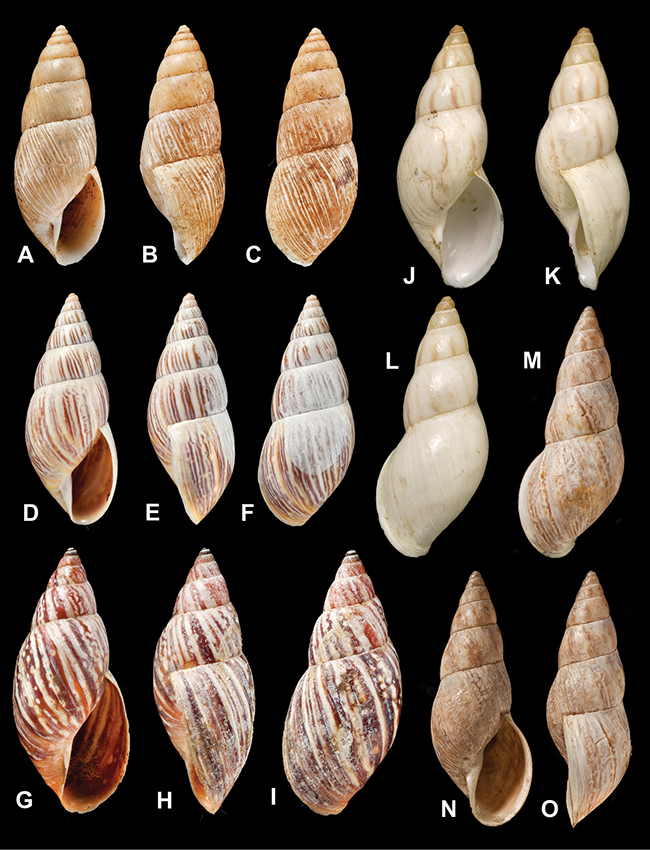
*Drymaeus* species. **A–C** *Drymaeus (Drymaeus) cylindricus* da Costa, 1901, lectotype NHMUK 1907.11.21.42 (H = 30.6) **D–F** *Drymaeus (Drymaeus) scitulus* (Reeve, 1849), lectotype NHMUK 1975217 (H = 29.5) **G–I** *Drymaeus (Drymaeus) vespertinus* (Pfeiffer, 1858), lectotype NHMUK 1975471 (H = 34.6) **J–L** *Drymaeus (Drymaeus) pseudofusoides* da Costa, 1906, holotype NHMUK 1907.11.21.11 (H = 33.6) **M–O** *Drymaeus (Drymaeus) lophoicus* (d’Orbigny, 1835), paralectotype NHMUK 1854.12.4.135 (H = 32.7). All enlarged.

**Figure 45.**
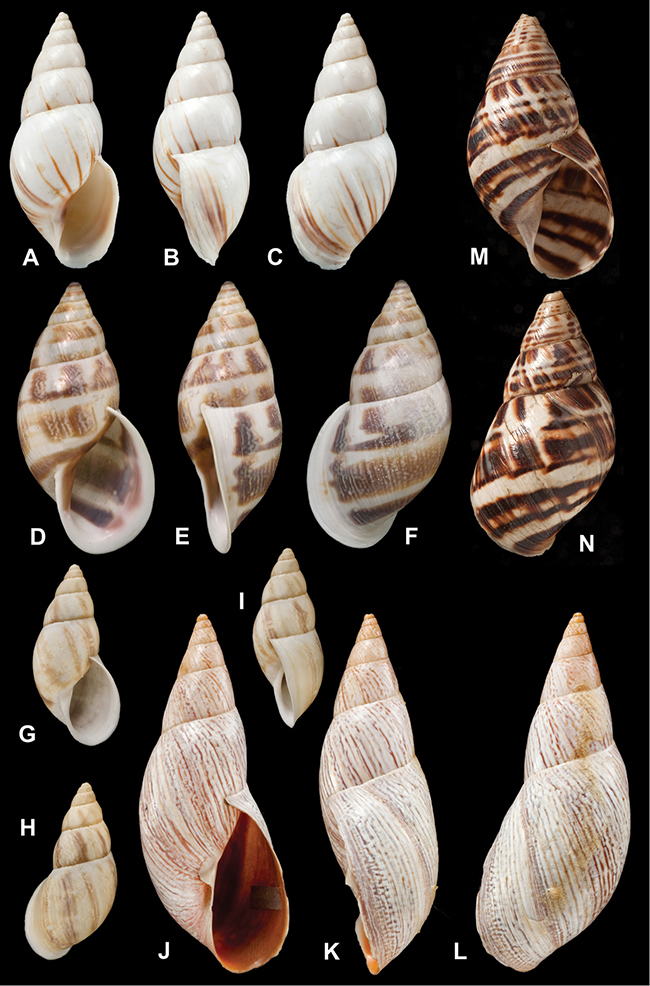
*Drymaeus* species. **A–C** *Drymaeus (Drymaeus) acuminatus* da Costa, 1906, holotype NHMUK 1907.11.21.10 (H = 33.4) **D–F** *Drymaeus (Drymaeus) felix* (Pfeiffer, 1862), lectotype NHMUK 1975206 (H = 34.0) **G–I** *Drymaeus (Drymaeus) fucatus* (Reeve, 1849), lectotype NHMUK 1874.12.11.224 (H = 23.5) **J–L** *Drymaeus (Drymaeus) farrisi* (Pfeiffer, 1858), lectotype NHMUK 1975506 (H = 46.8) **M–N** *Drymaeus (Drymaeus) poecilus* (d’Orbigny, 1835), paralectotype NHMUK 1854.12.4.155 (H = 34.8). All enlarged.

**Figure 46.**
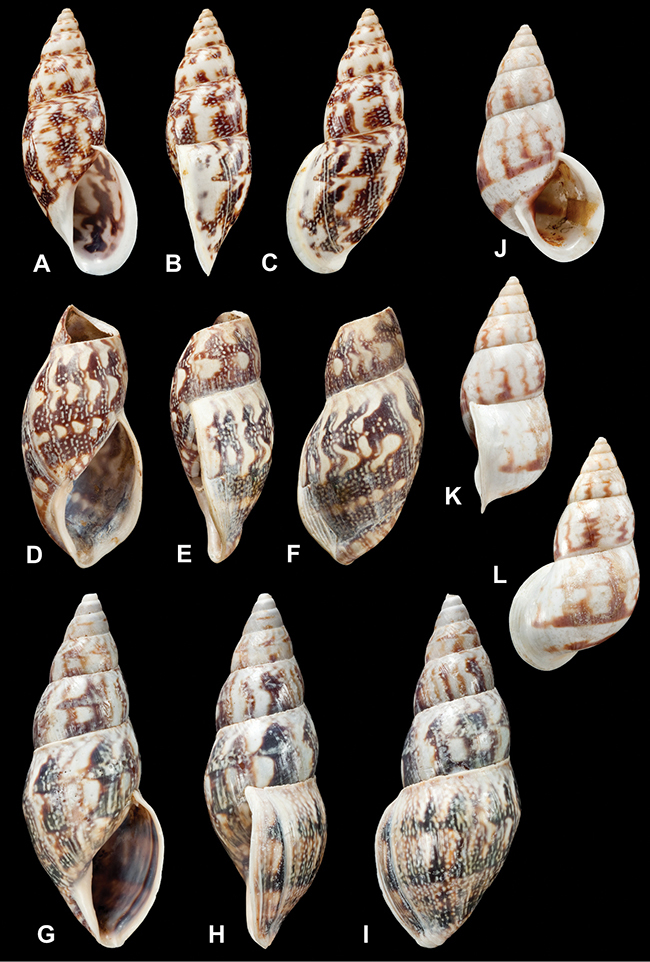
*Drymaeus* species. **A–C** *Drymaeus (Drymaeus) punctatus* da Costa, 1907, lectotype NHMUK 1907.11.21.20 (H = 36.6) **D–F** *Drymaeus (Drymaeus) pulcherrimus* (H. Adams, 1867), holotype NHMUK 1867.5.18.3 (H = 39.1) **G–I** *Drymaeus (Drymaeus) subhybridus* (da Costa, 1906), holotype NHMUK 1907.11.21.127 (H = 49.1) **J–L** *Drymaeus (Drymaeus) geometricus* (Pfeiffer, 1846), lectotype NHMUK 1975564 (H = 34.6). All enlarged.

**Figure 47.**
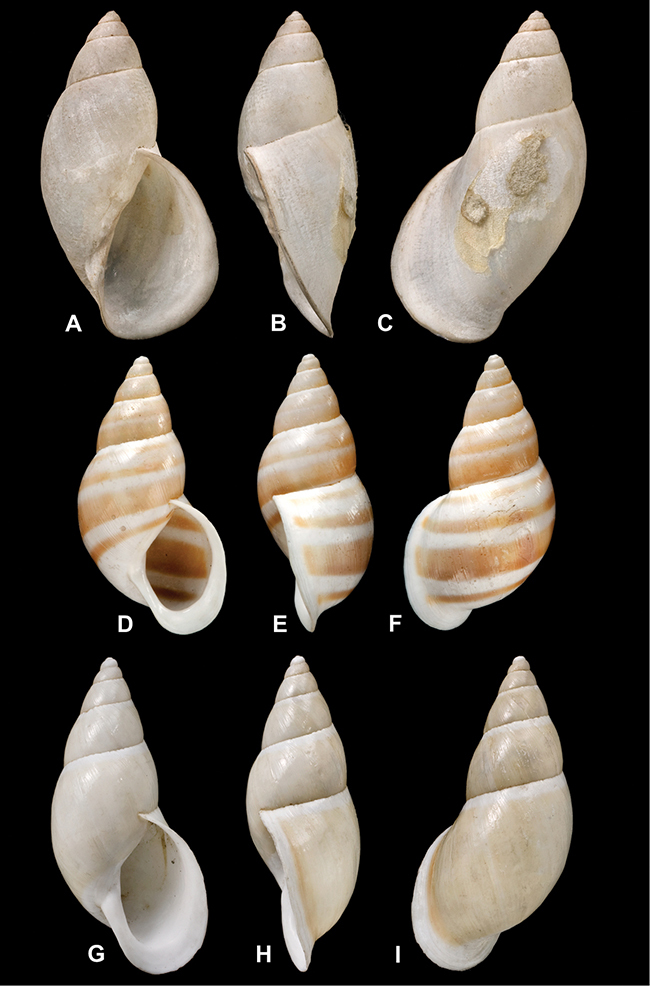
*Drymaeus* species. **A–C** *Drymaeus (Drymaeus) inclinatus* (Pfeiffer, 1862), lectotype NHMUK 1975532 (H = 33.1) **D–I** *Drymaeus (Drymaeus) quadrifasciatus* (Angas, 1878) **D–F** lectotype NHMUK 1879.1.21.3 (H = 28.9) **G–I** lectotype of *Bulimus (Otostomus) napo* Angas, 1878 NHMUK 1879.1.21.4 (H = 31.3). All enlarged.

**Figure 48.**
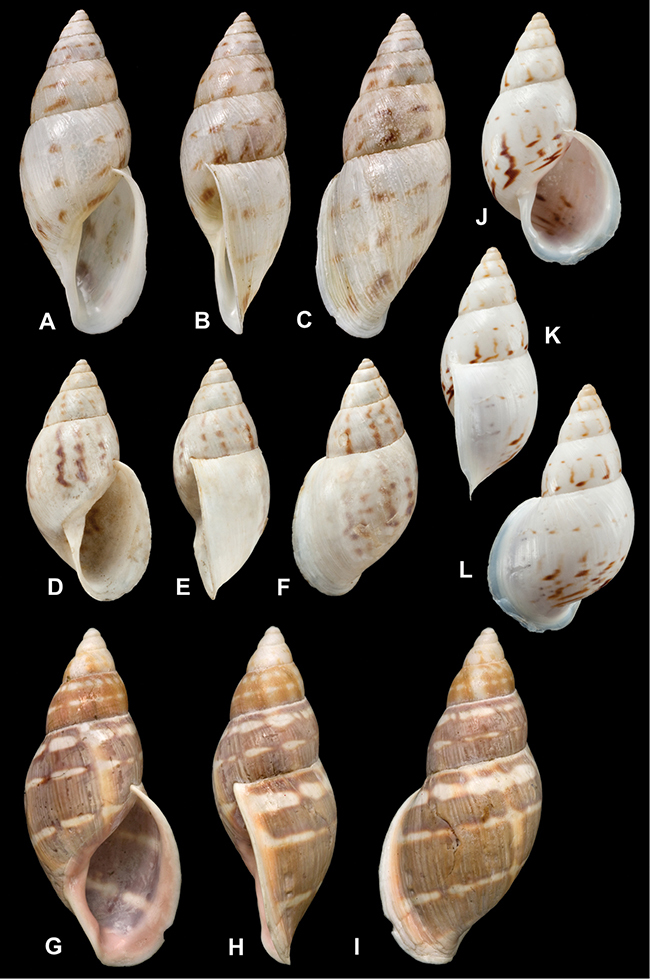
*Drymaeus* species. **A–C** *Drymaeus (Drymaeus) ochrocheilus* (E.A. Smith, 1877), lectotype NHMUK 1877.3.28.4 (H = 37.1) **D–F** *Drymaeus (Drymaeus) recedens* (Pfeiffer, 1864), lectotype NHMUK 1975477 (H = 27.3) **G–I** *Drymaeus (Drymaeus) spectatus* (Reeve, 1849), lectotype NHMUK 1874.12.11.226 (H = 39.2) **J–L** *Drymaeus (Drymaeus) regularis* Fulton, 1905, lectotype NHMUK 1905.11.17.2 (H = 29.6). All enlarged.

**Figure 49.**
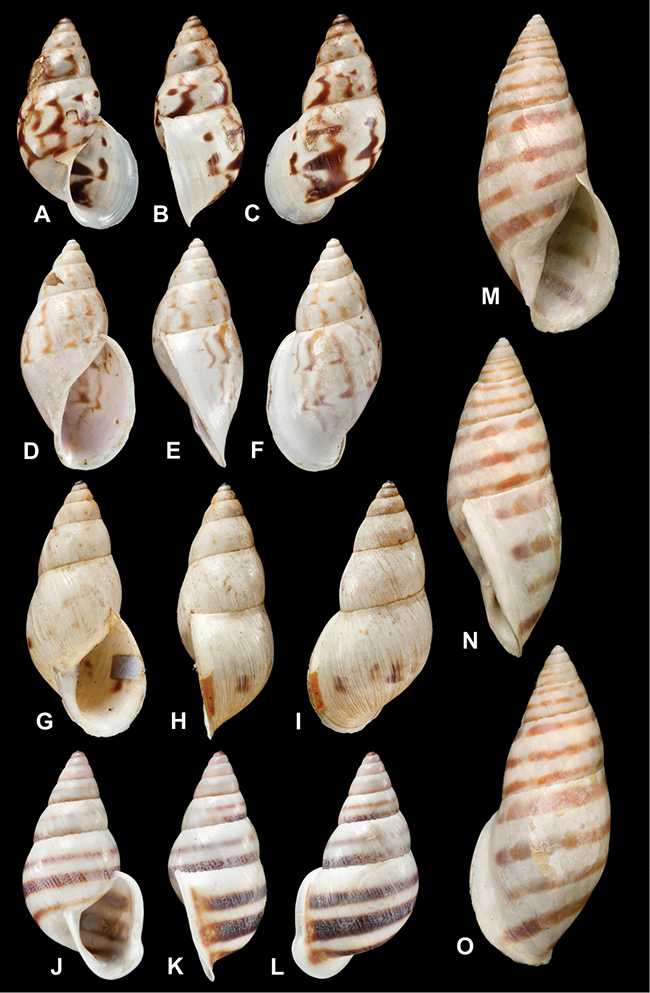
*Drymaeus* species. **A–C** *Drymaeus (Drymaeus) selli* (Preston, 1909), lectotype NHMUK 1915.1.6.36 (H = 24.0) **D–F** *Drymaeus (Drymaeus) serratus* (Pfeiffer, 1855), lectotype NHMUK 1975475 (H = 26.3) **G–I** *Drymaeus (Drymaeus) protractus* (Pfeiffer, 1855), lectotype NHMUK 1975494 (H = 29.3) **J–L** *Drymaeus (Drymaeus) bivittatus* (Sowerby I, 1833), lectotype of *Bulimus flexilabris* Pfeiffer, 1853 NHMUK 1975559 (H = 27.1) **M–O** *Drymaeus (Drymaeus) subinterruptus* (Pfeiffer, 1853), lectotype NHMUK 1975470 (H = 36.7). All enlarged.

**Figure 50.**
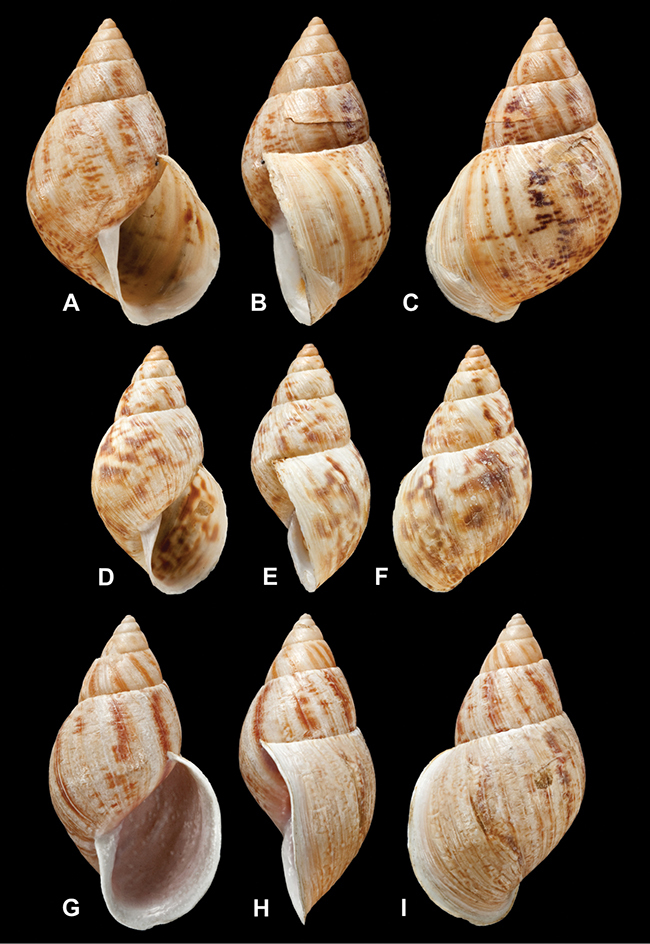
*Drymaeus* species. **A–C** *Drymaeus (Drymaeus) acervatus* (Pfeiffer, 1857), lectotype NHMUK 1975461 (H = 41.5) **D–F** *Drymaeus (Drymaeus) dunkeri* (Pfeiffer in Philippi 1846), lectotype NHMUK 1975512 (H = 34.8) **G–I** *Drymaeus (Drymaeus) lattrei* (Pfeiffer in Philippi 1846), lectotype NHMUK 1975555 (H = 42.6). All enlarged.

**Figure 51.**
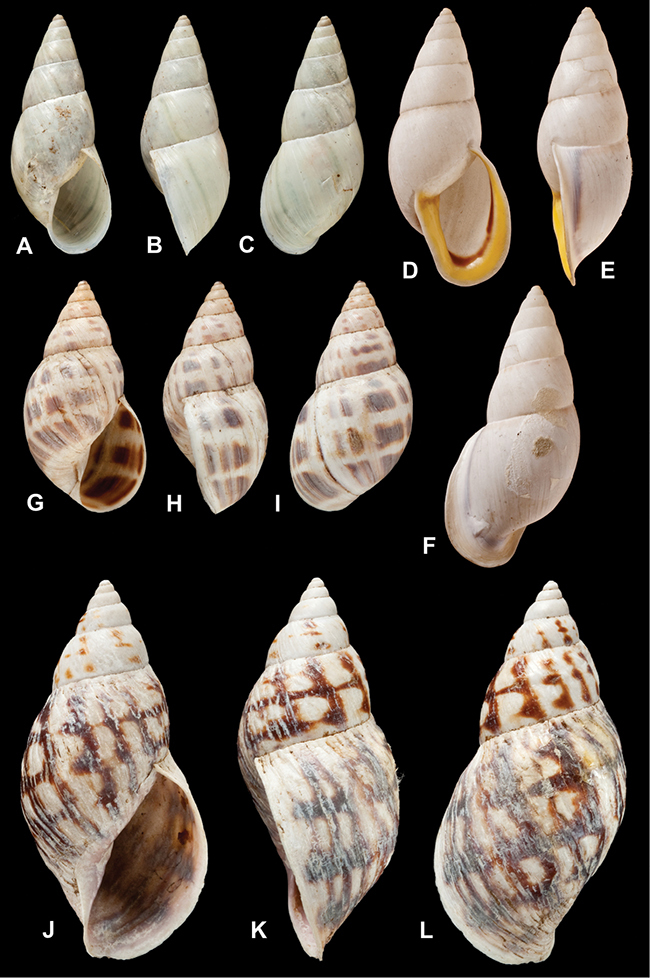
*Drymaeus* species. **A–C** *Drymaeus (Drymaeus) expatriatus* (Preston, 1909), lectotype NHMUK 1975201 (H = 28.1) **D–F** *Drymaeus (Drymaeus) trigonostomus* (Jonas, 1844), possible syntype of *Bulimus knorri* Pfeiffer, in Philippi, 1846 NHMUK 20100654 (H = 34.1) **G–I** *Drymaeus (Drymaeus) fenestrellus* (Martens, 1864), lectotype of *Bulimus (Mesembrinus) geali* H. Adams, 1867 NHMUK 1867.1.9.20 (H = 26.4) **J–L** *Drymaeus (Drymaeus) fenestratus* (Pfeiffer, 1846), lectotype NHMUK 1975525 (H = 44.0). All enlarged.

**Figure 52.**
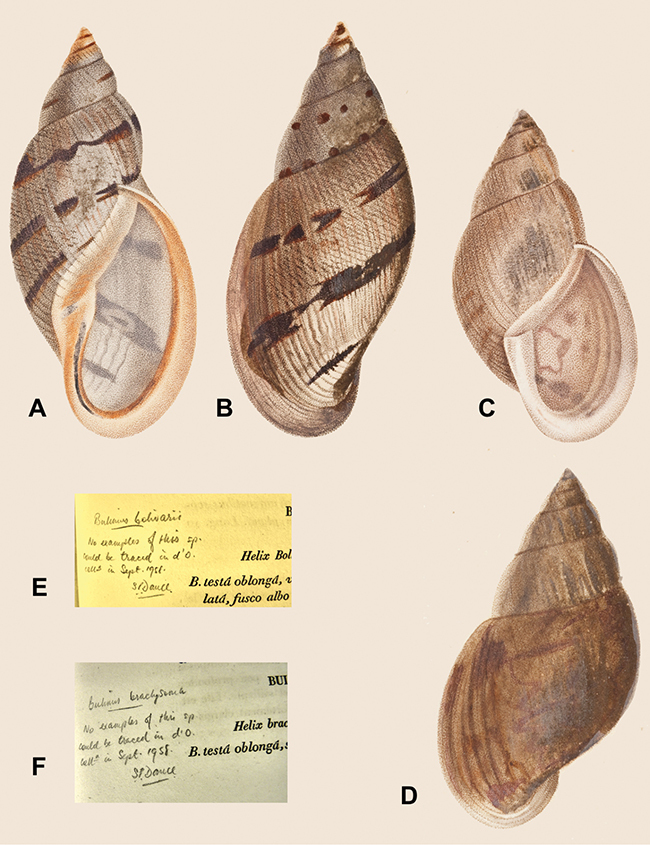
*Drymaeus* species. **A–B** *Drymaeus (Drymaeus) bolivarii* (d’Orbigny, 1835), d’Orbigny 1837 [1834–1847]: pl. 39 figs 5–6 **C–D** *Drymaeus (Drymaeus) brachysoma* (d’Orbigny, 1835), d’Orbigny 1837 [1834–1847]: pl. 39 figs 9–10 **E–F** Remarks of S.P. Dance in the margin of the text.

**Figure 53.**
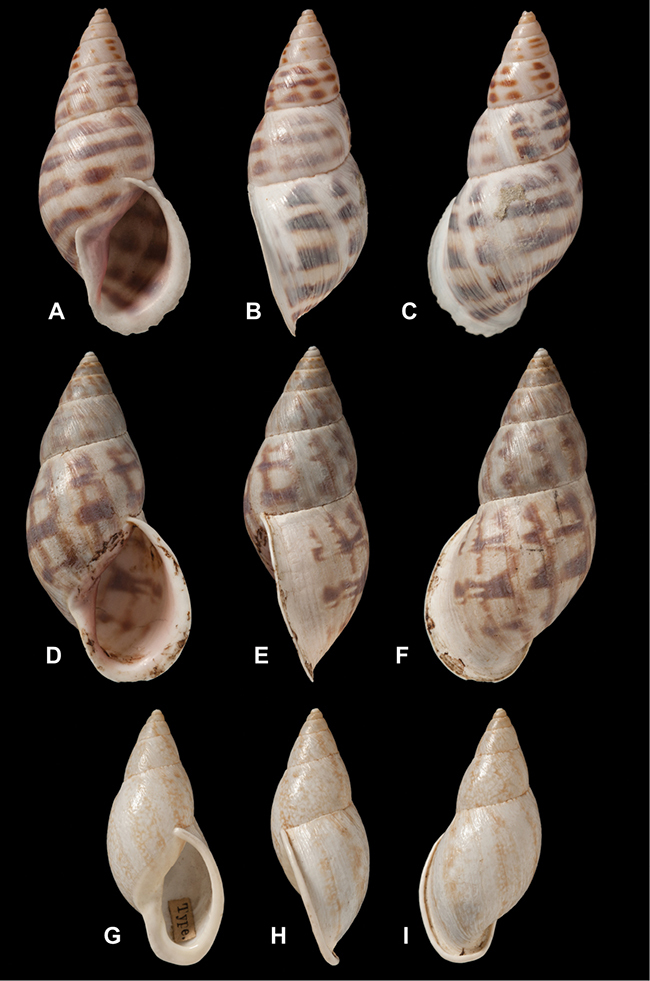
*Drymaeus* species. **A–C** *Drymaeus (Drymaeus) lilacinus lilacinus* (Reeve, 1849), probably syntype NHMUK 20100520 (H = 45.5) **D–F** *Drymaeus (Drymaeus) lilacinus jansoni* (Martens, 1893), lectotype NHMUK 1901.6.22.951 (H = 45.7) **G–I** *Drymaeus (Drymaeus) malleatus* (da Costa, 1898), holotype NHMUK 1907.11.21.130 (H = 34.5). All enlarged.

**Figure 54.**
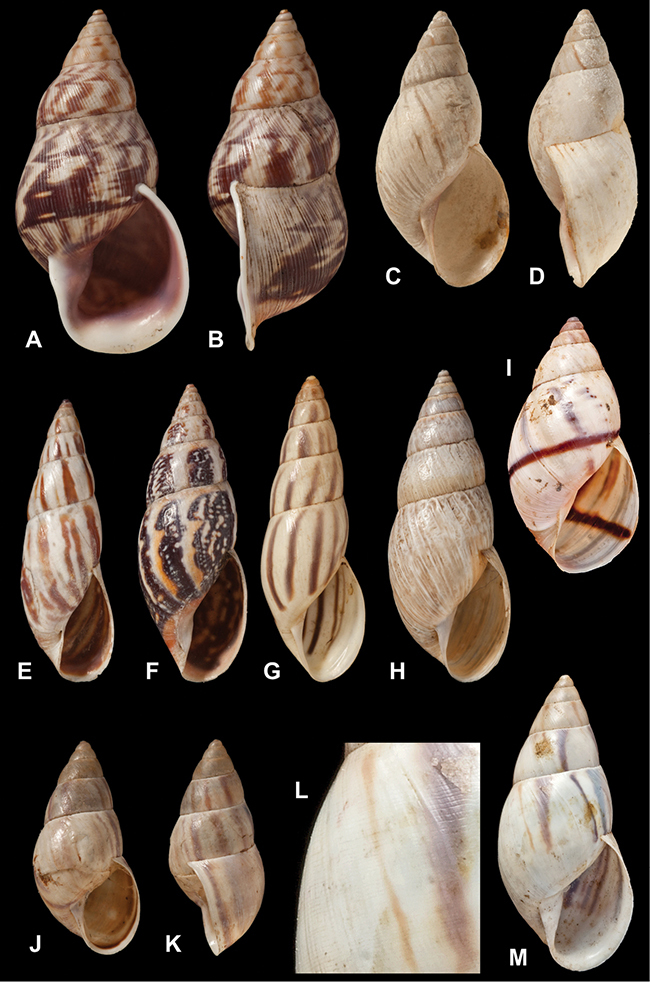
*Drymaeus* species. **A–B** *Drymaeus (Drymaeus) expansus perenensis* da Costa, 1901, lectotype NHMUK 1907.11.21.39 (H = 45.8) **C–D** *Drymaeus (Mesembrinus) ghiesbreghti iodostylus* (Pfeiffer, 1861), syntype NHMUK 1975568 (H = 32.2) **E** *Drymaeus (Drymaeus) trujillensis* (Philippi, 1867), possible syntype of *Bulimus (Otostomus) lamas* Higgins, 1868 NHMUK 1868.4.3.3 (H = 29.2) **F** *Drymaeus (Drymaeus) rubrovariegatus* (Higgins, 1868), possible syntype NHMUK 1868.4.3.1 (H = 32.8) **G** *Drymaeus (Drymaeus) pergracilis* (Rolle, 1904), lectotype NHMUK 1922.2.3.33 (H = 33.2) **H** *Drymaeus (Drymaeus) ponsonbyi* da Costa, 1907, holotype NHMUK 1907.11.21.27 (H = 33.5) **I** *Drymaeus (Mesembrinus) moritinctus* (Martens, 1893), lectotype NHMUK 1901.6.22.841 (H = 25.2) **J–K** *Drymaeus (Mesembrinus) recluzianus* (Pfeiffer, 1847), lectotype of *Bulimulus (Drymaeus) nubilus* Preston, 1903 NHMUK 1903.5.4.1 (H = 22.3) **L–M** *Drymaeus (Mesembrinus) oreades* (d’Orbigny, 1835) **L** sculpture on ultimate whorl **M** lectotype NHMUK 1854.121.4.161 (H = 31.6). All enlarged.

**Figure 55.**
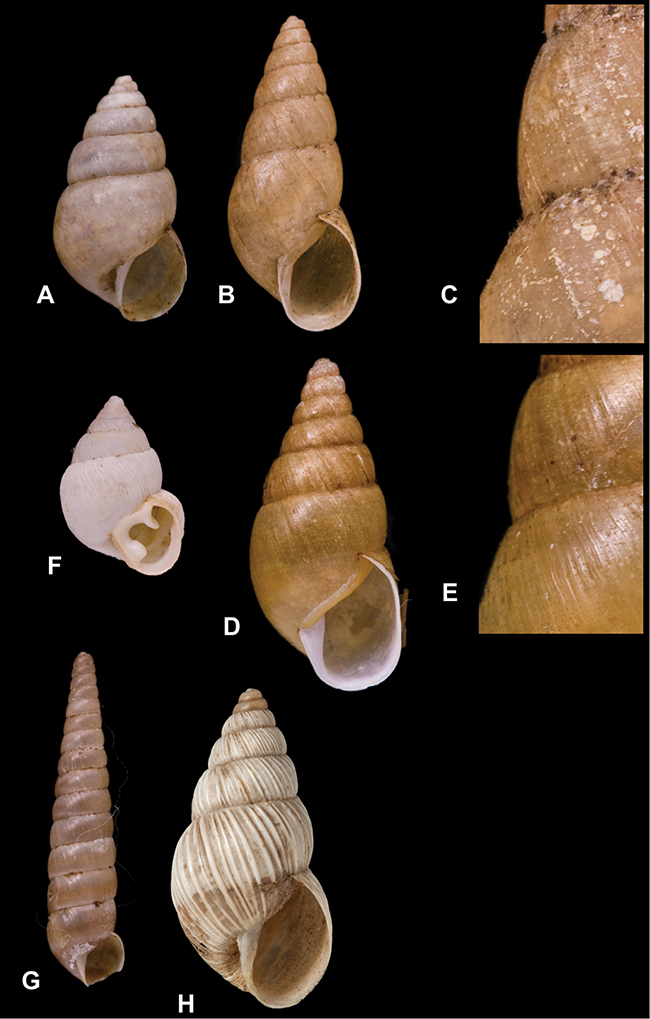
**A** *Protoglyptus pilosus* (Guppy, 1871), syntype NHMUK 1875.2.8.3 (H = 14.95) **B–C** *Protoglyptus martinicensis* (Pfeiffer, 1846), lectotype NHMUK 197451 (H = 19.5) **C** sculpture on dorsal side of (pen)ultimate whorl **D–E** *Protoglyptus sanctaeluciae* (E.A. Smith, 1889), lectotype NHMUK 1889.3.23.12 (H = 20.7) **E** sculpture on dorsal side of (pen)ultimate whorl **F** *Naesiotus lycodes* (Dall, 1917), possible paratype NHMUK 1937.6.18.11 (H = 10.0) **G** *Naesiotus habeli* (‘Stearns’ Dall, 1892), possible syntype of *Bulimulus (Pleuropyrgus) terebra* Reibisch, 1892 NHMUK 1894.6.8.2 (H = 19.1) **H** *Naesiotus orbignyi* (Pfeiffer, 1846), lectotype NHMUK 1975349 (H = 18.5). All enlarged.

**Figure 56.**
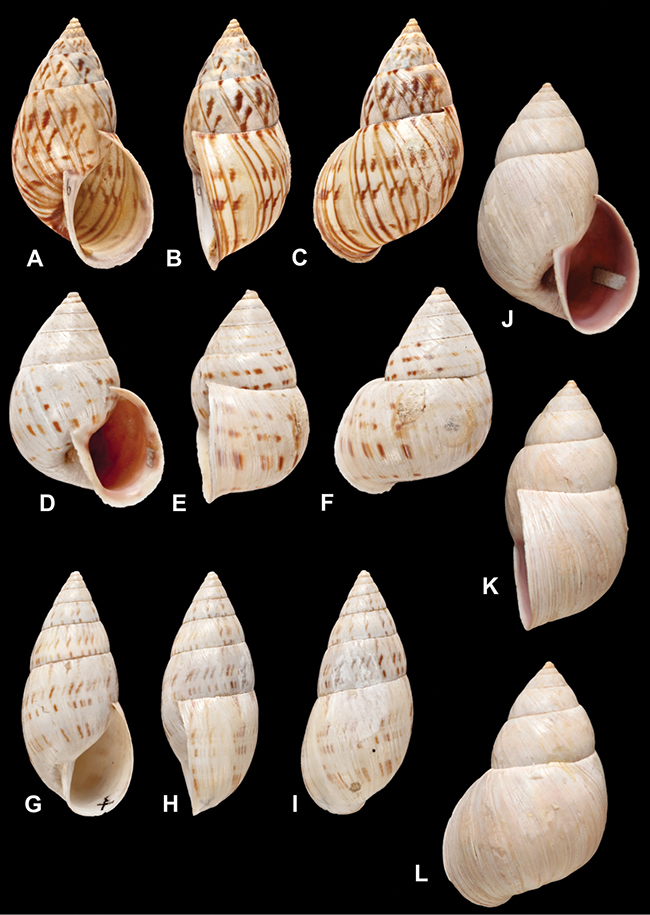
*Neopetraeus* species. **A–C** *Neopetraeus altoperuvianus* (Reeve, 1849), lectotype NHMUK 1975437 (H = 46.5) **D–F** *Neopetraeus atahualpa* (Dohrn, 1863), possible syntype NHMUK 1975436 (H = 38.5) **G–I** *Neopetraeus decussatus* (Reeve, 1849), lectotype NHMUK 1975180 (H = 37.7) **J–L** *Neopetraeus patasensis* (Pfeiffer, 1858), lectotype NHMUK 1975439 (H = 46.8). All enlarged.

**Figure 57.**
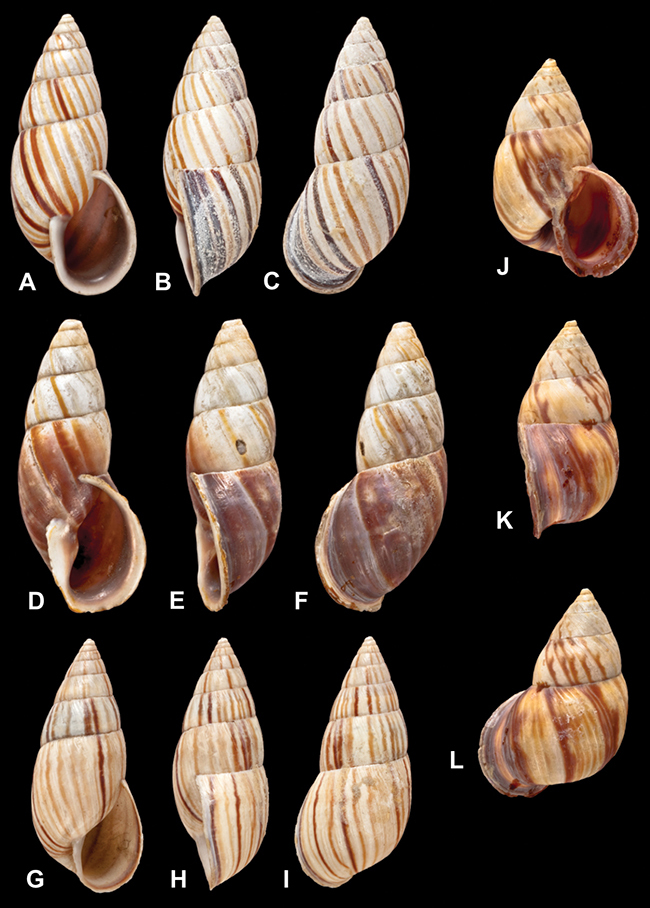
*Neopetraeus* species. **A–F** *Neopetraeus lobbii* (Reeve, 1849) **A–C** lectotype NHMUK 1975431 (H = 44.3) **D–F** lectotype of *Bulimus ptychostylus* Pfeiffer, 1858 NHMUK 1975430 (H = 46.7) **G–I** *Neopetraeus decussatus* (Reeve, 1849), lectotype of *Bulimus myristicus* Reeve, 1849 NHMUK 1975433 (H = 40.3) **J–L** *Neopetraeus platystomus* (Pfeiffer, 1858), lectotype NHMUK 1975428 (H = 34.4). All enlarged.

**Figure 58.**
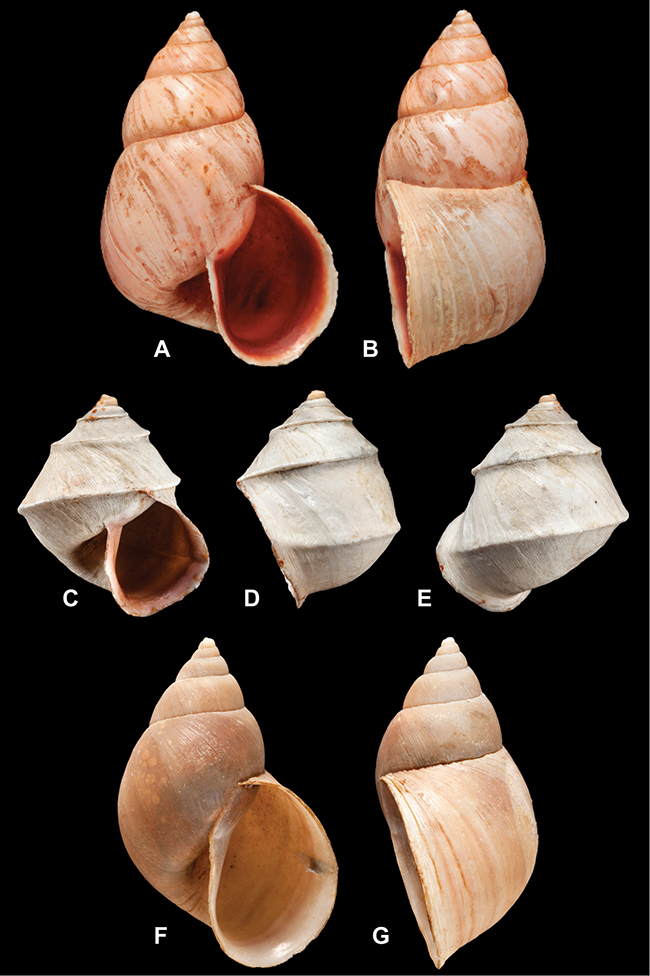
*Neopetraeus* species. **A–B** *Neopetraeus cora* (d’Orbigny, 1835), lectotype NHMUK 1854.12.4.124 (H = 43.9) **C–E** *Neopetraeus binneyanus* (Pfeiffer, 1857), lectotype NHMUK 1975426 (H = 25.2) **F–G** *Neopetraeus excoriatus* (Pfeiffer, 1855), lectotype NHMUK 1975500 (H = 38.4). All enlarged.

**Figure 59.**
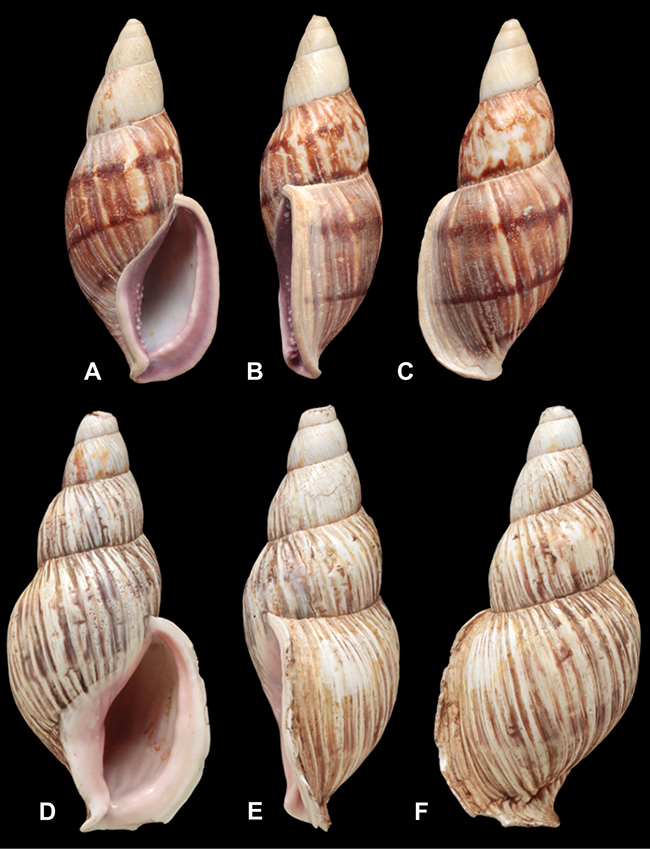
*Newboldius crichtoni* (Broderip, 1836) **A–C** Holotype of *Bulimus* (?) *illustris* Rolle, 1905 NHMUK 1922.2.24.40 (H = 61.2) **D–F** Holotype NHMUK 1958.9.3.4 (H = 71.3). All enlarged.

**Figure 60.**
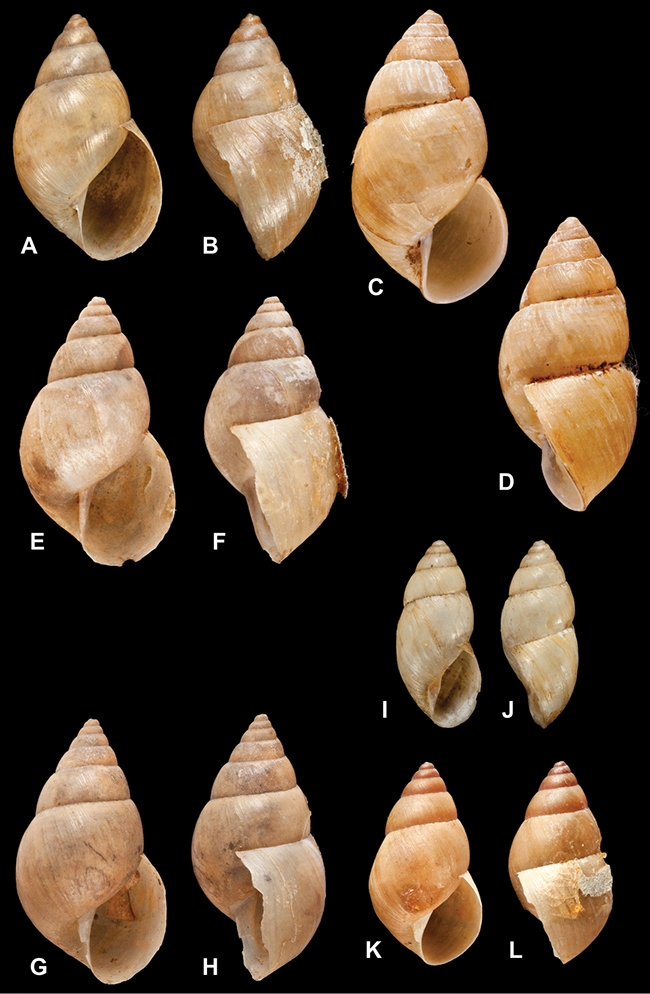
*Bulimulus* species. **A–B** *Bulimulus fuscus* (Guilding, 1828), lectotype of *Bulimus barbadensis* Pfeiffer, 1853 NHMUK 1974054 (H = 21.3) **C–D** *Bulimulus* (?) *dukinfieldi* Melvill, 1900 [nomen inquirendum], lectotype NHM 1900.9.27.2 (H = 28.1) **E–F** *Bulimulus cacticolus* (Reeve, 1849), syntype NHMUK 20100515 (H = 24.4) **G–H** *Bulimulus erectus* (Reeve, 1849), syntype NHMUK 20100516 (H = 22.0) **I–J** *Bulimulus mollicellus* (Reeve, 1849), lectotype NHMUK 1975185 (H = 17.2) **K–L** *Bulimulus coriaceus* (Pfeiffer, 1857), syntype NHMUK 1975409 (H = 17.1). All enlarged.

**Figure 61.**
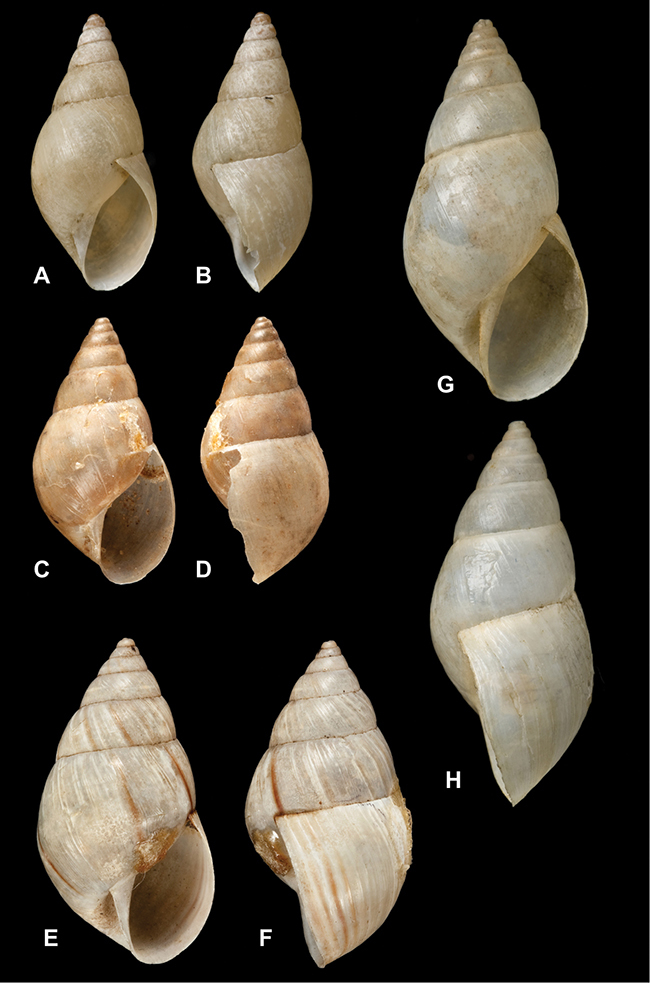
*Bulimulus* species. **A–B** *Bulimulus juvenilis* (Pfeiffer, 1855), lectotype NHMUK 1975161 (H = 19.5) **C–D** *Bulimulus tenuissimus* (d’Orbigny, 1835), syntype NHMUK 1854.12.4.163 (H = 19.1) **E–F** *Bulimulus pliculatus* (Pfeiffer, 1857), lectotype NHMUK 1975390 (H = 23.0) **G–H** *Bulimulus haplochrous* (Pfeiffer, 1855), lectotype NHMUK 1975405 (H = 28.6). All enlarged.

**Figure 62.**
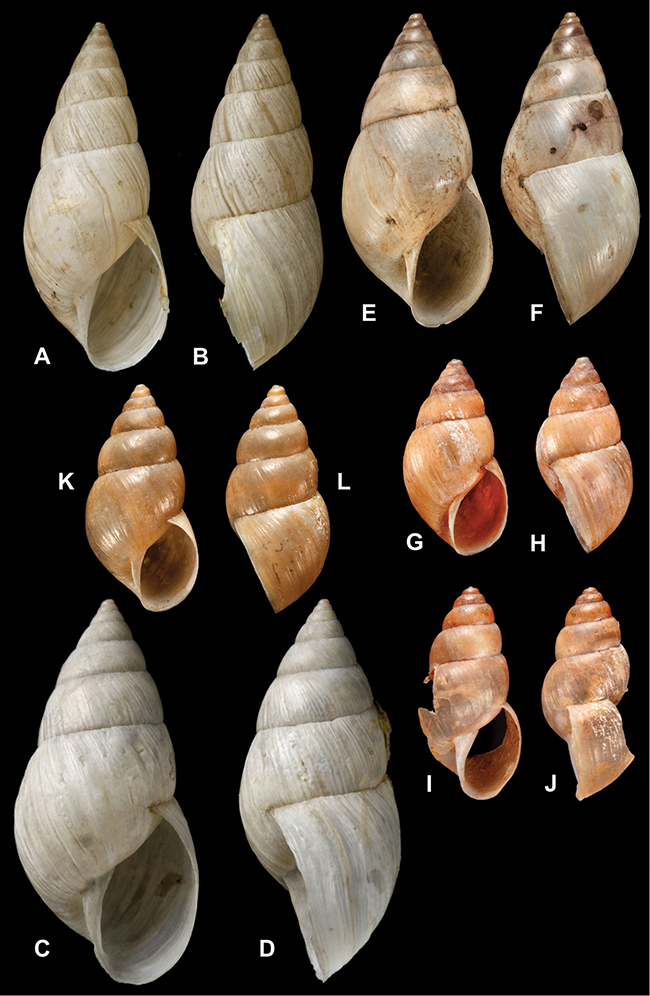
*Bulimulus* species. **A–B** *Bulimulus effeminatus* (Reeve, 1848), lectotype NHMUK 1975508 (H = 31.1) **C–D** *Bulimulus gelidus* (Reeve, 1849)[nomen inquirendum], lectotype NHMUK 1975402 (H = 32.5) **E–F** *Bulimulus heloicus* (d’Orbigny, 1835), syntype NHMUK 1854.12.4.164 (H = 25.6) **G–H** *Bulimulus inutilis* (Reeve, 1850), lectotype NHMUK 1975162 (H = 16.5) **I–J** *Bulimulus unicolor* (Sowerby I, 1833), lectotype of *Bulimus ignavus* Reeve, 1849 (H = 17.1) **K–L** *Bulimulus dysoni* (Pfeiffer, 1846), syntype NHMUK 1975453 (H = 19.2). All enlarged.

**Figure 63.**
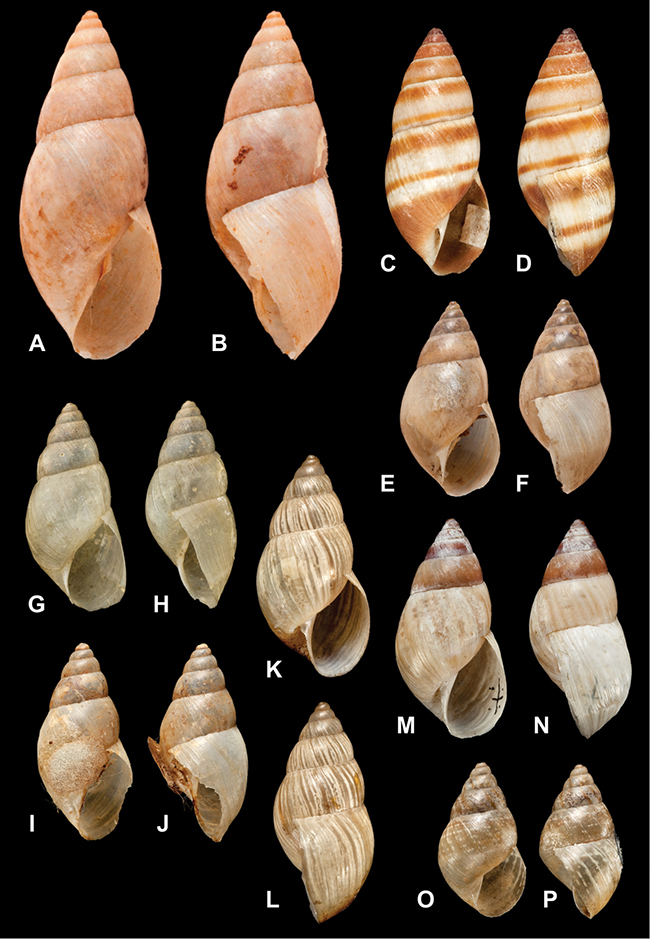
*Bulimulus* species. **A–B** *Bulimulus monachus* (Pfeiffer, 1857), lectotype NHMUK 20100584 (H = 31.2) **C–D** *Bulimulus guadalupensis* (Bruguière, 1789), lectotype of *Bulimus rubrifasciatus* Reeve, 1848 NHM 1975393 (H = 22.9) **E–F** *Bulimulus corneous* (Sowerby I, 1833), lectotype of *Bulimus nubeculatus* Pfeiffer, 1853 NHMUK 1975407 (H = 16.0) **G–H** *Bulimulus transparens* (Reeve, 1849), lectotype NHMUK 1975397 (H = 18.4) **I–J** *Bulimulus marcidus* (Pfeiffer, 1853), possible syntype NHMUK 20100649 (H = 18.0) **K–L** *Bulimulus turritellatus* (Beck, 1838), paralectotype of *Helix turritella* d’Orbigny 1835 NHMUK 1854.12.4.166 (H = 19.3) **M–P** *Bulimulus unicolor* (Sowerby I, 1833) **M–N** lectotype of *Bulimus petenensis* Morelet, 1851 NHMUK 1893.2.4.1176 (H = 18.7) **O–P** Possible syntype of *Bulimulus istapensis* Crosse and Fischer, 1873 NHMUK 1893.2.4.1185 (H = 11.2). All enlarged.

**Figure 64.**
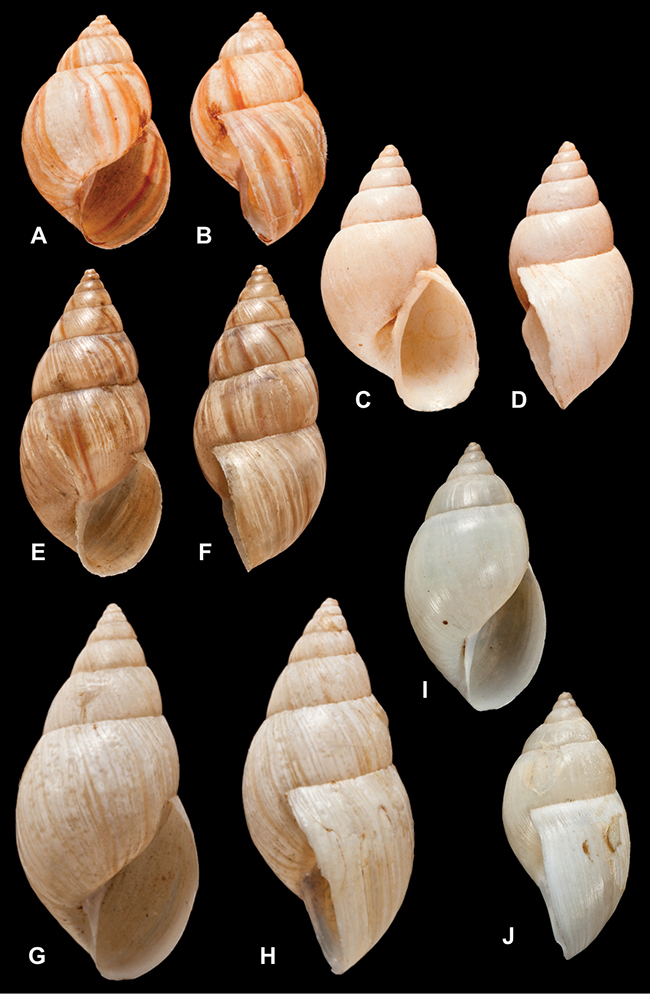
**A–B** *Bostryx apodemetes* (d’Orbigny, 1835), lectotype of *Bulimus pessulatus* Reeve, 1848 NHMUK 1975313 (H = 21.6). **C–J** *Bulimulus* species. **C–D** *Bulimulus pervius* (Pfeiffer, 1853), lectotype NHMUK 1975165 (H = 24.3) **E–H** *Bulimulus bonariensis* (Rafinesque, 1833) **E–F** *Bulimulus bonariensis sporadicus* (d’Orbigny, 1835), lectotype NHMUK 1854.12.4.160 (H = 27.8) **G–H** *Bulimulus bonariensis bonariensis* (Rafinesque, 1833), syntype of *Bulimus montevidensis* Pfeiffer, 1846 NHMUK 1975401 (H = 34.2) **I–J** *Bulimulus vesicalis* (Pfeiffer, 1853), lectotype NHMUK 1975395 (H = 24.8). All enlarged.

**Figure 65.**
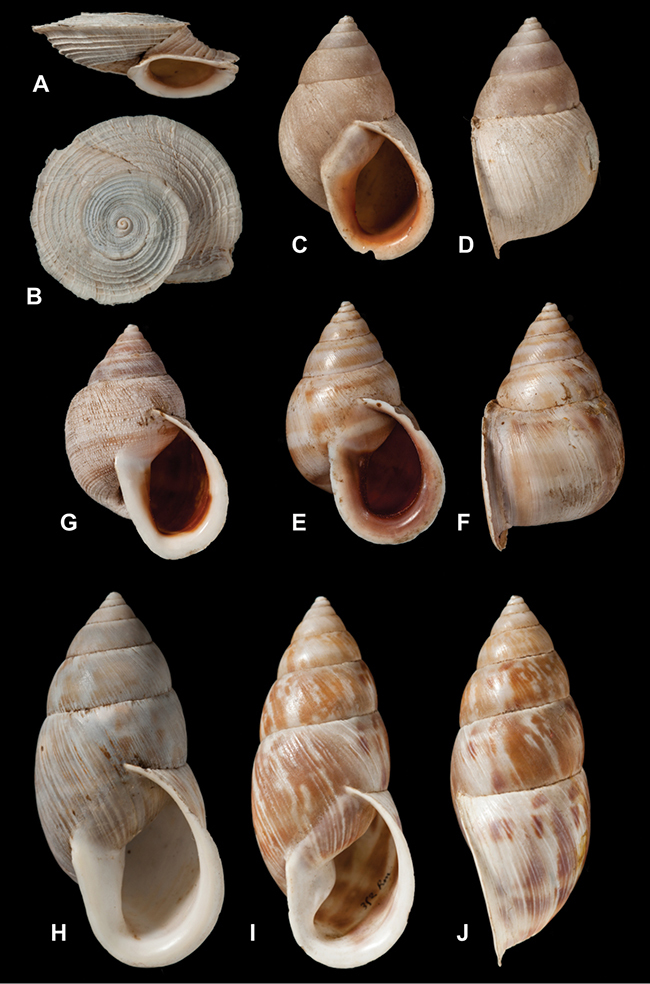
*Scutalus* species. **A–B** *Scutalus baroni* (Fulton, 1896), lectotype NHMUK 1896.6.23.1 (H = 12.7) **C–F** *Scutalus cretaceus* (Pfeiffer, 1855) **C–D** lectotype NHMUK 1975388 (H = 35.2) **E–F** lectotype of *Bulimulus (Drymaeus) baroni* Fulton, 1897 NHMUK 1897.8.3.173 (H = 34.3) **G** *Scutalus chiletensis* Weyrauch, 1967, paratype NHMUK 1975384 (H = 34.2) **H** *Scutalus grandiventris* Weyrauch, 1960, paratype NHMUK 1975386 (H = 54.1) **I–J** *Scutalus latecolumellaris* Preston, 1909, lectotype NHMUK 1922.2.24.39 (H = 54.0). All enlarged.

**Figure 66.**
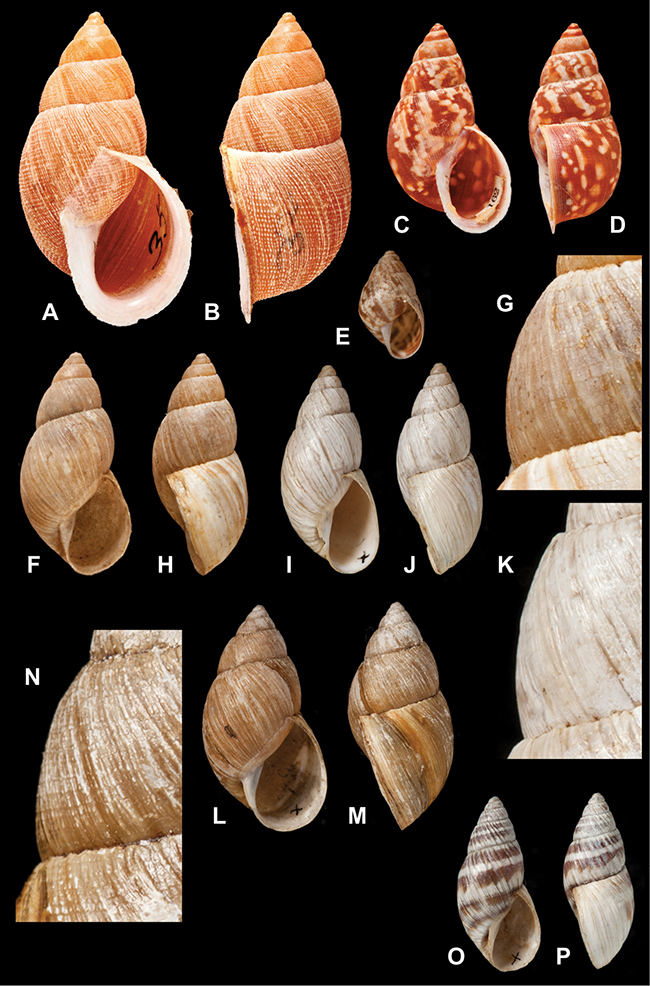
**A–D** *Scutalus* species. **A–B** *Scutalus proteus* Broderip in Broderip and Sowerby I 1832, lectotype NHMUK 20100638 (H = 44.1) **C–E** *Scutalus versicolor versicolor* (Broderip in Broderip and Sowerby I 1832) **C–D** possible syntype NHMUK 20100637 (H = 29.6) **E** lectotype of *Bulimus coagulatus* Reeve 1849 NHMUK 1975351 (H = 15.6) **F–P** *Kuschelenia* species. **F–N** *Kuschelenia (Kuschelenia) culminea culminea* (d’Orbigny, 1835) **F–H** paralectotype NHMUK 1854.12.4.198 (H = 31.2) **I–K** lectotype of *Bulimus confusus* Reeve, 1848 NHMUK 1975194 (H = 29.0) **L–N** lectotype of *Bulimus jussieui* Pfeiffer, 1846 NHMUK 1975170 (H = 31.6) **O–P** *Kuschelenia (Kuschelenia) bicolor* (Sowerby I, 1835), lectotype NHMUK 1975151 (H = 23.6). All enlarged.

**Figure 67.**
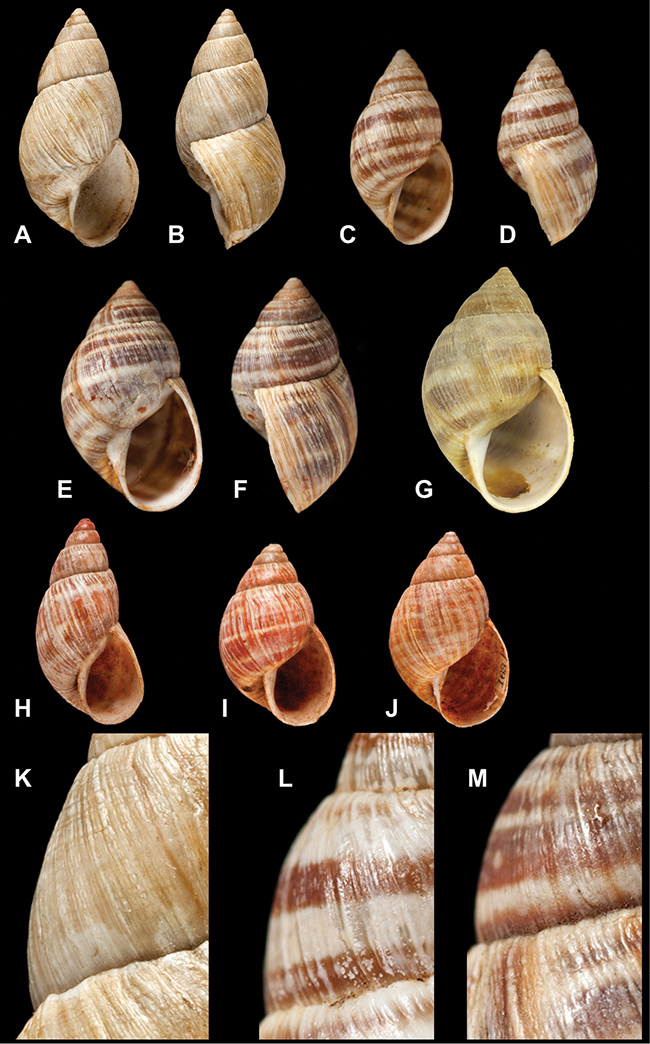
*Kuschelenia* species. **A–B, K** *Kuschelenia (Kuschelenia) culminea culminea* (d’Orbigny, 1835), paralectotype of *Helix lithoica* d’Orbigny, 1835 NHMUK 1854.12.4.197 (H = 34.3) **C–D, L** *Kuschelenia (Kuschelenia) culminea edwardsi* (Morelet, 1863), syntype NHMUK 1893.2.4.177 (H = 25.4) **E–G, M** *Kuschelenia (Kuschelenia) gayi* (Pfeiffer, 1857) **E–F** lectotype NHMUK 1975382 (H = 27.7) **G** syntype of *Helix thamnoica* var. C d’Orbigny, 1835 NHMUK 1854.12.4.110 (H = 32.2) **H–J** *Kuschelenia (Kuschelenia) bicolor* (Sowerby I, 1835) **H–I** lectotype resp. paralectotype of *Helix polymorpha* d’Orbigny, 1835 NHMUK 1854.12.4.199 (H = 23.6 resp. 21.2) **J** possible syntype of *Bulinus badius* Sowerby I 1835 NHMUK 20100631 (H = 25.8). All enlarged.

**Figure 68.**
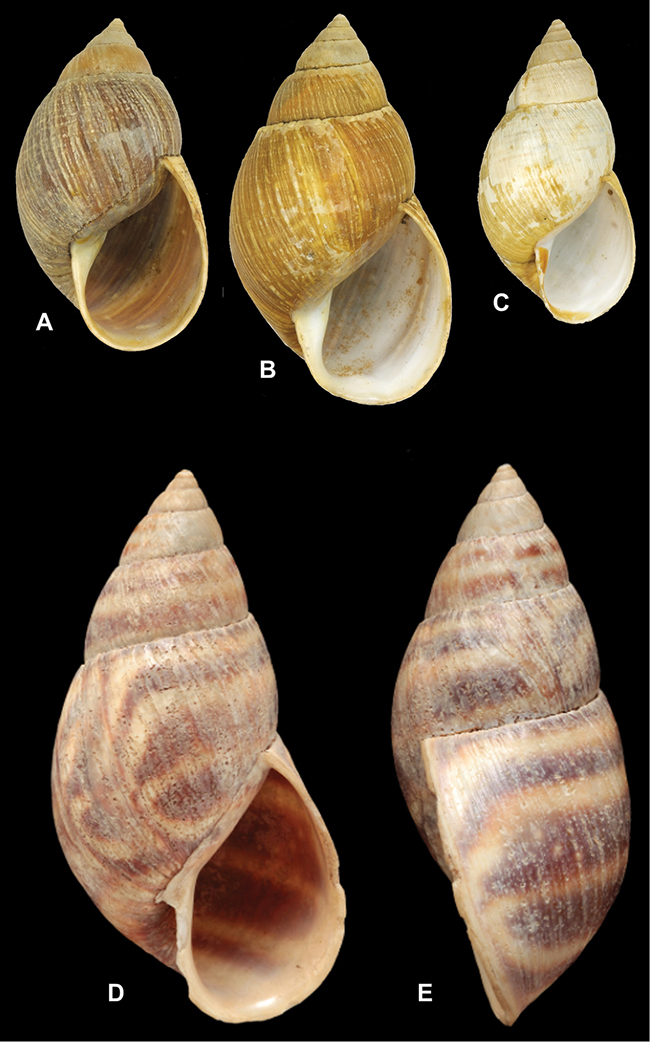
*Kuschelenia* species. **A** *Kuschelenia (Kuschelenia) thamnoica* (d’Orbigny, 1835), paralectotype NHMUK 1854.12.4.111 (H = 44.0) **B–C** *Kuschelenia (Kuschelenia) alauda* Hupé, 1857 **B** paralectotype of *Helix thamnoica* var. B d’Orbigny 1835 NHMUK 1854.12.4.109 (H = 52.7) **C** lectotype of *Helix thamnoica* var. D d’Orbigny 1835 NHMUK 1854.12.4.108 (H = 40.8) **D–E** *Kuschelenia (Kuschelenia) tupacii* (d’Orbigny, 1835), paralectotype NHMUK 1854.12.4.113 (H = 76.0). All enlarged.

**Figure 69.**
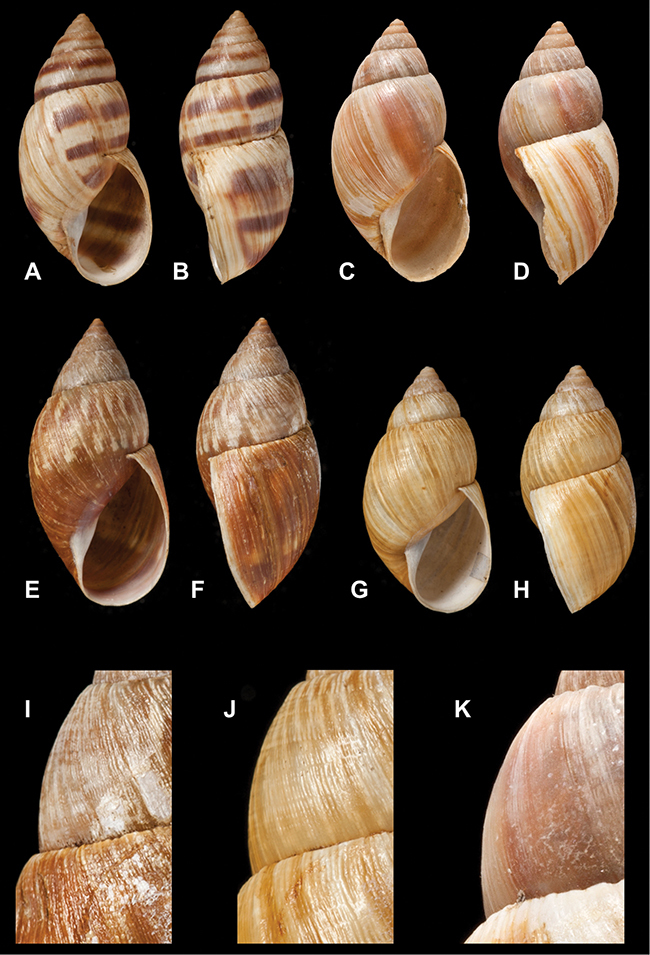
*Kuschelenia* species. **A–B** *Kuschelenia (Vermiculatus) aequatorius* Pfeiffer, 1853, lectotype NHMUK 1975377 (H = 37.8) **C–D, K** *Kuschelenia (Vermiculatus) caliginosus* (Reeve, 1849), lectotype NHMUK 20100518.1 (H = 35.4) **E–F, I** *Kuschelenia (Vermiculatus) anthisanensis* (Pfeiffer, 1853), lectotype NHMUK 1975372 (H = 40.2) **G–H, J** *Kuschelenia (Vermiculatus) cotopaxiensis* (Pfeiffer, 1853), lectotype NHMUK 1975370 (H = 33.9). All enlarged.

**Figure 70.**
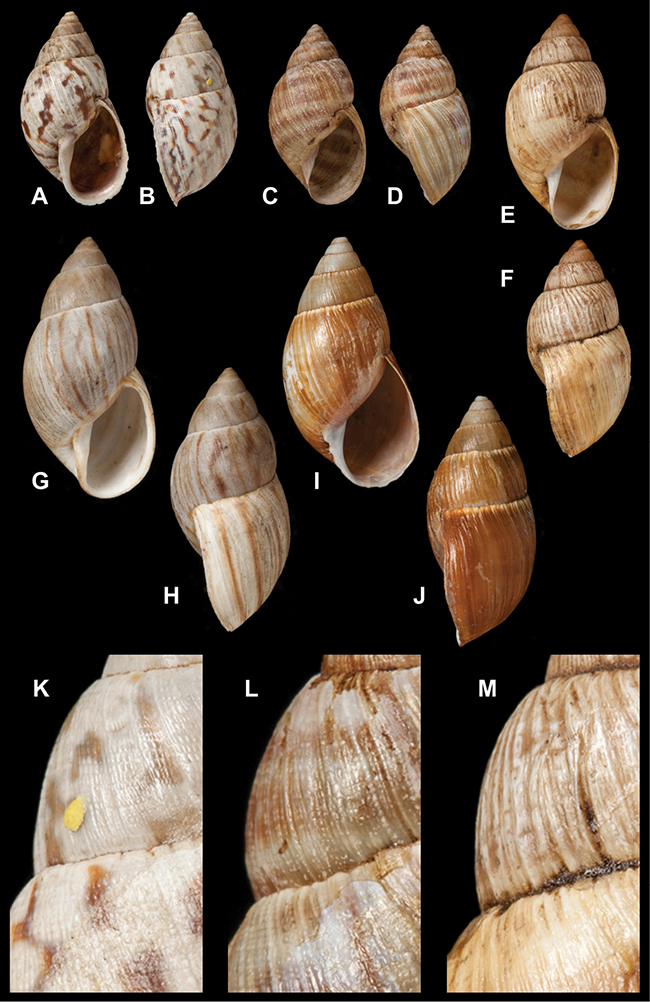
*Kuschelenia* species. **A–B, K** *Kuschelenia (Vermiculatus) aquilus* (Reeve, 1848), lectotype NHMUK 1975376 (H = 27.1) **C–D, L** *Kuschelenia (Vermiculatus) filaris* (Pfeiffer, 1853), syntype NHMUK 1975569 (H = 25.1) **E–F, M** *Kuschelenia (Vermiculatus) anthisanensis* (Pfeiffer, 1853), lectotype of *Bulimus subfasciatus* Pfeiffer, 1853 NHMUK 1975368 (H = 31.0) **G–H** *Kuschelenia (Vermiculatus) nucinus* (Reeve, 1850), lectotype NHMUK 1975379 (H = 36.8) **I–J** *Kuschelenia (Vermiculatus) petiti* (Pfeiffer, 1846), lectotype NHMUK 1975374 (H = 36.0). All enlarged

**Figure 71.**
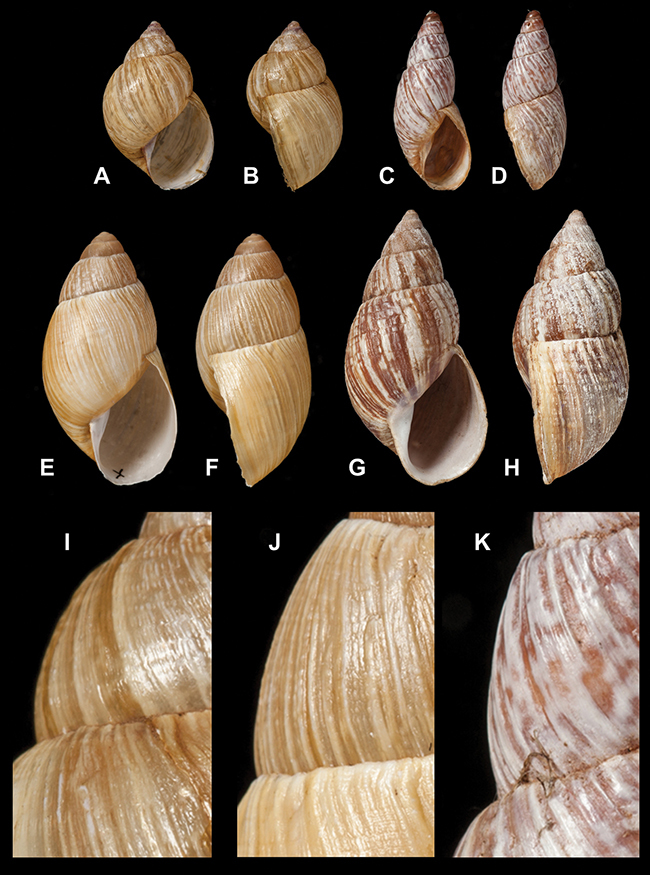
*Kuschelenia* species. **A–B, I** *Kuschelenia (Vermiculatus) peaki* (Breure, 1978), paratype NHMUK 1975579 (H = 23.5) **C–D, K** *Kuschelenia (Vermiculatus) quechuarum* (Crawford, 1939), holotype NHMUK 1939.4.17.226 (H = 21.5) **E–F, J** *Kuschelenia (Vermiculatus) ochracea* (Morelet, 1863), lectotype NHMUK 1893.2.4.164 (N = 36.0) **G–H** *Kuschelenia (Vermiculatus) purpuratus* (Reeve, 1849), lectotype NHMUK 1975364 (H = 37.3)

**Figure 72.**
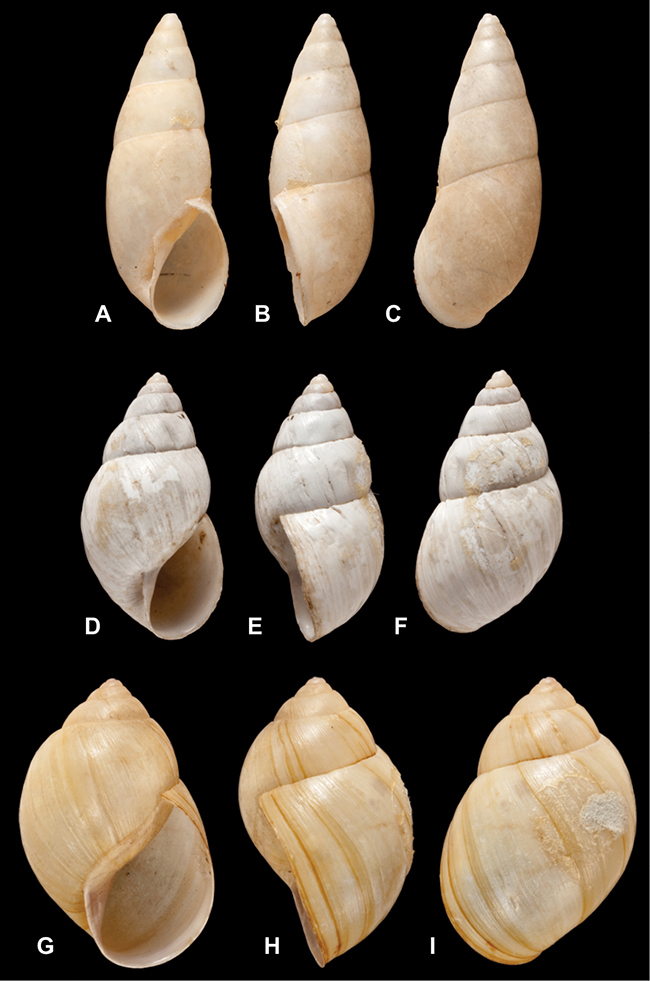
**A–C** *Naesiotus rimatus* (Pfeiffer, 1847), lectotype NHMUK 1975418 (H = 33.1) **D–I** *Rabdotus* species. **D–F** *Rabdotus schiedeanus schiedeanus* (Pfeiffer, 1841), probable syntype NHMUK 1975240 (H = 28.4) **G–I** *Rabdotus sufflatus* (Gould, 1859), syntype of *Bulimus juarezi* Pfeiffer, 1866 NHMUK 1975421 (H = 31.5). All enlarged.

**Figure 73.**
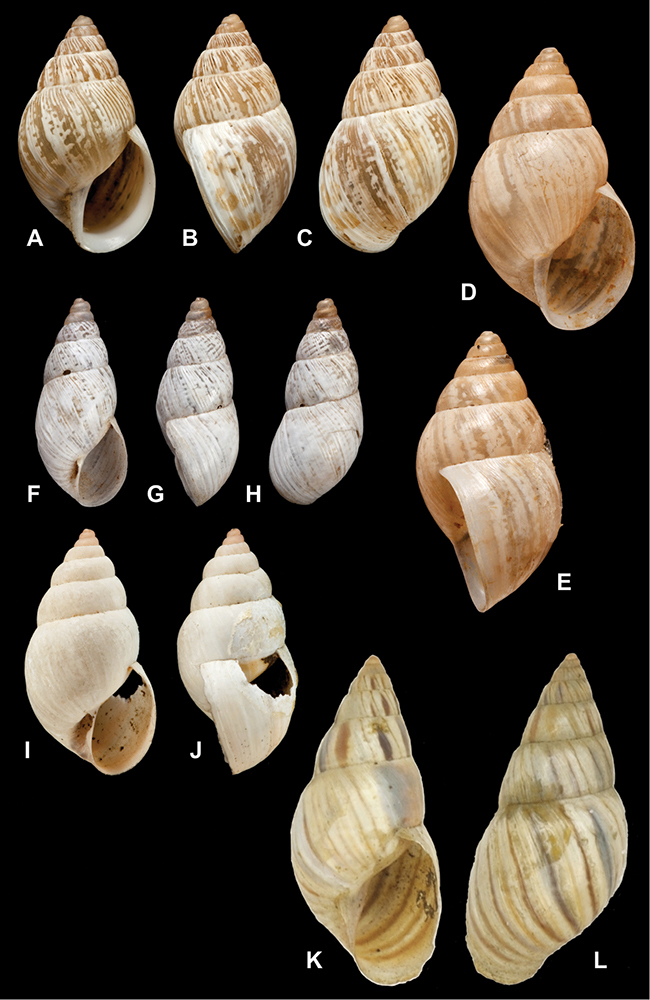
**A–E** *Rabdotus dealbatus* (Say, 1821). **A–C** paratype of *Bulimulus ragsdalei* Pilsbry 1890 NHMUK 1898.2.1.9 (H = 18.3) **D–E** lectotype of *Bulimus liquabilis* Reeve 1848 NHMUK 1975422 (H = 21.3) **F–H** *Naesiotus durangoanus* (Martens, 1893), holotype NHMUK 1901.6.22.871 (H = 15.0) **I–J** *Naesiotus* sp., possible syntype of *Bulimus clarus* Pfeiffer, 1857 [nomen inquirendum] NHMUK 1975487 (H = 15.9) **K–L** *Naesiotus pazianus* (d’Orbigny, 1835), paralectotype NHMUK 1854.12.4.196 (H = 24.3). All enlarged.

**Figure 74.**
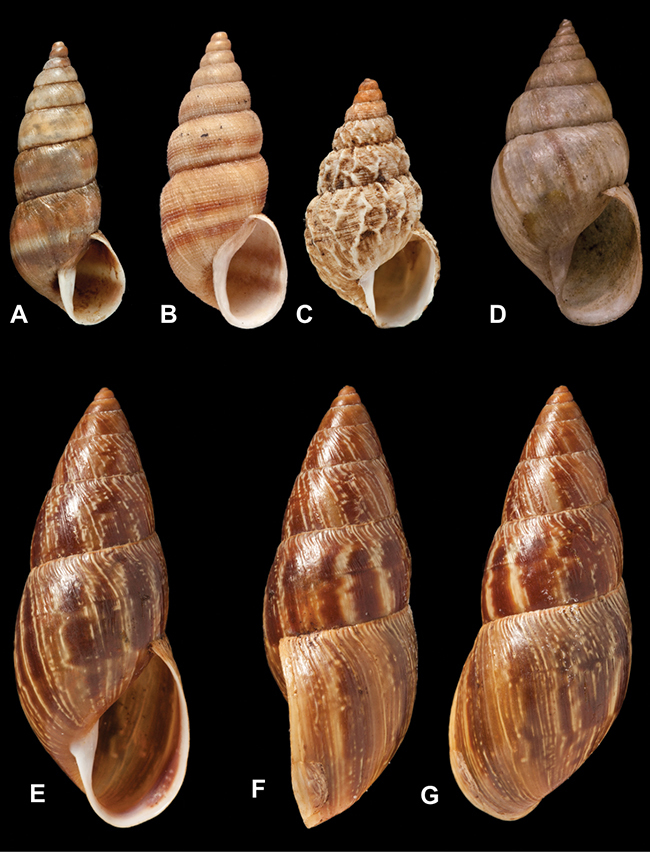
**A** *Naesiotus eschariferus* (Sowerby I, 1833), syntype NHMUK 1975153 (H = 18.4) **B** *Naesiotus rugulosus* (Sowerby I, 1838), lectotype NHMUK 1975176 (H = 20.5) **C** *Naesiotus sculpturatus* (Pfeiffer, 1846), lectotype NHMUK 1975174 (H = 14.1) **D** *Naesiotus munsterii* (d’Orbigny, 1837), syntype NHMUK 1854.12.4. (H = 21.7) **E–G** *Drymaeus (Drymaeus) laxostylus* (Rolle, 1904), holotype NHMUK 1922.2.23.32 (H = 39.9). All enlarged.

**Figure 75.**
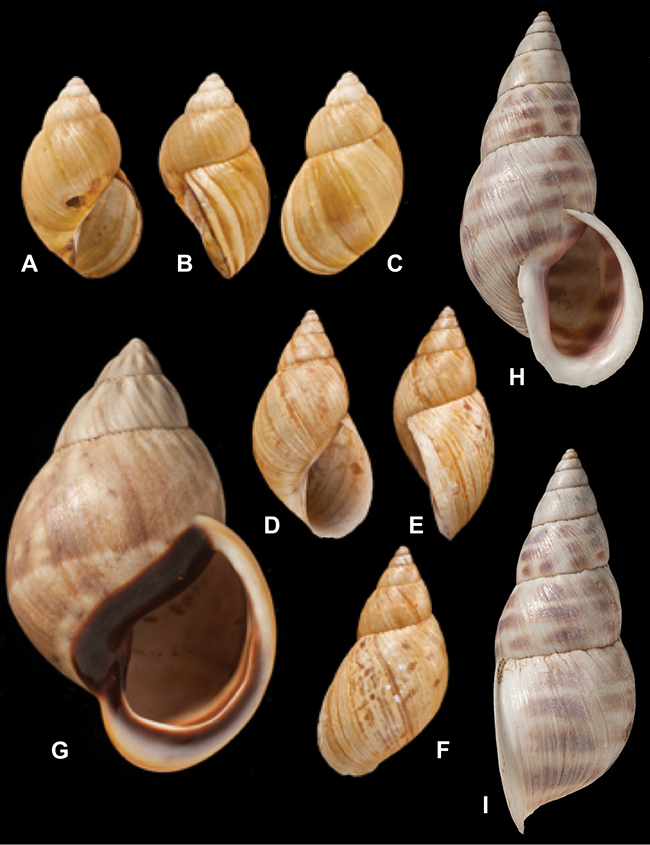
**A–C** *Stenostylus nigrolimbatus* (Pfeiffer, 1853), lectotype NHMUK 1975549 (H = 28.0) **D–F** *Stenostylus meleagris* (Pfeiffer, 1853), lectotype NHMUK 20100583 (H = 31.3) **G** *Auris chrysostoma* (Moricand, 1836), probable syntype of *Bulimus swainsoni* Pfeiffer 1845 NHMUK 20100586 (H = 60.0) **H–I** *Drymaeus (Drymaeus) lilacinus* (Reeve, 1849), lectotype of *Bulimus patricius* Reeve, 1849 NHMUK 1874.12.11.220 (H = 52.1). All enlarged.

**Figure L1.**
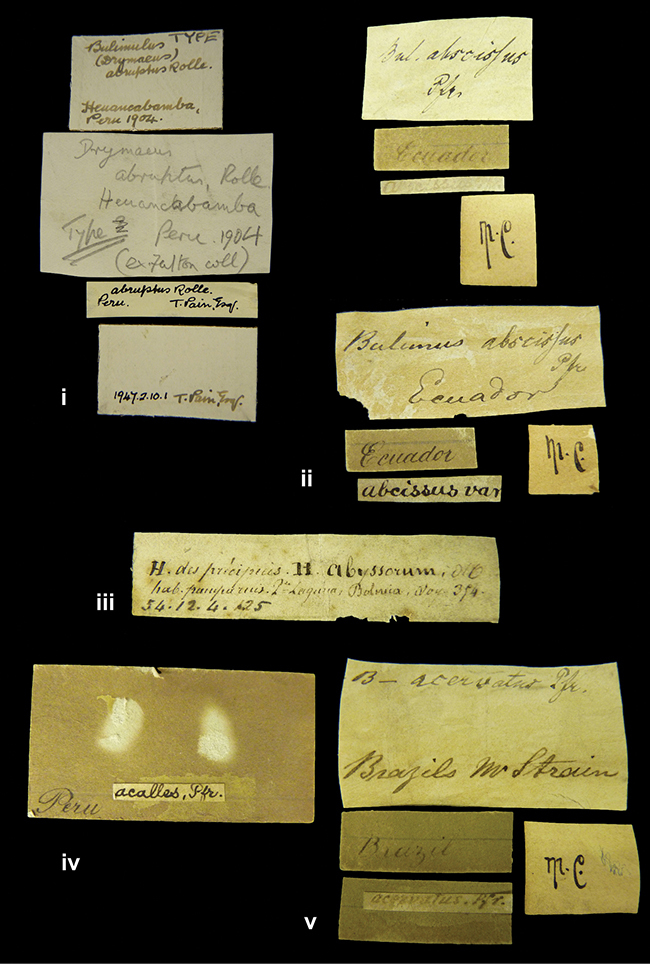
Labels. **i** *Bulimulus (Drymaeus) abruptus* Rolle, 1904 **ii** *Bulimus abscissus* Pfeiffer, 1855 **iii** *Helix abyssorum* d’Orbigny, 1835 **iv** *Bulimus acalles* Pfeiffer, 1853 **v** *Bulimus acervatus* Pfeiffer, 1857.

**Figure L2.**
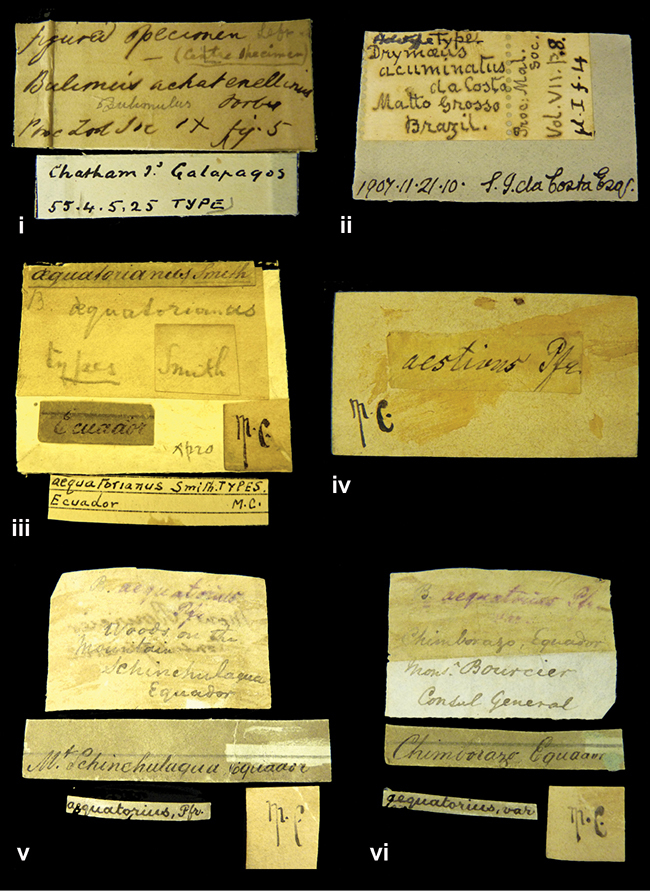
Labels. **i** *Bulimus achatellinus* Forbes, 1850 **ii** *Drymaeus acuminatus* da Costa, 1906 **iii** *Bulimus (Drymaeus) aequatorianus* E.A. Smith, 1877 **iv** *Bulimus aestivus* Pfeiffer, 1857 **v–vi** *Bulimus aequatorius* Pfeiffer, 1853.

**Figure L3.**
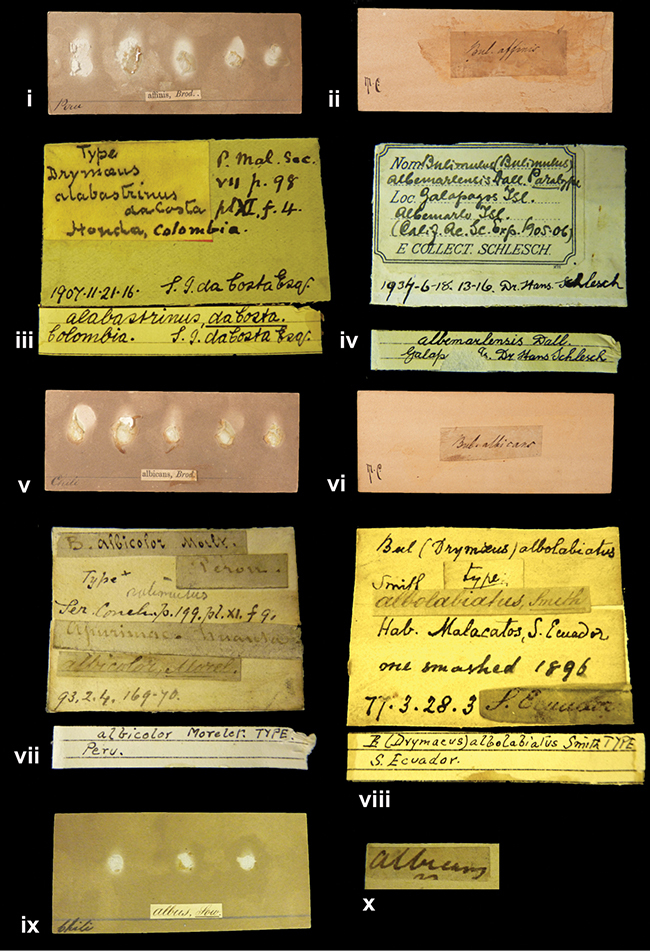
Labels. **i–ii** *Bulinus affinis* Broderip in Broderip and Sowerby I 1832 **iii** *Drymaeus alabastrinus* da Costa, 1906 **iv** *Bulimulus (Naesiotus) albemarlensis* Dall, 1917 **v–vi** *Bulinus albicans* Broderip in Broderip and Sowerby I 1832 **vii** *Bulimus albicolor* Morelet, 1863 **viii** *Bulimus (Drymaeus) albolabiatus* E.A. Smith, 1877 **ix–x** *Bulinus albus* Sowerby I, 1833.

**Figure L4.**
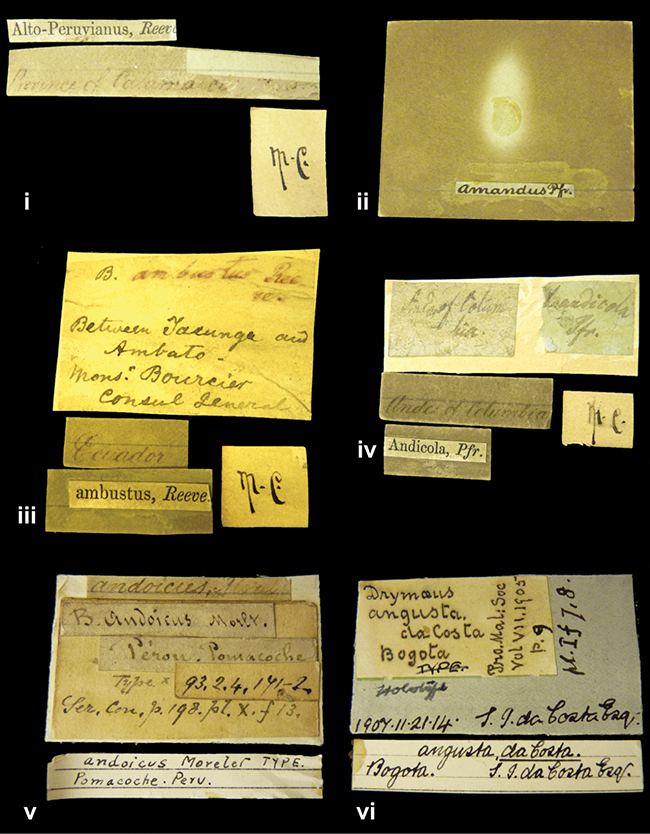
Labels. **i** *Bulimus altoperuvianus* Reeve, 1849 **ii** *Bulimus amandus* Pfeiffer, 1855 **iii** *Bulimus ambustus* Reeve, 1849 **iv** *Bulimus andicola* Pfeiffer, 1847 **v** *Bulimus andoicus* Morelet, 1863 **vi** *Drymaeus angustus* da Costa, 1906.

**Figure L5.**
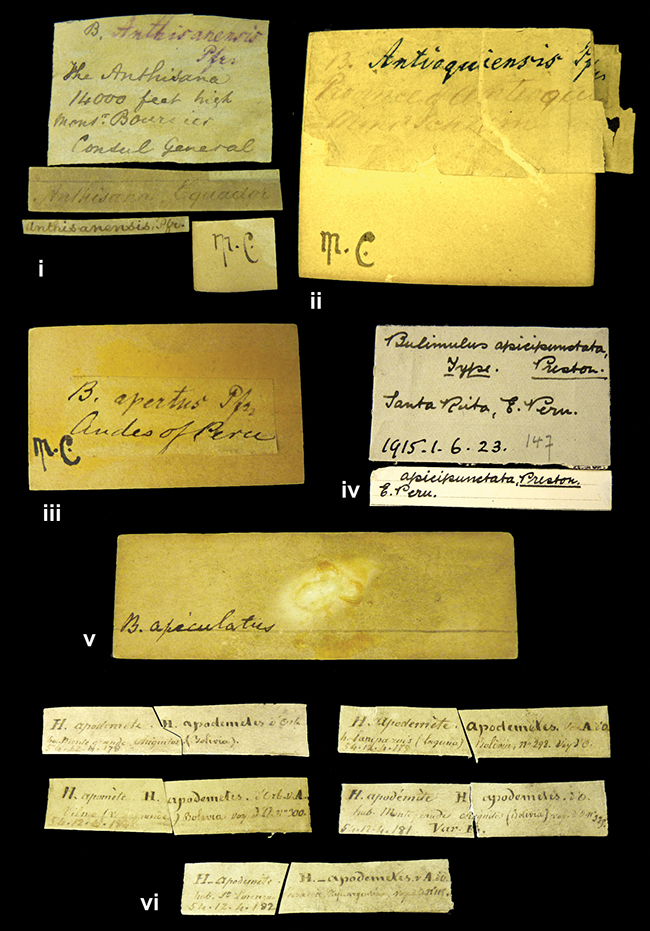
Labels. **i** *Bulimus anthisanensis* Pfeiffer, 1853 **ii** *Bulimus antioquensis* Pfeiffer, 1855 **iii** *Bulimus apertus* Pfeiffer in Dunker et al., 1855 **iv** *Bulimulus apicepunctata* Preston, 1914 **v** *Bulimus apiculatus* J.E. Gray, 1834 **vi** *Helix apodemeta* d’Orbigny, 1835.

**Figure L6.**
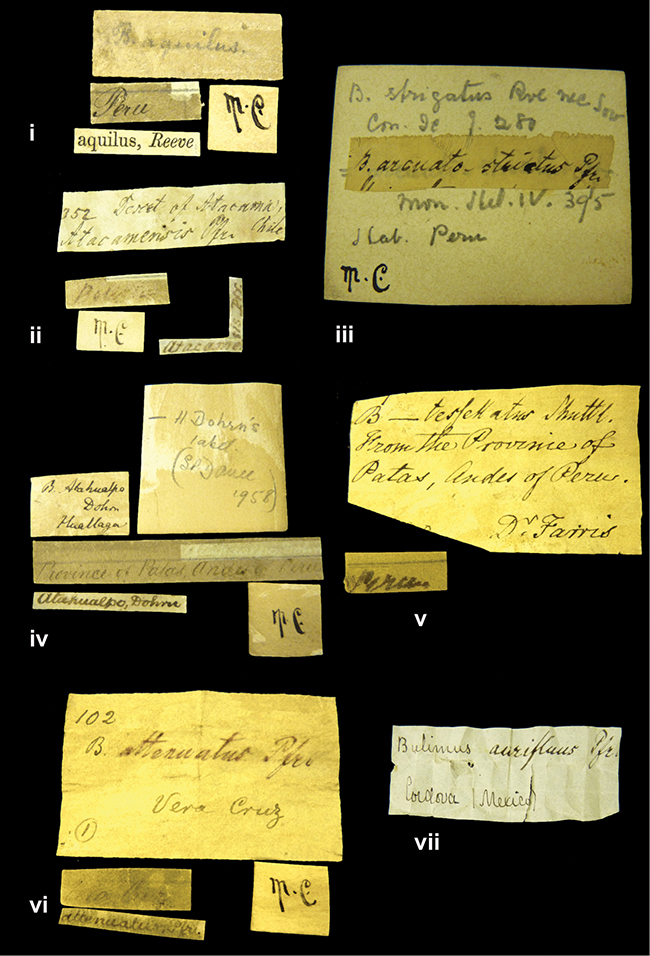
Labels. **i** *Bulimus aquilus* Reeve, 1848 **ii** *Bulimus atacamensis* Pfeiffer, 1856 **iii** *Bulimus arcuatostriatus* Pfeiffer, 1855 **iv–v** *Bulimulus atahualpa* Dohrn, 1863 **vi** *Bulimus attenuatus* Pfeiffer, 1853 **vii** *Bulimus aurifluus* Pfeiffer 1857.

**Figure L7.**
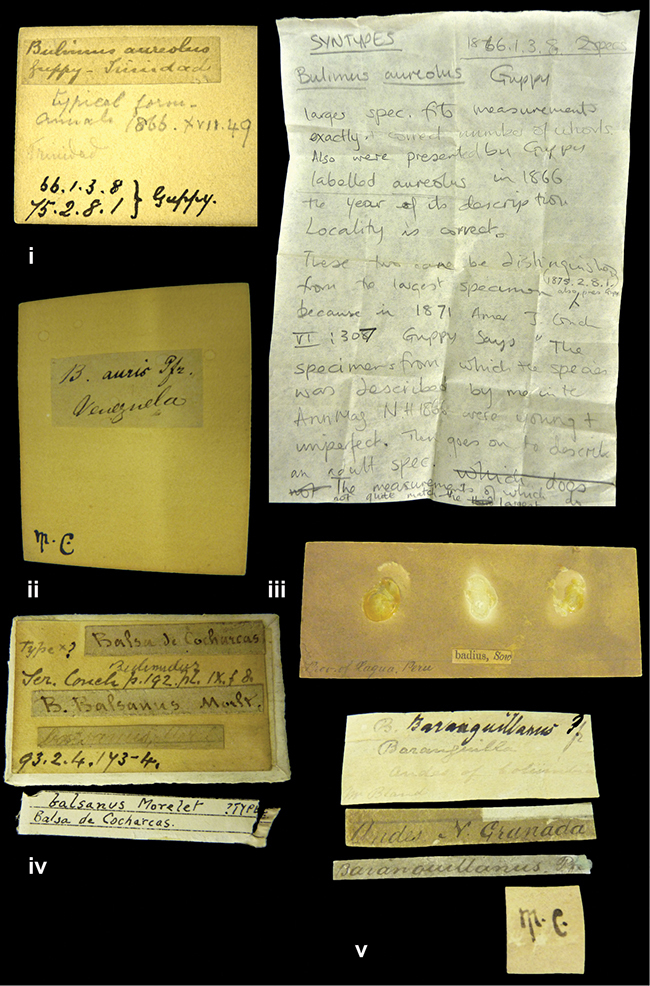
Labels. **i** *Bulimus aureolus* Guppy, 1866 **ii** *Bulimus auris* Pfeiffer, 1866 **iii** *Bulinus badius* Sowerby I, 1835 **iv** *Bulimus balsanus* Morelet, 1863 **v** *Bulimus baranguillanus* Pfeiffer 1853.

**Figure L8.**
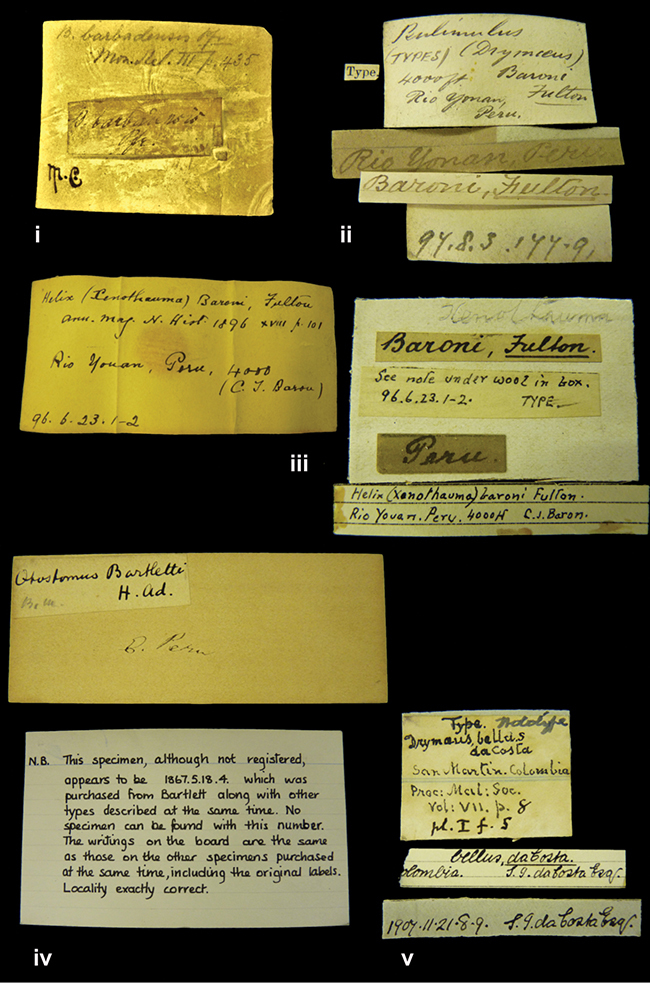
Labels. **i** *Bulimus barbadensis* Pfeiffer, 1853 **ii** *Bulimulus (Drymaeus) baroni* Fulton, 1897 **iii** *Helix (Xenothauma) baroni* Fulton, 1896 **iv** *Otostomus bartletti* H. Adams, 1867 **v** *Drymaeus bellus* da Costa, 1906.

**Figure L9.**
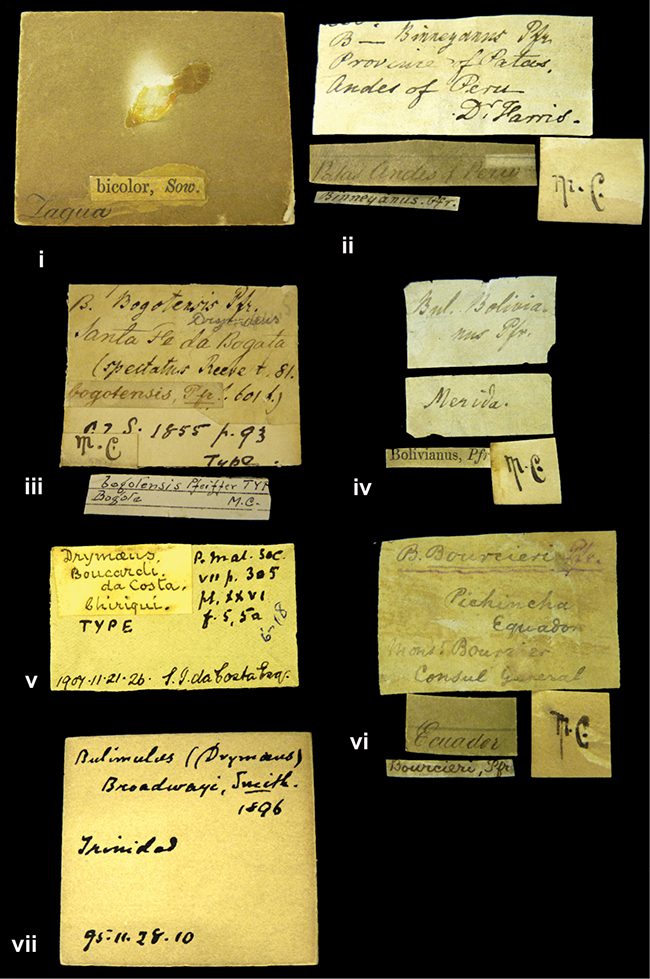
Labels. **i** *Bulinus bicolor* Sowerby I, 1835 **ii** *Bulimus binneayanus* Pfeiffer, 1857 **iii** *Bulimus bogotensis* Pfeiffer, 1855 **iv** *Bulimus bolivianus* Pfeiffer, 1846 **v** *Drymaeus boucardi* da Costa, 1907 **vi** *Bulimus bourcieri* Pfeiffer, 1853 **vii** *Bulimulus (Drymaeus) broadwayi* E.A. Smith, 1896.

**Figure L10.**
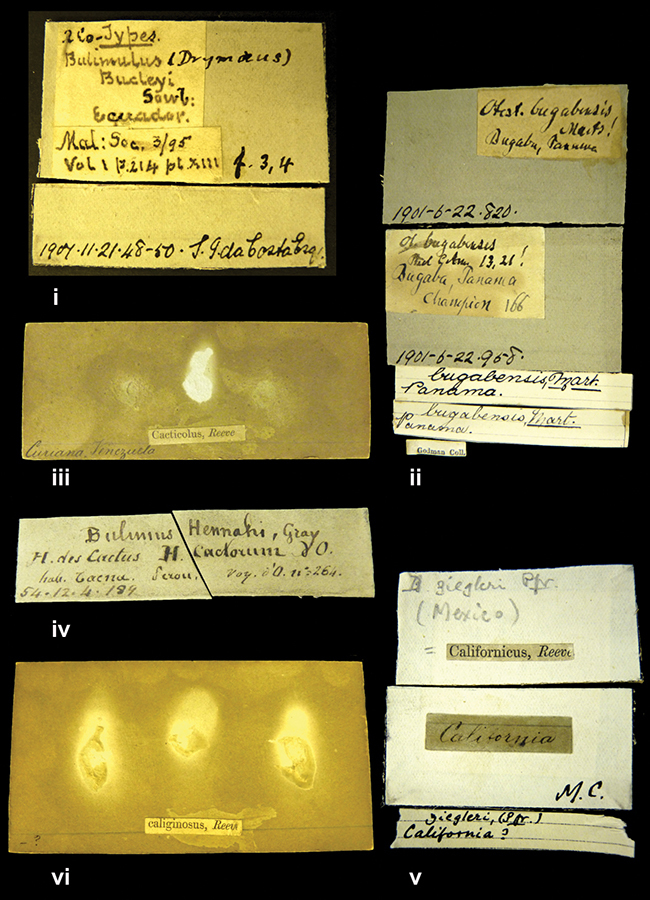
Labels. **i** *Bulimulus (Drymaeus) buckleyi* Sowerby III, 1895 **ii** *Otostomus bugabensis* Martens, 1893 **iii** *Bulimus cacticolus* Reeve, 1849 **iv** *Helix cactorum* d’Orbigny, 1835 **v** *Bulimus californicus* Reeve, 1848 **vi** *Bulimus caliginosus* Reeve, 1849.

**Figure L11.**
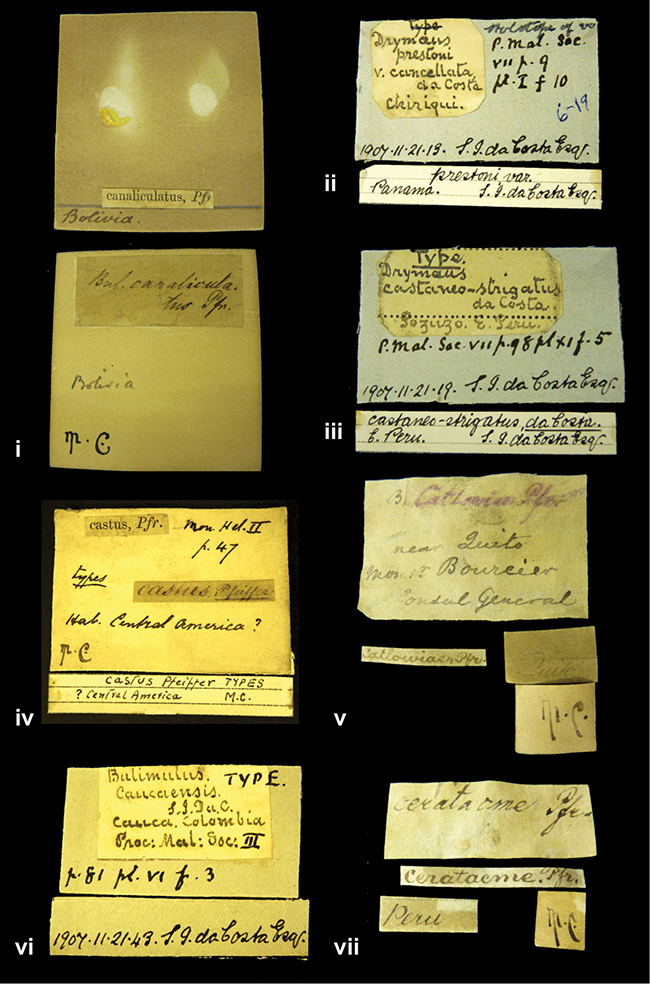
Labels. **i** *Bulimus canaliculatus* Pfeiffer, 1845 **ii** *Drymaeus prestoni cancellata* da Costa, 1906 **iii** *Drymaeus castaneostrigatus* da Costa, 1906 **iv** *Bulimus castus* Pfeiffer, 1847 **v** *Bulimus catlowiae* Pfeiffer, 1853 **vi** *Bulimulus (Drymaeus) caucaensis* da Costa, 1898 **vii** *Bulimus ceratacme* Pfeiffer, 1855.

**Figure L12.**
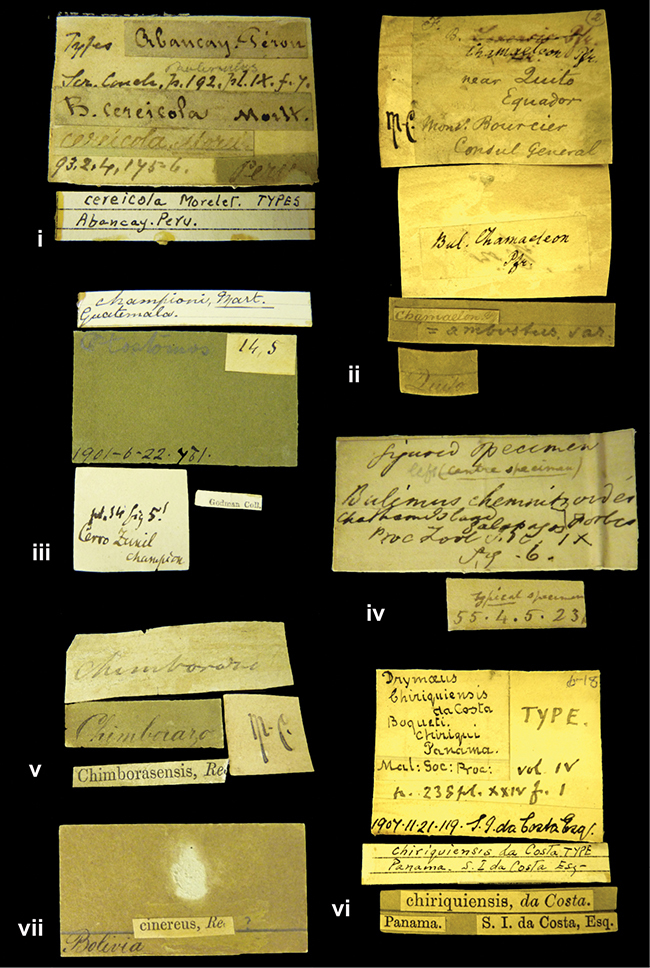
Labels. **i** *Bulimus cercicola* Morelet, 1863 **ii** *Bulimus chamaeleon* Pfeiffer, 1855 **iii** *Otostomus championi* Martens, 1893 **iv** *Bulimus chemnitzioides* Forbes, 1850 **v** *Bulimus chimborasensis* Reeve, 1848 **vi** *Drymaeus chiriquiensis* da Costa, 1901 **vii** *Bulimus cinereus* Reeve, 1849.

**Figure L13.**
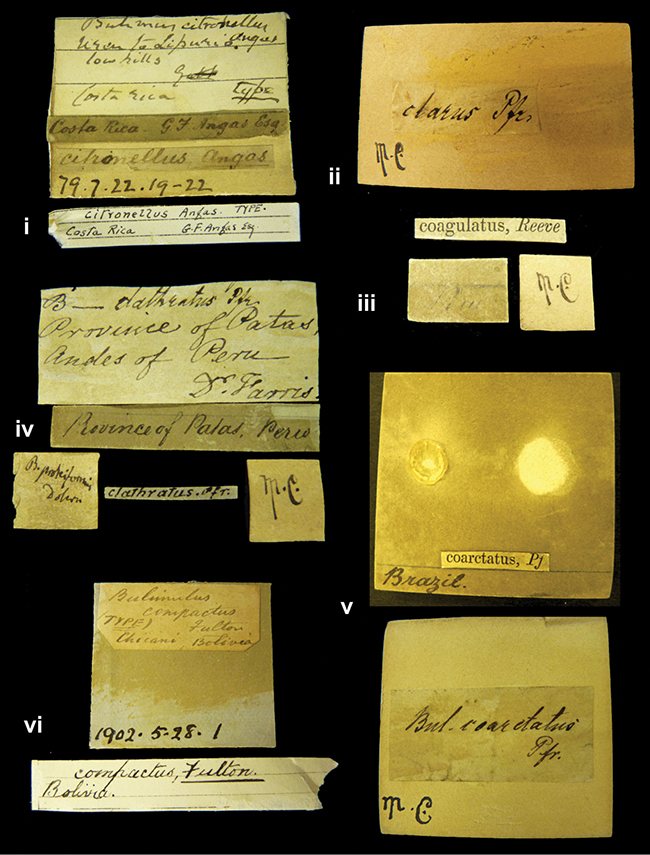
Labels. **i** *Bulimus citronellus* Angas, 1879 **ii** *Bulimus clarus* Pfeiffer, 1857 **iii** *Bulimus clathratus* Pfeiffer, 1858 **iv** *Bulimus coagulatus* Reeve, 1849 **v** *Bulimus coarctatus* Pfeiffer, 1845 **vi** *Bulimulus compactus* Fulton, 1902.

**Figure L14.**
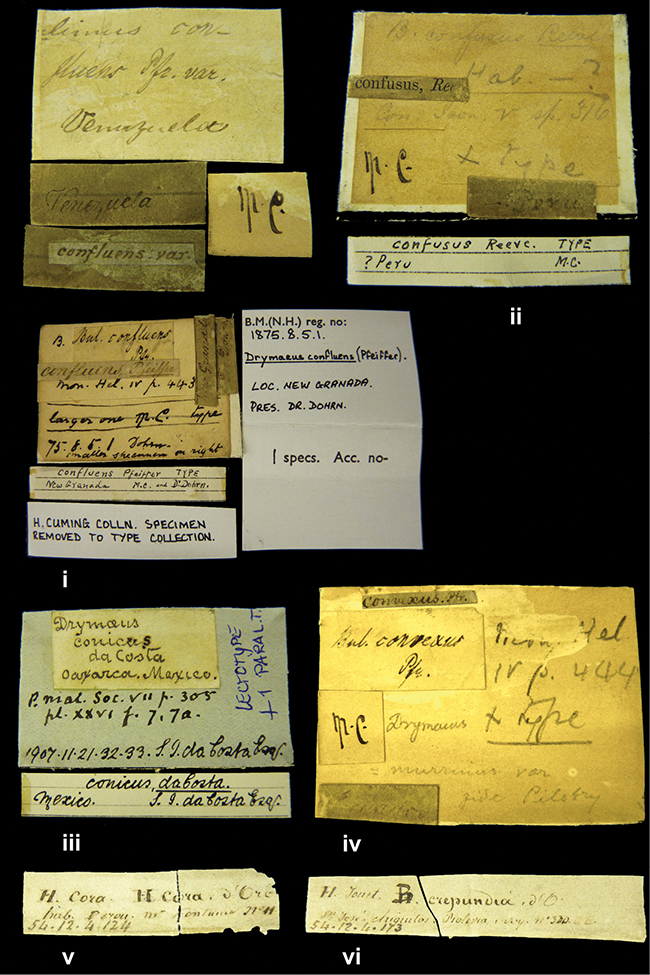
Labels. **i** *Bulimus confluens* Pfeiffer, 1855 **ii** *Bulimus confusus* Reeve, 1848 **iii** *Drymaeus conicus* da Costa, 1907 **iv** *Bulimus convexus* Pfeiffer, 1855 **v** *Helix cora* d’Orbigny, 1835 **vi** *Helix crepundia* d’Orbigny, 1835.

**Figure L15.**
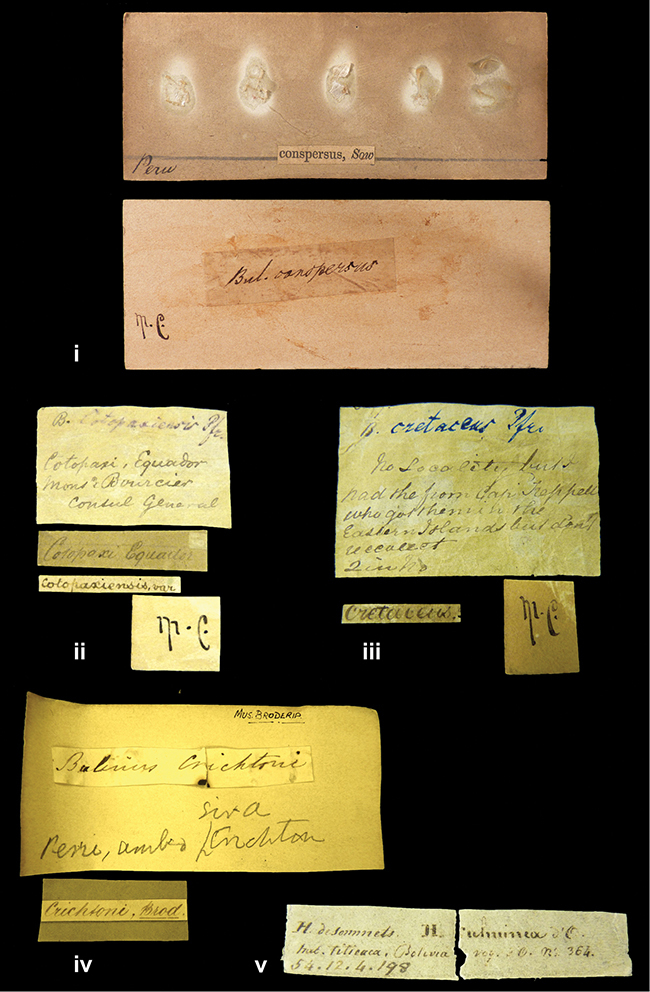
Labels. **i** *Bulinus conspersus* Sowerby I, 1833 **ii** *Bulimus cotopaxiensis* Pfeiffer, 1853 **iii** *Bulimus cretaceus* Pfeiffer, 1855 **iv** *Bulinus crichtoni* Broderip, 1836 **v** *Helix culminea* d’Orbigny, 1835.

**Figure L16.**
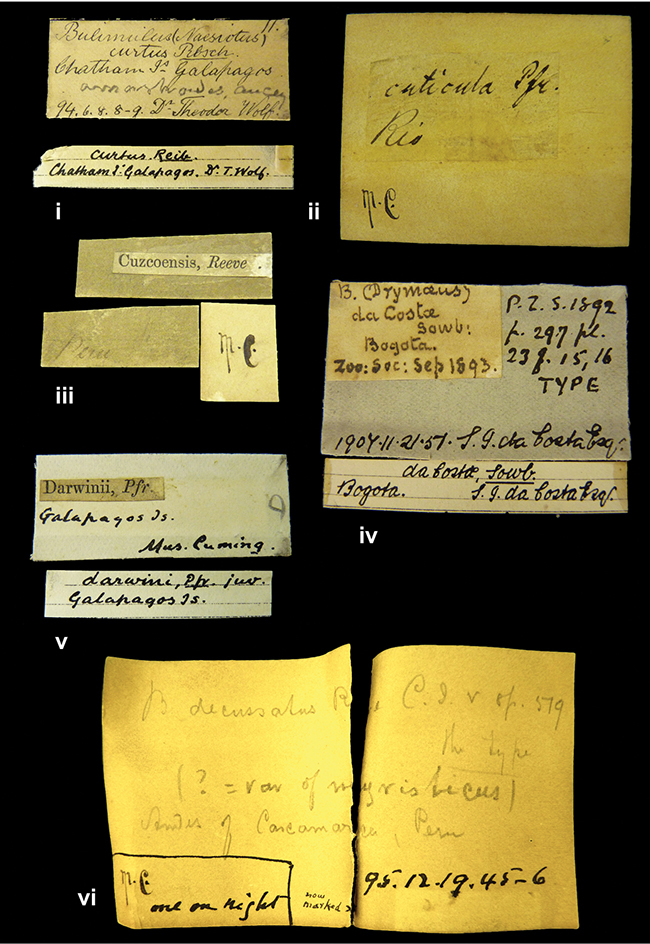
Labels. **i** *Bulimulus (Naesiotus) curtus* Reibisch, 1892 **ii** *Bulimus cuticula* Pfeiffer, 1855 **iii** *Bulimus cuzcoensis* Reeve, 1849 **iv** *Bulimulus dacostae* Sowerby III, 1892 **v** *Bulimus darwini* Pfeiffer, 1846 **vi** *Bulimus decussatus* Reeve, 1849.

**Figure L17.**
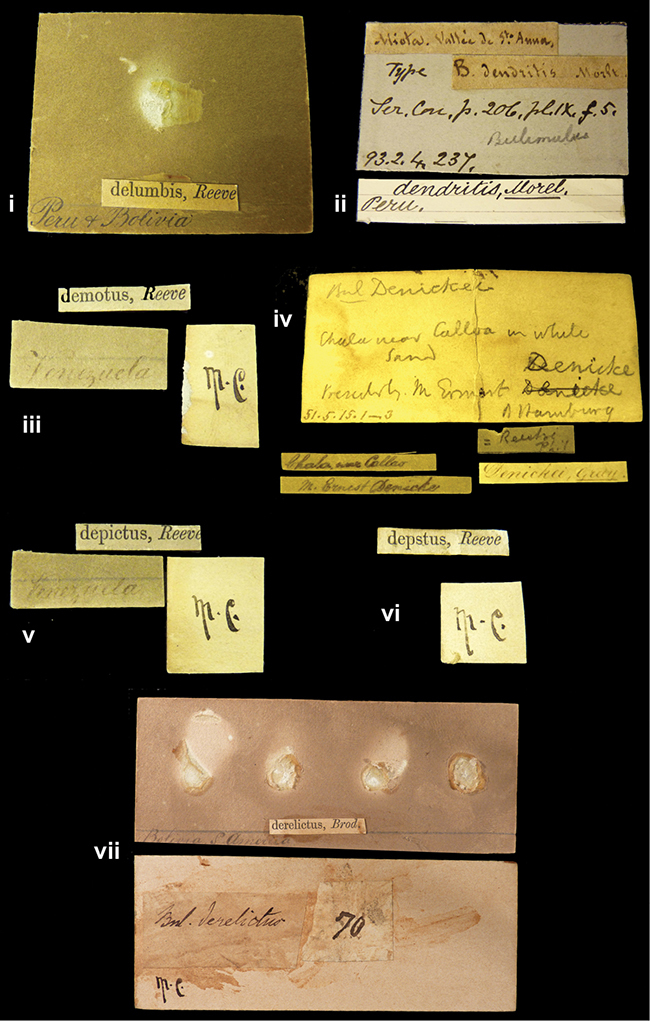
Labels. **i** *Bulimus delumbis* Reeve, 1849 **ii** *Bulimus dentritis* Morelet, 1863 **iii** *Bulimus demotus* Reeve, 1850 **iv** *Bulimus denickei* J.E. Gray, 1852 **v** *Bulimus depictus* Reeve, 1849 **vi** *Bulimus depstus* Reeve, 1849 **vii** *Bulinus derelictus* Broderip in Broderip and Sowerby I 1832.

**Figure L18.**
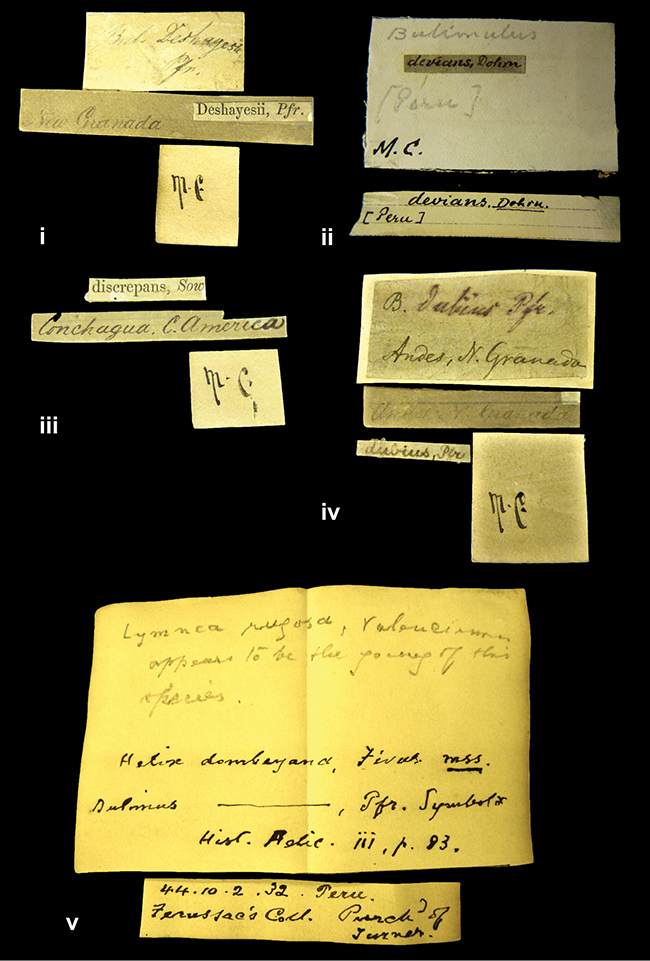
Labels. **i** *Bulimus deshayesi* Pfeiffer, 1845 **ii** *Bulimus devians* Dohrn, 1863 **iii** *Bulinus discrepans* Sowerby I, 1833 **iv** *Bulimus dubius* Pfeiffer, 1853 **v** *Bulimus dombeyanus* ‘Férussac’ Pfeiffer, 1846.

**Figure L19.**
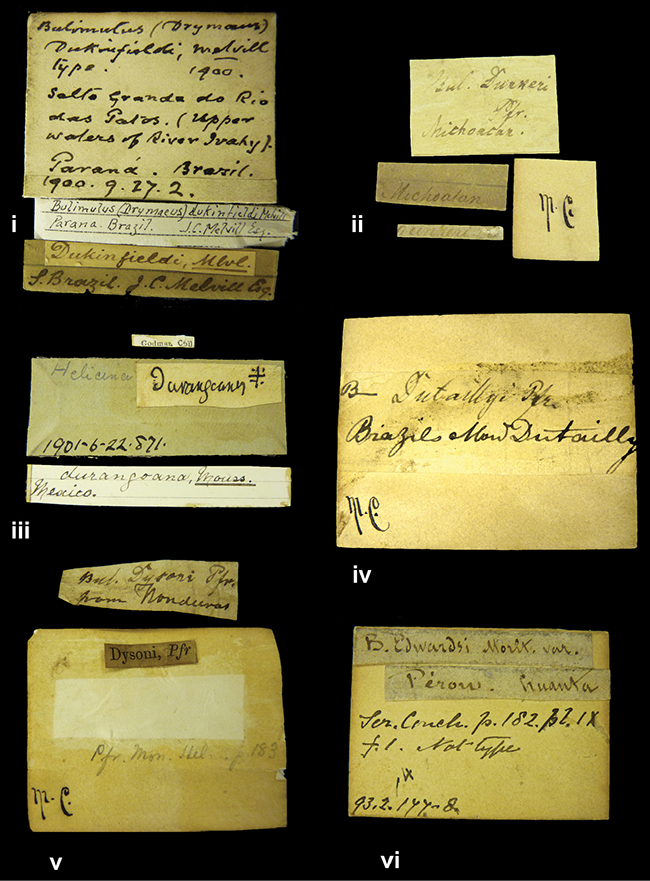
Labels. **i** *Bulimulus (Drymaeus) dukinfieldi* Melvill, 1900 **ii** *Bulimus dunkeri* Pfeiffer in Philippi 1846 **iii** *Bulimulus (Peronaeus) durangoanus* Martens, 1893 **iv** *Bulimus dutaillyi* Pfeiffer, 1857 **v** *Bulimus dysoni* Pfeiffer, 1846 **vi** *Bulimus edwardsi* Morelet, 1863.

**Figure L20.**
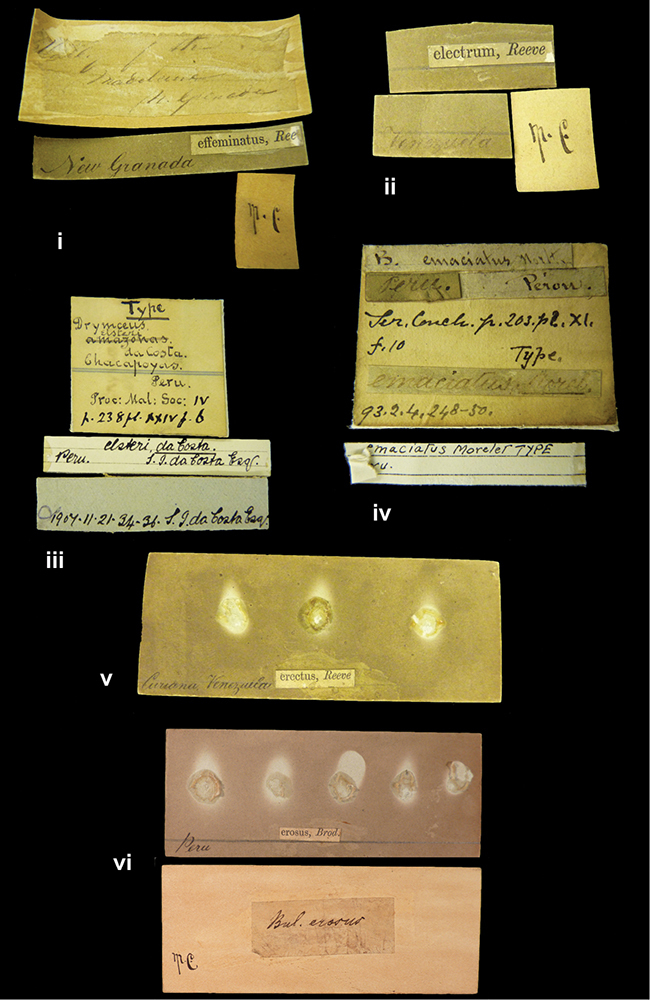
Labels. **i** *Bulimus effeminatus* Reeve 1848 **ii** *Bulimus electrum* Reeve, 1848 **iii** *Drymaeus elsteri* da Costa, 1901 **iv** *Bulimus emaciatus* Morelet, 1863 **v** *Bulimus erectus* Reeve, 1849 **vi** *Bulimus erosus* Broderip in Broderip and Sowerby I 1832.

**Figure L21.**
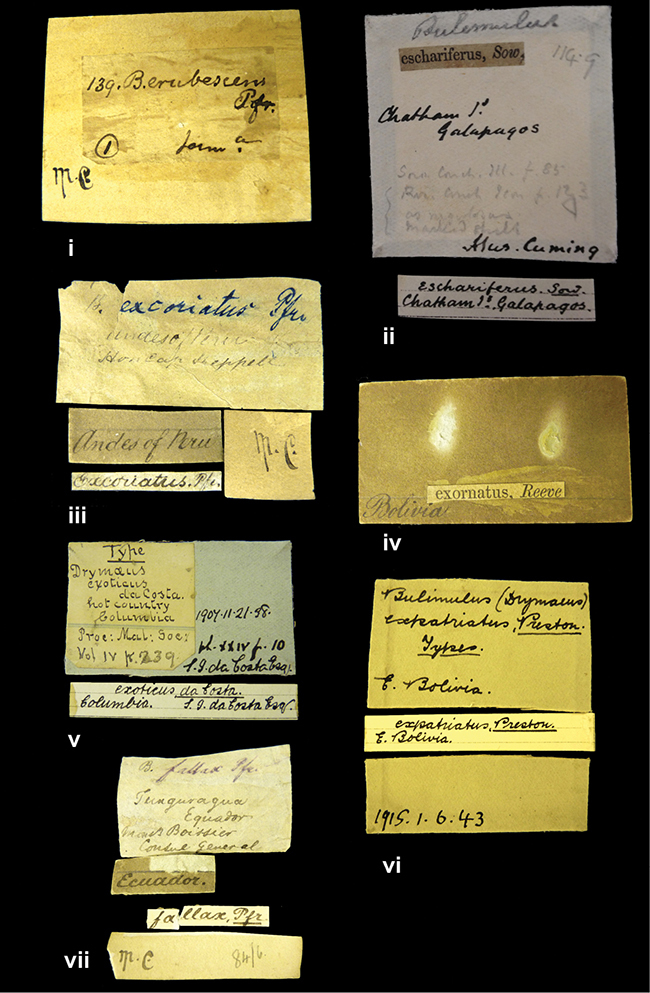
Labels. **i** *Bulimus erubescens* Pfeiffer, 1847 **ii** *Bulinus eschariferus* Sowerby I, 1833 **iii** *Bulimus excoriatus* Pfeiffer, 1855 **iv** *Bulimus exornatus* Reeve, 1849 **v** *Drymaeus exoticus* da Costa, 1901 **vi** *Bulimulus (Drymaeus) expatriatus* Preston, 1909 **vii** *Bulimus fallax* Pfeiffer, 1853.

**Figure L22.**
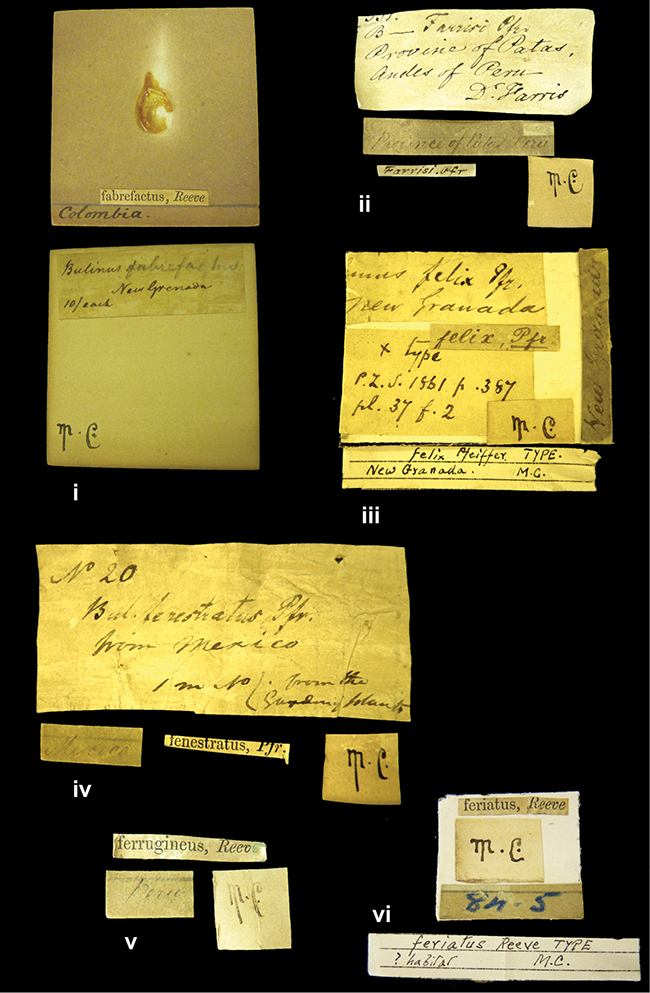
Labels. **i** *Bulimus fabrefactus* Reeve, 1848 **ii** *Bulimus farrisi* Pfeiffer, 1858 **iii** *Bulimus felix* Pfeiffer, 1862 **iv** *Bulimus fenestratus* Pfeiffer, 1846 **v** *Bulimus ferrugineus* Reeve, 1849 **vi** *Bulimus feriatus* Reeve, 1848.

**Figure L23.**
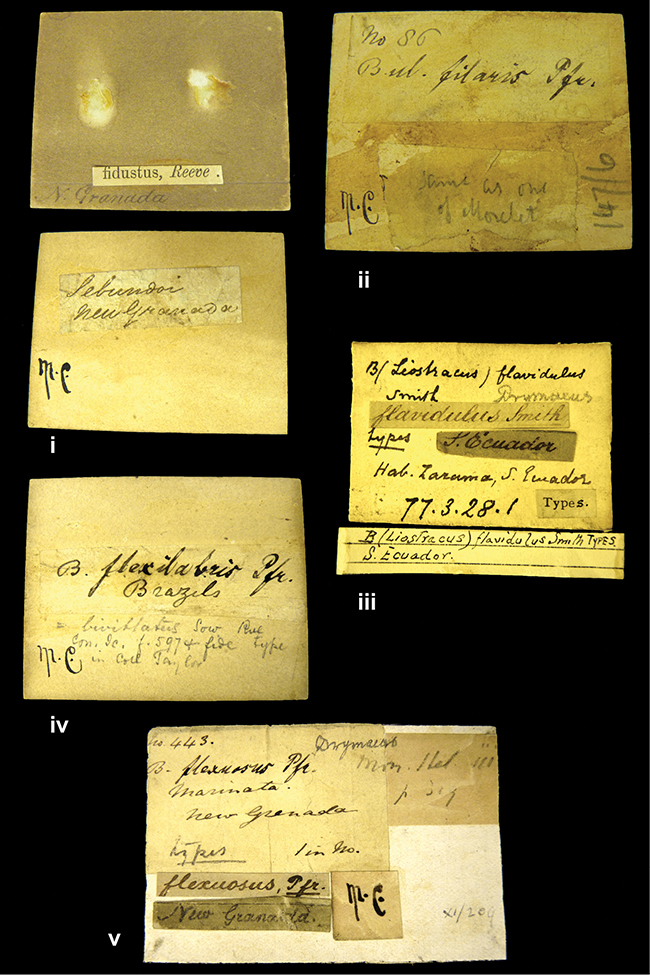
Labels. **i** *Bulimus fidustus* Reeve, 1849 **ii** *Bulimus filaris* Pfeiffer, 1853 **iii** *Bulimus (Liostracus) flavidulus* E.A. Smith, 1877 **iv** *Bulimus flexilabris* Pfeiffer, 1853 **v** *Bulimus flexuosus* Pfeiffer, 1853.

**Figure L24.**
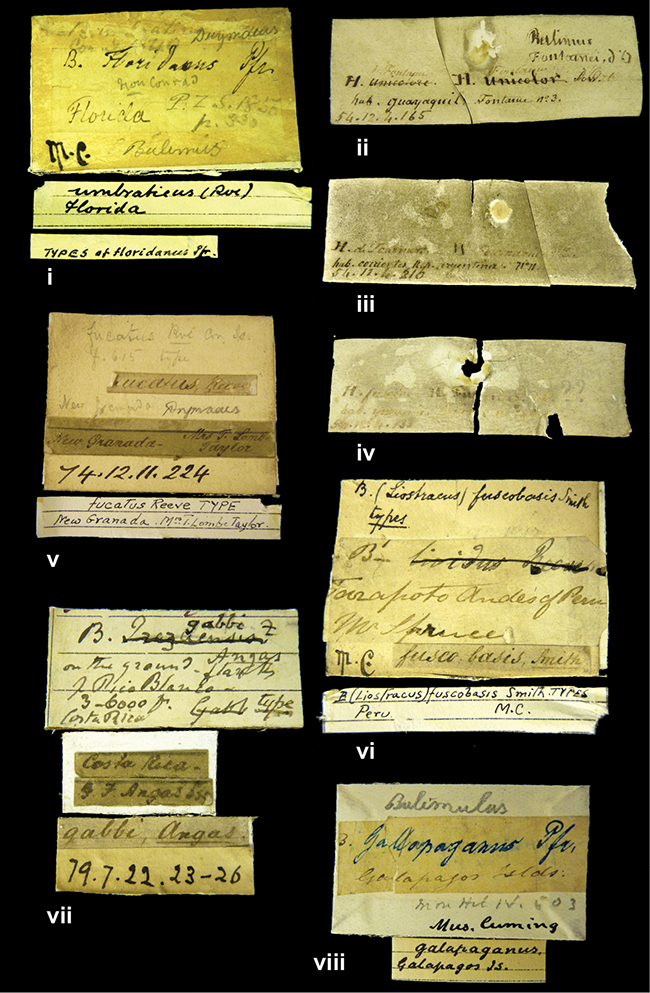
Labels. **i** *Bulimus floridanus* Pfeiffer, 1857 **ii** *Bulimus fontainii* d’Orbigny, 1838 **iii** *Bulimus fourmiersi* d’Orbigny, 1837 **iv** *Helix fusoides* d’Orbigny, 1835 **v** *Bulimus fucatus* Reeve, 1849 **vi** *Bulimus (Liostracus) fuscobasis* E.A. Smith, 1877 **vii** *Bulimus gabbi* Angas, 1879 **viii** *Bulimus galapaganus* Pfeiffer, 1855.

**Figure L25.**
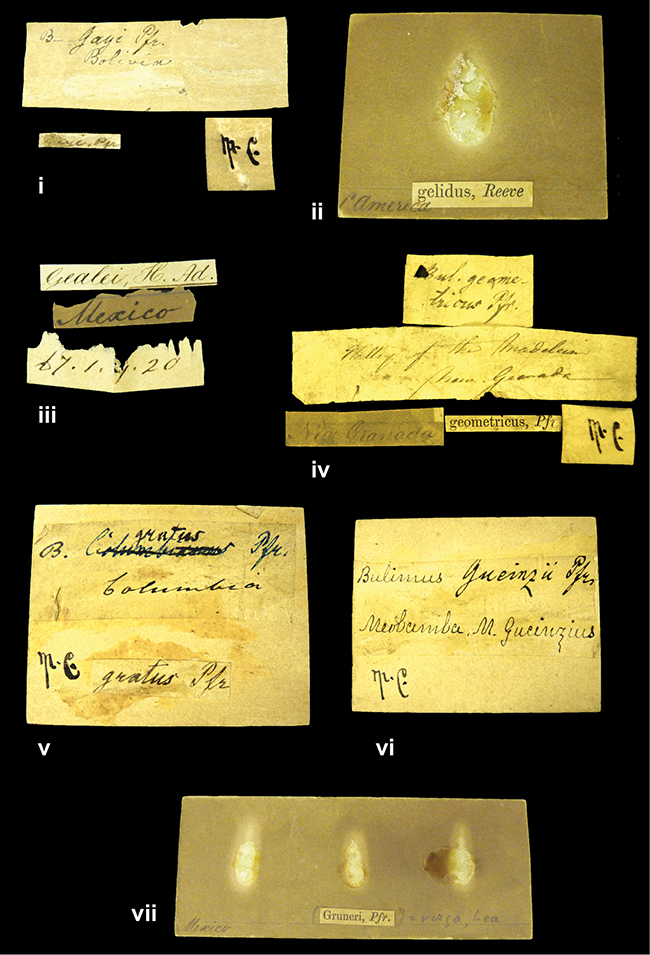
Labels. **i** *Bulimus gayi* Pfeiffer, 1857 **ii** *Bulimus gelidus* Reeve, 1849 **iii** *Bulimus (Mesembrinus) gealei* H. Adams,1867 **iv** *Bulimus geometricus* Pfeiffer, 1846 **v** *Bulimus columbiensis* Pfeiffer, 1855 = *Bulimus gratus* Pfeiffer, 1856 **vi** *Bulimus gueinzii* Pfeiffer, 1857 **vii** *Bulimus gruneri* Pfeiffer, 1846.

**Figure L26.**
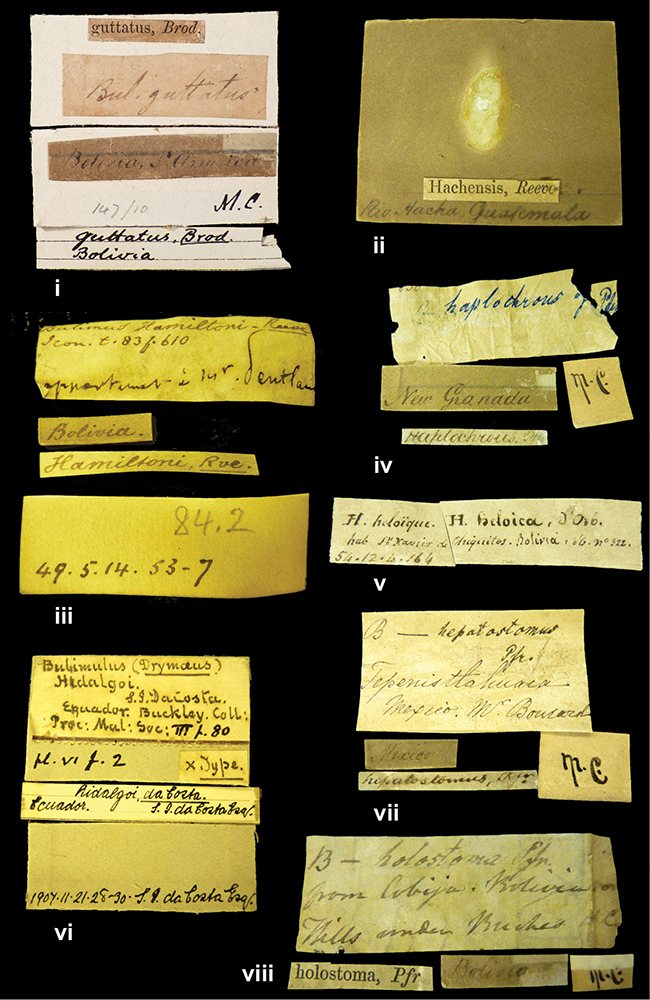
Labels. **i** *Bulinus guttatus* Broderip in Broderip and Sowerby I 1832 **ii** *Bulimus hachensis* Reeve, 1850 **iii** *Bulimus hamiltoni* Reeve, 1849 **iv** *Bulimus haplochrous* Pfeiffer, 1855 **v** *Helix heloica* d’Orbigny, 1835 **vi** *Bulimulus (Drymaeus) hidalgoi* da Costa, 1898 **vii** *Bulimus hepatostomus* Pfeiffer, 1861 **viii** *Bulimus holostoma* Pfeiffer, 1846.

**Figure L27.**
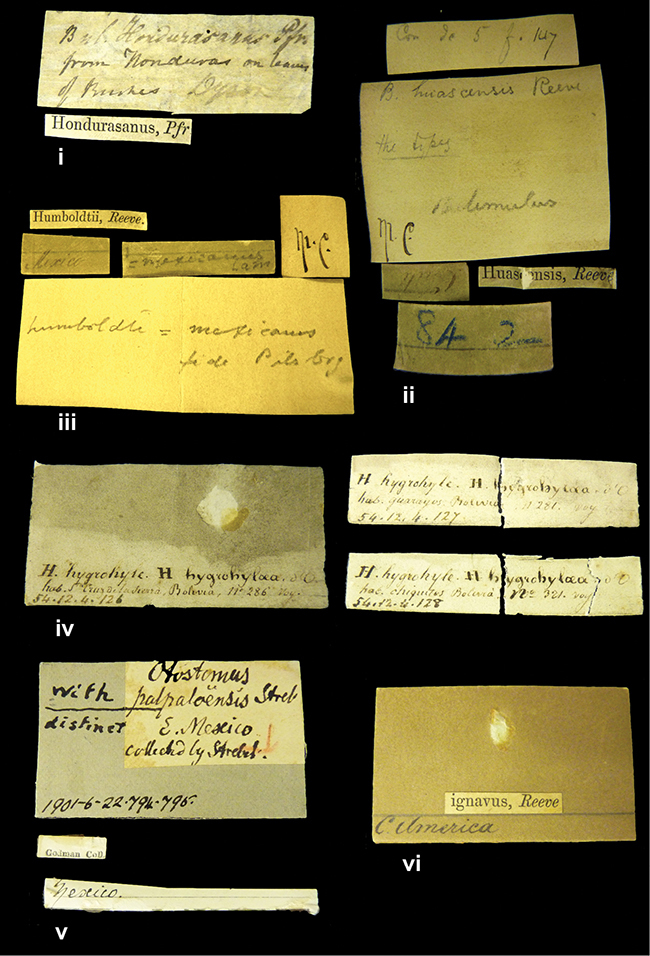
Labels. **i** *Bulimus hondurasanus* Pfeiffer, 1846 **ii** *Bulimus huascensis* Reeve, 1848 **iii** *Bulimus humboldtii* Reeve, 1849 **iv** *Helix hygrohylaea* d’Orbigny, 1835 **v** *Otostomus emeus hypozonus* Martens, 1893 **vi** *Bulimus ignavus* Reeve, 1849.

**Figure L28.**
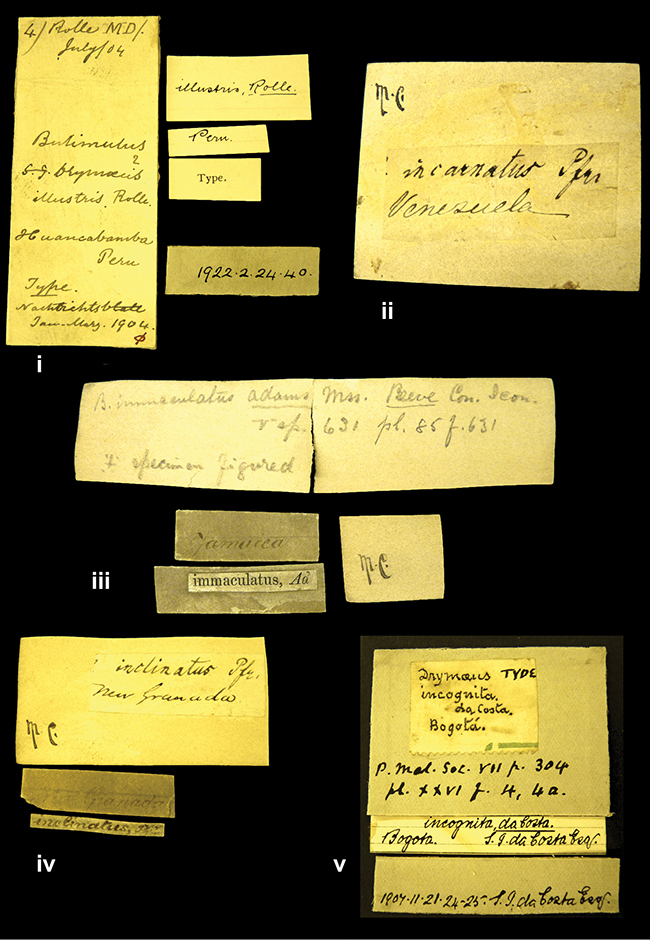
Labels. **i** *Bulimus (?) illustris* Rolle, 1905 **ii** *Bulimus incarnatus* Pfeiffer, 1855 **iii** *Bulimus immaculatus* C.B. Adams in Reeve 1850 **iv** *Bulimus inclinatus* Pfeiffer, 1862 **v** *Drymaeus incognita* da Costa, 1907.

**Figure L29.**
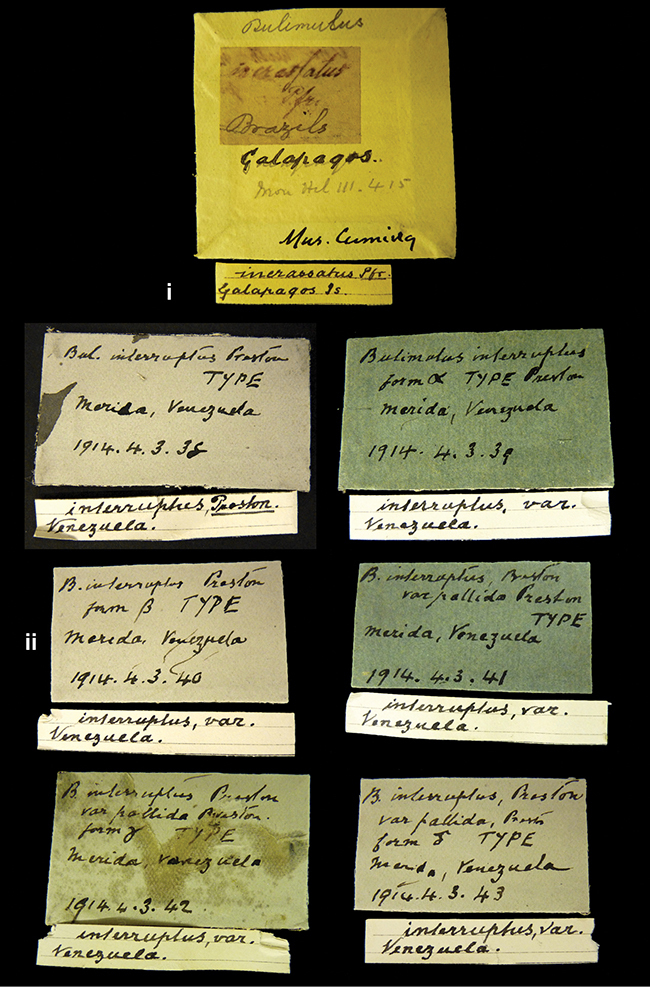
Labels. **i** *Bulimus incrassatus* Pfeiffer, 1853 **ii** *Bulimulus (Drymaeus) interruptus* Preston, 1909.

**Figure L30.**
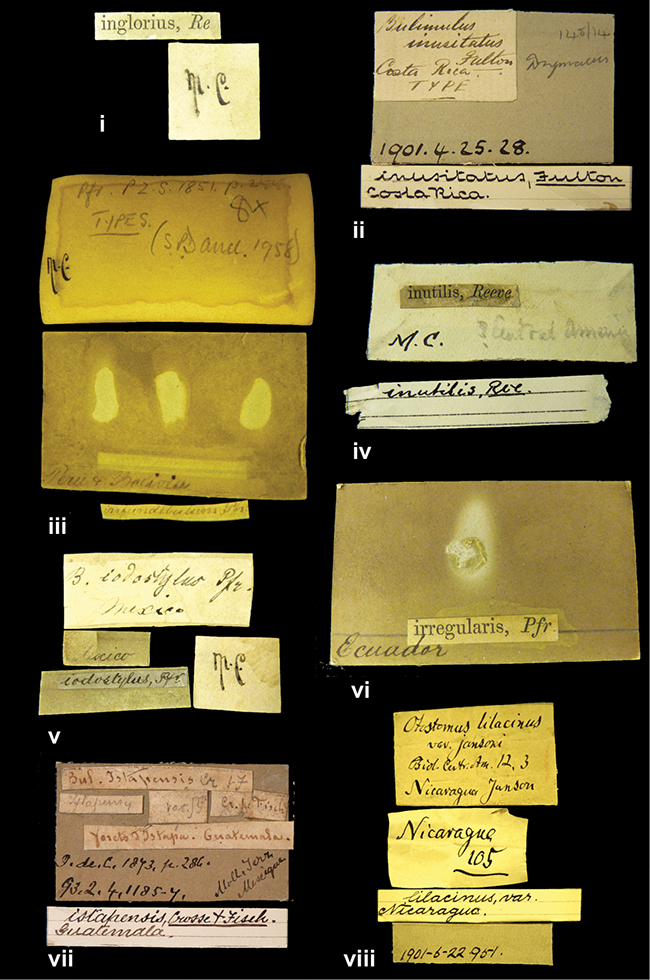
Labels. **i** *Bulimus inglorius* Reeve, 1848 **ii** *Bulimulus (Drymaeus) inusitatus* Fulton, 1900 **iii** *Bulimus infundibulum* Pfeiffer, 1853 **iv** *Bulimus inutilis* Reeve, 1850 **v** *Bulimus iodostylus* Pfeiffer, 1861 **vi** *Bulimus irregularis* Pfeiffer, 1848 **vii** *Bulimulus istapensis* Crosse and Fischer, 1873 **viii** *Otostomus lilacinus jansoni* Martens, 1893.

**Figure L31.**
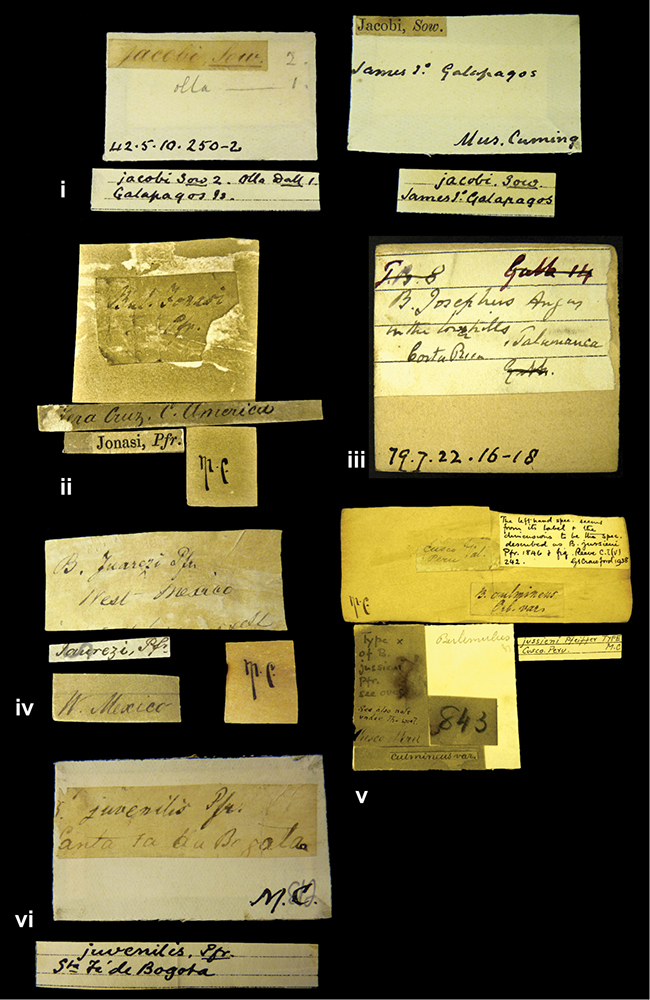
Labels. **i** *Bulinus jacobi* Sowerby I, 1833 **ii** *Bulimus jonasi* Pfeiffer in Philippi 1846 **iii** *Bulimus josephus* Angas, 1878 **iv** *Bulimus juarezi* Pfeiffer, 1866 **v** *Bulimus jussieui* Pfeiffer, 1846 **vi** *Bulimus juvenilis* Pfeiffer, 1855.

**Figure L32.**
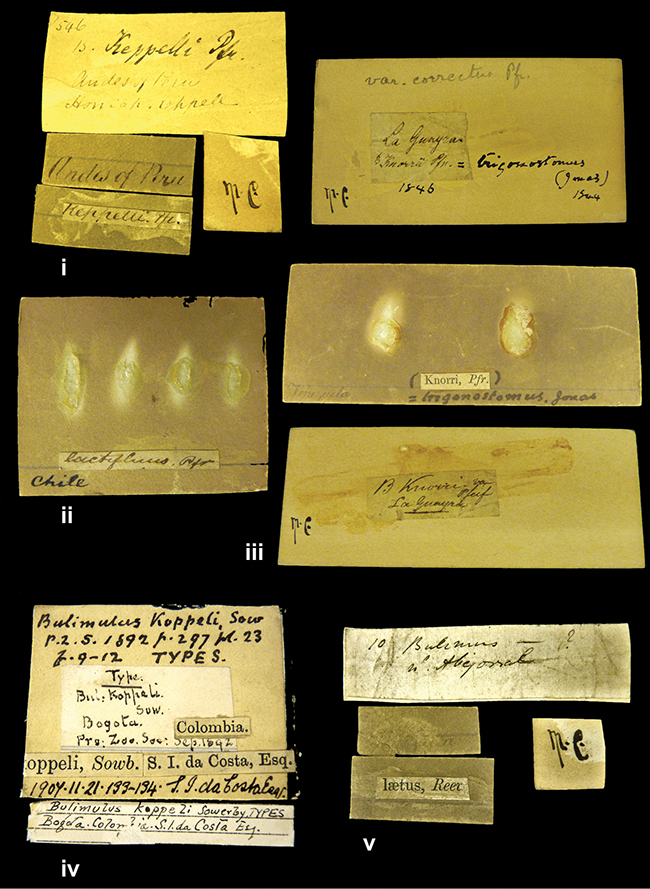
Labels. **i** *Bulimus keppelli* Pfeiffer, 1853 **ii** *Bulimus lactifluus* Pfeiffer, 1857 **iii** *Bulimus knorri* Pfeiffer in Philippi 1846 **iv** *Bulimus koppeli* Sowerby III, 1892 **v** *Bulimus laetus* Reeve, 1849.

**Figure L33.**
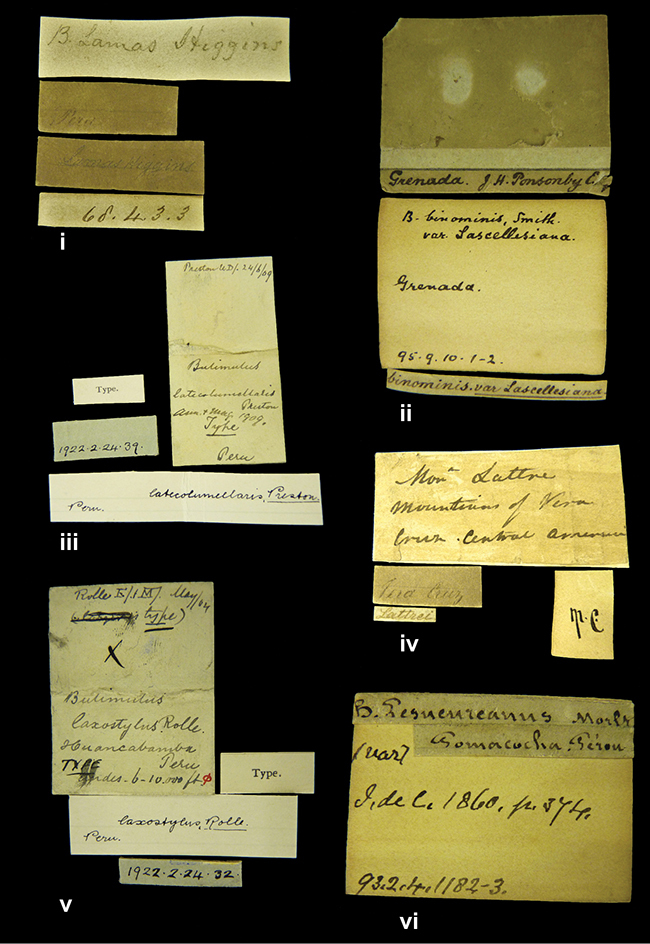
Labels. **i** *Bulimus (Otostomus) lamas* Higgins, 1868 **ii** *Bulimulus (Drymaeus) binominis lascellianus* E.A. Smith, 1895 **iii** *Bulimulus latecolumellaris* Preston, 1909 **iv** *Bulimus lattrei* Pfeiffer in Philippi 1846 **v** *Bulimulus laxostylus* Rolle, 1904 **vi** *Bulimus lesueureanus* Morelet, 1860.

**Figure L34.**
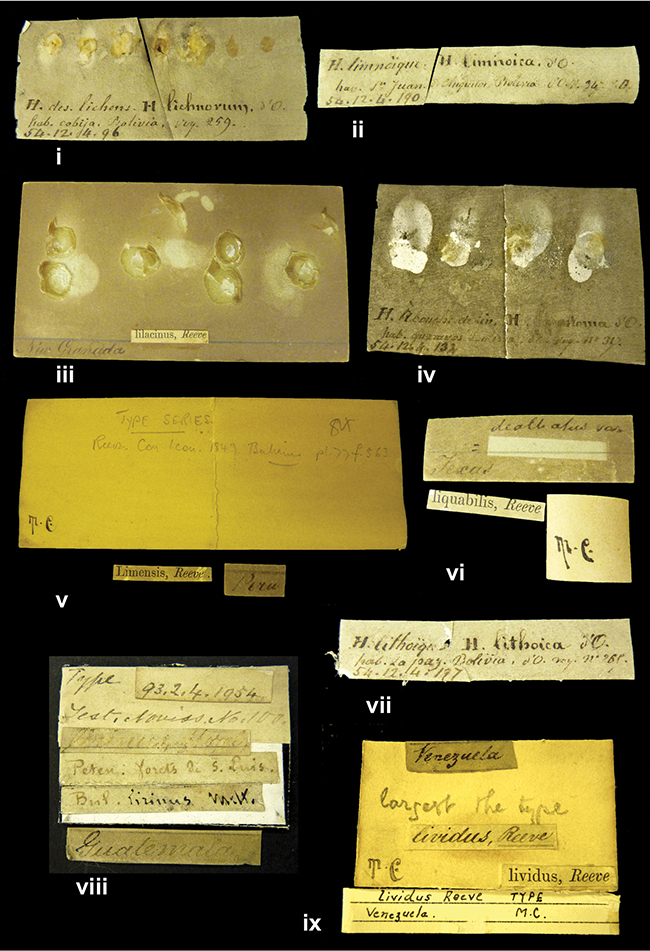
Labels. **i** *Helix lichnorum* d’Orbigny, 1835 **ii** *Helix limonoica* d’Orbigny, 1835 **iii** *Bulimus lilacinus* Reeve, 1849 **iv** *Helix linostoma* d’Orbigny, 1835 **v** *Bulimus limensis* Reeve, 1849 **vi** *Bulimus liquabilis* Reeve, 1848 **vii** *Helix lithoica* d’Orbigny, 1835 **viii** *Bulimus lirinus* Morelet, 1851 **ix** *Bulimus lividus* Reeve, 1850.

**Figure L35.**
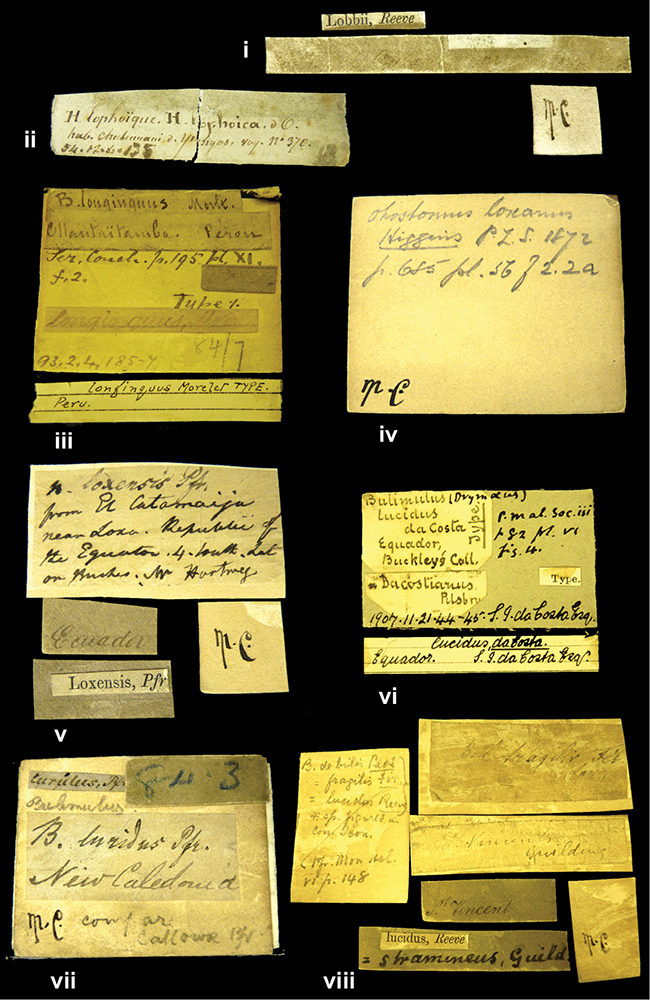
Labels. **i** *Bulimus lobbii* Reeve, 1849 **ii** *Helix lophoica* d’Orbigny, 1835 **iii** *Bulimus longinquus* Morelet, 1863 **iv** *Otostomus loxanus* Higgins, 1872 **v** *Bulimus loxensis* Pfeiffer, 1846 **vi** *Bulimulus (Drymaeus) lucidus* Da Costa, 1898 **vii** *Bulimus luridus* Pfeiffer, 1863 **viii** *Bulimus lucidus* Reeve, 1848.

**Figure L36.**
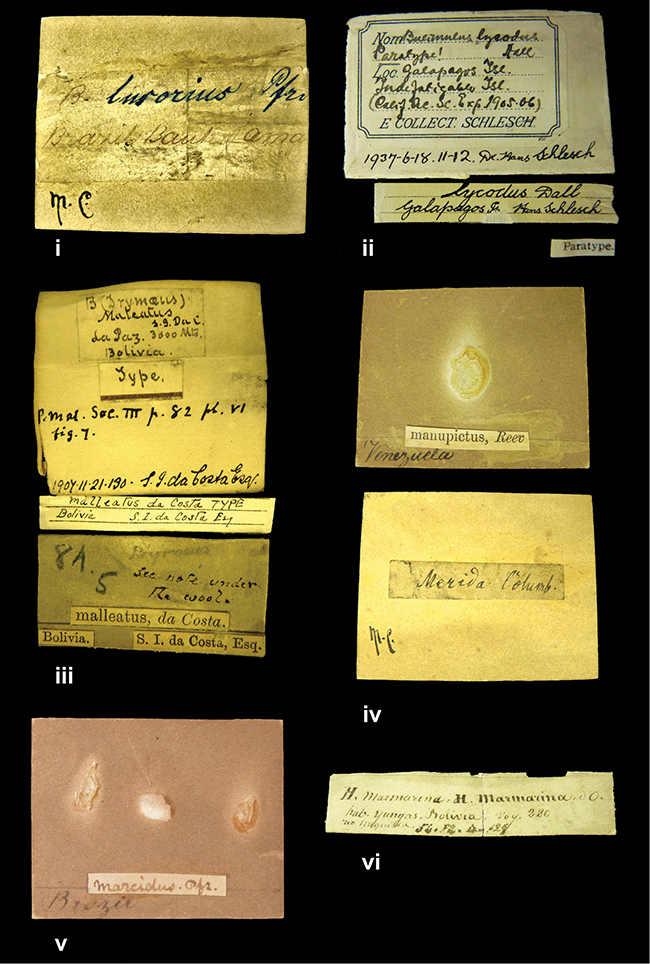
Labels. **i** *Bulimus lusorius* Pfeiffer, 1855 **ii** *Bulimulus (Naesiotus) lycodus* Dall, 1917 **iii** *Bulimulus (Drymaeus) malleatus* da Costa, 1898 **iv** *Bulimus manupictus* Reeve, 1848 **v** *Bulimus marcidus* Pfeiffer, 1853 **vi** *Helix marmarina* d’Orbigny, 1835.

**Figure L37.**
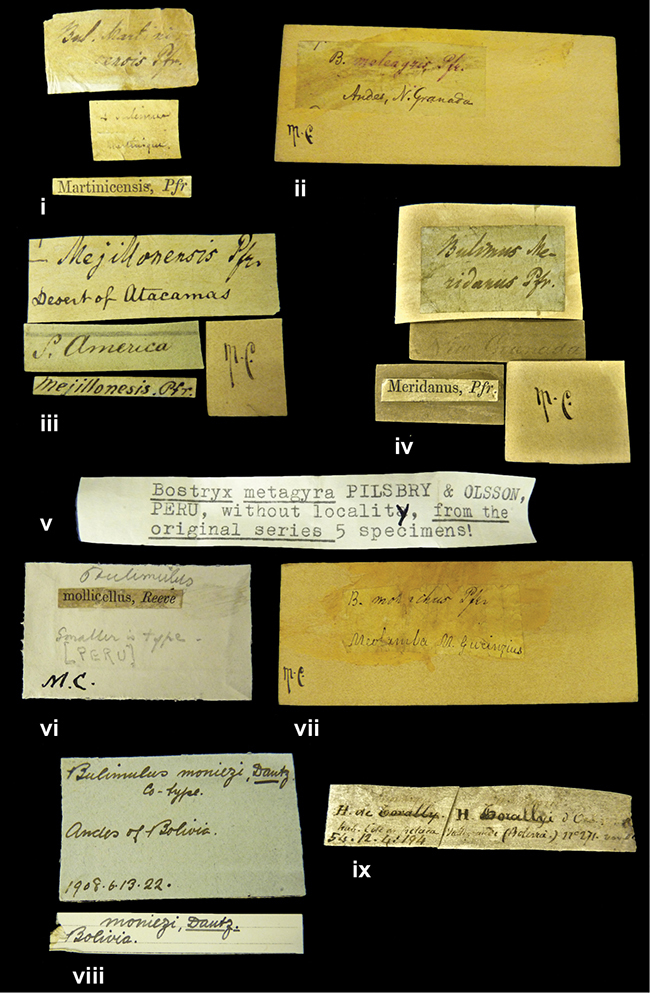
Labels. **i** *Bulimus martinicensis* Pfeiffer, 1846 **ii** *Bulimus meleagris* Pfeiffer, 1853 **iii** *Bulimus mejillonensis* Pfeiffer in Pfeiffer and Dunker, 1857 **iv** *Bulimus meridanus* Pfeiffer, 1846 **v** *Bostryx metagyra* Pilsbry & Olsson, 1949 **vi** *Bulimus mollicellus* Reeve, 1849 **vii** *Bulimus monachus* Pfeiffer, 1857 **viii** *Bulimulus (Bostryx) moniezi* Dautzenberg, 1896 **ix** *Bulimus montagnei* d’Orbigny, 1837.

**Figure L38.**
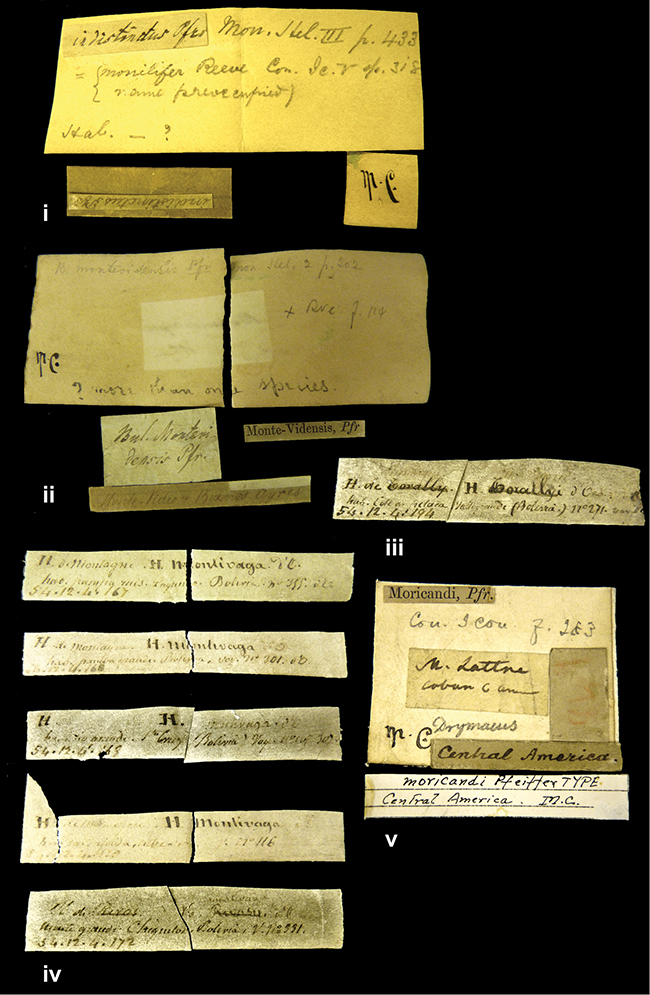
Labels. **i** *Bulimus monilifer* Reeve, 1848 **ii** *Bulimus montevidensis* Pfeiffer, 1846 **iii** *Bulimus montagnei* d’Orbigny, 1837 **iv** *Helix montivaga* d’Orbigny, 1835 **v** *Bulimus moricandi* Pfeiffer, 1847.

**Figure L39.**
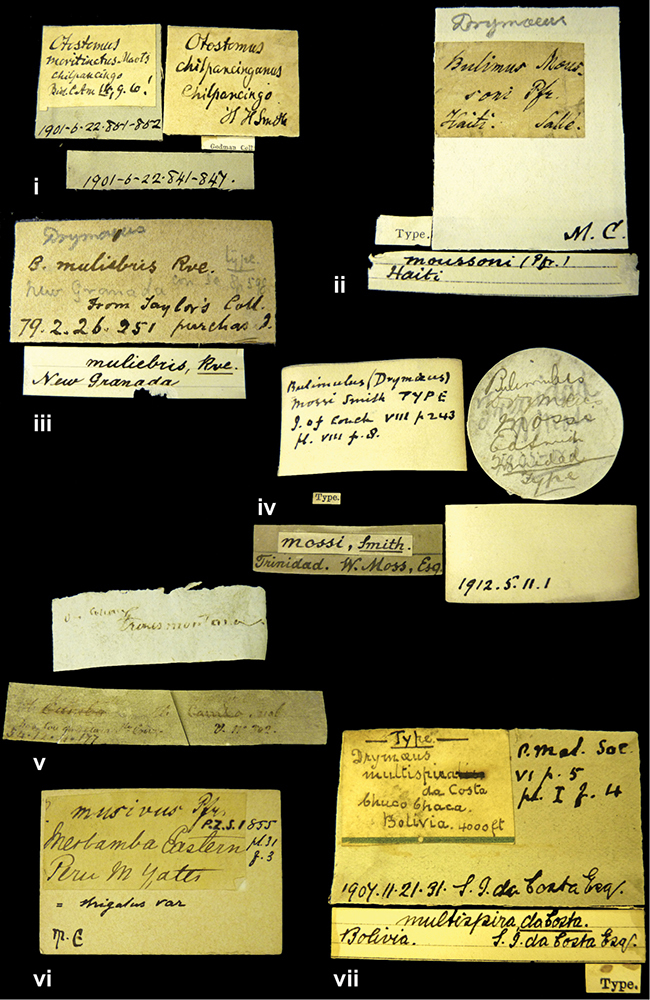
Labels. **i** *Otostomus moritinctus* Martens, 1893 **ii** *Bulimus moussoni* Pfeiffer, 1853 **iii** *Bulimus muliebris* Reeve, 1849 **iv** *Bulimulus (Drymaeus) mossi* E.A. Smith, 1896 **v** *Bulimus munsterii* d’Orbigny, 1837 **vi** *Drymaeus multispira* da Costa 1904 **vii** *Bulimus musivus* Pfeiffer, 1855.

**Figure L40.**
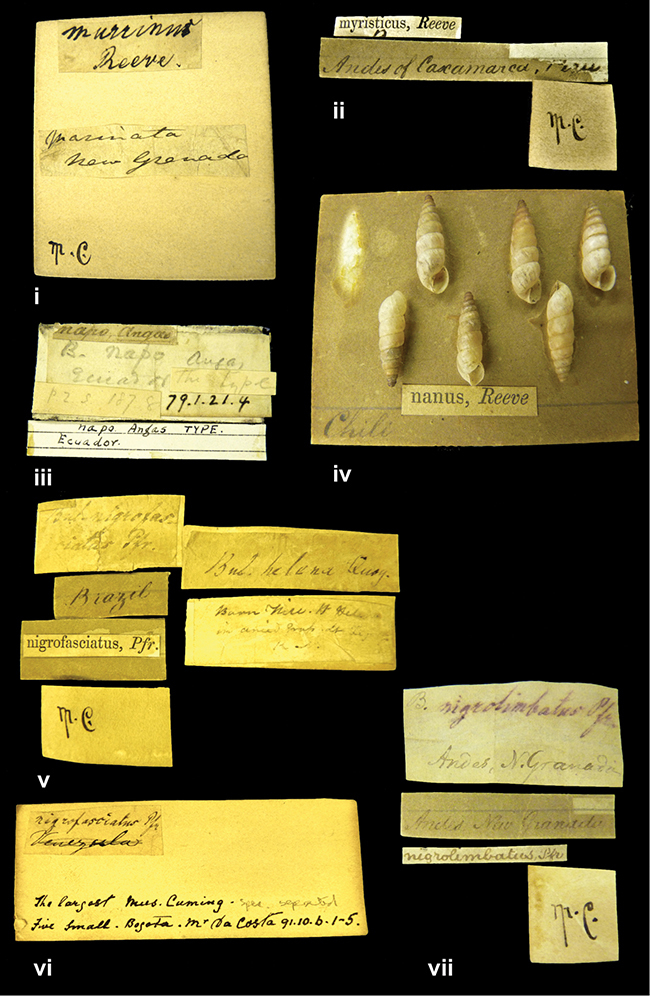
Labels. **i** *Bulimus murrinus* Reeve, 1848 **ii** *Bulimus myristicus* Reeve, 1849 **iii** *Bulimus (Otostomus) napo* Angas, 1878 **iv** *Bulimus nanus* Reeve, 1849 **v–vi** *Bulimus nigrofasciatus* Pfeiffer in Philippi 1846 **vii** *Bulimus nigrolimbatus* Pfeiffer, 1853.

**Figure L41.**
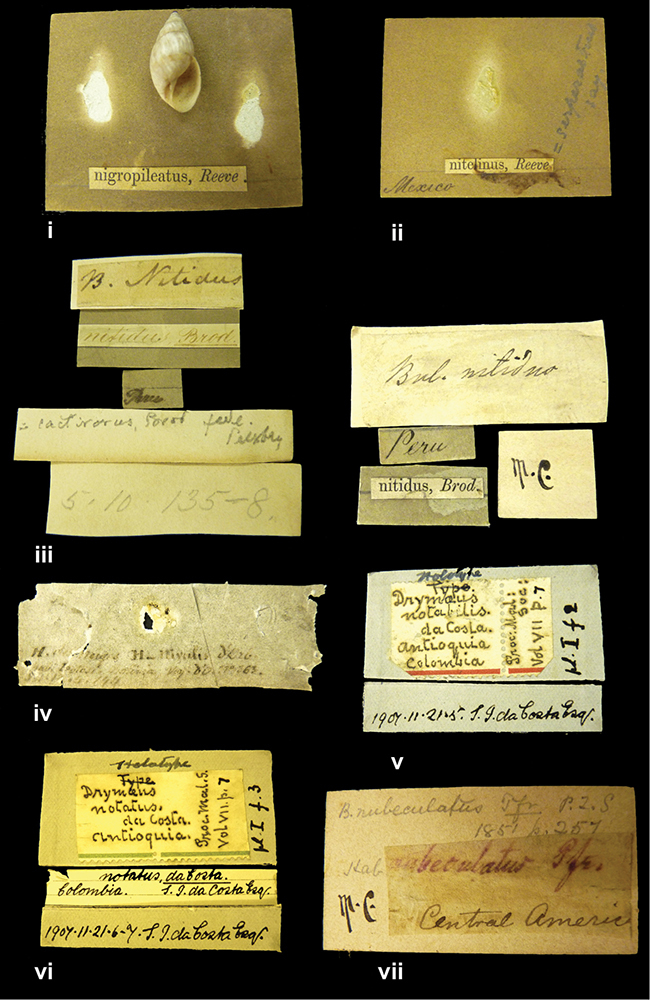
Labels. **i** *Bulimus nigropileatus* Reeve, 1849 **ii** *Bulimus nitelinus* Reeve, 1849 **iii** *Bulinus nitidus* Broderip in Broderip and Sowerby I 1832 **iv** *Helix nivalis* d’Orbigny, 1835 **v** *Drymaeus notabilis* da Costa, 1906 **vi** *Drymaeus notatus* da Costa, 1906 **vii** *Bulimus nubeculatus* Pfeiffer, 1853.

**Figure L42.**
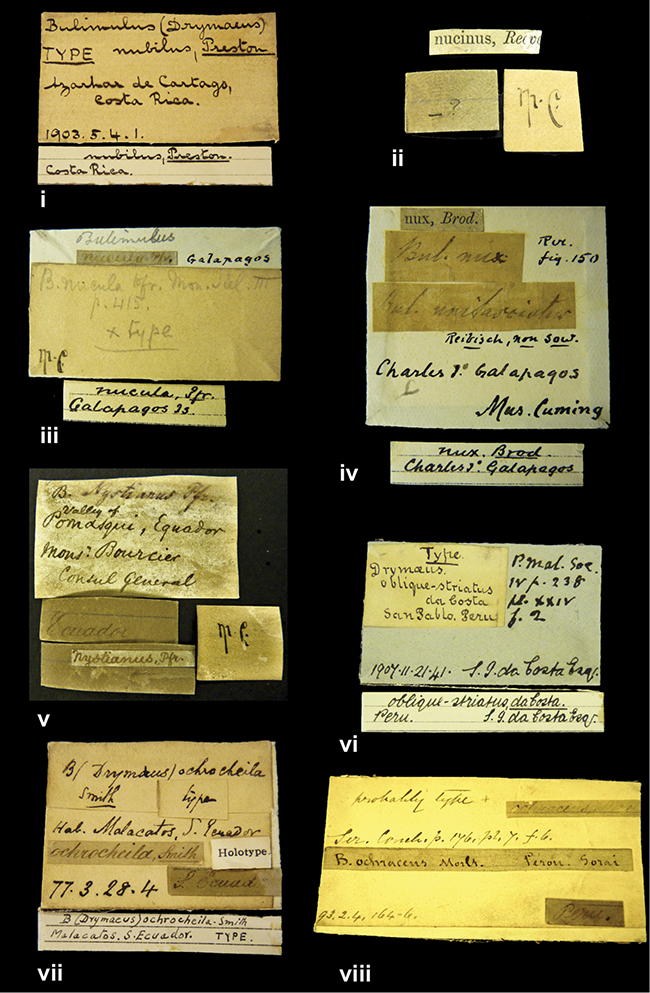
Labels. **i** *Bulimulus (Drymaeus) nubilus* Preston, 1903 **ii** *Bulimus nucinus* Reeve, 1850 **iii** *Bulimus nucula* Pfeiffer, 1853 **iv** *Bulinus nux* Broderip, 1832 **v** *Bulimus nystianus* Pfeiffer, 1853 **vi** *Drymaeus obliquistriatus* da Costa, 1901 **vii** *Bulimus (Drymaeus) ochrocheilus* E.A. Smith, 1877 **viii** *Bulimus ochraceus* Morelet, 1863.

**Figure L43.**
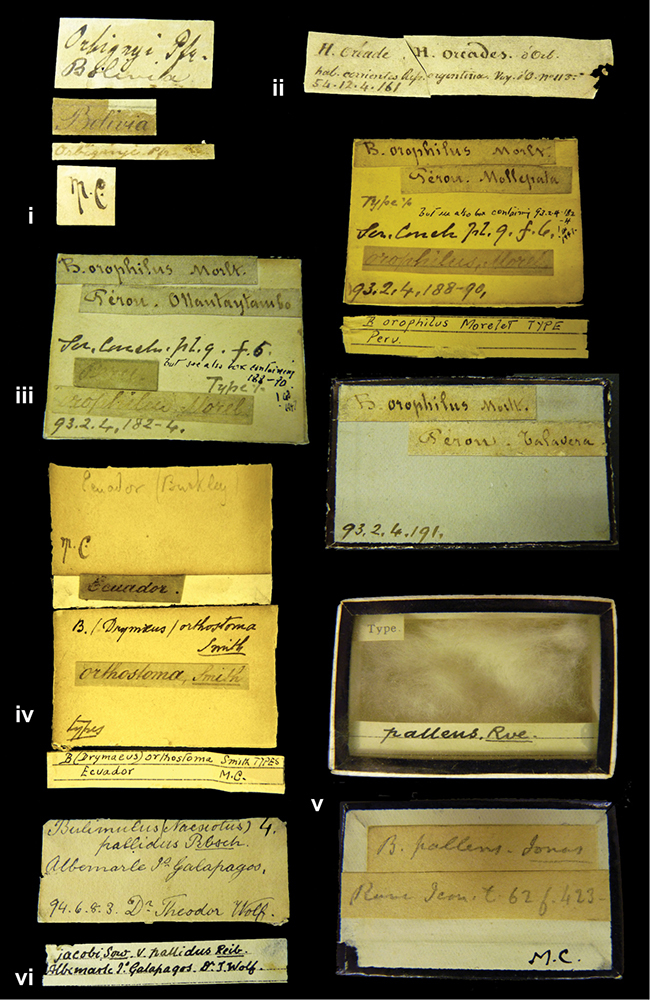
Labels. **i** *Bulimus orbignyi* Pfeiffer, 1846 **ii** *Helix oreades* d’Orbigny, 1835 **iii** *Bulimus orophilus* Morelet, 1860 **iv** *Bulimus (Drymaeus) orthostoma* E.A. Smith, 1877 **v** *Bulimus pallens* Reeve, 1849 **vi** *Bulimulus (Naesiotus) pallidus* Reibisch, 1892.

**Figure L44.**
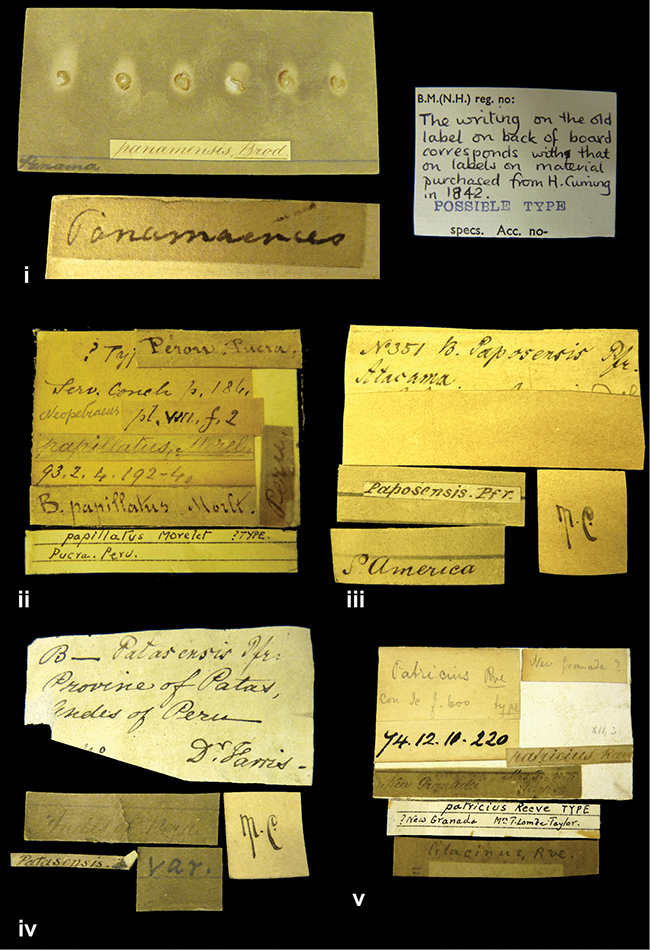
Labels. **i** *Bulinus panamensis* Broderip in Broderip and Sowerby I 1832 **ii** *Bulimus papillatus* Morelet, 1860 **iii** *Bulimus paposensis* Pfeiffer, 1856 **iv** *Bulimus patasensis* Pfeiffer, 1858 **v** *Bulimus patricius* Reeve, 1849.

**Figure L45.**
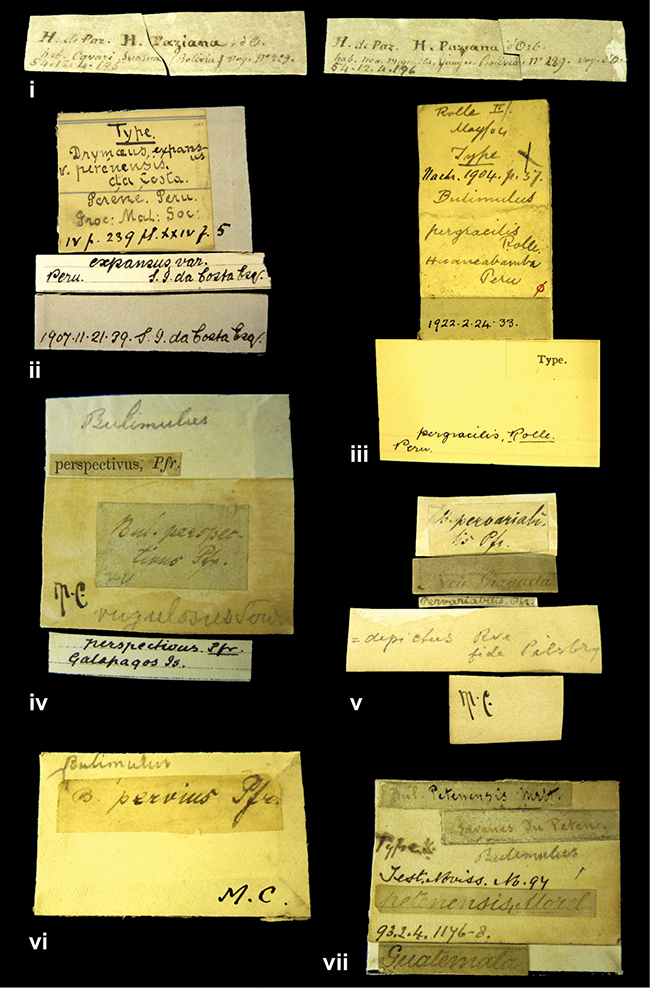
Labels. **i** *Helix paziana* d’Orbigny, 1835 **ii** *Drymaeus expansus perenensis* da Costa, 1901 **iii** *Bulimulus pergracilis* Rolle, 1904 **iv** *Bulimus perspectivus* Pfeiffer, 1846 **v** *Bulimus pervariabilis* Pfeiffer, 1853 **vi** *Bulimus pervius* Pfeiffer, 1853 **vii** *Bulimus petenensis* Morelet, 1851.

**Figure L46.**
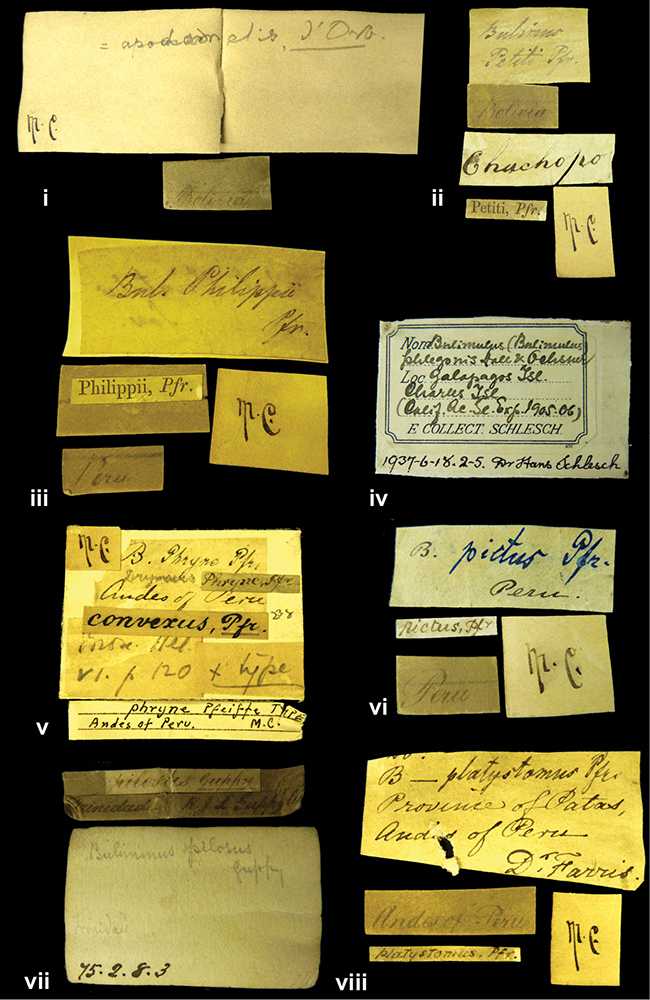
Labels. **i** *Bulimus pessulatus* Reeve, 1848 **ii** *Bulimus petiti* Pfeiffer, 1846 **iii** *Bulimus philippii* Pfeiffer, 1842 **iv** *Bulimulus (Naesiotus) ustulatus* var. *phlegonis* Dall & Ochsner, 1928 **v** *Bulimus phryne* Pfeiffer, 1863 **vi** *Bulimus pictus* Pfeiffer, 1855 **vii** *Buliminus pilosus* Guppy, 1871 **viii** *Bulimus platystomus* Pfeiffer, 1858.

**Figure L47.**
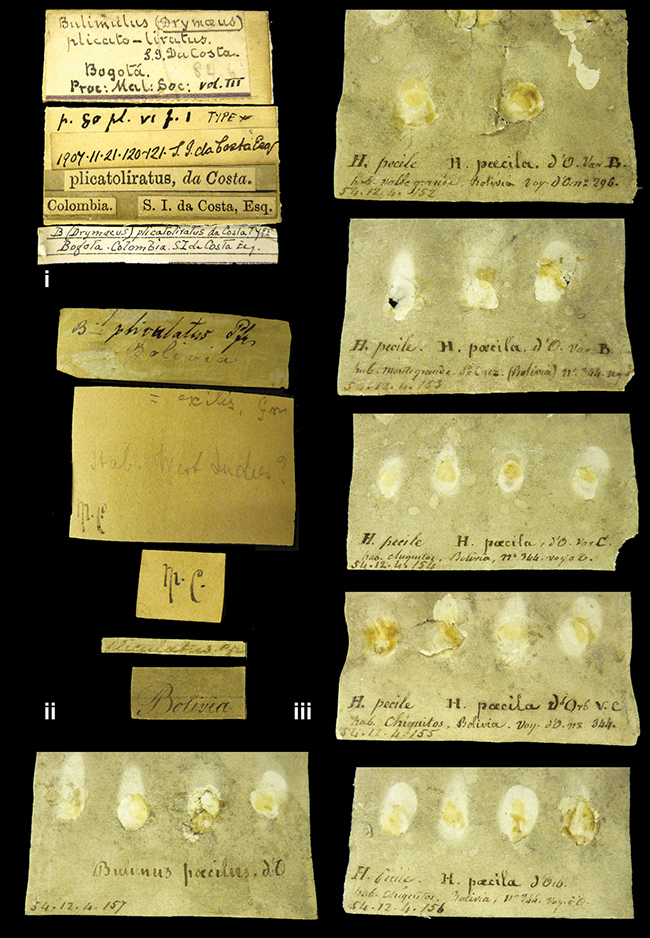
Labels. **i** *Bulimulus (Drymaeus) plicatoliratus* Da Costa, 1898 **ii** *Bulimus pliculatus* Pfeiffer, 1857 **iii** *Helix poecila* d’Orbigny, 1835.

**Figure L48.**
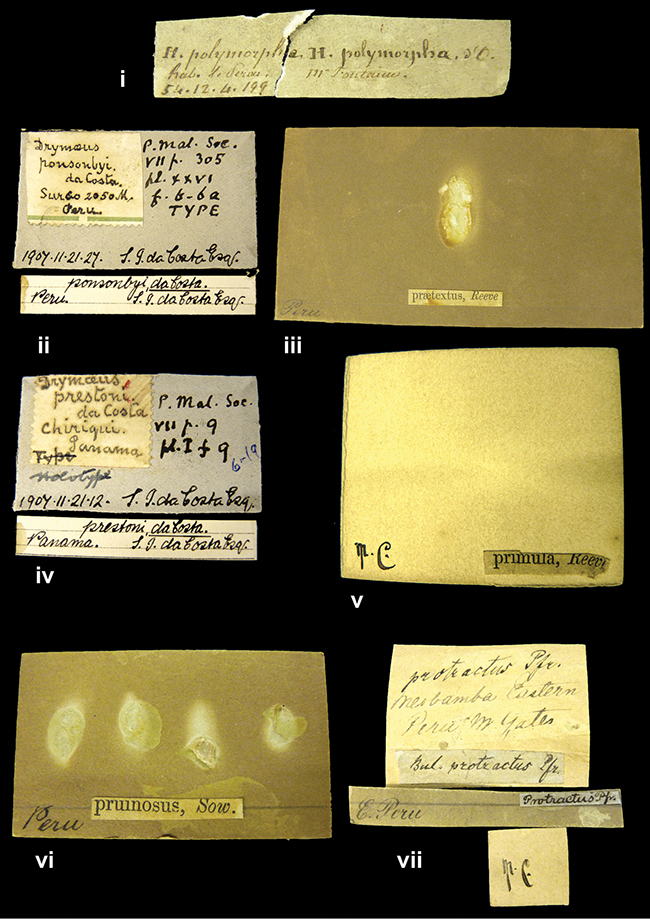
Labels. **i** *Helix polymorpha* d’Orbigny, 1835 **ii** *Drymaeus ponsonbyi* da Costa, 1907 **iii** *Bulimus praetextus* Reeve, 1849 **iv** *Drymaeus prestoni* da Costa, 1906 **v** *Bulimus primula* Reeve, 1848 **vi** *Bulinus pruinosus* Sowerby I, 1833 **vii** *Bulimus protractus* Pfeiffer, 1855.

**Figure L49.**
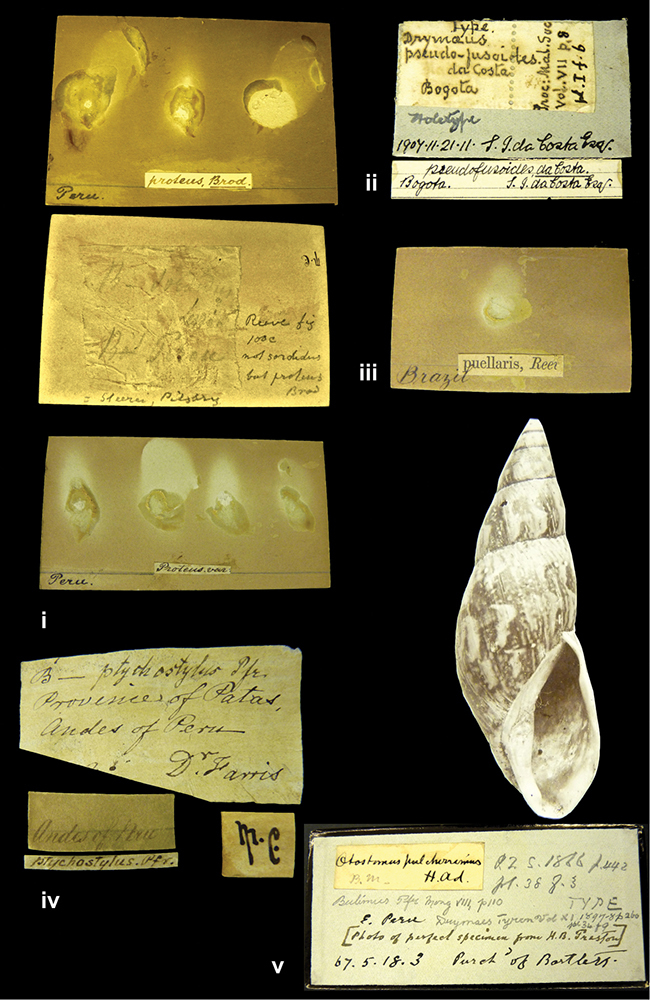
Labels. **i** *Bulinus proteus* Broderip in Broderip and Sowerby I 1832 **ii** *Drymaeus pseudofusoides* da Costa, 1906 **iii** *Bulimus puellaris* Reeve, 1850 **iv** *Bulimus ptychostylus* Pfeiffer, 1858 **v** *Otostomus pulcherrimus* H. Adams, 1867.

**Figure L50.**
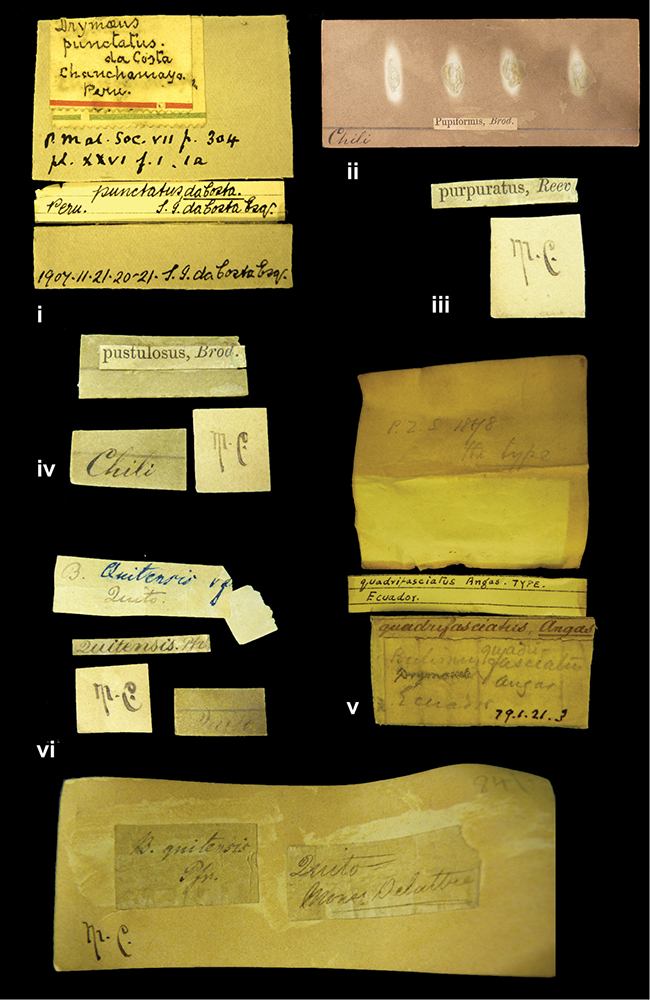
Labels. **i** *Drymaeus punctatus* da Costa, 1907 **ii** *Bulinus pupiformis* Broderip in Broderip and Sowerby I 1832 **iii** *Bulimus purpuratus* Reeve, 1849 **iv** *Bulinus pustulosus* Broderip in Broderip and Sowerby I 1832 **v** *Bulimus (Otostomus) quadrifasciatus* Angas, 1878 **vi** *Bulimus quitensis* Pfeiffer, 1848.

**Figure L51.**
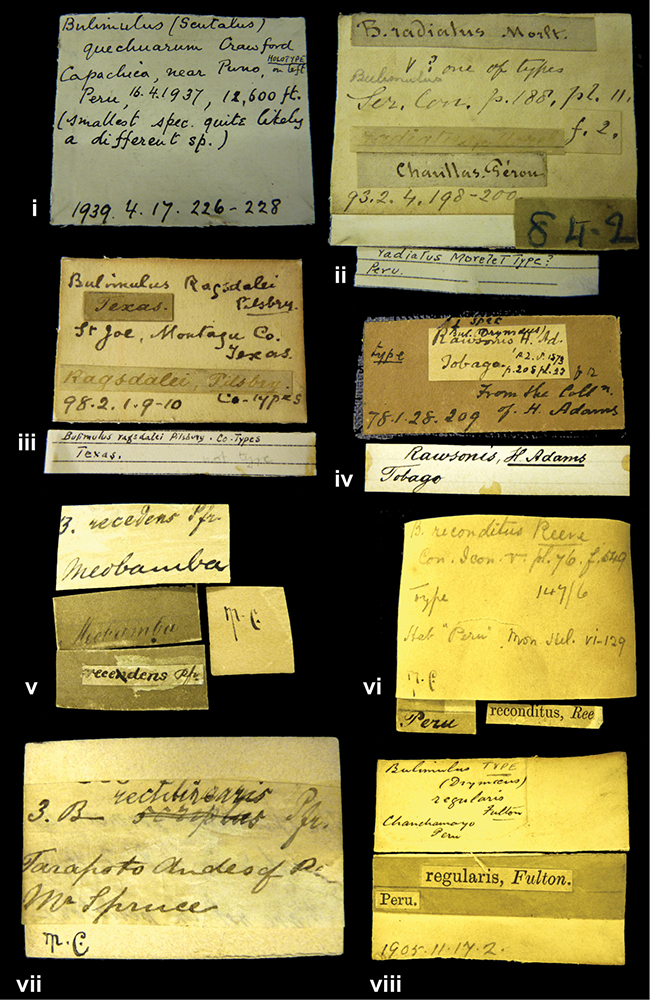
Labels. **i** *Bulimulus (Scutalus) quechuarum* Crawford, 1939 **ii** *Bulimus radiatus* Morelet, 1863 **iii** *Bulimulus ragsdalei* Pilsbry, 1890 **iv** *Bulimulus (Drymaeus) rawsonis* H. Adams, 1873 **v** *Bulimus recedens* Pfeiffer, 1864 **vi** *Bulimus reconditus* Reeve, 1849 **vii** *Bulimus rectilinearis* Pfeiffer, 1855 **viii** *Drymaeus regularis* Fulton, 1905.

**Figure L52.**
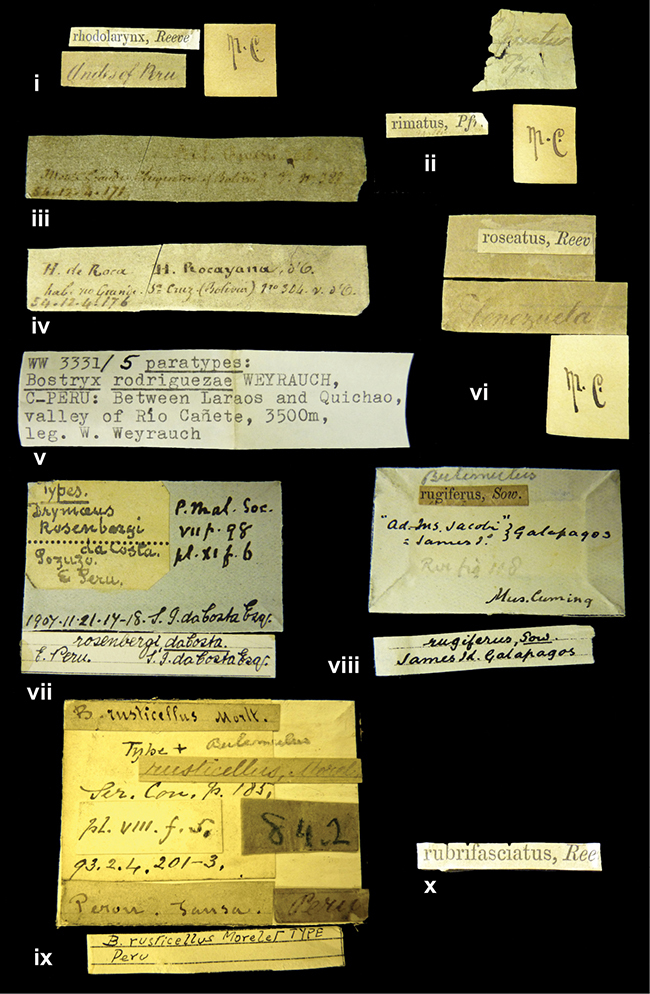
Labels. **i** *Bulimus rhodolarynx* Reeve, 1849 **ii** *Bulimus rimatus* Pfeiffer, 1847 **iii** *Bulimus rivasii* d’Orbigny, 1837 **iv** *Helix rocayana* d’Orbigny, 1835 **v** *Bostryx (Bostryx) rodriguezae* Weyrauch, 1967 **vi** *Bulimus roseatus* Reeve, 1848 **vii** *Drymaeus rosenbergi* da Costa, 1906 **viii** *Bulinus rugiferus* Sowerby I, 1833 **ix** *Bulimus rusticellus* Morelet, 1860 **x** *Bulimus rubrifasciatus* Reeve, 1848.

**Figure L53.**
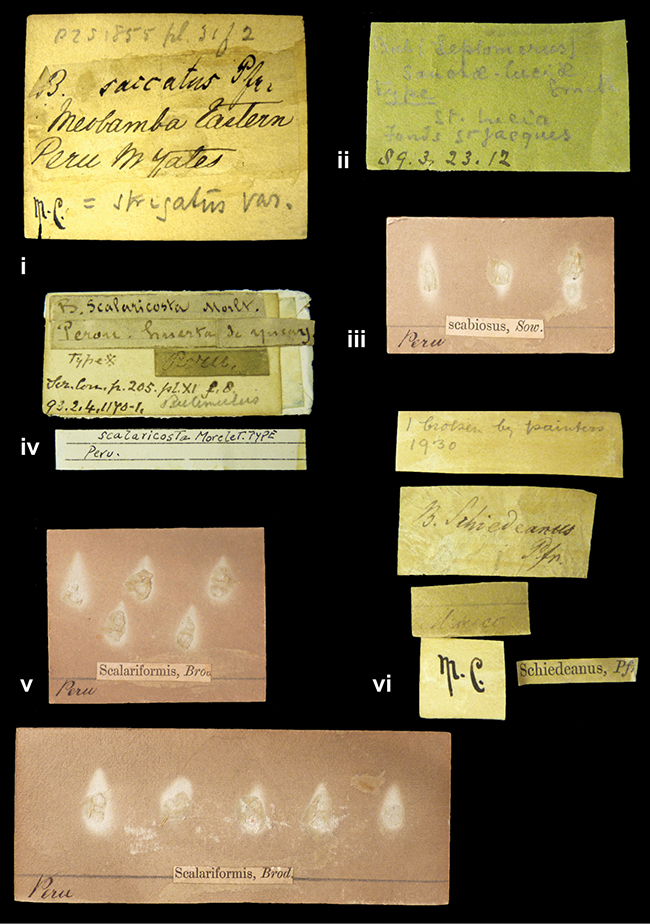
Labels. **i** *Bulimus saccatus* Pfeiffer, 1855 **ii** *Bulimus (Leptomerus) sanctaeluciae* E.A. Smith, 1889 **iii** *Bulinus scabiosus* Sowerby I, 1833 **iv** *Bulimus scalaricosta* Morelet, 1860 **v** *Bulinus scalariformis* Broderip in Broderip and Sowerby I 1832 **vi** *Bulimus schiedeanus* Pfeiffer, 1841.

**Figure L54.**
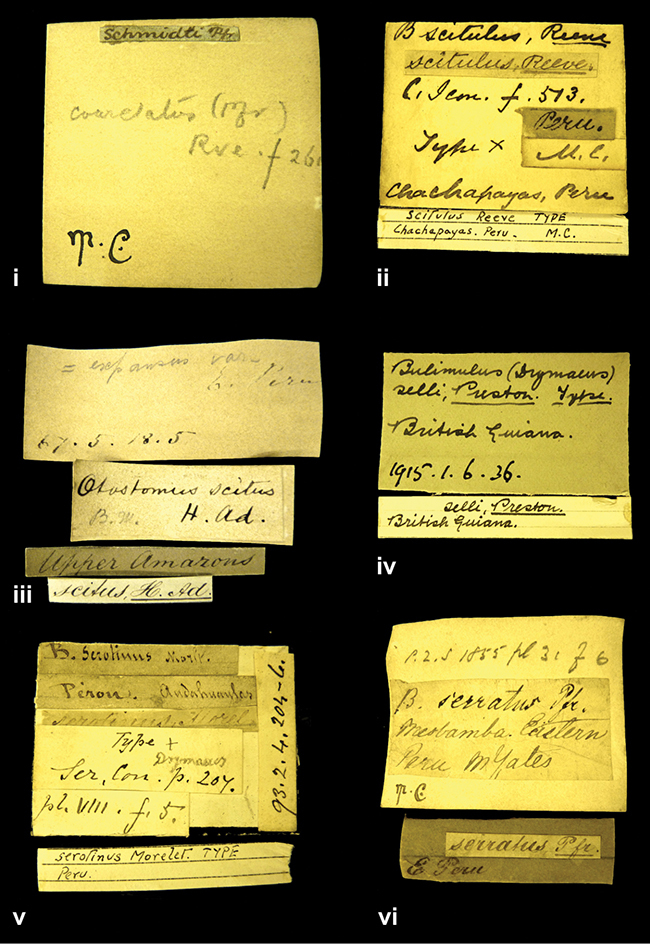
Labels. **i** *Bulimus schmidti* Pfeiffer, 1854 **ii** *Bulimus scitulus* Reeve, 1849 **iii** *Otostomus scitus* H. Adams, 1867 **iv** *Bulimulus (Drymaeus) selli* Preston, 1909 **v** *Bulimus serotinus* Morelet, 1860 **vi** *Bulimus serratus* Pfeiffer, 1855.

**Figure L55.**
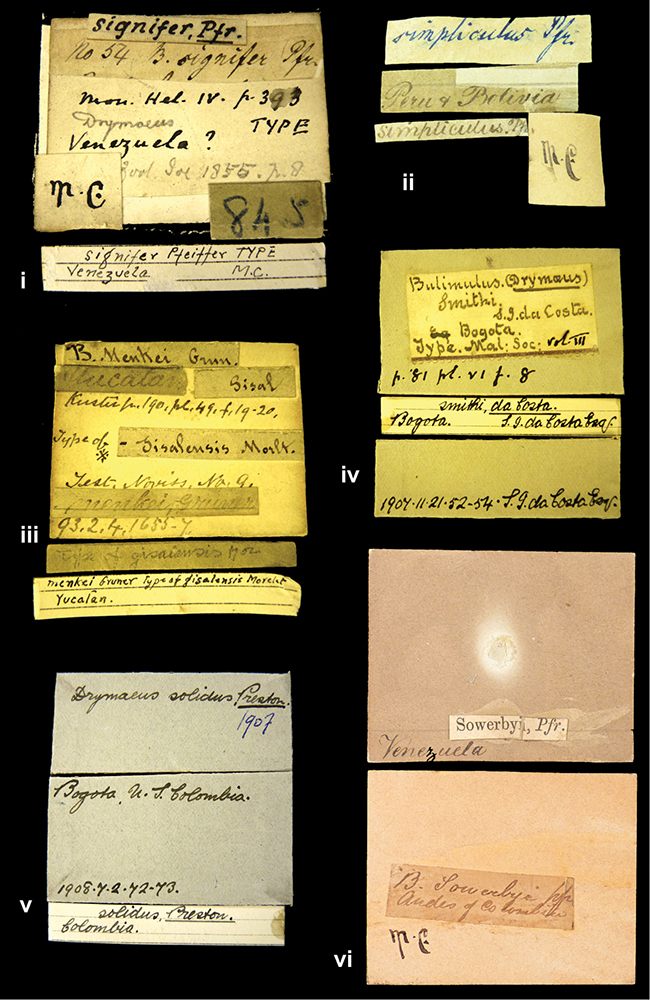
Labels. **i** *Bulimus signifer* Pfeiffer, 1855 **ii** *Bulimus simpliculus* Pfeiffer, 1855 **iii** *Bulimus sisalensis* Morelet, 1849 **iv** *Bulimulus (Drymaeus) smithii* da Costa, 1898 **v** *Bulimulus (Drymaeus) solidus* Preston, 1907 **vi** *Bulimus sowerbyi* Pfeiffer, 1847.

**Figure L56.**
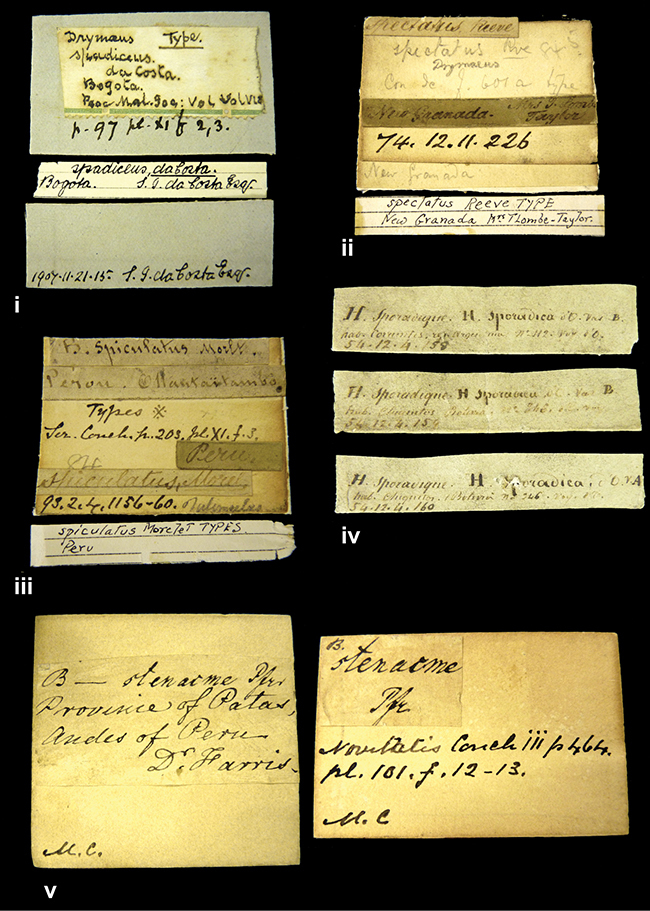
Labels. **i** *Drymaeus spadiceus* da Costa, 1906 **ii** *Bulimus spectatus* Reeve, 1849 **iii** *Bulimus spiculatus* Morelet, 1860 **iv** *Helix sporadica* d’Orbigny, 1835 **v** *Bulimus stenacme* Pfeiffer, 1857.

**Figure L57.**
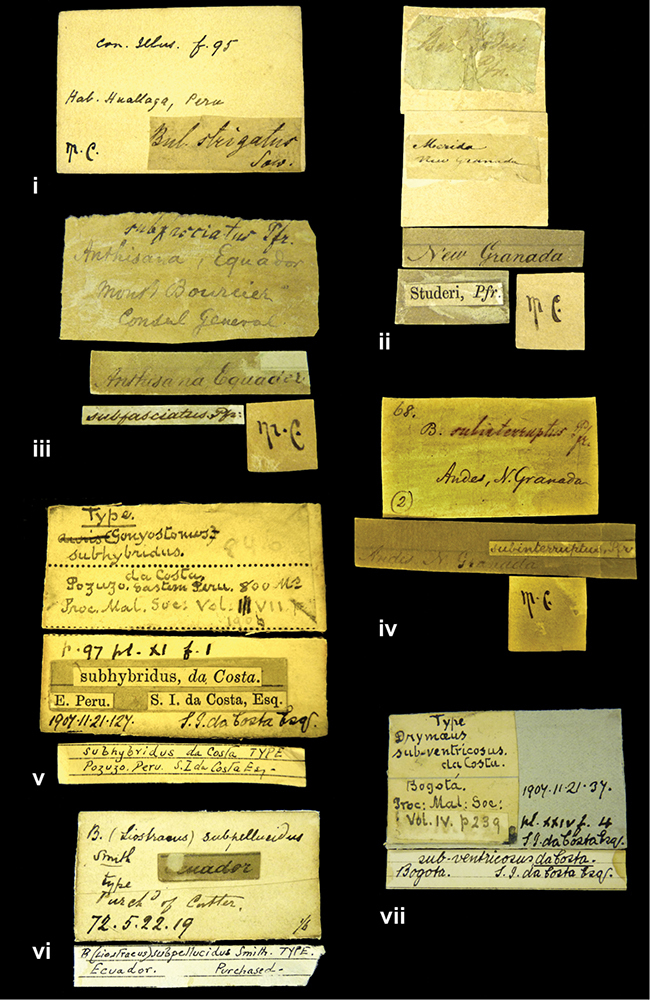
Labels. **i** *Bulinus strigatus* Sowerby I, 1833 **ii** *Bulimus studeri* Pfeiffer, 1847 **iii** *Bulimus subfasciatus* Pfeiffer, 1853 **iv** *Gonyostomus subhybridus* da Costa, 1906 **v** *Bulimus subinterruptus* Pfeiffer, 1853 **vi** *Bulimulus (Liostracus) subpellucidus* E.A. Smith, 1877 **vii** *Drymaeus subventricosus* da Costa, 1901.

**Figure L58.**
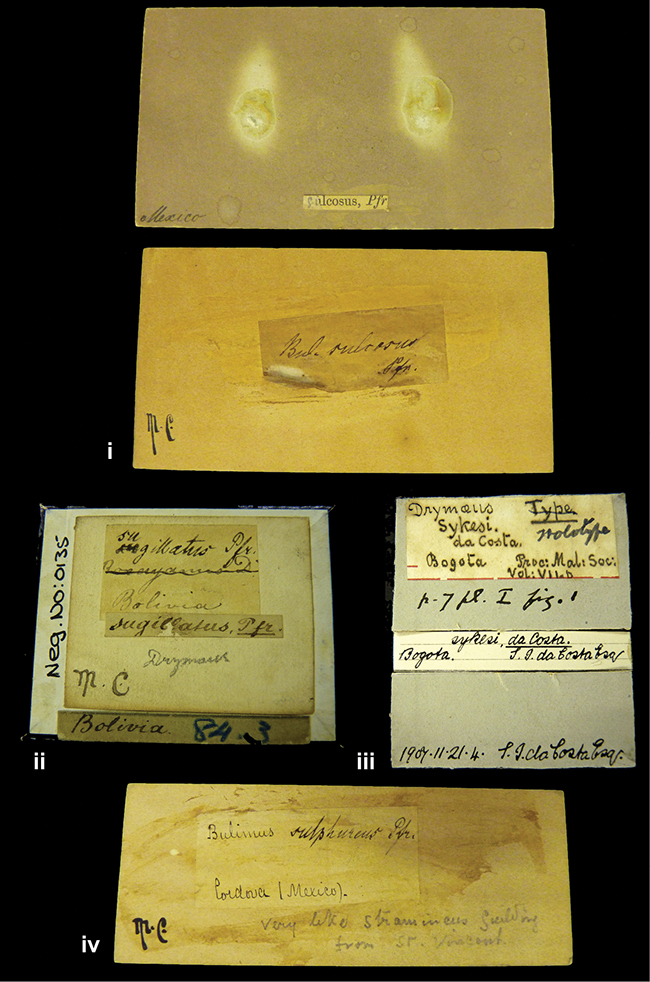
Labels. **i** *Bulimus sulcosus* Pfeiffer, 1841 **ii** *Bulimus sugillatus* Pfeiffer, 1857 **iii** *Drymaeus sykesi* da Costa, 1906 **iv** *Bulimus sulphureus* Pfeiffer, 1857.

**Figure L59.**
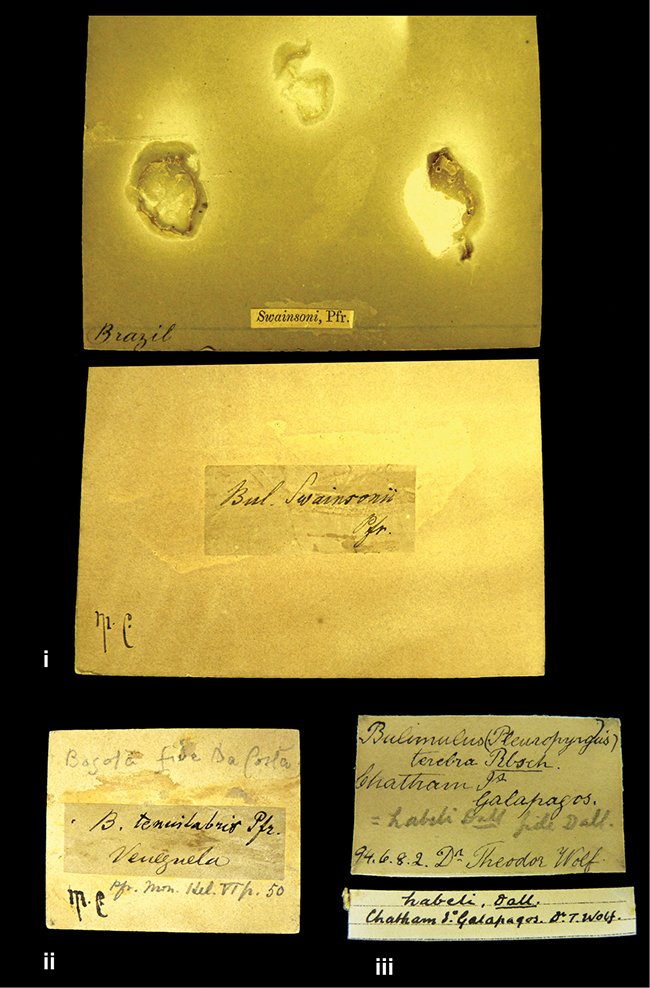
Labels. **i** *Bulimus swainsoni* Pfeiffer, 1845 **ii** *Bulimus tenuilabris* Pfeiffer, 1866 **iii** *Bulimulus (Pleuropyrgus) terebra* Reibisch, 1892.

**Figure L60.**
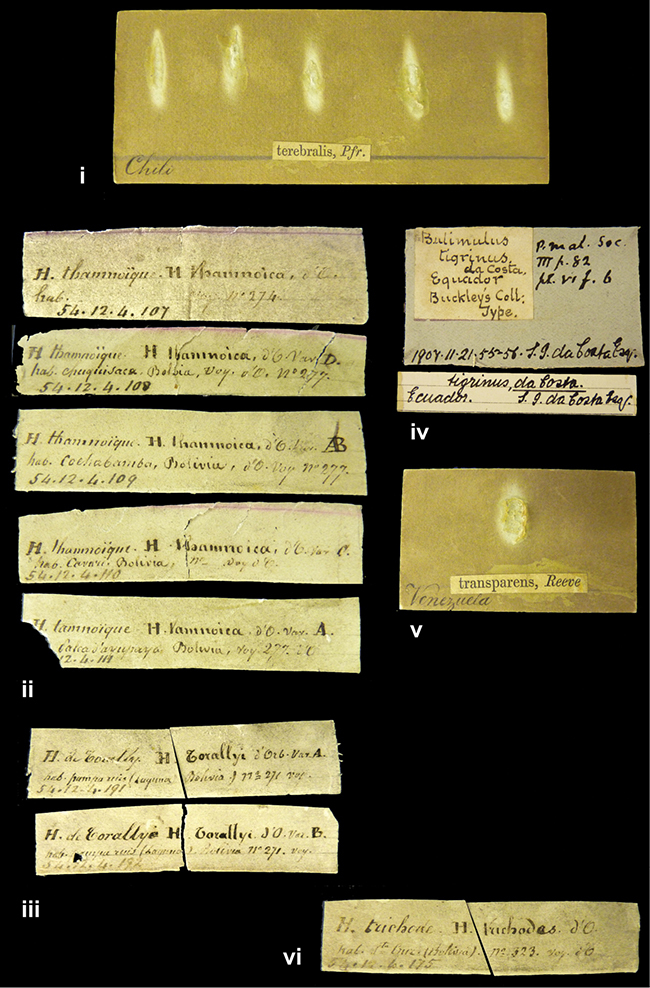
Labels. **i** *Bulimus terebralis* Pfeiffer, 1842 **ii** *Helix thamnoica* d’Orbigny, 1835 **iii** *Helix torallyi* d’Orbigny, 1835 **iv** *Bulimulus (Drymaeus) tigrinus* da Costa, 1898 **v** *Bulimus transparens* Reeve, 1849 **vi** *Helix trichoda* d’Orbigny, 1835.

**Figure L61.**
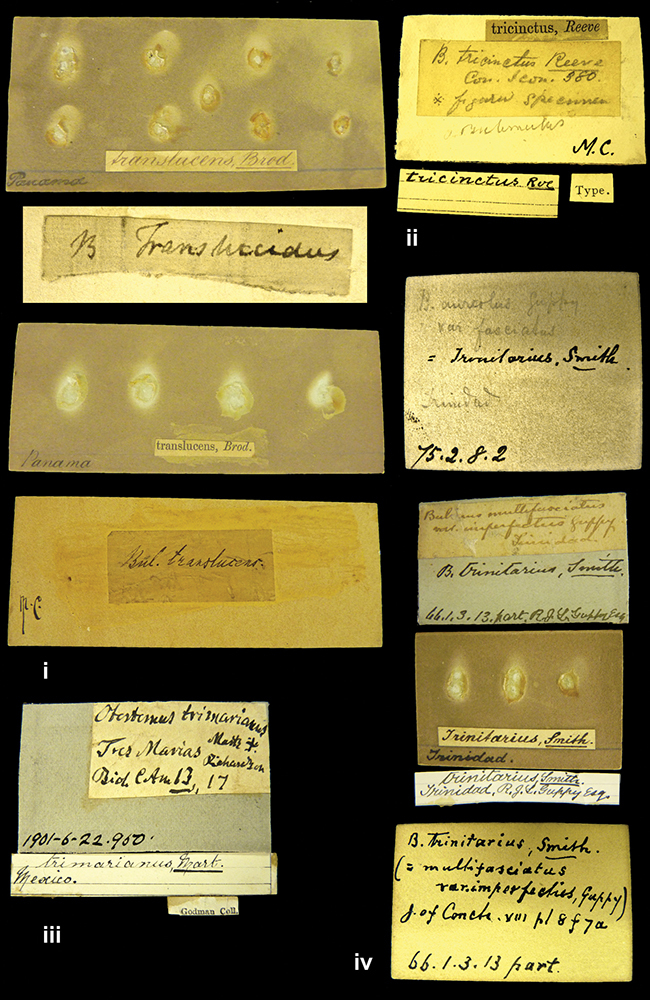
Labels. **i** *Bulinus translucens* Broderip in Broderip and Sowerby I 1832 **ii** *Bulimus tricinctus* Reeve, 1848 **iii** *Otostomus trimarianus* Martens, 1893 **iv** *Bulimulus (Drymaeus) trinitarius* E.A. Smith, 1896.

**Figure L62.**
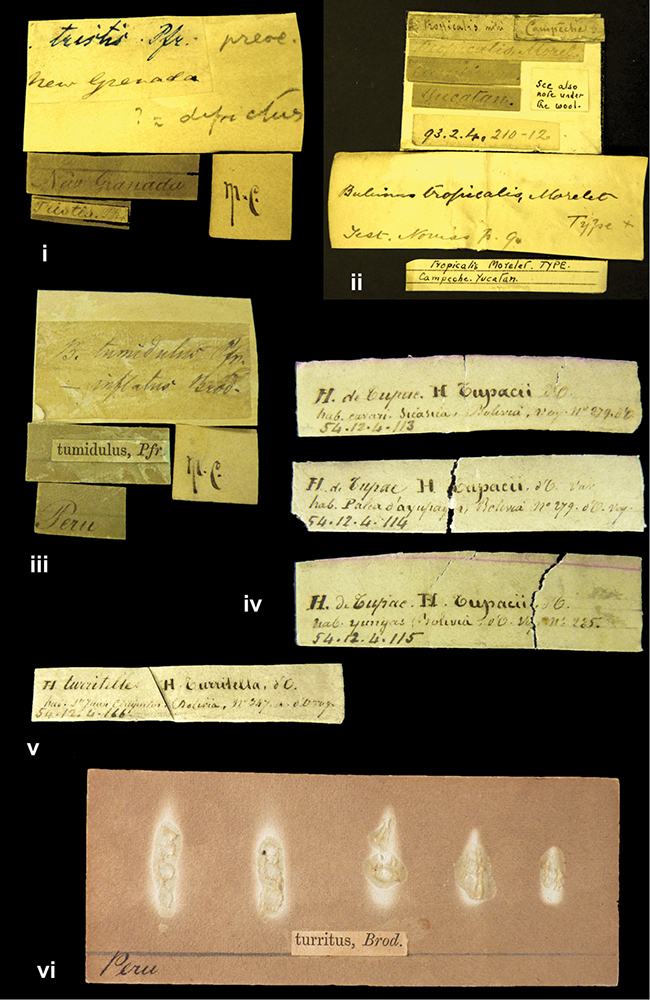
Labels. **i** *Bulimus tristis* Pfeiffer, 1855 **ii** *Bulimus tropicalis* Morelet, 1849 **iii** *Bulimus tumidulus* Pfeiffer, 1842 **iv** *Helix tupacii* d’Orbigny, 1835 **v** *Helix turritella* d’Orbigny, 1835 **vi** *Bulinus turritus* Broderip in Broderip and Sowerby I 1832.

**Figure L63.**
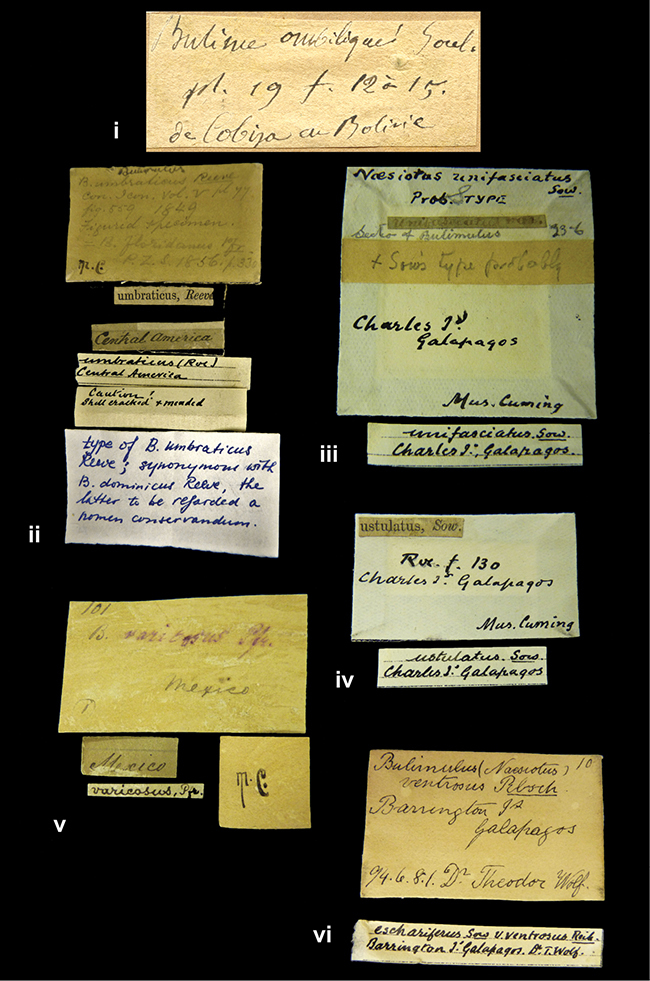
Labels. **i** *Bulimus umbilicaris* Souleyet, 1842 **ii** *Bulimus umbricatus* Reeve, 1849 **iii** *Bulinus unifasciatus* Sowerby I, 1833 **iv** *Bulinus ustulatus* Sowerby I, 1833 **v** *Bulimus varicosus* Pfeiffer, 1853 **vi** *Bulimulus (Naesiotus) ventrosus* Reibisch, 1892.

**Figure L64.**
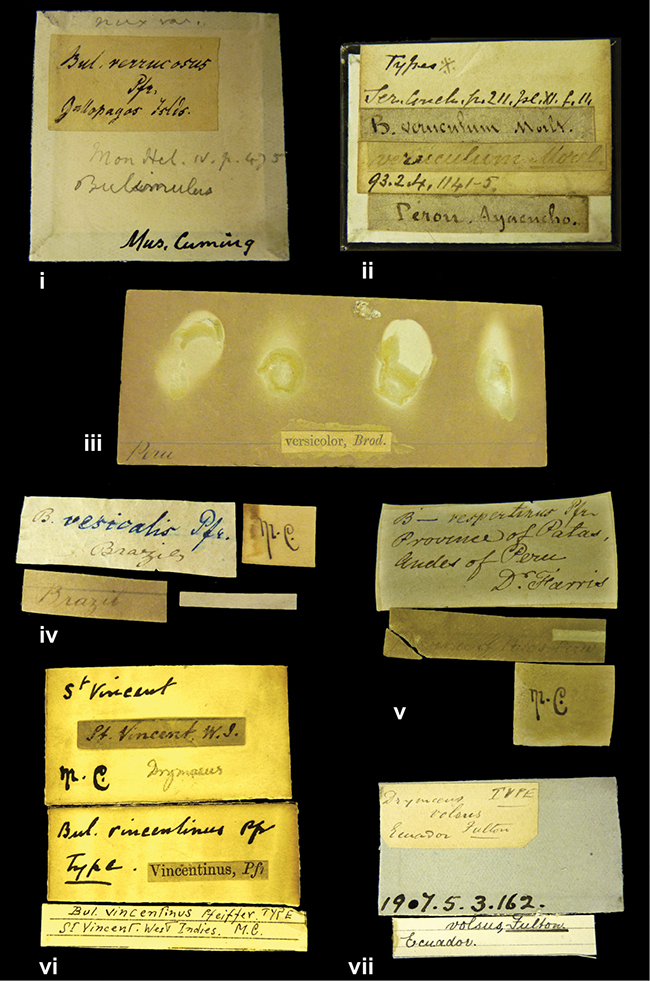
Labels. **i** *Bulimus verrucosus* Pfeiffer, 1855 **ii** *Bulimus veruculum* Morelet, 1860 **iii** *Bulinus versicolor* Broderip in Broderip and Sowerby I 1832 **iv** *Bulimus vesicalis* Pfeiffer, 1853 **v** *Bulimus vespertinus* Pfeiffer, 1858 **vi** *Bulimus vincentinus* Pfeiffer, 1846 **vii** *Drymaeus volsus* Fulton, 1907.

**Figure L65.**
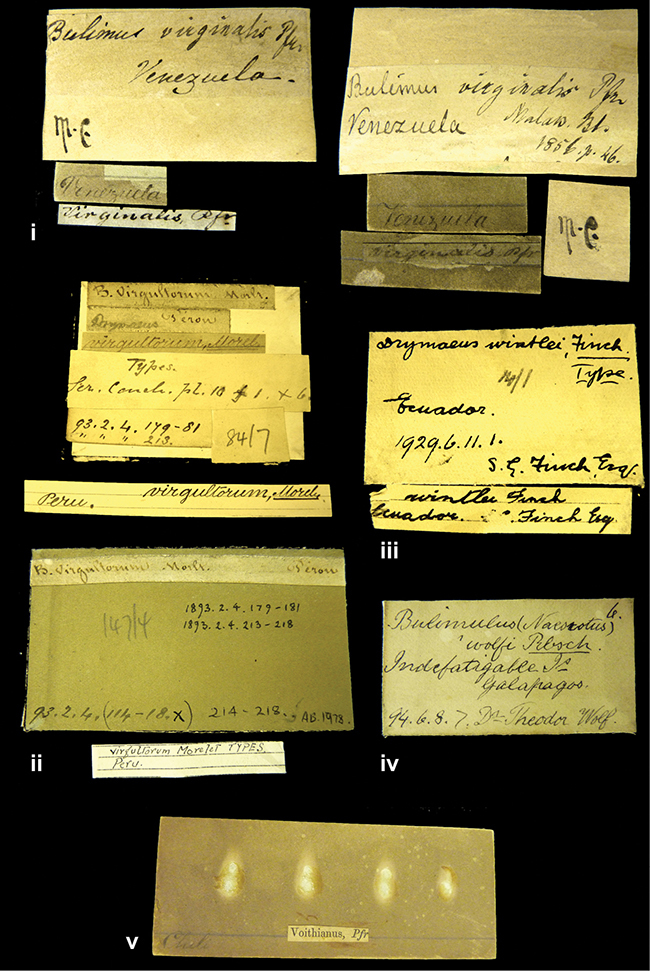
Labels. **i** *Bulimus virginalis* Pfeiffer, 1856 **ii** *Bulimus virgultorum* Morelet, 1863 **iii** *Drymaeus wintlei* Finch, 1929 **iv** *Bulimulus (Naesiotus) wolfi* Reibisch, 1892 **v** *Bulimus voithianus* Pfeiffer, 1847.

**Figure L66.**
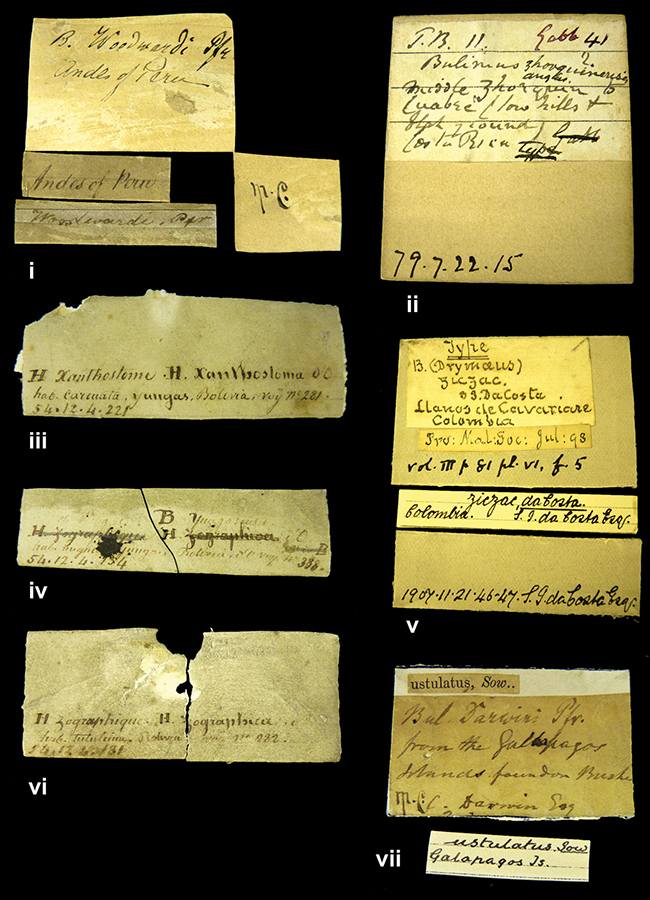
Labels. **i** *Bulimus woodwardi* Pfeiffer, 1857 **ii** *Bulimus zhorquinensis* Angas, 1879 **iii** *Helix xanthostoma* d’Orbigny, 1835 **iv** *Bulimus yungasensis* d’Orbigny, 1837 **v** *Bulimulus (Drymaeus) ziczac* da Costa, 1898 **vi** *Helix zo[o]graphica* d’Orbigny, 1835 **vii** *Bulimus darwini* Pfeiffer, 1846.

**Figure L67.**
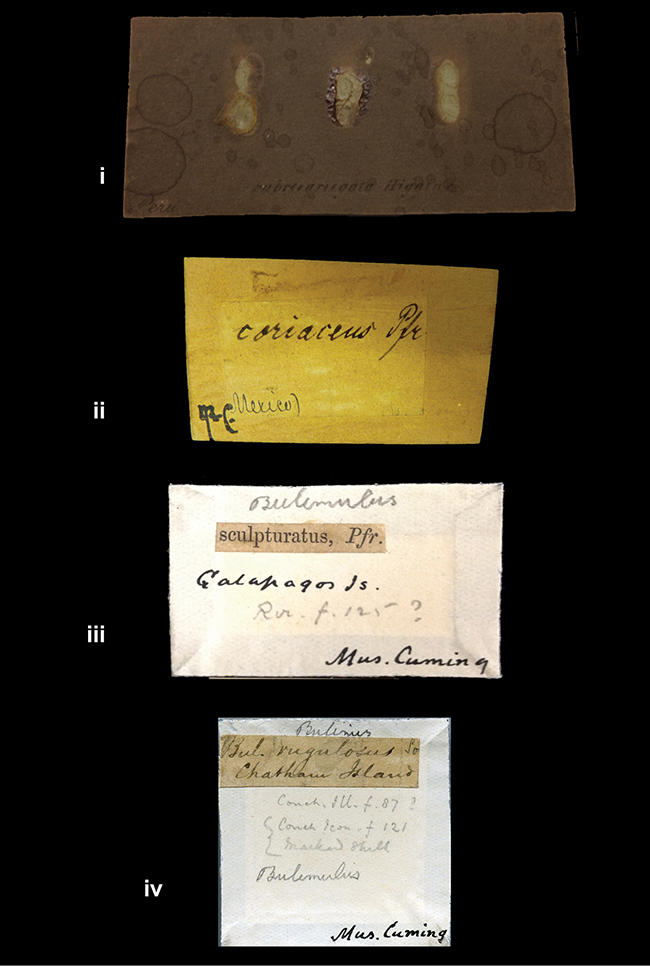
Labels. **i** *Bulimus (Otostomus) rubrovariegatus* Higgins, 1868 **ii** *Bulimus coriaceus* Pfeiffer, 1857 **iii** *Bulimus sculpturatus* Pfeiffer, 1846 **iv** *Bulinus rugulosus* Sowerby I 1833.
